# Mechanochemical disassembly pathways of self-assembled polymer-decorated Pd_n_L_2n_ supramolecular architectures

**DOI:** 10.1038/s41467-026-74561-4

**Published:** 2026-06-19

**Authors:** Tim David, Regina Lennarz, Jan A. Meissner, Anne Germann, Jan Meisner, Bernd M. Schmidt

**Affiliations:** 1 Institute for Organic Chemistry and Macromolecular Chemistry, Heinrich Heine University Düsseldorf, Düsseldorf, Germany; 2 Institute for Physical Chemistry, Heinrich Heine University Düsseldorf, Düsseldorf, Germany

**Keywords:** Self-assembly, Molecular capsules, Organic molecules in materials science

## Abstract

Supramolecular Pd_n_L_2n_ architectures are versatile molecular platforms with applications spanning catalysis, sensing, and therapeutic delivery. Whereas their thermodynamically driven assembly has been extensively studied, controlled strategies for disassembly remain scarce. Here, we show that ultrasound provides a powerful stimulus to trigger and probe disassembly pathways. Polymer-decorated ligands were synthesised to generate Pd_n_L_2n_ assemblies bearing appended polymer chains. Under ultrasound, shear forces transmitted through these chains apply mechanical stress to the square-planar palladium complexes, leading to ligand dissociation. Using Pd_2_L_4_ cages, we explore the disassembly pathways and demonstrate ultrasound-triggered release of the anticancer drug cisplatin. Furthermore, we demonstrate that quantitative and entirely reversible activation can be achieved through solid-state ball milling. To illustrate the generality of this approach, we extend it to a Pd_12_L_24_ nanosphere, one of the heaviest discrete self-assembled macromolecules reported (133 kDa), and achieve its controlled disassembly. Furthermore, ramped steered molecular dynamics simulations employing a tailored machine-learning interatomic potential reveal the force thresholds for Pd–N bond rupture and a stepwise disassembly pathway. This work establishes ultrasound as a broadly applicable strategy to access otherwise inaccessible kinetic pathways in supramolecular chemistry, with potential for applications in drug delivery and responsive materials.

## Abstract (web summary)

Supramolecular Pd_n_L_2n_ architectures are versatile molecular platforms but controlled strategies for disassembly remain scarce. Here, the authors show that ultrasound provides a powerful stimulus to trigger and probe disassembly for Pd_n_L_2n_ macromolecules bearing appended polymer chains.

## Introduction

Coordination-driven self-assembly has enabled the construction of a wide range of discrete metallosupramolecular architectures with increasing structural complexity, from compact molecular containers towards giant spherical assemblies^1–4^. Their well-defined three-dimensional cavities with tuneable size and several possibilities for endo- and exohedral functionalisation^5,6^ have proven them exceptionally versatile for a variety of applications, including stimuli-responsive smart materials^7,8^, fusion with soft matter applications^9,10^, drug delivery^11,12^, regio- and stereoselective catalysis^13–15^, nanoreactors^16,17^, and the stabilisation of reactive species^18–21^. Among the different classes of metal-organic assemblies, Pd_n_L_2n_-type structures stand out due to their well-understood self-assembly behaviour^22^, their structural predictability^23,24^, and use in catalysis^25–28^. Their self-assembly from Pd^2+^ ion precursors in combination with different ditopic N-donor ligands exhibiting different bend angles and functionalisation is a versatile method to produce functional cages from small Pd_2_L_4_-type structures towards giant Pd_48_L_96_-type molecular spheres, as shown by Clever^29^, Fujita^30^, and others^31^. In particular, Hiraoka et al. have studied the self-assembly of Pd_n_L_2n_-type metal-organic-cages (MOCs) in great detail, showing that while the process eventually produces well-defined structures, it goes through a network of competing pathways and transient intermediates that coexist in dynamic equilibrium^32–34^. While palladium-based coordination cages are typically valued for their geometric precision, thermodynamic stability, and versatile applications, their dynamic metal-ligand bonds enable structural rearrangements^35^, cage-to-cage transformations by ligand exchange^36^ and stimuli-responsiveness to mechanical stress^37^. Several metal-ligand systems, such as N-heterocyclic carbene complexes with Ag^38,39^, Ru^39^, and Cu^40^, as well as ferrocene^41,42^, ruthenocene^41^, and Pd–N donor bonds^10,37^, have been discovered as mechanophores in recent years, demonstrating that coordination chemistry can also be used to trigger mechanical function. In addition to metal-ligand systems, numerous organic mechanophores have shown great potential for various applications, from the activation and release of drugs^43,44^ to molecular sensors^45^ towards catalysts^39^. Among these, pioneering work by Göstl and Herrmann has shown that disulfide-based mechanophores embedded in polymer chains can be selectively cleaved by ultrasound^46^, generating reactive thiol intermediates that initiate subsequent reaction cascades, enabling controlled release of drugs for cancer therapy in combination with fluorophores^47,48^. Recent studies demonstrated that microbubbles with mechanoresponsive polymer shells can accelerate this process as well as other mechanochemical activations of several mechanophores^49–51^. Beyond covalent systems, supramolecular architectures (e.g., knots^52^, rotaxanes^53–56^, and MOCs^10,37^) themselves can act as mechanophores or be integrated into polymeric structures, as shown by Leigh, De Bo, and us. All the aforementioned mechanophores are typically activated in solution by ultrasound, which generates strong local shear forces through acoustic cavitation that draw nearby polymer chains toward the collapsing cavitation bubble centre, selectively targeting the force-sensitive moieties of the embedded mechanophores^57–61^. Parallel to experimental advances in mechanochemistry, computational tools have been developed to enable ab initio investigations of force-induced reactions. This is achieved by incorporating an external potential term modelling the mechanical force in addition to the established Born-Oppenheimer potential energy surface, resulting in a force-modified potential energy surface (FM-PES)^62,63^. On this FM-PES, reaction path optimisations and ab initio steered molecular dynamics (AISMD) simulations can provide insight into mechanochemical reactivity, revealing especially the importance of dynamic effects^64–67^. The most significant drawback of AISMD, however, is the comparatively long computational time, which limits the simulation timescale and system size. This has restricted the investigation of force-modified reaction dynamics to mechanophores based on small molecular motifs. While classical force field potentials enable the simulation of very large systems, they do not allow the breakage or formation of bonds, making them unsuited for the study of chemical reactions. Reactive force fields can circumvent this restriction, but their parametrisation is complex and requires considerable expertise^68^. In recent years, machine learning interatomic potentials (MLIPs) have emerged as a powerful alternative for reactive simulations^69,70^. Universal MLIPs such as MACE^71^ and FairChem’s Universal Models for Atoms (UMA)^72^, trained on large-scale ab initio datasets, can reach accuracy comparable to the quantum chemical training data within their training domain but orders of magnitude faster. This is rapidly expanding the scope of systems that can be studied, enabling large-scale reactive dynamics over extended time scales.

## Results and Discussion

### Synthesis and characterisation of polymer-decorated metal-organic cages

**Fig. 1:**
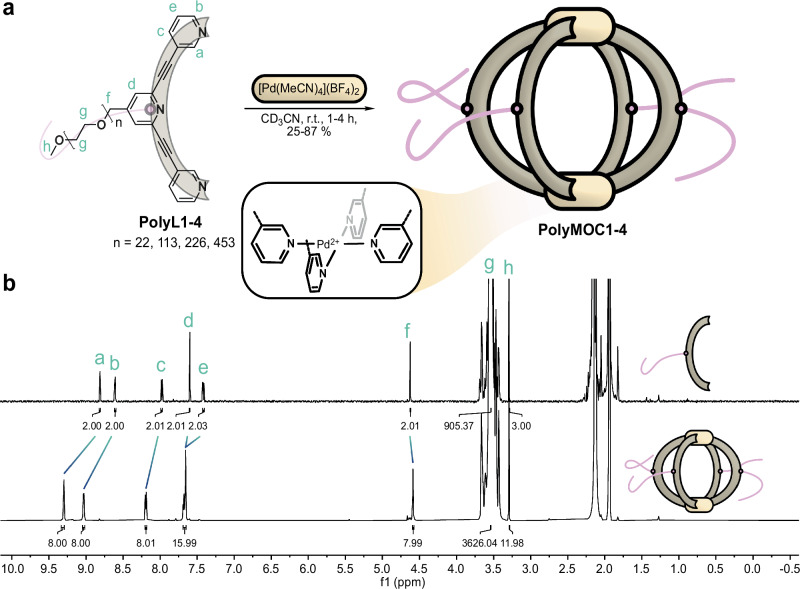
Synthesis and characterisation of metallosupramolecular mechanophores. **a** Synthetic overview for polymer-decorated metal-organic cages **PolyMOC1-4**. **b** Stacked ^1^H NMR spectra (600 MHz, CD_3_CN, 298 K) of **PolyL3** (top) and **PolyMOC3** (bottom).

Herein, we present the synthesis of Pd_2_L_4_-type polymer-decorated metal-organic cages (PolyMOCs), differing in the length of the attached polyethylene glycol monomethyl ether (mPEG) chain (**PolyMOC1-4**). PEG was specifically selected due to its superior solubility in water and organic solvents and excellent biocompatibility. For the ligand design, we chose the 2,6-bis(pyridine-3-ylethynyl)pyridine because of its well-known self-assembly behaviour when reacted with tetrakis(acetonitrile)palladium(II) tetrafluoroborate. Four isostructural polymeric ligands **PolyL1-4** can be obtained from (2,6-bis(pyridin-3-ylethynyl)pyridin-4-yl)methanol^6^ (**L3**) by using an Appel reaction to convert the alcohol functionality into benzyl bromide (**4**) in high yields. Following this, gram-scale synthesis of **PolyL1-4** was achieved in respective yields of 89%, 83%, 84% and 25% via Williamson ether synthesis using poly(ethylene glycol) monomethyl ethers with a different number of repeating units of 22, 113, 226, and 453 and corresponding average molecular weights of 1, 5, 10, and 20 kDa, respectively. **PolyL1** and **PolyL2** with a respective molecular weight of approximately 1 and 5 kDa were characterised using ^1^H, ^13^C, and further 2D NMR spectroscopy techniques, as well as matrix-assisted laser desorption mass spectrometry (MALDI-MS), and Fourier-transformation infrared spectroscopy (FT-IR) (Supplementary Figs. S148–155, S162–168). The analysis of polymeric compounds becomes more challenging as the polymer length and the molecular weight increases. Nevertheless, we could achieve meaningful ^1^H-^13^C HSQC and ^1^H-^13^C HMBC correlation spectra in addition to the ^1^H NMR spectra, FT-IR spectrum, and the MALDI-MS spectrum for **PolyL3** equipped with a 10 kDa mPEG (Supplementary Figs. S173–179). In the case of **PolyL4**, which is appended by a 20 kDa mPEG chain, the polymeric ligand could be analysed by ^1^H NMR techniques, including ^1^H-^1^H COSY and NOESY measurements, FT-IR, and MALDI-MS (Supplementary Figs. S184–188). Complete conversion of the mPEG could not be achieved due to the low reactivity, hampered diffusion, and increased viscosity of the 20 kDa mPEG in solution. The unreacted mPEG could not be separated from the product mixture because they are nearly identical in size, solubility, and polarity due to the polymer chain, resulting in a 1:2 mixture of **PolyL4** and residual mPEG. The polymer-decorated ligands **PolyL1–4** readily self-assembled into the corresponding Pd_2_L_4_-type polymeric cages **PolyMOC1–4** upon combination with tetrakis(acetonitrile)palladium(II) tetrafluoroborate in acetonitrile at room temperature (Fig. 1). **PolyMOC1**, a wax-like solid at room temperature, was obtained in 87% yield. Precipitation into cooled diethyl ether afforded **PolyMOC2** and **PolyMOC3** as colourless solids in 84% and 77% yield, respectively. The ^1^H NMR spectra of the polymeric cage structures show a single set of sharp signals that differ only in the integral of the signals corresponding to the polymer chain appended, indicating the formation of well-defined species (Supplementary Figs. S156, 169, 180, 189). Because of its electron-withdrawing effect, coordination to Pd^2+^ ions causes a downfield shift of all proton resonances compared to the corresponding signals in the spectra of the non-coordinated ligands, with the most noticeable deshielding seen for protons H_a_ and H_b_ (Δδ = ~0.45 ppm) that are located next to the coordination sites (Fig. 1). Notably, increasing the mPEG chain length resulted in prolonged assembly times, with reactions requiring one hour for **PolyMOC1-3** and up to four hours for **PolyMOC4**. Because the ligand precursor **PolyL4** was used as a mixture containing residual unreacted mPEG, the self-assembly afforded a mixture of **PolyMOC4** and excess mPEG. Given that four ligands are incorporated per cage, unreacted mPEG accumulates in the final mixture, leading to an overall **PolyMOC4** to mPEG ratio of 1:16 (see Supplementary Information, Fig. S184, S189). The successful formation of **PolyMOC1-4** was corroborated through 2D ^1^H NMR correlation experiments (COSY and NOESY) and unambiguously confirms the successful self-assembly of the gigantic polymeric cage architectures.

### Ultrasound-triggered disassembly of PolyMOCs

**Fig. 2:**
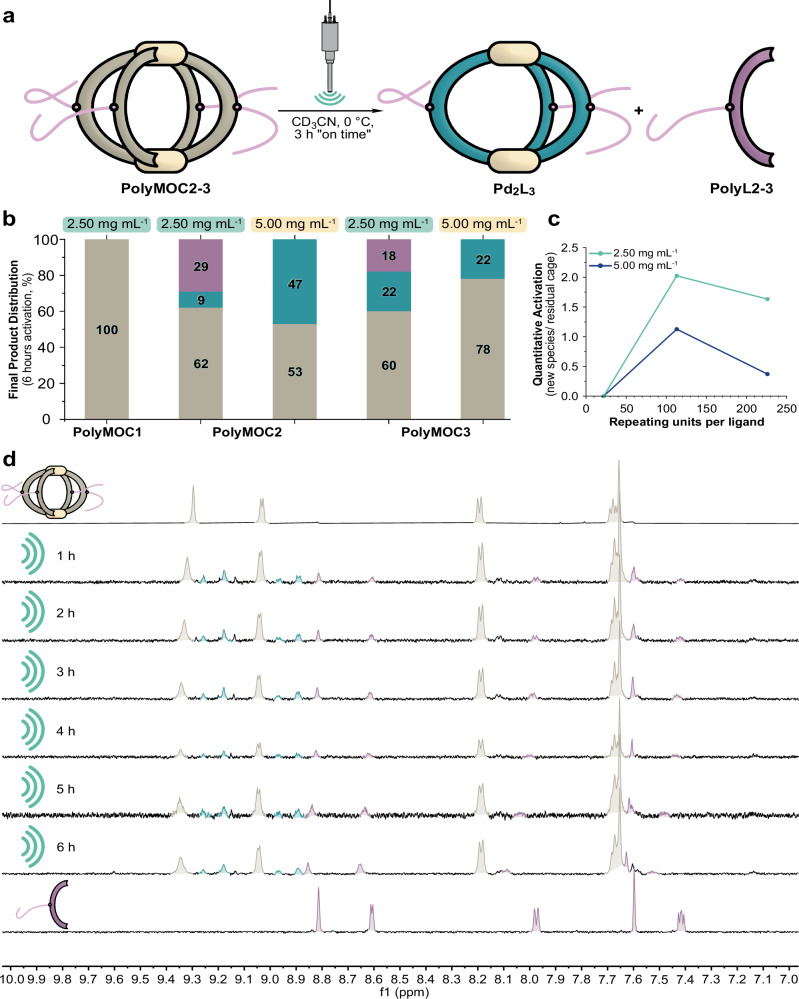
Sonochemical activation of the metallosupramolecular mechanophores. **a** Schematic overview of the activation of metallosupramolecular mechanophores (**PolyMOC2-3**) activation using nitrogen as saturation gas. **b** Final product distributions after 6 h of sonochemical activation of **PolyMOC1-3** at the indicated polymer mass concentrations, shown as percentages of intact PolyMOC (brown), Pd_2_L_3_ species (turquoise), and free PolyL (purple). **c** Quantitative activation (new species per residual PolyMOC) against polymer chain length at 2.50 mg mL^−1^ (turquoise) and 5.00 mg mL^−1^ (blue) during sonochemical activation. **d** Stacked ^1^H NMR spectra (600 MHz, CD_3_CN, 298 K) of **PolyMOC3** (top), after each hour of sonochemical activation (middle), and **PolyL3** (bottom).

To explore the mechanical responsiveness of the polymer-decorated Pd_2_L_4_-type cages, we subjected **PolyMOC1-3** to ultrasound irradiation in solution. Upon ultrasound irradiation, the polymer chains translate cavitation-induced shear forces toward the cage structure, mechanically activating the Pd–N bonds and triggering the disassembly, which is modulated by the chain length, solvent properties, concentration, and the saturation gas. The ultrasound-induced disassembly was monitored over six hours using hourly ^1^H NMR aliquots. To assess the intrinsic stability of the non-polymeric cage **MOC3**, we first subjected it to ultrasound in acetonitrile under nitrogen. The spectra showed no changes compared to the initial sample, confirming that the cage architecture remains intact under these conditions (Supplementary Fig. S27). In contrast, ultrasound treatment of the polymeric ligand **PolyL3** produced a new resonance at 2.75 ppm, indicative of alcohol end groups from PEG backbone cleavage (Supplementary Fig. S29). To exclude dilution-induced effects, the stability of the PolyMOCs in concentrations used during sonochemical activation experiments was also verified by ^1^H NMR (Supplementary Figs. S23–25). Then, we systematically exposed **PolyMOC1-3** to ultrasound, initially under nitrogen saturation. Because diatomic nitrogen absorbs more energy during sonochemical activation than the monoatomic gas argon, which was used as a reference^73,74^, the selection of saturation gas plays a key role. In addition, the molar concentration during sonication has a strong influence on the activation efficiency. At higher concentrations and for longer polymer chains, the chains become increasingly entangled and exhibit hydrodynamic interactions, preventing them from being efficiently drawn towards the collapsing cavitation bubble centres, disfavouring activation^61^. Moreover, the rapidly increasing molecular weights of the PolyMOCs with longer polymer chains lead to significant changes in the molar concentration during sonication when the polymer mass concentration is kept constant. To systematically quantify the influence of polymer chain length and concentration, **PolyMOC1-3**, featuring 22, 113, and 226 repeating units per ligand, respectively, were subjected to ultrasound at a constant polymer mass concentration of 2.50 mg mL^−1^. In addition, **PolyMOC2** and **PolyMOC3** were further investigated using a higher polymer mass concentration of 5.00 mg mL^−1^. We quantified the activation efficiency by determining the final product distribution of free PolyL, Pd_2_L_3_ species and intact PolyMOC after six hours of sonochemical treatment. The analysis was achieved by comparing the integrals of the proton resonances corresponding to H_b_ of each individual species and dividing the amount of protons by the total sum of the corresponding proton resonances across the final mixture, thereby yielding the relative percentage of each product (see Supplementary Figs. S69–72). For **PolyMOC1**, bearing the shortest polymer chains (22 repeating units per ligand), no Pd–N bond activation was detected under either used concentration regime (Fig. 2b, Supplementary Figs. S32–36). This clearly demonstrates that the attached chains are too short to enable efficient force transduction and, consequently, fail to mechanically activate the supramolecular cage mechanophore. In contrast, **PolyMOC2** (113 repeating units per ligand), equipped with a polymer chain five times longer than **PolyMOC1**, was activated using a polymer mass concentration of 2.50 mg mL^−1^, equal to 125 µM, and exhibited a pronounced activation yielding 29% of **PolyL2**, 9% of Pd_2_L_3_ species, and 62% of intact **PolyMOC2** in the final product mixture. Increasing the polymer mass concentration to 5.00 mg mL^−1^ results in enhanced cage mechanophore activation, leaving 47% Pd_2_L_3_ species alongside 53% intact **PolyMOC2** in the final product mixture (Fig. 2b). Notably, under these more concentrated conditions, the Pd_2_L_3_ intermediates do not undergo further activation to yield free **PolyL2**. To quantitatively assess the activation efficiency, we determined the number of newly formed discrete molecular species per remaining intact cage in the final product distribution. This was achieved by normalising the integrals of the corresponding ^1^H NMR signals and relating them to the normalised integral of the intact PolyMOC signal (see Supplementary Figs. S69–72). Considering that complete dissociation of one Pd_2_L_3_ unit generates three polymer-decorated ligands upon further activation, the total number of newly formed species relative to intact cage increases when secondary activation occurs. In the case of **PolyMOC2**, doubling the polymer mass concentration decreases the number of discrete species per residual intact cage from 2.03 to 1.19 (Fig. 2c). This demonstrates that increasing the concentration not only alters the final product distribution but also significantly reduces the overall degree of disassembly. **PolyMOC3**, featuring polymer chains twice as long as those of **PolyMOC2**, was sonochemically activated at the same polymer mass concentration (2.50 mg mL^−1^). This resulted in a final product distribution of 18% free **PolyL3**, 22% Pd_2_L_3_ species, and 60% intact **PolyMOC3** (Fig. 2b). While the overall activation efficiency remained comparable to that of **PolyMOC2** under identical concentration conditions, the distribution of the newly formed species shifted significantly. For **PolyMOC2**, the initially generated Pd_2_L_3_ intermediates undergo almost quantitative subsequent activation, producing 29% non-coordinated **PolyL2** in the final mixture. In contrast, the Pd_2_L_3_ species formed from **PolyMOC3** display significantly reduced susceptibility toward further activation, remaining at 22% Pd_2_L_3_ and yielding only 18% free **PolyL3** (Fig. 2b). This leads to a substantially lower quantitative activation, reflected by a reduced total number of newly formed discrete species per remaining intact cage of 1.63 for **PolyMOC3**, compared to 2.03 for **PolyMOC2** (Fig. 2c). Interestingly, the lower activation ratio observed for **PolyMOC3** compared to **PolyMOC2** is, at first glance, counterintuitive, as longer polymer chains are generally expected to enhance mechanophore activation^61^. We attribute this behaviour to more pronounced hydrodynamic interactions and chain entanglement effects in the longer polymers, which likely dissipate mechanical energy and reduce the efficiency of force transduction to the cage core. This interpretation was further supported by experiments in which the mass concentration of **PolyMOC3** was doubled to 5.00 mg mL^−1^, corresponding to a mechanophore concentration of 125 µM, identical to that used for **PolyMOC2** activation (2.50 mg mL^−1^). Under these conditions, sonochemical treatment yielded a final product distribution of 22% Pd_2_L_3_-type species and 78% intact **PolyMOC3** (Fig. 2b). Notably, **PolyMOC3** exhibited behaviour analogous to that previously observed for **PolyMOC2** when activated at a polymer mass concentration of 5.00 mg mL^−1^, with no further activation of the Pd_2_L_3_ intermediates detected. Consequently, this regime yielded the lowest overall quantitative activation, with only 0.37 discrete free species formed per residual intact cage (Fig. 2c). This comparison emphasises the critical importance of careful concentration control in supramolecular mechanophore systems, particularly when distinguishing between mass- and molar-based concentrations, as well as the significant influence of polymer chain length on activation behaviour.

We next investigated the effect of the saturation gas. **PolyMOC2** and **PolyMOC3** were exposed to ultrasound under argon at a polymer concentration of 2.50 mg mL^−1^. Time-dependent ^1^H NMR analysis revealed the appearance of a new set of signals after three hours (**PolyMOC3**) and four hours (**PolyMOC2**), corresponding to a previously unobserved species, which we assume to be a Pd_2_L_2_-type structure. Notably, using argon, the longer-chain **PolyMOC3** activated more rapidly than **PolyMOC2** (Supplementary Figs. S44, 56). When the polymer concentration was increased to 5.00 mg mL^−1^, ^1^H NMR of both PolyMOCs still showed residual proton resonances corresponding to the initial cage assemblies after six hours, albeit only in trace amounts. In both cases, signals of the proposed Pd_2_L_2_-type species were observed in the ^1^H NMR analysis of the resulting sonication mixture (Supplementary Figs. S47, 59). These results again confirm that higher polymer concentration decreased the activation rate. We also examined the influence of solvent by replacing CD_3_CN with D_2_O during sonication. The higher surface tension, lower vapour pressure, and enhanced radical formation when D_2_O is used create a markedly harsher sonochemical environment^75^. Indeed, when **PolyMOC3** was sonicated in D_2_O under both nitrogen and argon saturation, no defined species were detected after four hours of activation (Supplementary Figs. S62, 65). Instead, the spectra suggested complete degradation of all cage species. This indicates that sonochemical activation in aqueous media is irreversible. To examine the general reversibility after sonochemical activation, the systems were investigated in CD_3_CN again. Remarkably, **PolyMOC3** could be reassembled when activation was carried out under nitrogen at a polymer concentration of 5.00 mg mL^−1^. Freeze-drying the sonicated mixture after six hours and redissolving the solid in CD_3_CN (25 mM, identical to the initial self-assembly conditions) led to quantitative reformation of **PolyMOC3** (Supplementary Information, Chapter VIII). Our findings suggest that the degree of reversibility is governed by partial reduction of Pd^2+^ ions under sonication, aligning with known pathways in ultrasound-assisted Pd^0^ nanoparticle synthesis^76^. Encouraged by these findings, we further explored an alternative activation pathway, the mechanical “push” activation via ball milling in the solid state. **PolyMOC2** was subjected to ball milling at 20 Hz for 20 min. The ^1^H NMR analysis of the resulting solid reveals a ^1^H NMR spectrum identical to that of the free ligand **PolyL2**, indicating complete activation. Redissolving the solid in CD_3_CN (25 mM) and stirring for one hour led to the quantitative reassembly of **PolyMOC2**, demonstrating that solid state ball mill activation also achieves fully reversible mechanophore activation (Supplementary information, Chapter X)^77^.

### Controlled release of the anti-cancer drug cisplatin from PolyMOCs

**Fig. 3:**
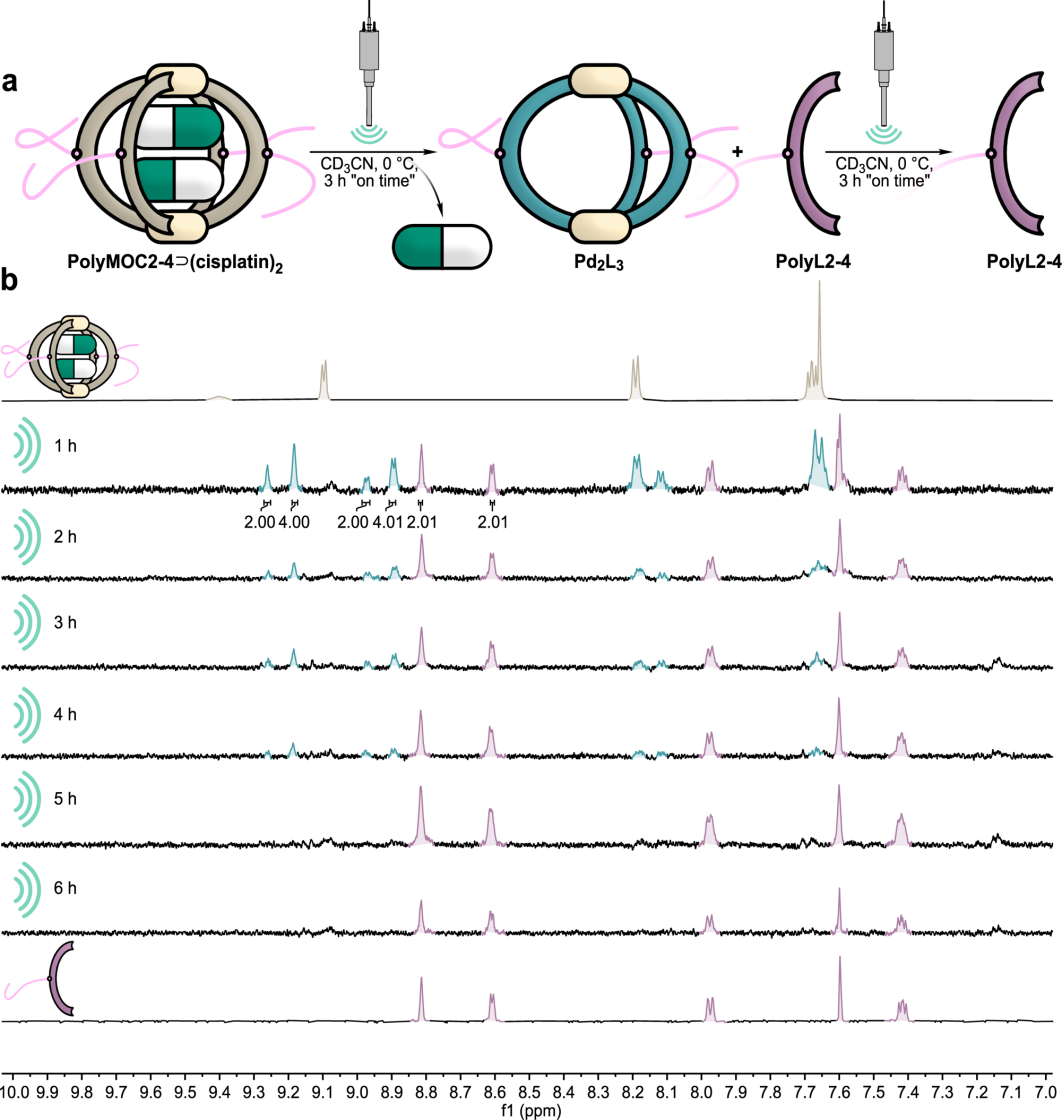
Ultrasound-induced anticancer drug release from metallosupramolecular mechanophores. **a** Schematic overview of the ultrasound-induced targeted release of cisplatin and disassembly of **PolyMOC2-4**. **b** Stacked ^1^H NMR spectra (600 MHz, CD_3_CN, 298 K) of **PolyMOC3⊃(cisplatin)**_**2**_ (top), after each hour of sonochemical activation (middle), and **PolyL3** (bottom).

To explore the biomedical potential for our ultrasound-activation strategy, we investigated the controlled release of the anticancer drug cisplatin. Therefore, we conducted a detailed investigation of both **PolyMOC2⊃(cisplatin)**_**2**_ and **PolyMOC3⊃(cisplatin)**_**2**_ under ultrasound activation with nitrogen and argon as saturation gas at the same concentration of 2.5 mg mL^−1^. Remarkably, both **PolyMOC⊃(cisplatin)**_**2**_ complexes exhibited similar or almost identical activation profiles under identical saturation gas conditions, with nearly identical kinetics and disassembly mechanisms observed under nitrogen as well as under argon saturation (Supplementary Information, Chapter IX). However, there was a noticeable difference in activation behaviour between the two gases, indicating that the nature of the saturation gas has a significant influence on the activation process, whereas polymer chain length does not. When nitrogen was used as the saturation gas, ultrasound activation of both host-guest complexes led to the full dissociation of one polymeric ligand within the first hour (Fig. 3a, b), revealing signals corresponding to a Pd_2_L_3_ species. The signals of the protons H_a_ and H_b_ split into two distinct sets in a 2:4 ratio, and additional signals corresponding to the free polymeric ligands appeared with an integral ratio of six for the Pd_2_L_3_ species and two for each proton H_a_ and H_b_ of the corresponding free ligands (Fig. 3b).

The gradual change in the ratio between the signals of the Pd_2_L_3_ species and those of the free ligands clearly revealed that the Pd_2_L_3_ species continued to undergo activation until complete dissociation was reached. After four hours of ultrasound activation for **PolyMOC3⊃(cisplatin)**_**2**_ and five hours of sonication for **PolyMOC2⊃(cisplatin)**_**2**_, only resonances corresponding to the free ligands remained in the ^1^H NMR spectra (Supplementary Figs. S87, 98). This clearly confirms the complete disassembly of the cages together with the full release of cisplatin. Switching the saturation gas from nitrogen to argon dramatically altered the activation pathway. Under otherwise identical experimental conditions, both host-guest complexes showed complete dissociation after only one hour, with the ^1^H NMR spectra displaying only the proton resonances corresponding to the free polymeric ligands **PolyL2** and **PolyL3** (Supplementary Figs. S92, 104). These findings further confirm that the sonication environment is significantly harsher in the presence of argon, which can be rationalised by the lower energy absorption capacity of the monoatomic gas compared to the diatomic nitrogen.

### Synthesis, characterisation and disassembly of Pd_12_L_24_-type nanospheres

**Fig. 4:**
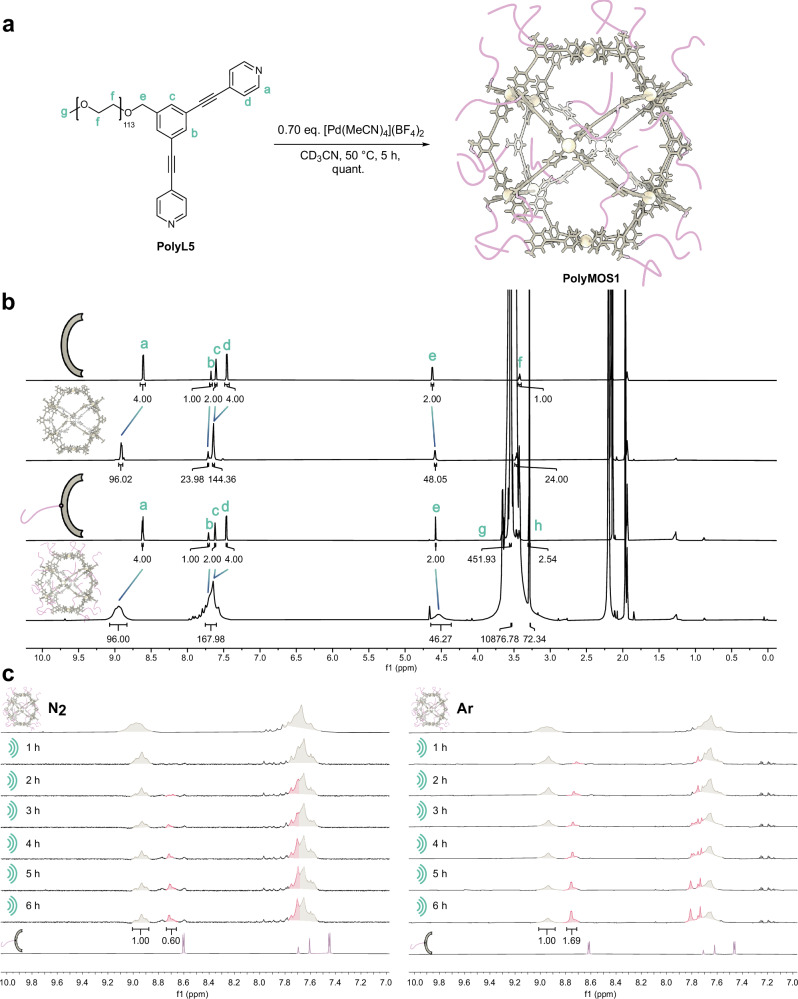
Synthesis, characterisation, and sonochemical activation of the polymer-decorated nanosphere. **a** Synthetic overview for the self-assembly of **PolyMOS1**. **b** Stacked ^1^H NMR spectra (600 MHz, CD_3_CN, 298 K) of **L4** (top), **MOS1** (middle top), **PolyL4** (middle bottom) and **PolyMOS1** (bottom). **c** Stacked ^1^H NMR spectra (600 MHz, CD_3_CN, 298 K) of **PolyMOS1** (top), after each hour of sonochemical activation (middle), and **PolyL4** (bottom) under nitrogen saturation (left) and argon saturation (right).

Our herein presented approach is not confined to the ubiquitous Pd_2_L_4_-type cage architectures, it can be seamlessly translated to virtually any Pd_n_L_2n_-type system. To push the limits, we synthesised one of the heaviest discrete Pd^2+^ assemblies known to date^4,30^. The self-assembly of 24 polymer-decorated ligands with twelve Pd^2+^ atoms yield a Pd_12_L_24_ nanosphere with a respective molecular weight of 133 kDa. Owing to its size and complexity, ^1^H NMR spectroscopy remains an exceptionally powerful tool for elucidating the structures of such large molecular assemblies. We therefore first targeted the synthesis of the novel non-polymeric isostructural Pd_12_L_24_ nanosphere **MOS1**. Self-assembly of the benzylic alcohol **L4** with tetrakis(acetonitrile)palladium(II) tetrafluoroborate in CD_3_CN at 50 °C yields **MOS1** in quantitative conversion within five hours (Supplementary Figs. S196). Further characterisation by ^13^C NMR and a suite of homo- and heteronuclear 2D techniques (COSY, NOESY, HSQC, and HMBC) confirmed the clean formation of a single reaction product (Supplementary Figs. S195–201). ^1^H DOSY NMR experiments revealed hydrodynamic radii of 5.1 Å for **L4** and 25.5 Å for **MOS1**, consistent with a molecular diameter of ca. 5 nm, which is in excellent agreement with reported Pd_12_L_24_ nanospheres (Supplementary Figs. S234–235)^5,30,78^. Finally, electrospray ionisation mass spectrometry corroborated the assembly, with well-resolved charge states from M^9+^ down to M^17+^ (Supplementary Fig. S202). Self-assembly of **PolyL5** with Pd^2+^ for five hours at 50 °C afforded **PolyMOS1**, a polymer-decorated Pd_12_L_24_ nanosphere obtained in almost quantitative yield with an impressive molecular weight of 132.6 kDa (Fig. 4a), along with the expected downfield shifts of the signals corresponding to the pyridine protons upon palladium coordination (Fig. 4b).

To probe the response of **PolyMOS1** towards mechanical activation, we prepared 7.5 mg mL^−1^ solutions in CD_3_CN, saturated them with either nitrogen or argon, and subjected the samples to ultrasound irradiation. In contrast to the disassembly of **PolyMOC1-3,** ^1^H NMR monitoring of **PolyMOS1** revealed no signals attributable to the free ligand **PolyL5** under either nitrogen or argon saturation after six hours of sonochemical activation (Fig. 4c). Instead, a gradual loss of the characteristic **PolyMOS1** resonances was observed over time, accompanied by the emergence of new sets of signals corresponding to disassembly products. Due to the limited analytical accessibility of such high-molecular-weight species, the exact nature of these products cannot be assigned with certainty. Nevertheless, the activation proceeded faster under argon than under nitrogen. After six hours of sonication with nitrogen as saturation gas, resonances of intact **PolyMOS1** were still present in the ^1^H NMR spectrum, accompanied by at least a second set of signals that we assign to disassembly fragments. Integration of the signal corresponding to H_a_ of **PolyMOS1** against this new set of signals revealed a ratio of 1:0.6 (Fig. 4c). Under argon saturation, the sonochemical activation was extended to a total of nine hours to probe whether free **PolyL5** signals would be recognisable in the ^1^H NMR spectrum. After six hours of activation, the ratio of the corresponding signal H_a_ to that of the newly formed fragments was 1:1.7 (Fig. 4c), increasing to 1:3.8 after nine hours (Supplementary Figs. S116–119). We therefore suggest that the large Pd_12_L_24_ nanospheres undergo a stepwise activation, gradually breaking into progressively smaller fragments during the activation process. The results of the sonication experiments document the qualitative disassembly of the initial Pd_12_L_24_ nanosphere. Additionally, the sonication experiments allow for comparison in activation efficiency when different saturation gases are used.

### Computational simulations of PolyMOC and PolyMOS disassembly

To further elucidate the observed experimental behaviour and access mechanistic details at the molecular level, computational studies were carried out, providing insights into both the qualitative disassembly mechanism and the magnitude of force needed to trigger this process. Simulating the disassembly of large supramolecular assemblies poses a serious challenge for quantum chemical methods due to the large system size, particularly when including solvent molecules explicitly. A tremendous speedup can be achieved when a MLIP is used. Benchmarking different potentials using a model Pd^2+^-complex showed that the energies obtained for restraint optimisations using FAIRChem’s pre-trained UMA-S^72^ are in good agreement with utilised density functional theory methods (Supplementary Fig. S120). However, using UMA-S for geometry optimisations leads to highly asymmetric, deformed structures, which is an indicator for improper description (Supplementary Fig. S121). Therefore, we fine-tuned UMA-S to describe the disassembly of the Pd_2_L_4_ PolyMOCs (Supplementary Information, Chapter XII). Using the resulting fine-tuned MLIP (labelled UMA-S-PdN), we simulated mechanochemically induced disassembly processes of the Pd_2_L_4_ MOCs solvated by acetonitrile. For this, we introduce ramped steered molecular dynamics (RS-MD), in which the external pulling force *F*(*t*) increases linearly as a function of the time *t*, i.e.,

1 $$F(t)=\frac{{dk}}{{dt}}\cdot t$$

**Fig. 5:**
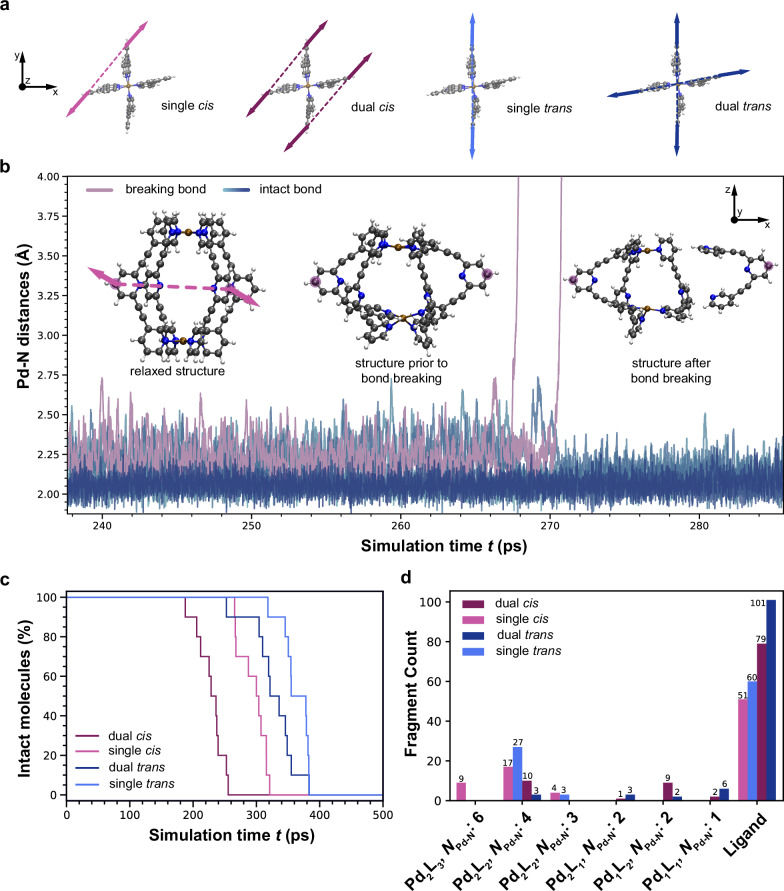
Pulling geometries, bond dissociation and fragmentation of the Pd_2_L_4_ MOC in RS-MD simulations. **a** Illustration of the different pulling geometries utilised for RS-MD simulations. The Pd_2_L_4_ MOC is shown in top view. **b** Distance-time profiles of all eight Pd–N coordination bonds (single pair cis-pulling,$\,\dot{k}$ = 9.6 pN ps^−1^) of one exemplary RS-MD trajectory. Blue curves: Pd–N bonds that remain intact. Purple curves: Pd–N bonds that undergo scission (distance > 3.0 Å). Included are representative snapshots showing the relaxed cage, the stretched configuration prior to bond breaking and the system after bond scission. Solvent is omitted in the visualisation for clarity. The Pd_2_L_4_ MOC is presented in side view. **c** Survival fraction of intact cages (%) versus time for the different pulling geometries ($\dot{k}\,$= 9.6 pN ps^−1^). **d** Fragments observed at the end of the Pd_2_L_4_ RS-MD simulations. Shown are the results of all trajectories and force ramps, i.e., 30 trajectories each for single *trans*, single *cis*, dual *cis* and dual *trans* pulling. *N*_Pd-N_ indicates the number of Pd–N bonds in the fragment.

Here, $\dot{k}=\frac{{dk}}{d{{{\rm{t}}}}}$ was chosen to be a constant value, and *k* is the force constant of the external pulling force. For the Pd_2_L_4_ MOC we defined two pulling geometries based on the relative positions of the ligands used as attachment points: *cis*, where forces are applied to two adjacent ligands, and *trans*, where forces are applied to two opposite ligands (Fig. 5a). Beyond this single pair pulling, we also applied external pulling forces to two independent pairs of attachment points simultaneously, engaging all four ligands of the Pd_2_L_4_ cage. Again, we distinguish between *trans* pulling (attachment points at opposite ligands) and *cis* pulling (attachment points at adjacent ligands) (Fig. 5a). For each of these four pulling modes, we performed ten MLIP-RS-MD simulations with $\dot{k}$ = 4.8 pN ps^−1^, 9.6 pN ps^−1^_,_ 19.2 pN ps^−1^, respectively, and five simulations without external force. Without force, no disassembly is observed within 500 ps of simulation time. The average times and corresponding pulling forces for bond rupture events across these different simulations are listed in Table 1.

**Table 1:** Average Pd–N bond dissociation times *t*_*D*_ and forces *F*_*D*_ obtained from MLIP-RSMD simulations of the Pd_2_L_4_ MOC using different pulling modes

$\dot{k}$ (pN · ps^−1^)	single *cis*		single *trans*		dual *cis*		dual *trans*	
	*t*_*D*_ (ps)	*F*_*D*_ (nN)	*t*_*D*_ (ps)	*F*_*D*_ (nN)	*t*_*D*_ (ps)	*F*_*D*_ (nN)	*t*_*D*_ (ps)	*F*_*D*_ (nN)
4.8	554	2.66	691	3.32	396	1.90	629	3.02
9.6	295	2.84	363	3.49	228	2.19	328	3.15
19.2	153	2.95	189	3.64	117	2.25	167	3.21

To assess the onset of disassembly, we initially focused on the single *cis*-pulling geometry. Trajectory analysis shows that either the two ligands being pulled on dissociate nearly simultaneously, yielding Pd_2_L_2_, or only one ligand being pulled on detaches, forming Pd_2_L_3_. As shown in Fig. 5b, the Pd–N coordination distances for a representative trajectory at $\dot{k}$ = 9.6 pN ps^−1^ reveal a near-simultaneous rupture of the two Pd–N bonds of one pulled ligand. The behaviour is illustrated by snapshots taken from the same trajectory. In contrast, RS-MD simulations with pulling forces applied in single *trans* position result exclusively in the formation of Pd_2_L_2_. This pulling mode also shows the highest forces necessary for dissociation, making it the least mechanoresponsive activation mode. For the dual *trans* pulling mode, forces necessary for the first bond scission event remain high, showing the same low force responsivity. In most trajectories using the dual *trans* pulling mode, all four ligands detach. Dual *cis* pulling requires the least amount of force to induce a reaction out of all four pulling modes. Comparing the RS-MD simulations of all four pulling geometries at $\dot{k}$ = 9.6 pN ps^−1^ shows that *cis* pulling generally leads to a faster activation of the Pd_2_L_4_ MOC (Fig. 5c). Across all simulations, the Pd_2_L_2_ fragment is the most frequently formed species besides the free ligand. Pd_2_L_3_ is formed exclusively under single *cis* pulling, whereas fragments containing only one ligand or only one Pd centre appear when two pulling forces are applied simultaneously, either in *cis* or *trans* (Fig. 5d). Furthermore, we analysed the kinetics of the Pd_2_L_4_ disassembly to gain insights into how strongly the free activation energy ∆*G*^‡^ is coupled to external pulling force (Supplementary Information, Chapter XII). Assuming a linear dependence of ∆*G*^‡^ on external force *F*, we were able to approximate the change of the activation energy with force, $\frac{{\mbox{d}}\triangle {\mbox{G}}^{{\ddagger}} \,}{{\mbox{d}}F}$, with −2.36, −2.48, −2.90 and −3.85 kcal mol^−1^ nN^−1^ for single *trans*, dual *trans*, single *cis*, and dual *cis* respectively. The force-coupling of ∆*G*^‡^ is modest compared to other mechanophores. For the Pd^2+^-complex studied by Küng et al.^10^, ∆*G*^‡^ changes by approximately 16.0 kcal mol^−1^ nN^−1^ (*cis* pulling) and 7.5 kcal mol^−1^ nN^−1^ (*trans* pulling), what we trace back to the unfavourable angle between the ligands being pulled on and the scissile Pd–N bond, caused by the non-linear structure of the ligands. Nonetheless, *cis* pulling more efficiently activates Pd–N scission than *trans* pulling, in accordance with previous studies^10^.

**Fig. 6:**
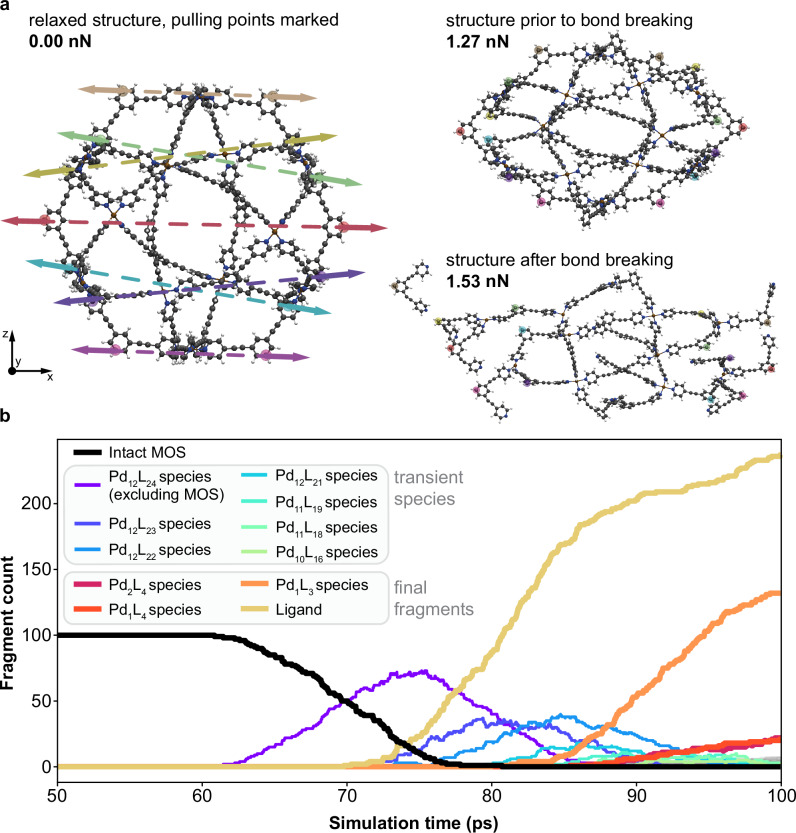
Snapshots and time-resolved fragmentation of Pd12L24 in RS-MD simulations using multiple pulling pairs. **a** Snapshots from RS-MD simulations of Pd_12_L_24_ in acetonitrile with multiple pulling pairs ($\dot{k}=17.5$ pN ps^−1^; solvent is omitted for clarity). Three representative structures picked from one trajectory: a relaxed structure (left), a structure prior to bond breaking (top right) and a structure after bond scission (bottom right). The coloured arrows indicate the multiple pulling pairs on which force is applied. **b** Fragmentation of Pd_12_L_24_ MOS over time during RS-MD simulations using a force ramp of 17.5 pN ps^−1^. Species of varying connectivity are grouped by Pd_X_L_Y_ compositions (Supplementary Figs. S133–134, Supplementary Table S17). Fragments that occur less than ten times in the 100 simulations are marked in grey.

A similar approach was used to study the disassembly of the supramolecular Pd_12_L_24_ sphere. 120 RS-MD simulations of a Pd_12_L_24_ MOS solvated in 530 acetonitrile molecules using the UMA-S-PdN potential were conducted. Force was applied to seven pulling pairs in total (Fig. 6a, Supplementary Fig. S128b). Simulations were performed using $\dot{k}$ = 5.8 pN ps^−1^ (10 simulations), 11.7 pN ps^−1^ (10 simulations) and 17.5 pN ps^−1^ (100 simulations). The simulation times depend on the applied force ramp and are listed in Table S8 of the Supplementary Information. For each force ramp, multiple ligands dissociate, and the nanosphere is deconstructed over time (Fig. 6). The first bond breaks occur at average forces of 1.15, 1.17 and 1.22 nN for the selected force ramps. Pulling on multiple pairs, not only do single ligands detach, but the Pd_12_L_24_ nanosphere disassembles into smaller fragments. Assuming linear dependence of free activation energy ∆G^‡^ on the external pulling force *F*, we obtain $\frac{{\mbox{d}}\triangle {\mbox{G}}^{{\ddagger}} \,}{{\mbox{d}}F}=-\!8.59\,{{\mathrm{kcal}}}$ mol^−1^ nN^−1^ for the RS-MD simulations on Pd_12_L_24_, with multiple (i.e., seven) pulling pairs. Analysing the fragmentation for the 100 simulations employing a force ramp of $\dot{k\ }=17.5$ pN ps^−1^ reveals that although a large diversity of fragments is formed during the simulations, in particular Pd_11_ and Pd_10_ species, the most frequently found fragments at the end of the simulations are the free ligand, Pd_2_L_4_, Pd_1_L_4_, and Pd_1_L_3_ (Fig. 6b). This suggests that the dissociation mechanism proceeds stepwise via pulling out of Pd_1_ and Pd_2_ fragments from the MOS. This corroborates the stepwise fragmentation of the Pd_12_L_24_ polyMOS postulated from analysis of the experimental results.

In summary, we have developed a versatile synthetic strategy for polymer-decorated ligands that enables the preparation of metallosupramolecular cage mechanophores across the Pd_n_L_2n_ family as well as their mechanochemical disassembly. Through systematic variation of polymer chain length, concentration, saturation gas, and solvent, using a series of Pd_2_L_4_ cages (**PolyMOC1-4**), we uncovered key condition principles governing sonochemical activation. We confirmed that efficient force transduction requires a minimum polymer length, increases with chain extension, but is eventually suppressed by entanglement effects. Importantly, we demonstrated that reassembly of activated PolyMOCs is possible under specific conditions. Extensive molecular dynamics simulations based on a novel, fine-tuned MLIP were performed using ramped steering. These simulations allowed elucidation of the force-induced disassembly pathways using multiple modes of pulling and force ramps. Beyond these fundamental insights, we further showcased the functional utility of the sonochemical activation by encapsulating the anticancer drug cisplatin within polymer-decorated cages, studying their stability in aqueous media, and achieving targeted drug release upon ultrasound irradiation. Additionally, we demonstrated that solid-state ball milling provides a powerful alternative to achieve quantitative disassembly in a fully reversible manner. Finally, we highlighted the generality of our approach by synthesising one of the heaviest discrete macromolecules to date, a polymer-decorated palladium coordination nanosphere, **PolyMOS1**, and probing its sonochemical responsiveness. Also, for this system, RS-MD simulations could provide an understanding of the dissociation pathways of Pd_n_L_2n_ MOCs using multiple pulling atoms and force ramps. Together, these findings establish a broadly applicable platform for the design of polymer-embedded mechanophores and open new directions for controllable, stimuli-responsive supramolecular architectures.

## Methods

Detailed procedures and methods are given in the supplementary information.

### General synthetic strategy for polymer-decorated architectures

The synthesis of polymer-decorated Pd_n_L_2n_ architectures begins with the synthesis of the corresponding polymeric ligands. For this purpose, we start from commercially available 3,5-dibromobenzylalcohol or (2,6-dibromopyridin-4-yl)methanol. Two consecutive Sonogashira couplings, the first of which is trimethylsilyl acetylene coupling, followed by in situ deprotection and coupling of 3- or 4-iodopyridine, produce ligands with differing bend angles for the synthesis of Pd_2_L_4_- (**L1-3**) or Pd_12_L_24_-type (**L4**) structures. An alternative synthetic pathway involves a Sonogashira coupling of trimethylsilyl acetylene with 3- or 4-bromopyridine, followed by deprotection of the alkyne. A subsequent Sonogashira cross-coupling with the corresponding benzene or pyridine core then furnishes the target ligands (**L1-3**). To introduce polymer chains, we used Appel reactions to convert the benzylic alcohols into benzyl bromides, which were subsequently reacted in a Williamson ether synthesis with poly(ethylene glycol) monomethyl ether to afford the polymer-functionalised ligands (**PolyL1-5**). Self-assembly of the polymer-decorated architectures was achieved by combining the polymeric ligands with tetrakis(acetonitrile)palladium(II) tetrafluoroborate in acetonitrile (see the experimental details).

### Sonication experiments

All sonication experiments were carried out with a Vibra cell VCX 750 sonicator (*P* = 750 W) with a frequency of 20 kHz, a full wave probe (13 mm) and a pulse sequence of 1 s on, 1 s off. Before sonication, solutions of the ligands (1 mg mL^−1^) and of the assembled architectures (non-polymeric: 1 mg mL^−1^; polymeric: 2.5–7.5 mg mL^−1^) were prepared in dry CH_3_CN, CD_3_CN or D_2_O, cooled to 0 °C in an ice-water bath, and saturated with argon or nitrogen by bubbling the gases through the solutions for ten minutes. During sonication, the temperature was maintained at 0 °C, and the saturation gas was continuously bubbled through the solution throughout the entire process. The progress of sonication was monitored using two approaches. In the first method, sonication was carried out in non-deuterated solvents, after which the solvent was removed by freeze-drying and the resulting residue was analysed by ^1^H NMR spectroscopy. In the second method, sonication was performed directly in deuterated solvents, and ^1^H NMR spectra were recorded every hour over a period of six hours.

### Ball milling experiments

All ball milling experiments were carried out using a Retsch MM 400 ball mill operating at a frequency of 20 Hz. 20 mg of **PolyMOC2** and three zirconium oxide balls were placed in a 2 mL Eppendorf tube, and the sample was milled for 20 min. Afterwards, 1.25 mg of the resulting solid was dissolved in 0.5 mL CD_3_CN (c = 2.5 mg mL^−1^) and analysed by ^1^H NMR to monitor the reaction progress. The remaining solid was redissolved in CD_3_CN to reach a concentration of 20.0 mM. The resulting solution was stirred for one hour at room temperature, and the successful reassembly was confirmed by ^1^H NMR analysis.

### Computations

The Python-based software Minirauche that integrates TeraChem^79,80^ and the FAIRChem package^81^ was used to perform (RS)-MD, respectively. For fine-tuning the UMA-S MLIP^72^, a dataset of 1,600,332 structures of six different molecular fragments was created. To construct this dataset, calculations were performed on the level of density functional theory (DFT) employing the B3LYP functional^82^ using D3-dispersion correction^83^ and the 6–31 G* basis set^84–86^ with LanL2DZ effective core potential^87^ for Pd^2+^. The RS-MD simulations were conducted using the fine-tuned UMA-S-PdN model which was fine-tuned on this dataset. The simulations were run in an NVT ensemble using the Bussi-Parrinello version of Langevin dynamics^88^ with a timestep of 1 fs, a thermostat temperature of 300 K, and a thermostat damping parameter of 7 ps^−1^.

### Software used in the study

Data collection/data analysis: ChemDraw 22.0.0, MestReNova V 14.2.0-26256, TeraChem V 1.9, FAIRChem package, Minirauche.

## Supplementary information

Supplementary Information

Transparent Peer Review file

## Data Availability

All data are available from the corresponding authors upon request. All data supporting the findings of this study are documented within manuscript and supplementary information and the Source Data are openly available on Zenodo at https://zenodo.org/records/20394633. Due to the large total file size, the complete RS-MD trajectories are not deposited in the repository. Access to the complete RS-MD trajectories can be obtained by contacting the corresponding authors. Requests will be answered within four weeks and access will be provided for an agreed period sufficient to retrieve the data.

## Code availability

The software Minirauche used for the simulations in this study is publicly available on Zenodo at 10.5281/zenodo.20394633.
