# Supplementary material for: Mechanochemical disassembly pathways of self-assembled polymer-decorated PdnL2n supramolecular architectures
Source: Nat Commun. 2026 Jun 19;17:5459. doi: 10.1038/s41467-026-74561-4 (PMC13282382; doi:10.1038/s41467-026-74561-4)
Supplement: Supplementary file 1 — Supplementary Information [file 41467_2026_74561_MOESM1_ESM.pdf]

Supplementary Information

Mechanochemical disassembly pathways of self-assembled polymer-decorated Pd_nL_{2n} supramolecular architectures

Tim David[1], Regina Lennarz[2], Jan A. Meissner[2], Anne Germann[2], Jan Meisner[2*] and Bernd M. Schmidt[1*]

[1]Institute for Organic Chemistry and Macromolecular Chemistry, Heinrich Heine University Düsseldorf, Universitätsstraße 1, 40225 Düsseldorf, Germany

[2]Institute for Physical Chemistry, Heinrich Heine University Düsseldorf, Universitätsstraße 1, 40225 Düsseldorf, Germany

Contents

I. ***General*** **........ 6**

NMR measurements 6

IR spectroscopy 6

Mass spectrometry 6

Sonication experiments 6

Ball milling 7

II. ***Experimental details*** **........ 8**

Synthesis of MOC1 8

Synthesis of 3-((trimethylsilyl)ethynyl)pyridine (**1**) 8

Synthesis of 3-ethynylpyridine (**2**) 9

Synthesis of 3,5-bis(pyridin-3-ylethynyl)phenol (**L1**) 9

Synthesis of **MOC1** 10

Synthesis of MOC2 11

Synthesis of (3,5-bis(pyridin-3-ylethynyl)phenyl)methanol (**L2**) 11

Synthesis of **MOC2** 12

Synthesis of MOC3 12

Synthesis of (2,6-dibromopyridin-4-yl)methanol (**3**) 13

Synthesis of (2,6-bis(pyridin-3-ylethynyl)pyridin-4-yl)methanol (**L3**) 13

Synthesis of **MOC3** 14

Synthesis of PolyMOC1 15

Synthesis of 3,3'-((4-(bromomethyl)pyridine-2,6-diyl)bis(ethyne-2,1-diyl))dipyridine (**4**) 15

Synthesis of **PolyL1** 16

Synthesis of **PolyMOC1** 17

Synthesis of PolyMOC2 17

Synthesis of **PolyL2** 18

Synthesis of **PolyMOC2** 19

Synthesis of PolyMOC3 19

Synthesis of **PolyL3** 20

Synthesis of **PolyMOC3** 21

Synthesis of PolyMOC4 21

Synthesis of **PolyL4** 22

Synthesis of **PolyMOC4** 23

Synthesis of MOS1 ($Pd_{12}L4_{24}$) 23

Synthesis of (3,5-bis((trimethylsilyl)ethynyl)phenyl)methanol (**5**) 24
Synthesis of (3,5-bis(pyridin-4-ylethynyl)phenyl)methanol (**L4**) 24
Synthesis of **MOS1** ($Pd_{12}L4_{24}$) 25

Synthesis of PolyMOS1 ($Pd_{12}L4_{24}$) 26

Synthesis of 4,4'-((5-(bromomethyl)-1,3-phenylene)bis(ethyne-2,1-diyl))dipyridine (**6**) 26
Synthesis of **PolyL5** 27
Synthesis of **PolyMOS1** ($Pd_{12}L4_{24}$) 28

Cisplatin encapsulations 29

MOC1 cisplatin encapsulation 29
MOC2 cisplatin encapsulation 29
MOC3 cisplatin encapsulation 30
PolyMOC2 cisplatin encapsulation 31
PolyMOC3 cisplatin encapsulation 31
PolyMOC4 cisplatin encapsulation 32

III. Stability of cisplatin inclusion complexes in aqueous media 33

MOC1⊃(cisplatin)$_2$ 33
MOC2⊃(cisplatin)$_2$ 34
MOC3⊃(cisplatin)$_2$ 34
PolyMOC2⊃(cisplatin)$_2$ 35
PolyMOC3⊃(cisplatin)$_2$ 39

IV. Investigating the role of polymer chains and cage stability under sonication conditions 45

PolyMOC and PolyMOC⊃(cisplatin)$_2$ under high dilution conditions 45
Sonication of MOC3 and MOC3⊃(cisplatin)$_2$ 48
Sonication of polymeric ligand PolyL3 50

V. Acoustic properties of the applied ultrasound field 52

VI. Ultrasound-triggered disassembly of PolyMOC 54

PolyMOC1 54

Sonication in acetonitrile 54

Nitrogen as saturation gas (c = 0.77 mg mL^{-1}, 125 µM) 54
Nitrogen as saturation gas (c = 2.50 mg mL^{-1}, 412 µM) 56

PolyMOC2 58

Sonication in acetonitrile 58

Nitrogen as saturation gas (c = 2.50 mg mL^{-1}, 125 µM) 58
Nitrogen as saturation gas (c = 5.00 mg mL^{-1}, 250 µM) 61
Argon as saturation gas (c = 2.50 mg mL^{-1}, 125 µM) 62

Argon as saturation gas (c = 5.00 mg mL^{-1}, 250 μM) ... 64

PolyMOC3 ... **67**

Sonication in acetonitrile ... **67**

Nitrogen as saturation gas (c = 2.50 mg mL^{-1}, 62.5 μM) ... 67

Nitrogen as saturation gas (c = 5.00 mg mL^{-1}, 125 μM) ... 70

Argon as saturation gas (c = 2.50 mg mL^{-1}, 62.5 μM) ... 71

Argon as saturation gas (c = 5.00 mg mL^{-1}, 125 μM) ... 73

Sonication in water ... **75**

Nitrogen as saturation gas ... 75

Argon as saturation gas ... 77

VII. Influence of chain length and concentration on PolyMOC activation rates ... ***80***

VIII. Reversibility studies of disassembled PolyMOCs ... ***84***

Sonication with nitrogen as saturation gas and 2.50 mg mL^{-1} concentration ... 84

Sonication with nitrogen as saturation gas and 5.00 mg mL^{-1} concentration ... 85

Sonication with argon as saturation gas and 2.50 mg mL^{-1} concentration ... 87

Sonication with argon as saturation gas and 5.00 mg mL^{-1} concentration ... 88

IX. Ultrasound-triggered cisplatin release from PolyMOC ... ***89***

Cisplatin release from PolyMOC2⊃(cisplatin)$_2$ ... **89**

Nitrogen as saturation gas ... 89

Argon as saturation gas ... 92

Cisplatin release from PolyMOC3⊃(cisplatin)$_2$ ... **95**

Nitrogen as saturation gas ... 95

Argon as saturation gas ... 98

Cisplatin release from PolyMOC4⊃(cisplatin)$_2$ ... **102**

Nitrogen as saturation gas ... 102

Argon as saturation gas ... 104

X. Ball milling induced disassembly and reassembly of PolyMOC2 ... ***107***

XI. Ultrasound-triggered disassembly of nanospheres ... ***110***

Nitrogen as saturation gas ... 110

Argon as saturation gas ... 112

XII. Molecular dynamics simulations and computational analysis of Pd_nL_{2n} disassembly ***114***

Benchmarking of potentials using a Pd(II)Py_4 model compound ... 114

Generation of ML training data ... 116

Fine tuning UMA-S ... 118

Ramped steered molecular dynamics simulations (RS-MD) ... 120

RS-MD simulations – pulling modes 122
RS-MD simulations – setup 123
RS-MD simulations – results 126
Kinetic analysis 132
Fragment analysis 135
XIII. Spectra* *139
Synthesis 139
Cisplatin encapsulations 184
DOSY experiments 190
XIV. References* *192

I. General

Chemicals and solvents were purchased from Sigma-Aldrich, VWR/Merck, Tokyo Chemical Industry, BLDpharm, and Fisher Scientific and were used without further purification. Dry solvents were obtained from an MBraun solvent purification system. Reactions were monitored by thin layer chromatography (TLC) using silica gel plates from Macherey Nagel (ALUGRAM® Xtra SIL G/UV254). Column chromatography was done with silica gel from Macherey Nagel (Silica 60 M, 0.04-0.063 mm). The eluents are stated individually for each reaction. The solvents were removed under reduced pressure by using a rotary evaporator at 50 °C, if not stated otherwise.

NMR measurements

All NMR spectra were measured on a Bruker Avance III – 300, a Bruker Avance III – 600, and a Bruker Avance NEO Evo – 600 spectrometer at 25 °C using deuterated solvents and their residual protonated solvent signals as internal standards for ^{1}H and ^{13}C spectra (^{1}H: δ($CDCl_3$) = 7.26 ppm; ^{1}H: δ(D_2O) = 4.79 ppm; ^{1}H: δ(CD_3CN) = 1.94 ppm; ^{13}C δ($CDCl_3$) = 77.16 ppm; ^{13}C δ(CD_3CN) = 118.26, 1.32 ppm). The splitting patterns are abbreviated as follows: singlet (s), doublet (d), triplet (t), quartet (q), quintet (p), heptet (hept), multiplet (m), and broad (br). All ^{1}H NMR spectra of polymeric compounds were baseline corrected using a Whittaker Smoother: filter=45, smooth factor=16000, if not stated otherwise.

IR spectroscopy

Infrared spectra were measured with a Shimadzu IR Affinity-1 with ATR technique.

Mass spectrometry

MALDI mass spectrometry was performed on a MALDI-TOF/TOF UltrafleXtreme (Bruker Daltonics). ESI mass spectrometry was performed on a UHR-QTOF maXis 4G (Bruker Daltonics).

Sonication experiments

All sonication experiments were performed with a Vibra cell VCX 750 sonicator (750 W) with a frequency of 20 kHz, an amplitude of 30 %, a full wave probe (13 mm), and a pulse sequence of 1 s on and 1 s off. The distance between the titanium tip and the bottom of the Suslick vessek was approximately 1 cm. The Suslick vessels were made by the Glasbläserei at Heinrich-Heine-University Düsseldorf.

Ball milling

All ball milling experiments were performed with a Retsch MM 400 with a frequency of 20 Hz, zirconium oxide balls (3 balls, 20 mg sample), for 20 minutes in 2 mL Eppendorf tubes.

II. Experimental details

Synthesis of MOC1

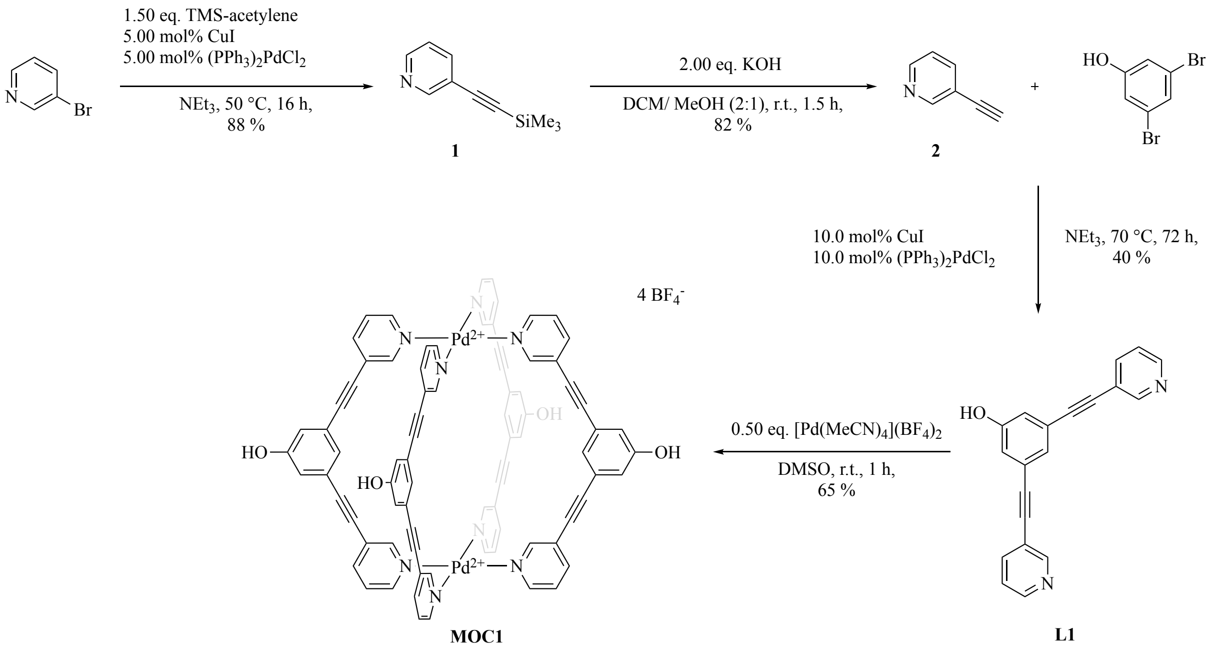

Figure S1: Synthetic overview of cage **MOC1**.

Synthesis of 3-((trimethylsilyl)ethynyl)pyridine (1)

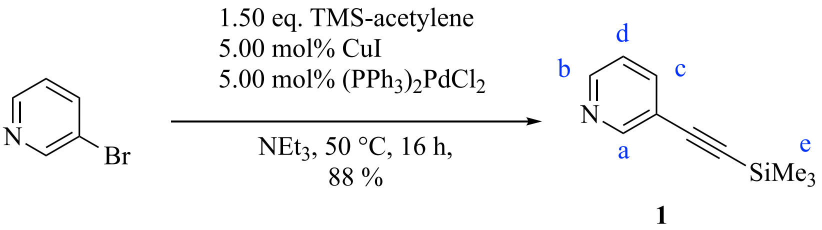

3-Bromopyridine (2.93 mL, 30.0 mmol, 1.00 eq.) was dissolved in 63.0 mL triethylamine and degassed with nitrogen for 10 minutes. Trimethylsilylacetylene (6.23 mL, 45.0 mmol, 1.50 eq.), dichlorobis(triphenylphosphine)palladium(II) (1.05 g, 1.50 mmol, 5.00 mol%), and copper(I) iodide (0.29 g, 1.50 mmol, 5.00 mol%) were added, and the yellow suspension was stirred for 16 hours at 50 °C. The black mixture was filtered through Celite with DCM (200 mL), washed with water (3 x 100 mL), dried over magnesium sulfate, and the solvent was removed under reduced pressure. The crude product was purified *via* column chromatography (cyclohexane/ ethyl acetate 99:1) to yield **1** (4.65 g, 26.5 mmol, 88 %) as a bright yellow oil.

^{1}**H NMR** (300 MHz, $CDCl_3$): δ 8.69 (s, 1H, **a**), 8.52 (s, 1H, **b**), 7.74 (ddd, J = 7.9, 2.2, 1.7 Hz, 1H, **c**), 7.26 – 7.19 (m, 1H, **d**), 0.26 (s, 8H, **e**).

All the analytical data obtained was in accordance with the literature[1].

Synthesis of 3-ethynylpyridine (2)

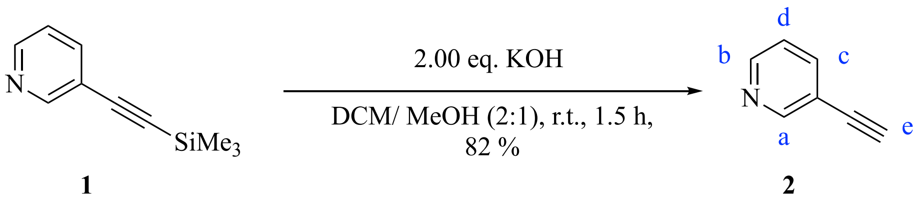

1 (1.76 g, 10.1 mmol, 1.00 eq.) was dissolved in a 2:1 dichloromethane/ methanol mixture (44.0 mL). Anhydrous potassium hydroxide (1.13 g, 20.1 mmol, 2.00 eq.) was added, and the mixture was stirred at room temperature for 2 hours. The reaction was diluted with 30.0 mL water, the phases were separated, and the aqueous phase was further extracted twice with 50.0 mL dichloromethane. The combined organic phases were dried over magnesium sulfate, and the solvent was removed under reduced pressure to yield **2** (0.85 g, 8.26 mmol, 82 %) as a brown oil that slowly crystallizes over time.

^{1}H NMR (300 MHz, $CDCl_3$): δ 8.76 – 8.69 (m, 1H, **a**), 8.57 (dd, J = 4.9, 1.7 Hz, 1H, **b**), 7.77 (dt, J = 7.8, 1.9 Hz, 1H, **c**), 7.31 – 7.21 (m, 1H, **d**), 3.22 (s, 1H, **e**).
All the analytical data obtained was in accordance with the literature[2].

Synthesis of 3,5-bis(pyridin-3-ylethynyl)phenol (L1)

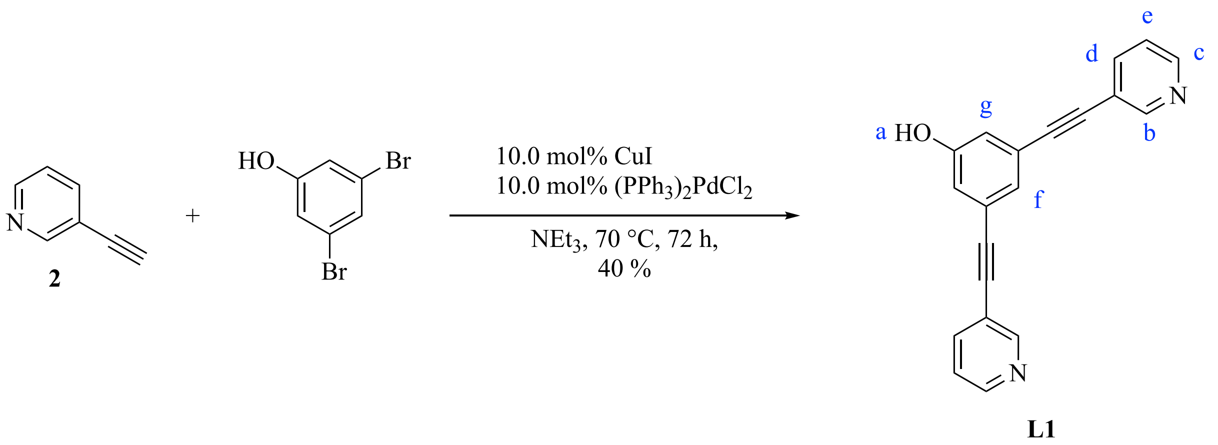

3,5-dibromophenol (504 mg, 2.00 mmol, 1.00 eq.), **2** (825 mg, 8.00 mmol, 4.00 eq.), dichlorobis(triphenylphosphine)palladium(II) (140 mg, 0.20 mmol, 10.0 mol%) and copper(I) iodide (38.0 mg, 0.20 mmol, 10.0 mol%) were dissolved in 20.0 mL of degassed triethylamine and stirred at 70 °C for 72 hours under an argon atmosphere. The reaction mixture was diluted with 50.0 mL of ethyl acetate and filtered through celite. The solvent was removed under reduced pressure. The crude product was purified by column chromatography (dichloromethane/ methanol 97:3) to yield **L1** (236 mg, 0.79 mmol, 40 %) as a colorless solid.

^{1}H NMR (300 MHz, DMSO-d6): δ 10.17 (s, 1H, **a**), 8.81 – 8.74 (m, 2H, **b**), 8.61 (dd, J = 4.9, 1.7 Hz, 2H, **c**), 8.00 (ddd, J = 7.9, 2.2, 1.7 Hz, 2H, **d**), 7.48 (ddd, J = 7.9, 4.9, 0.9 Hz, 2H, **e**), 7.25 (t, J = 1.4 Hz, 1H, **f**), 7.02 (d, J = 1.4 Hz, 2H, **g**); **^{1}H NMR** (300 MHz, CD_3CN): δ 8.74 (dd, J = 2.3, 0.9 Hz, 2H, **a**), 8.57 (dd, J = 4.9, 1.7 Hz, 2H, **b**), 7.89 (ddd, J = 7.9, 2.2, 1.7 Hz, 2H, **c**), 7.49 (s, 1H, **d**), 7.38 (ddd, J = 7.9, 4.9, 0.9 Hz, 2H, **e**), 7.27 (t, J = 1.4 Hz, 1H, **f**), 7.05 (d, J = 1.4 Hz, 2H, **g**).

All the analytical data obtained was in accordance with the literature[3].

Synthesis of MOC1

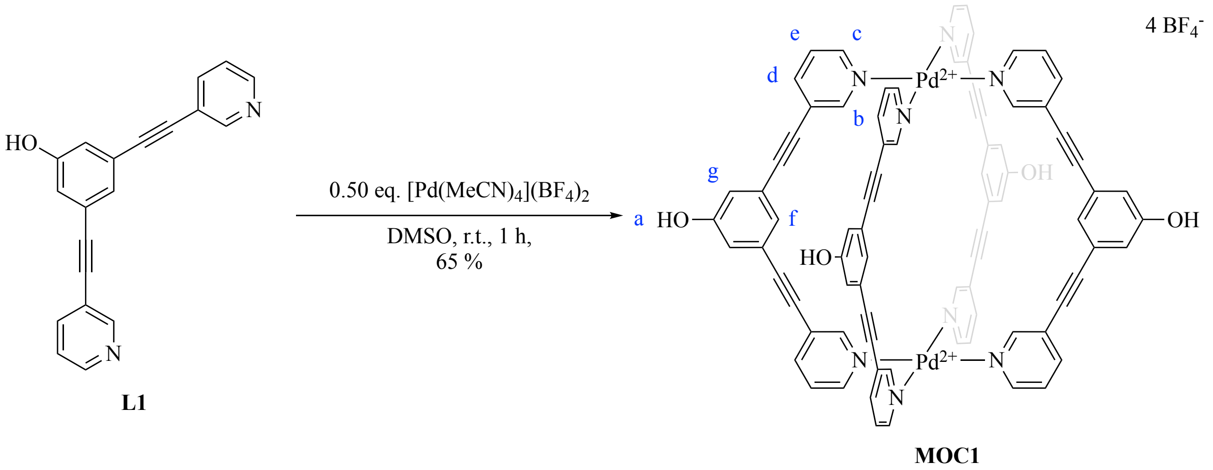

Tetrakis(acetonitrile)palladium(II) tetrafluoroborate (22.2 mg, 49.9 µmol, 1.00 eq.) and **L1** (29.5 mg, 99.7 µmol, 2.00 eq.) were dissolved in 2.00 mL dimethyl sulfoxide and stirred for one hour at room temperature. The addition of acetone and diethyl ether led to the precipitation of the crude product. The off-white solid was washed with acetone and dried in air to yield **MOC1** (28.3 mg, 16.2 µmol, 65 %).

^{1}H NMR (300 MHz, DMSO-d6): δ 10.31 (s, 4H, **a**), 9.58 (s, 8H, **b**), 9.34 (d, J = 5.5 Hz, 8H, **c**), 8.27 (dt, J = 8.1, 1.6 Hz, 8H, **d**), 7.80 (dd, J = 8.1, 5.7 Hz, 8H, **e**), 7.39 (t, J = 1.4 Hz, 4H, **f**), 7.08 (d, J = 1.4 Hz, 8H, **g**); **^{1}H NMR** (300 MHz, CD_3CN): δ 9.36 (s, 8H, **a**), 9.14 – 8.91 (m, 8H, **b**), 8.08 (dt, J = 8.1, 1.5 Hz, 8H, **c**), 7.81 – 7.51 (m, 12H, **d**), 7.43 (t, J = 1.4 Hz, 4H, **e**), 7.11 (d, J = 1.4 Hz, 8H, **f**).

All the analytical data obtained was in accordance with the literature[3].

Synthesis of MOC2

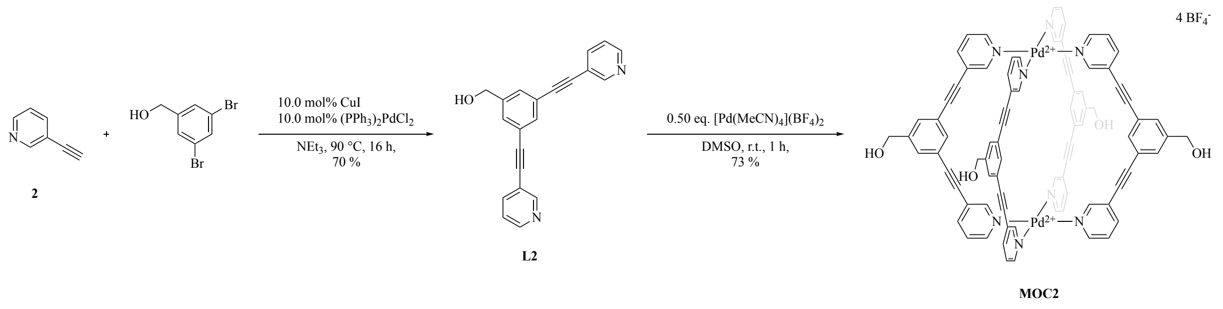

Figure S2: Synthetic overview of cage **MOC2**.

Synthesis of (3,5-bis(pyridin-3-ylethynyl)phenyl)methanol (L2)

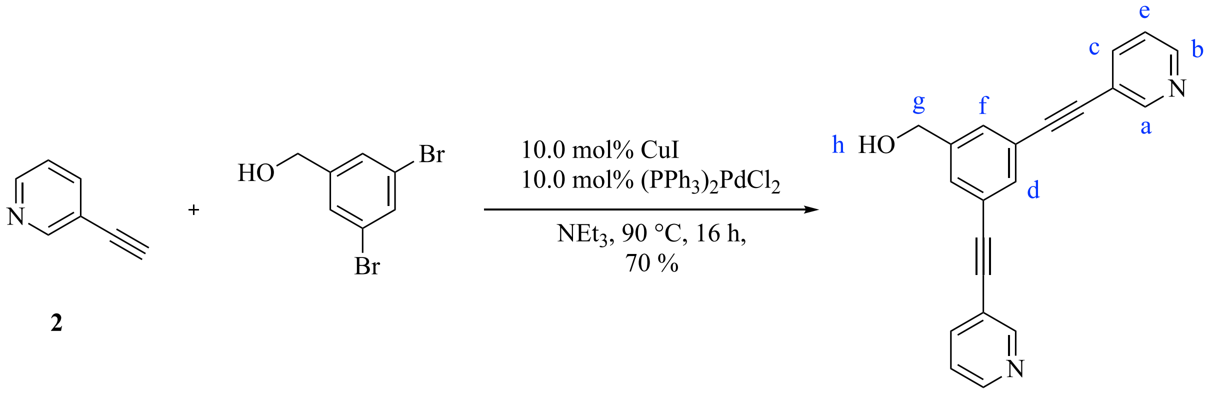

In a nitrogen atmosphere, **2** (0.54 g, 5.24 mmol, 3.00 eq.), 3,5-dibromobenzylalcohol (0.46 g, 1.75 mmol, 1.00 eq.), dichlorobis(triphenylphosphine)palladium(II) (122 mg, 0.17 mmol, 10.0 mol%) and copper(I) iodide (33.0 mg, 0.17 mmol, 10.0 mol%) were dissolved in triethylamine and degassed with nitrogen for 10 minutes. The suspension was stirred at 90 °C for 16 hours. The reaction mixture was filtered through Celite using ethyl acetate and dichloromethane before solvent removal. The crude product was purified *via* column chromatography (ethyl acetate/ cyclohexane 95:5) to yield **L2** (0.38 g, 1.22 mmol, 70 %) as an off-white solid.

^{1}H NMR (300 MHz, $CDCl_3$): δ 8.76 (dd, J = 2.2, 0.9 Hz, 2H, **a**), 8.56 (dd, J = 4.9, 1.7 Hz, 2H, **b**), 7.81 (ddd, J = 7.9, 2.2, 1.7 Hz, 2H, **c**), 7.66 (t, J = 1.6 Hz, 1H, **d**), 7.56 (dt, J = 1.5, 0.7 Hz, 2H, **e**), 7.30 (ddd, J = 7.9, 4.9, 0.9 Hz, 2H, **f**), 4.78 – 4.70 (m, 2H, **g**), 2.26 (t, J = 5.9 Hz, 1H, **h**). All the analytical data obtained was in accordance with the literature[4].

Synthesis of MOC2

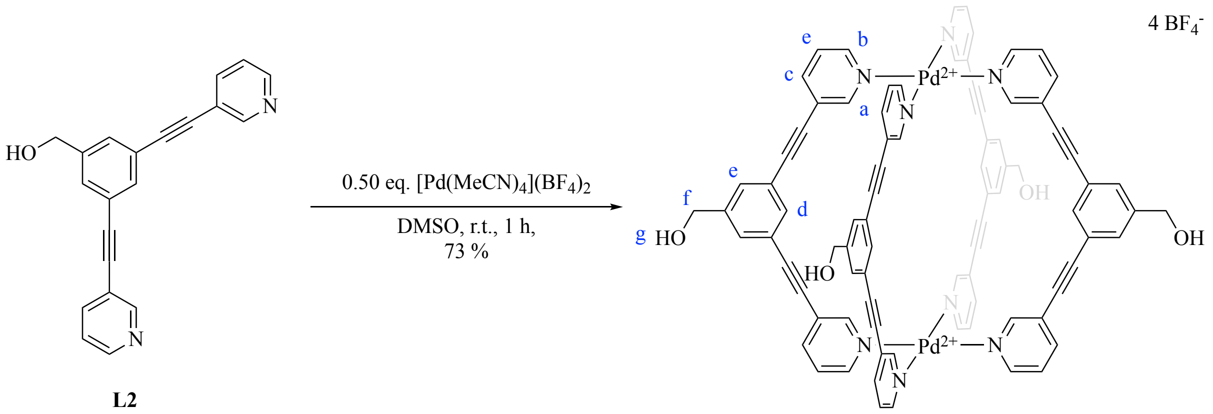

L2

MOC2

Tetrakis(acetonitrile)palladium(II) tetrafluoroborate (13.4 mg, 30.0 µmol, 1.00 eq.) and **L2** (18.7 mg, 60.0 µmol, 2.00 eq.) were dissolved in 1.20 mL dimethyl sulfoxide and stirred for one hour at room temperature. The addition of ethyl acetate led to the precipitation of the crude product. The off-white solid was washed with ethyl acetate and diethyl ether and dried in air to yield **MOC2** (16.5 mg, 9.16 µmol, 73 %).

^{1}H NMR (300 MHz, CD_3CN): δ 9.42 (d, J = 1.8 Hz, 8H, **a**), 9.09 (ddd, J = 5.8, 1.4, 0.5 Hz, 8H, **b**), 8.14 – 8.05 (m, 8H, **c**), 7.81 (t, J = 1.6 Hz, 4H, **d**), 7.68 – 7.57 (m, 16H, **e**), 4.60 (dd, J = 5.6, 0.9 Hz, 8H, **f**), 3.42 (t, J = 5.7 Hz, 4H, **g**); **^{1}H NMR** (600 MHz, DMF-d7): δ 9.96 (d, J = 1.9 Hz, 8H, **a**), 9.65 (dd, J = 6.0, 1.4 Hz, 8H, **b**), 8.60 – 8.31 (m, 8H, **c**), 7.93 (dd, J = 8.0, 5.9 Hz, 8H, **d**), 7.69 (dt, J = 1.8, 0.9 Hz, 8H, **e**), 7.57 (d, J = 1.7 Hz, 4H, **f**), 5.57 (t, J = 5.4 Hz, 4H, **g**), 4.67 (d, J = 5.4 Hz, 8H, **h**).

All the analytical data obtained was in accordance with the literature[3].

Synthesis of MOC3

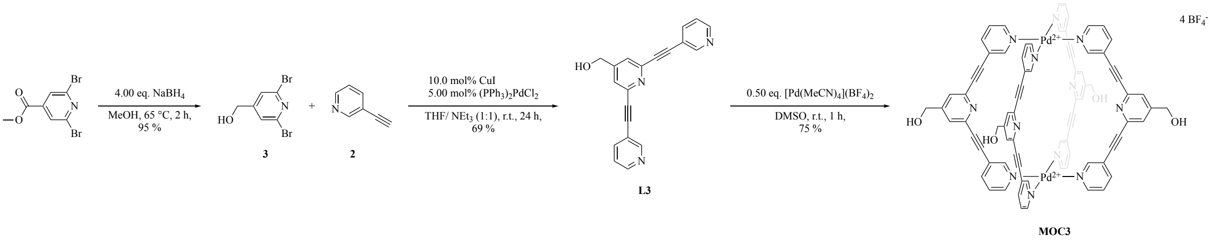

Figure S3: Synthetic overview of cage **MOC3**.

Synthesis of (2,6-dibromopyridin-4-yl)methanol (3)

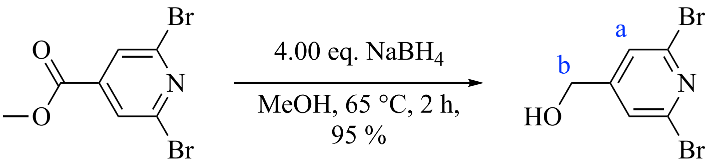

3

Methyl 2,6-dibromoisonicotinate (2.27 g, 7.70 mmol, 1.00 eq.) was dissolved in 34.0 mL of methanol, and sodium borohydride (1.17 g, 30.8 mmol, 4.00 eq.) was added slowly at room temperature over the course of 10 minutes. After complete addition, the mixture was heated to 65 °C for three hours. To the now orange solution, 20.0 mL of 1M HCl was added, and the mixture was concentrated to approximately 10.0 mL volume. The mixture was neutralized by the addition of sodium carbonate, and the resulting suspension was further stirred for two hours. The white precipitate was dissolved in 20.0 mL of dichloromethane and diluted with 20.0 mL of water. The organic phase was extracted three times with 20.0 mL of dichloromethane and subsequently dried over magnesium sulfate, yielding the crude product after solvent removal. Purification of the crude product by column chromatography (cyclohexane/ ethyl acetate 3:1) yields **3** (1.96 g, 7.34 mmol, 95 %) as a colorless powder.

^{1}**H NMR** (300 MHz, $CDCl_3$): δ 7.47 (t, J = 0.9 Hz, 2H, **a**), 4.72 (dt, J = 5.8, 0.9 Hz, 2H, **b**). All the analytical data obtained was in accordance with the literature[5].

Synthesis of (2,6-bis(pyridin-3-ylethynyl)pyridin-4-yl)methanol (L3)

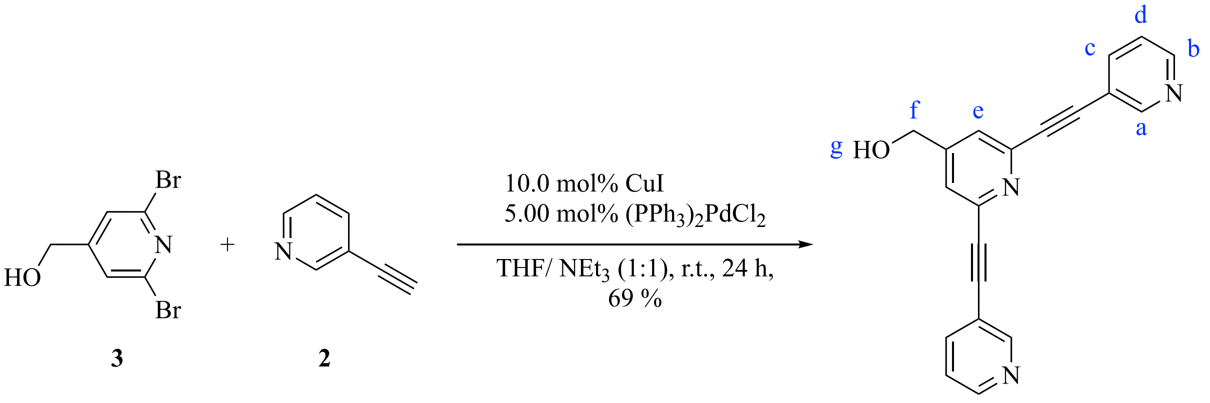

3 **2** **L3**

2 (364 mg, 3.53 mmol, 2.80 eq.), **3** (336 mg, 1.26 mol, 1.00 eq.), copper(I) iodide (24.0 mg, 0.13 mmol, 10 mol%) and dichlorobis(triphenylphosphine)palladium(II) (44.2 mg, 0.06 mmol, 5.00 mol%) were added to a 100 mL, nitrogen-flushed Schlenk flask. To this mixture 25.0 mL of dry tetrahydrofuran and triethylamine were added, and the yellow suspension was stirred for

24 hours in the dark. The reaction mixture was diluted with 50.0 mL of water and extracted three times with 100 mL of dichloromethane. The organic phase was washed two times with 50.0 mL of brine before the organic phase was dried over magnesium sulfate. The solvent was removed under reduced pressure to give the crude product. Column chromatography (dichloromethane/ acetone 2:1) yields pure **L3** (0.27 g, 0.87 mmol, 69 %) as an off-white solid.

^{1}H NMR (300 MHz, CD_3CN): δ 8.81 (dd, J = 2.2, 0.9 Hz, 2H, **a**), 8.60 (dd, J = 4.9, 1.7 Hz, 2H, **b**), 7.97 (ddd, J = 7.9, 2.2, 1.7 Hz, 2H, **c**), 7.58 (t, J = 0.9 Hz, 2H, **d**), 7.41 (ddd, J = 7.9, 4.9, 0.9 Hz, 2H, **e**), 4.71 – 4.62 (m, 2H, **f**), 3.55 (t, J = 5.9 Hz, 1H **g**).
All the analytical data obtained was in accordance with the literature[6].

Synthesis of MOC3

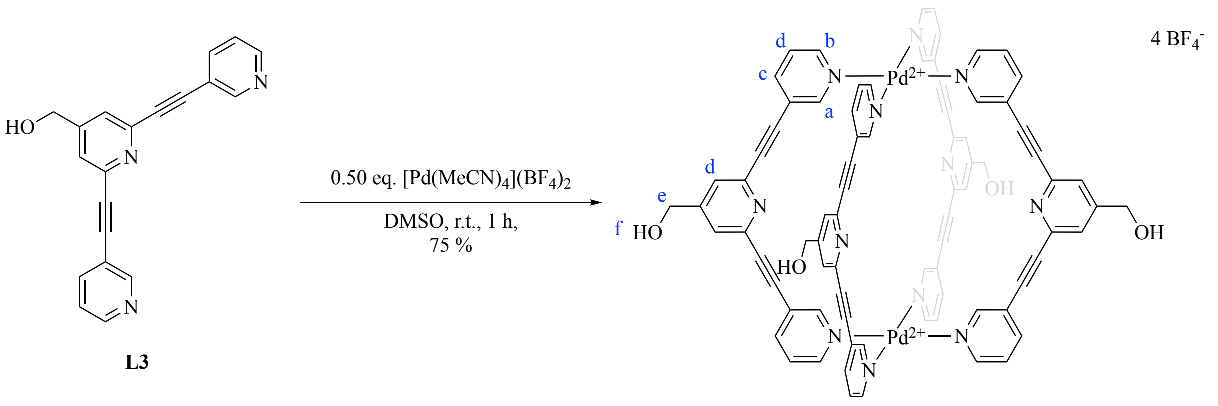

L3

MOC3

Tetrakis(acetonitrile)palladium(II) tetrafluoroborate (22.9 mg, 52.0 µmol, 1.00 eq.) and **L3** (32.1 mg, 103 µmol, 2.00 eq.) were dissolved in 2.05 mL dimethyl sulfoxide and stirred for one hour at room temperature. The addition of ethyl acetate led to the precipitation of the crude product. The off-white solid was washed with ethyl acetate and diethyl ether and dried in air to yield **MOC3** (35.0 mg, 19.4 µmol, 75 %).

^{1}H NMR (600 MHz, CD_3CN): δ 9.33 (s, 8H, **a**), 9.07 – 9.02 (m, 8H, **b**), 8.16 (dt, J = 8.0, 1.6 Hz, 8H, **c**), 7.68 – 7.62 (m, 16H, **d**), 4.63 (dt, J = 5.6, 0.9 Hz, 8H, **e**), 3.56 (t, J = 5.5 Hz, 4H, **f**).
All the analytical data obtained was in accordance with the literature[6].

Synthesis of PolyMOC1

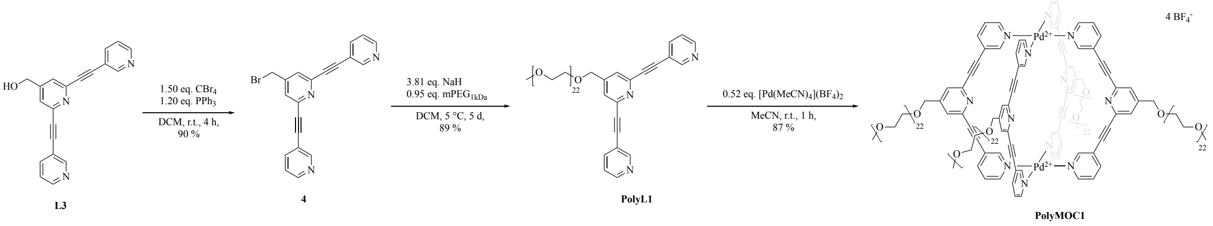

Figure S4: Synthetic overview of cage **PolyMOC1**.

Synthesis of 3,3'-((4-(bromomethyl)pyridine-2,6-diyl)bis(ethyne-2,1-diyl))dipyridine (4)

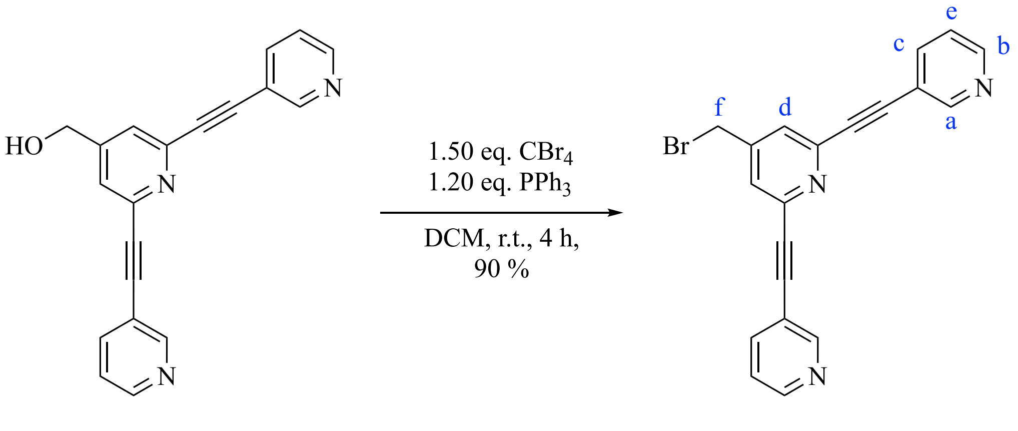

L3 **4**

L3 (202 mg, 0.65 mmol, 1.00 eq.), triphenylphosphine (205 mg, 0.78 mmol, 1.20 eq.), and carbon tetrabromide (323 mg, 0.98 mmol, 1.50 eq.) were added to a 100 mL Schlenk flask and purged with argon in the dark before 10 mL of dry dichloromethane was added. The reaction mixture was stirred in the absence of light for four hours. The orange reaction mixture was directly purified by column chromatography (dichloromethane/ acetone 4:1) *via* wet loading to obtain **4** (220 mg, 0.59 mmol, 90 %) as a colorless powder.

^{1}H NMR (600 MHz, $CDCl_3$): δ 8.85 (dd, J = 2.2, 0.9 Hz, 2H, **a**), 8.62 (dd, J = 5.0, 1.7 Hz, 2H, **b**), 7.93 (dt, J = 7.9, 1.9 Hz, 2H, **c**), 7.57 (d, J = 0.6 Hz, 2H, **d**), 7.37 (ddd, J = 7.9, 5.0, 0.9 Hz, 2H, **e**), 4.41 (s, 2H, **f**).

All the analytical data obtained was in accordance with the literature[7].

Synthesis of PolyL1

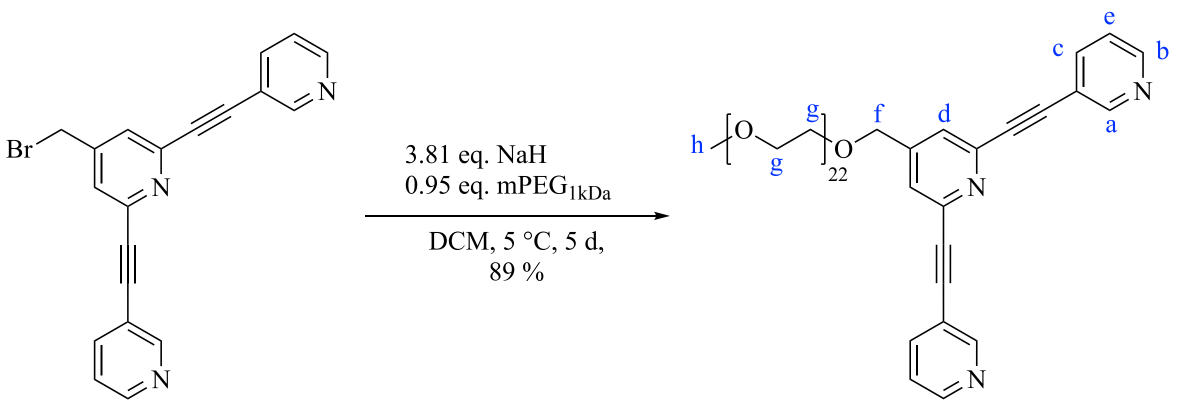

4

PolyL1

Poly(ethylene glycol) monomethyl ether (M_n = 1 kDa, 0.34 g, 0.34 mmol, 1.00 eq.) was added to a 25.0 mL Schlenk flask, purged with nitrogen, and dissolved in 15.0 mL of dry dichloromethane. The colorless solution was cooled to 5 °C, subsequently, sodium hydride (54.0 mg, 1.36 mmol, 4.00 eq.) was added. The resulting suspension was stirred for one hour. Then, compound **4** (0.134 g, 0.36 mmol, 1.05 eq.) was added, and the mixture was stirred for five days at 5 °C before the reaction was quenched with 0.75 mL of saturated ammonium chloride solution. The reaction was stirred for one hour and then filtered into a separation funnel. 30.0 mL of water and 3.00 mL of saturated ammonium chloride solution were added, and the aqueous phase was extracted thrice with 30.0 mL of dichloromethane. The combined organic phases were washed twice with 75.0 mL of water before the aqueous phase was further extracted two times with 25.0 mL of dichloromethane. The combined organic layers were dried over magnesium sulfate before the solvent was removed under reduced pressure. The crude product was dissolved in 50.0 mL of water and was washed twice with 50.0 mL of diethyl ether. Then, the aqueous phase was dried under reduced pressure to yield **PolyL1** (392 mg, 0.30 mmol, 89 %) as an off-white wax-like solid.

^{1}H NMR (600 MHz, CD_3CN): δ 9.01 – 8.72 (m, 2H, **a**), 8.61 (dd, J = 4.9, 1.6 Hz, 2H, **b**), 7.97 (dd, J = 7.9, 1.8 Hz, 2H, **c**), 7.59 (d, J = 1.0 Hz, 2H, **d**), 7.50 – 7.24 (m, 2H, **e**), 4.62 (s, 2H, **f**), 3.55 (s, 88H, **g**), 3.29 (s, 3H, **h**); **^{13}C{^{1}H} NMR** (151 MHz, CD_3CN): δ 153.35 (**1**), 151.22 (**2**), 150.69 (**3**), 143.95 (**4**), 139.96 (**5**), 125.94 (**6**), 124.46 (**7**), 119.92 (**8**), 91.86 (**9**), 86.44 (**10**), 71.18 (**11**), 71.01 (**12**), 58.91 (**13**); **FT-IR (ATR)**: 2883.58, 1465.90, 1417.68, 1342.46, 1342.46, 1280.73, 1240.23, 1145.72, 1105.21, 1060.85, 962.48, 842.89, 705.95; **MALDI-MS**: $[C_{65}H_{103}N_3O_{23}+ H]^+$ calculated: 1295.5 m/z, found: 1316.8m/z.

Synthesis of PolyMOC1

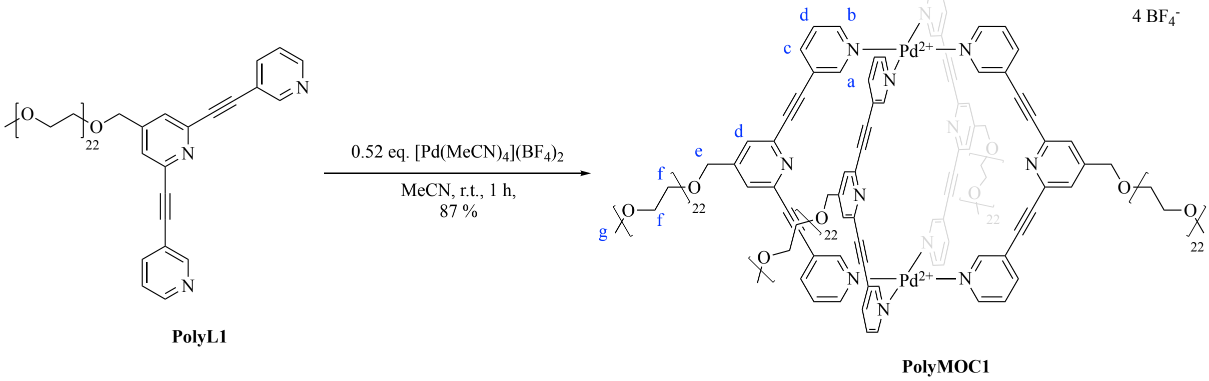

PolyL1 (284 mg, 219 µmol, 1.00 eq.) and tetrakis(acetonitrile)palladium(II) tetrafluoroborate (51.0 mg, 115 µmol, 0.52 eq.) were dissolved in 4.60 mL acetonitrile, and the reaction mixture was stirred vigorously for one hour at room temperature. The solvent was removed under reduced pressure to yield **PolyMOC1** (0.20 g, 47.9 µmol, 87 %) as an off-white solid.

^{1}H NMR (600 MHz, CD_3CN): δ 9.33 (s, 8H, **a**), 9.06 (dd, J = 4.8, 3.0 Hz, 8H, **b**), 8.46 – 7.97 (m, 8H, **c**), 8.05 – 6.76 (m, 16H, **d**), 4.59 (s, 8H, **e**), 3.55 (s, 352H, **f**), 3.29 (s, 12H, **g**); **^{13}C{^{1}H} NMR**: owing to the high intensity PEG signal and the low concentration of the other molecular components, no meaningful ^{13}C{^{1}H} NMR spectrum could be obtained; **FT-IR (ATR)**: 2866.22, 1465.90, 1344.38, 1280.73, 1242.16, 1197.79, 1097.50, 1060.85, 947.05, 842.89, 692.44; **Mp.**: 55 °C.

Synthesis of PolyMOC2

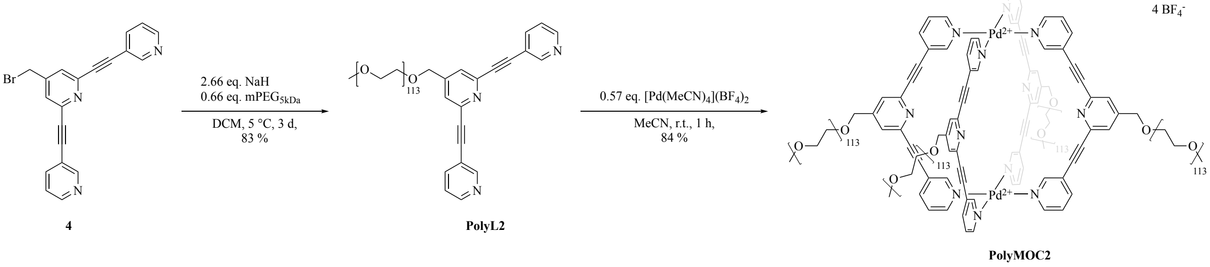

Figure S5: Synthetic overview of cage **PolyMOC2**.

Synthesis of PolyL2

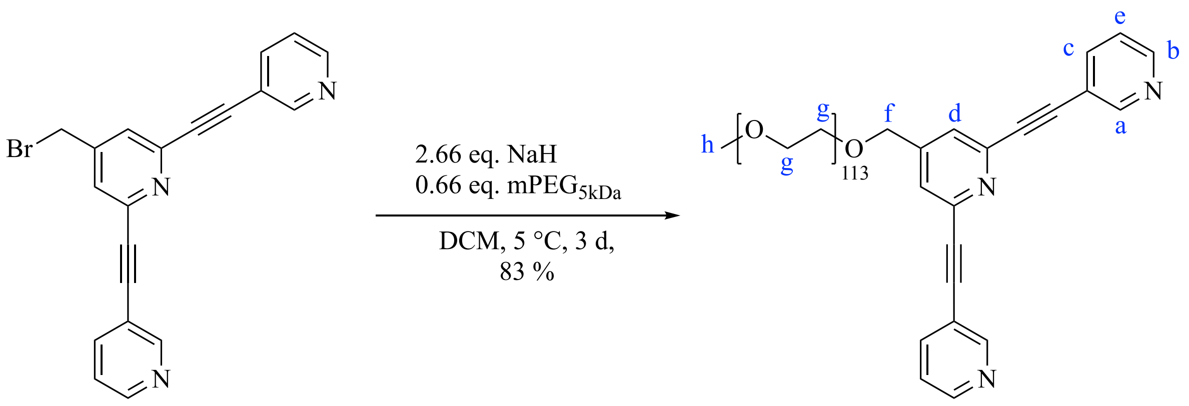

4 **PolyL2**

Poly(ethylene glycol) monomethyl ether (M_n = 5 kDa, 1.25 g, 0.25 mmol, 1.00 eq.) was added to a 25.0 mL Schlenk flask, purged with nitrogen, and dissolved in 17.0 mL of dry dichloromethane. The colorless solution was cooled to 5 °C, subsequently, sodium hydride (40.0 mg, 1.00 mmol, 4.00 eq.) was added. The resulting suspension was stirred for one hour. Then, compound **4** (0.14 g, 0.38 mmol, 1.50 eq.) was added, and the mixture was stirred for three days at 5 °C before the reaction was quenched with 0.75 mL of saturated ammonium chloride solution. The reaction was stirred for one hour and then filtered into a separation funnel. 30.0 mL of water and 3.00 mL of saturated ammonium chloride solution were added, and the aqueous phase was extracted thrice with 30.0 mL of dichloromethane. The combined organic phases were washed twice with 75.0 mL of water before the aqueous phase was further extracted two times with 25.0 mL of dichloromethane. The combined organic layers were dried over magnesium sulfate before the solvent was removed under reduced pressure. The crude product was dissolved in dichloromethane and precipitated in cold diethyl ether. Filtration, followed by washing with diethyl ether and drying in air, yields **PolyL2** (1.10 g, 0.21 mmol, 83 %) as a colorless powder.

^{1}H NMR (600 MHz, CD_3CN): δ 8.81 (dd, J = 2.2, 0.9 Hz, 2H, **a**), 8.61 (dd, J = 4.9, 1.7 Hz, 2H, **b**), 7.98 (dt, J = 7.9, 1.9 Hz, 2H, **c**), 7.60 (d, J = 0.9 Hz, 2H, **d**), 7.42 (ddd, J = 7.9, 4.9, 0.9 Hz, 2H, **e**), 4.63 (s, 2H, **f**), 3.55 (s, 452H, **g**), 3.29 (s, 3H, **h**); **^{13}C{^{1}H} NMR** (151 MHz, CD_3CN): δ 153.33 (**1**), 151.21 (**2**), 150.69 (**3**), 143.91 (**4**), 139.96 (**5**), 125.92 (**6**), 124.46 (**7**), 119.89 (**8**), 91.84 (**9**), 86.43 (**10**), 71.17 (**11**), 71.00 (**12**), 58.90 (**13**); **FT-IR (ATR)**: 2881.65, 1465.90, 1454.33, 1359.82, 1340.53, 1278.81, 1240.23, 1145.72, 1095.57, 1060.85, 960.55, 947.05, 840.96; **MALDI-MS**: $[C_{247}H_{467}N_3O_{114}+ H]^+$ calculated: 5300.1 m/z, found: 5368.3 m/z.

Synthesis of PolyMOC2

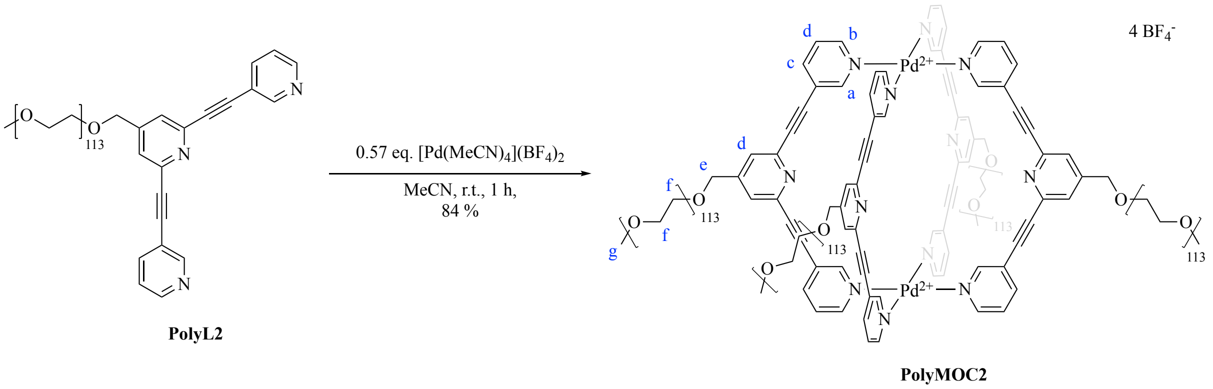

PolyL2 (0.49 g, 92.9 µmol, 1.00 eq.) and tetrakis(acetonitrile)palladium(II) tetrafluoroborate (23.6 mg, 53.1 µmol, 0.57 eq.) were dissolved in 2.10 mL acetonitrile, and the reaction mixture was stirred vigorously for one hour at room temperature. Precipitation in cold diethyl ether and subsequent washing with cold diethyl ether before drying in air yields **PolyMOC2** (0.43 g, 19.5 µmol, 84 %) as a colorless solid.

^{1}H NMR (600 MHz, CD_3CN): δ 9.30 (s, 8H, **a**), 9.03 (d, J = 5.9 Hz, 8H, **b**), 8.19 (d, J = 7.9 Hz, 8H, **c**), 7.89 – 7.01 (m, 16H, **d**), 4.59 (s, 8H, **e**), 3.55 (s, 1808H, **f**), 3.29 (s, 12H, **g**); **^{13}C{^{1}H} NMR**: owing to the high intensity PEG signal and the low concentration of the other molecular components, no meaningful ^{13}C{^{1}H} NMR spectrum could be obtained; **FT-IR (ATR)**: 2881.65, 1465.90, 1359.82, 1342.46, 1278.81, 1240.23, 1145.72, 1099.43, 1060.85, 960.55, 947.05, 840.96; **Mp.**: 65 °C.

Synthesis of PolyMOC3

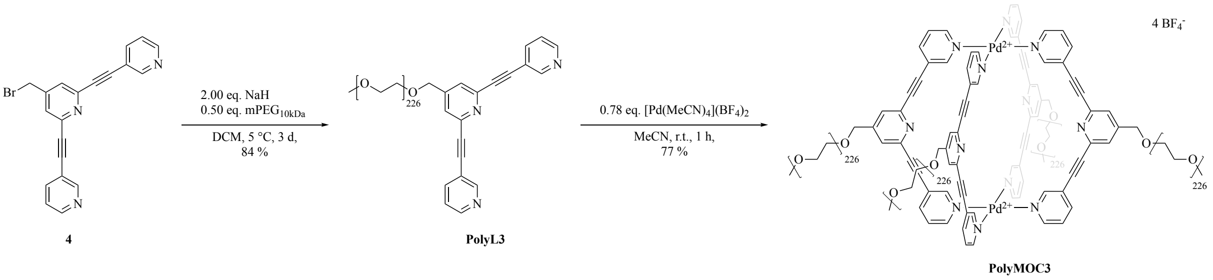

Figure S6: Synthetic overview of cage **PolyMOC3**.

Synthesis of PolyL3

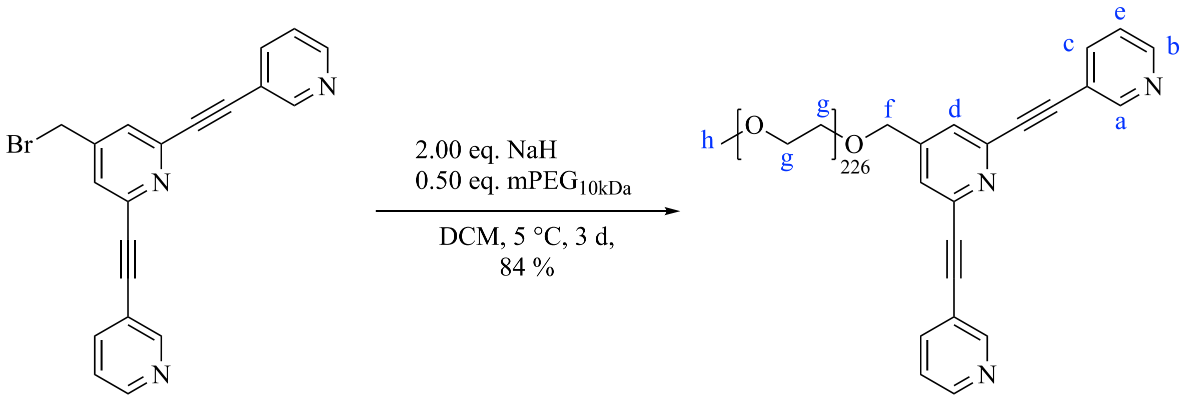

4

PolyL3

Poly(ethylene glycol) monomethyl ether (M_n = 10 kDa, 1.50 g, 0.15 mmol, 1.00 eq.) was added to a 25.0 mL Schlenk flask, purged with nitrogen, and dissolved in 12.0 mL of dry dichloromethane. The colorless solution was cooled to 5 °C, subsequently, sodium hydride (24.0 mg, 0.60 mmol, 4.00 eq.) was added. The resulting suspension was stirred for one hour. Then, compound **4** (0.11 g, 0.30 mmol, 2.00 eq.) was added, and the mixture was stirred for three days at 5 °C before the reaction was quenched with 0.50 mL of saturated ammonium chloride solution. The reaction was stirred for one hour and then filtered into a separation funnel. 20.0 mL of water and 2.00 mL of saturated ammonium chloride solution were added, and the aqueous phase was extracted thrice with 25.0 mL of dichloromethane. The combined organic phases were washed twice with 50.0 mL of water before the aqueous phase was further extracted two times with 25.0 mL of dichloromethane. The combined organic layers were dried over magnesium sulfate before the solvent was removed under reduced pressure. The crude product was dissolved in dichloromethane and precipitated in cold diethyl ether. Filtration, followed by washing with diethyl ether and drying in air yields **PolyL3** (1.25 g, 0.12 mmol, 81 %) as a colorless powder.

^{1}H NMR (600 MHz, CD_3CN): δ 8.81 (dd, J = 2.2, 0.9 Hz, 2H, **a**), 8.61 (dd, J = 4.9, 1.7 Hz, 2H, **b**), 8.05 – 7.89 (m, 2H, **c**), 7.60 (s, 2H, **d**), 7.42 (ddd, J = 7.9, 4.9, 0.9 Hz, 2H, **e**), 4.63 (d, J = 0.9 Hz, 2H, **f**), 3.55 (s, 904H, **g**), 3.29 (s, 3H, **h**); **^{13}C{^{1}H} NMR**: owing to the high intensity PEG signal and the low concentration of the other molecular components, no meaningful ^{13}C{^{1}H} NMR spectrum could be obtained; **FT-IR (ATR)**: 2881.65, 2860.43, 1465.90, 1340.53, 1278.81, 1240.23, 1145.72, 1097.50, 1058.92, 960.55, 947.05, 840.96; **MALDI-MS**: $[C_{473}H_{919}N_3O_{227} + H]^+$ calculated: 10276.1 m/z, found: 9686.2 m/z.

Synthesis of PolyMOC3

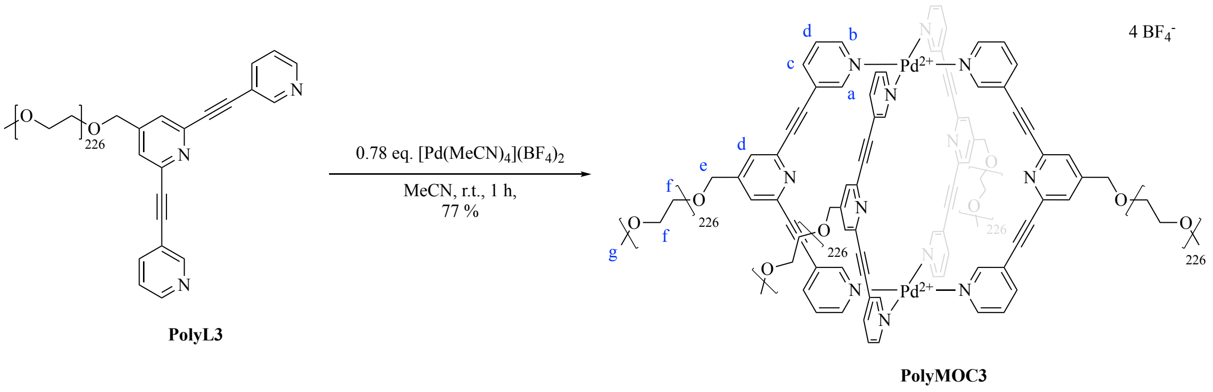

PolyL3 (0.45 g, 43.5 µmol, 1.00 eq.) and tetrakis(acetonitrile)palladium(II) tetrafluoroborate (15.0 mg, 33.7 µmol, 0.78 eq.) were dissolved in 1.74 mL acetonitrile, and the reaction mixture was stirred vigorously for one hour at room temperature. Precipitation in cold diethyl ether and subsequent washing with cold diethyl ether before drying in air, yields **PolyMOC3** (0.35 g, 8.40 µmol, 77 %) as a colorless solid.

^{1}H NMR (600 MHz, CD_3CN): δ 9.30 (s, 8H, **a**), 9.03 (d, J = 5.9 Hz, 8H, **b**), 8.19 (d, J = 7.9 Hz, 8H, **c**), 7.84 – 7.44 (m, 16H, **d**), 4.59 (s, 8H, **e**), 3.55 (s, 3616H, **f**), 3.29 (s, 12H, **g**); **^{13}C{^{1}H} NMR**: owing to the high intensity PEG signal and the low concentration of the other molecular components, no meaningful ^{13}C{^{1}H} NMR spectrum could be obtained; **FT-IR (ATR)**: 2883.58, 1465.90, 1359.82, 1342.46, 1278.81, 1242.16, 1145.72, 1097.50, 1058.92, 960.55, 947.05, 840.96; **Mp.**: 70 °C.

Synthesis of PolyMOC4

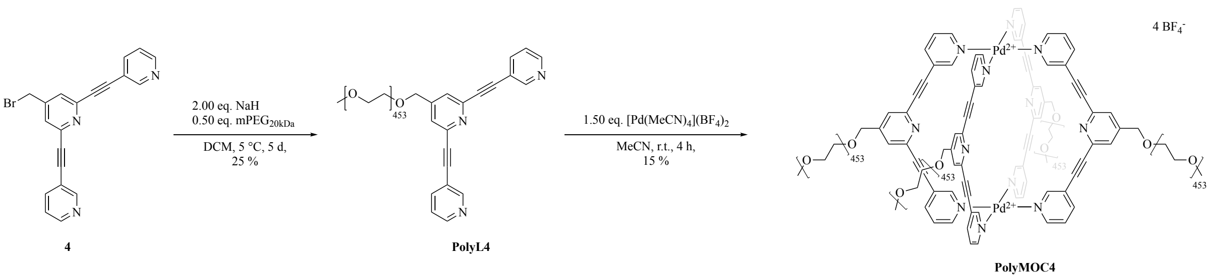

Figure S7: Synthetic overview of cage **PolyMOC4**.

Synthesis of PolyL4

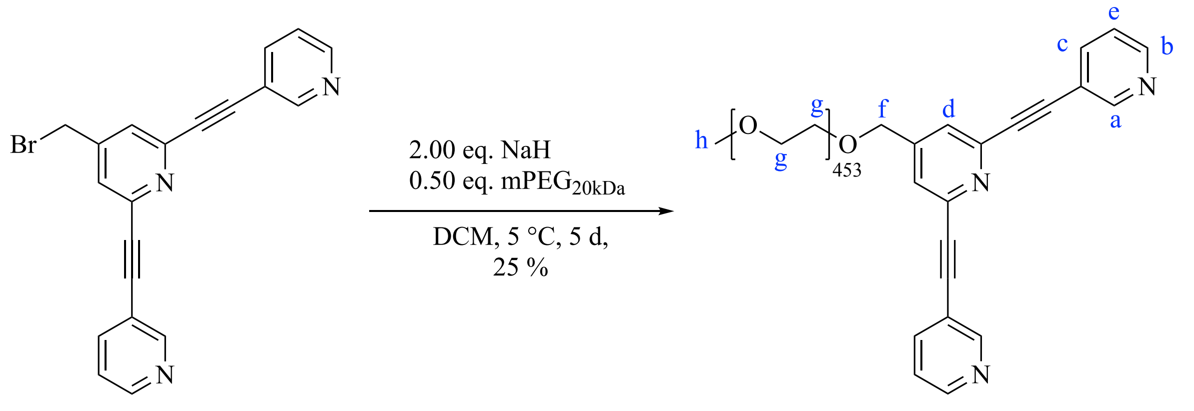

4 **PolyL4**

Poly(ethylene glycol) monomethyl ether (M_n = 20 kDa, 1.48 g, 74.0 µmol, 1.00 eq.) was added to a 25.0 mL Schlenk flask, purged with argon, and dissolved in 9.00 mL of dry dichloromethane. The colorless solution was cooled to 5 °C, subsequently, sodium hydride (12.0 mg, 0.30 mmol, 4.00 eq.) was added. The resulting suspension was stirred for one hour. Then, compound **4** (55.4 mg, 148 µmol, 2.00 eq.) was added, and the mixture was stirred for five days at 5 °C before the reaction was quenched with 0.25 mL of saturated ammonium chloride solution. The reaction mixture was stirred for one hour and then filtered into a separation funnel. 15.0 mL of water and 2.00 mL of saturated ammonium chloride solution were added, and the aqueous phase was extracted thrice with 25 mL of dichloromethane. The combined organic phases were washed twice with 50 mL of water before the aqueous phase was further extracted two times with 25 mL of dichloromethane. The combined organic layers were dried over magnesium sulfate before the solvent was removed under reduced pressure. The crude product was dissolved in dichloromethane and precipitated in cold diethyl ether. Filtration, followed by washing with diethyl ether and drying in air, yields a 1:2 mixture of **PolyL4** and **mPEG$_{20kDa}$** (1.12 g, 55.2 µmol, 75 %) as a colorless powder, containing **PolyL4** (0.37 g, 18.2 µmol, 25 %). The mass fraction was determined by the ^{1}H NMR spectrum (Figure S160). The product mixture was used without further purification.

^{1}H NMR (600 MHz, CD_3CN): δ 8.81 (s, 2H, **a**), 8.61 (d, J = 5.0 Hz, 2H, **b**), 7.97 (d, J = 8.2 Hz, 2H, **c**), 7.60 (s, 2H, **d**), 7.50 – 7.25 (m, 2H, **e**), 4.63 (s, 2H, **f**), 3.55 (s, 1812H, **g**), 3.29 (s, 3H, **h**); 3.55 (s, 3624H, PEG-**a**), 3.29 (s, 6H, PEG-**b**), 2.80 – 2.66 (m, 2H, PEG-**c**); **^{13}C{^{1}H} NMR**: owing to the high intensity PEG signal and the low concentration of the other molecular components, no meaningful ^{13}C{^{1}H} NMR spectrum could be obtained; **FT-IR (ATR)**: 2881.65, 1465.90, 1359.82, 1340.53, 1278.81, 1240.23, 1145.72, 1097.50, 1060.85, 960.55,

947.05, 840.96; **MALDI-MS**: $[C_{927}H_{1828}N_3O_{454} + H]^+$ calculated: 20270.0 m/z, found: 20942.9 m/z.

Synthesis of PolyMOC4

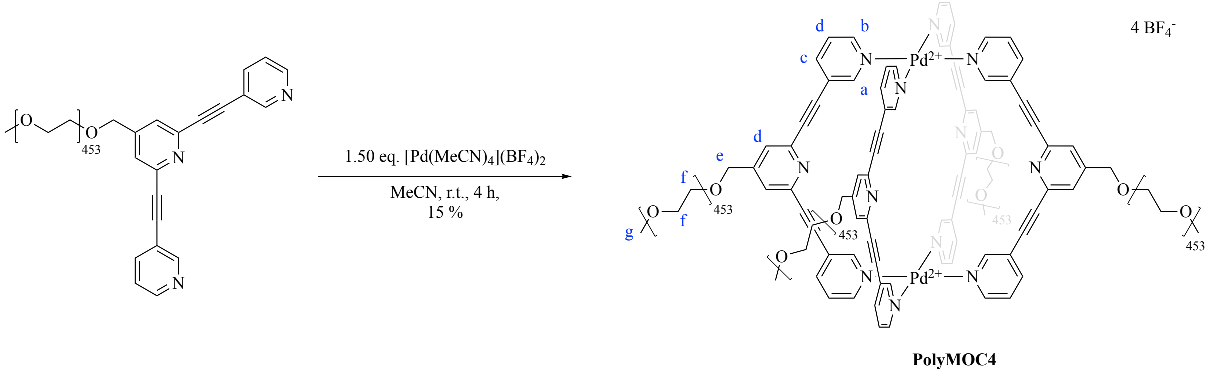

The mixture of **PolyL4** and **mPEG$_{20kDa}$** (0.20 g, 9.85 µmol, 1.00 eq.), containing **PolyL4** (66.9 mg, 3.33 µmol, 1.00 eq.) and tetrakis(acetonitrile)palladium(II) tetrafluoroborate (2.00 mg, 5.00 µmol, 1.50 eq.), was dissolved in 1.00 mL acetonitrile, and the reaction mixture was stirred vigorously for four hours at room temperature. Precipitation in cold diethyl ether and subsequent washing with cold diethyl ether before drying in air yields a 1:16 mixture of **PolyMOC4** and **mPEG$_{20kDa}$** (0.17 g, 8.12 µmol, 83 %), containing **PolyMOC4** (10.0 mg, 0.12 µmol, 15 %) as a colorless solid.

^{1}H NMR (600 MHz, CD_3CN): δ 9.30 (s, 8H, **a**), 9.03 (d, J = 5.9 Hz, 8H, **b**), 8.19 (d, J = 7.9 Hz, 8H, **c**), 7.88 – 7.02 (m, 16H, **d**), 4.59 (s, 8H, **e**), 3.55 (s, 7248H, **f**), 3.29 (s, 12H, **g**); 3.55 (s, 28992H, **PEG-a**), 3.29 (s, 48H, **PEG-b**), 2.77 (t, J = 5.9 Hz, 16H, **PEG-c**); **^{13}C{^{1}H} NMR**: owing to the high intensity PEG signal and the low concentration of the other molecular components, no meaningful ^{13}C{^{1}H} NMR spectrum could be obtained; **FT-IR (ATR)**: 2881.65, 1465.90, 1359.33, 1342.46, 1278.81, 1240.23, 1145.72, 1099.43, 1060.85, 960.55, 947.05, 840.96; **Mp.**: 70 °C

Synthesis of MOS1 ($Pd_{12}L4_{24}$)

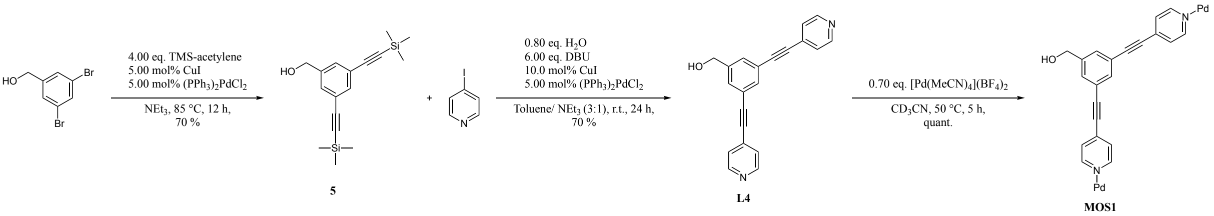

Figure S8: Synthetic overview of metal-organic sphere **MOS1**.

Synthesis of (3,5-bis((trimethylsilyl)ethynyl)phenyl)methanol (5)

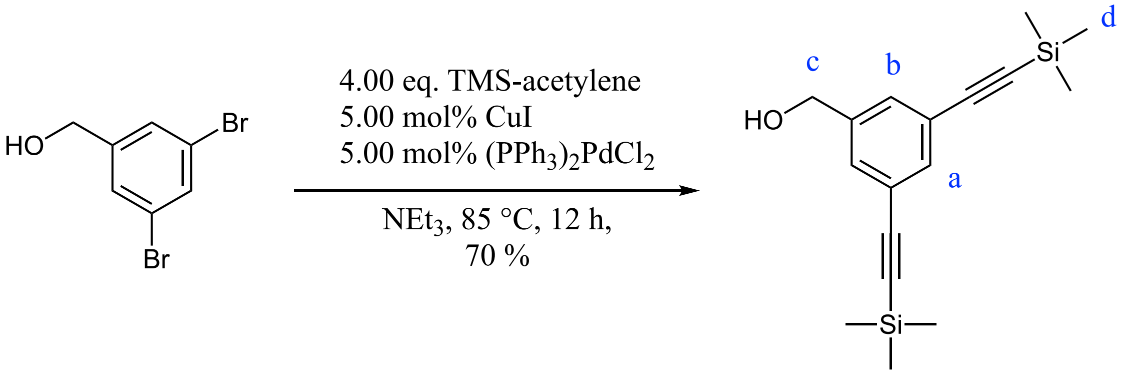

5

3,5-Dibromobenzylalcohol (5.32 g, 20.0 mmol, 1.00 eq.), copper(I) iodide (190 mg, 1.00 mmol, 5.00 mol%) and dichlorobis(triphenylphosphine)palladium(II) (702 mg, 1.00 mmol, 5.00 mol%) were added to a 100 mL, nitrogen-flushed Schlenk flask. To this 45.0 mL of triethylamine was added, followed by the addition of trimethylsilylacetylene (11.4 mL, 80.0 mmol, 4.00 eq.). The mixture was stirred at 85 °C for twelve hours. The reaction mixture was allowed to cool to room temperature and was diluted with 50.0 mL of diethyl ether and 100 mL of water. The mixture was extracted three times with 120 mL of dichloromethane, and the combined organic phases were dried over magnesium sulfate. The solvent was removed under reduced pressure to give the crude product. Column chromatography (*n*-hexane/ ethyl acetate 95:5) yields pure **5** (4.19 g, 13.9 mmol, 70 %) as a brown solid.

^{1}H NMR (600 MHz, $CDCl_3$): δ 7.51 (t, J = 1.5 Hz, 1H, **a**), 7.41 (dt, J = 1.5, 0.7 Hz, 2H, **b**), 4.66 – 4.62 (m, 2H, **c**), 0.24 (s, 18H, **d**).
All the analytical data obtained was in accordance with the literature[8].

Synthesis of (3,5-bis(pyridin-4-ylethynyl)phenyl)methanol (L4)

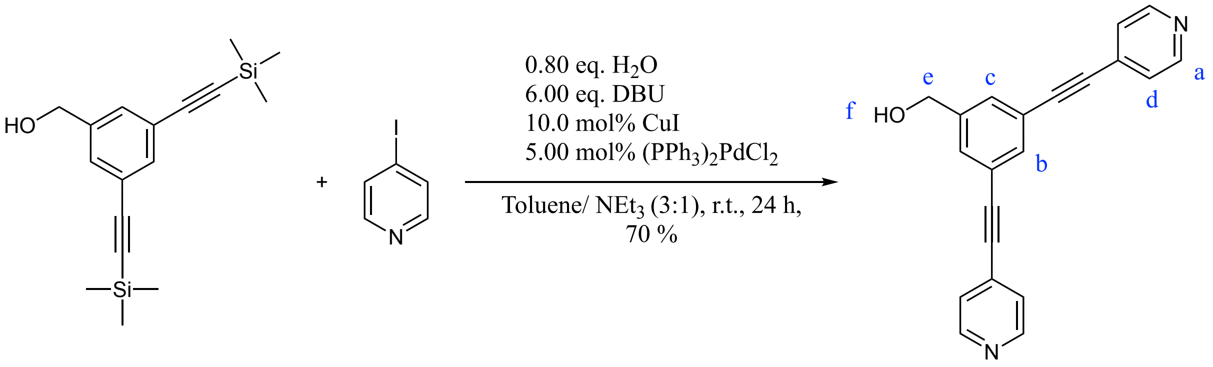

5 **L4**

A 100 mL Schlenk flask was charged with **5** (2.00 g, 6.64 mmol, 1.00 eq.), 4-iodopyridine (5.03 g, 24.6 mmol, 3.70 eq.), copper(I) iodide (126 mg, 0.66 mmol, 10.0 mol%) and dichlorobis(triphenylphosphine)palladium(II) (233 mg, 0.33 mmol, 5.00 mol%) and was purged with nitrogen. The solids were suspended in 33.0 mL of dry toluene and 11.0 mL of triethylamine. To this suspension, DBU (5.94 mL, 39.8 mmol, 6.00 eq.) and distilled water (96.0 µL, 5.31 mmol, 0.80 eq.) were added. The reaction mixture was stirred in the absence of light for 24 hours. The dark suspension was filtered into a separation funnel and was diluted with 100 mL of ethyl acetate. The organic phase was washed once with 100 mL of brine and once with 100 mL of water before drying over magnesium sulfate. The solvent was removed under reduced pressure, and the crude product was purified by column chromatography (**1.** cyclohexane/ ethyl acetate 1:1, **2.** ethyl acetate) to give **L4** (1.45 g, 4.66 mmol, 70 %) as a bright yellow solid.

^{1}H NMR (600 MHz, CD_3CN): δ 8.63 – 8.59 (m, 4H, **a**), 7.68 (d, J = 2.0 Hz, 1H, **b**), 7.61 (s, 2H, **c**), 7.48 – 7.44 (m, 4H, **d**), 4.63 (d, J = 5.8 Hz, 2H, **e**), 3.42 (t, J = 5.9 Hz, 1H, **f**); **^{1}H DOSY NMR** (600 MHz, CD_3CN): log(D) = -8.90, D = 1.25 x10^{-9} m^2 s^{-1}.
All the analytical data obtained was in accordance with the literature[9].

Synthesis of MOS1 ($Pd_{12}L4_{24}$)

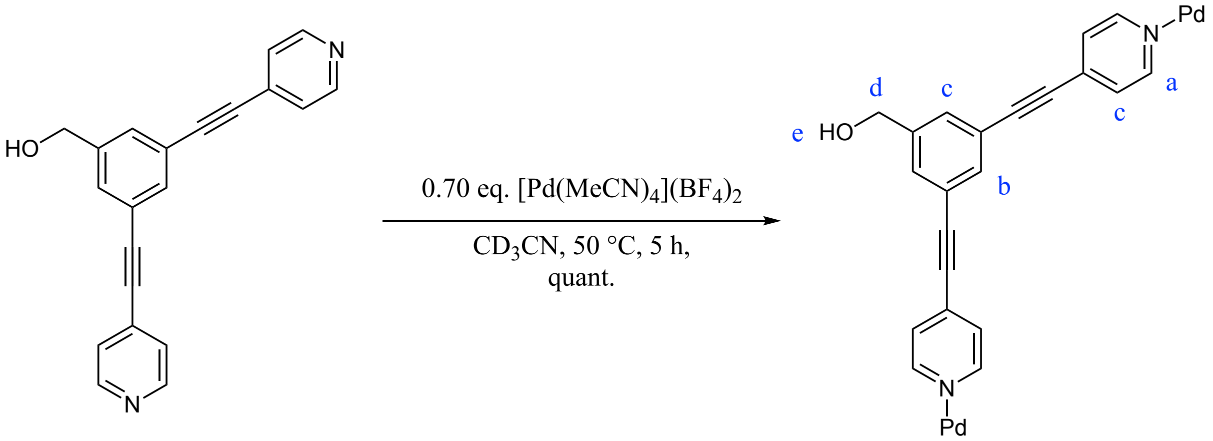

L4

MOS1

A 5 ml flask was charged with **L4** (4.30 mg, 14.0 µmol, 1.00 eq.) and tetrakis(acetonitrile)palladium(II) tetrafluoroborate (4.40 mg, 10.0 µmol, 0.70 eq.). To this mixture 920 µL of deuterated acetonitrile was added, and the suspension was stirred at 50 °C for five hours. The reaction was monitored by ^{1}H NMR analysis and shows quantitative formation of **MOS1** (6.33 mg, 0.58 µmol, quant.) after five hours.

^{1}H NMR (600 MHz, CD_3CN): δ 8.91 (d, J = 6.2 Hz, 96H, **a**), 7.72 (s, 24H, **b**), 7.73 – 6.93 (m, 144H, **c**), 4.59 (d, J = 5.7 Hz, 48H, **d**), 3.56 – 3.35 (m, 24H, **e**); **^{13}C{^{1}H} NMR** (151 MHz, CD_3CN): δ 151.99 (**1**), 145.21 (**2**), 136.39 (**3**), 135.36 (**4**), 132.70 (**5**), 129.91 (**6**), 122.56 (**7**), 98.31 (**8**), 86.45 (**9**), 63.22 (**10**); **^{1}H DOSY NMR** (600 MHz, CD_3CN): log(D) = -9.60, D = 2.50 x10^{-10} m^2 s^{-1}, **ESI-MS**: $[C_{504}H_{386}N_{48}O_{24}Pd_{12}B_{15}F_{60}]^{9+}$ calculated: 1119.13 m/z, found: 1117.28 m/z, $[C_{504}H_{386}N_{48}O_{24}Pd_{12}B_{14}F_{56}]^{10+}$ calculated: 998.61 m/z, found: 996.95 m/z, $[C_{504}H_{386}N_{48}O_{24}Pd_{12}B_{13}F_{52}]^{11+}$ calculated: 899.92 m/z, found: 898.23 m/z, $[C_{504}H_{386}N_{48}O_{24}Pd_{12}B_{12}F_{48}]^{12+}$ calculated: 817.67 m/z, found: 814.13 m/z, $[C_{504}H_{386}N_{48}O_{24}Pd_{12}B_{9}F_{36}]^{15+}$ calculated: 636.87 m/z, found: 638.06 m/z, $[C_{504}H_{386}N_{48}O_{24}Pd_{12}B_{8}F_{32}]^{16+}$ calculated: 591.69 m/z, found: 594.45 m/z, $[C_{504}H_{386}N_{48}O_{24}Pd_{12}B_{8}F_{32}]^{17+}$ calculated: 551.71 m/z, found: 550.09 m/z.

Synthesis of PolyMOS1 ($Pd_{12}L4_{24}$)

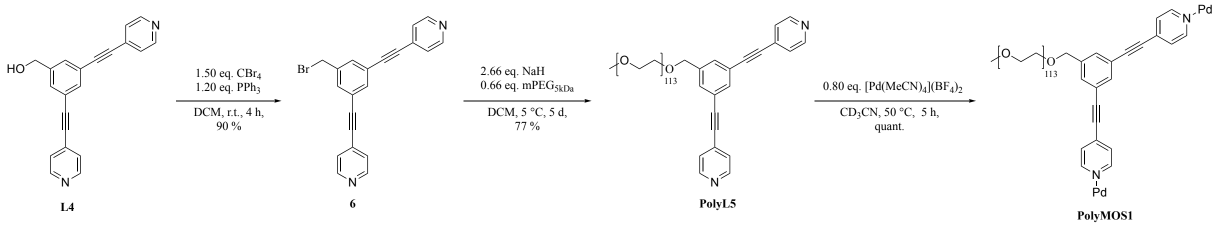

Figure S9: Synthetic overview of metal-organic sphere **PolyMOS1**.

Synthesis of 4,4'-((5-(bromomethyl)-1,3-phenylene)bis(ethyne-2,1-diyl))dipyridine (6)

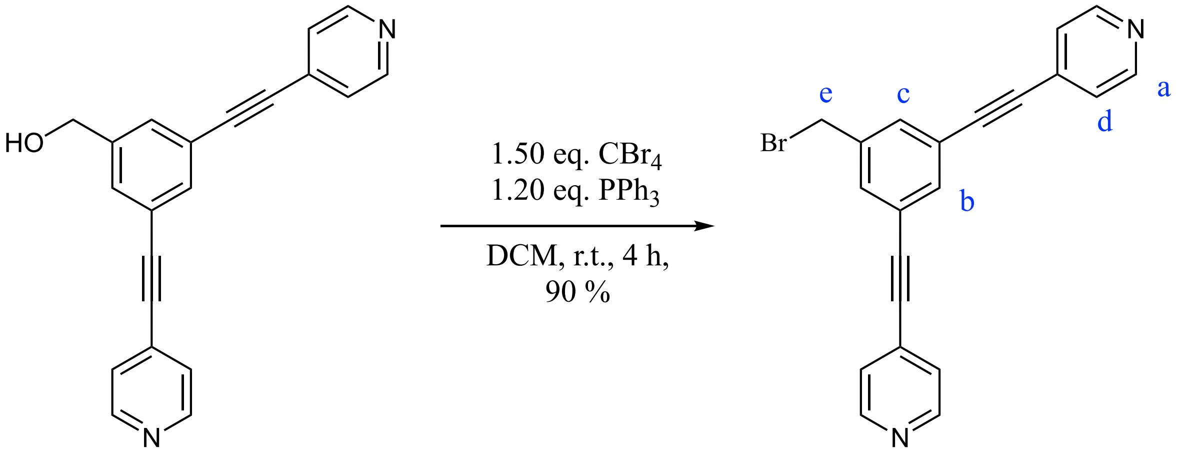

L4 **6**

L4 (200 mg, 0.65 mmol, 1.00 eq.), triphenylphosphine (203 mg, 0.77 mmol, 1.20 eq.), and carbon tetrabromide (321 mg, 0.97 mmol, 1.50 eq.) were added to a 25 mL Schlenk flask and purged with argon in the dark before 10 mL of dry dichloromethane was added. The reaction mixture was stirred in the absence of light for four hours. The orange reaction mixture was

directly purified by column chromatography (dichloromethane/ acetone 4:1) *via* wet loading to obtain **4** (218 mg, 0.58 mmol, 90 %) as a colorless powder.

^{1}H NMR (600 MHz, $CDCl_3$): δ 8.64 (d, J = 6.0 Hz, 4H, **a**), 7.68 (t, J = 1.5 Hz, 1H, **b**), 7.60 (d, J = 1.5 Hz, 2H, **c**), 7.41 – 7.37 (m, 4H, **d**), 4.47 (s, 2H, **e**); **^{13}C{^{1}H} NMR** (151 MHz, $CDCl_3$): δ 150.08 (**1**), 139.00 (**2**), 134.91(**3**), 132.93 (**4**), 130.91 (**5**), 125.67 (**6**), 123.50 (**7**), 92.07 (**8**), 88.04 (**9**), 31.62 (**10**); **FT-IR (ATR)**: 3032.10, 2974.23, 2218.14, 1940.39, 1813.09, 1595.13, 1547.27, 1489.05, 1438.90, 1406.11, 1357.89, 1234.44, 1213.23, 989.48, 970.19, 883.40, 815.89, 796.60, 740.67, 686.66, 650.01; **ESI (HRMS)**: $[C_{21}H_{13}BrN_2 + H]^+$ calculated: 373.0340 m/z, found: 373.0342 m/z.

Synthesis of PolyL5

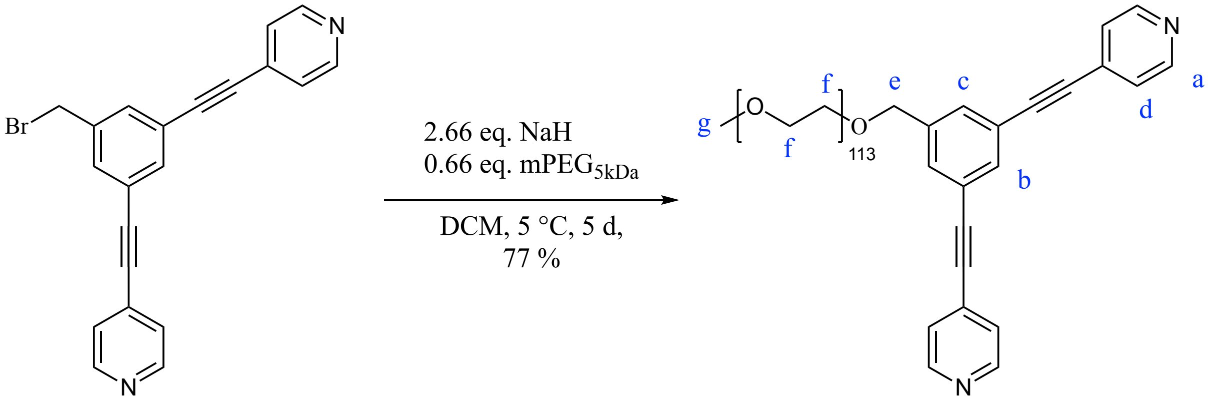

6 **PolyL5**

Poly(ethylene glycol) methyl ether (M_n = 5 kDa, 845 mg, 0.17 mmol, 1.00 eq.) was added to a 25.0 mL Schlenk flask, purged with nitrogen, and dissolved in 11.0 mL of dry dichloromethane. The colorless solution was cooled to 5 °C, subsequently, sodium hydride (27.0 mg, 0.68 mmol, 4.00 eq.) was added. The resulting suspension was stirred for one hour. Then, compound **6** (94.6 mg, 0.25 mmol, 1.50 eq.) was added, and the mixture was stirred for five days at 5 °C before the reaction was quenched with 0.60 mL of saturated ammonium chloride solution. The reaction was stirred for one hour and then filtered into a separation funnel. 20.0 mL of water and 2.00 mL of saturated ammonium chloride solution were added, and the aqueous phase was extracted thrice with 25.0 mL of dichloromethane. The combined organic phases were washed twice with 50.0 mL of water before the aqueous phase was further extracted two times with 25.0 mL of dichloromethane. The combined organic layers were dried over magnesium sulfate before the solvent was removed under reduced pressure. The crude product was dissolved in

dichloromethane and precipitated in cold diethyl ether. Filtration, followed by washing with diethyl ether and drying in air, yields **PolyL4** (686 mg, 0.13 mmol, 77 %) as a colorless powder.

^{1}H NMR (600 MHz, CD_3CN): δ 8.64 – 8.60 (m, 4H, **a**), 7.71 (s, 1H, **b**), 7.62 (s, 2H, **c**), 7.48 – 7.44 (m, 4H, **d**), 4.58 (s, 2H, **e**), 3.55 (s, 452H, **f**), 3.29 (s, 3H, **g**); **^{13}C{^{1}H} NMR** (151 MHz, CD_3CN): δ 151.04 (**1**), 141.74 (**2**), 134.57 (**3**), 132.28 (**4**), 131.39 (**5**), 126.38 (**6**), 123.61 (**7**), 92.94 (**8**), 88.20 (**9**), 72.18 (**10**), 71.16 (**11**), 58.89 (**12**); **FT-IR (ATR)**: 2945.30, 2881.65, 2740.85, 1595.13, 1465.90, 1456.26, 1411.89, 1359.82, 1340.53, 1278.81, 1240.23, 1145.72, 1099.43, 1060.85, 989.48, 960.55, 947.05, 840.96, 686.59; **MALDI-MS**: $[C_{248}H_{468}N_2O_{114} + H]^+$ calculated: 5300.1 m/z, found: 5455.3m/z.

Synthesis of PolyMOS1 ($Pd_{12}L4_{24}$)

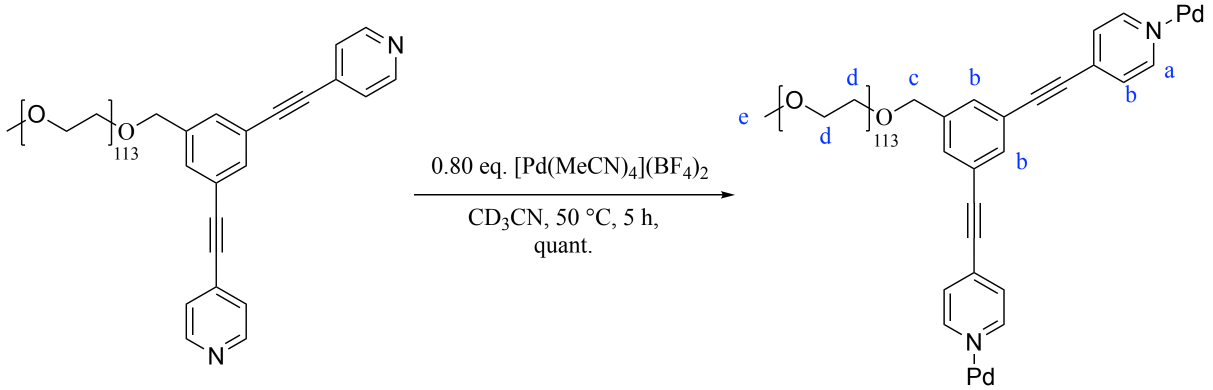

PolyL5

PolyMOS1

A 5 mL flask was charged with **PolyL4** (130 mg, 25.0 µmol, 1.00 eq.) and tetrakis(acetonitrile)palladium(II) tetrafluoroborate (8.70 mg, 20.0 µmol, 0.80 eq.). To this mixture 1.60 mL of deuterated acetonitrile was added, and the resulting solution was stirred at 50 °C for five hours. The reaction was monitored by ^{1}H NMR analysis and shows quantitative formation of **PolyMOS1** (MW = 133 kDa, 134 mg, 1.02 µmol, quant.) after five hours.

^{1}H NMR (600 MHz, CD_3CN): δ 9.22 – 8.72 (m, 96H, **a**), 7.90 – 7.04 (m, 168H, **b**), 4.54 (s, 48H, **c**), 3.55 (s, 10848H, **d**), 3.29 (s, 72H, **e**); **^{13}C{^{1}H} NMR**: owing to the high intensity PEG signal and the low concentration of the other molecular components, no meaningful ^{13}C{^{1}H} NMR spectrum could be obtained.

Cisplatin encapsulations

All encapsulation experiments were performed either as described in the literature or with modifications based on the procedure by Crowley *et al.*[10]

MOC1 cisplatin encapsulation

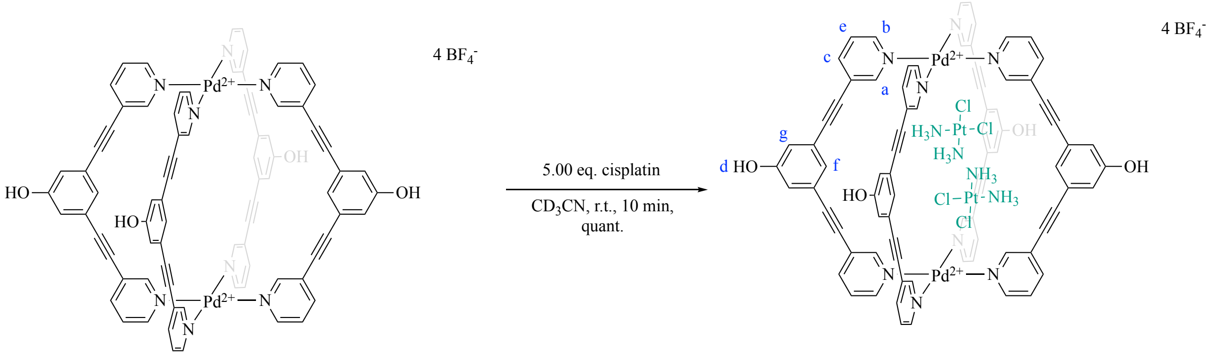

MOC1 **MOC1⊃(cisplatin)$_2$**

MOC1 (5.20 mg, 3.00 µmol, 1.00 eq.) was dissolved in 0.75 mL of CD_3CN, and cisplatin (4.50 mg, 15.0 µmol, 5 eq.) was added. The resulting yellow suspension was sonicated in an ultrasonic bath (45.0 kHz, 80.0 W) for ten minutes, followed by the removal of residual cisplatin *via* filtration through a syringe filter (0.70 µm) into an NMR tube. ^{1}H NMR analysis revealed the successful and quantitative encapsulation of cisplatin, resulting in **MOC1⊃(cisplatin)$_2$** (7.00 mg, 3.00 µmol, quant.).

^{1}H NMR (300 MHz, CD_3CN): δ 9.38 (s, 8H, **a**), 9.11 – 9.03 (m, 8H, **b**), 8.07 (dt, J = 8.1, 1.5 Hz, 8H, **c**), 7.77 (s, 4H, **d**), 7.62 (ddd, J = 8.1, 5.8, 0.7 Hz, 8H, **e**), 7.43 (t, J = 1.4 Hz, 4H, **f**), 7.11 (d, J = 1.4 Hz, 8H, **g**).

MOC2 cisplatin encapsulation

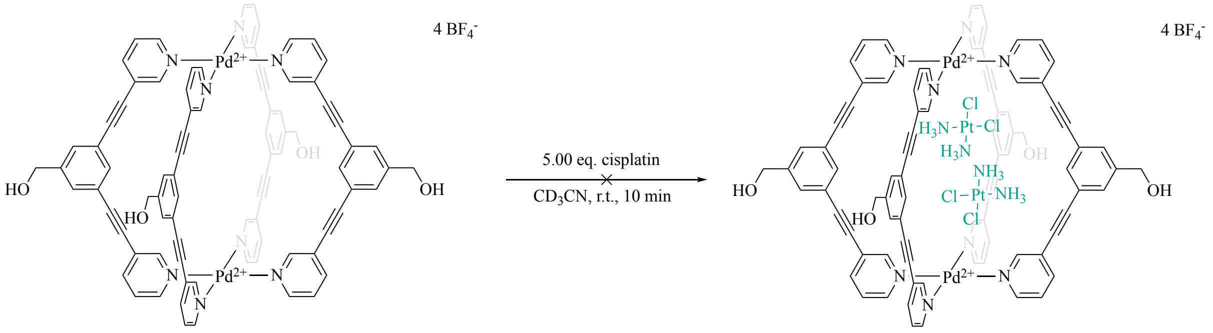

MOC2 **MOC2⊃(cisplatin)$_2$**

MOC2 (9.00 mg, 5.00 µmol, 1.00 eq.) was dissolved in 1.25 mL of CD_3CN, and cisplatin (7.50 mg, 25.0 µmol, 5.00 eq.) was added. The resulting yellow suspension was sonicated in an ultrasonic bath (45.0 kHz, 80.0 W) for ten minutes, followed by the removal of residual

cisplatin *via* filtration through a syringe filter (0.70 µm) into an NMR tube. ^{1}H NMR analysis revealed the unsuccessful encapsulation of cisplatin.

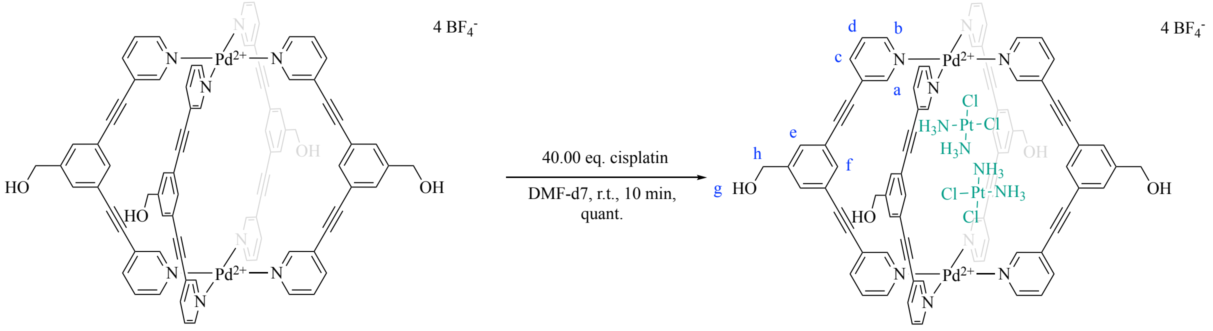

MOC2 **MOC2⊃(cisplatin)$_2$**

MOC2 (5.40 mg, 3.00 µmol, 1.00 eq.) was dissolved in 0.75 mL of DMF-d7, and cisplatin (4.47 mg, 15.0 µmol, 40.0 eq.) was added. The resulting yellow solution was stirred for ten minutes at room temperature. The reaction was monitored by ^{1}H NMR analysis. To this solution, a further five equivalents of cisplatin were added, and the solution was stirred for ten minutes before the reaction was monitored again by ^{1}H NMR analysis. The same procedure was repeated twice with additional 10.0 and 20.0 equivalents of cisplatin, resulting in **MOC2⊃(cisplatin)$_2$** (7.19 mg, 3.00 µmol, quant.).

^{1}H NMR (600 MHz, DMF-d7): δ 9.95 (s, 8H, **a**), 9.74 (d, J = 5.9 Hz, 8H, **b**), 8.39 (d, J = 7.9 Hz, 8H, **c**), 7.93 (dd, J = 7.9, 5.9 Hz, 8H, **d**), 7.69 (d, J = 1.5 Hz, 8H, **e**), 7.58 (s, 4H, **f**), 5.56 (t, J = 5.4 Hz, 4H, **g**), 4.66 (d, J = 5.4 Hz, 8H, **h**).

MOC3 cisplatin encapsulation

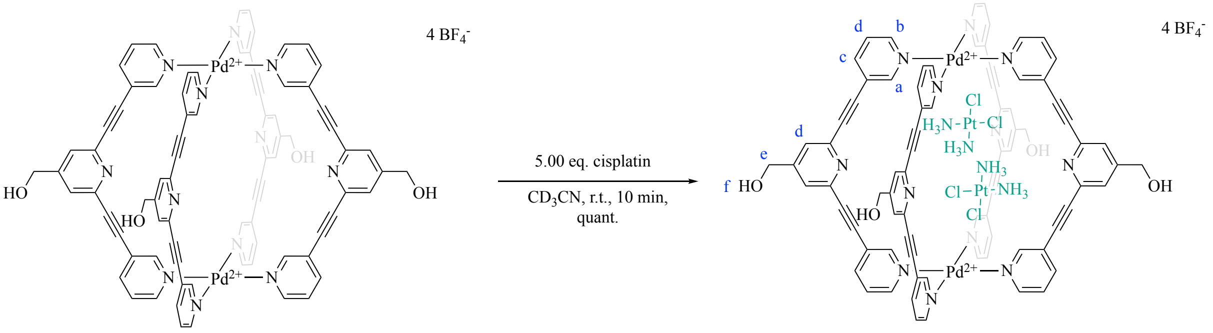

MOC3 **MOC3⊃(cisplatin)$_2$**

MOC3 (19.9 mg, 11.0 µmol, 1.00 eq.) was dissolved in 2.75 mL of CD_3CN, and cisplatin (16.4 mg, 55.0 µmol, 5.00 eq.) was added. The resulting yellow suspension was stirred vigorously at room temperature for ten minutes, followed by the removal of residual cisplatin *via* filtration through a syringe filter (0.70 µm) into an NMR tube. ^{1}H NMR analysis revealed

the successful and quantitative encapsulation of cisplatin, resulting in **MOC3⊃(cisplatin)₂** (26.0 mg, 11.0 µmol, quant.).

^{1}H NMR (600 MHz, CD_3CN): δ 9.43 (s (broad), 8H, **a**), 9.09 – 9.05 (m, 8H, **b**), 8.16 (dt, J = 8.0, 1.7 Hz, 8H, **c**), 7.84 – 6.74 (m, 16H, **d**), 4.63 (d, J = 5.5 Hz, 8H, **e**), 3.60 (t, J = 5.6 Hz, 4H, **f**).

PolyMOC2 cisplatin encapsulation

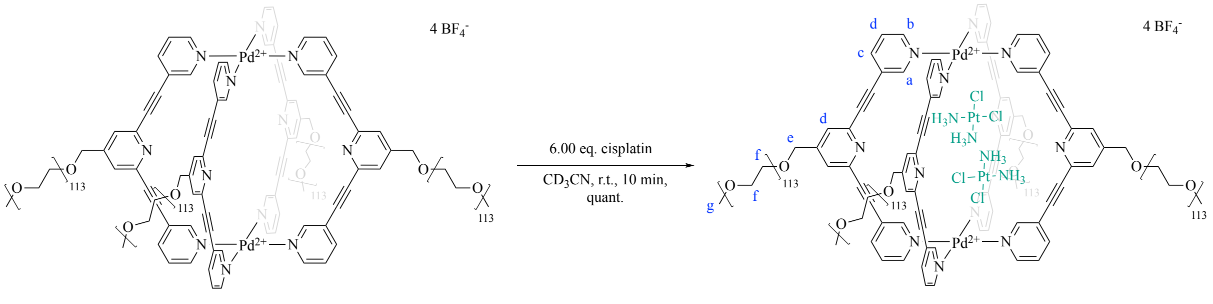

PolyMOC2 **PolyMOC2⊃(cisplatin)₂**

PolyMOC2 (21.8 mg, 1.00 µmol, 1.00 eq.) was dissolved in 0.50 mL of CD_3CN, and cisplatin (1.79 mg, 6.00 µmol, 6.00 eq.) was added. The resulting yellow suspension was stirred vigorously at room temperature for ten minutes, followed by the removal of residual cisplatin *via* filtration through a syringe filter (0.70 µm) into an NMR tube. ^{1}H NMR analysis revealed the successful and quantitative encapsulation of cisplatin, resulting in **PolyMOC2⊃(cisplatin)₂** (22.4 mg, 1.00 µmol, quant.).

^{1}H NMR (600 MHz, CD_3CN): δ 9.40 (s, 8H, **a**), 9.09 (d, J = 5.8 Hz, 8H, **b**), 8.19 (dt, J = 7.9, 1.6 Hz, 8H, **c**), 7.80 – 7.21 (m, 16H, **d**), 4.59 (s, 8H, **e**), 3.55 (s, 1808H, **f**), 3.29 (s, 12H, **g**).

PolyMOC3 cisplatin encapsulation

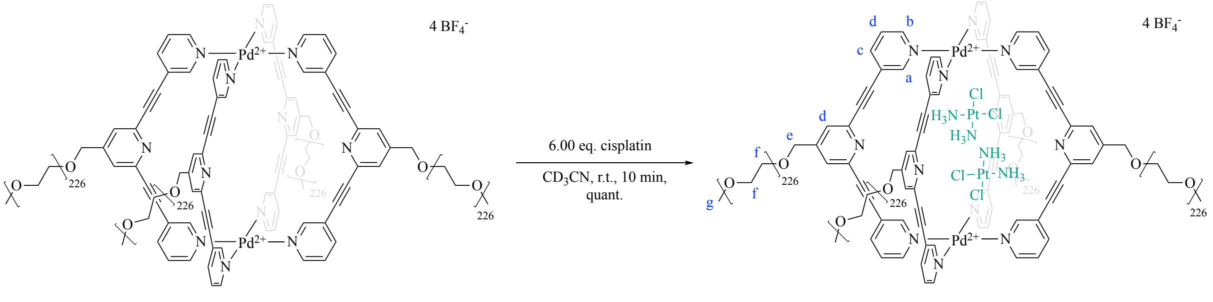

PolyMOC3 **PolyMOC3⊃(cisplatin)₂**

PolyMOC3 (42.0 mg, 1.00 µmol, 1.00 eq.) was dissolved in 0.50 mL of CD_3CN, and cisplatin (1.80 mg, 6.00 µmol, 6.00 eq.) was added. The resulting yellow suspension was stirred

vigorously at room temperature for ten minutes, followed by the removal of residual cisplatin *via* filtration through a syringe filter (0.70 µm) into an NMR tube. ^{1}H NMR analysis revealed the successful and quantitative encapsulation of cisplatin, resulting in **PolyMOC3⊃(cisplatin)$_2$** (42.3 mg, 1.00 µmol, quant.).

^{1}H NMR (600 MHz, CD_3CN): δ 9.40 (s, 8H, **a**), 9.10 (d, J = 5.9 Hz, 8H, **b**), 8.19 (d, J = 8.0 Hz, 8H, **c**), 7.79 – 7.41 (m, 16H, **d**), 4.59 (s, 8H, **e**), 3.55 (s, 3616H, **f**), 3.29 (s, 12H, **g**).

PolyMOC4 cisplatin encapsulation

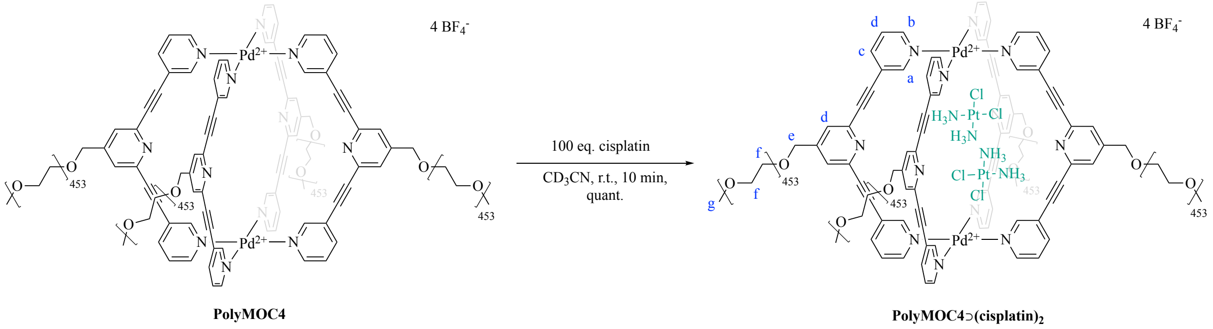

The 1:16 mixture of **PolyMOC4** and **mPEG$_{20kDa}$** (48.8 mg, 2.33 µmol, 1.00 eq.), containing **PolyMOC4** (2.87 mg, 35.3 nmol, 1.00 eq.), was dissolved in 0.30 mL of CD_3CN. Cisplatin (1.05 mg, 3.53 µmol, 100 eq.) was then added. The resulting yellow suspension was stirred vigorously at room temperature for ten minutes, followed by the removal of residual cisplatin *via* filtration through a syringe filter (0.70 µm) into an NMR tube. ^{1}H NMR analysis revealed the successful and quantitative encapsulation of cisplatin, resulting in **PolyMOC4⊃(cisplatin)$_2$** (2.89 mg, 35.3 nmol, quant.).

^{1}H NMR (600 MHz, CD_3CN): δ 9.39 (s, 8H, **a**), 9.10 (d, J = 5.8 Hz, 8H, **b**), 8.19 (d, J = 7.9 Hz, 8H, **c**), 8.00 – 7.12 (m, 16H, **d**), 4.59 (s, 8H, **e**), 3.55 (s, 7248H, **f**), 3.29 (s, 12H, **g**); 3.55 (s, 28992H, PEG-**a**), 3.29 (s, 48H, PEG-**b**), 2.81 (t, J = 6.0 Hz, 16H, PEG-**c**).

III. Stability of cisplatin inclusion complexes in aqueous media

To test whether a targeted release of cisplatin in water is possible with one of the cages, stability tests were performed. The respective cage was dissolved in CD_3CN, and cisplatin was encapsulated as described in Chapter II. Then, D_2O was added to the **MOC⊃(cisplatin)₂** complex solution in CD_3CN. To compare the signal shifts in the ^1H NMR spectrum, reference spectra of the cage in the same solvent mixture (CD_3CN/D_2O) were measured. The solution of **MOC2⊃(cisplatin)₂** in DMF-d7 (4.00 mM) was titrated with 0.50-11.0 vol% of D_2O.

MOC1⊃(cisplatin)₂

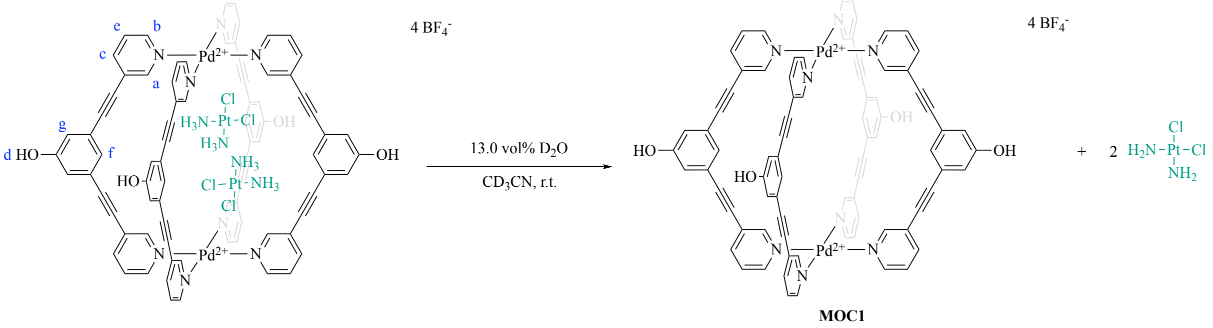

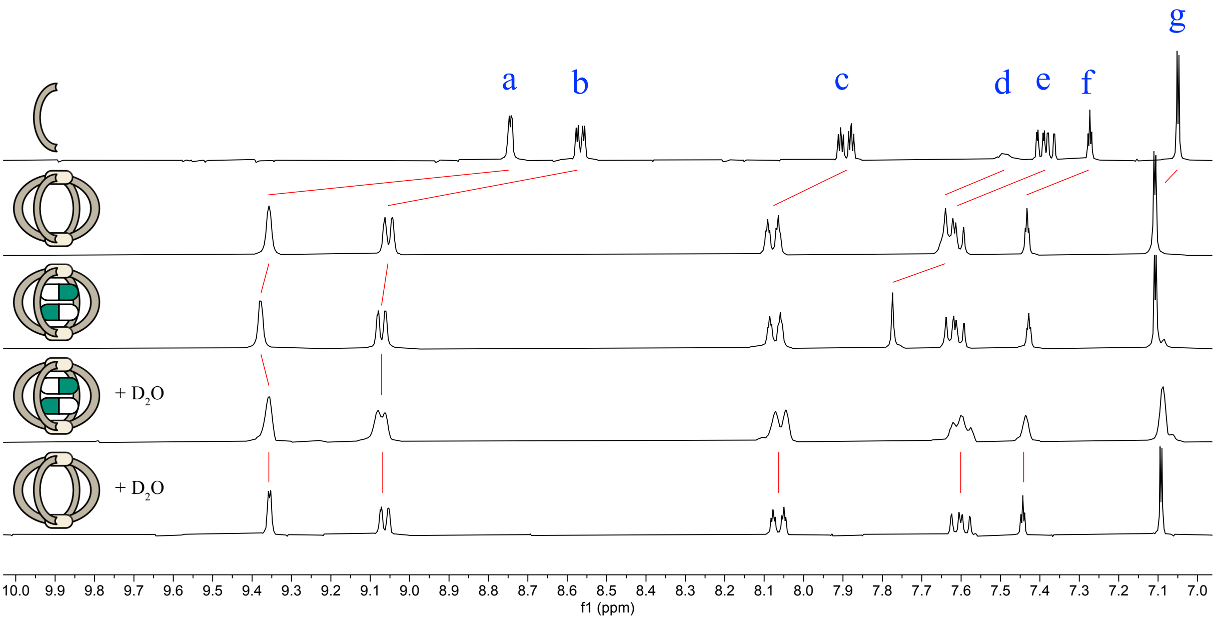

Figure S10: Stacked ^1H NMR spectra (300 MHz, CD_3CN, 298 K) spectra of **L1** (top), **MOC1, MOC1⊃(cisplatin)₂**, **MOC1⊃(cisplatin)₂** + D_2O (middle), and **MOC3** + D_2O (bottom).

MOC2⊃(cisplatin)₂

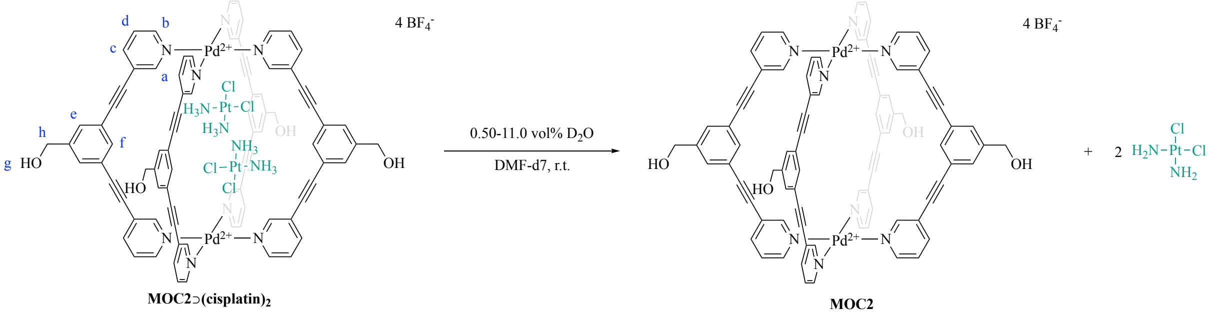

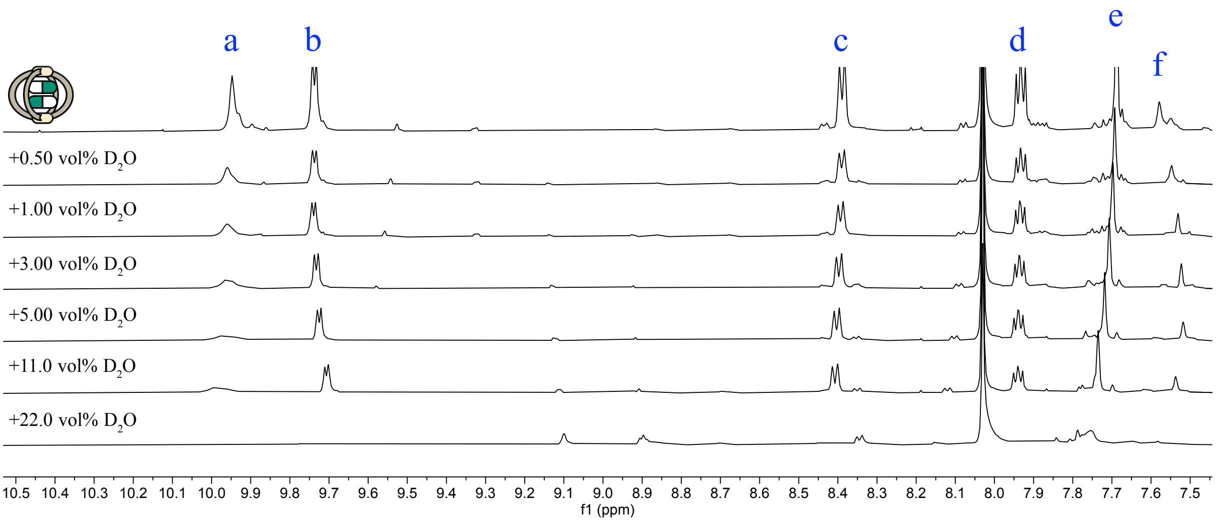

Figure S11: Stacked ^{1}H NMR spectra (600 MHz, DMF-d7, 298 K) spectra of **MOC2** (top), **MOC2** +0.50 vol% - 5.00 vol% D_2O(middle), and **MOC2** + 22.0 vol% D_2O (bottom).

MOC3⊃(cisplatin)₂

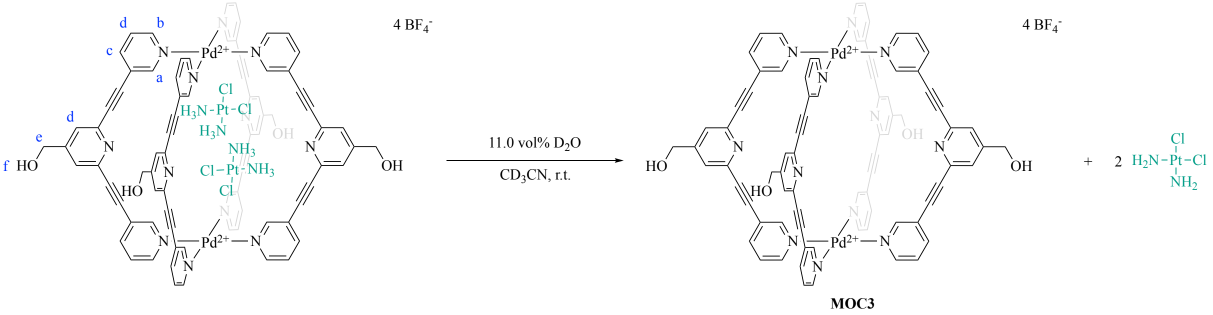

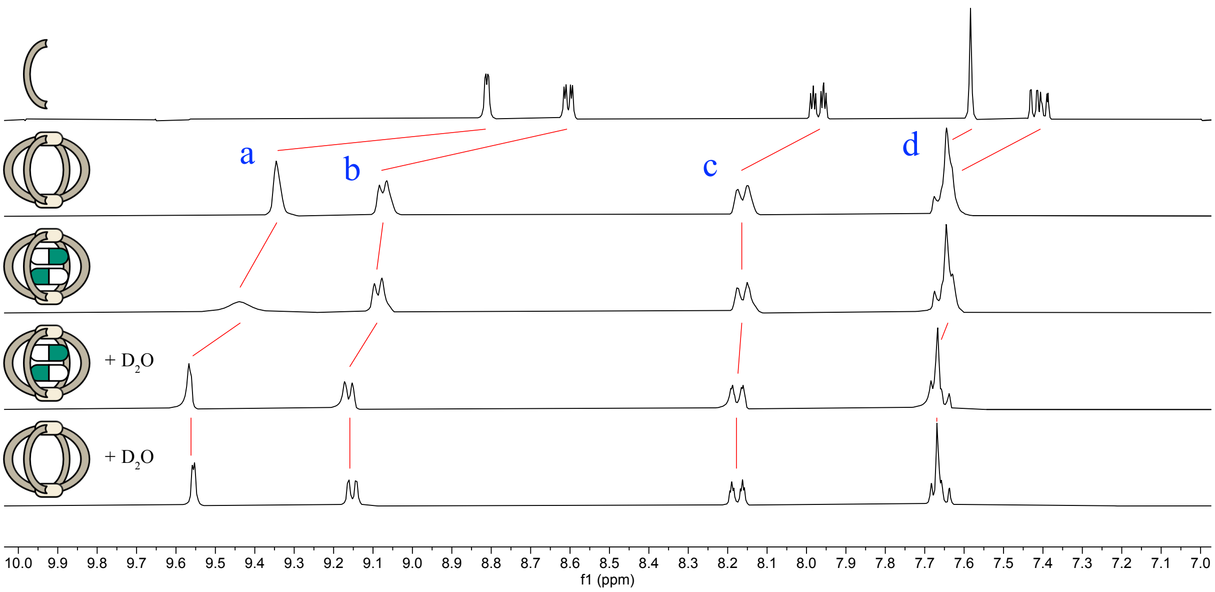

Figure S12: Stacked ^{1}H NMR spectra (300 MHz, CD_3CN, 298 K) spectra of **L3** (top), **MOC3, MOC3⊃(cisplatin)₂**, **MOC3⊃(cisplatin)₂** + D_2O (middle), and **MOC3** + D_2O (bottom).

PolyMOC2⊃(cisplatin)₂

To further investigate the behavior of the complex in aqueous media, a 1.00 mM solution of **PolyMOC2⊃(cisplatin)₂** or **PolyMOC2** (0.60 mL CD_3CN) was titrated stepwise with D_2O in the following amounts: 10 µL, 25 µL, 50 µL, 100 µL, 200 µL, and 400 µL. In both experiments, a similar shifting of the signals was observed. The signal for the proton (a) pointing into the cage, which forms a hydrogen bond with one of the chlorine atoms of cisplatin, sharpens noticeably upon the addition of just 100 µL. Simultaneously, signals corresponding to the free ligand emerge, indicating a partial opening of the cage. These observations strongly suggest the release of cisplatin from the cavity.

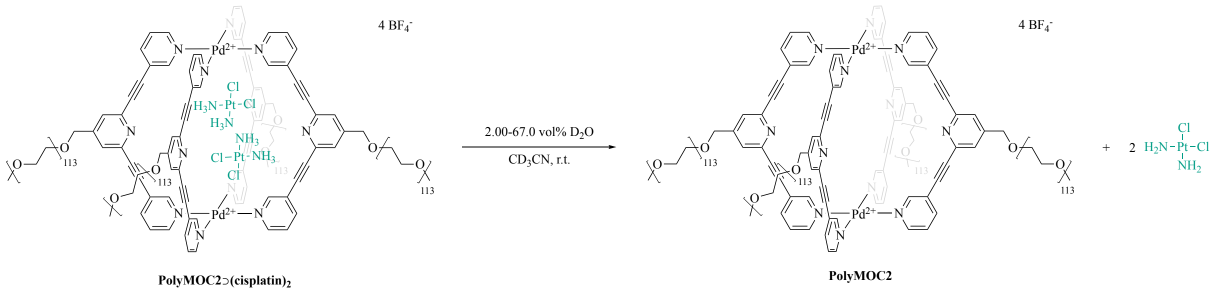

A 1.00 mM solution of **PolyMOC2⊃(cisplatin)₂** (0.60 mL CD_3CN) was titrated stepwise with D_2O in the following amounts: 10 µL, 25 µL, 50 µL, 100 µL, 200 µL, and 400 µL. The reaction was monitored by ^{1}H NMR analysis.

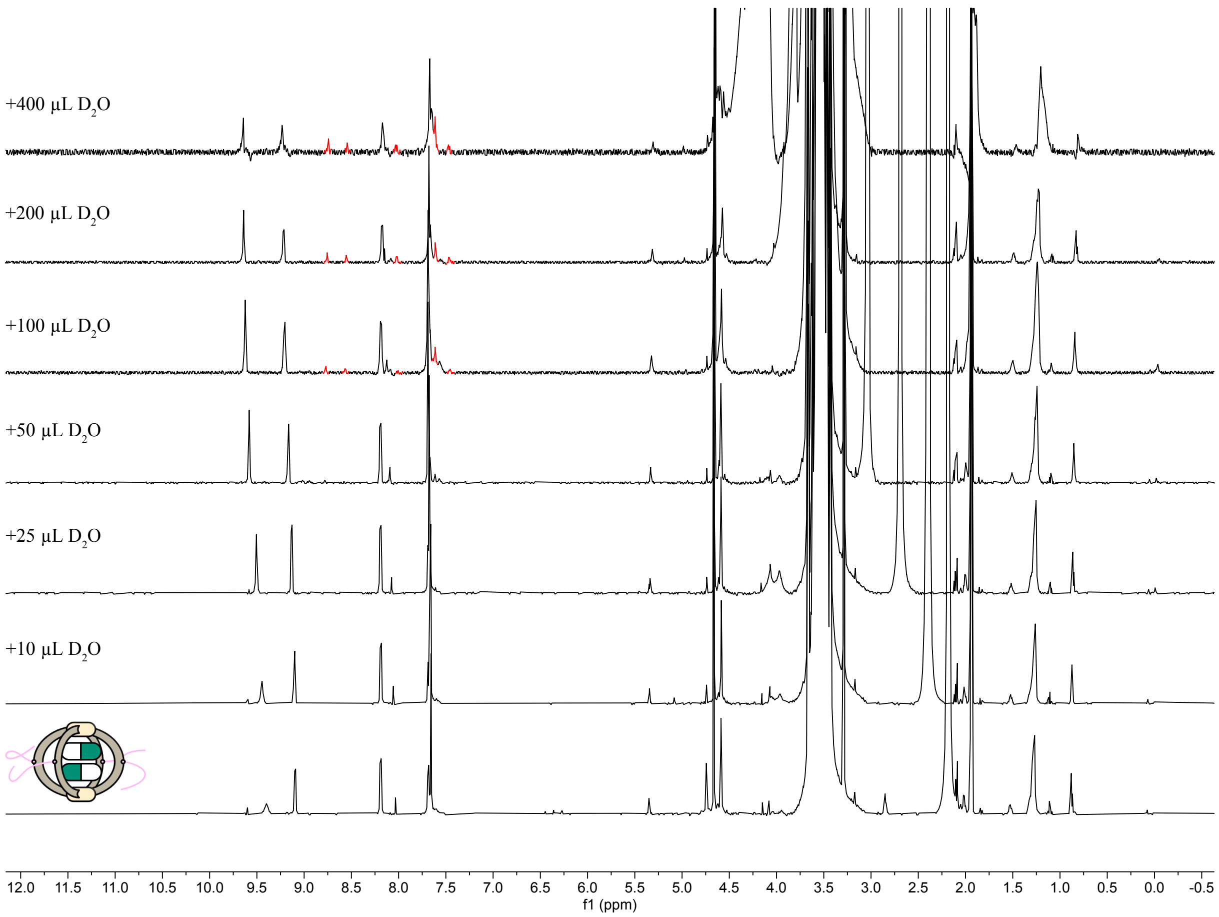

Figure S13: Stacked ^{1}H NMR spectra (600 MHz, CD_3CN, 298 K) showing the stepwise addition of D_2O to a 1.00 mM solution of **PolyMOC2⊃(cisplatin)₂** in CD_3CN. The bottom spectrum corresponds to the initial **PolyMOC2⊃(cisplatin)₂** complex before the addition of D_2O. The signals marked in red correspond to the free ligand (**PolyL2**).

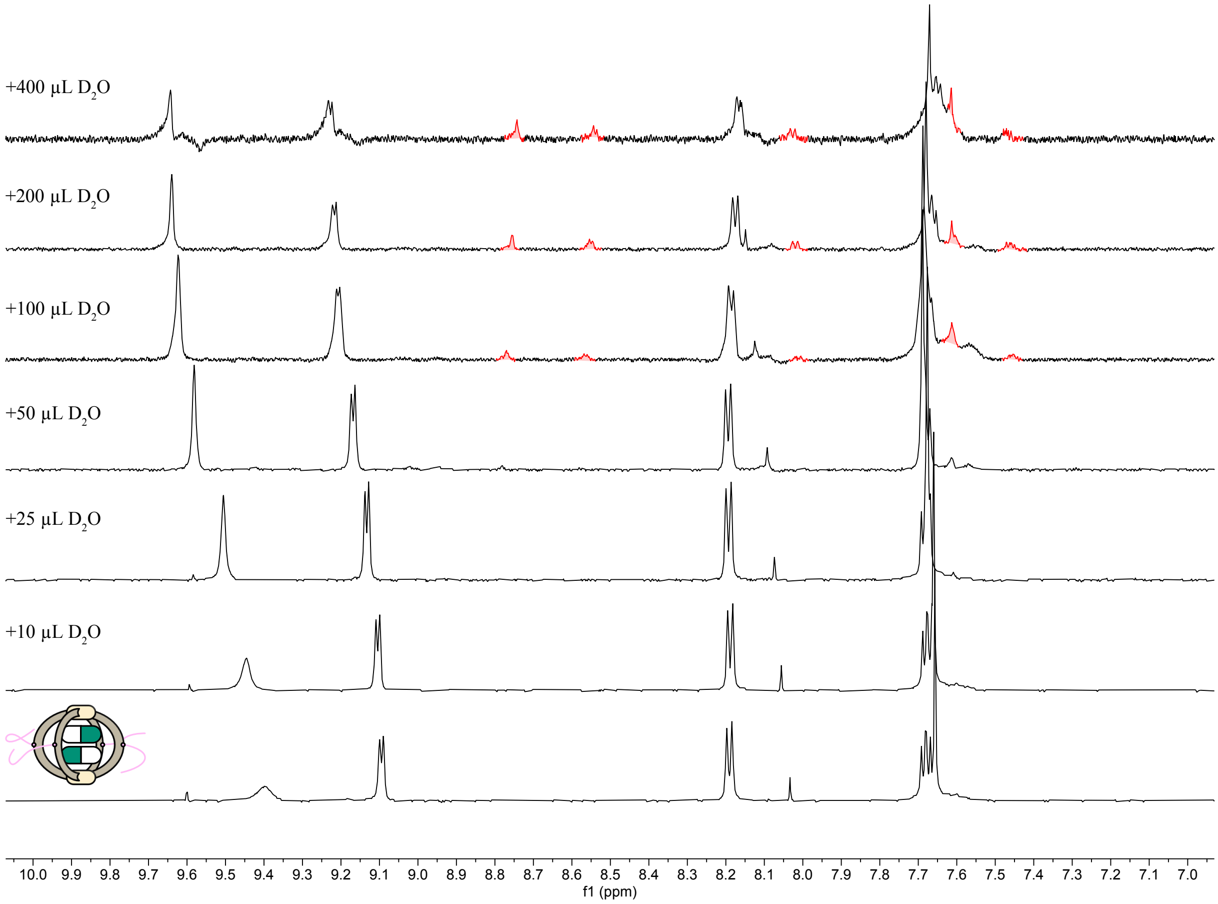

Figure S14: Enlarged view of stacked ^{1}H NMR spectra (600 MHz, CD_3CN, 298 K) showing the stepwise addition of D_2O to a 1.00 mM solution of **PolyMOC2⊃(cisplatin)₂** in CD_3CN. The bottom spectrum corresponds to the initial **PolyMOC2⊃(cisplatin)₂** complex before the addition of D_2O. The signals marked in red correspond to the free ligand (**PolyL2**).

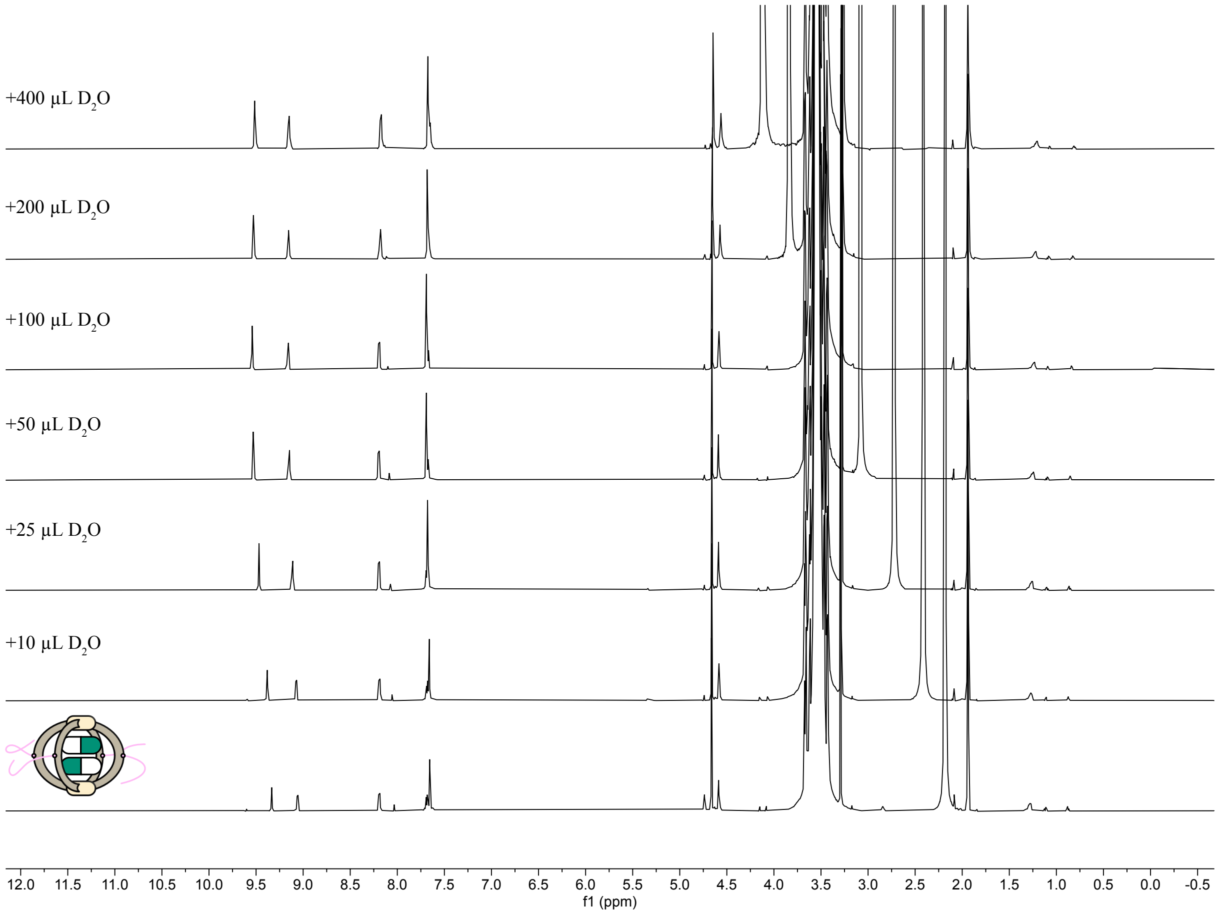

Figure S15: Stacked ^{1}H NMR spectra (600 MHz, CD_3CN, 298 K) showing the stepwise addition of D_2O to a 1.00 mM solution of **PolyMOC2** in CD_3CN. The bottom spectrum corresponds to **PolyMOC2** before the addition of D_2O.

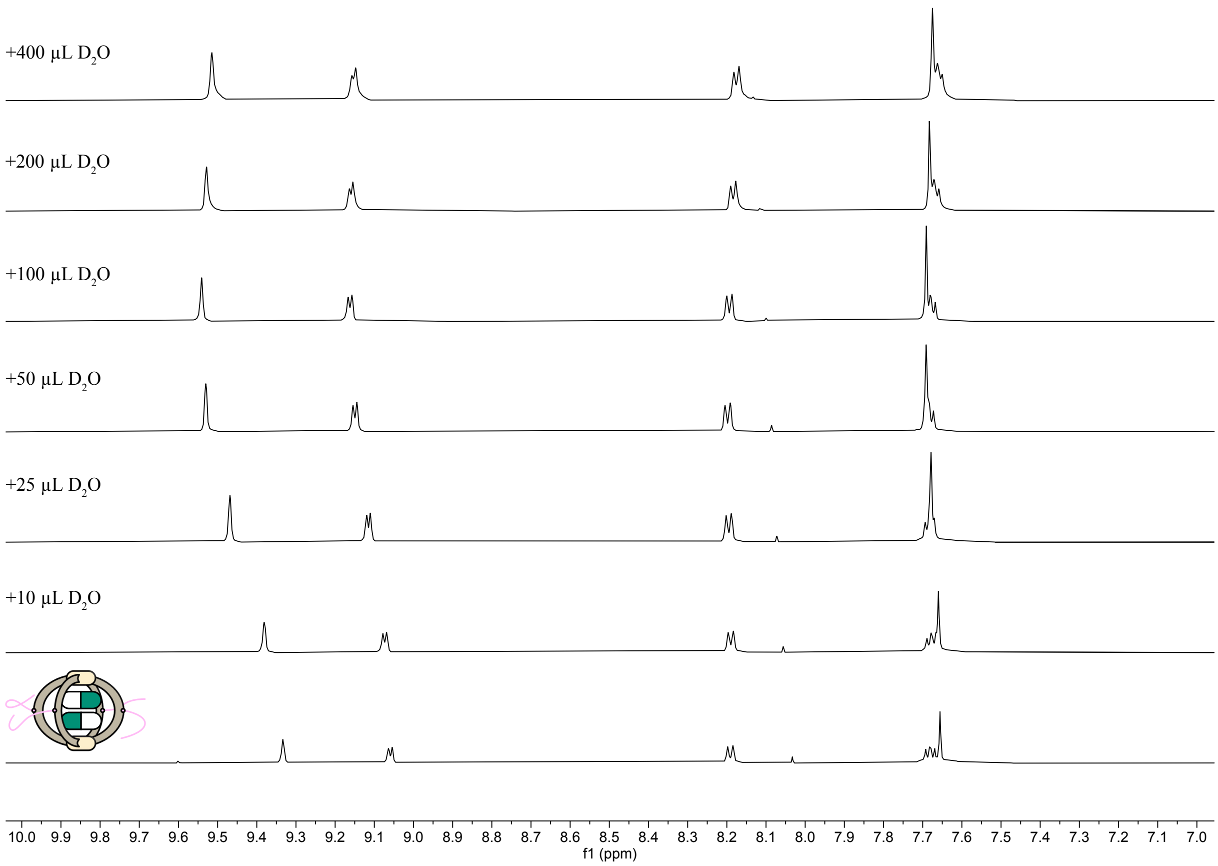

Figure S16: Enlarged view of stacked ^{1}H NMR spectra (600 MHz, CD_3CN, 298 K) showing the stepwise addition of D_2O to a 1.00 mM solution of **PolyMOC2** in CD_3CN. The bottom spectrum corresponds to **PolyMOC2** before the addition of D_2O.

PolyMOC3⊃(cisplatin)$_2$

Attempts to encapsulate cisplatin in **PolyMOC3** in H_2O or D_2O were also made; however, they were unsuccessful. To further investigate the behavior of the complex in aqueous media, a 1.00 mM solution of **PolyMOC3⊃(cisplatin)$_2$** or **PolyMOC3** (0.60 mL CD_3CN) was titrated stepwise with D_2O in the following amounts: 10 µL, 25 µL, 50 µL, 100 µL, 200 µL, 400 µL, 800 µL, and 1200 µL. In both experiments, a similar shifting of the signals was observed. The signal for the proton (a) pointing into the cage, which forms a hydrogen bond with one of the chlorine atoms of cisplatin, sharpens noticeably upon the addition of just 100 µL. Simultaneously, signals corresponding to the free ligand emerge, indicating a partial opening of the cage. These observations strongly suggest the release of cisplatin from the cavity.

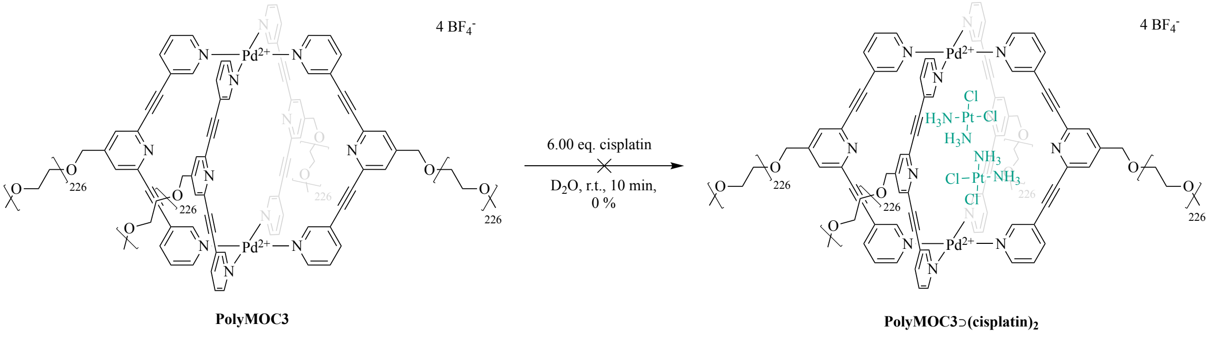

PolyMOC3 (20.3 mg, 0.49 µmol, 1.00 eq.) was dissolved in 0.25 mL of D_2O and cisplatin (0.87 mg, 2.92 µmol, 6.00 eq.) was added. The resulting yellow suspension was stirred vigorously at room temperature for ten minutes, followed by the removal of residual cisplatin *via* filtration through a syringe filter (0.70 µm) into an NMR tube. ^{1}H NMR analysis revealed the unsuccessful encapsulation of cisplatin.

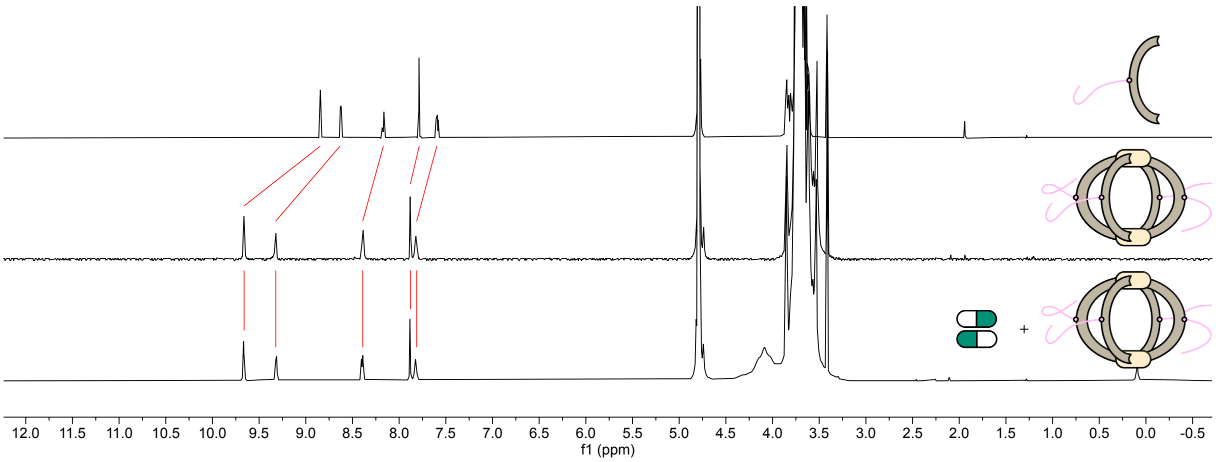

Figure S17: Stacked ^{1}H NMR spectra (600 MHz, D_2O, 298 K) spectra of **PolyL3** (top), **PolyMOC3** (middle), and **PolyMOC3** + 6.00 eq. **cisplatin** (bottom).

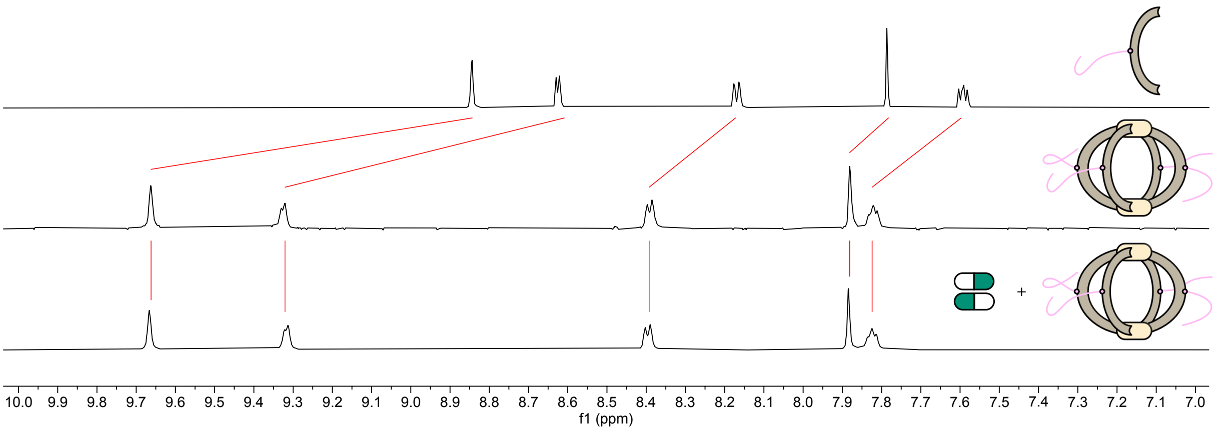

Figure S18: Enlarged view of stacked ^{1}H NMR spectra (600 MHz, D_2O, 298 K) spectra of **PolyL3** (top), **PolyMOC3** (middle), and **PolyMOC3** + 6.00 eq. **cisplatin** (bottom).

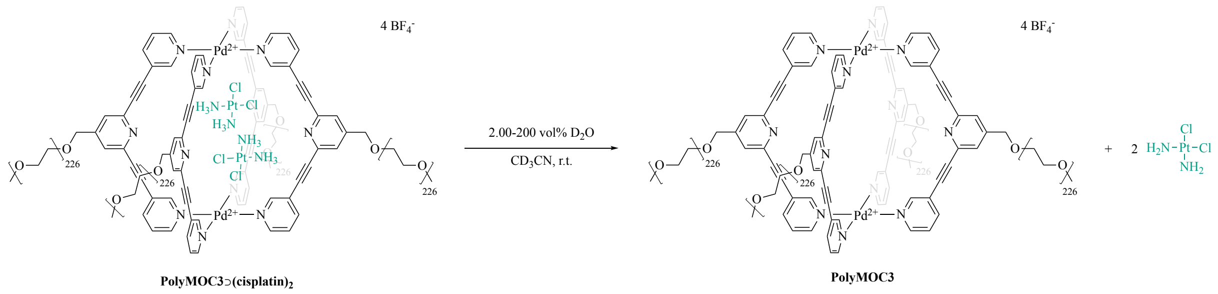

A 1.00 mM solution of **PolyMOC3⊃(cisplatin)₂** (0.60 mL CD_3CN) was titrated stepwise with D_2O in the following amounts: 10 µL, 25 µL, 50 µL, 100 µL, 200 µL, 400 µL, 800 µL, and 1200 µL. The reaction was monitored by ^1H NMR analysis.

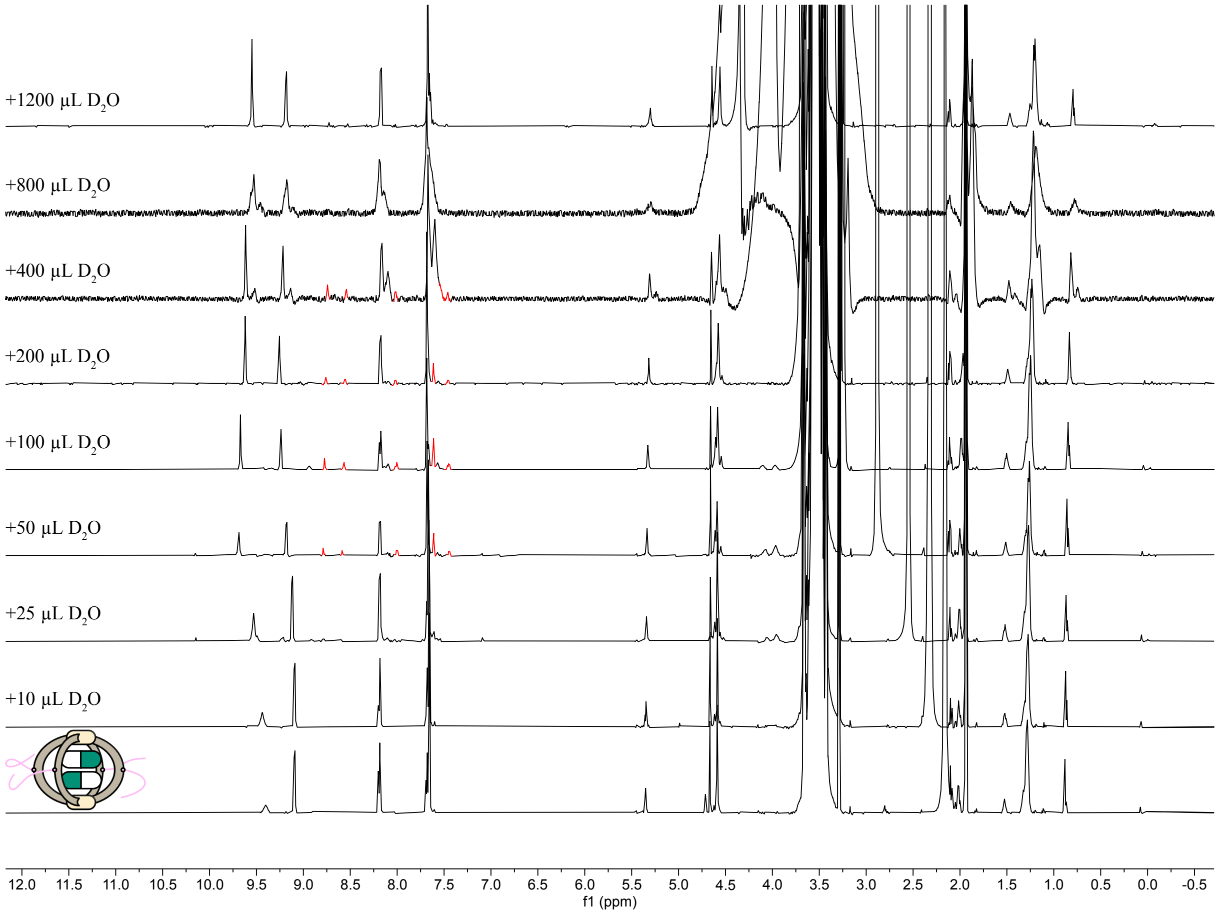

Figure S19: Stacked ^1H NMR spectra (600 MHz, CD_3CN, 298 K) showing the stepwise addition of D_2O to a 1.00 mM solution of **PolyMOC3⊃(cisplatin)₂** in CD_3CN. The bottom spectrum corresponds to the initial **PolyMOC3⊃(cisplatin)₂** complex before the addition of D_2O. The signals marked in red correspond to the free ligand (**PolyL3**).

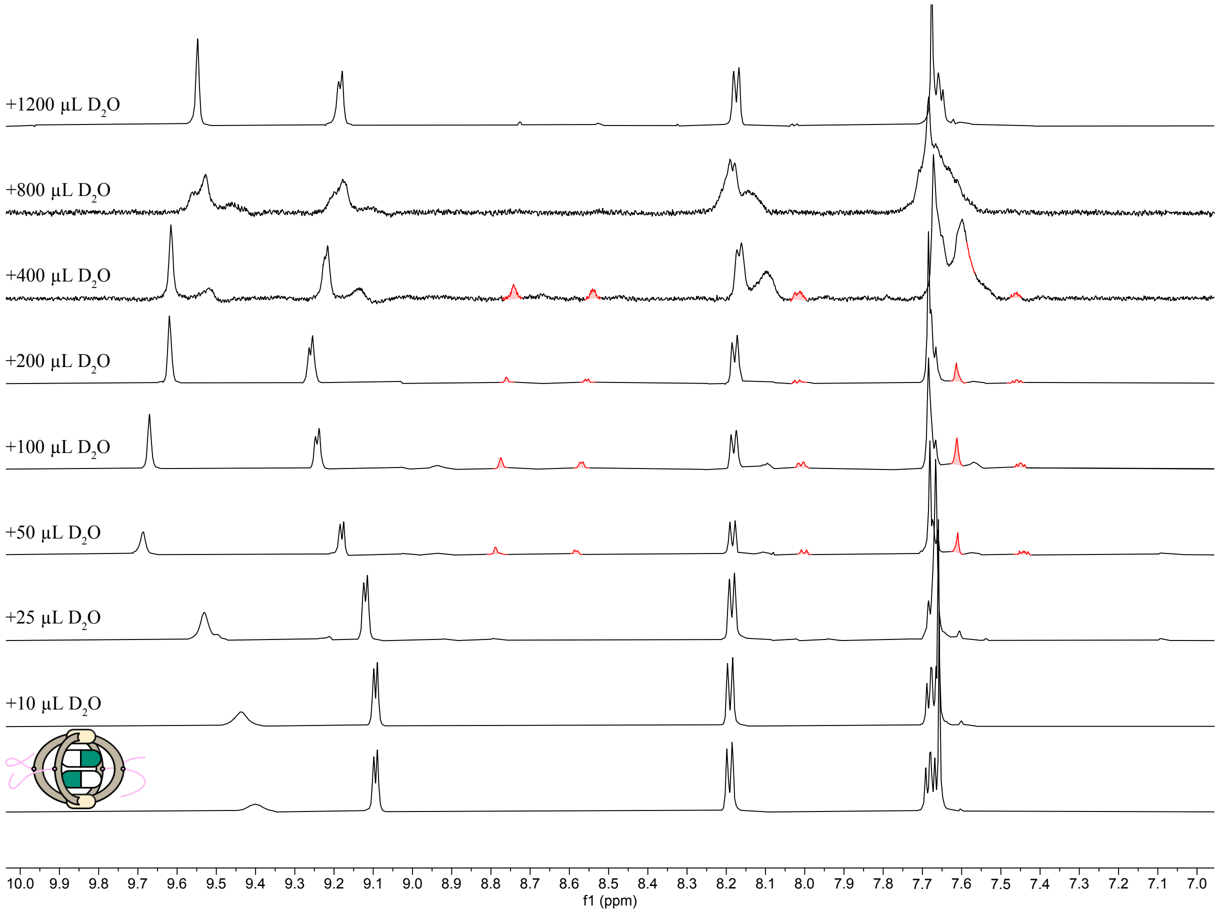

Figure S20: Enlarged view of stacked ^{1}H NMR spectra (600 MHz, CD_3CN, 298 K) showing the stepwise addition of D_2O to a 1.00 mM solution of **PolyMOC3⊃(cisplatin)₂** in CD_3CN. The bottom spectrum corresponds to the initial **PolyMOC3⊃(cisplatin)₂** complex before the addition of D_2O. The signals marked in red correspond to the free ligand (**PolyL3**).

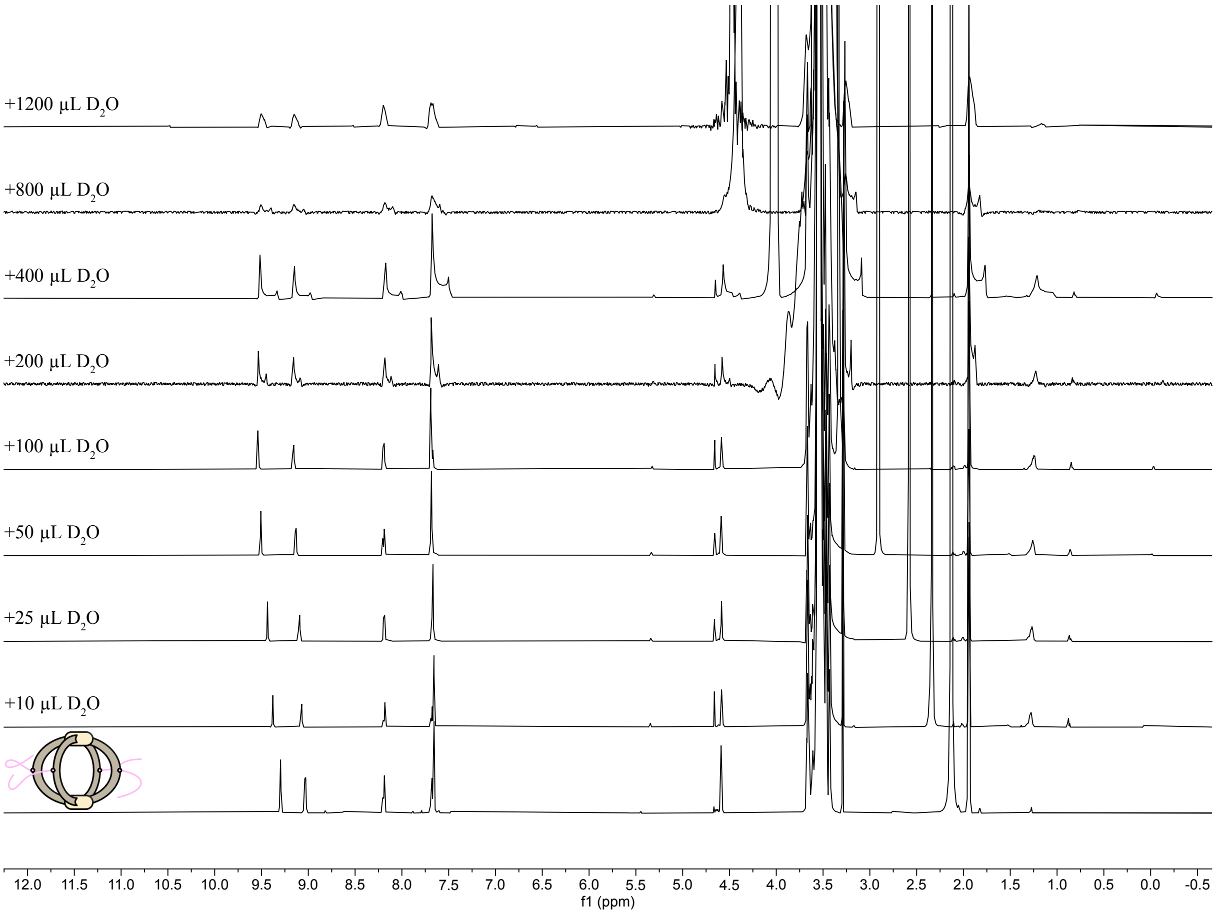

Figure S21: Stacked ^{1}H NMR spectra (600 MHz, CD_3CN, 298 K) showing the stepwise addition of D_2O to a 1.00 mM solution of **PolyMOC3** in CD_3CN. The bottom spectrum corresponds to **PolyMOC3** before the addition of D_2O.

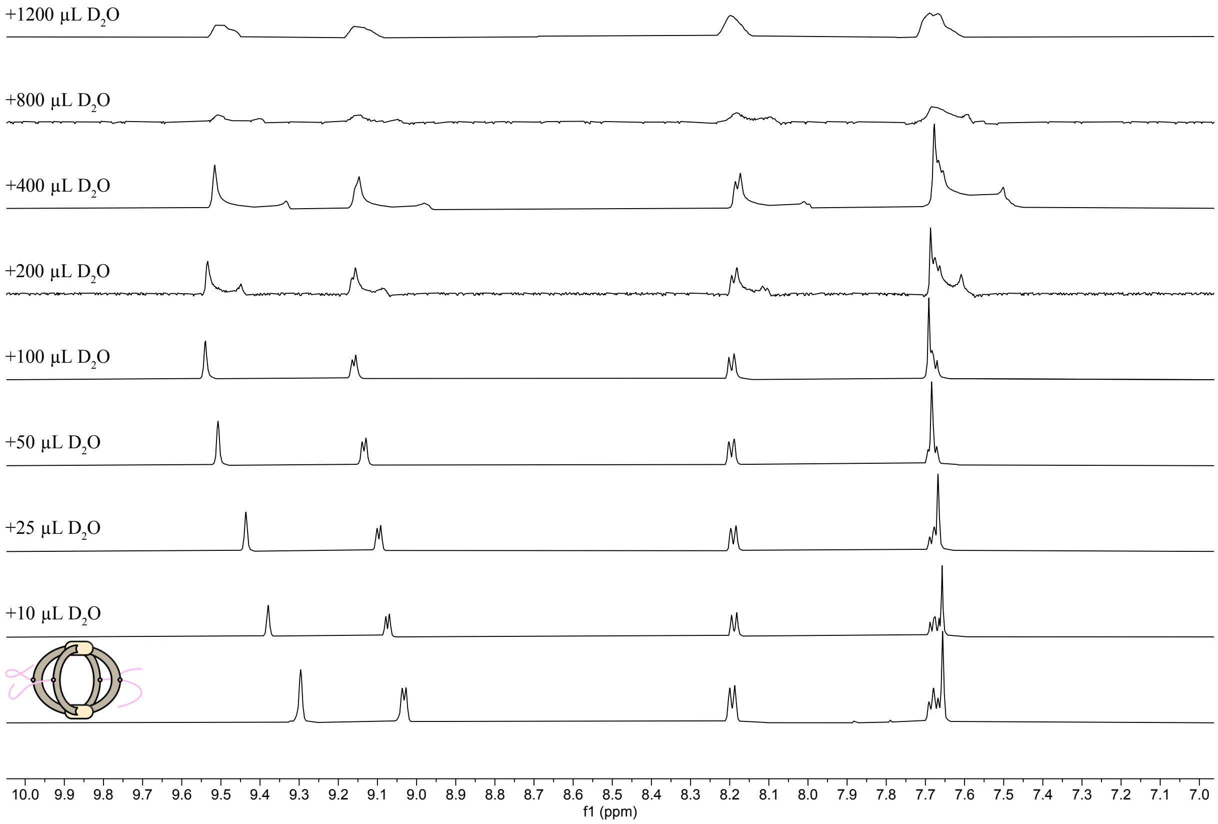

Figure S22: Enlarged view of stacked ^{1}H NMR spectra (600 MHz, CD_3CN, 298 K) showing the stepwise addition of D_2O to a 1.00 mM solution of **PolyMOC3** in CD_3CN. The bottom spectrum corresponds to **PolyMOC3** before the addition of D_2O.

IV. Investigating the role of polymer chains and cage stability under sonication conditions

To rule out dilution-induced dissociation or disassembly of both **PolyMOC** and **PolyMOC⊃(cisplatin)₂** complexes, ^{1}H NMR spectra were measured in CD_3CN and D_2O, at the highest dilution used in subsequent studies, unequivocally demonstrating their structural integrity. To demonstrate that the polymer chains are necessary for activating the cages, **MOC3** and **MOC3⊃(cisplatin)₂** were sonicated to show that the ultrasonic conditions have no effect on the cage or the inclusion complex. Additionally, the polymer ligand **PolyL3** was subjected to ultrasound to investigate the potential for backbone splitting.

PolyMOC and PolyMOC⊃(cisplatin)₂ under high dilution conditions

To unequivocally ascertain the stability of the PolyMOCs, we acquired ^{1}H NMR spectra at elevated concentrations and subsequently diluted the samples with deuterated solvent (CD_3CN or D_2O) to achieve final concentrations of 2.50 mg mL^{-1}, followed by a re-evaluation of the ^{1}H NMR spectrum and a comparison with the spectrum of the free ligand (Figure S23-26).

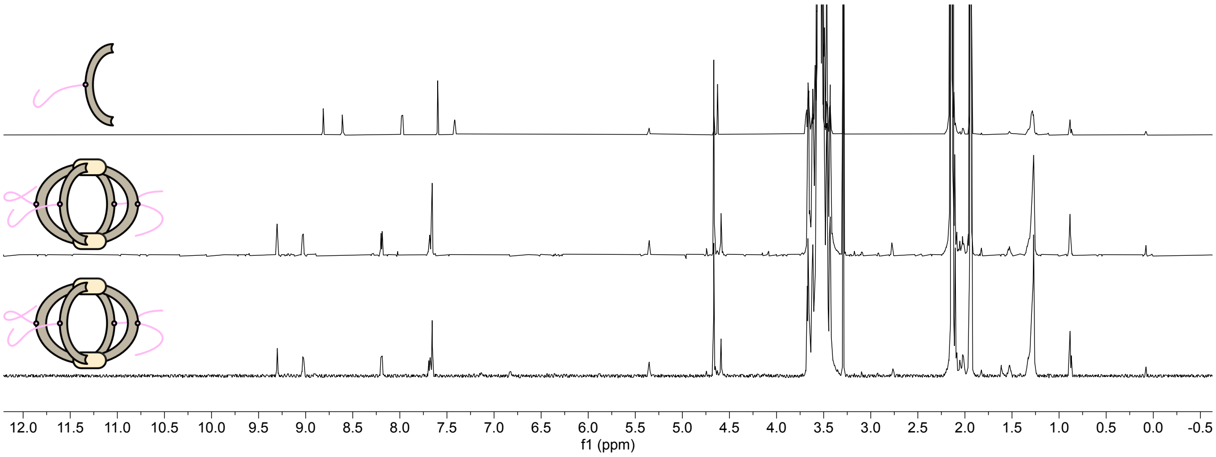

Figure S23: Stacked ^{1}H NMR spectra (600 MHz, CD_3CN, 298 K) of **PolyL2** (top), **PolyMOC2** (middle), and **PolyMOC2** at a concentration of 2.50 mg mL^{-1} (bottom).

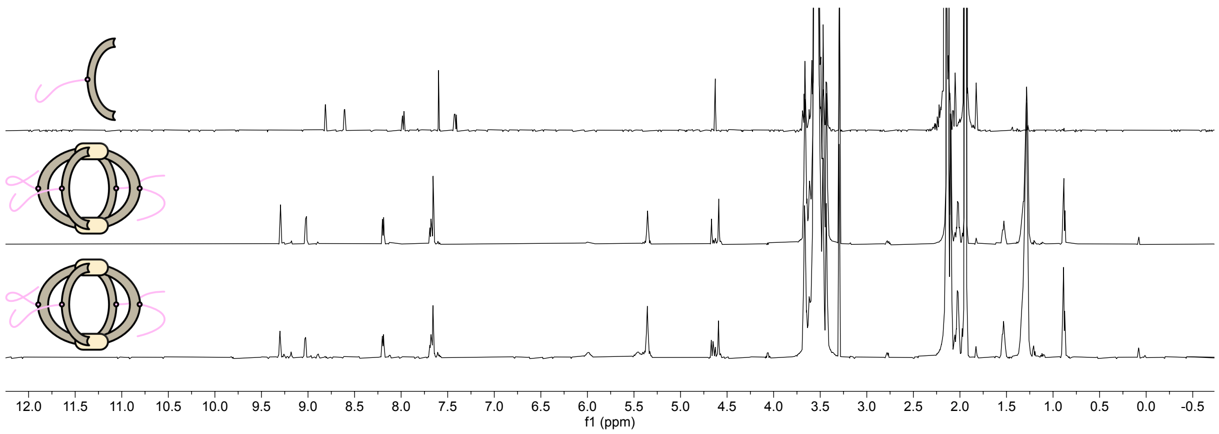

Figure S24: Stacked ^{1}H NMR spectra (600 MHz, CD_3CN, 298 K) of **PolyL3** (top), **PolyMOC3** (middle), and **PolyMOC3** at a concentration of 2.50 mg mL^{-1} (bottom).

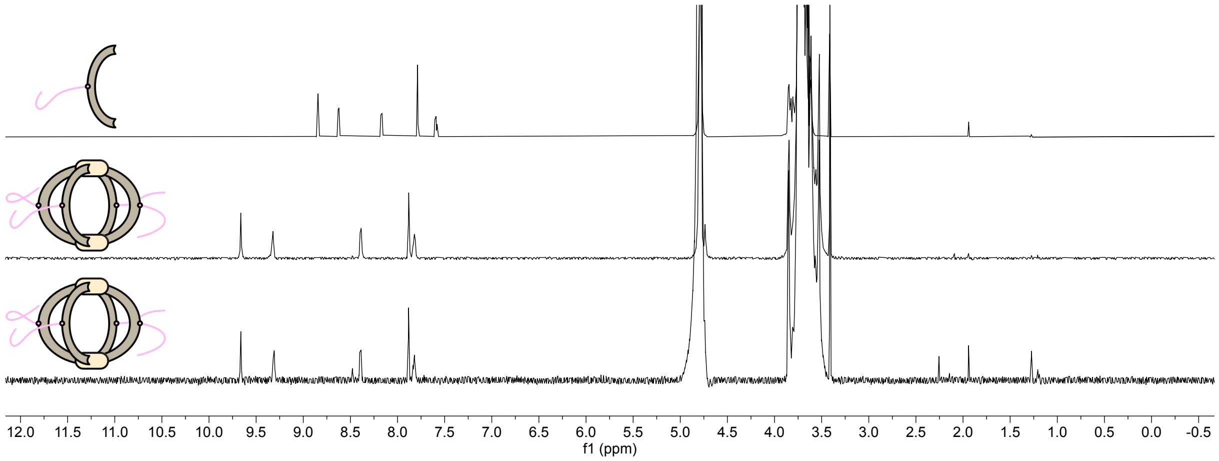

Figure S25: Stacked ^{1}H NMR spectra (600 MHz, D_2O, 298 K) of **PolyL3** (top), **PolyMOC3** (middle), and **PolyMOC3** at a concentration of 2.50 mg mL^{-1} (bottom).

To unequivocally ascertain the stability of the **PolyMOC⊃(cisplatin)₂** complexes, cisplatin was encapsulated in the corresponding PolyMOCs at a concentration of 2 mM (as described in Chapter II), subsequently diluted to 2.50 mg mL^{-1}, and analysed by ^{1}H NMR analysis.

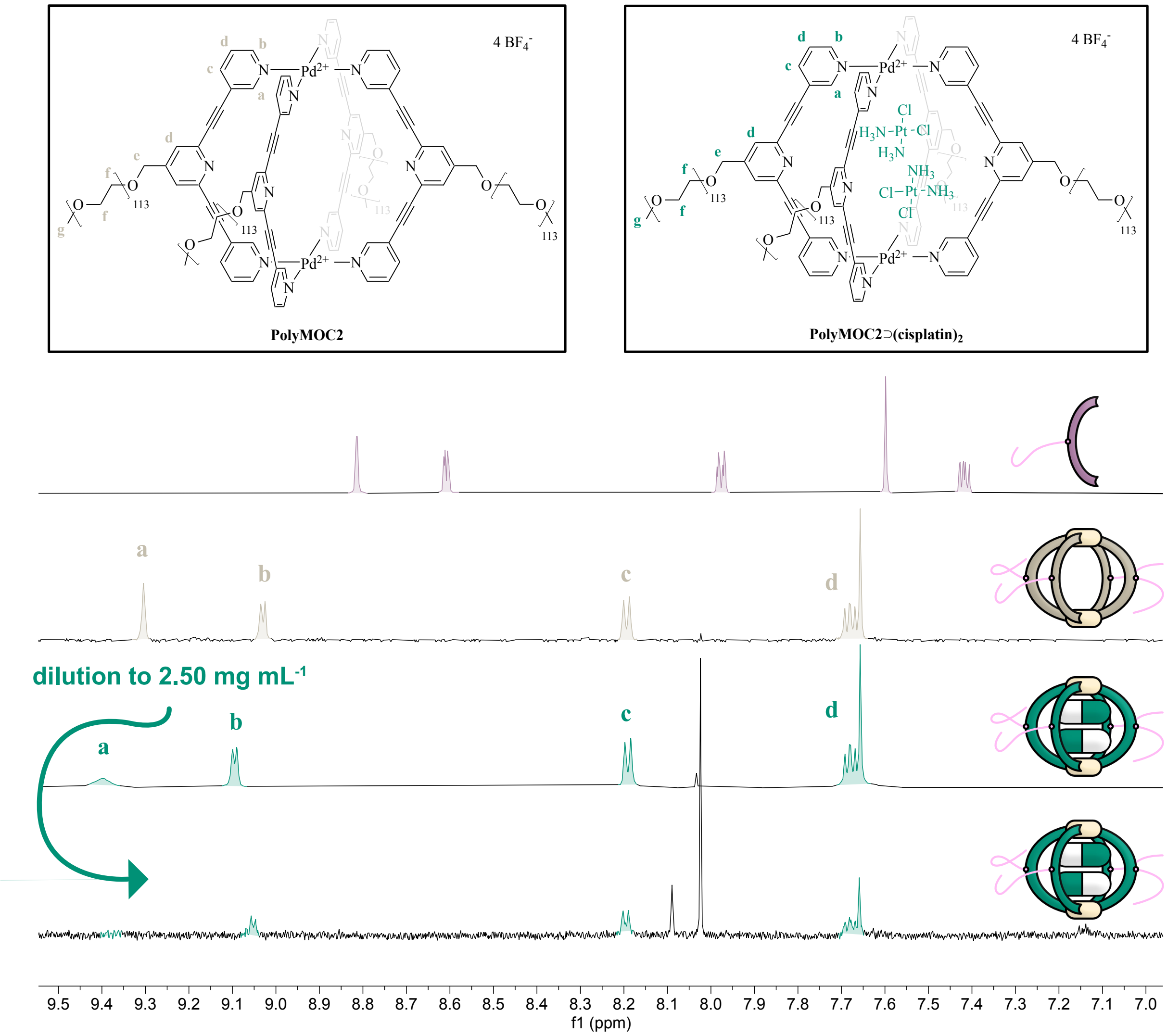

Figure S26: Stacked ^{1}H NMR spectra (600 MHz, CD_3CN, 298 K) of **PolyL2** (top), **PolyMOC2** (middle, top), **PolyMOC2⊃(cisplatin)₂** (middle, bottom), and **PolyMOC2⊃(cisplatin)₂** at a concentration of 2.50 mg mL^{-1} (bottom).

Sonication of MOC3 and MOC3⊃(cisplatin)$_2$

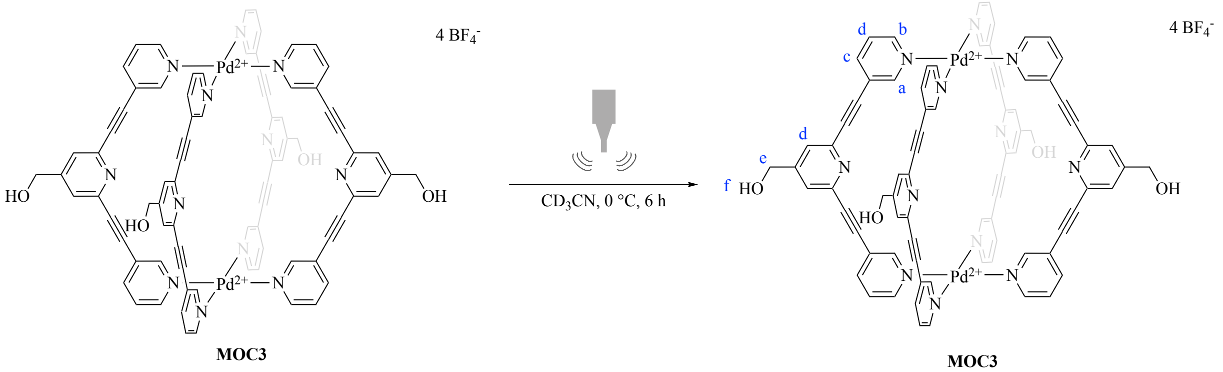

The **MOC3** solution in CD$_3$CN was transferred into an argon-purged Suslick vessel and diluted with dry acetonitrile to obtain the final concentration of 1.00 mg mL^{-1}. The sonication mixture was then cooled to 0 °C in an ice-water bath and degassed with argon for ten minutes before sonication. Sonication was performed for six hours (three hours effective time) using a 1 s on / 1 s off pulse sequence at 30 % amplitude, while the reaction mixture was continuously bubbled with argon. The resulting reaction mixture was freeze-dried using liquid nitrogen, and the solvent was pumped off before a ^{1}H NMR was recorded.

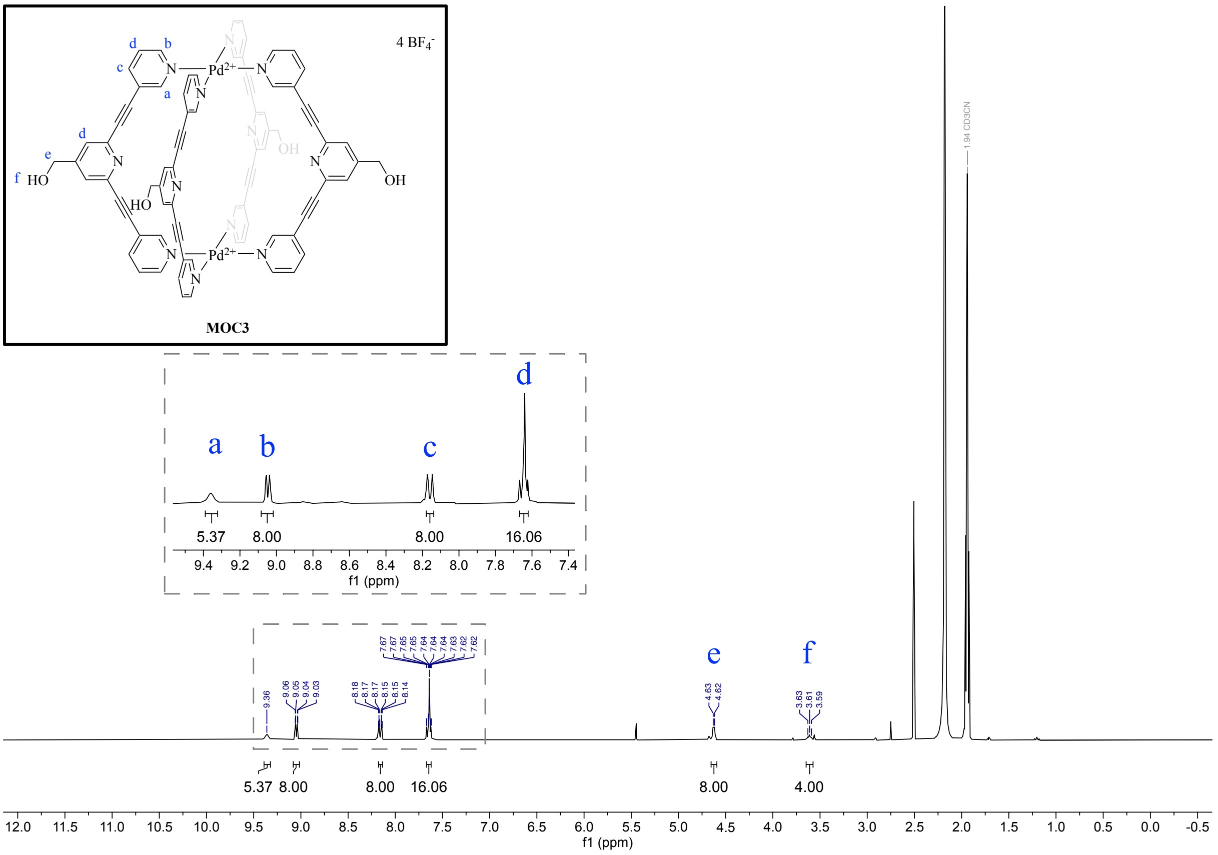

Figure S27: ^{1}H NMR spectrum (300 MHz, CD$_3$CN, 298 K) of **MOC3** after six hours of sonication (Ar).

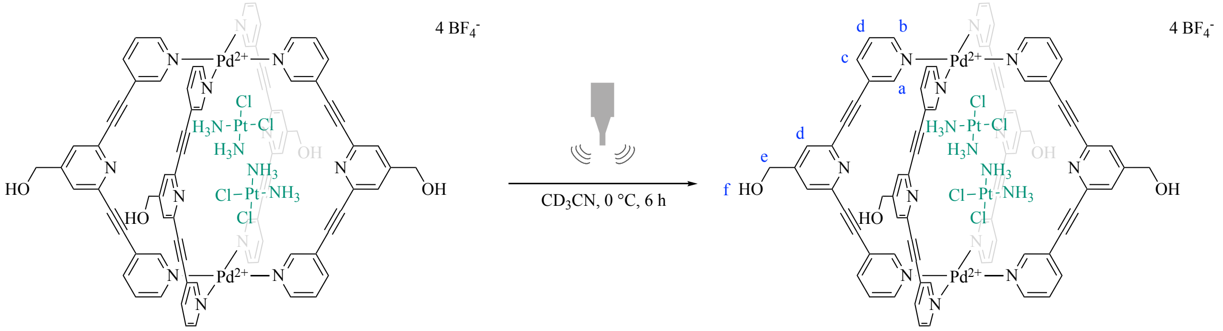

MOC3⊃(cisplatin)$_2$ **MOC3⊃(cisplatin)$_2$**

The **MOC3⊃(cisplatin)$_2$** solution in CD_3CN was transferred into an argon-purged Suslick vessel and diluted with dry acetonitrile to obtain the final concentration of 1.00 mg mL^{-1}. The sonication mixture was then cooled to 0 °C in an ice-water bath and degassed with argon for ten minutes before sonication. Sonication was performed for six hours (three hours effective time) using a 1 s on / 1 s off pulse sequence at 30 % amplitude, while the reaction mixture was continuously bubbled with argon. The resulting reaction mixture was freeze-dried using liquid nitrogen, and the solvent was pumped off before a ^{1}H NMR was recorded.

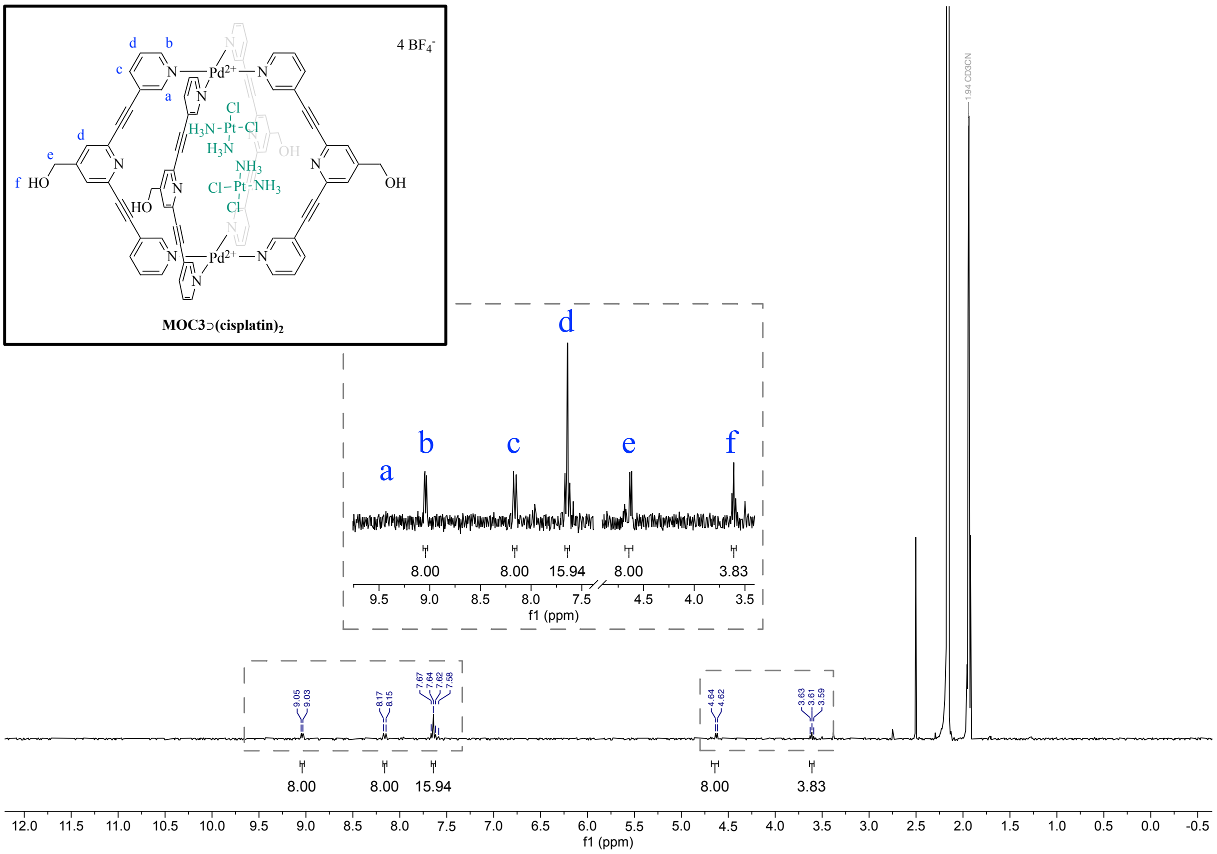

Figure S28: ^{1}H NMR spectrum (300 MHz, CD_3CN, 298 K) of **MOC3⊃(cisplatin)$_2$** after six hours of sonication (Ar).

Sonication of polymeric ligand PolyL3

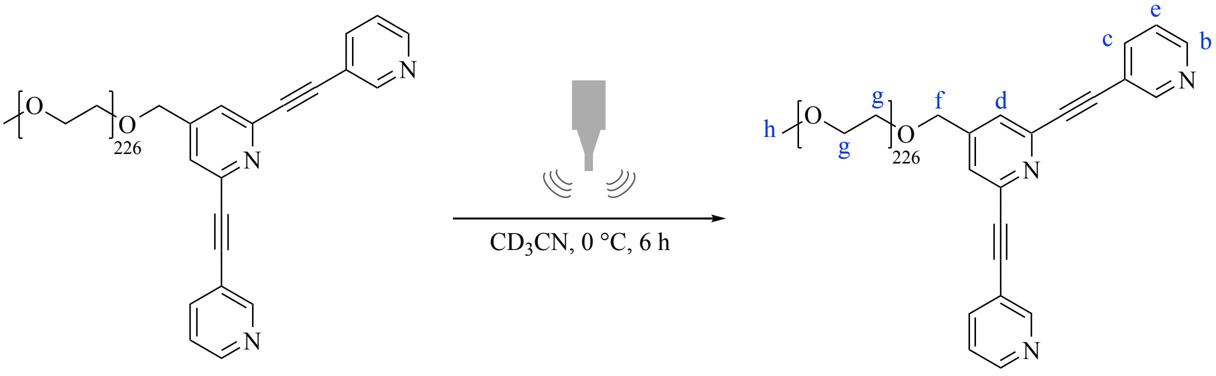

PolyL3 **PolyL3**

A 1.00 mg mL^{-1} solution of **PolyL3** in dry acetonitrile was placed in an argon-purged Suslick vessel. The sonication mixture was degassed for ten minutes with argon before sonication for six hours (three hours effective time) using a 1 s on / 1 s off pulse sequence at 30 % amplitude, while the reaction mixture was continuously bubbled with argon. The resulting reaction mixture was freeze-dried using liquid nitrogen, and the solvent was pumped off before a ^1H NMR was recorded.

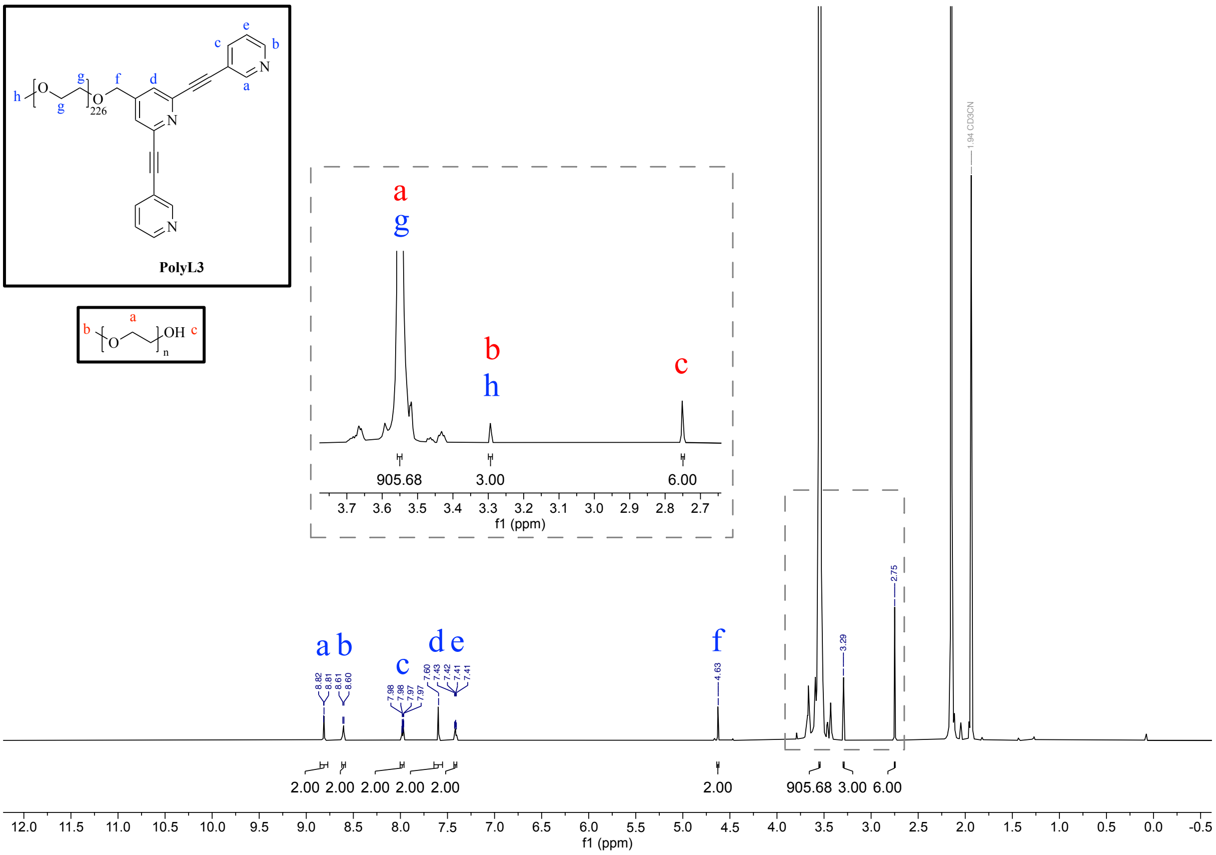

Figure S29: ^1H NMR spectrum (600 MHz, CD_3CN, 298 K) of **PolyL3** after six hours of sonication (Ar).

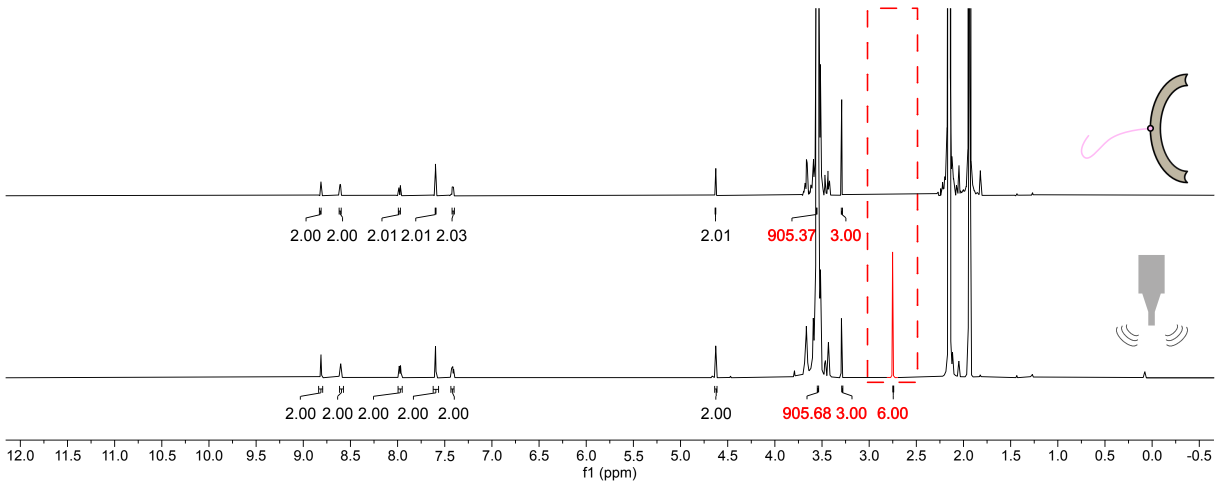

Figure S30: Stacked ^{1}H NMR spectra (600 MHz, CD_3CN, 298 K) of **PolyL3** (top) and **PolyL3** after six hours of sonication (Ar)(bottom). The unchanged ratio of the aromatic protons relative to the capping methyl group, along with the identical integral for the PEG chain, but the additionally highlighted red signal for free OH groups on PEG, clearly indicates that backbone splitting can occur under given conditions.

V. Acoustic properties of the applied ultrasound field

All sonication experiments were performed with a Vibra cell VCX 750 sonicator (750 W) with a frequency of 20 kHz, an amplitude A of 30 %, a full wave probe (13 mm), and a pulse sequence of 1 s on and 1 s off (p = 50 %). The acoustic power $P_{acoustic}$ was first estimated without accounting for energy losses (Eq. S1). This number was then used to calculate the acoustic intensity $I_{acoustic}$ (Eq. S3), which made it possible to calculate the pressure amplitude p_0 using the density of acetonitrile (782 kg m^3at 20 °C and ambient pressure)[11] and water (999 kg m^3at 0 °C and ambient pressure)[12], as well as the speed of sound in acetonitrile (1391.45 m s^{-1} (at 0.003 MPa and -3 °C))[13] and water (1406.04 m s^{-1} (at 0.09 MPa and 0 °C))[14] (Eq. S5). The corresponding displacement amplitude ε_0 was calculated from the pressure amplitude (Eq. S6). Lastly, the wavelength λ of the acoustic wave was calculated using the speed of sound in acetonitrile and water (Eq. S7).

$$P_{acoustic} = P_{electric} * A * p * losses \quad \text{(S1)}$$

$$P_{acoustic} = P_{electric}(750\ W) * A(0.3)^2 * p(0.5) * losses = \mathbf{33.8\ W} \quad \text{(S2)}$$

$$I_{acoustic} = \frac{P_{acoustic}}{A_{tip}} \quad \text{(S3)}$$

$$I_{acoustic} = \frac{P_{acoustic}\,(33.8\ W)}{A_{tip}\,(1.33\ cm^2)} = \mathbf{25.4\ \frac{W}{cm^2}} \quad \text{(S4)}$$

$$p_0 = \sqrt{2\rho_{solvent} c_{in\ solvent\ at\ used\ T} I_{acoustic}} \quad \text{(S5)}$$

$$\varepsilon_0 = \frac{p_0}{\rho_{solvent} c_{in\ solvent\ at\ used\ T} 2\pi f} \quad \text{(S6)}$$

$$\lambda = \frac{c}{f} \quad \text{(S7)}$$

Acetonitrile:

$$p_0 = \sqrt{2\rho_{solvent}(782\ kg\ m^3)\ c_{in\ solvent\ at\ used\ T}(1391.45\ \frac{m}{s})\ I_{acoustic}}(254000\ \frac{W}{m^2}) = \mathbf{7.43\ bar} \quad \text{(S8)}$$

$$\varepsilon_0 = \frac{p_0}{\rho c 2\pi f} = \frac{0.743 * 10^6 \frac{kg}{ms^2}}{782 \frac{kg}{m^3} * 1391.45 \frac{m}{s} * 2\pi * 20000 \frac{1}{s}} = \mathbf{5.43\ \mu m} \quad \text{(S9)}$$

$$\lambda = \frac{c}{f} = \frac{1391.45 \frac{m}{s}}{20000 \frac{1}{s}} = \mathbf{6.96\ cm} \quad \text{(S10)}$$

Water:

$$p_0 = \sqrt{2\rho_{solvent}(999\ kg\ m^3)\ c_{in\ solvent\ at\ used\ T}(1406.04\ \frac{m}{s})\ I_{acoustic}(254000\ \frac{W}{m^2})} = \mathbf{8.45\ bar} \quad \text{(S10)}$$

$$\varepsilon_0 = \frac{p_0}{\rho c 2\pi f} = \frac{0.845 * 10^6 \frac{kg}{ms^2}}{999 \frac{kg}{m^3} * 1406.04 \frac{m}{s} * 2\pi * 20000 \frac{1}{s}} = \mathbf{4.79\ \mu m} \quad \text{(S11)}$$

$$\lambda = \frac{c}{f} = \frac{1406.04 \frac{m}{s}}{20000 \frac{1}{s}} = \mathbf{7.03\ cm} \quad \text{(S12)}$$

VI. Ultrasound-triggered disassembly of PolyMOC

To explore the effect of different ultrasonic conditions on the disassembly of **PolyMOC1-3**, a series of experiments were conducted, systematically varying the sonication concentration, saturation gas, and polymer chain length. The goal was to elucidate the mechanism underlying the disassembly process of **PolyMOC1-3** in both organic and aqueous media under ultrasound exposure. For this purpose, **PolyMOC1-3** solutions (0.77 mg mL^{-1}, 2.50 mg mL^{-1} or 5.00 mg mL^{-1}) were prepared in dry or deuterated acetonitrile, deuterium oxide, or water. These solutions were first degassed in a Suslick vessel for ten minutes before undergoing sonication for six hours, while the reaction mixture was continuously bubbled with the saturation gas. The disassembly process was closely monitored using ^{1}H NMR spectroscopy.

Table S1: Summary of disassembly experiments and corresponding conditions.

PolyMOC	Solvent	Polymer Chain Length (RU)	Concentration (mg mL^{-1})	Concentration (µM)	Saturation Gas	Reversibility	Result/Discription
1	CD_3CN	22	2.50	412	Nitrogen	no	no activation 100% inatct PolyMOC1
1	CD_3CN	22	0.77	125	Nitrogen	no	no activation 100% inatct PolyMOC1
2	CD_3CN	113	2.50	125	Nitrogen	no	62% PolyMOC2, 9% Pd_2L_3, 29% PolyL2
2	CD_3CN	113	5.00	250	Nitrogen	no	53% PolyMOC2, 47% Pd_2L_3
2	CD_3CN	113	2.50	125	Argon	no	0% PolyMOC2, 100% Pd_2L_2
2	CD_3CN	113	5.00	250	Argon	no	PolyMOC2 traces, mostly Pd_2L_2
3	CD_3CN	226	2.50	62.5	Nitrogen	no	60% PolyMOC2, 22% Pd_2L_3, 18% PolyL2
3	CD_3CN	226	5.00	125	Nitrogen	**yes**	78% PolyMOC2, 22% Pd_2L_3
3	CD_3CN	226	2.50	62.5	Argon	no	0% PolyMOC3, 100% Pd_2L_2
3	CD_3CN	226	5.00	125	Argon	no	PolyMOC3 traces, mostly Pd_2L_2
3	D_2O	226	2.50	62.5	Nitrogen	no	Oxidation, no corresponding signals after 4 hours
3	D_2O	226	2.50	62.5	Argon	no	Oxidation, no corresponding signals after 3 hours

PolyMOC1

Sonication in acetonitrile

Nitrogen as saturation gas (c = 0.77 mg mL^{-1}, 125 µM)

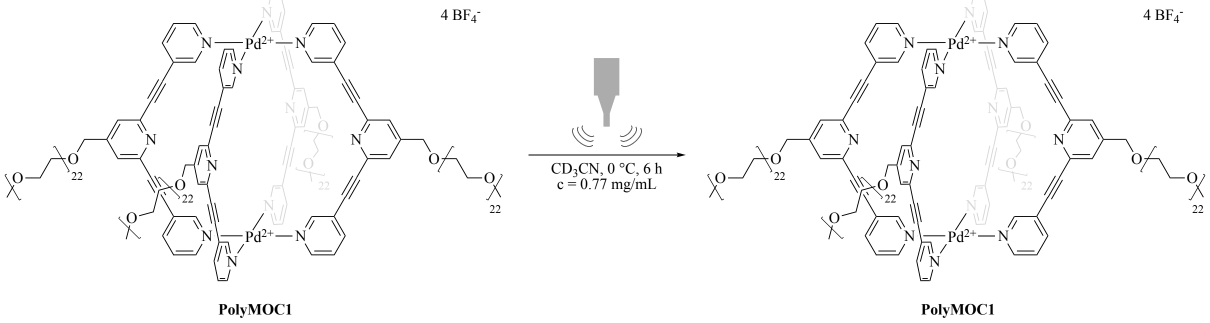

Figure S31: Reaction scheme depicting the sonication-induced disassembly of **PolyMOC1** (0.77 mg mL^{-1}) under nitrogen saturation.

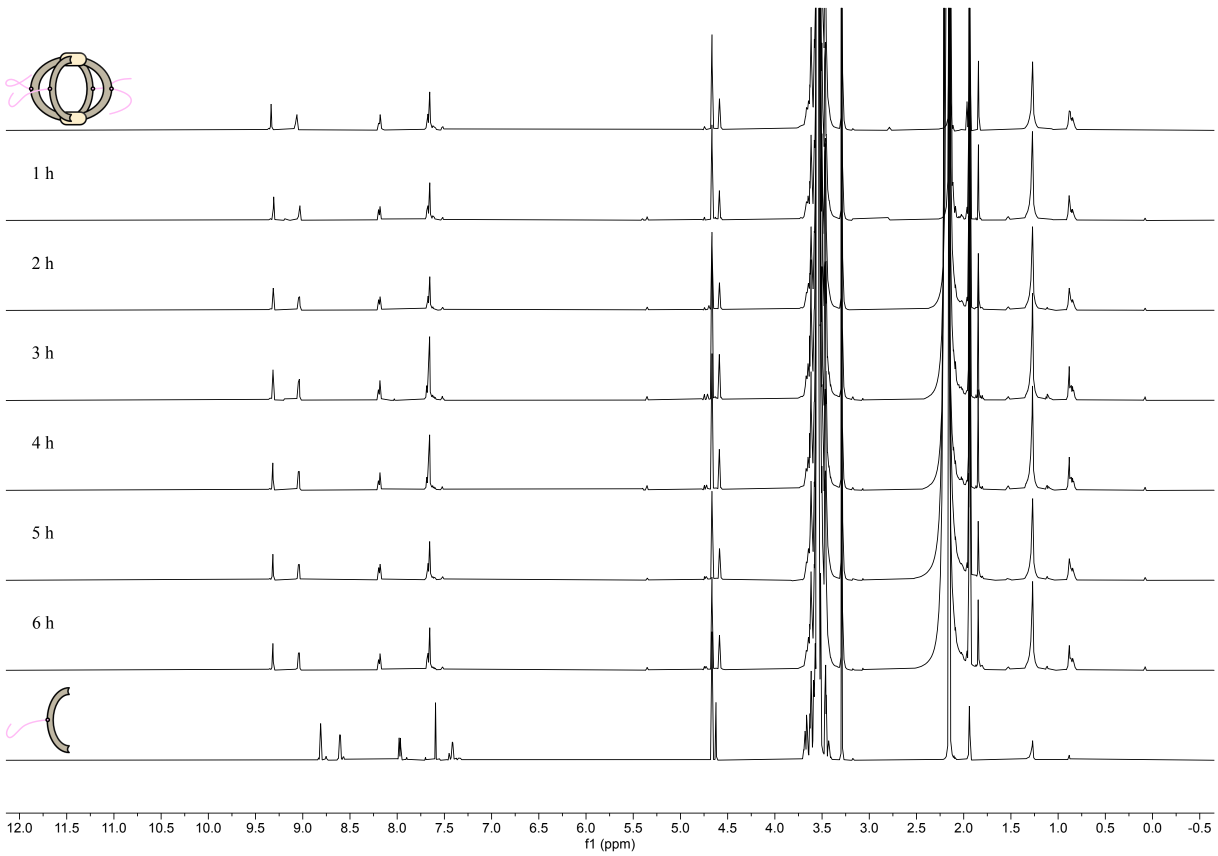

Figure S32: Stacked ^{1}H NMR spectra (600 MHz, CD_3CN, 298 K) showing the disassembly of **PolyMOC1** progressing over time. The spectra correspond to the initial complex (top), six sequential measurements recorded after each hour of sonication (middle), and **PolyL1** (bottom).

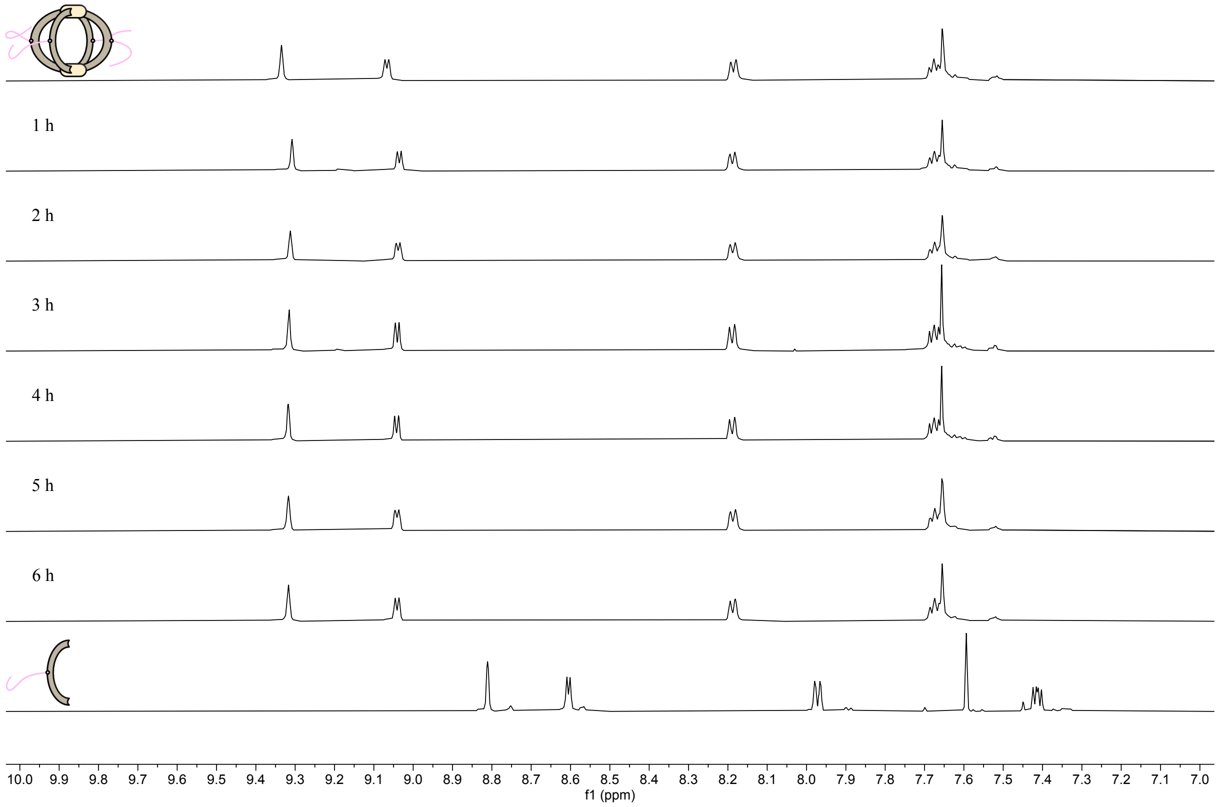

Figure S33: Enlarged view of stacked ^{1}H NMR spectra (600 MHz, CD_3CN, 298 K) showing the disassembly of **PolyMOC1** progressing over time. The spectra correspond to the initial complex (top), six sequential measurements recorded after each hour of sonication (middle), and **PolyL1** (bottom).

Nitrogen as saturation gas (c = 2.50 mg mL^{-1}, 412 µM)

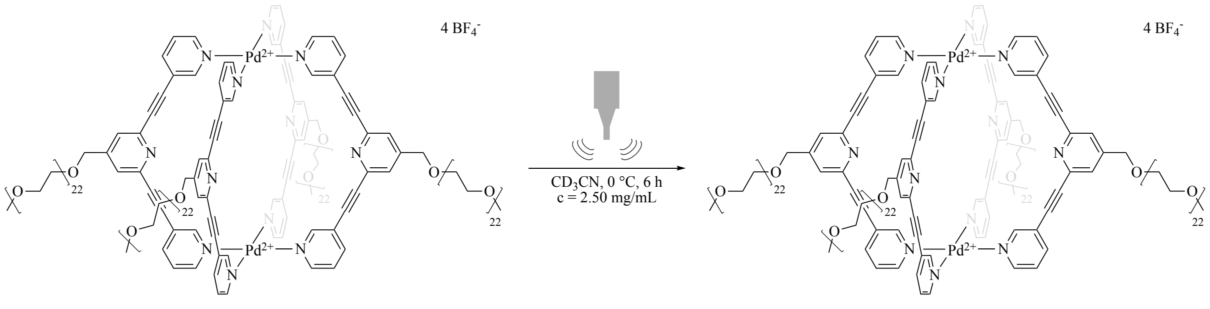

Figure S34: Reaction scheme depicting the sonication-induced disassembly of **PolyMOC1** (0.77 mg mL^{-1}) under nitrogen saturation.

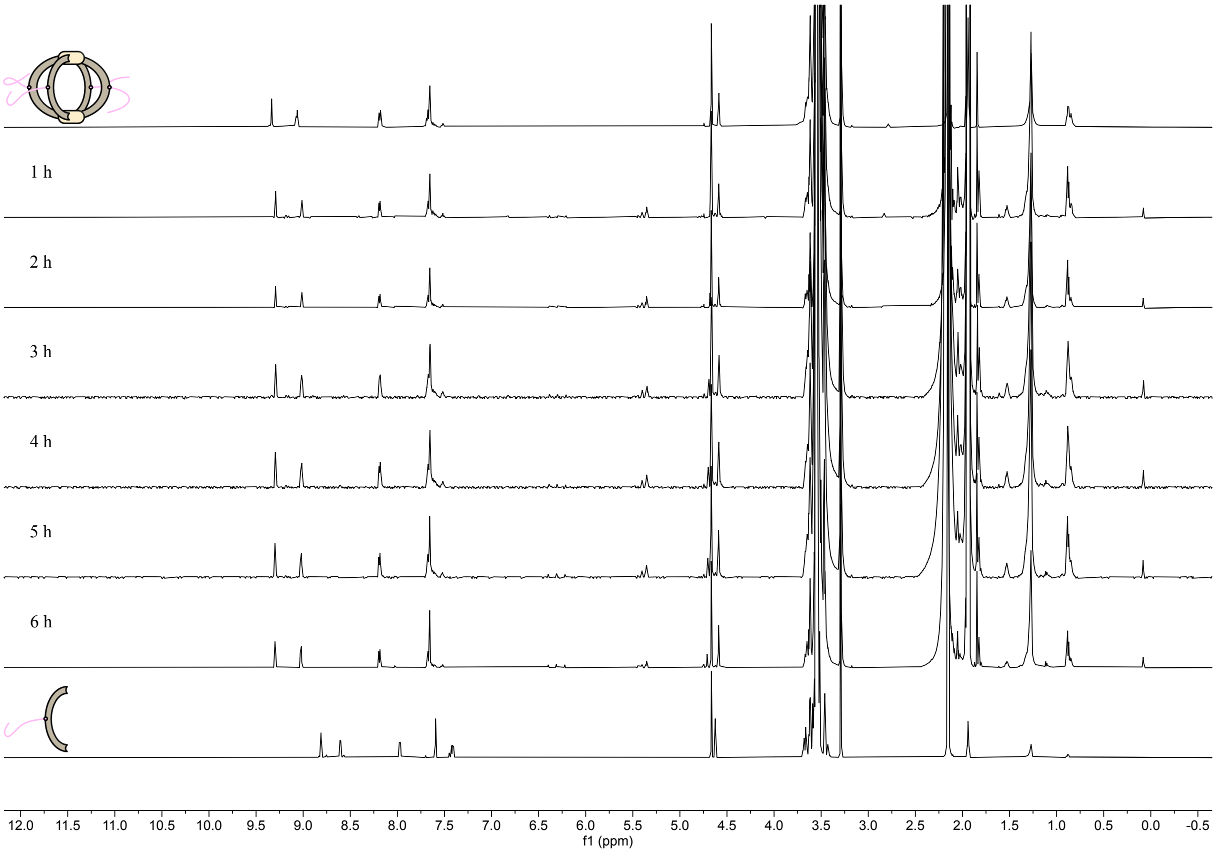

Figure S35: Stacked ^{1}H NMR spectra (600 MHz, CD_3CN, 298 K) showing the disassembly of **PolyMOC1** progressing over time. The spectra correspond to the initial complex (top), six sequential measurements recorded after each hour of sonication (middle), and **PolyL1** (bottom).

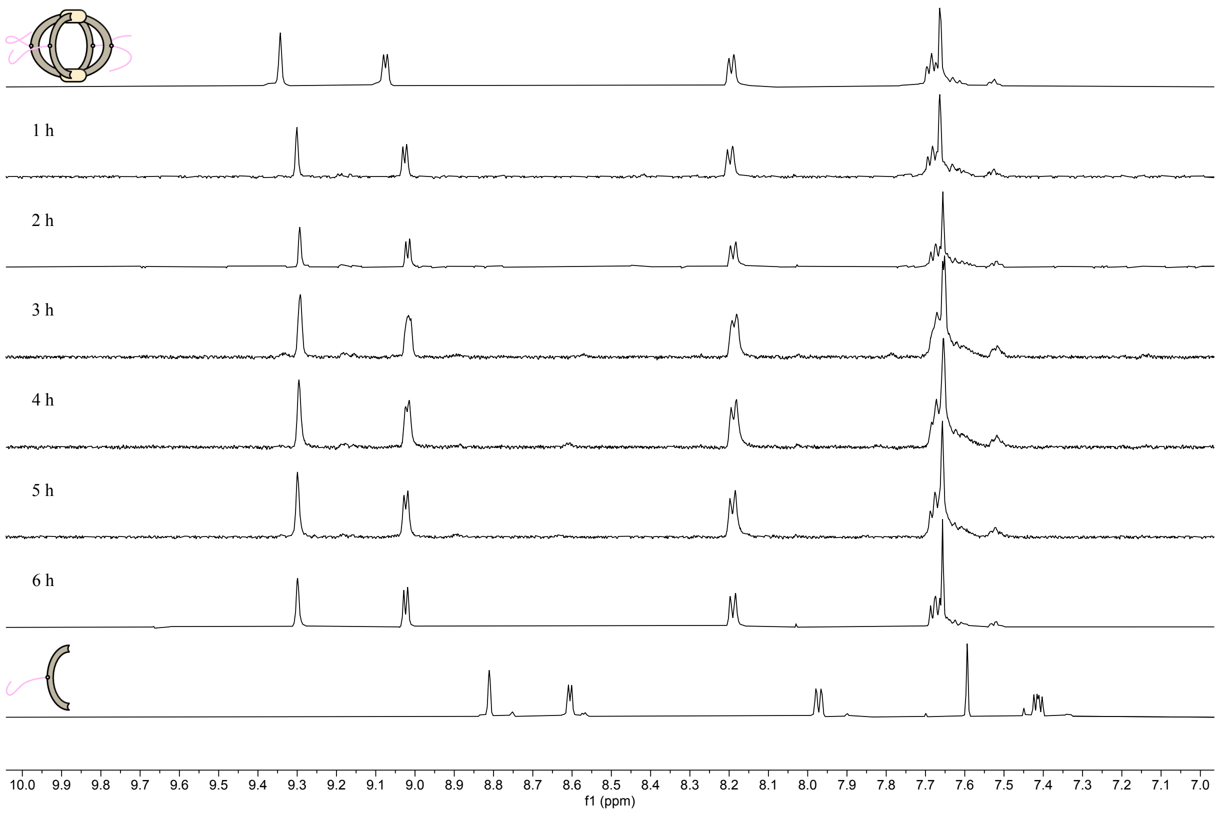

Figure S36: Enlarged view of stacked ^{1}H NMR spectra (600 MHz, CD_3CN, 298 K) showing the disassembly of **PolyMOC1** progressing over time. The spectra correspond to the initial complex (top), six sequential measurements recorded after each hour of sonication (middle), and **PolyL1** (bottom).

PolyMOC2

Sonication in acetonitrile

Nitrogen as saturation gas (c = 2.50 mg mL^{-1}, 125 µM)

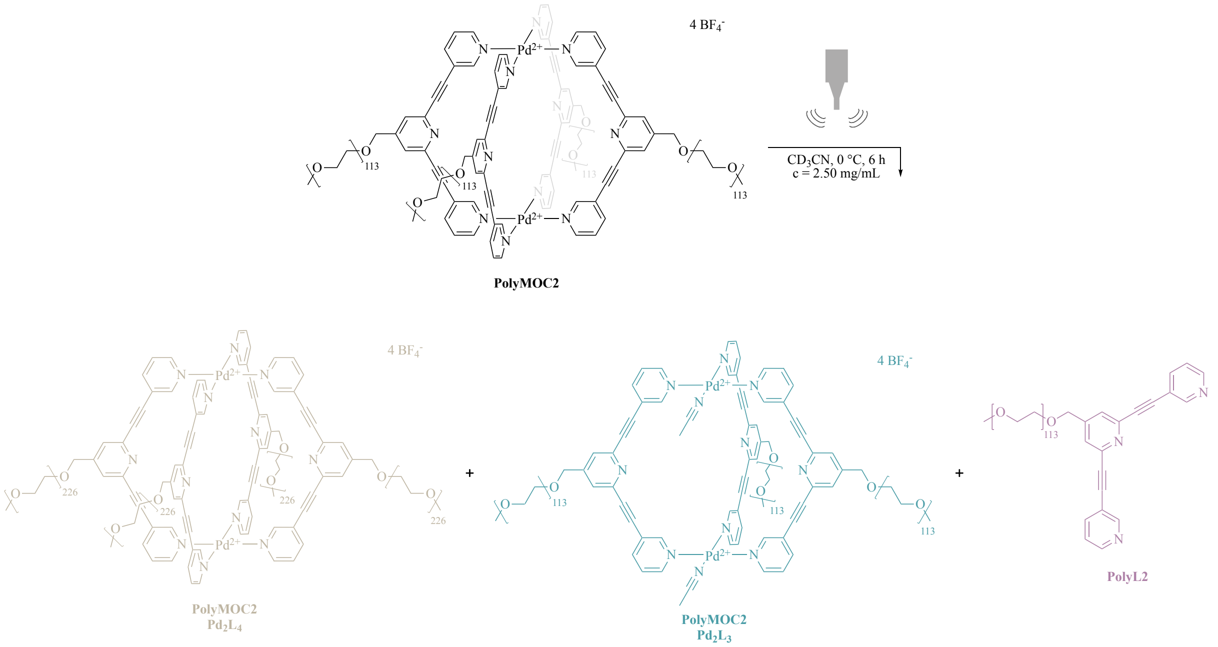

Figure S37: Reaction scheme depicting the sonication-induced disassembly of **PolyMOC2** (2.50 mg mL^{-1}) under nitrogen saturation. Over time, a mixture of **Pd_2L_4**, **Pd_2L_3**, and the free ligand **PolyL2** was observed.

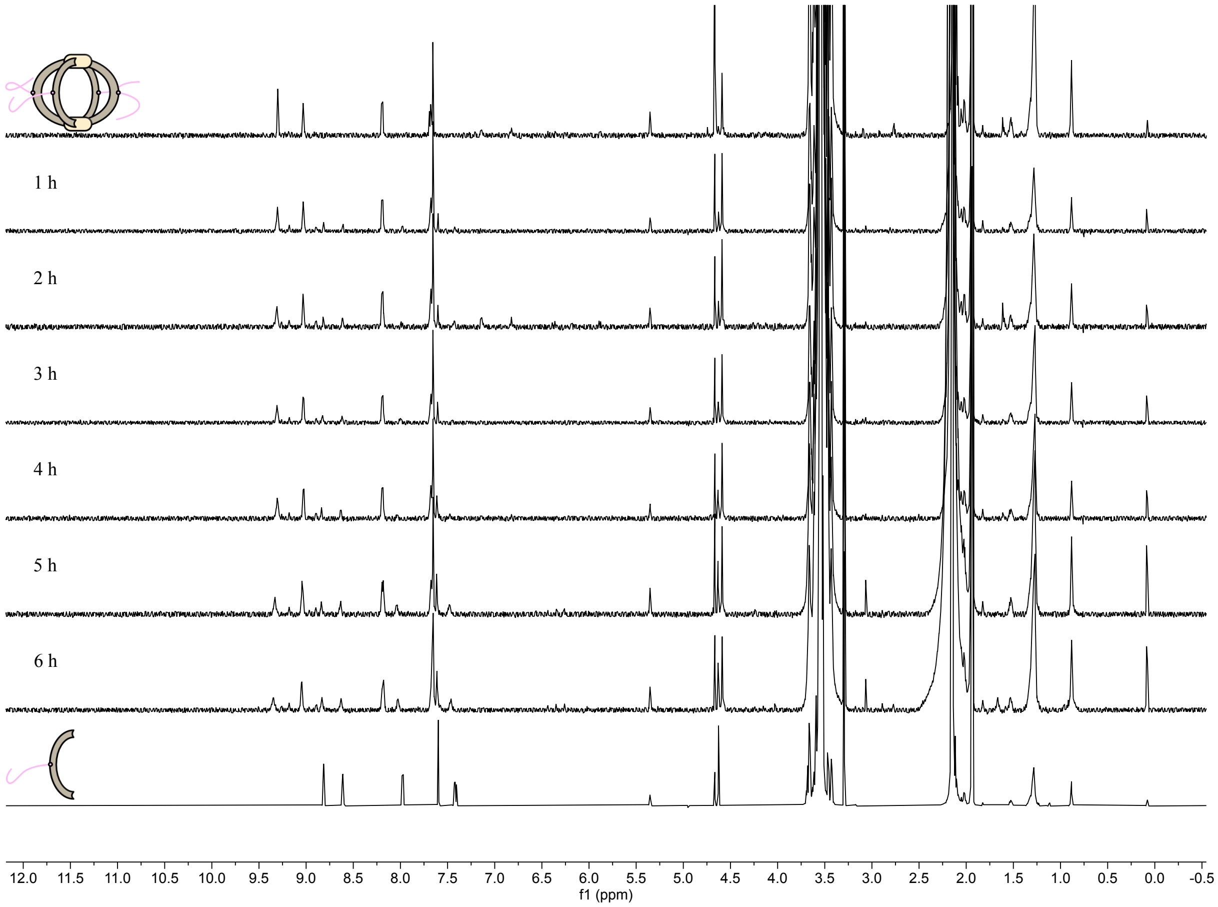

Figure S38: Stacked ^{1}H NMR spectra (600 MHz, CD_3CN, 298 K) showing the disassembly of **PolyMOC2** progressing over time. The spectra correspond to the initial complex (top), six sequential measurements recorded after each hour of sonication (middle), and **PolyL2** (bottom).

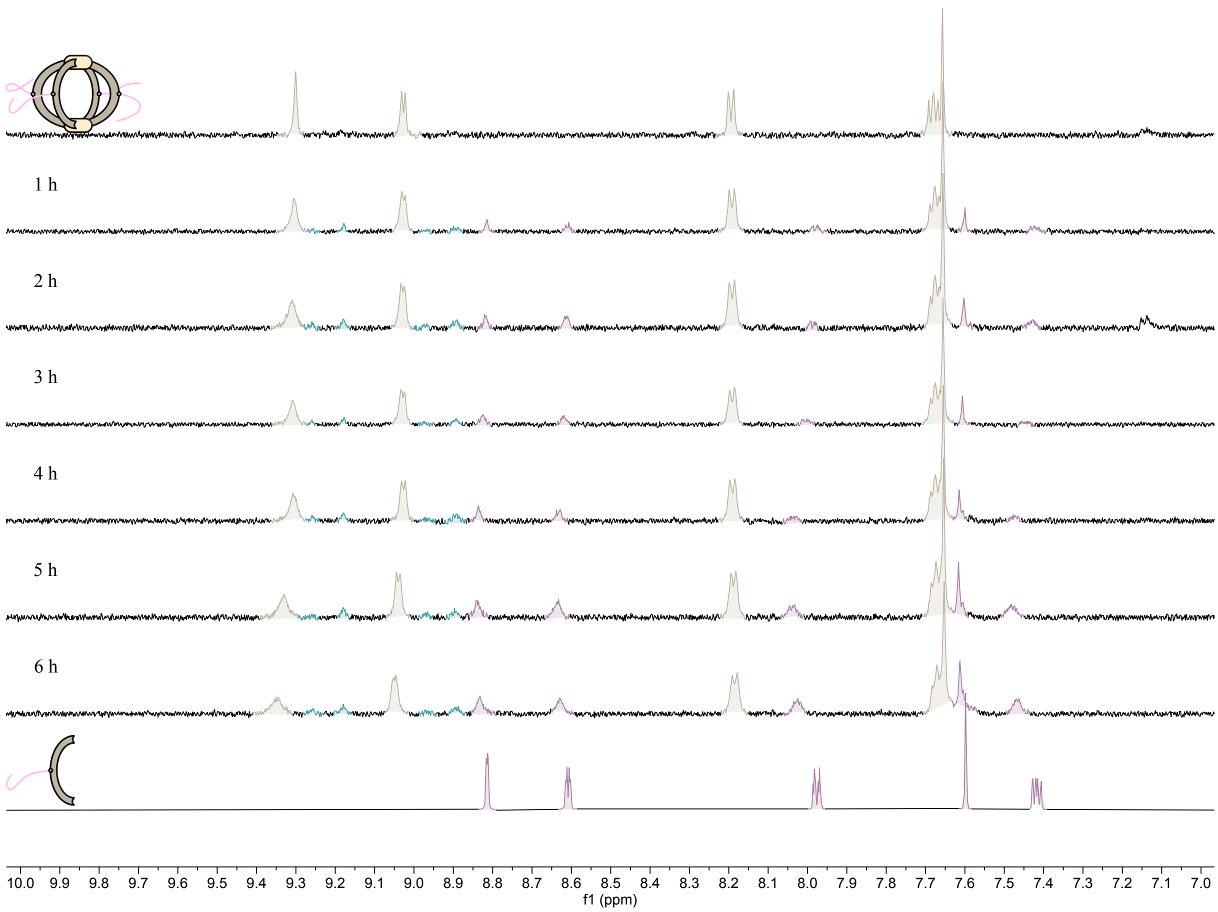

Figure S39: Enlarged view of stacked ^{1}H NMR spectra (600 MHz, CD_3CN, 298 K) showing the disassembly of **PolyMOC2** progressing over time. The spectra correspond to the initial complex (top), six sequential measurements recorded after each hour of sonication (middle), and **PolyL2** (bottom). The ^{1}H NMR signals are color-coded to indicate their respective species: **Pd_2L_4** (brown), **Pd_2L_3** (blue), and **PolyL2** (purple).

Nitrogen as saturation gas (c = 5.00 mg mL^{-1}, 250 µM)

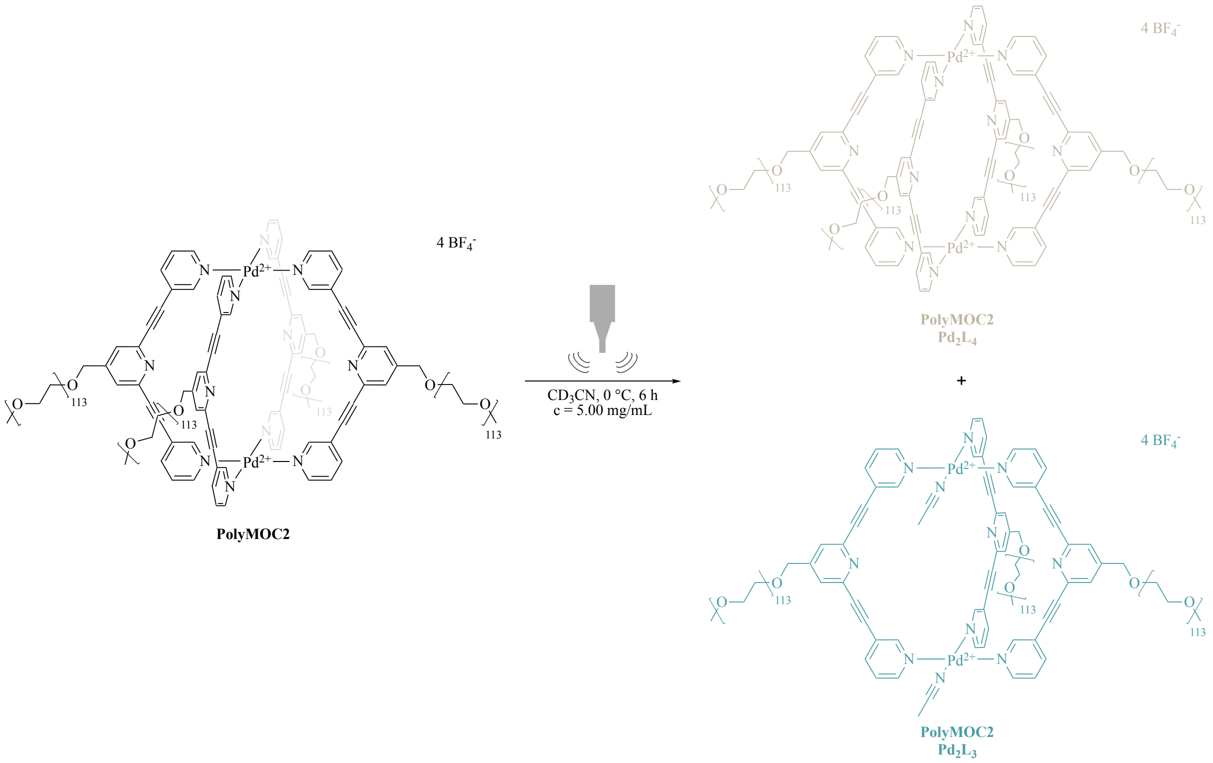

Figure S40: Reaction scheme depicting the sonication-induced disassembly of **PolyMOC2** (5.00 mg mL^{-1}) under nitrogen saturation. Over time, a mixture of **Pd_2L_4** and **Pd_2L_3** was observed.

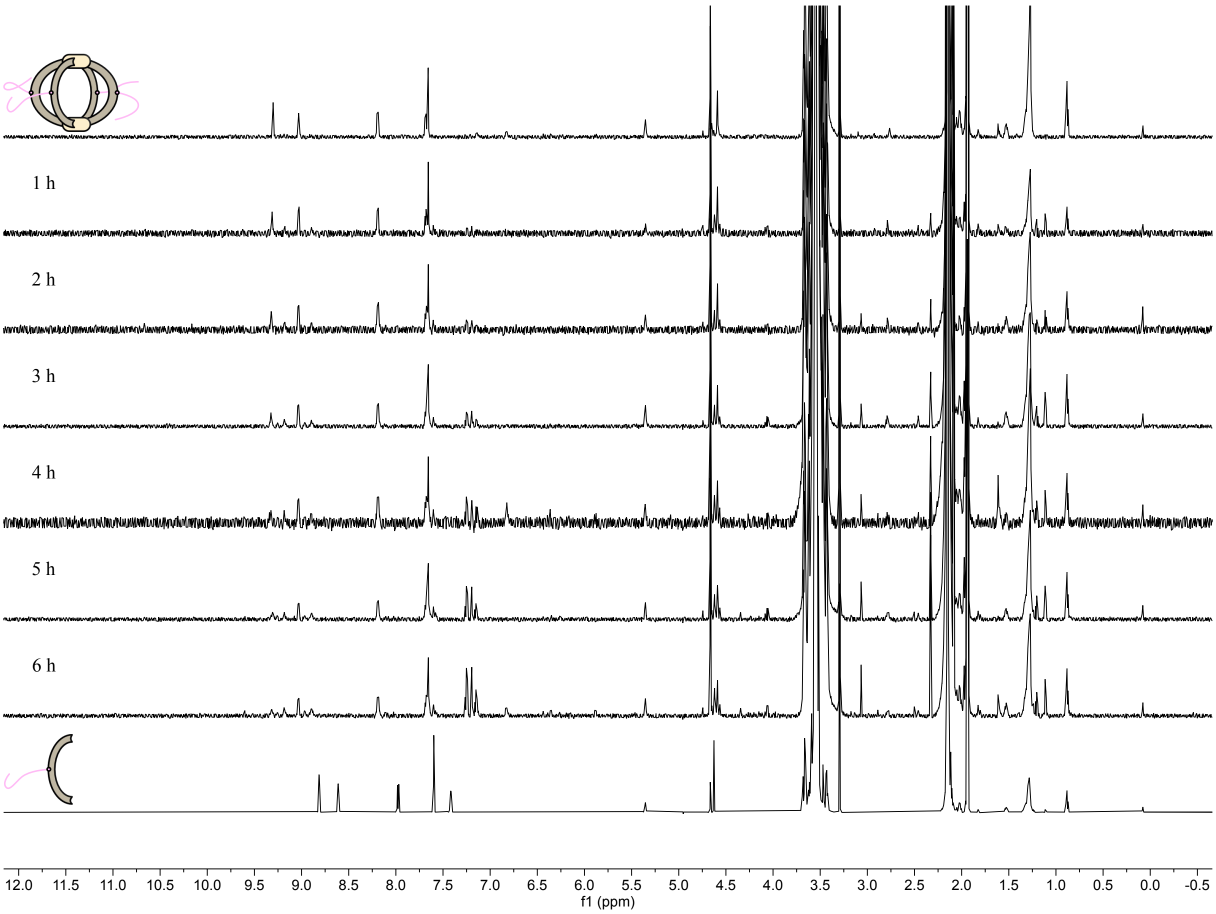

Figure S41: Stacked ^{1}H NMR spectra (600 MHz, CD_3CN, 298 K) showing the disassembly of **PolyMOC2** progressing over time. The spectra correspond to the initial complex (top), six sequential measurements recorded after each hour of sonication (middle), and **PolyL2** (bottom).

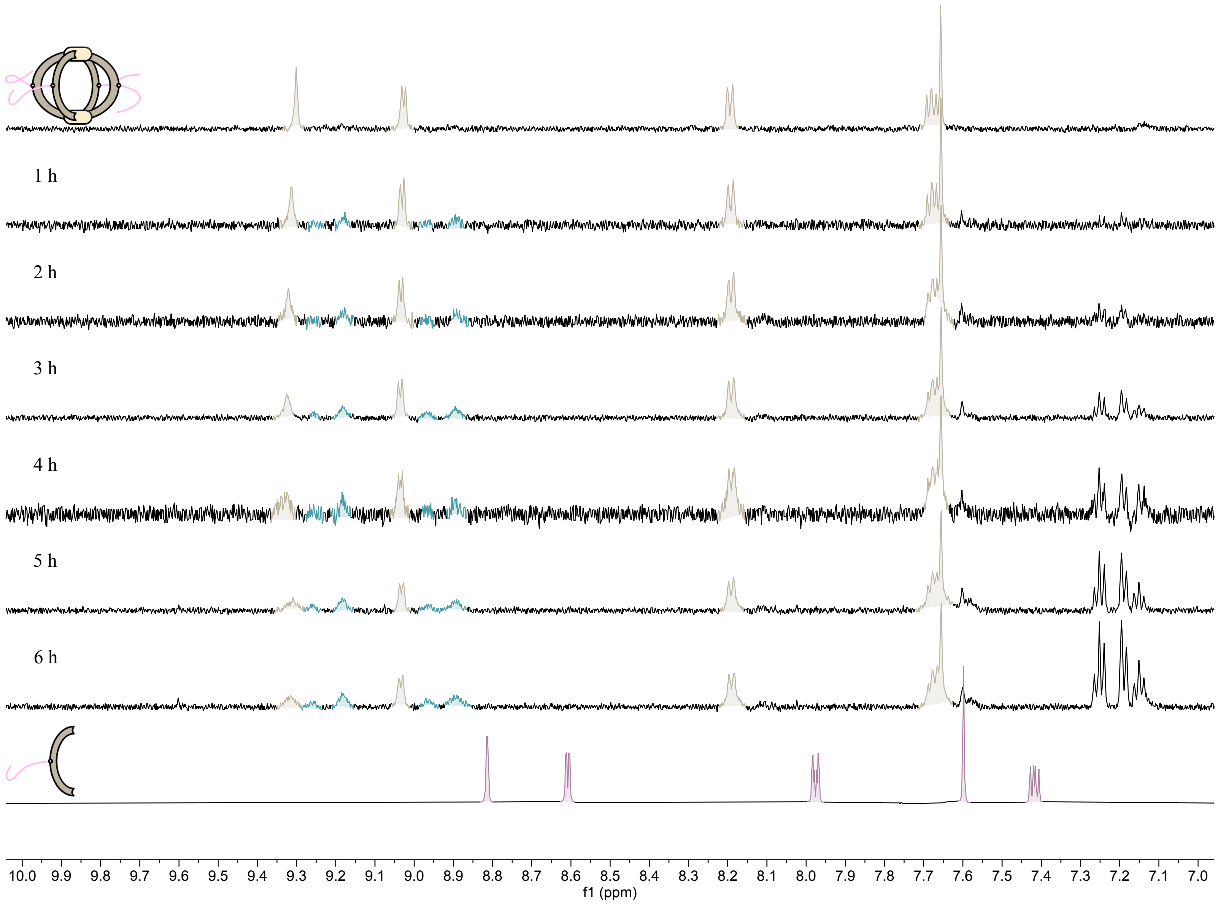

Figure S42: Enlarged view of stacked ^{1}H NMR spectra (600 MHz, CD_3CN, 298 K) showing the disassembly of **PolyMOC2** progressing over time. The spectra correspond to the initial complex (top), six sequential measurements recorded after each hour of sonication (middle), and **PolyL2** (bottom). The ^{1}H NMR signals are color-coded to indicate their respective species: **Pd_2L_4** (brown), **Pd_2L_3** (blue), and **PolyL2** (purple).

Argon as saturation gas (c = 2.50 mg mL^{-1}, 125 µM)

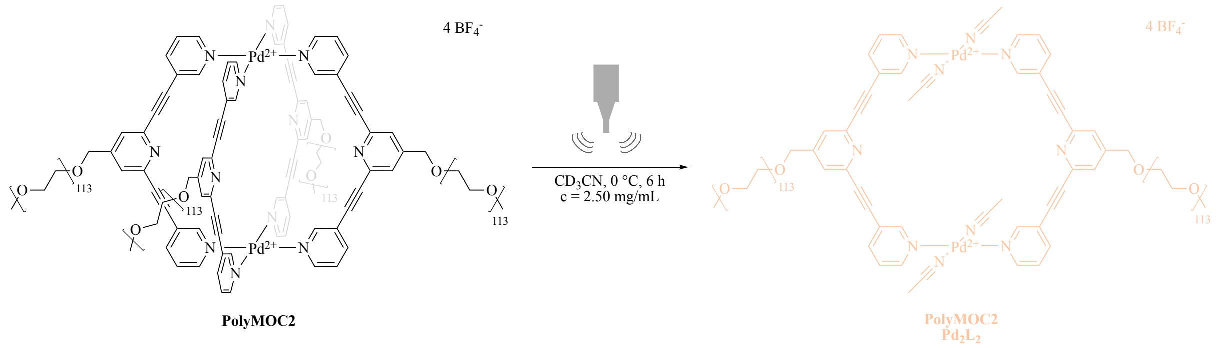

Figure S43: Reaction scheme depicting the sonication-induced disassembly of **PolyMOC2** (2.50 mg mL^{-1}) under argon saturation. Over time, only one **Pd_2L_2** species was observed.

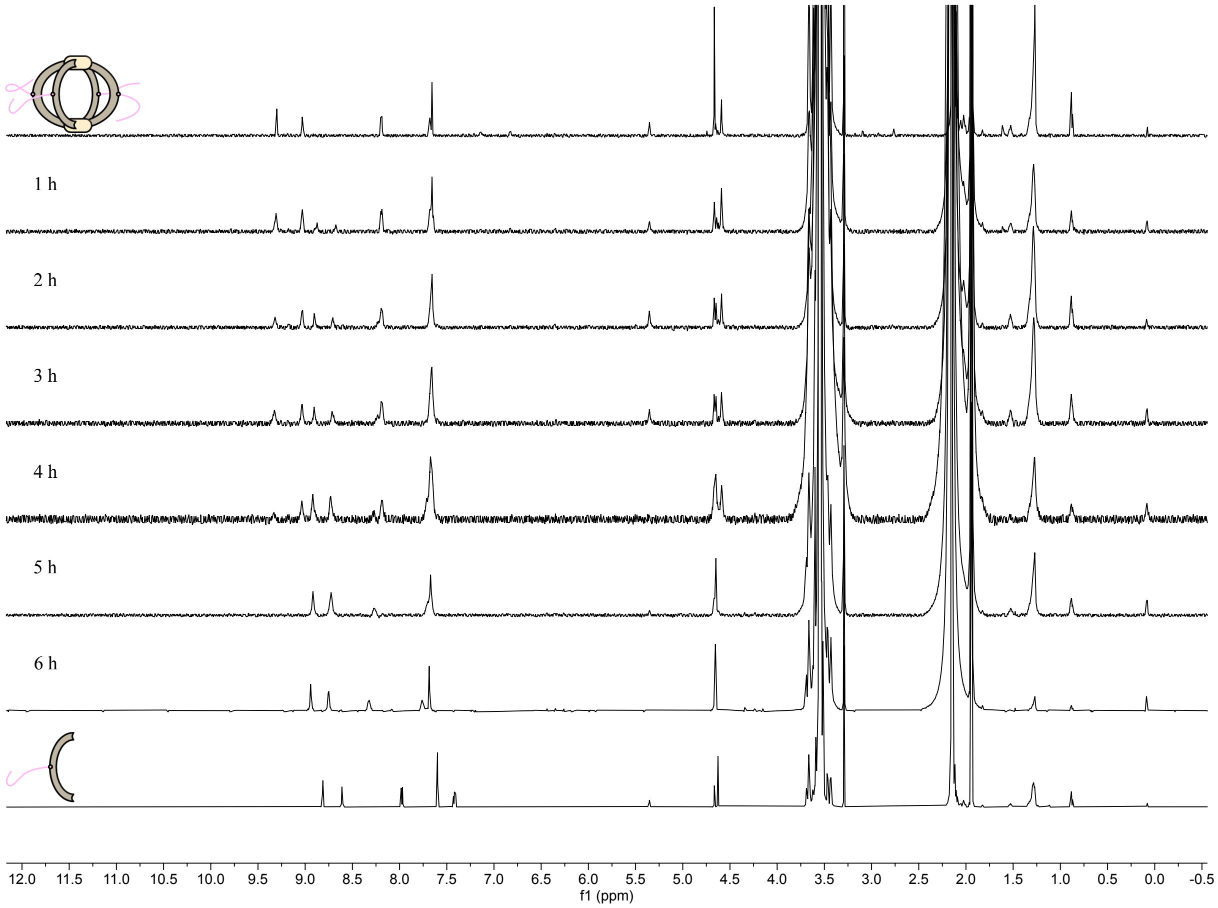

Figure S44: Stacked ^{1}H NMR spectra (600 MHz, CD_3CN, 298 K) showing the disassembly of **PolyMOC2** progressing over time. The spectra correspond to the initial complex (top), six sequential measurements recorded after each hour of sonication (middle), and **PolyL2** (bottom).

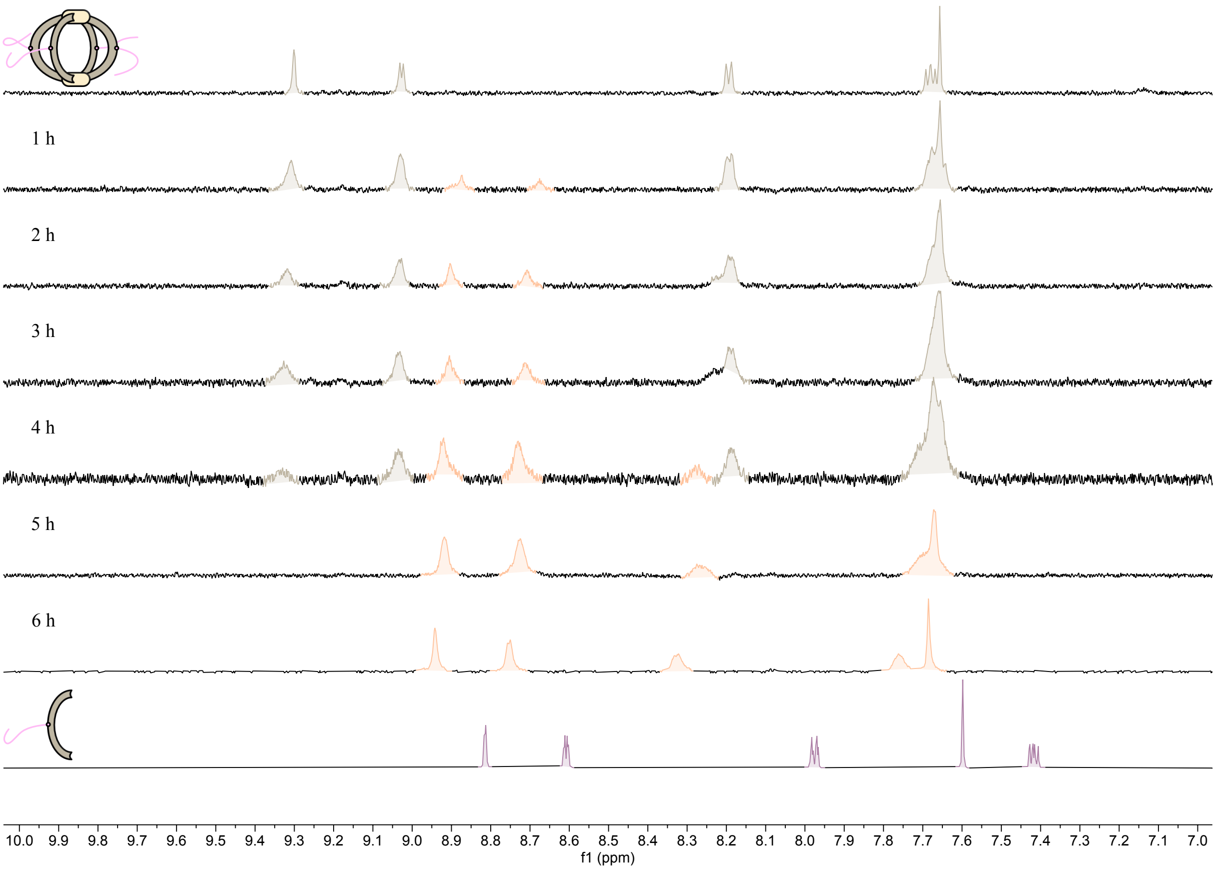

Figure S45: Enlarged view of stacked ^{1}H NMR spectra (600 MHz, CD_3CN, 298 K) showing the disassembly of **PolyMOC2** progressing over time. The spectra correspond to the initial complex (top), six sequential measurements recorded after each hour of sonication (middle), and **PolyL2** (bottom). The ^{1}H NMR signals are color-coded to indicate their respective species: **Pd_2L_4** (brown), **Pd_2L_2** (orange), and **PolyL2** (purple).

Argon as saturation gas (c = 5.00 mg mL^{-1}, 250 µM)

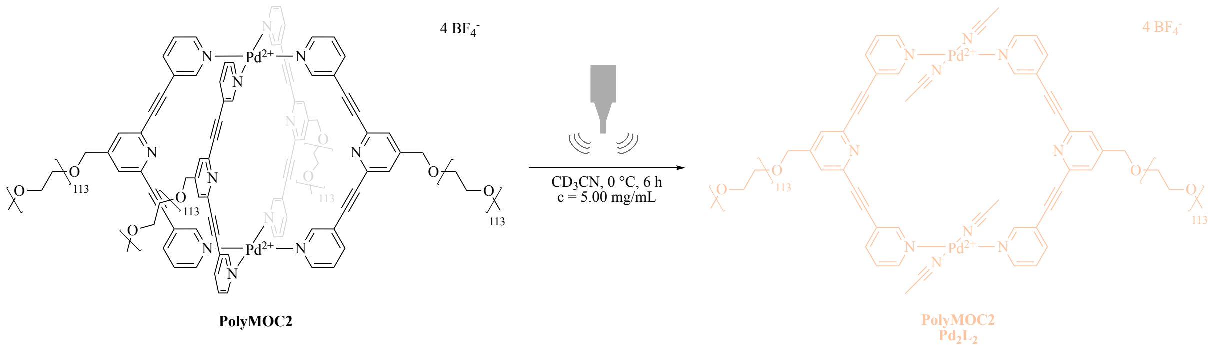

Figure S46: Reaction scheme depicting the sonication-induced disassembly of **PolyMOC2** (5.00 mg mL^{-1}) under argon saturation. Over time, only one **Pd_2L_2** species was observed.

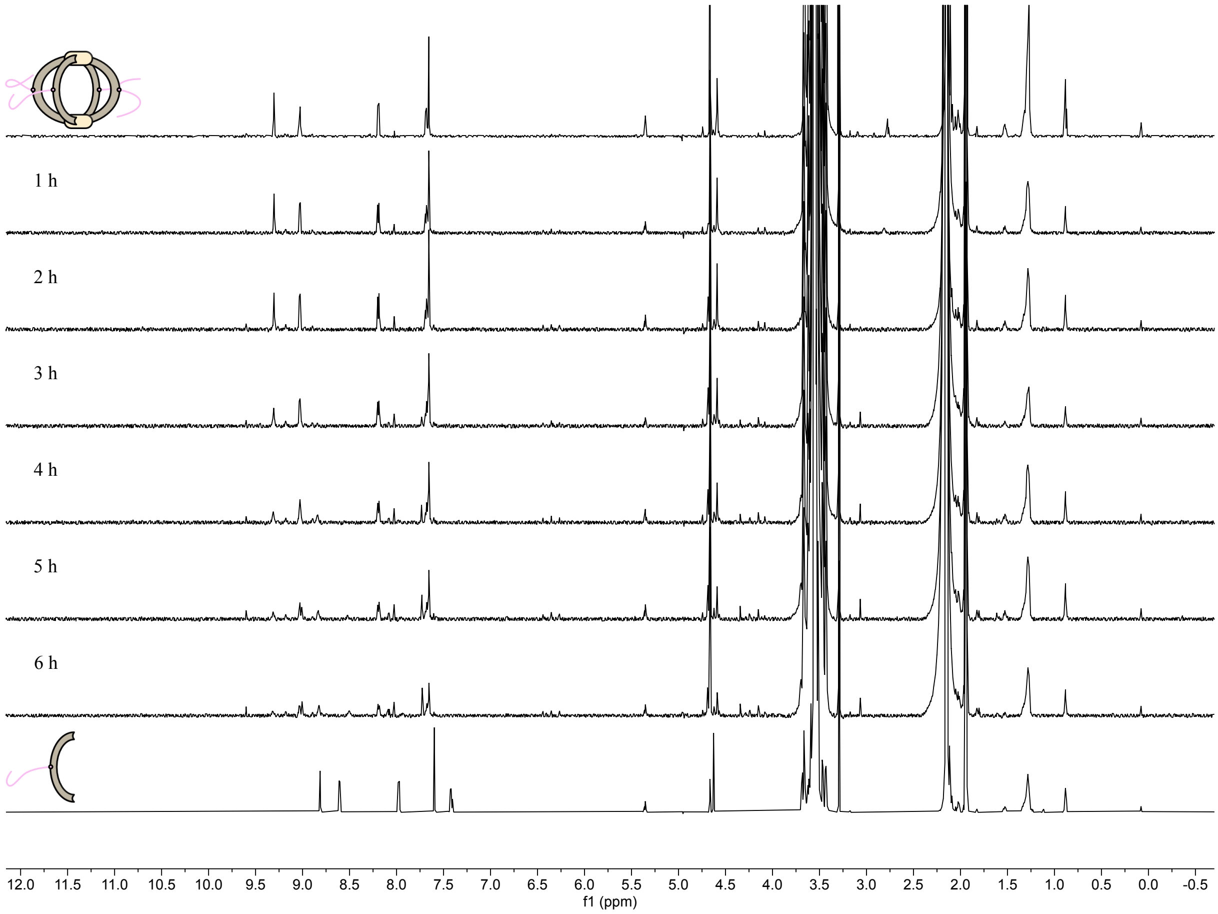

Figure S47: Stacked ^{1}H NMR spectra (600 MHz, CD_3CN, 298 K) showing the disassembly of **PolyMOC2** progressing over time. The spectra correspond to the initial complex (top), six sequential measurements recorded after each hour of sonication (middle), and **PolyL2** (bottom).

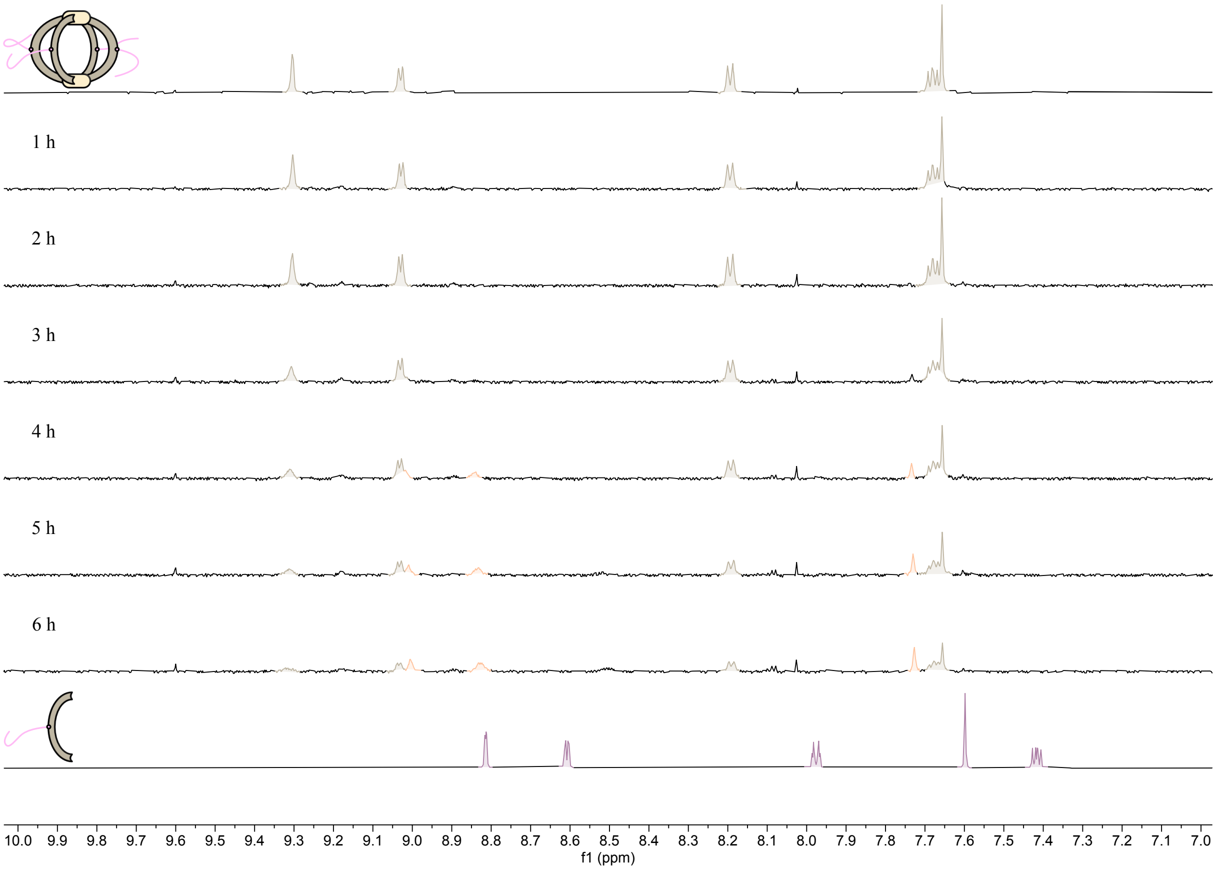

Figure S48: Enlarged view of stacked ^{1}H NMR spectra (600 MHz, CD_3CN, 298 K) showing the disassembly of **PolyMOC2** progressing over time. The spectra correspond to the initial complex (top), six sequential measurements recorded after each hour of sonication (middle), and **PolyL2** (bottom). The ^{1}H NMR signals are color-coded to indicate their respective species: **Pd_2L_4** (brown), **Pd_2L_2** (orange), and **PolyL3** (purple).

PolyMOC3

Sonication in acetonitrile

Nitrogen as saturation gas (c = 2.50 mg mL^{-1}, 62.5 µM)

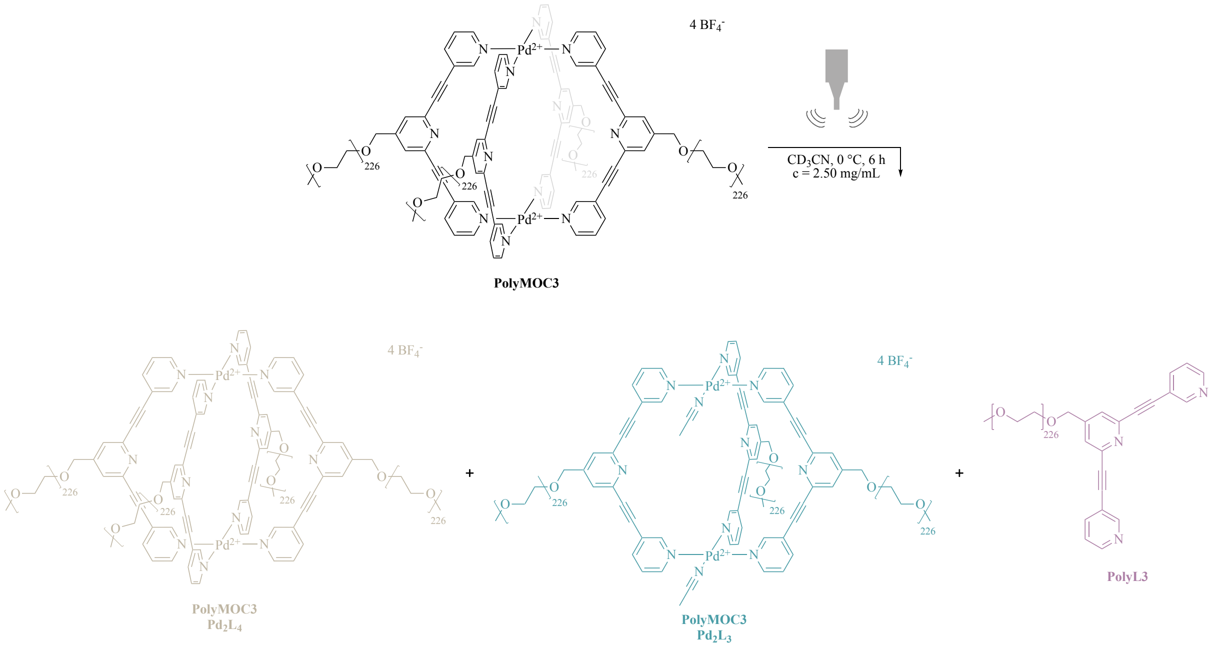

Figure S49: Reaction scheme depicting the sonication-induced disassembly of **PolyMOC3** (2.50 mg mL^{-1}) under nitrogen saturation. Over time, a mixture of **Pd_2L_4**, **Pd_2L_3**, and the free ligand **PolyL3** was observed.

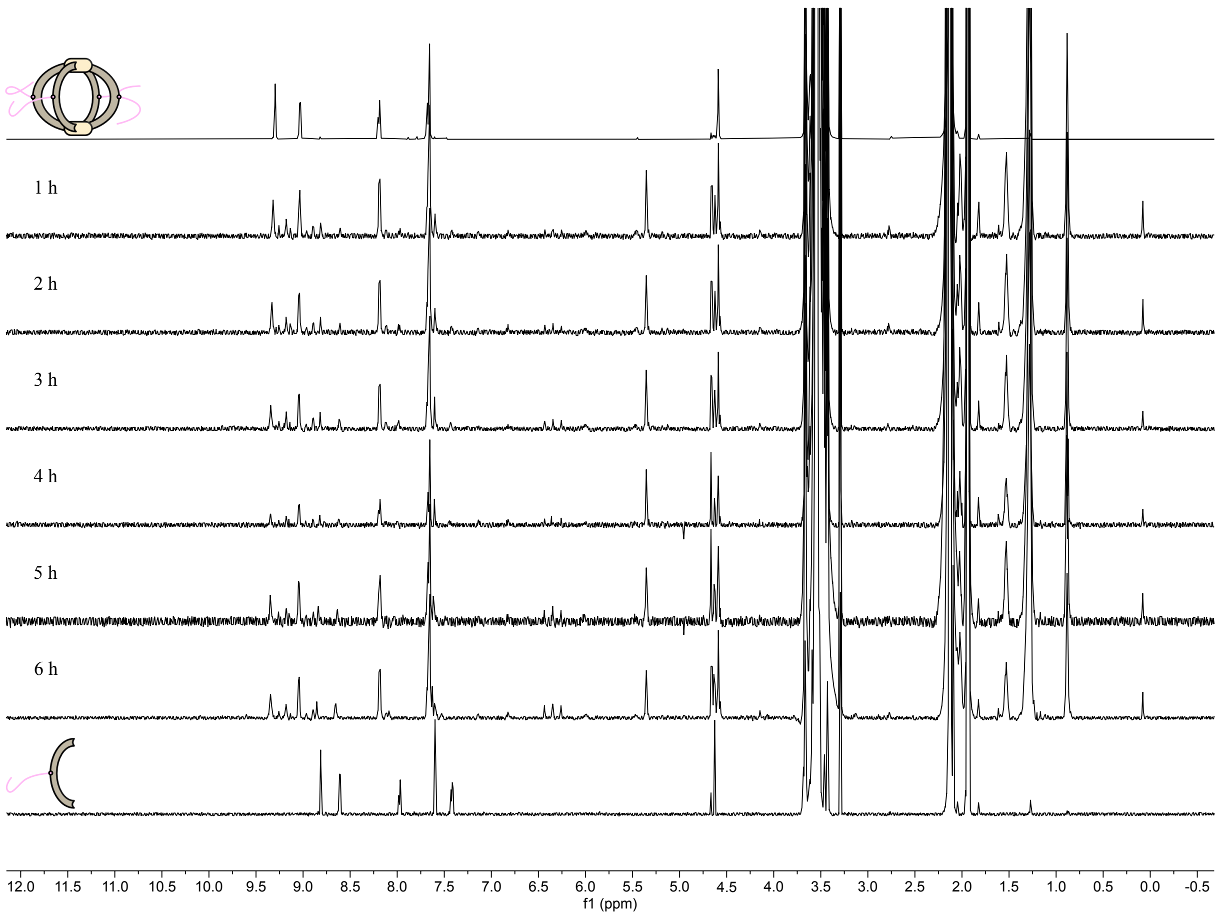

Figure S50: Stacked ^{1}H NMR spectra (600 MHz, CD_3CN, 298 K) showing the disassembly of **PolyMOC3** progressing over time. The spectra correspond to the initial complex (top), six sequential measurements recorded after each hour of sonication (middle), and **PolyL3** (bottom).

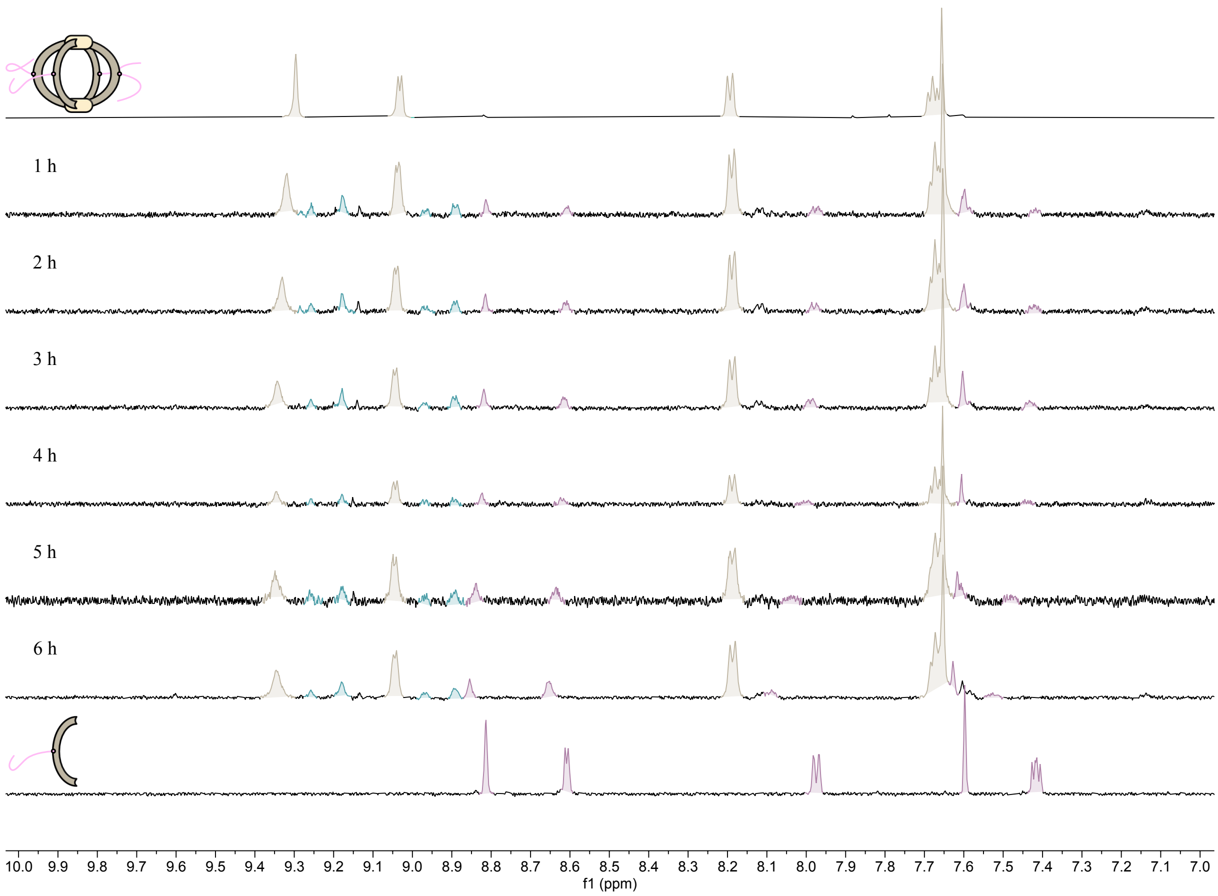

Figure S51: Enlarged view of stacked ^{1}H NMR spectra (600 MHz, CD_3CN, 298 K) showing the disassembly of **PolyMOC3** progressing over time. The spectra correspond to the initial complex (top), six sequential measurements recorded after each hour of sonication (middle), and **PolyL3** (bottom). The ^{1}H NMR signals are color-coded to indicate their respective species: **Pd_2L_4** (brown), **Pd_2L_3** (blue), and **PolyL3** (purple).

Nitrogen as saturation gas (c = 5.00 mg mL^{-1}, 125 μM)

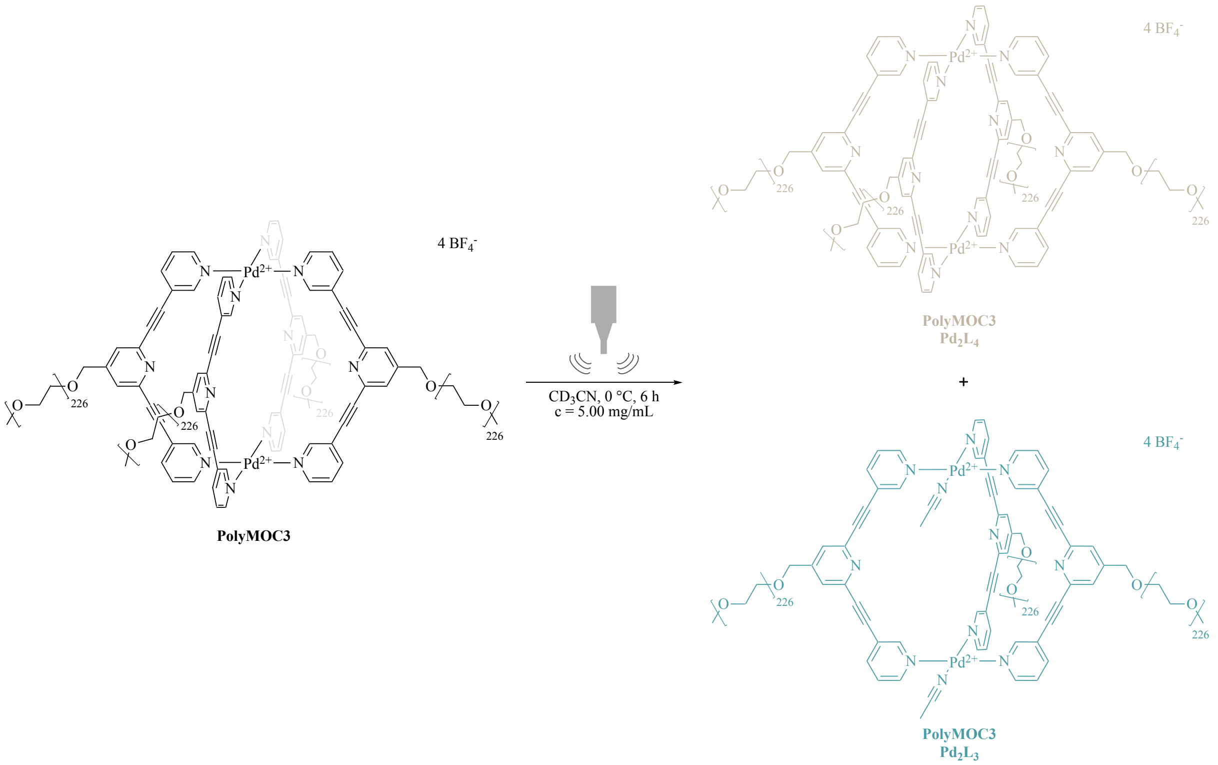

Figure S52: Reaction scheme depicting the sonication-induced disassembly of **PolyMOC3** (5.00 mg mL^{-1}) under nitrogen saturation. Over time, a mixture of **Pd$_2$L$_4$** and **Pd$_2$L$_3$** was observed.

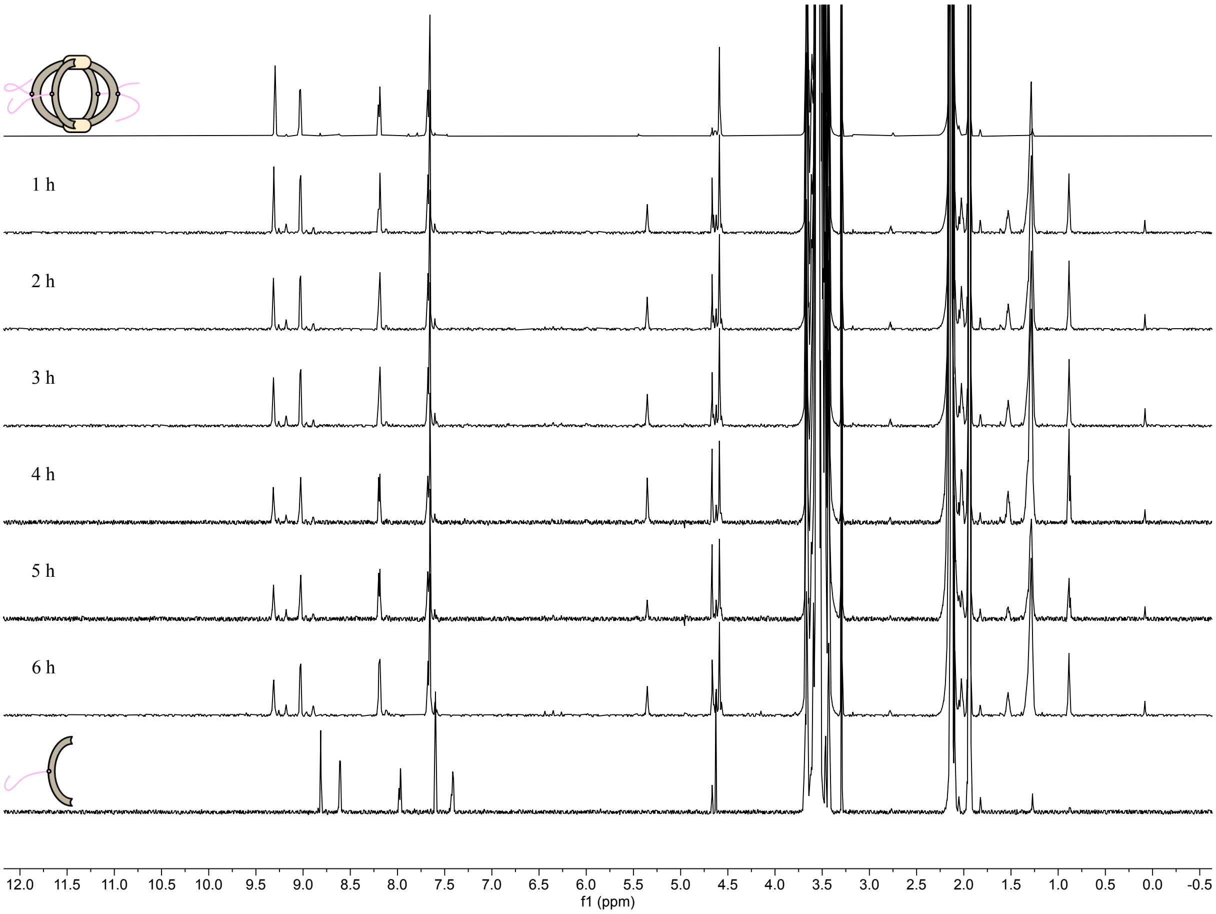

Figure S53: Stacked ^{1}H NMR spectra (600 MHz, CD_3CN, 298 K) showing the disassembly of **PolyMOC3** progressing over time. The spectra correspond to the initial complex (top), six sequential measurements recorded after each hour of sonication (middle), and **PolyL3** (bottom).

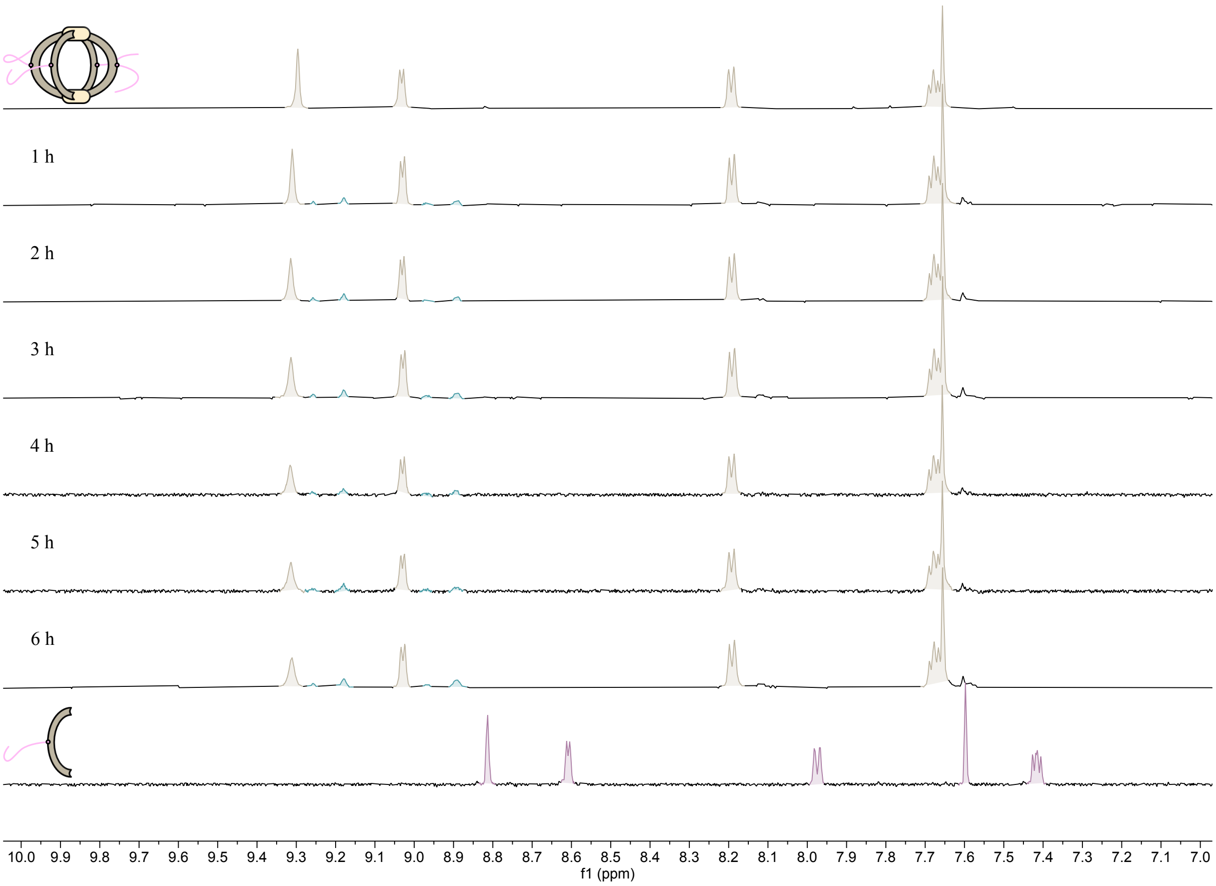

Figure S54: Enlarged view of stacked ^{1}H NMR spectra (600 MHz, CD_3CN, 298 K) showing the disassembly of **PolyMOC3** progressing over time. The spectra correspond to the initial complex (top), six sequential measurements recorded after each hour of sonication (middle), and **PolyL3** (bottom). The ^{1}H NMR signals are color-coded to indicate their respective species: **Pd_2L_4** (brown), **Pd_2L_3** (blue), and **PolyL3** (purple).

Argon as saturation gas (c = 2.50 mg mL^{-1}, 62.5 µM)

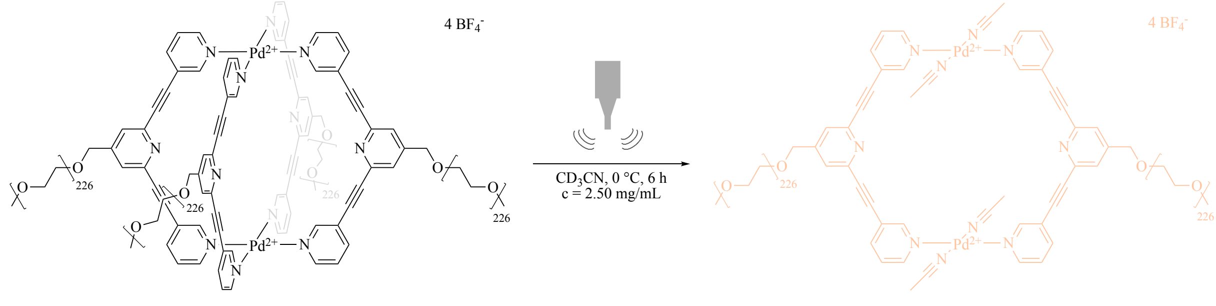

PolyMOC3

PolyMOC3 Pd_2L_2

Figure S55: Reaction scheme depicting the sonication-induced disassembly of **PolyMOC3** (2.50 mg mL^{-1}) under argon saturation. Over time, only one **Pd_2L_2** species was observed.

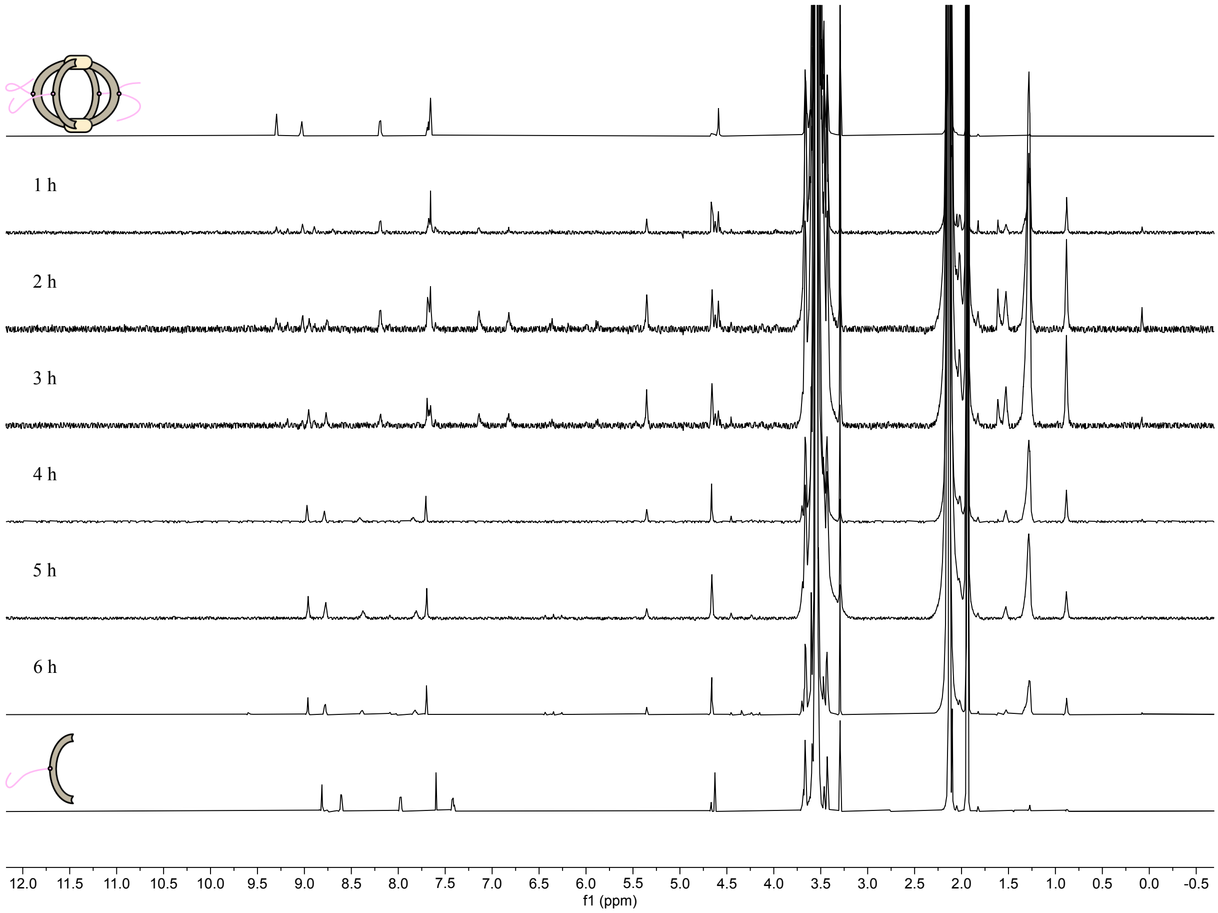

Figure S56: Stacked ^{1}H NMR spectra (600 MHz, CD_3CN, 298 K) showing the disassembly of **PolyMOC3** progressing over time. The spectra correspond to the initial complex (top), six sequential measurements recorded after each hour of sonication (middle), and **PolyL3** (bottom).

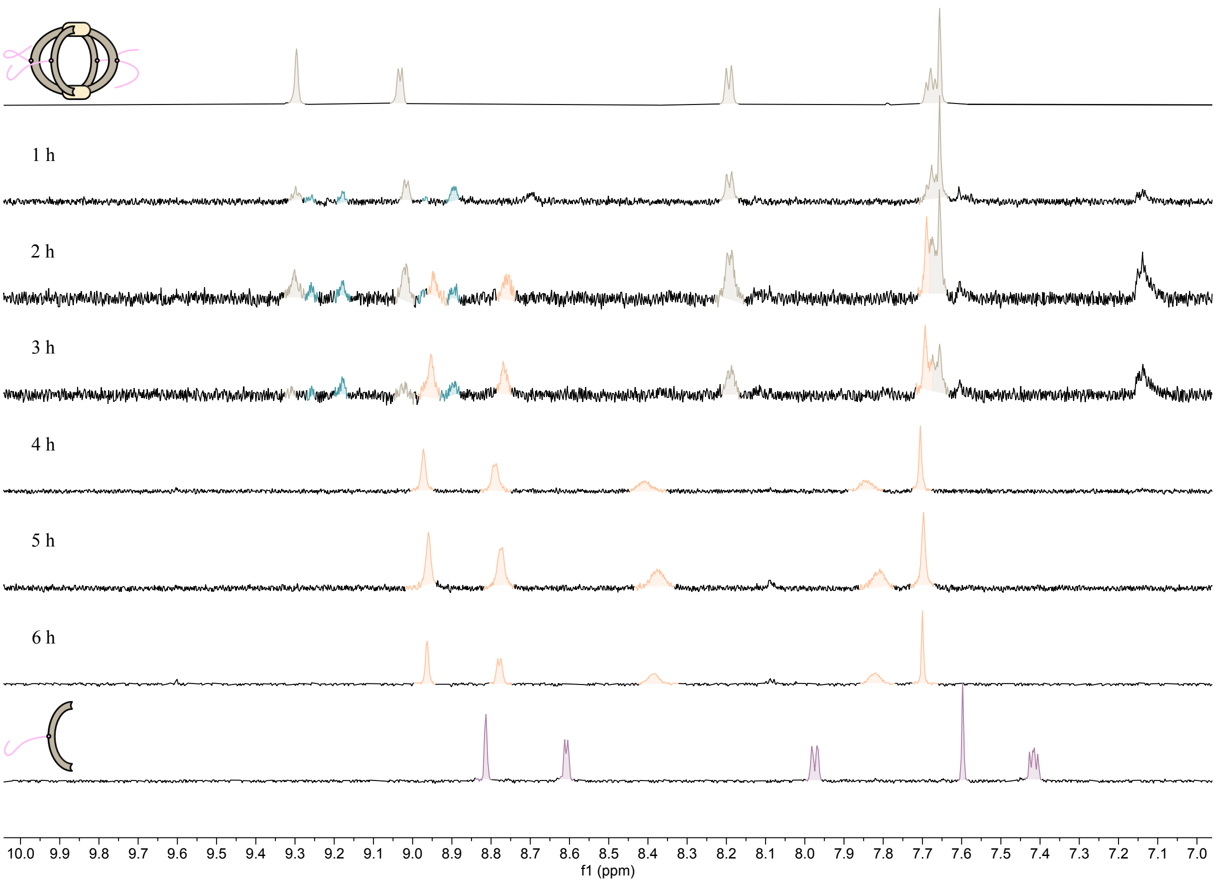

Figure S57: Enlarged view of stacked ^{1}H NMR spectra (600 MHz, CD_3CN, 298 K) showing the disassembly of **PolyMOC3** progressing over time. The spectra correspond to the initial complex (top), six sequential measurements recorded after each hour of sonication (middle), and **PolyL3** (bottom). The ^{1}H NMR signals are color-coded to indicate their respective species: **Pd_2L_4** (brown), **Pd_2L_3** (blue), **Pd_2L_2** (orange), and **PolyL3** (purple).

Argon as saturation gas (c = 5.00 mg mL^{-1}, 125 µM)

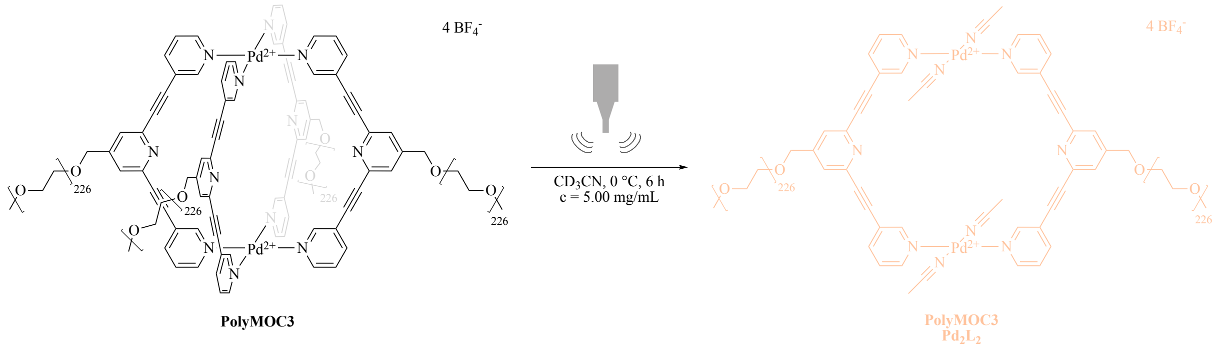

Figure S58: Reaction scheme depicting the sonication-induced disassembly of **PolyMOC3** (5.00 mg mL^{-1}) under argon saturation. Over time, only one **Pd_2L_2** species was observed.

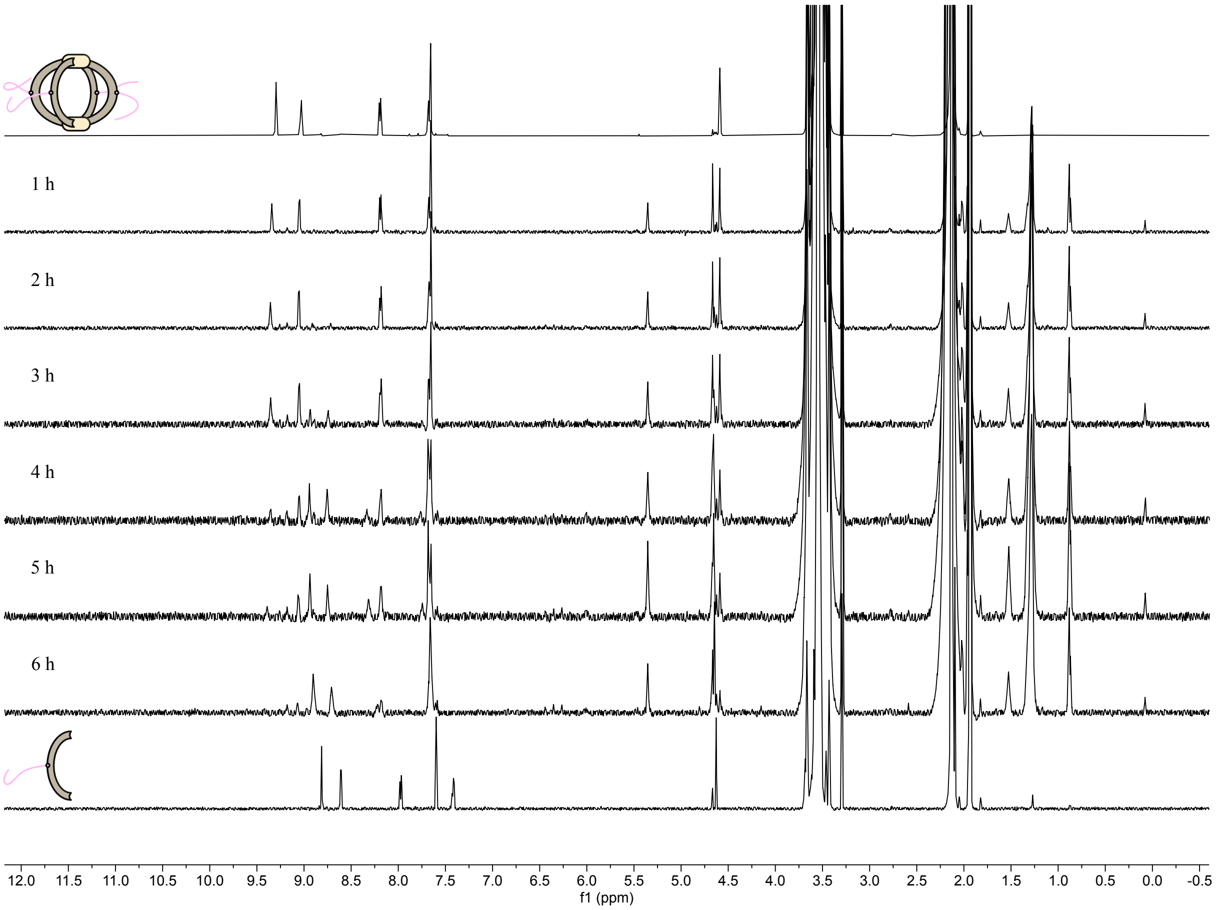

Figure S59: Stacked ^{1}H NMR spectra (600 MHz, CD_3CN, 298 K) showing the disassembly of **PolyMOC3** progressing over time. The spectra correspond to the initial complex (top), six sequential measurements recorded after each hour of sonication (middle), and **PolyL3** (bottom).

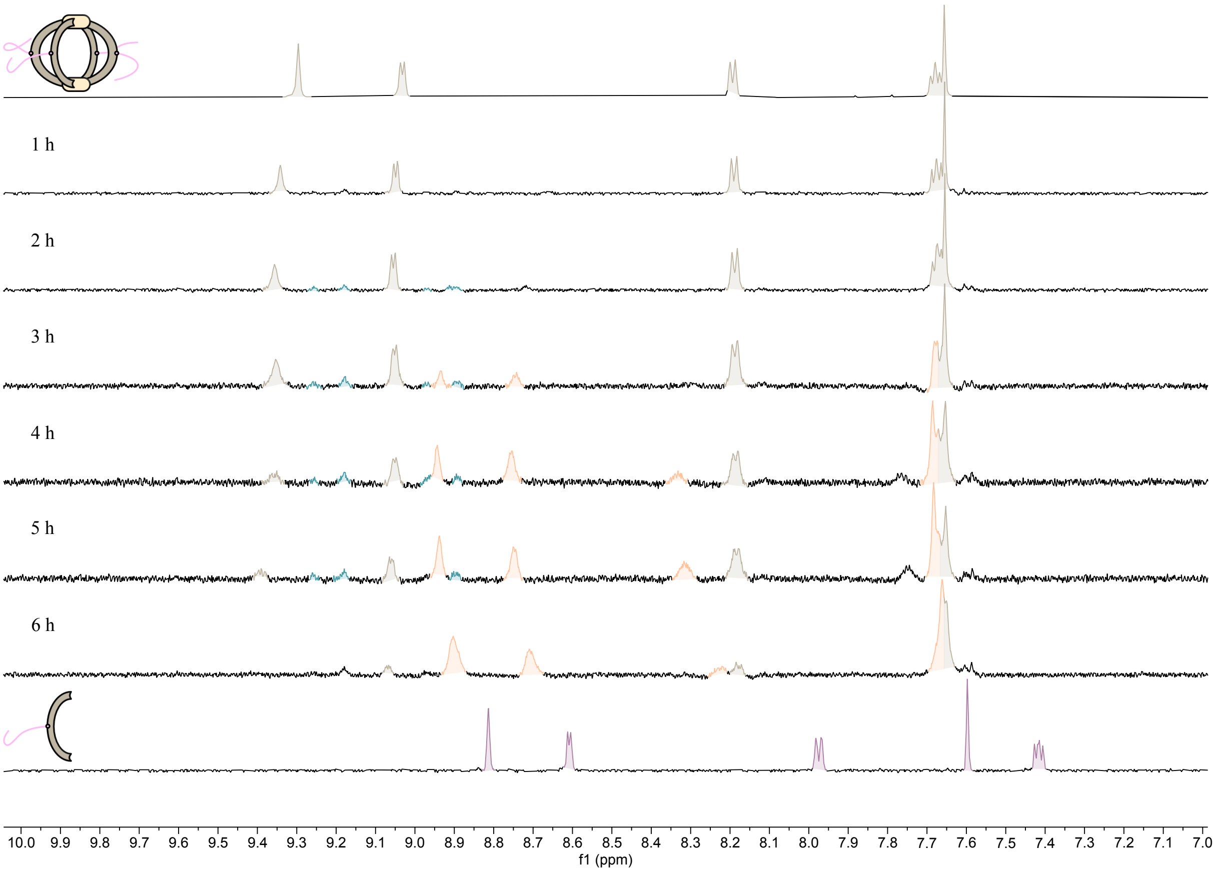

Figure S60: Enlarged view of stacked ^{1}H NMR spectra (600 MHz, CD_3CN, 298 K) showing the disassembly of **PolyMOC2** progressing over time. The spectra correspond to the initial complex (top), six sequential measurements recorded after each hour of sonication (middle), and **PolyL3** (bottom). The ^{1}H NMR signals are color-coded to indicate their respective species: **Pd_2L_4** (brown), **Pd_2L_3** (blue), **Pd_2L_2** (orange), and **PolyL3** (purple).

Sonication in water

Nitrogen as saturation gas

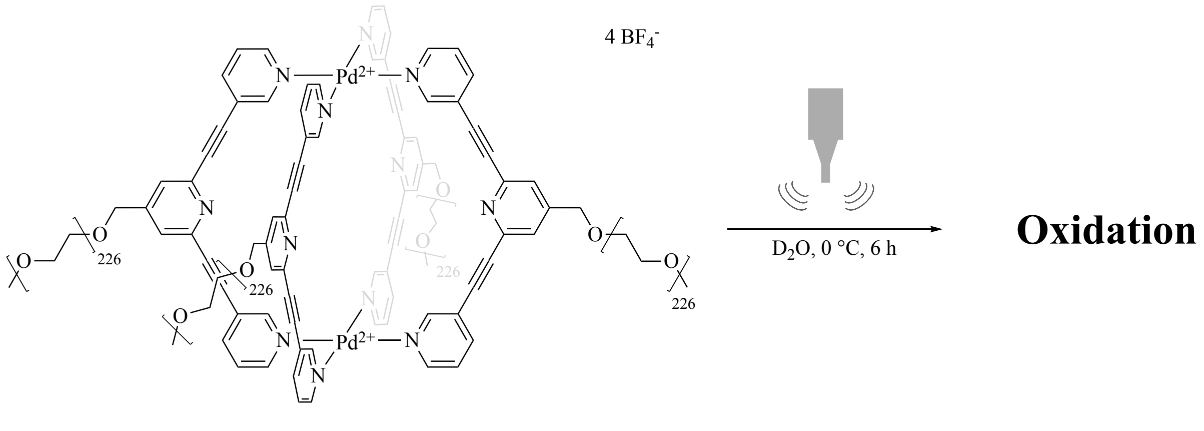

Figure S61: Reaction scheme depicting the sonication-induced disassembly of **PolyMOC3** (2.50 mg mL^{-1}) under nitrogen saturation.

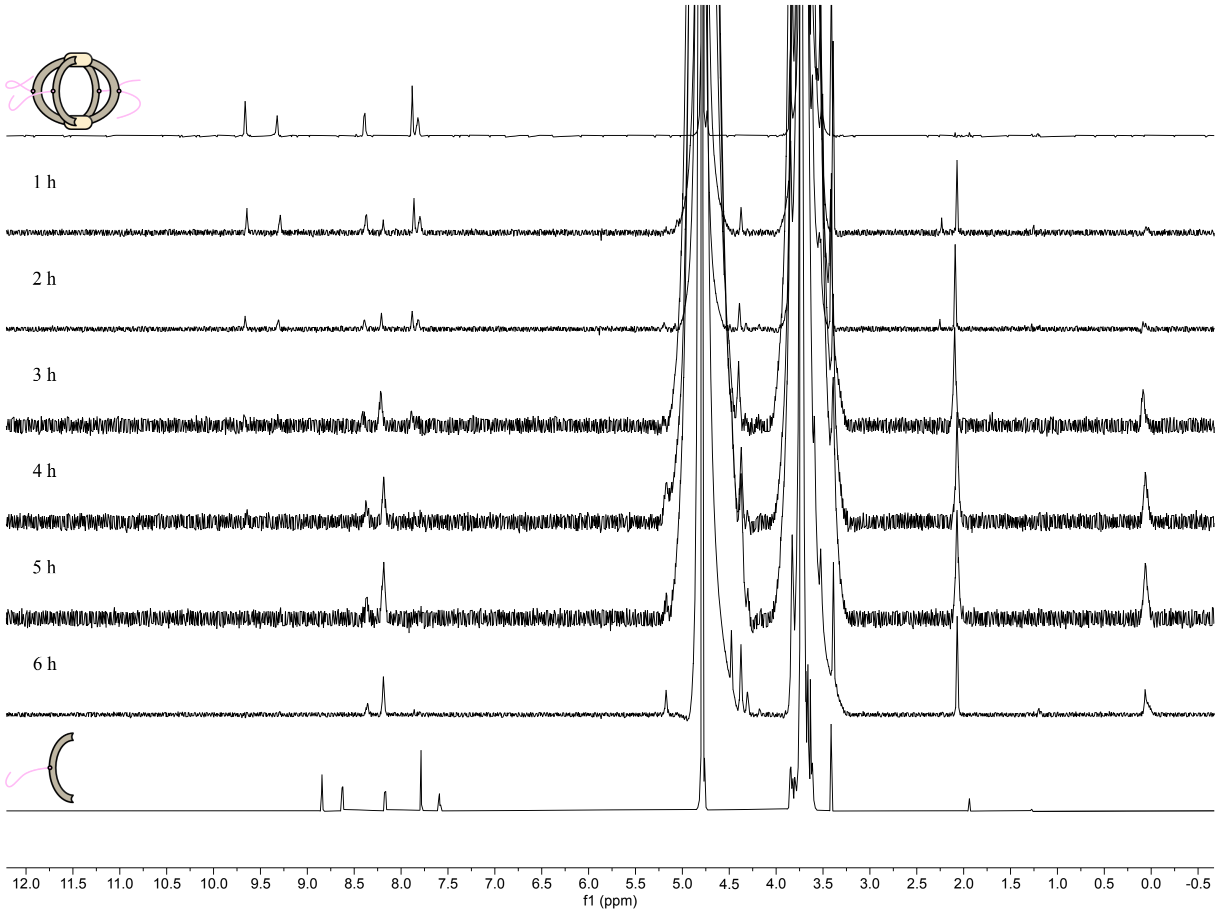

Figure S62: Stacked ^{1}H NMR spectra (600 MHz, D_2O, 298 K) showing the disassembly of **PolyMOC3** progressing over time. The spectra correspond to the initial complex (top), six sequential measurements recorded after each hour of sonication (middle), and **PolyL3** (bottom).

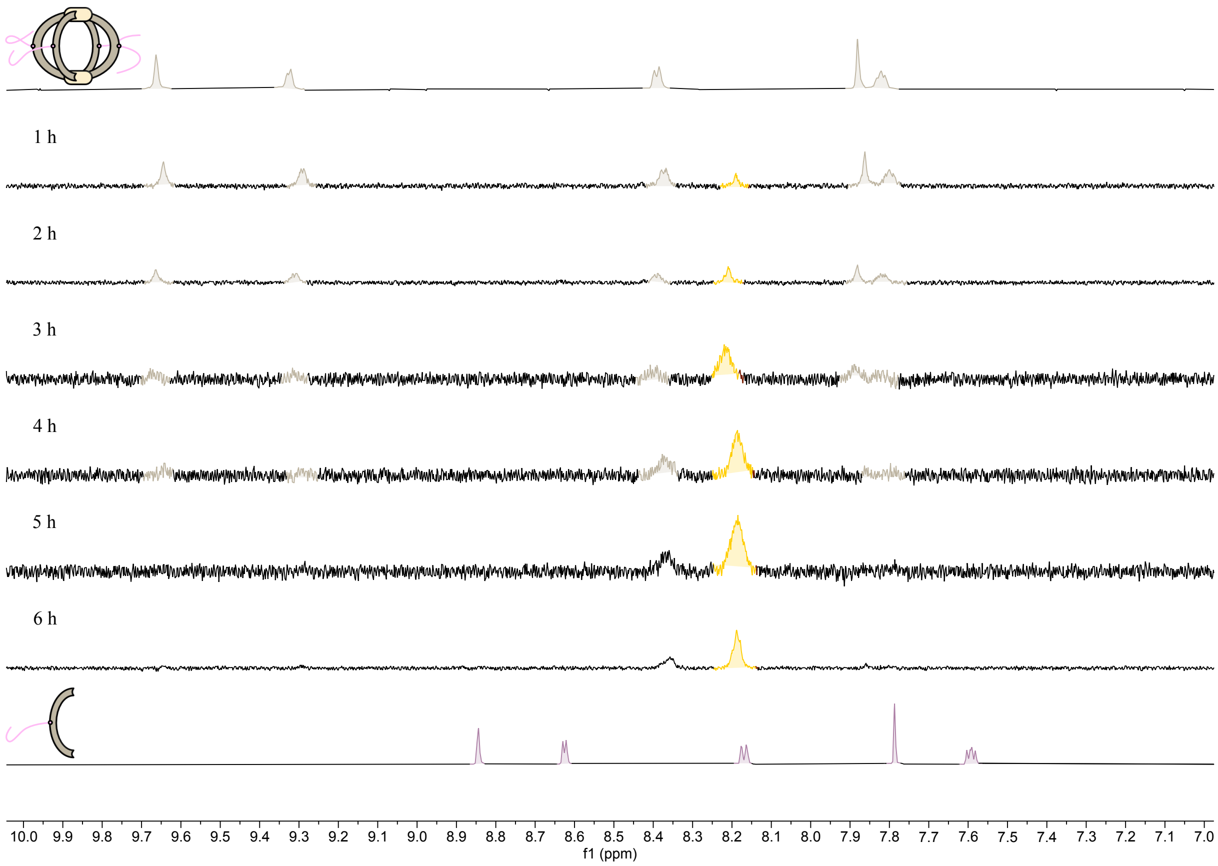

Figure S63: Enlarged view of stacked ^{1}H NMR spectra (600 MHz, D_2O, 298 K) showing the disassembly of **PolyMOC3** progressing over time. The spectra correspond to the initial complex (top), six sequential measurements recorded after each hour of sonication (middle), and **PolyL3** (bottom). The ^{1}H NMR signals are color-coded to indicate their respective species: **Pd_2L_4** (brown), **PolyL3** (purple) and formic acid (yellow) formed from PEG oxidation.

Argon as saturation gas

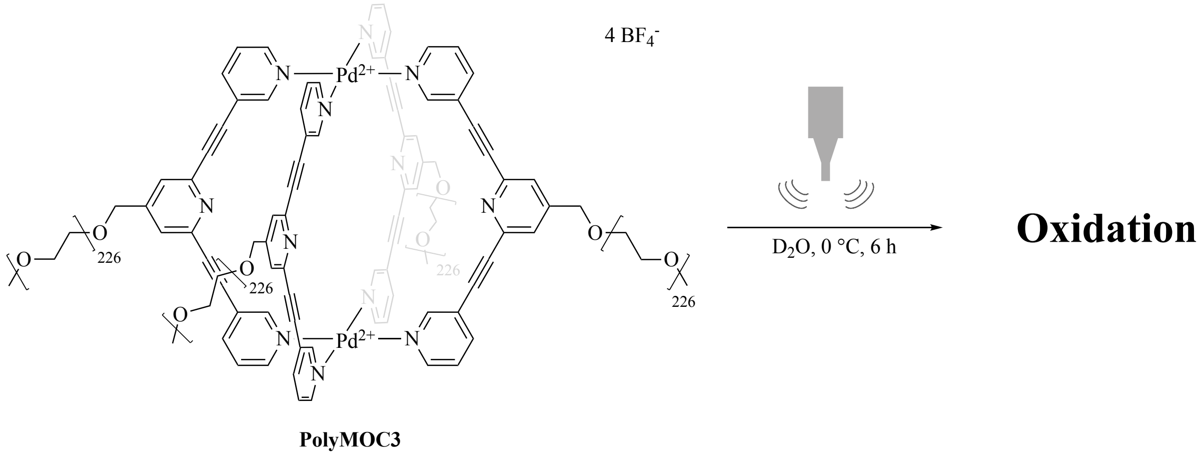

Figure S64: Reaction scheme depicting the sonication-induced disassembly of **PolyMOC3** (2.50 mg mL^{-1}) under argon saturation.

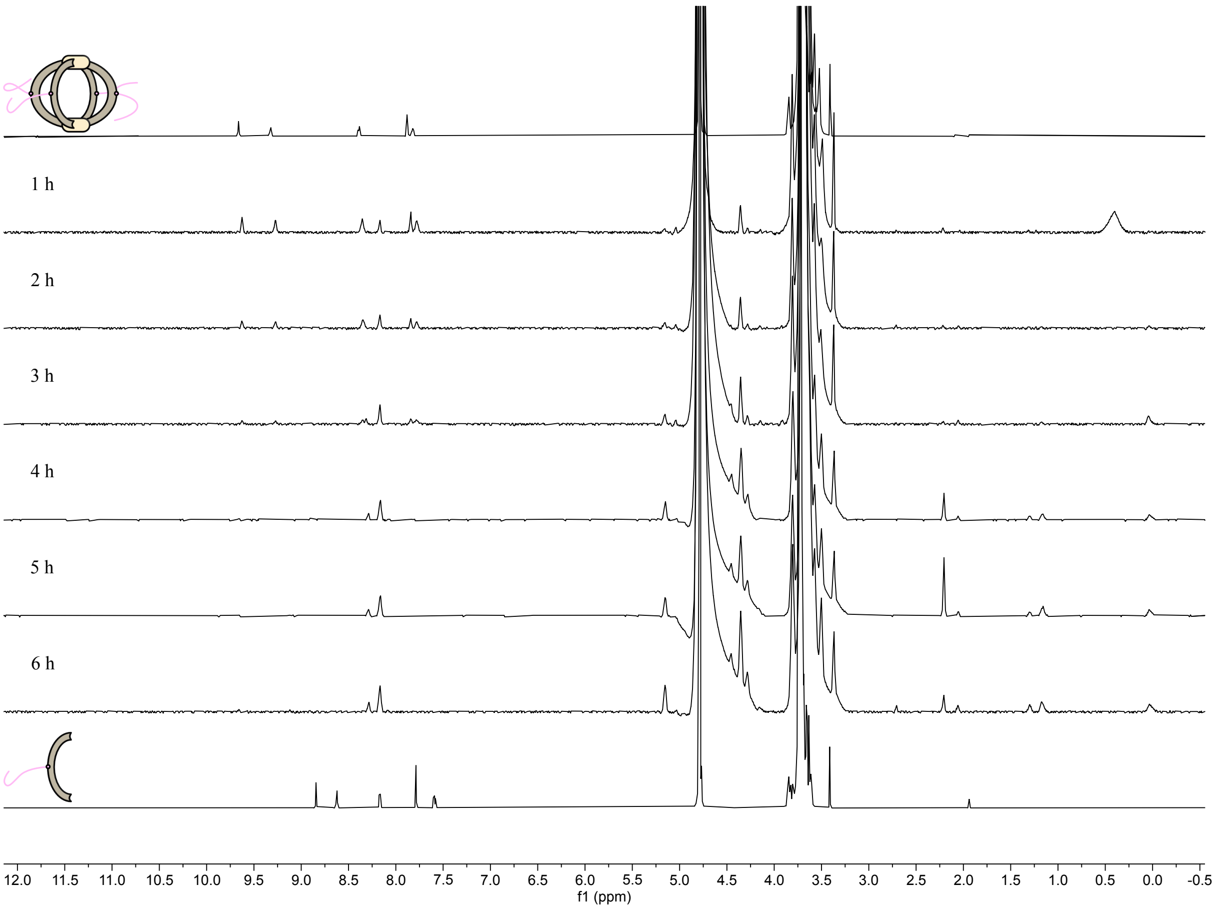

Figure S65: Stacked ^{1}H NMR spectra (600 MHz, D_2O, 298 K) showing the disassembly of **PolyMOC3** progressing over time. The spectra correspond to the initial complex (top), six sequential measurements recorded after each hour of sonication (middle), and **PolyL3** (bottom).

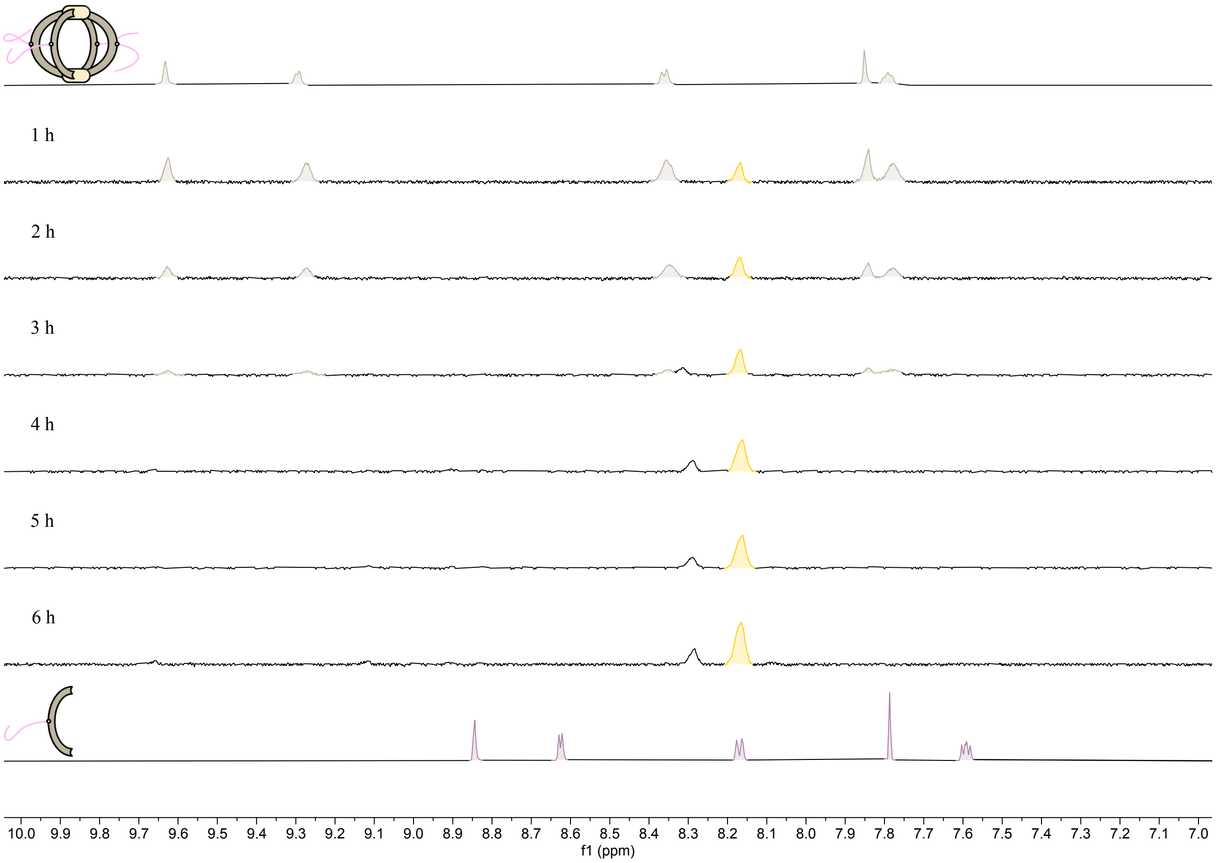

Figure S66: Enlarged view of stacked ^{1}H NMR spectra (600 MHz, D_2O, 298 K) showing the disassembly of **PolyMOC3** progressing over time. The spectra correspond to the initial complex (top), six sequential measurements recorded after each hour of sonication (middle), and **PolyL3** (bottom). The ^{1}H NMR signals are color-coded to indicate their respective species: **Pd_2L_4** (brown), **PolyL3** (purple) and formic acid (yellow) formed from PEG oxidation.

VII. Influence of chain length and concentration on PolyMOC activation rates

To investigate the differences in activation rates of **PolyMOC1-3** with varying chain lengths per **PolyL** (22 repeating units ≈1 kDa, 113 ≈ 5 kDa, 226 ≈ 10 kDa), we quantified the activation efficiency by determining the final product distribution of intact PolyMOC, Pd_2L_3 species and free PolyL after six hours of sonochemical treatment (Figure S67). The analysis was achieved by comparing the integrals of the proton resonances corresponding to H_b of each individual species and dividing the number of protons by the total sum of the corresponding proton resonances across the final mixture, thereby yielding the relative percentage of each product. Furthermore, we calculated the ratio of newly formed species per residual PolyMOC after six hours of sonochemical activation (Figure S68). For this purpose, the measured integrals were divided by the number of corresponding protons in the related species (6 H_b protons per Pd_2L_3 = 1 Pd_2L_3; 2 H_b protons per PolyL = 1 PolyL) and then compared against the normalized cage molecule (8 H_b protons per PolyMOC = 1 PolyMOC) (Tab. S1). To compare these rates, two concentration regimes were considered: (i) the polymer concentration in solution of 2.50 mg mL^{-1} (Figure S69-70) and (ii) the polymer concentration in solution of 5.00 mg mL^{-1} (Figure S71-72).

Table S2: Calculated cage activation after six hours of sonochemical activation in CD_3CN using concentrations of 2.50 mg mL^{-1} or 5.00 mg mL^{-1} expressed as newly formed discrete species per residual intact PolyMOC.

PolyMOC	Chain lenght per ligand (RU)	New species per cage (2.50 mg mL^{-1})	New species per cage (5.00 mg mL^{-1})
1	22	0	-
2	113	2.03	1.19
3	226	1.66	0.37

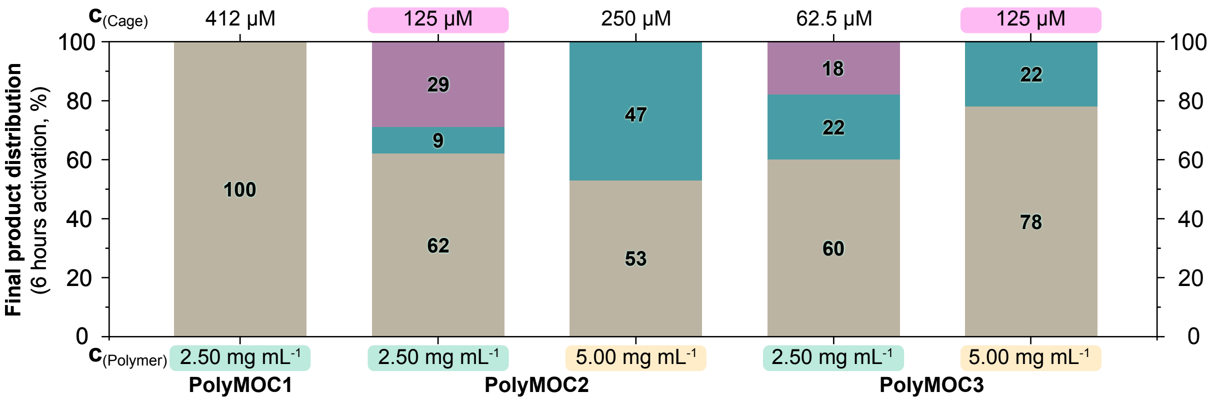

Figure S67: Final product distributions after 6 hours of sonochemical activation of **PolyMOC1-3** at the indicated polymer mass concentrations, shown as percentages of intact PolyMOC (brown), Pd_2L_3 species (turquoise), and free PolyL (purple).

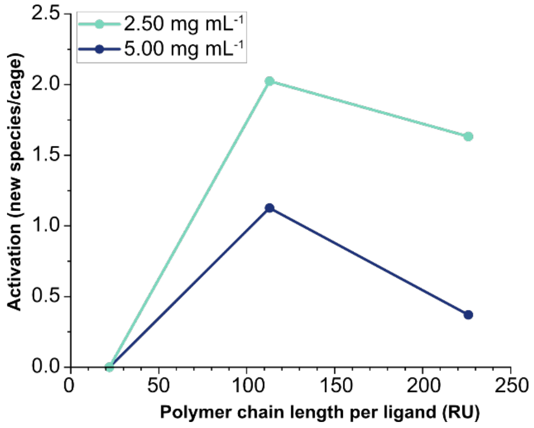

Figure S68: **PolyMOC** activation (newly formed species during sonication) against chain length (repeating units) at polymer concentration of 2.50 mg mL^{-1} (turquoise) and 5.00 mg mL^{-1} (blue) with corresponding polymer chain length per **PolyL**.

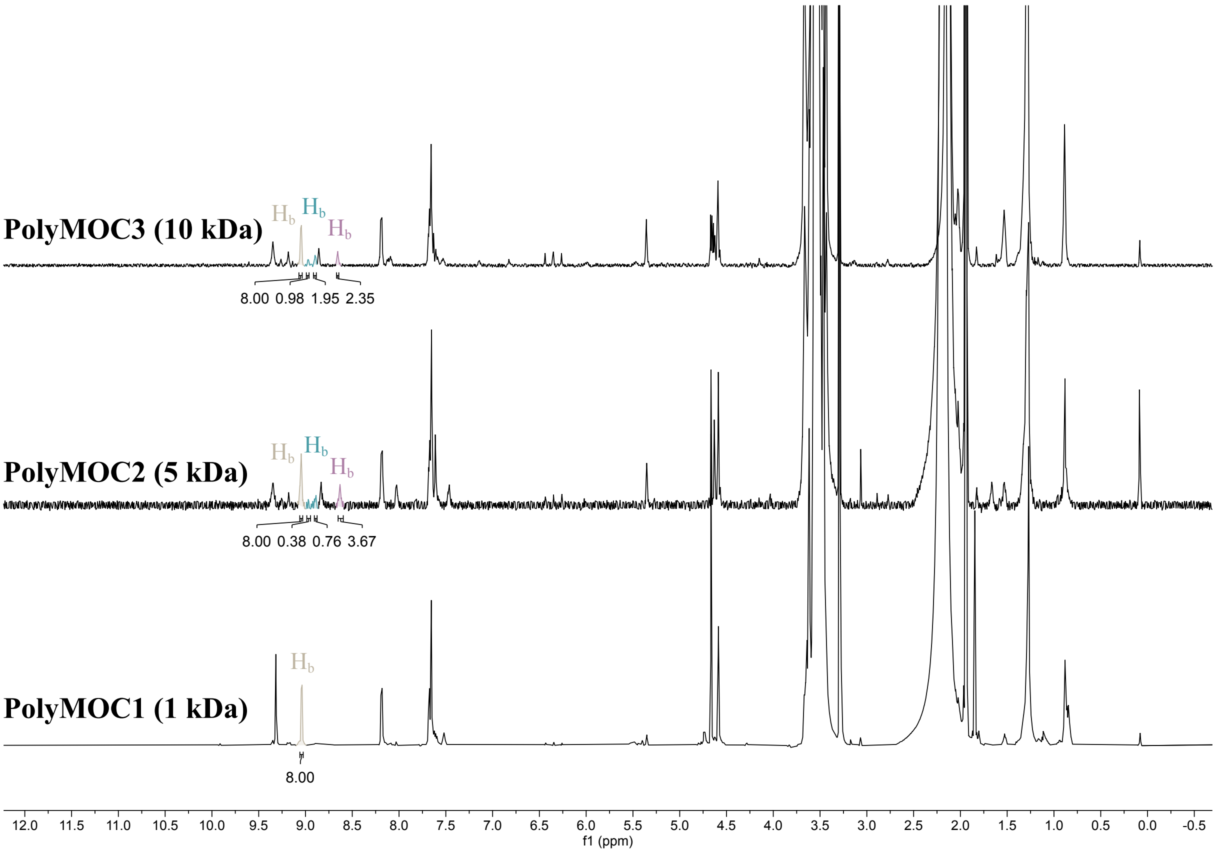

Figure S69: Stacked ^{1}H NMR spectra (600 MHz, CD_3CN, 298 K) of **PolyMOC1-3** after six hours of sonochemical activation in CD_3CN using a polymer concentration of 2.50 mg mL^{-1}. Signals corresponding to proton H_b of the respective species used for the calculation of the activation rates are color-coded to indicate their respective species: **Pd_2L_4** (brown), **Pd_2L_3** (blue), and **PolyL** (purple).

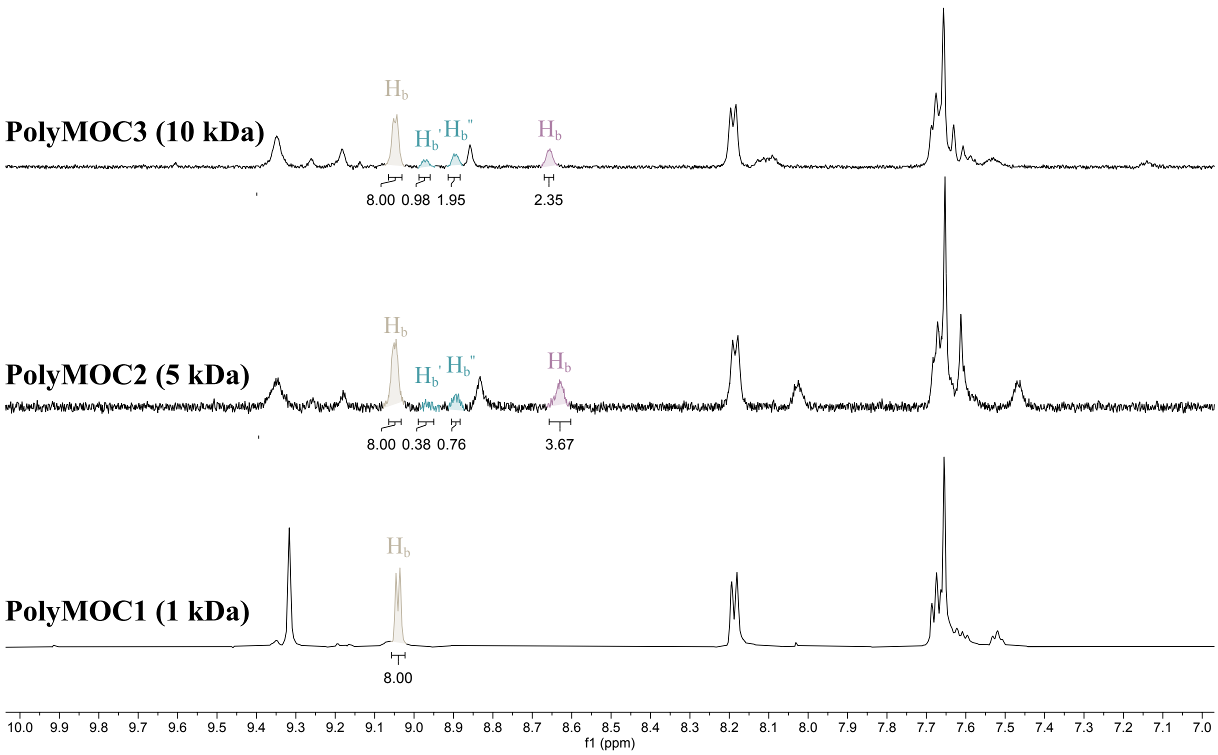

Figure S70: Enlarged view of stacked ^{1}H NMR spectra (600 MHz, CD_3CN, 298 K) of **PolyMOC1-3** after six hours of sonochemical activation in CD_3CN using a polymer concentration of 2.50 mg mL^{-1}. Signals corresponding to proton H_b of the respective species used for the calculation of the activation rates are color-coded to indicate their respective species: **Pd_2L_4** (brown), **Pd_2L_3** (blue), and **PolyL** (purple).

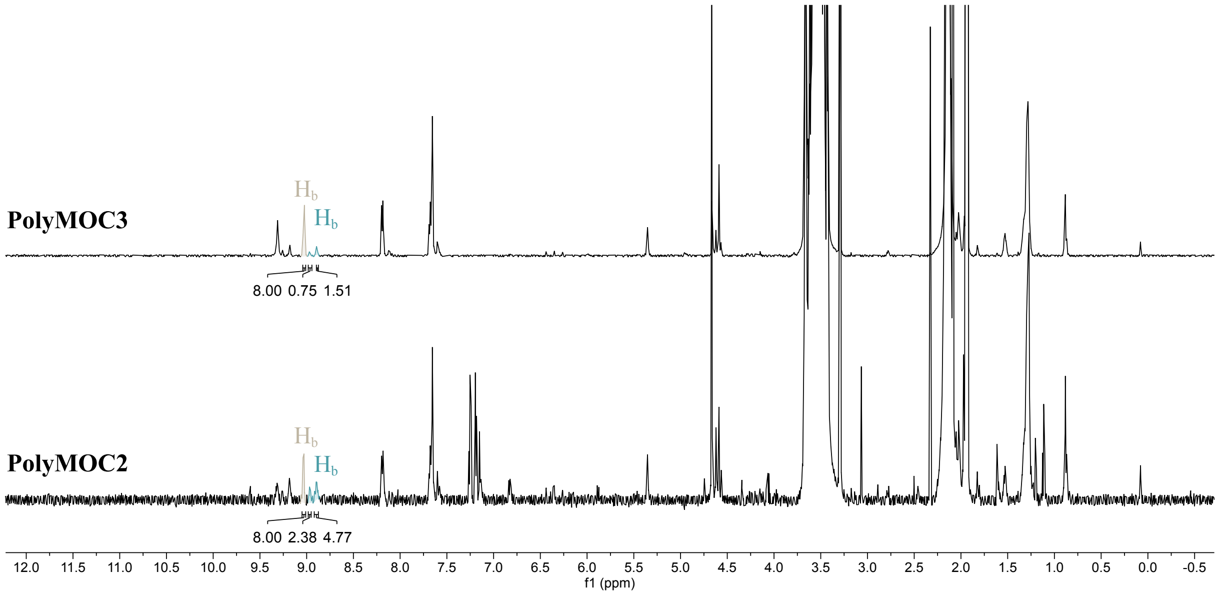

Figure S71: Stacked ^{1}H NMR spectra (600 MHz, CD_3CN, 298 K) of **PolyMOC1-3** after six hours of sonochemical activation in CD_3CN using a polymer concentration of 5.00 mg mL^{-1}. Signals corresponding to proton H_b of the respective species used for the calculation of the activation rates are color-coded to indicate their respective species: **Pd_2L_4** (brown), and **Pd_2L_3** (blue).

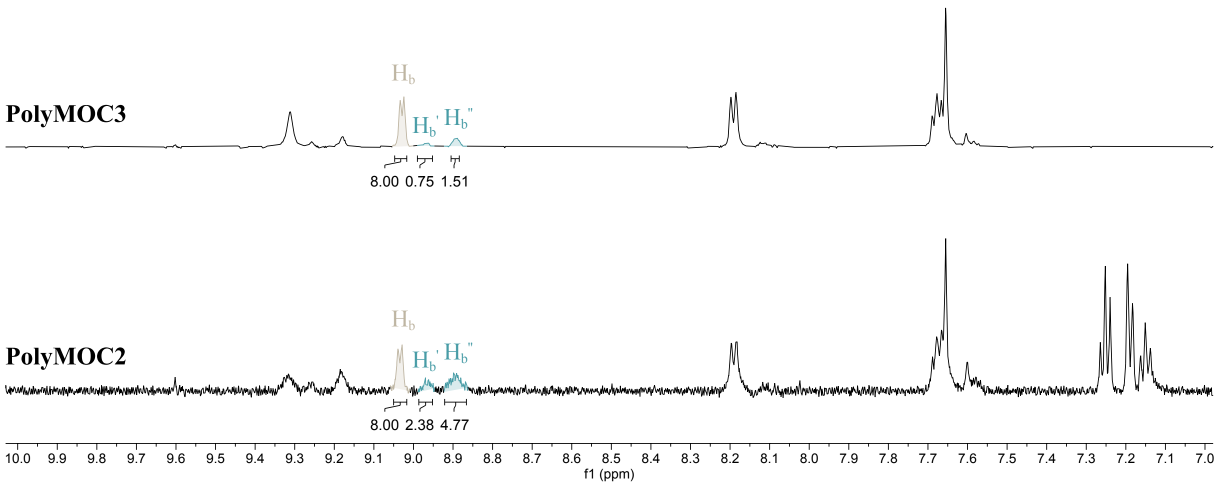

Figure S72: Enlarged view of stacked ^{1}H NMR spectra (600 MHz, CD_3CN, 298 K) of **PolyMOC1-3** after six hours of sonochemical activation in CD_3CN using a polymer concentration of 5.00 mg mL^{-1}. Signals corresponding to proton H_b of the respective species used for the calculation of the activation rates are color-coded to indicate their respective species: **Pd_2L_4** (brown), and **Pd_2L_3** (blue).

VIII. Reversibility studies of disassembled PolyMOCs

To assess whether the disassembled **PolyMOCs** can be reassembled, the freeze-dried samples obtained after sonication were analyzed in two separate experiments. In the first, a portion of the freeze-dried solid was redissolved at the same concentration in which it had been sonicated, and a ^{1}H NMR spectrum was recorded immediately. In the second, the remaining solid was redissolved at the concentration used for the initial synthesis of **PolyMOC3** (25.0 mM in CD_3CN), stirred for one hour, and subsequently analyzed by ^{1}H NMR spectroscopy.

Reversible reformation of the cages was only observed under conditions of higher concentration and when nitrogen was used as the saturation gas. Under these conditions, the activation led primarily to a $\mathbf{Pd_2L_4}$ species, with only a minor formation of $\mathbf{Pd_2L_3}$ and **PolyL3**. However, upon further activation, the process became irreversible, likely due to the reduction of the palladium(II) precursor under the sonication conditions, preventing the reformation of the original cage structure.

Sonication with nitrogen as saturation gas and 2.50 mg mL^{-1} concentration

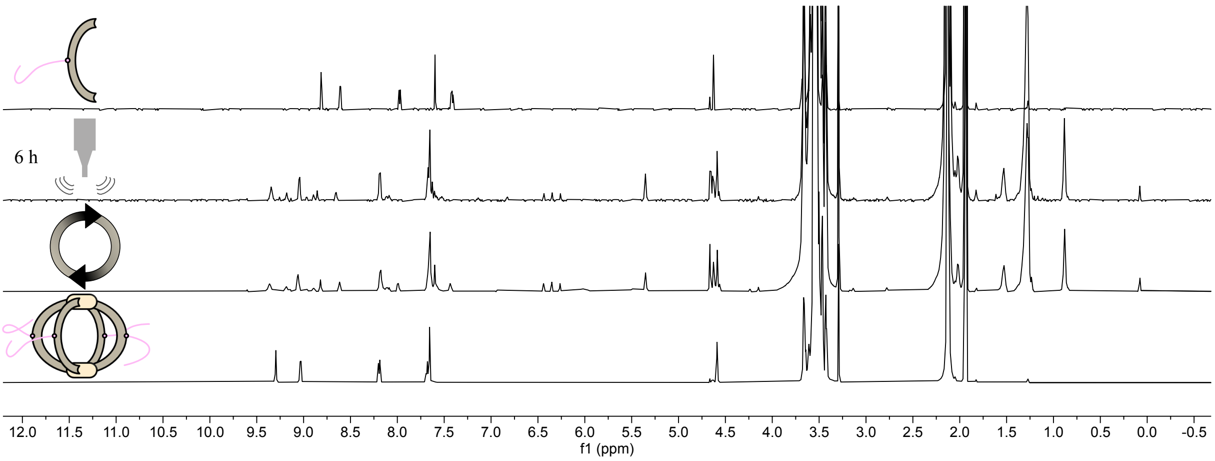

Figure S73: Stacked ^{1}H NMR spectra (600 MHz, CD_3CN, 298 K) showing the disassembly of **PolyMOC3** progressing over time. The spectra correspond to the **PolyL3** (top), product mixture after six hours of sonication (N_2, 2.50 mg mL^{-1}) (middle top), after stirring for one hour in CD_3CN (25 mM) (middle bottom), and **PolyMOC3** before sonication (bottom).

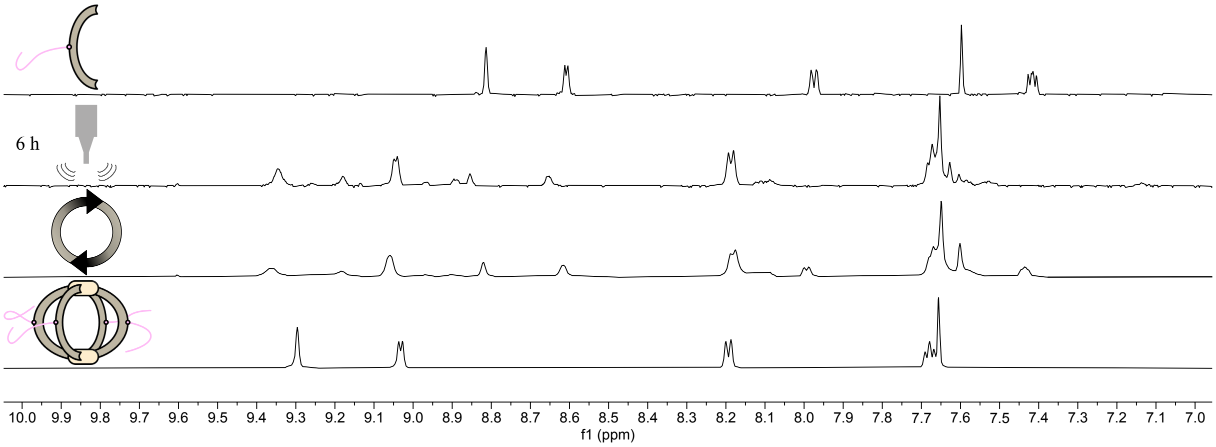

Figure S74: Enlarged view of stacked ^{1}H NMR spectra (600 MHz, CD_3CN, 298 K) showing the disassembly of **PolyMOC3** progressing over time. The spectra correspond to the **PolyL3** (top), product mixture after six hours of sonication (N_2, 2.50 mg mL^{-1}) (middle top), after stirring for one hour in CD_3CN (25 mM) (middle bottom), and **PolyMOC3** before sonication (bottom).

Sonication with nitrogen as saturation gas and 5.00 mg mL^{-1} concentration

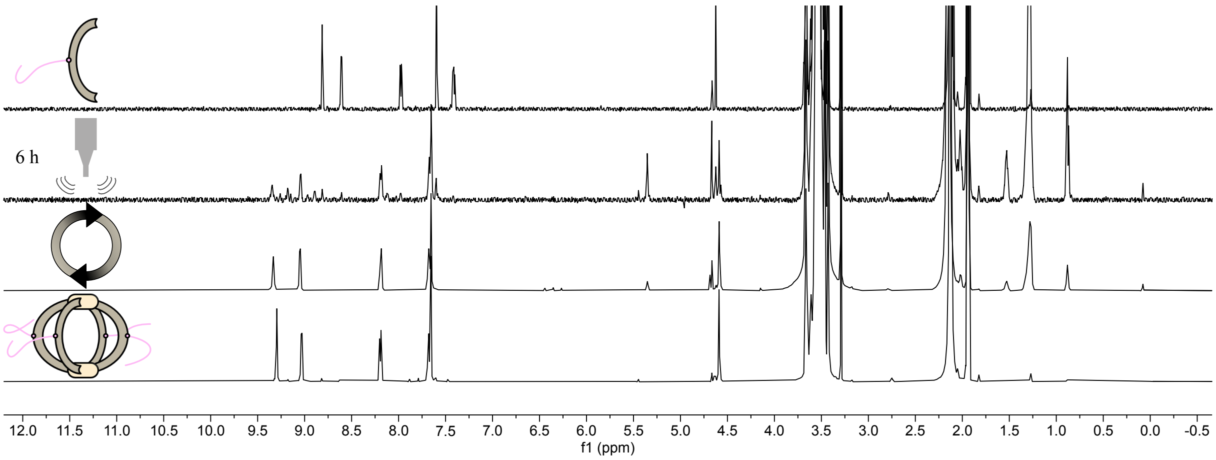

Figure S75: Stacked ^{1}H NMR spectra (600 MHz, CD_3CN, 298 K) showing the disassembly of **PolyMOC3** progressing over time. The spectra correspond to the **PolyL3** (top), product mixture after six hours of sonication (N_2, 5.00 mg mL^{-1}) (middle top), after stirring for one hour in CD_3CN (25 mM) (middle bottom), and **PolyMOC3** before sonication (bottom).

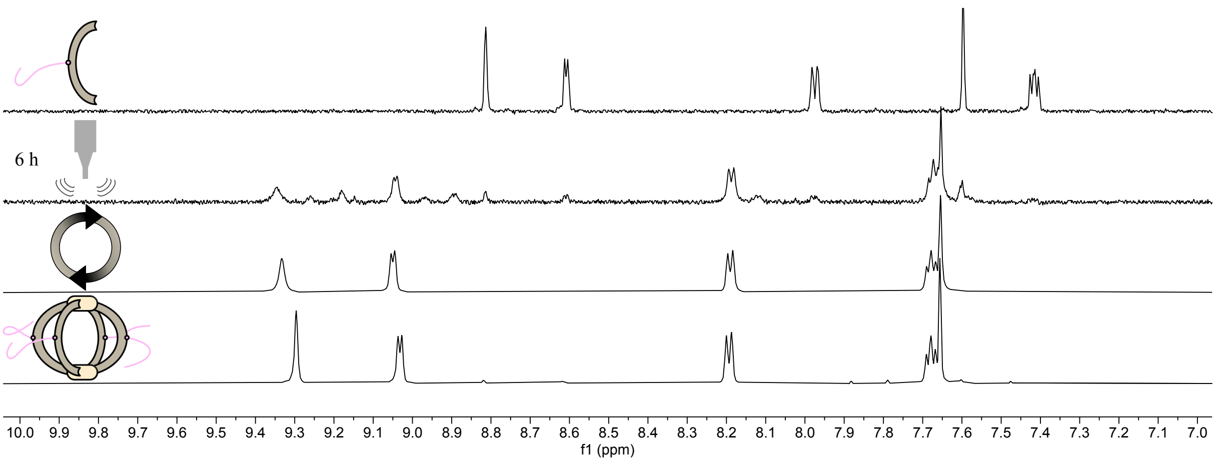

Figure S76: Enlarged view of stacked ^{1}H NMR spectra (600 MHz, CD_3CN, 298 K) showing the disassembly of **PolyMOC3** progressing over time. The spectra correspond to the **PolyL3** (top), product mixture after six hours of sonication (N_2, 5.00 mg mL^{-1}) (middle top), after stirring for one hour in CD_3CN (25 mM) (middle bottom), and **PolyMOC3** before sonication (bottom).

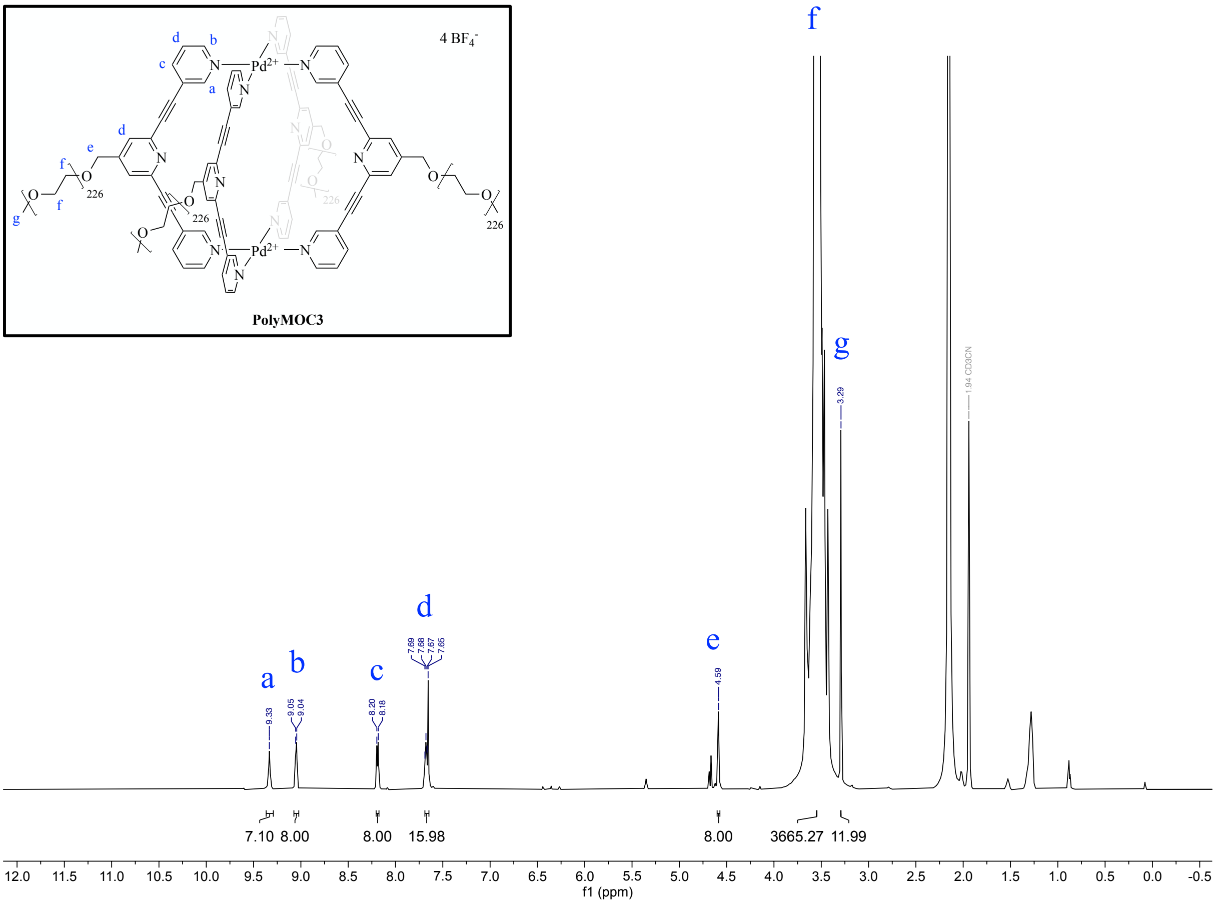

Figure S77: ^{1}H NMR spectrum (600 MHz, CD_3CN, 298 K) of reassembled **PolyMOC3**.

Sonication with argon as saturation gas and 2.50 mg mL^{-1} concentration

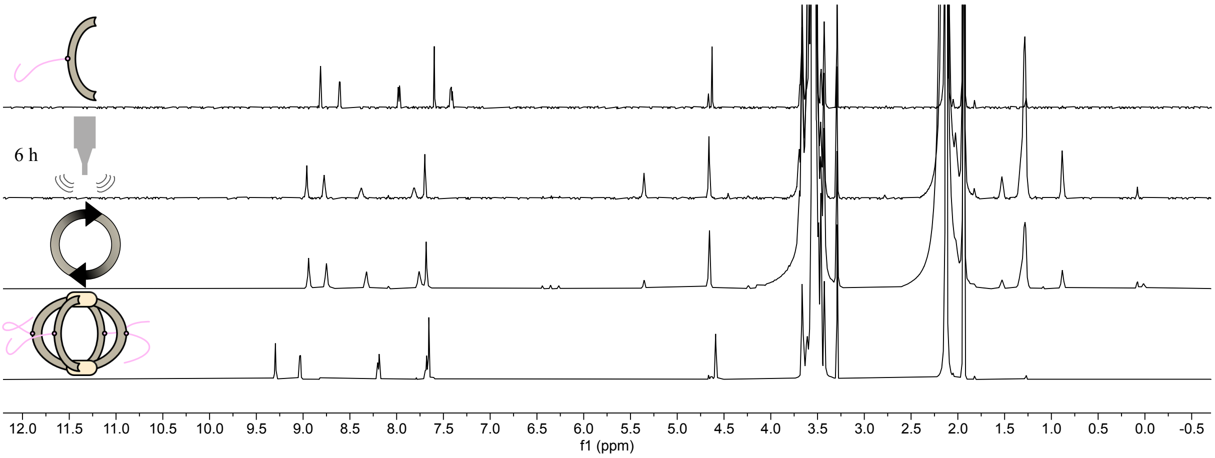

Figure S78: Stacked ^{1}H NMR spectra (600 MHz, CD_3CN, 298 K) showing the disassembly of **PolyMOC3** progressing over time. The spectra correspond to the **PolyL3** (top), product mixture after six hours of sonication (Ar, 2.50 mg mL^{-1}) (middle top), after stirring for one hour in CD_3CN (25 mM) (middle bottom), and **PolyMOC3** before sonication (bottom).

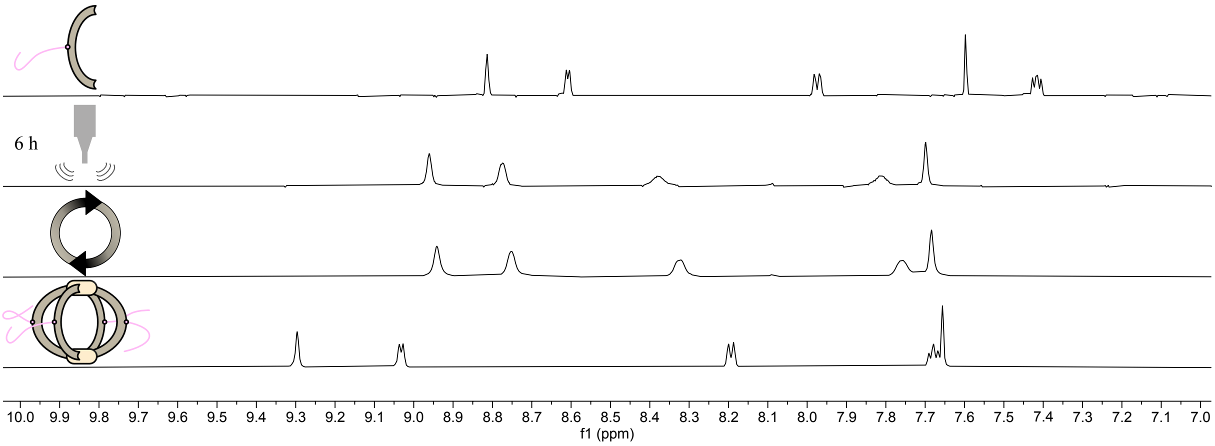

Figure S79: Enlarged view of stacked ^{1}H NMR spectra (600 MHz, CD_3CN, 298 K) showing the disassembly of **PolyMOC3** progressing over time. The spectra correspond to the **PolyL3** (top), product mixture after six hours of sonication (Ar, 2.50 mg mL^{-1}) (middle top), after stirring for one hour in CD_3CN (25 mM) (middle bottom), and **PolyMOC3** before sonication (bottom).

Sonication with argon as saturation gas and 5.00 mg mL^{-1} concentration

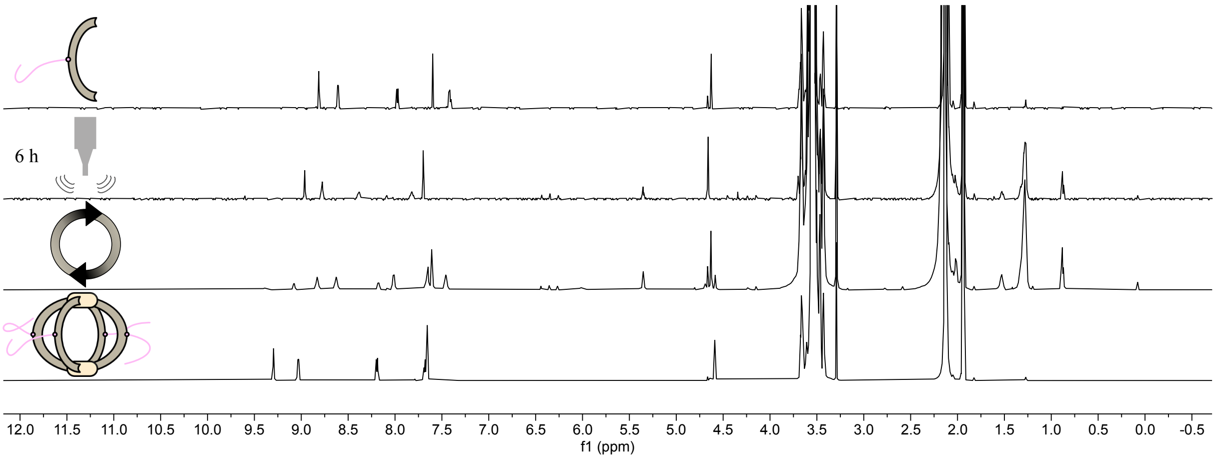

Figure S80: Stacked ^{1}H NMR spectra (600 MHz, CD_3CN, 298 K) showing the disassembly of **PolyMOC3** progressing over time. The spectra correspond to the **PolyL3** (top), product mixture after six hours of sonication (Ar, 5.00 mg mL^{-1}) (middle top), after stirring for one hour in CD_3CN (25 mM) (middle bottom), and **PolyMOC3** before sonication (bottom).

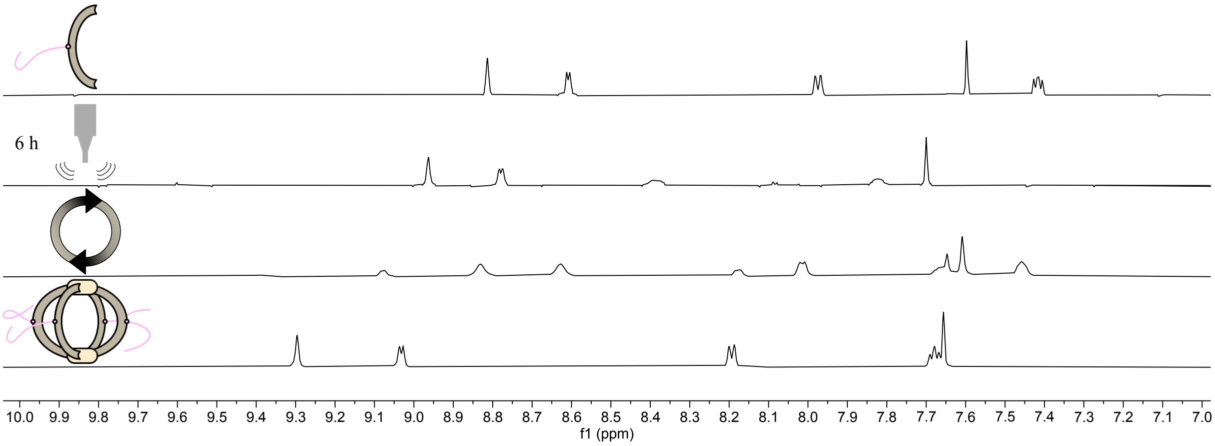

Figure S81: Enlarged view of stacked ^{1}H NMR spectra (600 MHz, CD_3CN, 298 K) showing the disassembly of **PolyMOC3** progressing over time. The spectra correspond to the **PolyL3** (top), product mixture after six hours of sonication (Ar, 5.00 mg mL^{-1}) (middle top), after stirring for one hour in CD_3CN (25 mM) (middle bottom), and **PolyMOC3** before sonication (bottom).

IX. Ultrasound-triggered cisplatin release from PolyMOC

For each cisplatin release experiment, cisplatin was encapsulated in the PolyMOCs, as described in the experimental details. The **PolyMOC⊃(cisplatin)₂** solution in CD_3CN was transferred into a nitrogen- or argon-purged Suslick vessel and diluted with dry or deuterated acetonitrile to obtain final concentrations of 2.50 mg mL^{-1} (**PolyMOC2** and **PolyMOC3**) or 5.00 mg mL^{-1} (**PolyMOC4**). The sonication mixture was then cooled to 0 °C in an ice-water bath and degassed with nitrogen or argon for ten minutes before sonication. Sonication was performed for six hours (three hours effective time) using a 1 s on / 1 s off pulse sequence at 30 % amplitude, while the reaction mixture was continuously bubbled with the respective saturation gas.

Table S3: Summary of cisplatin release experiments and corresponding conditions.

PolyMOC	Solvent	Polymer Chain Length (RU)	Concentration (mg mL^{-1})	Saturation Gas	Result/Discription
2	CD_3CN	113	2.50	Nitrogen	Cisplatin release 0.5h, complete PolyMOC dissociation 5h
2	CD_3CN	113	2.50	Argon	Cisplatin release and complete PolyMOC dissociation 0.5h
3	CD_3CN	226	2.50	Nitrogen	Cisplatin release 0.5h, complete PolyMOC dissociation 4h
3	CD_3CN	226	2.50	Argon	Cisplatin release and complete PolyMOC dissociation 0.5h
4	CD_3CN	453	5.00	Nitrogen	Cisplatin released
4	CD_3CN	453	5.00	Argon	Cisplatin released

Cisplatin release from PolyMOC2⊃(cisplatin)₂

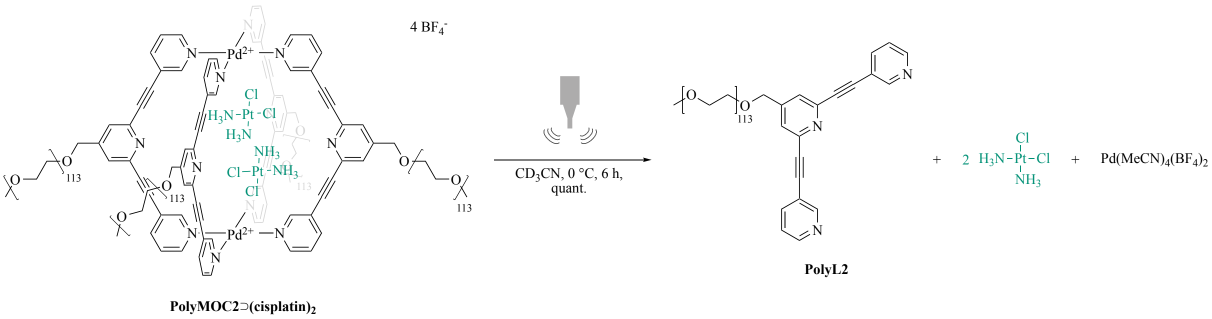

Figure S82: Reaction equation for the release of cisplatin from **PolyMOC2⊃(cisplatin)₂**.

Nitrogen as saturation gas

The release experiment, using nitrogen as the saturation gas, was conducted in CD_3CN. Before and after each hour of sonication, a ^1H NMR spectrum was recorded to monitor the release of cisplatin and the disassembly of the cage.

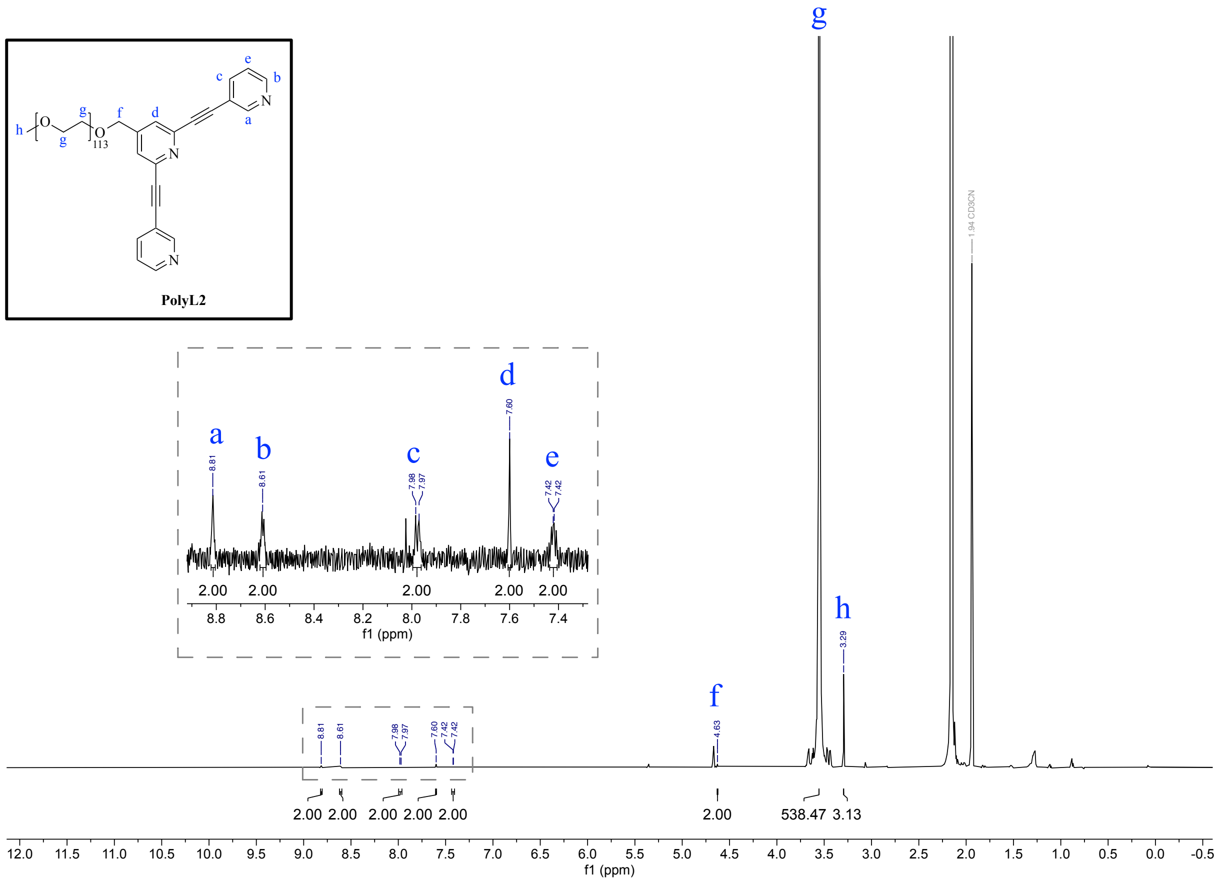

Figure S83: ^{1}H NMR spectrum (600 MHz, CD_3CN, 298 K) of **PolyMOC2⊃(cisplatin)₂** after six hours of sonication (N_2).

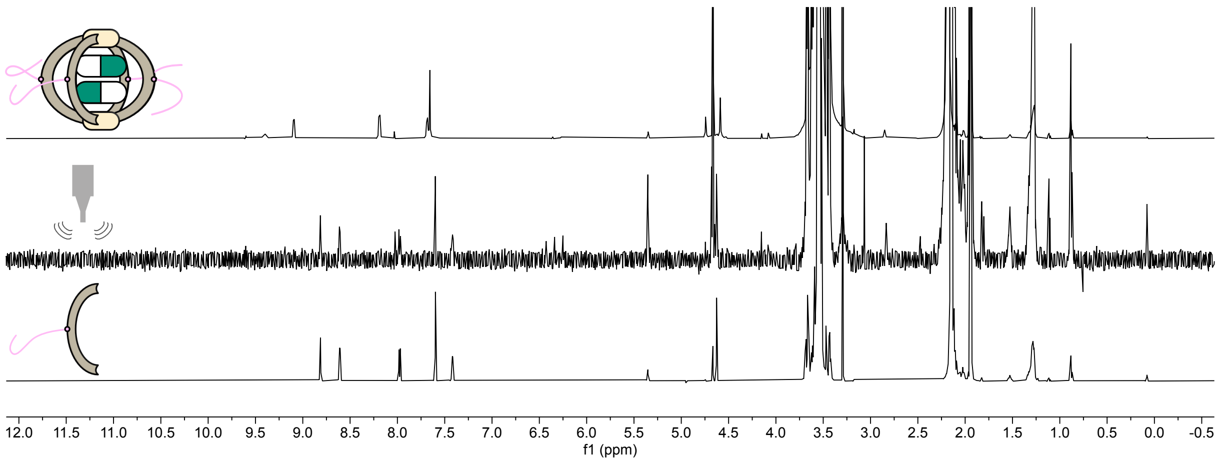

Figure S84: Stacked ^{1}H NMR spectra (600 MHz, CD_3CN, 298 K) of **PolyMOC2⊃(cisplatin)$_2$** (top), after six hours of sonication (N_2) (middle), and **PolyL2** (bottom).

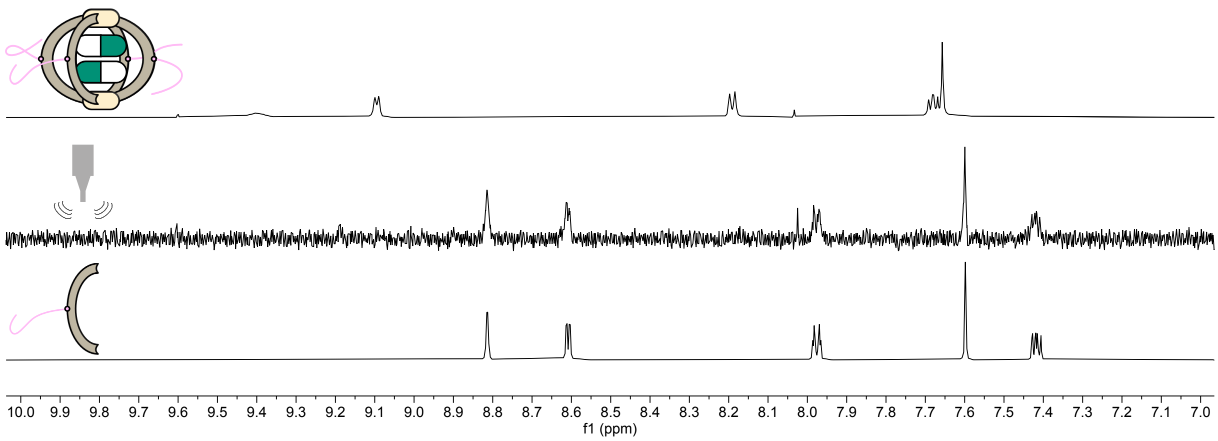

Figure S85: Enlarged view of stacked ^{1}H NMR spectra (600 MHz, CD_3CN, 298 K) of **PolyMOC2⊃(cisplatin)₂** (top), after six hours of sonication (N_2) (middle), and **PolyL2** (bottom).

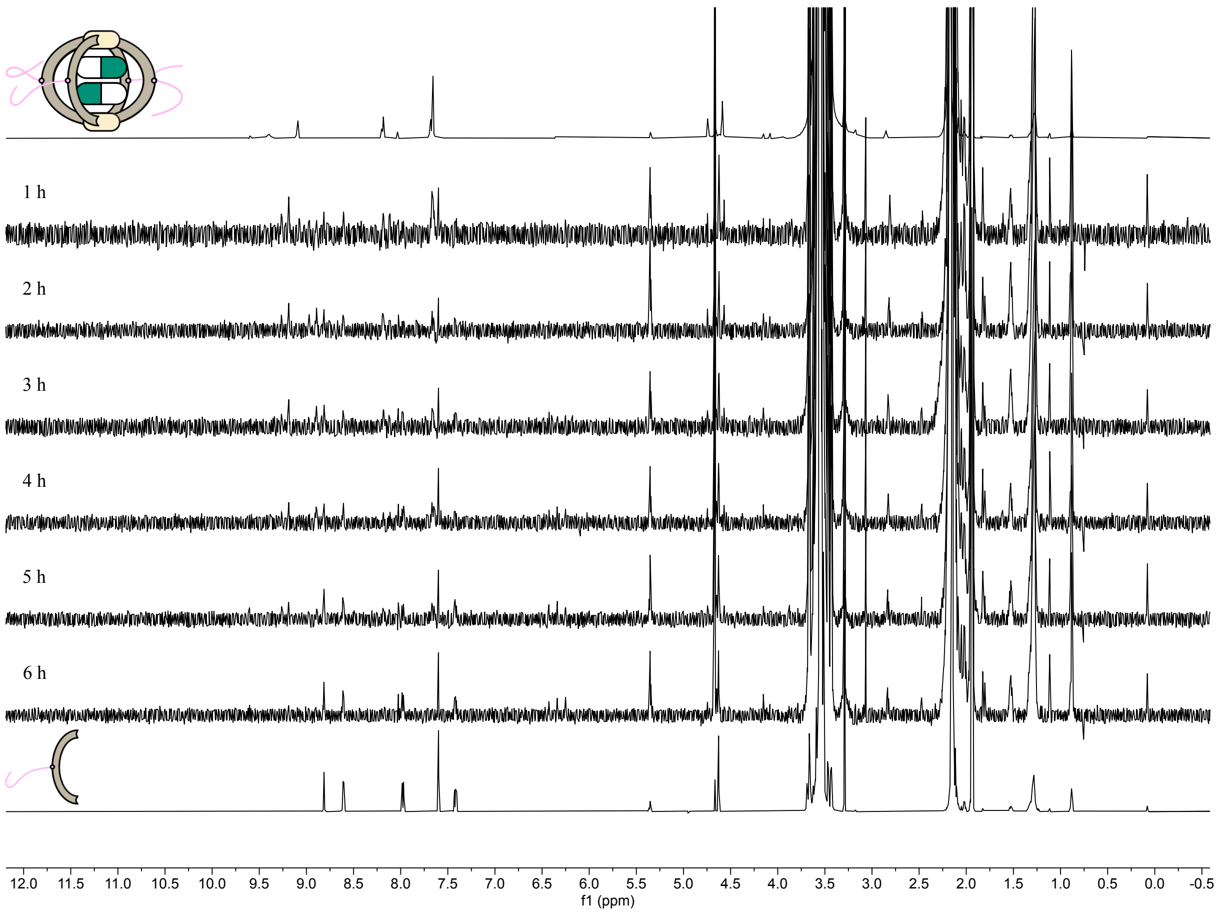

Figure S86: Stacked ^{1}H NMR spectra (600 MHz, CD_3CN, 298 K) showing the release progression of **PolyMOC2⊃(cisplatin)₂** over time. The spectra correspond to the initial complex (top), six sequential measurements recorded after each hour of sonication (middle), and **PolyL2** (bottom).

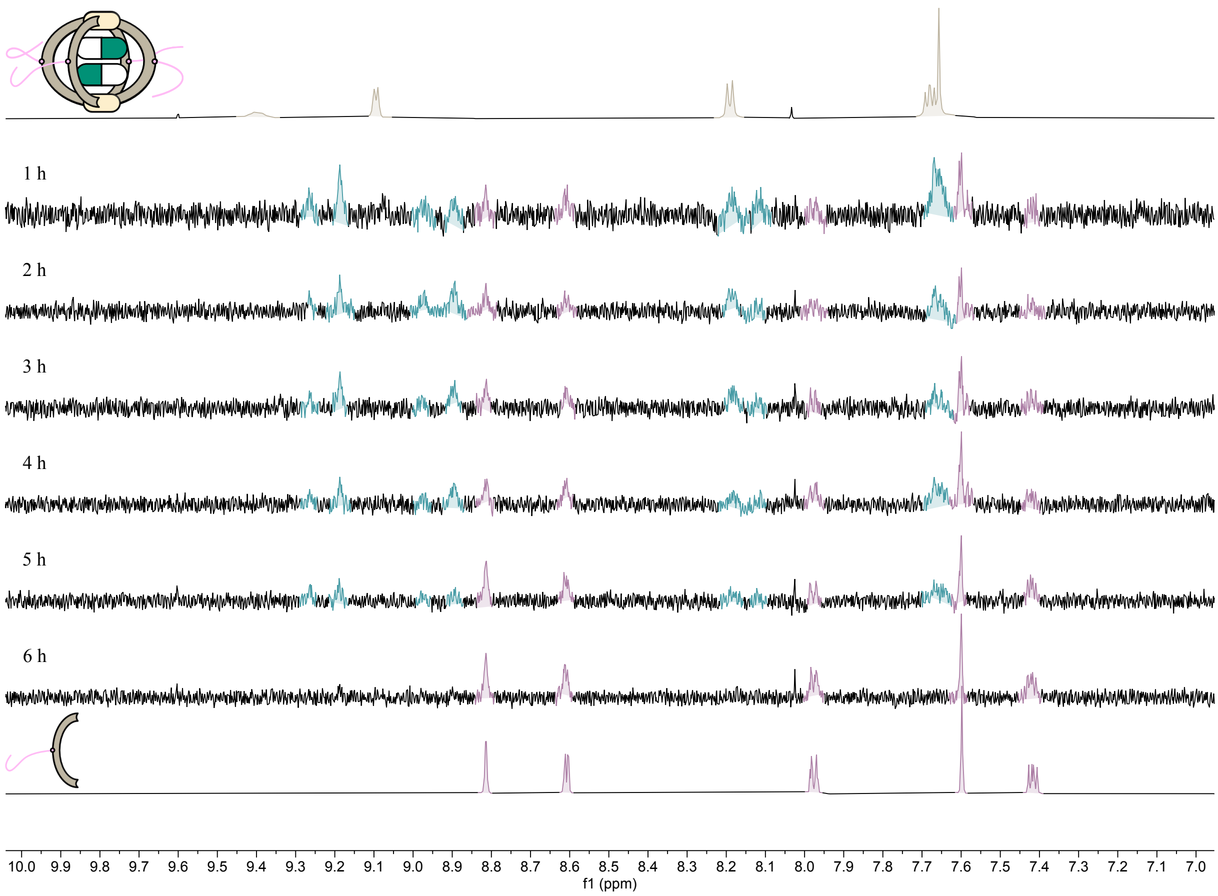

Figure S87: Enlarged view of stacked ^{1}H NMR spectra (600 MHz, CD_3CN, 298 K) showing the release progression of **PolyMOC2⊃(cisplatin)₂** over time. The spectra correspond to the initial complex (top), six sequential measurements recorded after each hour of sonication (middle), and **PolyL2** (bottom). The ^{1}H NMR signals are color-coded to indicate their respective species: **Pd_2L_4** (brown), **Pd_2L_3** (blue), and **PolyL2** (purple).

Argon as saturation gas

The release experiment, using argon as the saturation gas, was conducted in CD_3CN. Before and after each hour of sonication, a ^{1}H NMR spectrum was recorded to monitor the release of cisplatin and the disassembly of the cage. After just one hour of sonication, the cage was completely disassembled, and the cisplatin was fully released. Over the following five hours, only byproducts from the degradation of the PEG polymer chain and the ligand itself were observed. To confirm this unexpected result, the experiment was repeated twice, yielding the same outcome.

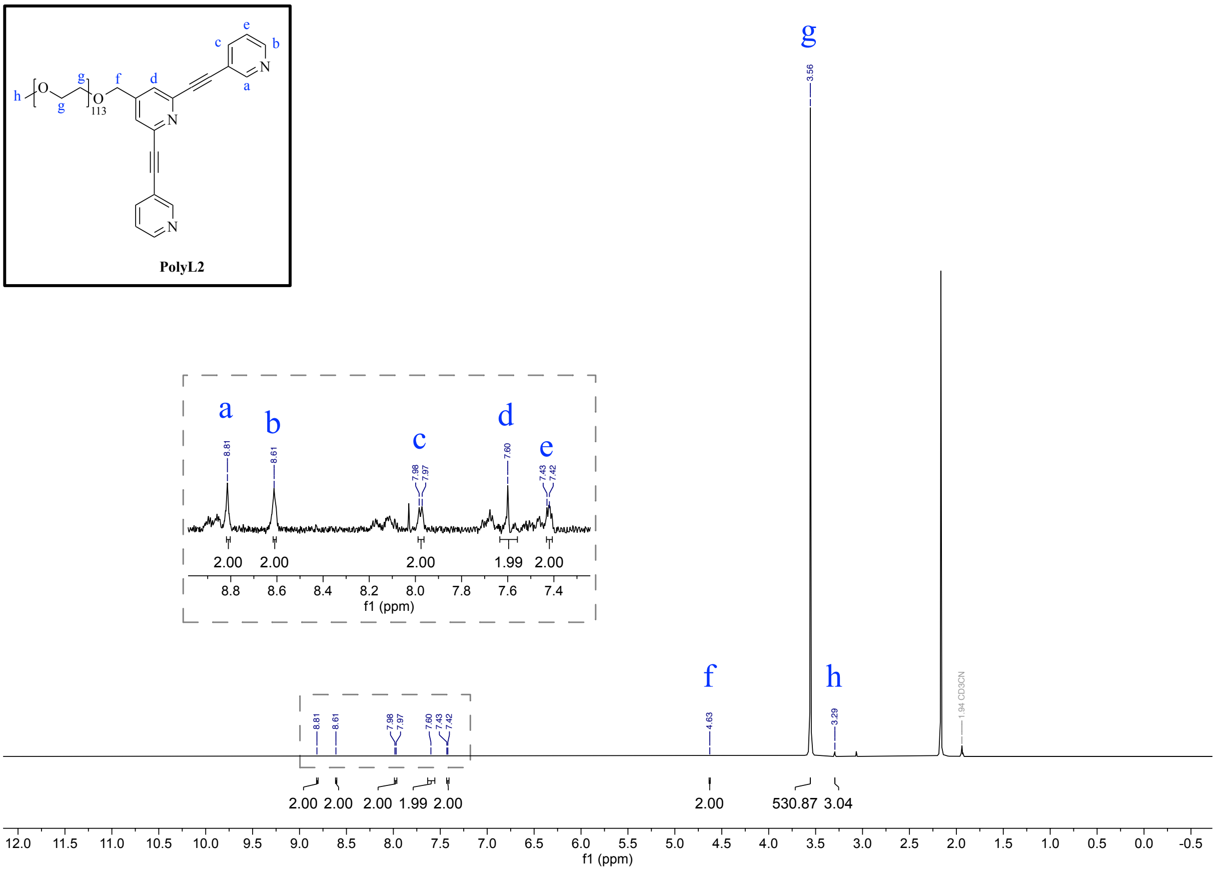

Figure S88: ^{1}H NMR spectrum (600 MHz, CD_3CN, 298 K) of **PolyMOC2⊃(cisplatin)₂** after six hours of sonication (Ar).

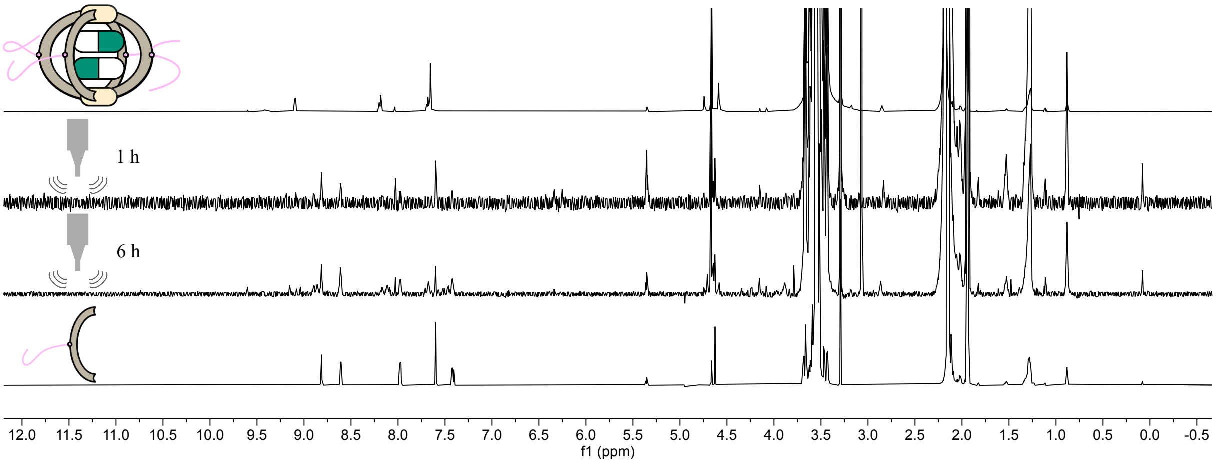

Figure S89: Stacked ^{1}H NMR spectra (600 MHz, CD_3CN, 298 K) of **PolyMOC2⊃(cisplatin)₂** (top), after one and after six hours of sonication (Ar) (middle), and **PolyL2** (bottom).

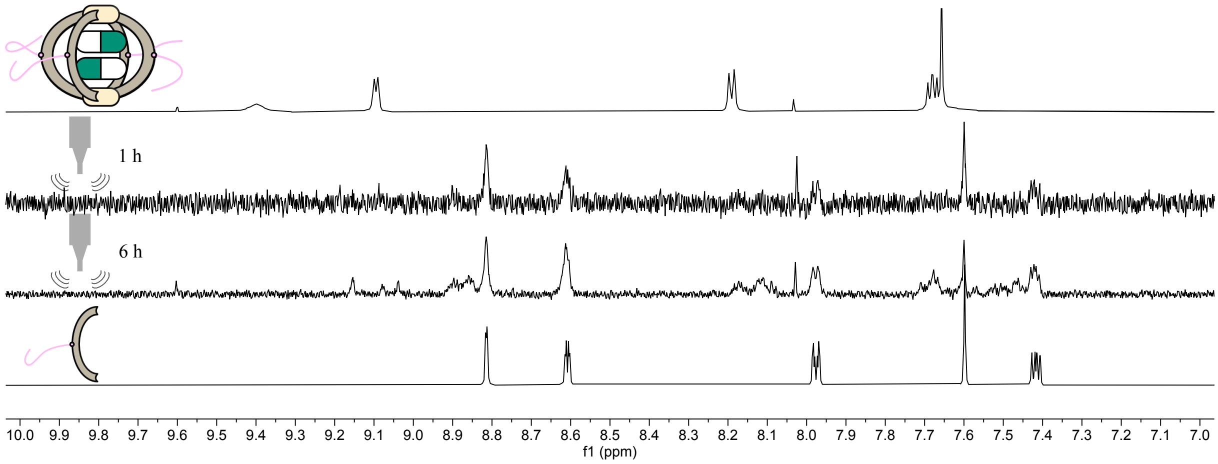

Figure S90: Enlarged view of stacked ^{1}H NMR spectra (600 MHz, CD_3CN, 298 K) of **PolyMOC2⊃(cisplatin)₂** (top), after one and after six hours of sonication (Ar) (middle), and **PolyL2** (bottom).

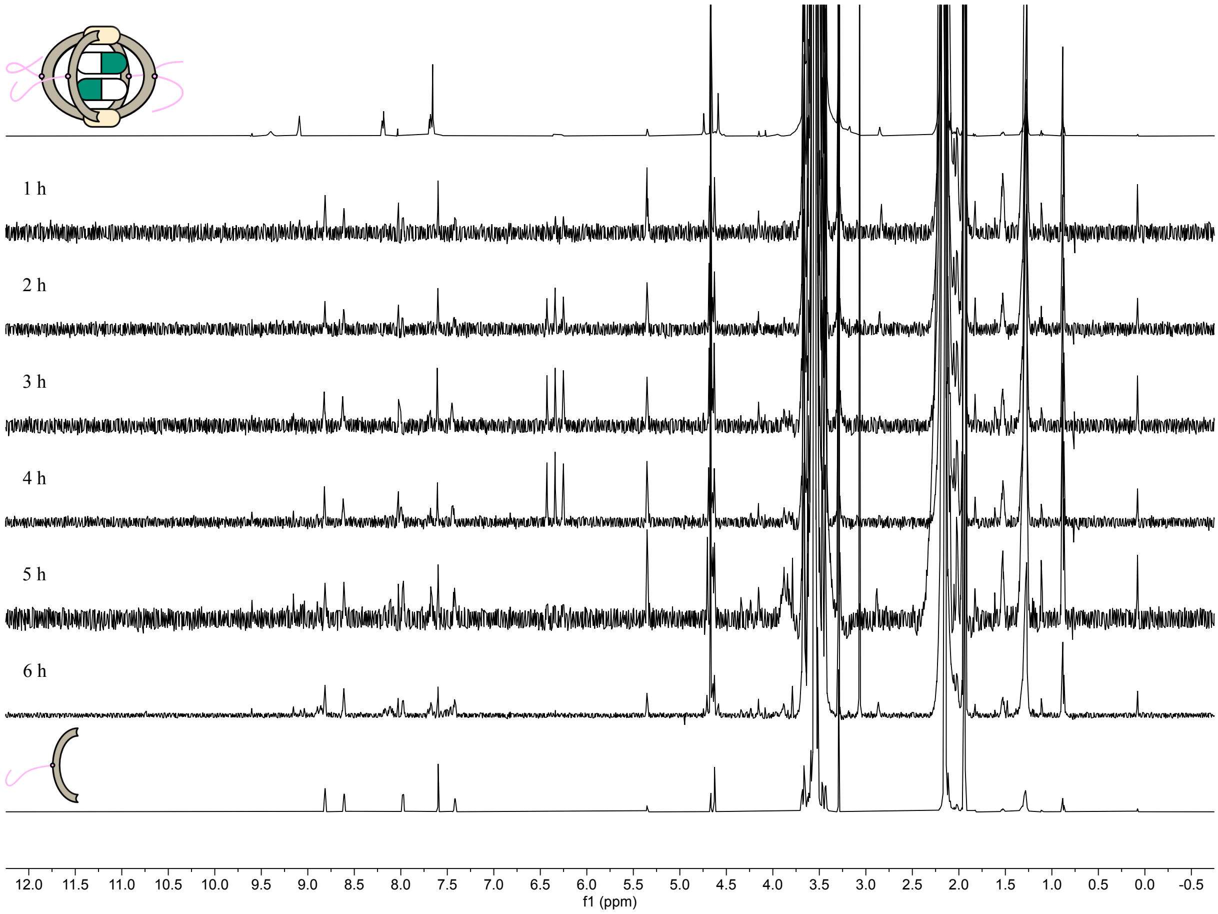

Figure S91: Stacked ^{1}H NMR spectra (600 MHz, CD_3CN, 298 K) showing the release progression of **PolyMOC2⊃(cisplatin)₂** over time. The spectra correspond to the initial complex (top), six sequential measurements recorded after each hour of sonication (middle), and **PolyL2** (bottom).

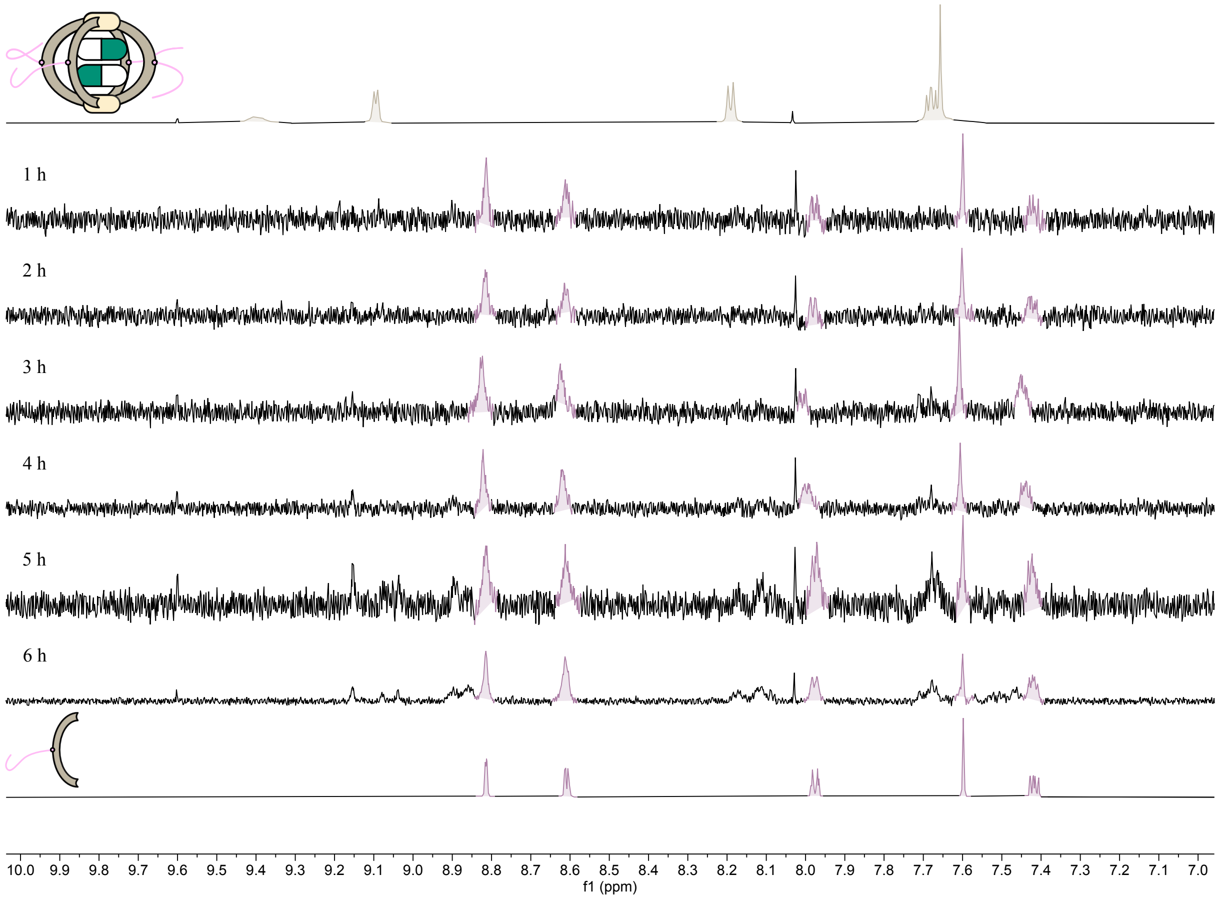

Figure S92: Enlarged view of stacked ^{1}H NMR spectra (600 MHz, CD_3CN, 298 K) showing the release progression of **PolyMOC2⊃(cisplatin)₂** over time. The spectra correspond to the initial complex (top), six sequential measurements recorded after each hour of sonication (middle), and **PolyL2** (bottom).

Cisplatin release from PolyMOC3⊃(cisplatin)₂

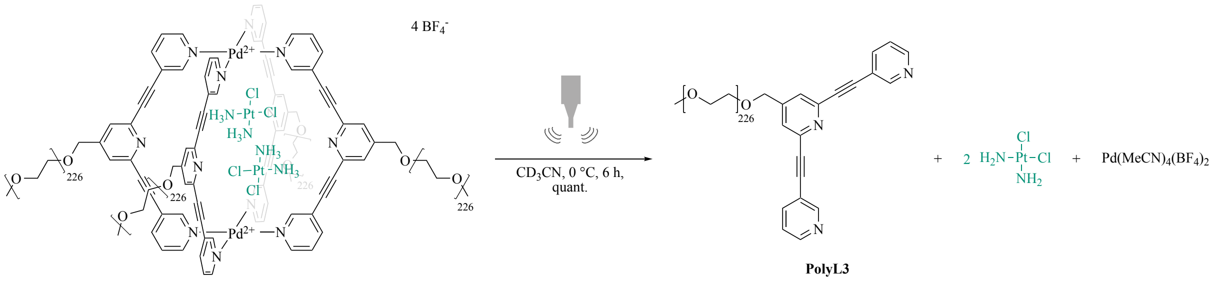

PolyMOC3⊃(cisplatin)₂

Scheme S93: Reaction equation for the release of cisplatin from **PolyMOC3⊃(cisplatin)₂**.

Nitrogen as saturation gas

The release experiment, using nitrogen as the saturation gas, was conducted in CD_3CN. Before sonication and after each hour of sonication, a ^{1}H NMR spectrum was recorded to monitor the release of cisplatin and the disassembly of the cage.

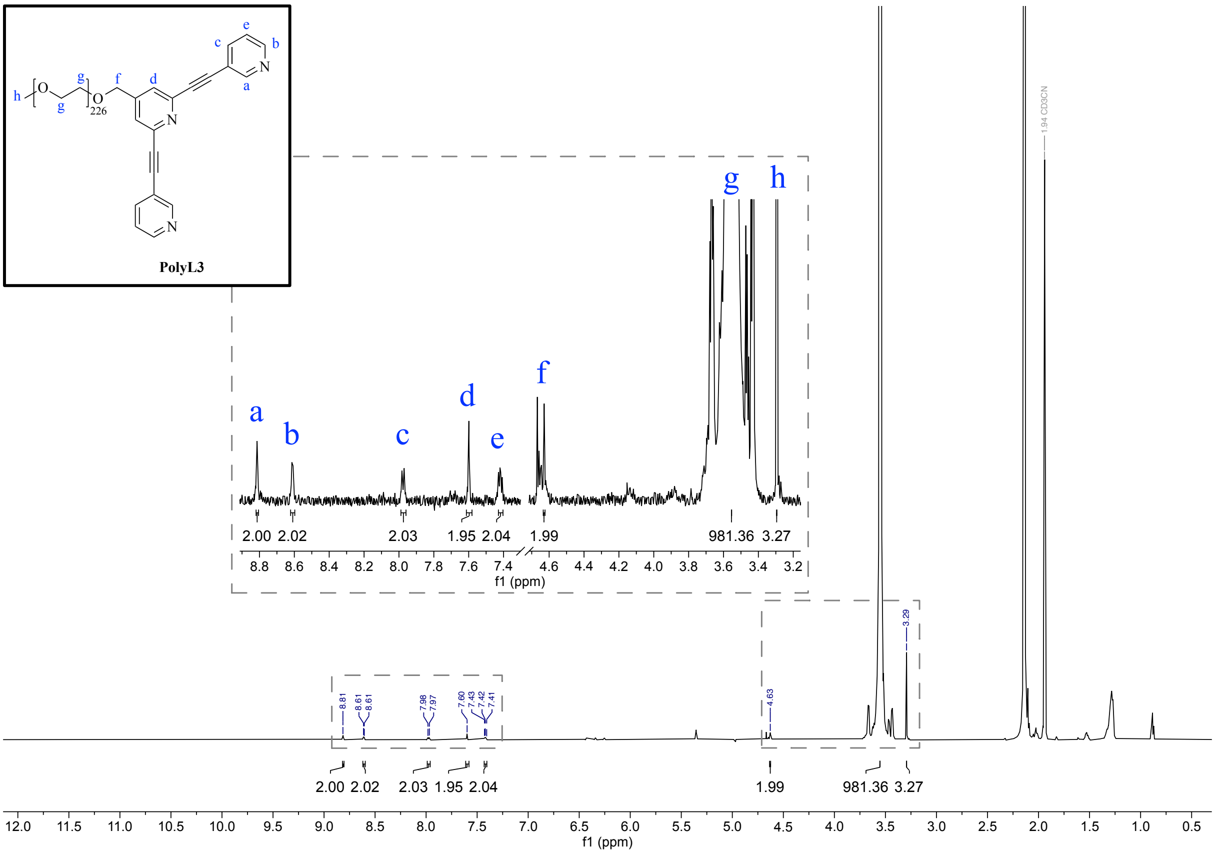

Figure S94: ^{1}H NMR spectrum (600 MHz, CD_3CN, 298 K) of **PolyMOC3⊃(cisplatin)$_2$** after six hours of sonication (N_2).

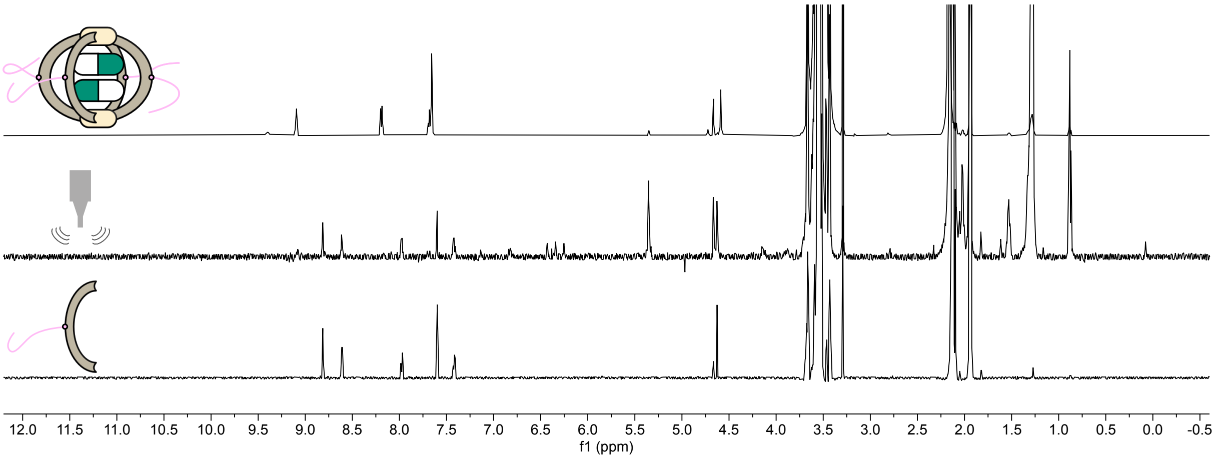

Figure S95: Stacked ^{1}H NMR spectra (600 MHz, CD_3CN, 298 K) of **PolyMOC3⊃(cisplatin)$_2$** (top), after six hours of sonication (N_2) (middle), and **PolyL3** (bottom).

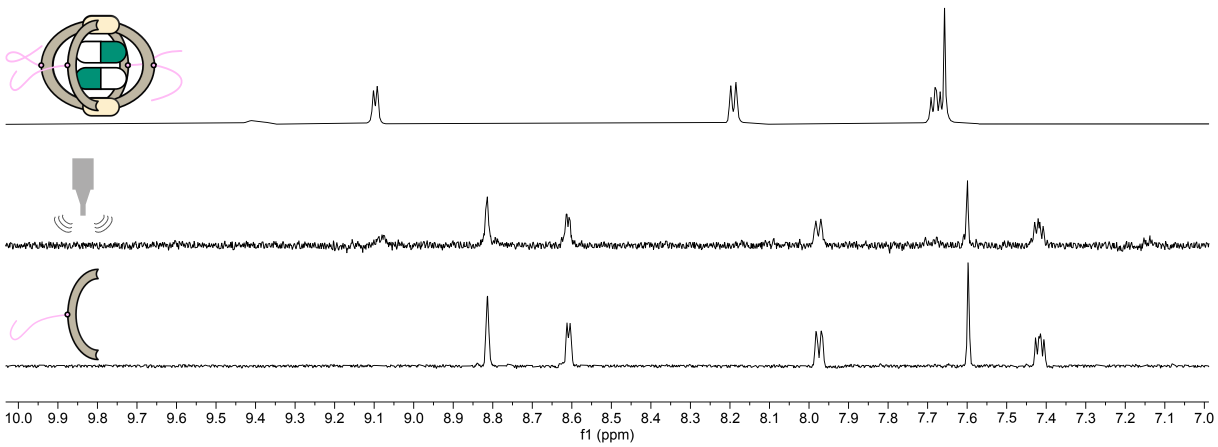

Figure S96: Enlarged view of stacked ^{1}H NMR spectra (600 MHz, CD_3CN, 298 K) of **PolyMOC3⊃(cisplatin)₂** (top), after six hours of sonication (N_2) (middle), and **PolyL3** (bottom).

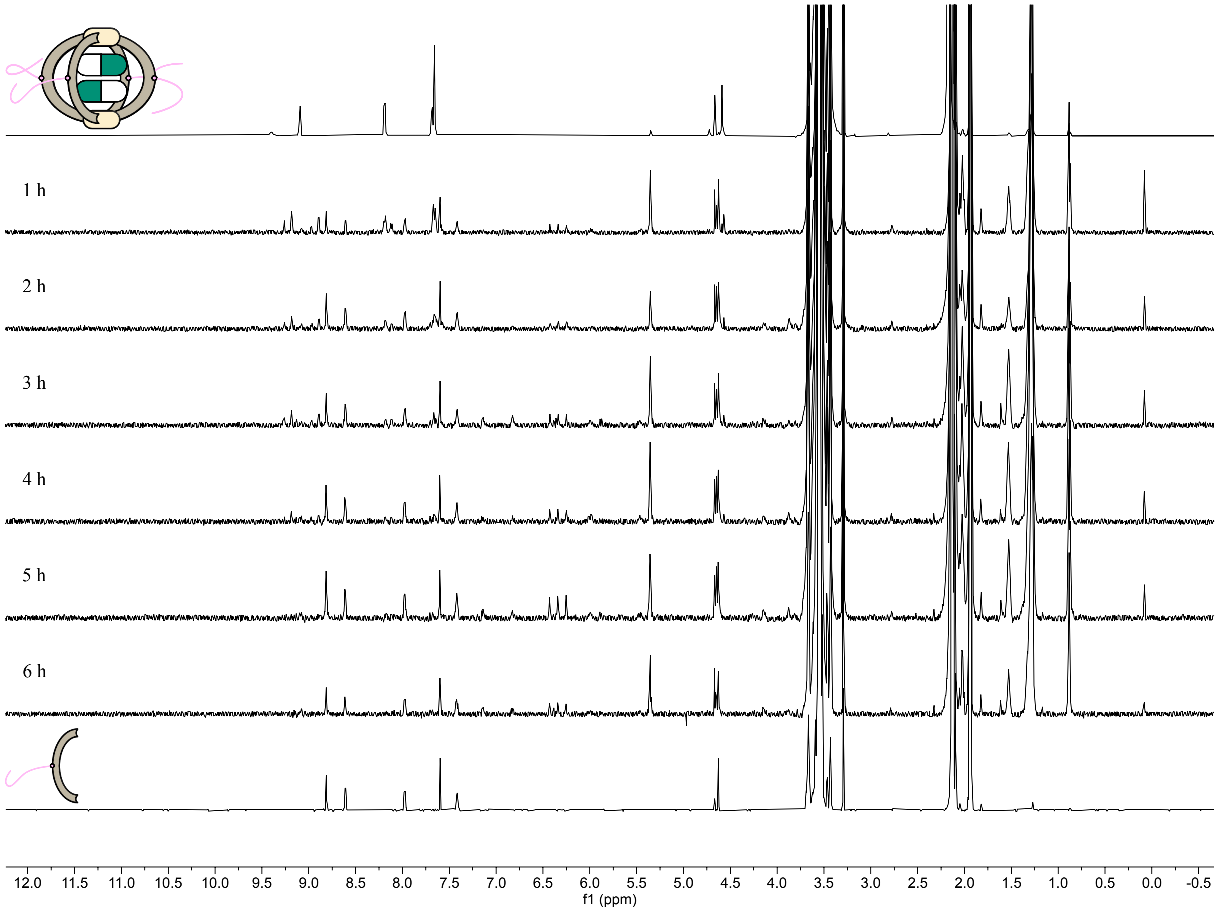

Figure S97: Stacked ^{1}H NMR spectra (600 MHz, CD_3CN, 298 K) showing the release progression of **PolyMOC3⊃(cisplatin)₂** over time. The spectra correspond to the initial complex (top), six sequential measurements recorded after each hour of sonication (middle), and **PolyL3** (bottom).

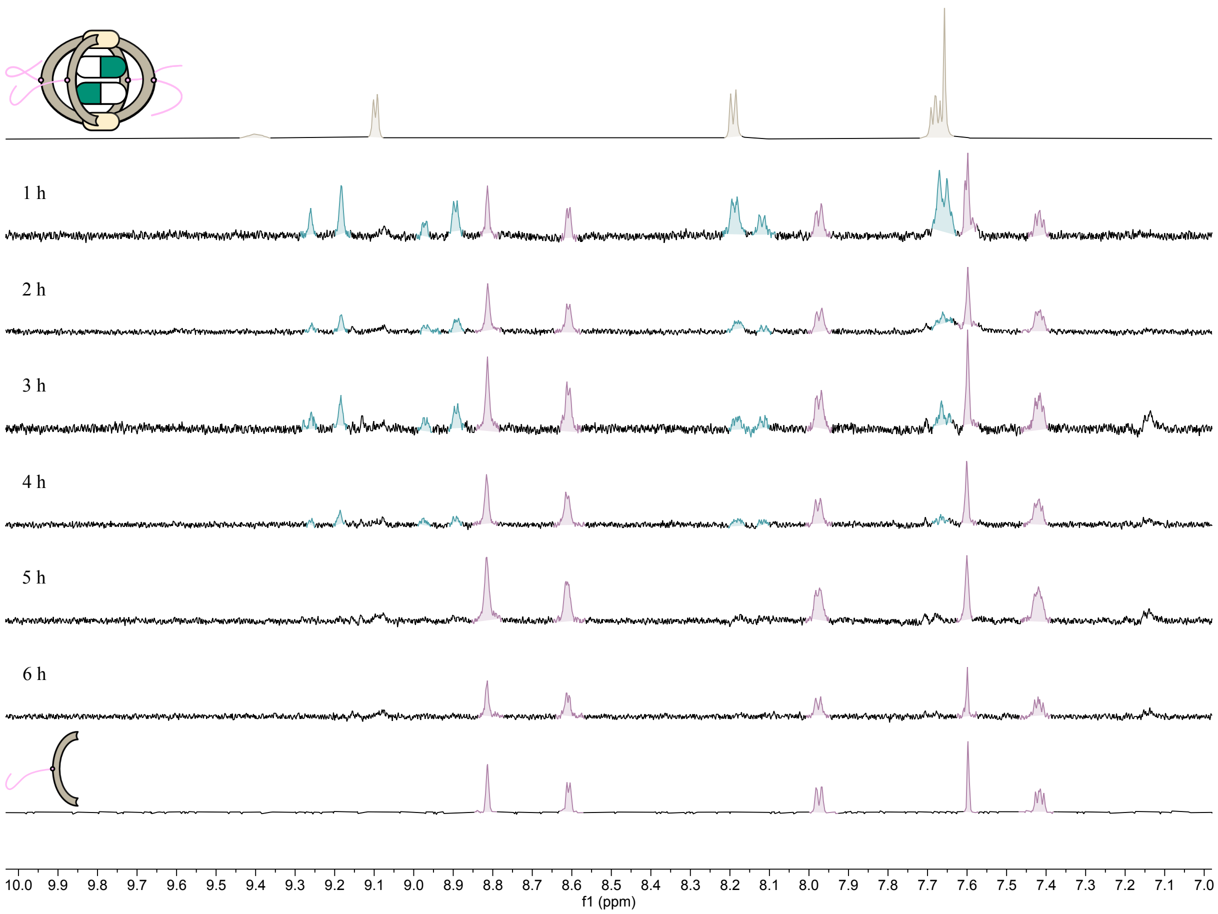

Figure S98: Enlarged view of stacked ^{1}H NMR spectra (600 MHz, CD_3CN, 298 K) showing the release progression of **PolyMOC3⊃(cisplatin)₂** over time. The spectra correspond to the initial complex (top), six sequential measurements recorded after each hour of sonication (middle), and **PolyL3** (bottom). The ^{1}H NMR signals are color-coded to indicate their respective species: **Pd_2L_4** (brown), **Pd_2L_3** (blue), and **PolyL3** (purple).

Argon as saturation gas

The release experiment, using argon as the saturation gas, was conducted in CD_3CN. Before and after each hour of sonication, a ^{1}H NMR spectrum was recorded to monitor the release of cisplatin and the disassembly of the cage. After just one hour of sonication, the cage was completely disassembled, and the cisplatin was fully released. Over the following five hours, only byproducts from the degradation of the PEG polymer chain and the ligand itself were observed. To confirm this unexpected result, the experiment was repeated twice, yielding the same outcome.

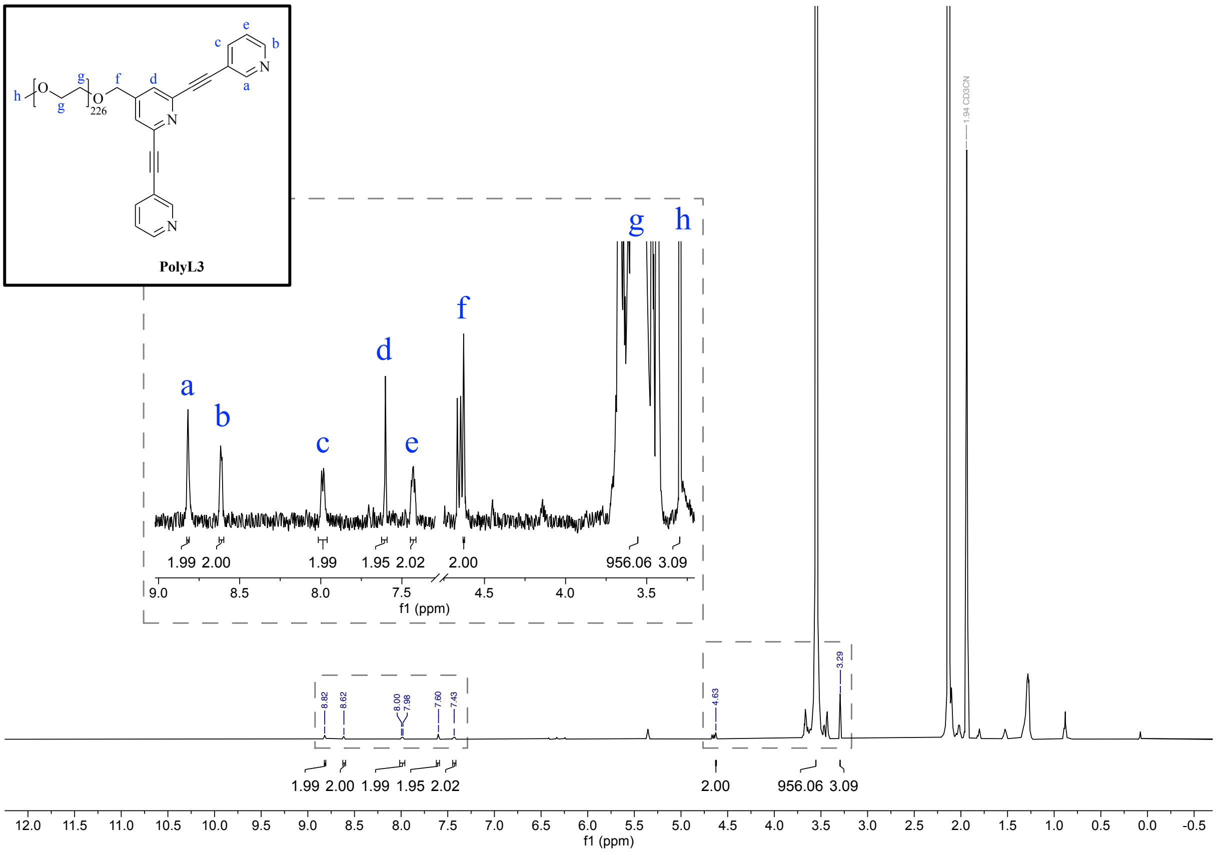

Figure S99: ^{1}H NMR spectrum (600 MHz, CD_3CN, 298 K) of **PolyMOC3⊃(cisplatin)$_2$** after one hour of sonication (Ar).

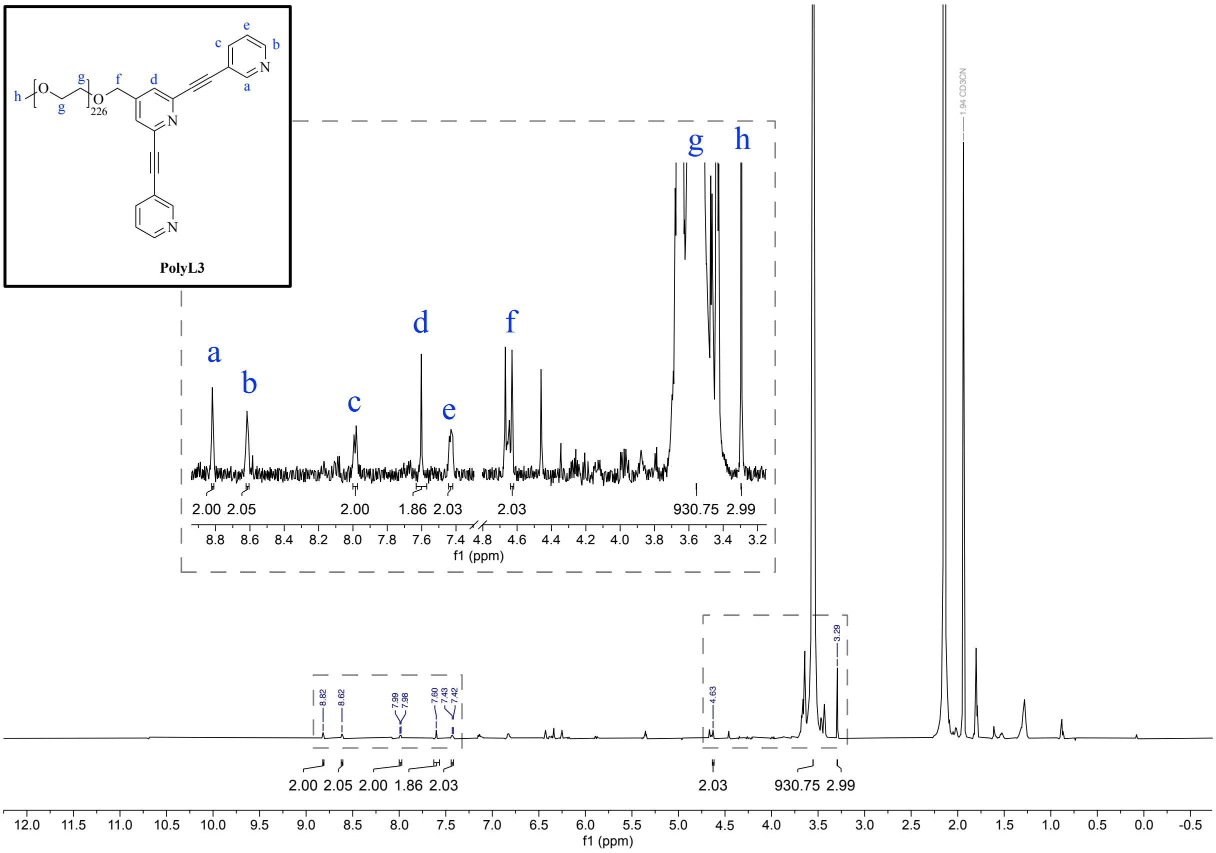

Figure S100: ^{1}H NMR spectrum (600 MHz, CD_3CN, 298 K) of **PolyMOC3⊃(cisplatin)$_2$** after six hours of sonication (Ar).

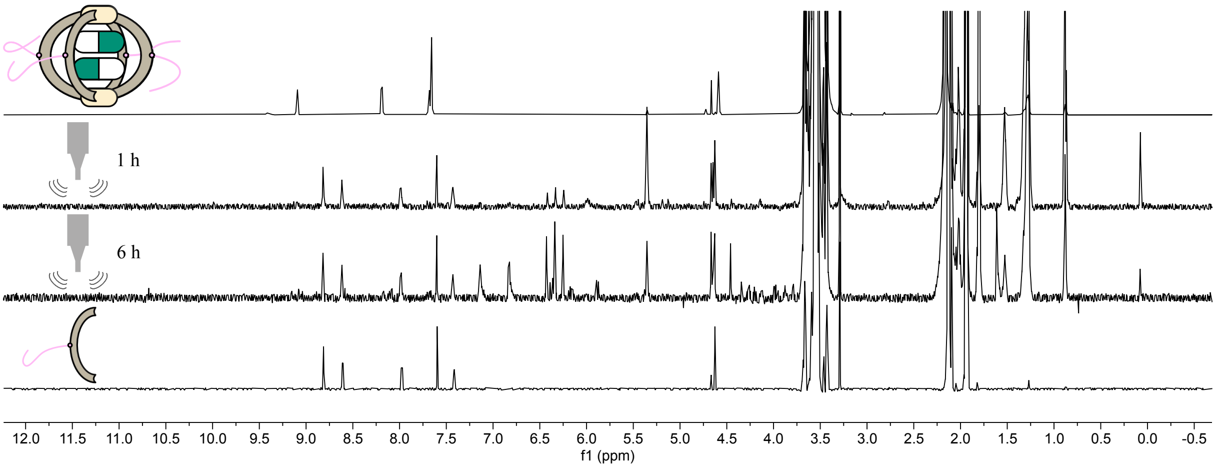

Figure S101: Stacked ^{1}H NMR spectra (600 MHz, CD_3CN, 298 K) of **PolyMOC3⊃(cisplatin)$_2$** (top), after one and after six hours of sonication (Ar) (middle), and **PolyL3** (bottom).

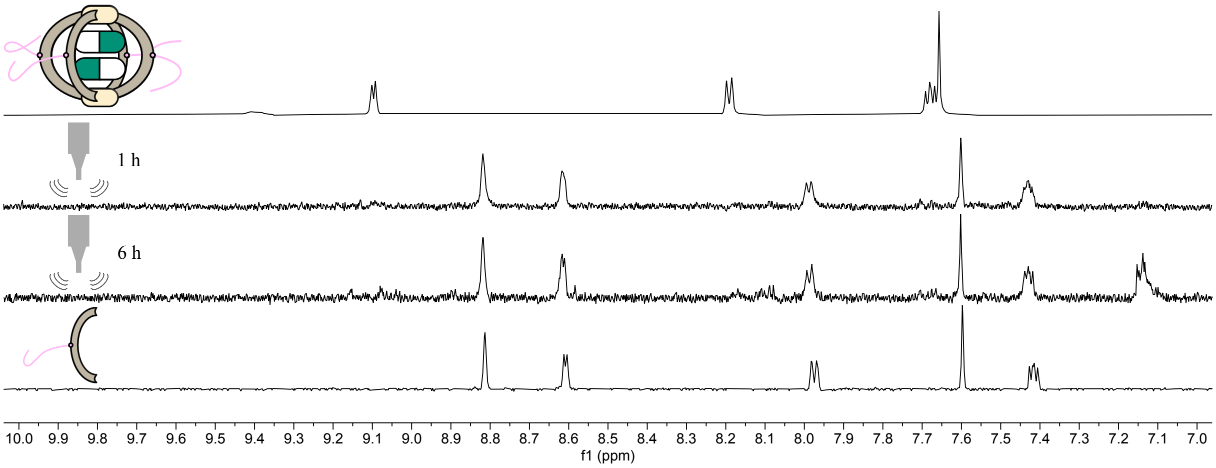

Figure S102: Enlarged view of stacked ^{1}H NMR spectra (600 MHz, CD_3CN, 298 K) of **PolyMOC3⊃(cisplatin)$_2$** (top), after one and six hours of sonication (Ar) (middle), and **PolyL3** (bottom).

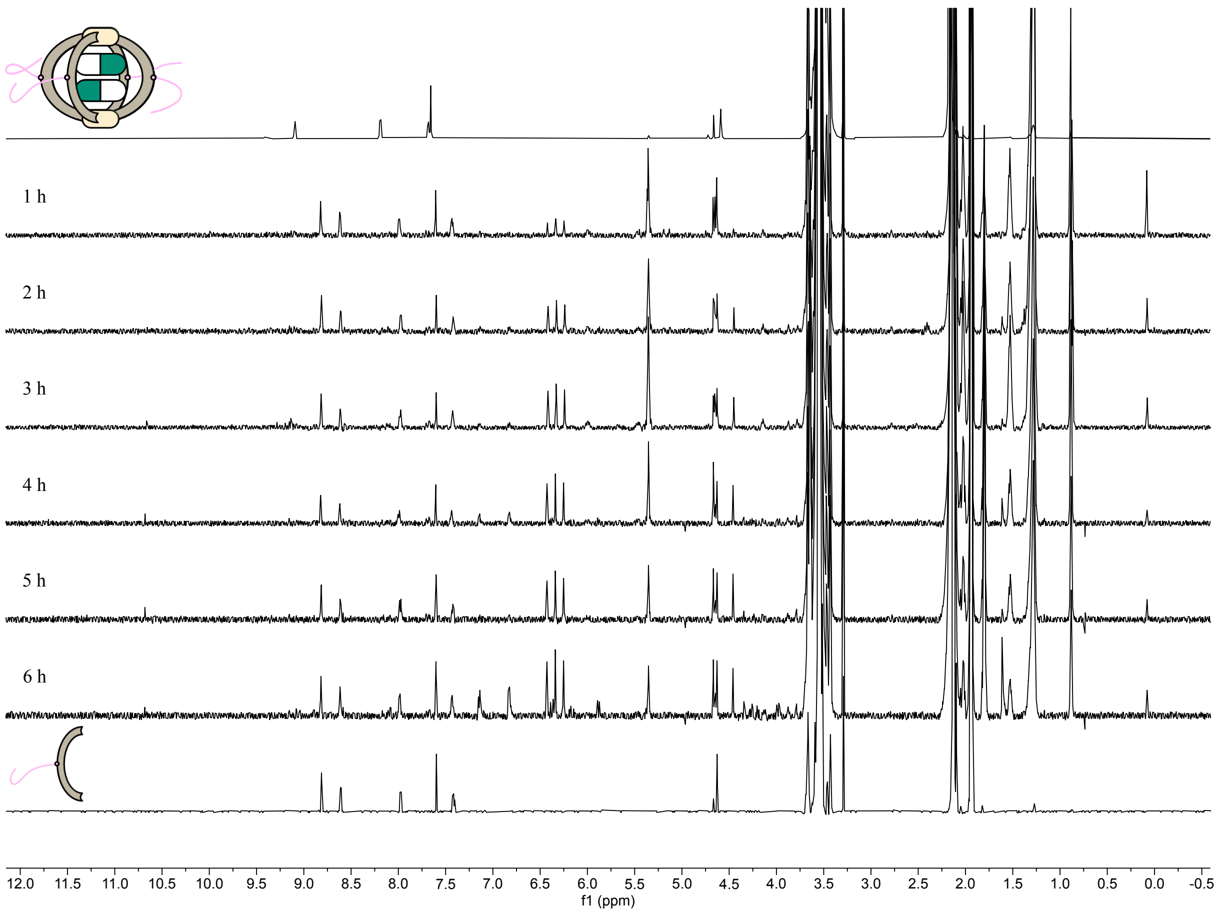

Figure S103: Stacked ^{1}H NMR spectra (600 MHz, CD_3CN, 298 K) showing the release progression of **PolyMOC3⊃(cisplatin)$_2$** over time. The spectra correspond to the initial complex (top), six sequential measurements recorded after each hour of sonication (middle), and **PolyL3** (bottom).

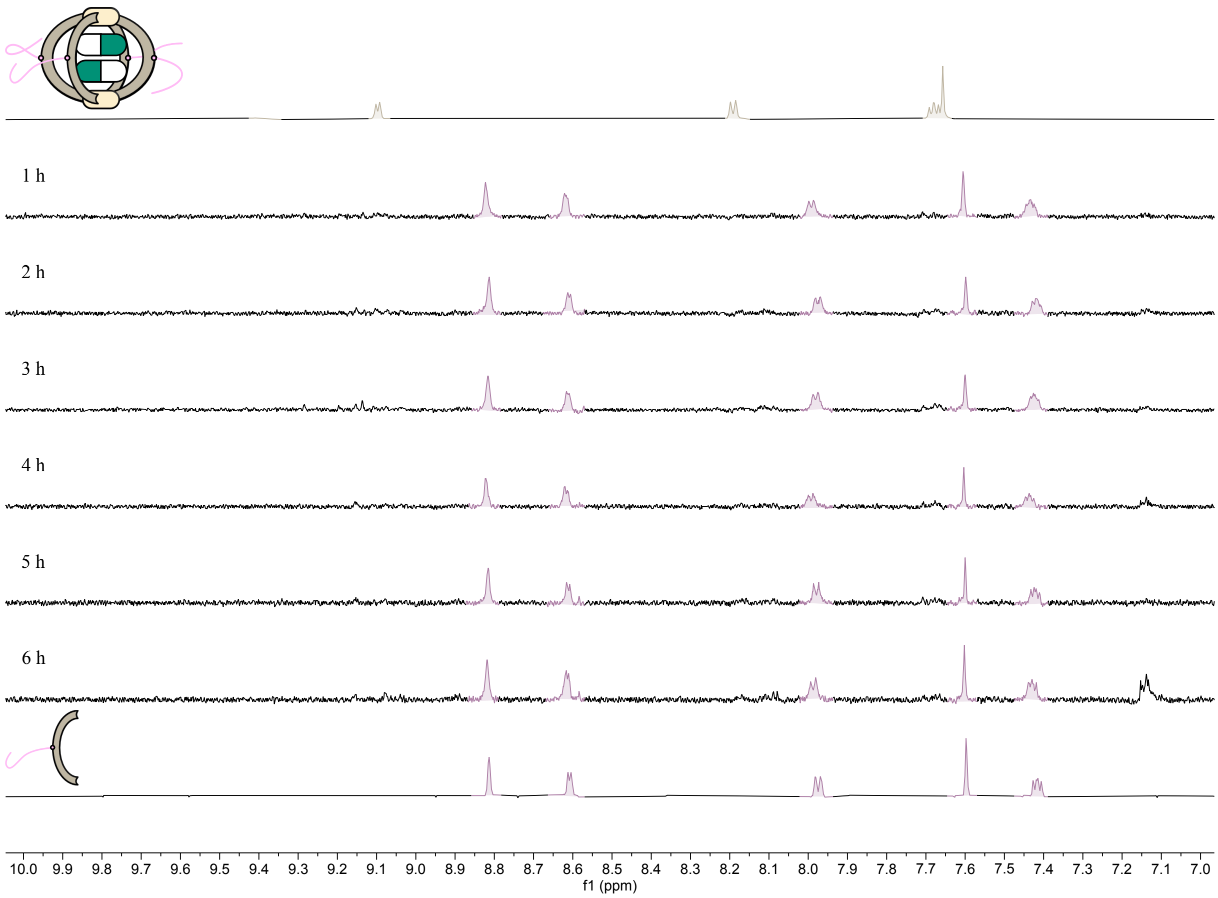

Figure S104: Enlarged view of stacked ^{1}H NMR spectra (600 MHz, CD_3CN, 298 K) showing the release progression of **PolyMOC3⊃(cisplatin)₂** over time. The spectra correspond to the initial complex (top), six sequential measurements recorded after each hour of sonication (middle), and **PolyL3** (bottom). The ^{1}H NMR signals are color-coded to indicate their respective species: **Pd_2L_4** (brown), and **PolyL3** (purple).

Cisplatin release from PolyMOC4⊃(cisplatin)$_2$

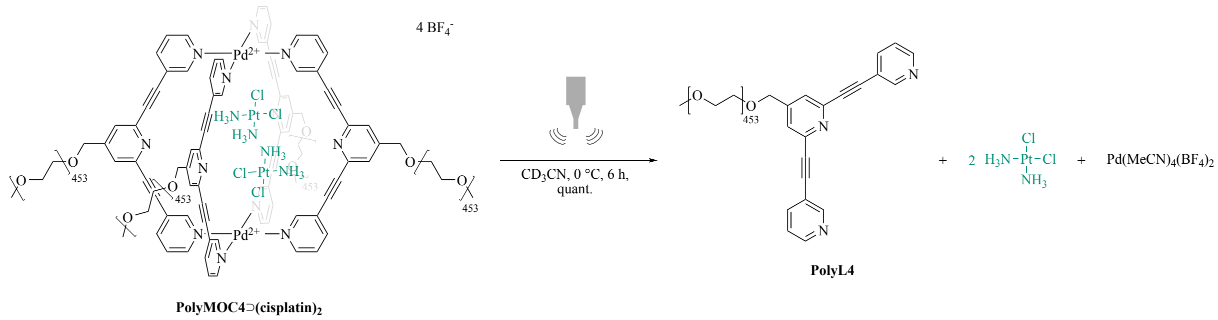

Scheme S105: Reaction equation for the release of cisplatin from **PolyMOC4⊃(cisplatin)₂**.

Nitrogen as saturation gas

The release experiment, using nitrogen as the saturation gas, was carried out in dry CH_3CN. After sonication, the entire mixture was transferred into a 50 mL round-bottom flask, frozen in liquid nitrogen, and freeze-dried under vacuum on a Schlenk line. A ^{1}H NMR spectrum was recorded at the same concentration (5.00 mg mL^{-1}) and compared to the initial complex

(**PolyMOC4⊃(cisplatin)₂**) and the free ligand **PolyL4**, confirming the complete activation of the cage.

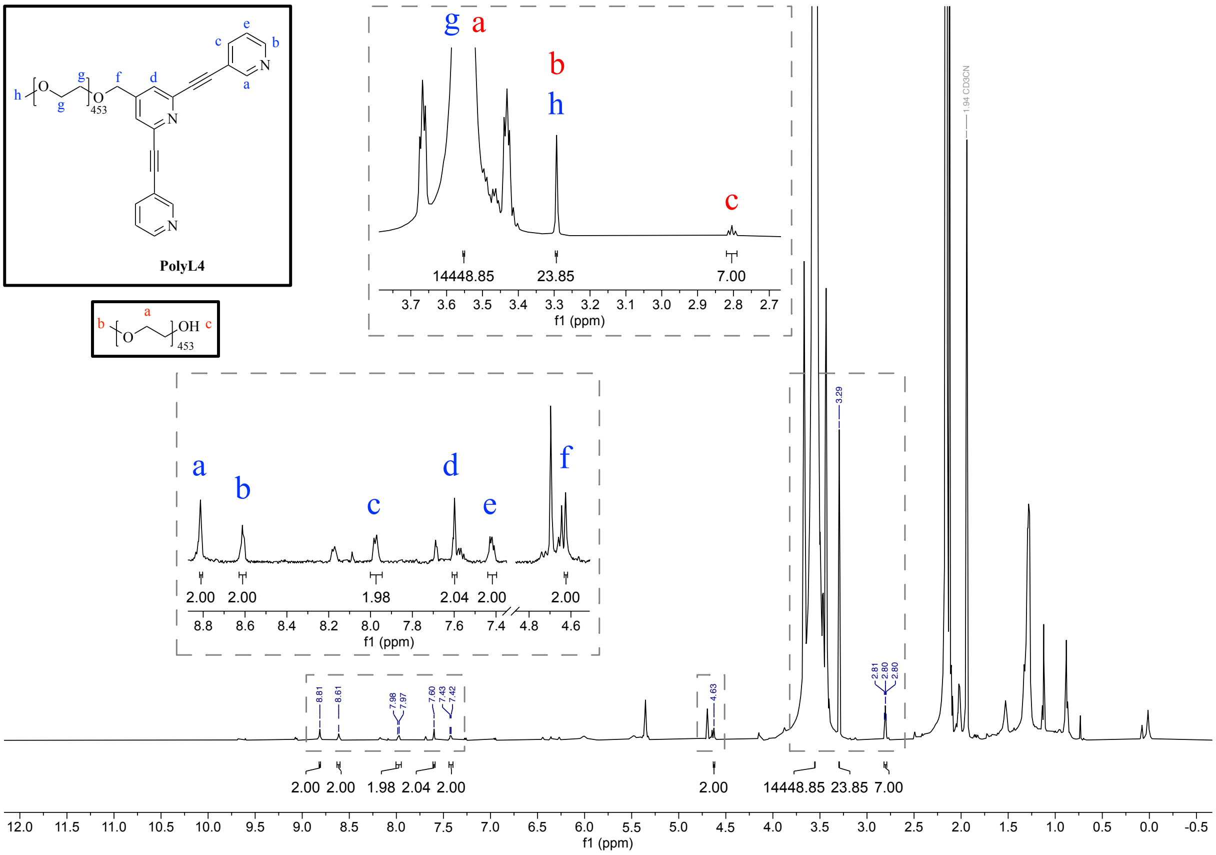

Figure S106: ^{1}H NMR spectrum (600 MHz, CD_3CN, 298 K) of **PolyMOC4⊃(cisplatin)₂** after six hours of sonication (N_2).

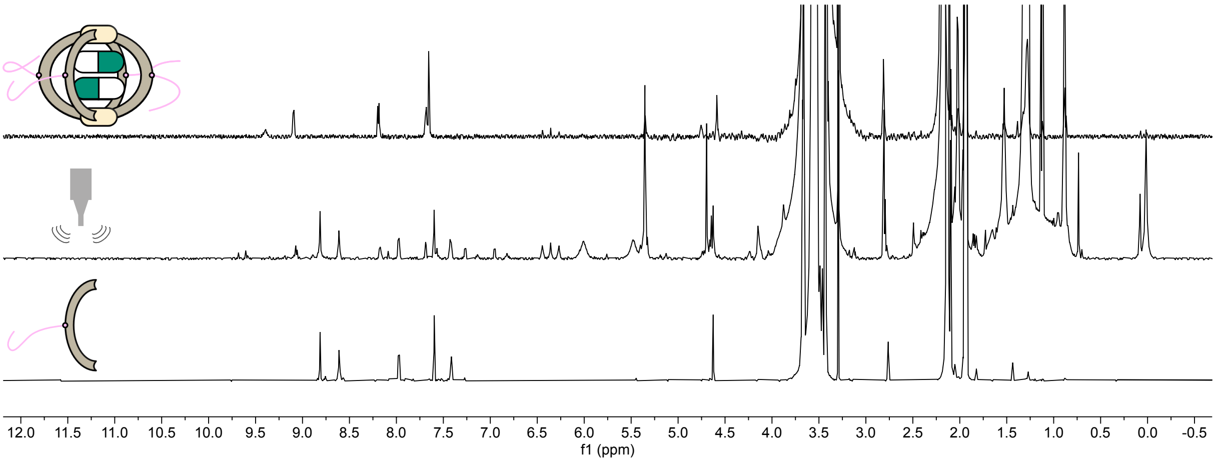

Figure S107: Stacked ^{1}H NMR spectra (600 MHz, CD_3CN, 298 K) spectra of **PolyMOC4⊃(cisplatin)₂** (top), after six hours of sonication (N_2) (middle), and **PolyL4** (bottom).

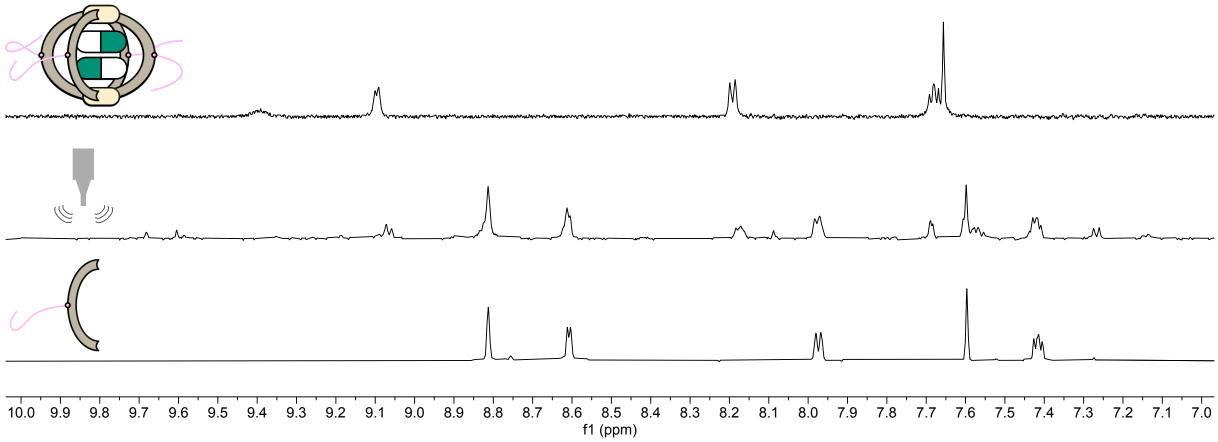

Figure S108: Enlarged view of stacked ^{1}H NMR spectra (600 MHz, CD_3CN, 298 K) of **PolyMOC4⊃(cisplatin)$_2$** (top), after six hours of sonication (N_2) (middle), and **PolyL4** (bottom).

Argon as saturation gas

The release experiment, using argon as the saturation gas, was carried out in dry CH_3CN. After sonication, the entire mixture was transferred into a 50 mL round-bottom flask, frozen in liquid nitrogen, and freeze-dried under vacuum on a Schlenk line. A ^{1}H NMR spectrum was recorded at the same concentration (5.00 mg mL^{-1}) and compared to the initial complex (**PolyMOC4⊃(cisplatin)$_2$**) and the free ligand **PolyL4**, confirming the complete activation of the cage. The signal-to-noise ratio and the broadening of the signals in the ^{1}H NMR spectrum of the activated cage was insufficient for precise signal integration. Therefore, the spectrum is presented without baseline and phase correction.

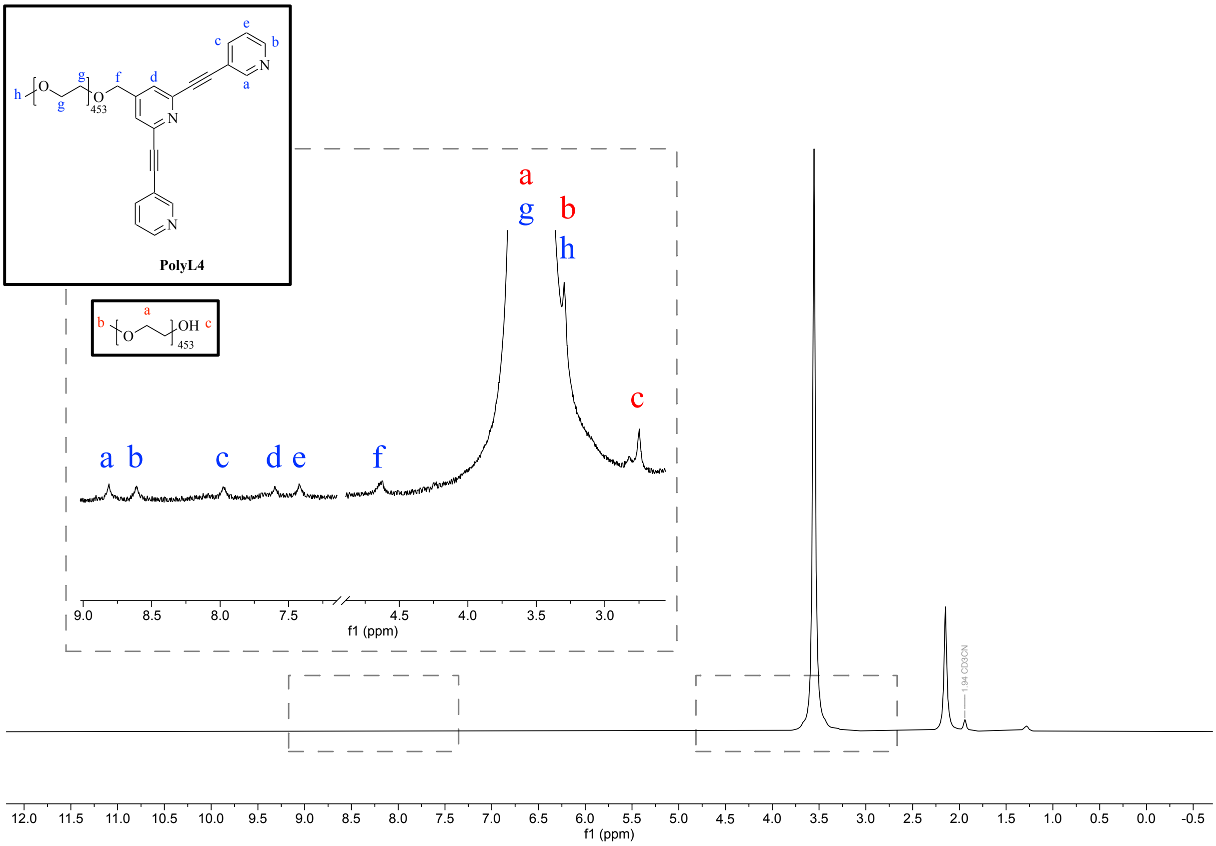

Figure S109: ^{1}H NMR spectrum (600 MHz, CD_3CN, 298 K) of **PolyMOC4⊃(cisplatin)$_2$** after six hours of sonication (Ar).

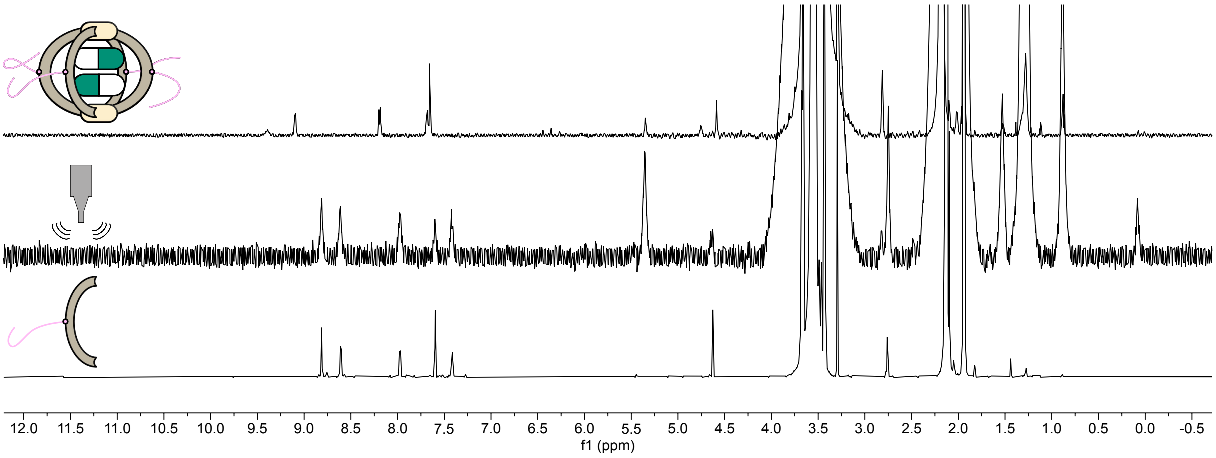

Figure S110: Stacked ^{1}H NMR spectra (600 MHz, CD_3CN, 298 K) spectra of **PolyMOC4⊃(cisplatin)$_2$** (top), after six hours of sonication (Ar) (middle), and **PolyL4** (bottom).

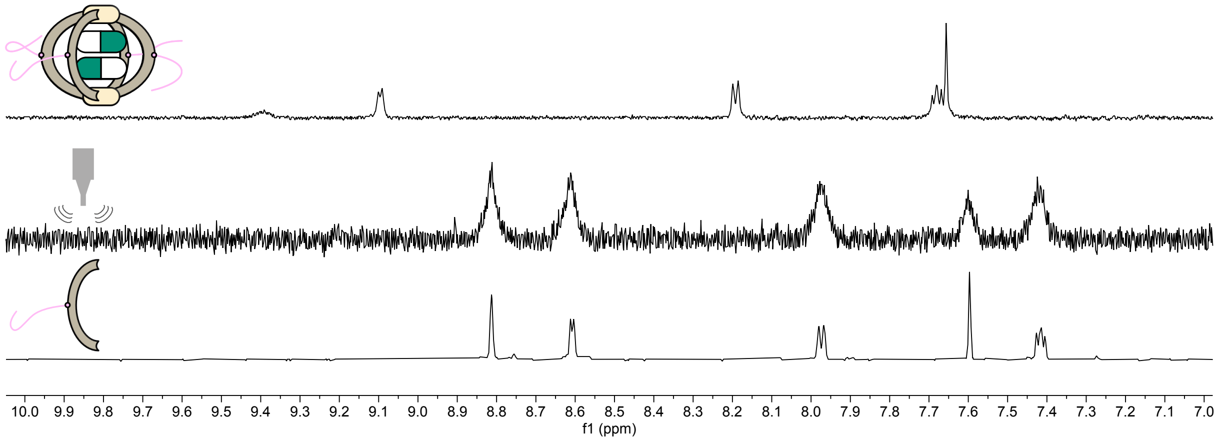

Figure S111: Enlarged view of stacked ^{1}H NMR spectra (600 MHz, CD_3CN, 298 K) spectra of **PolyMOC4⊃(cisplatin)₂** (top), after six hours of sonication (Ar) (middle), and **PolyL4** (bottom).

X. Ball milling induced disassembly and reassembly of PolyMOC2

All ball milling experiments were performed with a Retsch MM 400 with a frequency of 20.0 Hz. 20.0 mg of **PolyMOC2** and three zirconium oxide balls were added to a 2 mL Eppendorf tube, and the activation was done for 20 minutes. Then 1.25 mg was dissolved in 0.50 mL CD_3CN, and a ^1H NMR (c = 2.50 mg mL^{-1}) was measured to monitor the reaction progress (Fig. S112, middle bottom). The residual solid was redissolved in CD_3CN to reach a concentration of 20.0 mM. The resulting mixture was stirred for one hour at room temperature before a ^1H NMR was recorded (Fig. S112, middle top).

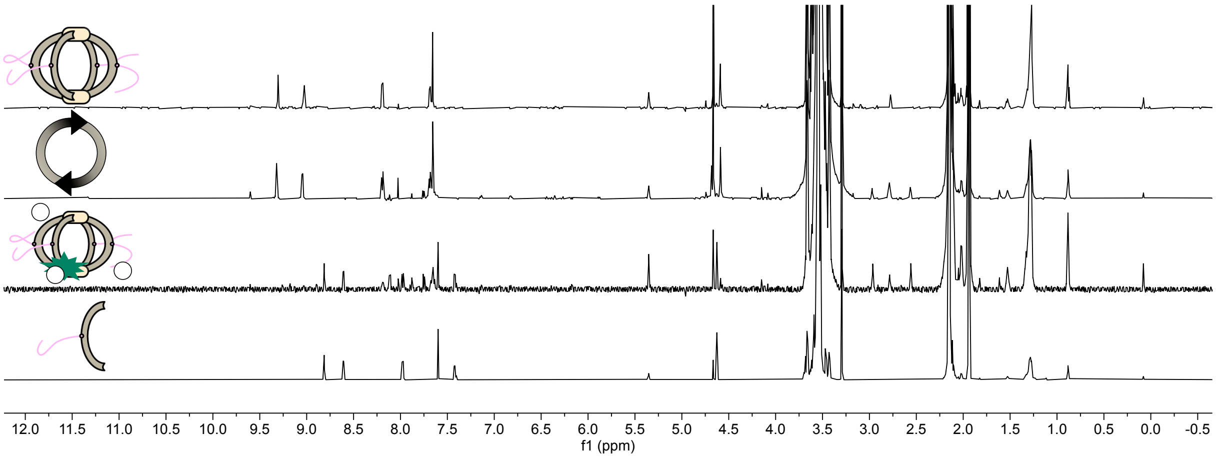

Figure S112: Stacked ^1H NMR spectra (600 MHz, CD_3CN, 298 K) of **PolyMOC2** (top), reversible self-assembly of **PolyMOC2** after ball mill activation (middle top), **PolyMOC2** after 20 minutes of ball milling (middle bottom), and **PolyL2** (bottom).

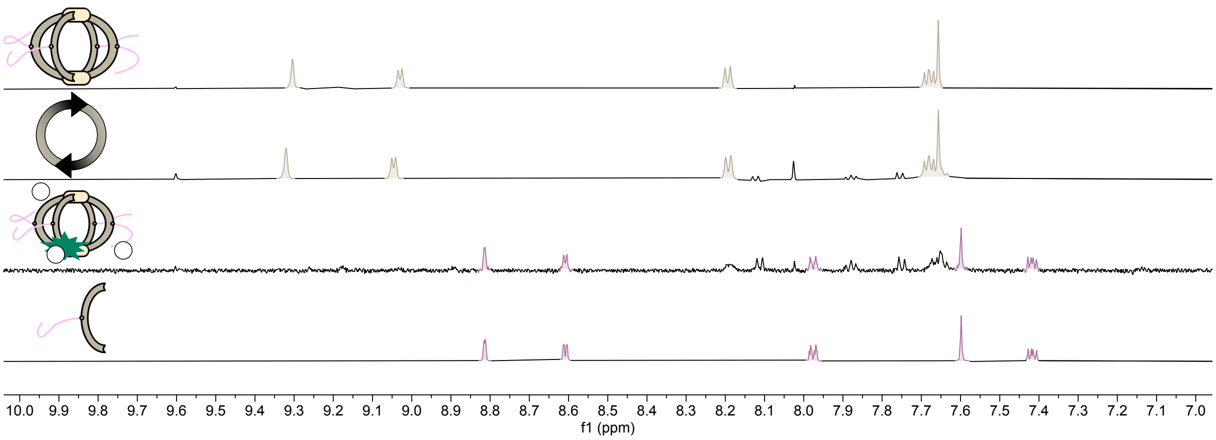

Figure S113: Enlarged view of stacked ^1H NMR spectra (600 MHz, CD_3CN, 298 K) of **PolyMOC2** (top), reversible self-assembly of **PolyMOC2** after ball mill activation (middle top), **PolyMOC2** after 20 minutes of ball milling (middle bottom), and **PolyL2** (bottom). The ^1H NMR signals are color-coded to indicate their respective species: $\mathbf{Pd_2L_4}$ (brown) and **PolyL2** (purple).

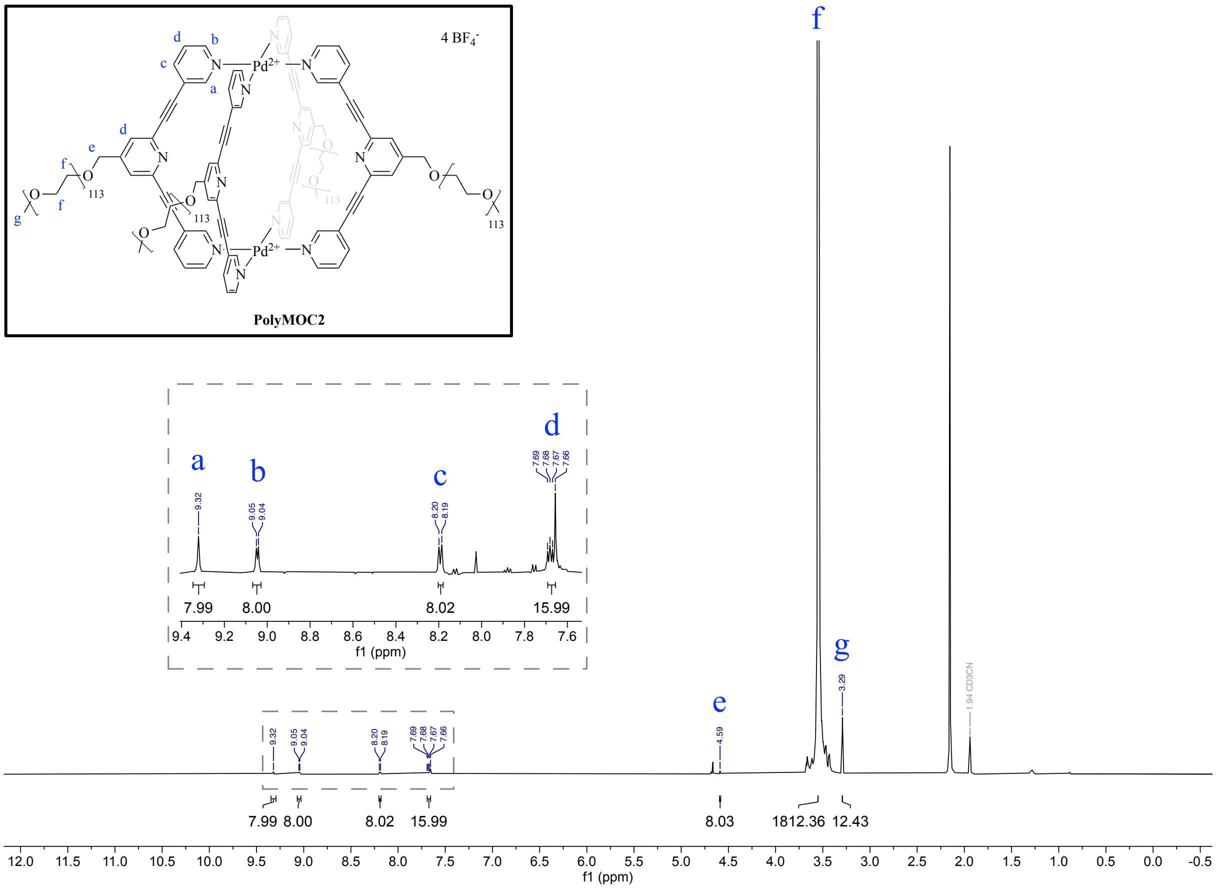

Figure S114: ^{1}H NMR spectrum (600 MHz, CD_3CN, 298 K) of reversible self-assembled **PolyMOC2** after ball mill activation.

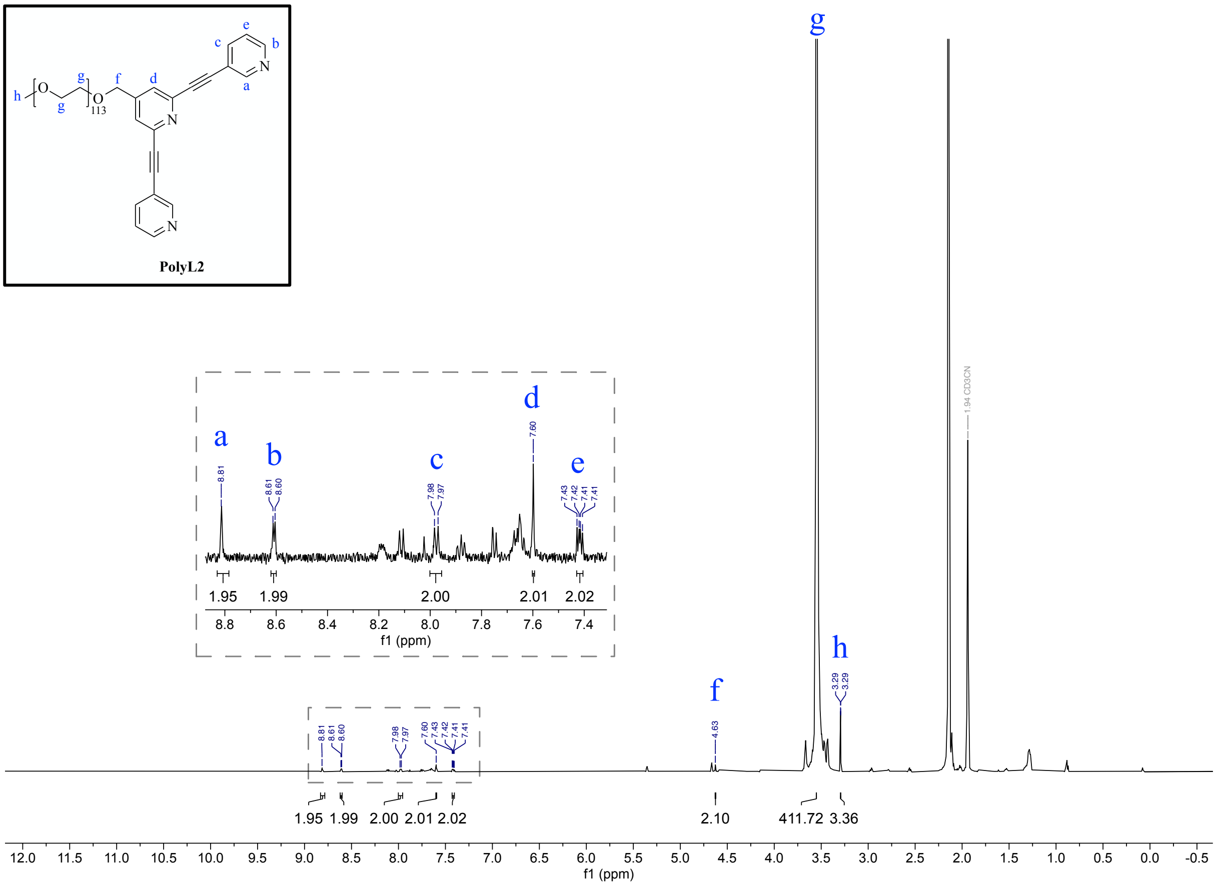

Figure S115: ^{1}H NMR spectrum (600 MHz, CD_3CN, 298 K) of **PolyMOC2** after 20 minutes of ball mill activation.

XI. Ultrasound-triggered disassembly of nanospheres

We also exposed **PolyMOS1** to ultrasound in deuterated acetonitrile to see if the previously developed ultrasound irradiation protocol could be applied as a universal protocol for the activation of Pd_nL_{2n} species. To do this, a **PolyMOS1** solution (7.50 mg mL^{-1}) was prepared in deuterated acetonitrile, degassed with either argon or nitrogen for ten minutes in a Suslick vessel, and then subjected to sonication for six hours (nitrogen) and nine hours (argon) at 0 °C. ^1H NMR spectroscopy was used to closely monitor the disassembly process.

Nitrogen as saturation gas

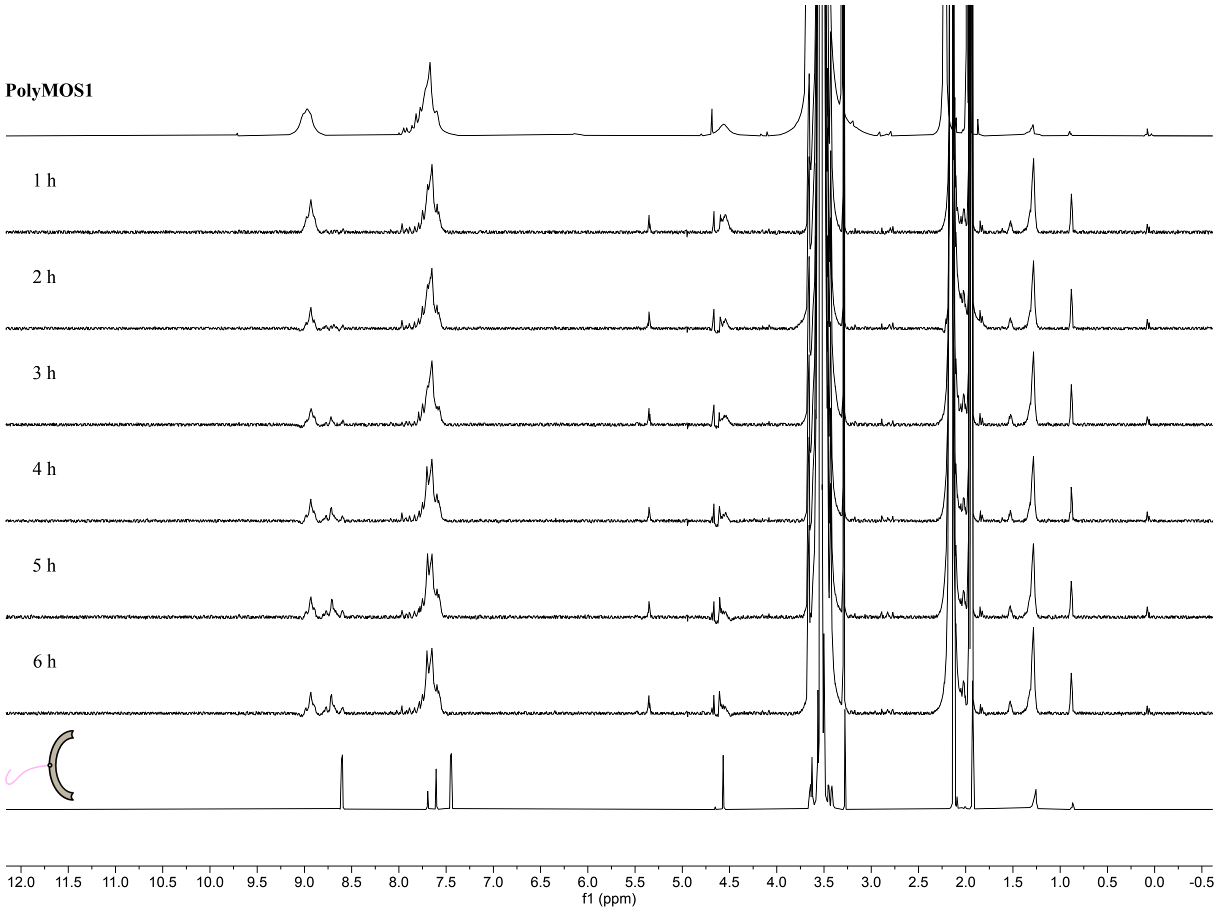

Figure S116: Stacked ^1H NMR spectra (600 MHz, CD_3CN, 298 K) showing the disassembly of **PolyMOS1** progressing over time. The spectra correspond to the initial nanosphere (top), six sequential measurements recorded after each hour of sonication (middle), and **PolyL5** (bottom).

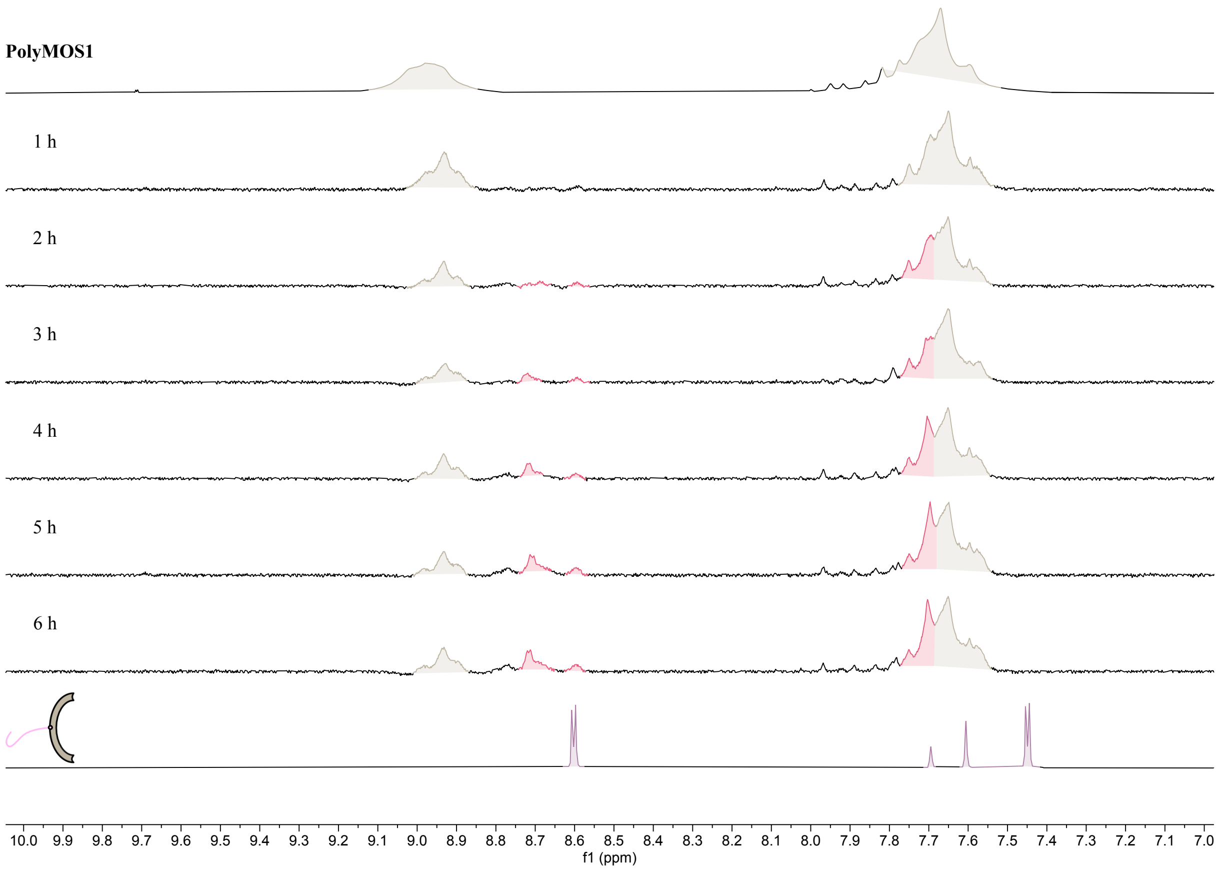

Figure S117: Enlarged view of stacked ^{1}H NMR spectra (600 MHz, CD_3CN, 298 K) showing the disassembly of **PolyMOS1** progressing over time. The spectra correspond to the initial nanosphere (top), six sequential measurements recorded after each hour of sonication (middle), and **PolyL5** (bottom). The ^{1}H NMR signals are color-coded to indicate their respective species: **PolyMOS1** (brown), unidentified products from disassembly (red), and **PolyL5** (purple).

Argon as saturation gas

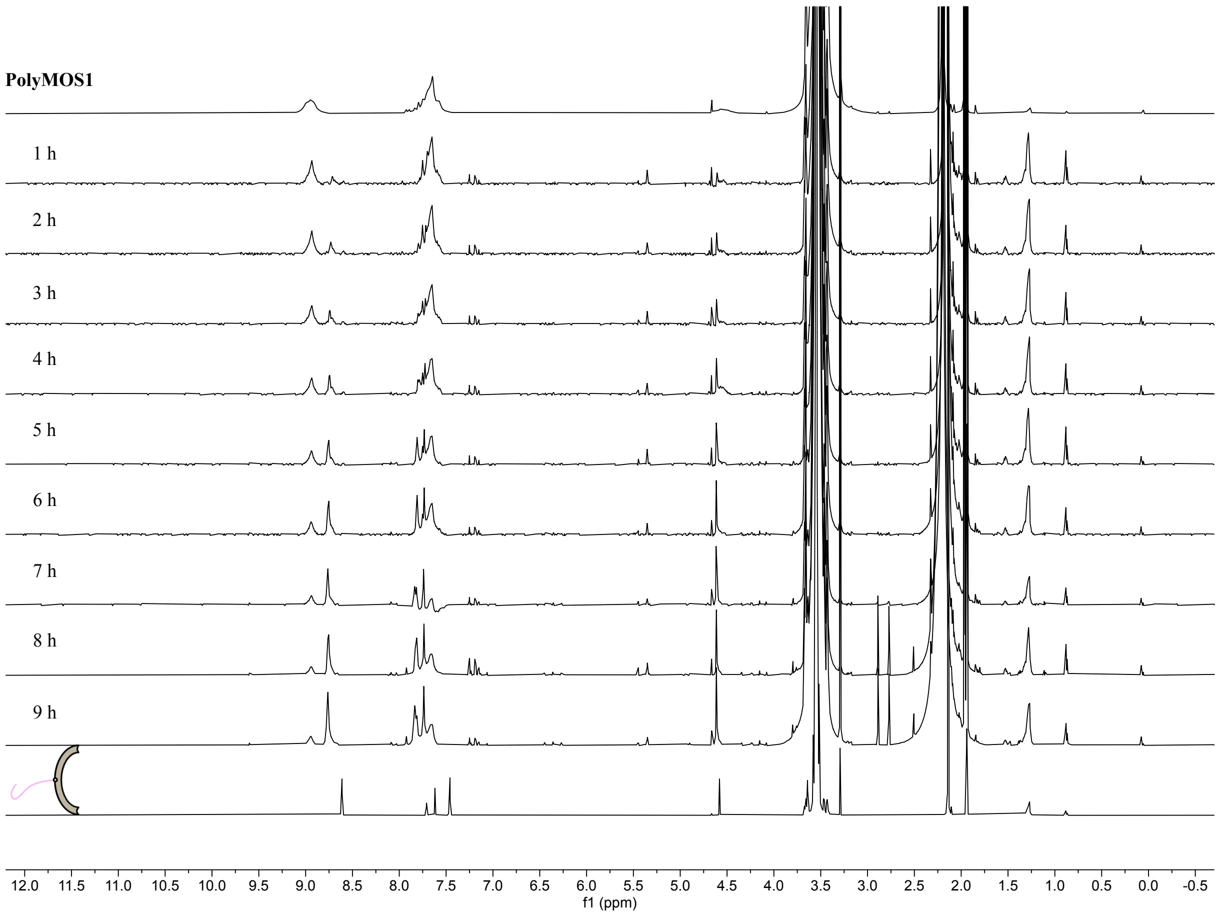

Figure S118: Stacked ^{1}H NMR spectra (600 MHz, CD_3CN, 298 K) showing the disassembly of **PolyMOS1** progressing over time. The spectra correspond to the initial nanosphere (top), nine sequential measurements recorded after each hour of sonication (middle), and **PolyL5** (bottom).

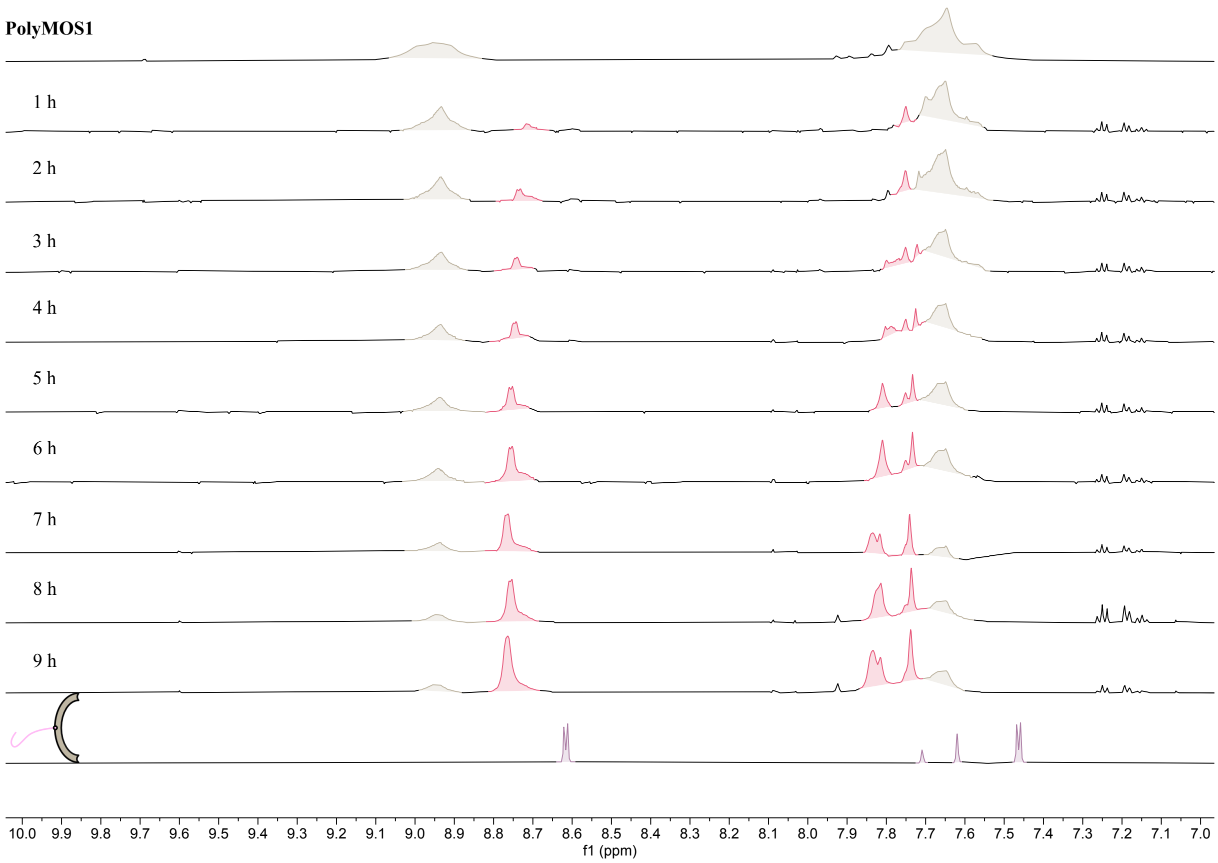

Figure S119: Enlarged view of stacked ^{1}H NMR spectra (600 MHz, CD_3CN, 298 K) showing the disassembly of **PolyMOS1** progressing over time. The spectra correspond to the initial nanosphere (top), six sequential measurements recorded after each hour of sonication (middle), and **PolyL5** (bottom). The ^{1}H NMR signals are color-coded to indicate their respective species: **PolyMOS1** (brown), unidentified products from disassembly (red), and **PolyL5** (purple).

XII. Molecular dynamics simulations and computational analysis of Pd_nL_{2n} disassembly

Benchmarking of potentials using a Pd(II)Py_4 model compound

As the number of atoms in the MOC structures is very high (134 in Pd_2L_4 and 828 $Pd_{12}L_{24}$), and explicit solvent molecules need to be included in the simulations to properly describe the force-induced ligand dissociation reaction, performing large-scale molecular dynamics (MD) simulations at the DFT level are not feasible. To find a suitable method for the computation of the potential energy for these simulations, we used a square-planar Pd(II) complex with four pyridine ligands as a model compound as a benchmark system for Pd–N chemistry. A single acetonitrile (MeCN) molecule was also included in the system to capture the full process of ligand dissociation and replacement by a solvent molecule. The total charge of the system is +2. The following methods were compared in this benchmark (Table S4):

Table S4: Level of theory and programs employed for the benchmark of different computational methods to describe the dissociation of a Pd-N bond.

Level of theory	Specifications	Program
DFT	B3LYP+D3[15-19]/6-31G*[20-22] with LanL2DZ ECP for Pd^{2+}[23]	TeraChem[24-26]
DFT	cam-B3LYP+D3[27]/6-31G* with LanL2DZ ECP for Pd^{2+}	TeraChem
DFT	cam-B3LYP+D3/def2-TZVP[28] with def2-ECP for Pd^{2+}[29]	TurboMole[30]
Semi-empirical	GFN2-xTB[17,31]	xTB
MLIP	pre-trained SpookyNet[32] model	SpookyNet
MLIP	pre-trained UMA-S[33]	FAIRchem
MLIP	pre-trained UMA-M[33]	FAIRchem

The geometry was optimized using the DL-FIND algorithm library[34]. For the DFT methods, the respective programs (TeraChem and TurboMole), were interfaced to DL-FIND via ChemShell[35]. For GFN2-xTB and the machine-learned interatomic potentials (MLIPs), a python-based in-house interface was used to connect to DL-FIND. We employed the pre-trained SpookyNet model as well as FAIRChem's Universal Model for Atoms (UMA), namely UMA-S and UMA-M. A restrained scan of one of the Pd–N distances was performed as an approximation of the Pd–N dissociation coordinate. Geometries with distances restrained between 2.0 Å and 3.5 Å were subsequently optimized, in steps of 0.1 Å. The jump in the energy profiles results from the rearrangement of ligands following the dissociation of one

pyridine (Figure S120). Table S3 shows the obtained dissociation energies and the corresponding rupture forces F_R.

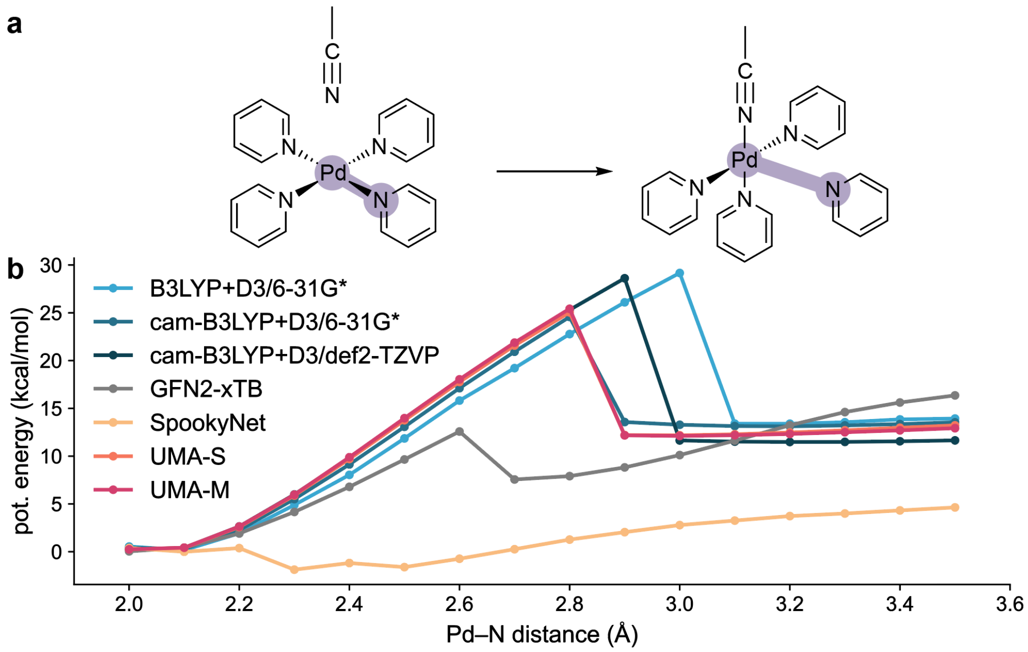

Figure S120: **a**: Pd(II)Py4 + MeCN model compound used for the comparison of electronic potentials. The Pd–N distance used in the constraint scan in marked in purple. **b**: Potential energy along the Pd–N distance coordinate, obtained from constraint scans computed at different levels of theory, relative to the respective reactant energy.

Table S5: Dissociation energies E_D and rupture forces F_R determined from the slope of the potential energy profile at its maximum, obtained using different methods

method	Dissociation energy E_D(kcal/mol)	Rupture Force F_R(nN)
B3LYP+D3/6-31G*	29.2	2.13
cam-B3LYP+D3/6-31G*	24.8	2.53
cam-B3LYP+D3/def2-TZVP	28.6	2.30
GFN2-xTB	12.6	2.03
SpookyNet	–	–
UMA-S	25.0	2.41
UMA-M	25.4	2.46

The SpookyNet MLIP fails to capture the barrier associated with the ligand exchange entirely, while GFN2-xTB massively underestimates it. Both UMA-S and UMA-M are able to reproduce DFT level results for the Pd–N dissociation reaction of the model compound. We therefore performed a geometry optimization of the Pd_2L_4 MOC using UMA-S and UMA-M, both of which resulted in a highly asymmetric, deformed structure, see Figure S121.

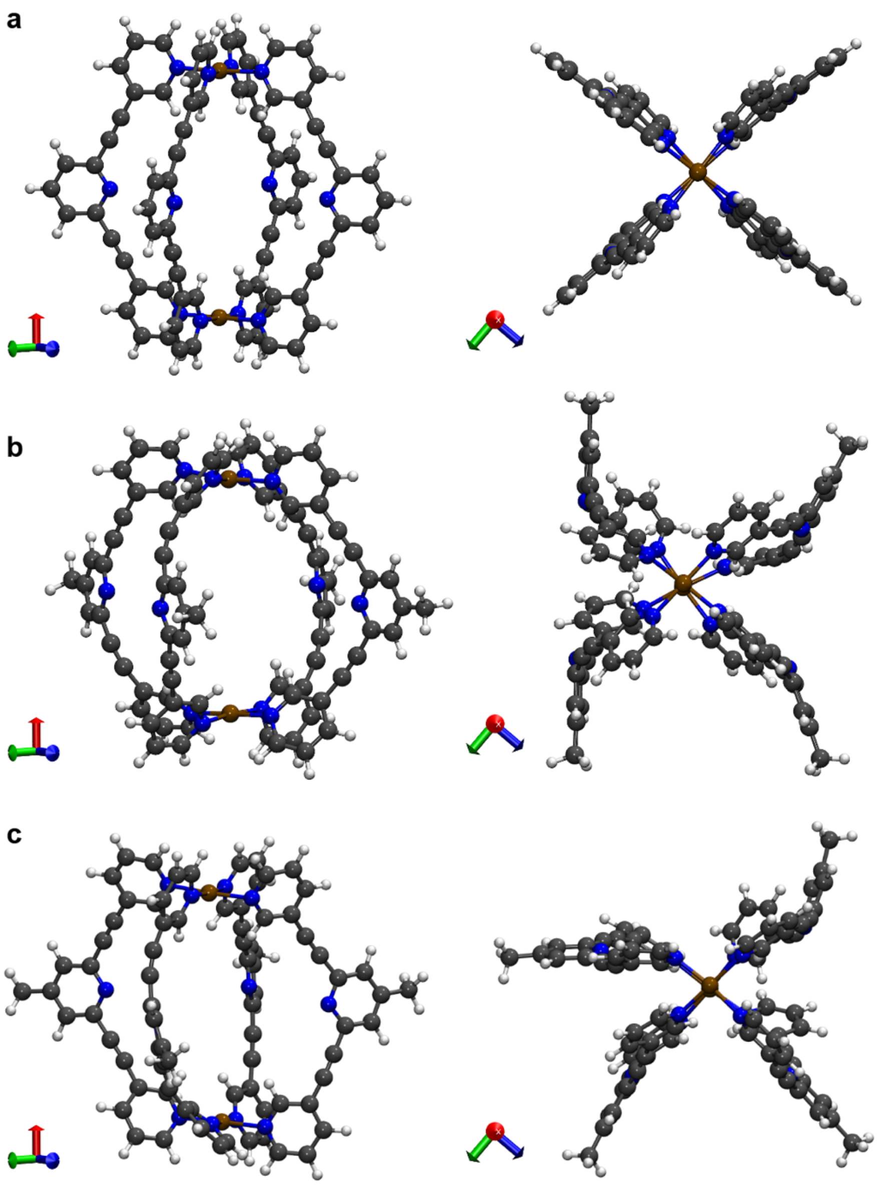

Figure S121: Geometry of the Pd_2L_4 MOC, optimized using **a**: DFT (B3LYP+D3/6-31G* with LanL2DZ ECP for Pd^{2+}) **b**: UMA-S. **c** UMA-M.

As none of the methods gives an accurate representation of Pd–N dissociation chemistry and MOC structure at a computational speed sufficient for the planned MD simulations, we fine–tuned the UMA-S MLIP.

Generation of ML training data

To construct the dataset for fine-tuning UMA-S, steered MD simulations for six different molecular fragments were conducted (Figure S122). The steering was necessary to increase the diversity of the dataset by sampling force-loaded molecular configurations and the associated structural distortions under pulling conditions. For the smaller fragments, the applied pulling

force was varied between 0.0 nN and 1.0 nN in increments of 0.25 nN, while for the Pd_2L_4 cage forces were varied between 0.0 nN and 3.0 nN in increments of 1.0 nN.

For fragments containing Pd–N bonds (a, b, c, f in Figure S122), a distance restraint (with a strong force constant of $2.2 \cdot 10^3$ kcal · mol^{-1} · Å^{-2}) was applied to systematically sample structures along the bond-stretching coordinate and ensure adequate coverage from the equilibrium geometry to bond dissociation. The restrained bond distance was varied in separate simulations over the series of 2.0, 2.2, 2.4, 2.6, 2.8, 2.9, 3.0, 3.1, 3.2, 4.0 and 5.0 Å. Furthermore, for the smallest of the systems (Figure S122a), the difference between Pd–N bond lengths of dissociating ligand and incoming acetonitrile molecule was restraint (using a force constant of $1.1 \cdot 10^3$ kcal · mol^{-1} · Å^{-2}) to specifically sample process of dissociation followed by the direct coordination of a nearby acetonitrile molecule to Pd(II). The bond difference was varied between 0.0 and 0.5 Å in increments of 0.1 Å. In addition to the Pd(II)-complexes, an isolated ligand with and without explicit acetonitrile solvent was included using SMD simulations. This approach yielded a dataset of 1,600,332 structures.

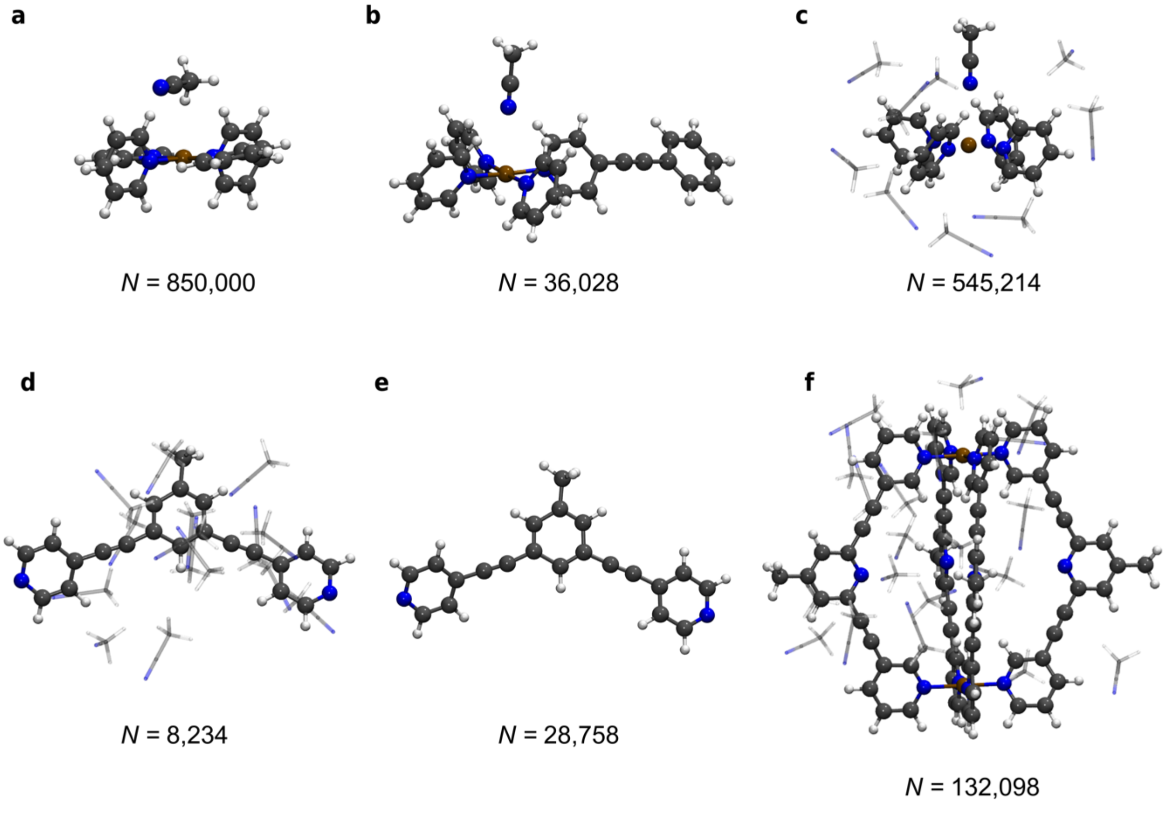

Figure S122: Structures used for the dataset generation for fine-tuning the UMA-S MLIP. The number N indicates the number of structures of each compound generated for training the MLIP. **a** Pd(II)Py_4 with a single acetonitrile molecule, N = 850,000. **b** Pd(II) coordinated by three pyridine and one 4-(phenylethinyl)pyridine with one reacting acetonitrile molecule, N = 36,028. **c** Pd(II)Py_4 with one reacting and nine surrounding acetonitrile molecules, N = 545,214. **d** Representative ligand of the $Pd_{12}L_{24}$ sphere with one reacting and 15 surrounding acetonitrile

molecules, $N = 8{,}234$. **e** Representative ligand of the $Pd_{12}L_{24}$ sphere, $N = 28{,}758$. **f** Pd_2L_4 MOC with 20 explicit acetonitrile molecules, $N = 132{,}098$.

Fine tuning UMA-S

To fine-tune the UMA-S pre-trained model, the dataset was split into training/validation/test (80/10/10) using a stratified scheme to preserve the distribution of system types and sampling configurations. Training used a single H100 CUDA device with 200,000 optimization steps, mini-batches of 20, and the AdamW optimizer[36] (learning rate $4 \cdot 10^{-4}$ and weight decay $1 \cdot 10^{-3}$). A cosine learning-rate schedule[37] with warmup was applied (warmup factor 0.2, learning rate factor 0.01, total horizon 200,000 steps). Model parameter gradients were clipped at a global-norm of 100. Graphs were built on-the-fly with a neighbor cutoff permitting up to 100 neighbors per atom. Batches were formed with a balanced batch sampler (shuffle enabled, seed 0). Validation was run every 500 steps and checkpoints were written every 1,000 steps (max 5 retained); the final model was selected by the minimum validation loss. The model was initialized from the released UMA-S checkpoint and fine-tuned with energy+force heads; periodic boundary conditions were disabled for training, matching the downstream setup.

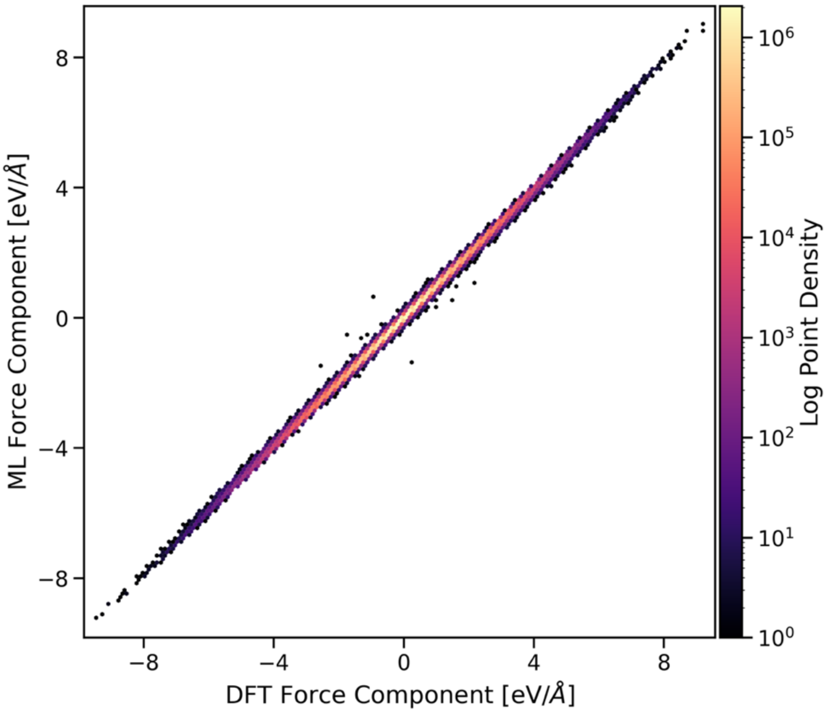

Figure S123: Parity plot of atomic force components from the fine-tuned UMA-S versus reference DFT (B3LYP+D3/6-31G* with LanL2DZ ECP for Pd^{2+}). The color scale indicates the logarithm of the point density. The overall root-mean-square error is 18.8 meV · $Å^{-1}$.

The quality of the model was quantified on the held-out test split and on an additional, strictly unseen trajectory to assess generalization beyond MD-path correlations. On the test split, the energy RMSE is 7.89 meV (0.18 kcal · mol^{-1}) and the force-component RMSE is 18.8 meV· $Å^{-1}$ (0.43 kcal · mol^{-1} · $Å^{-1}$). Figure S123 shows the corresponding DFT-versus-ML parity plot for force components. On the independent 1000 step trajectory (not used for training, validation, or model selection), the energy RMSE is 7.74 meV (0.18 kcal · mol^{-1}) and the force-component RMSE is 32.2 meV· $Å^{-1}$ (0.74 kcal · mol^{-1} · $Å^{-1}$). These results indicate that the fine-tuned UMA-S reproduces DFT energies and gradients at the accuracy needed for the ultrasound-controlled disassembly simulations, with reasonable transferability to unseen configurations.

The scan along one Pd–N distance coordinate was repeated using the fine-tuned MLIP (UMA-S-PdN), showing very good agreement with the DFT (B3LYP+D3/6-31G*) reference (Figure S124). A nudged elastic band (NEB)[38,39] path was optimized using the UMA-S-PdN. A total of 30 images were optimized between the model compound‘s reactant structure ($PdPy_4$ + MeCN) and the structure at the end point of the aforementioned restraint scan along the Pd–N coordinate ($PdPy_3MeCN$ + Py). For this, the NEB implementation in the DL-FIND[34] library was used. On the optimized images, DFT singlepoint energies were computed at the B3LYP+D3/6-31G* (with LanL2DZ ECP for Pd^{2+})[23] level. Both the MLIP NEB and the DFT energies are shown in Figure S125. The first small barrier along the path results from a rotation of one of the non-reactive pyridine ligands (Figure S124-125).

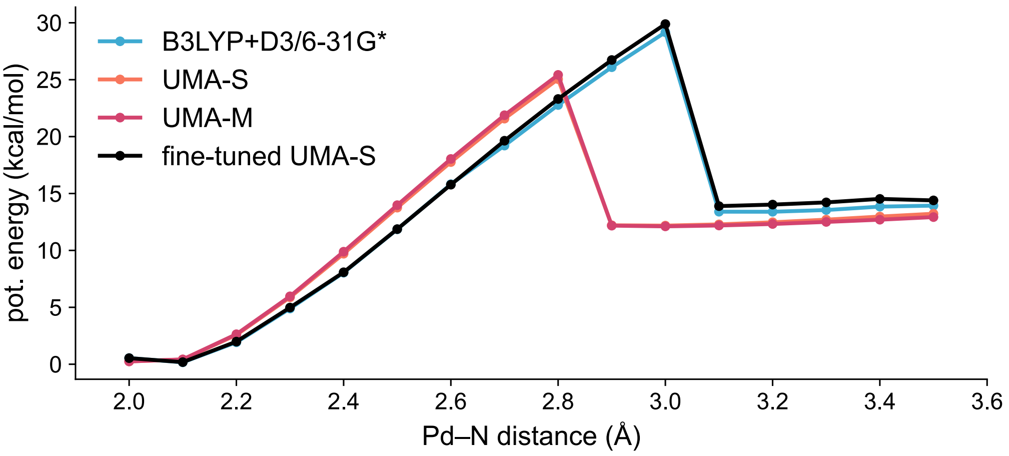

Figure S124: Potential energy along the Pd–N distance coordinate, obtained from constraint scans computed at different levels of theory, relative to the respective reactant energy. The UMA-S-PdN MLIP presented in this section is shown in black.

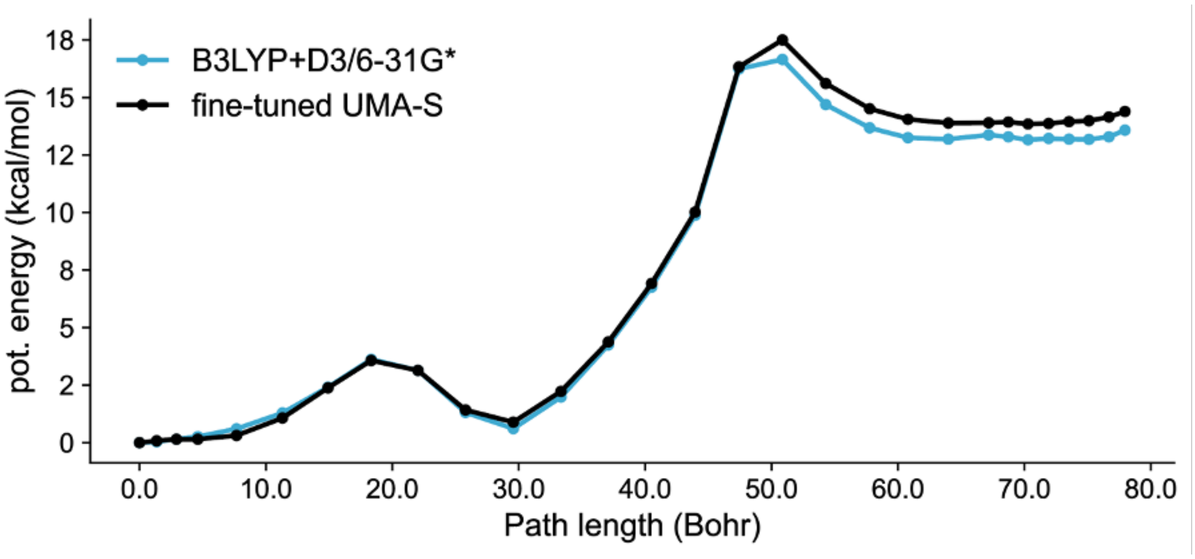

Figure S125: Potential energy along the NEB path from model compound reactant structure ($PdPy_4$ + MeCN) to the structure after ligand dissociation ($PdPy_3MeCN$ + Py). Energies were computed using the UMA-S-PdN MLIP (black), and B3LYP+D3/6-31G* (with LanL2DZ ECP for Pd^{2+}) (blue).

Ramped steered molecular dynamics simulations (RS-MD)

For the elucidation of the cage dissociation mechanism, we performed steered molecular dynamics (SMD)[40] simulations. Rather than applying an external force of constant strength, the external force applied in the simulation is gradually increased ("ramped up") throughout the runtime, hence the name "ramped steered molecular dynamics" (RS-MD) was chosen. The time-dependent force $F(t)$ is chosen to increase linearly (Eq. S13),

$$F(t) = \dot{k} \cdot t, \tag{S13}$$

where $\dot{k} = \frac{\mathrm{d}k}{\mathrm{d}t}$ is the force ramp, i.e., the slope of the force function and t is the simulation time. Figure S126 illustrates the linear increase of the external pulling force applied in the RS-MD protocol.

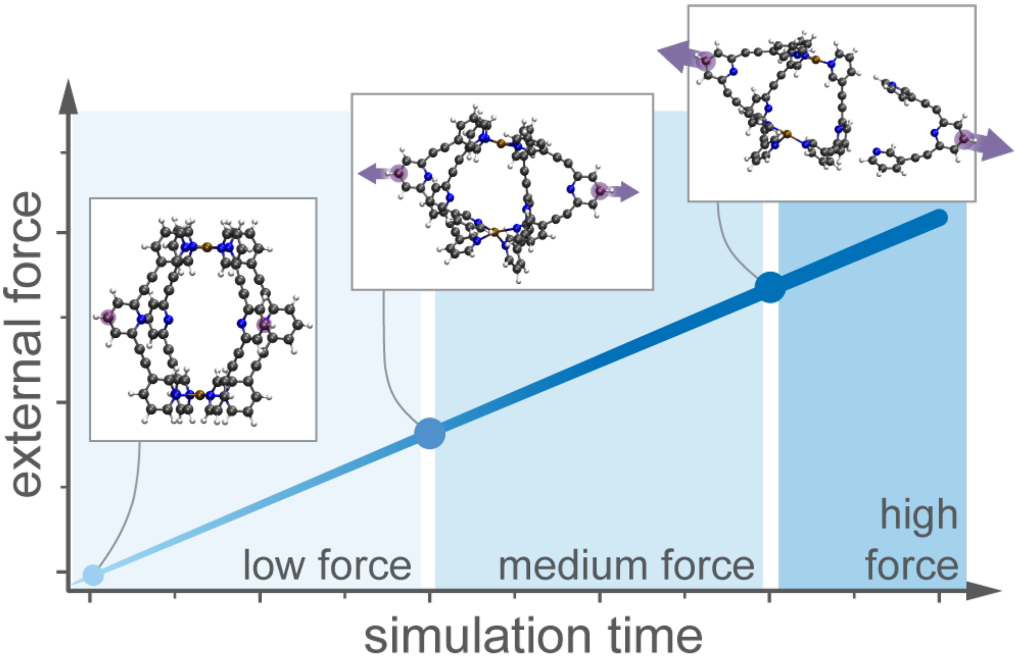

Figure S126: Schematic illustration of the RS-MD method. Linear force ramping results first in conformational changes, then in a mechanochemical bond scission reaction. Pulling forces acting on the molecule are marked in purple.

In the context of mechanochemical reaction discovery, RS-MD is intended to be a complement to the currently established "constrained geometries simulate external force" (CoGEF) method[41]. As MD simulations incorporate kinetic energy and, hence, thermal fluctuations, predictions made by RS-MD more accurately reflect real, dynamic systems. Since the RS-MD method simulates trajectories on the force-modified potential energy surface instead of relying on geometric constraints, no degrees of freedom are artificially restricted. By sampling the initial geometries and velocities from a probability distribution to generate randomized starting conditions, multiple RS-MD simulations can give a statistical distribution of possible outcomes, potentially offering insight into different reaction pathways.

The external force was included in the computations through a modification of the potential implemented in an in-house MD code, according to the adaptive-steering variant of the *force-modified potential energy surface* (FM-PES) method[40], which is equivalent to the *external force is explicitly included* (EFEI) method[42]. In this formalism, the effective force-modified potential is calculated as the sum of the electronic *ab initio* potential and the mechanical force term, $-F \cdot \Delta x$, where F is the external force and Δx is the distance between the two atoms to which force is applied. To achieve the force-ramping, F was increased by a constant value at each MD step, starting from $F = 0$ nN at the beginning of the RS-MD simulations.

All RS-MD simulations were performed using the UMA-S-PdN described in the previous section. The simulations were run in an NVT ensemble using the Bussi-Parrinello version of Langevin dynamics[43] with a timestep of 1 fs, a thermostat temperature of 300 K, and a friction coefficient of 7 ps^{-1}.

RS-MD simulations – pulling modes

Pulling forces were applied to the carbon atoms of the linker ligands where the polymer chains are attached in the experiment, in the following referred to as pulling points. In the RS-MD framework, pulling forces are applied to a pair of selected atoms. The fact that there are more than two pulling points in the MOCs, i.e. one per ligand in the Pd_2L_4 and $Pd_{12}L_{24}$ systems, was considered in the computations by performing simulations for different combinations of pulling pairs. In the smaller Pd_2L_4 cage, the four combinations of pulling pairs shown in Figure S127 were examined.

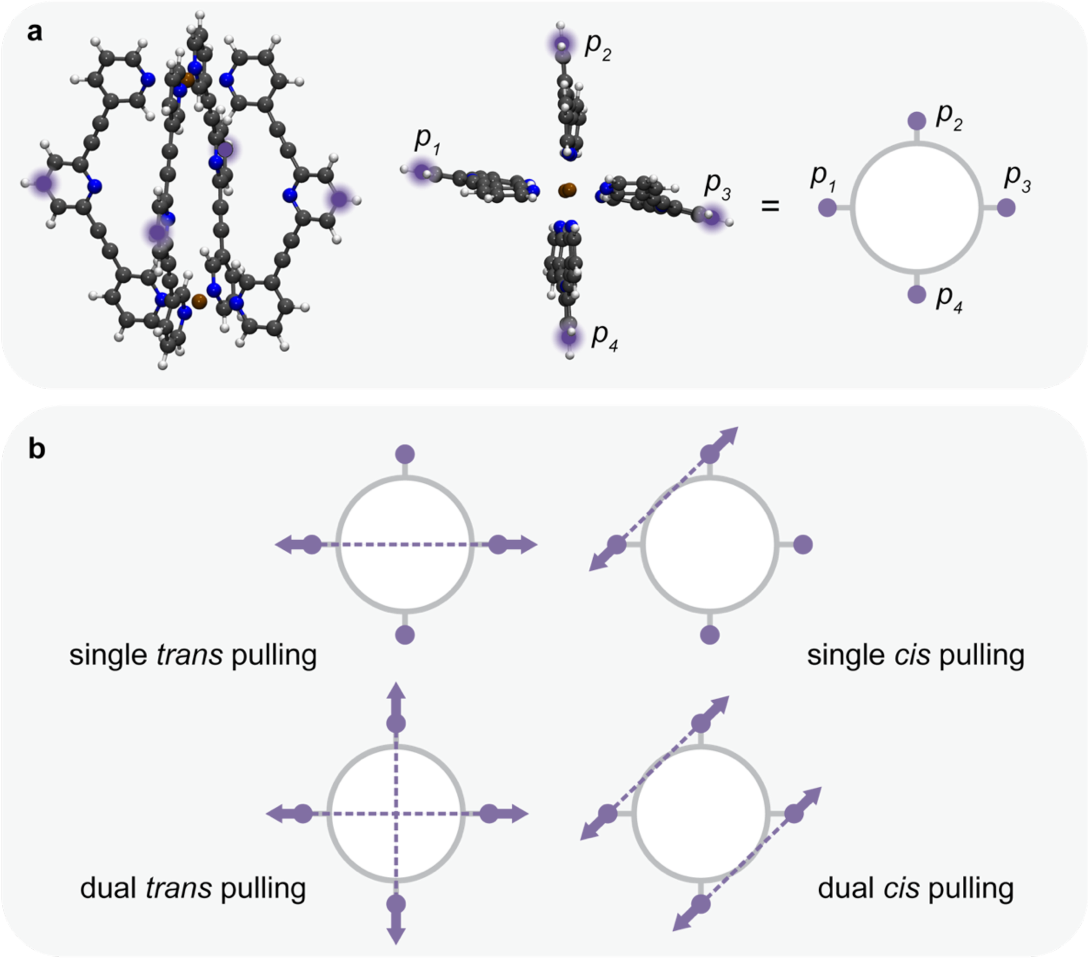

Figure S127: **a**: Pulling points (purple) on the Pd_2L_4 MOC structure. **b** Different combinations of pulling points chosen for the application of the ramping pulling force in RS-MD simulations.

For the $Pd_{12}L_{24}$ MOS, the total number of 24 pulling points makes it impossible to simulate all possible pulling modes. Assuming that the pulling force induced through ultrasound-triggered cavitation events acts along a main axis of the MOS, all pulling pairs that are orthogonal to this axis can be considered unaffected by the force. To determine which pulling pairs are in suitable alignment, the following procedure was used: First, the most distant pulling atoms were chosen as the main pulling pair, determining the force axis as the vector between the two atoms (red in Figure S128). Then, all possible combinations of pulling pairs using the remaining 22 pulling points were screened for alignment with this main pulling vector. Using maximum weight matching[44], six optimal pulling pairs which maximize the alignment with the force axis and the main pulling pair were identified, see Figure S128. RS-MD simulations of the $Pd_{12}L_{24}$ MOS were conducted using all seven aligned pulling pairs simultaneously (multi-pulling) to apply the external force to the system.

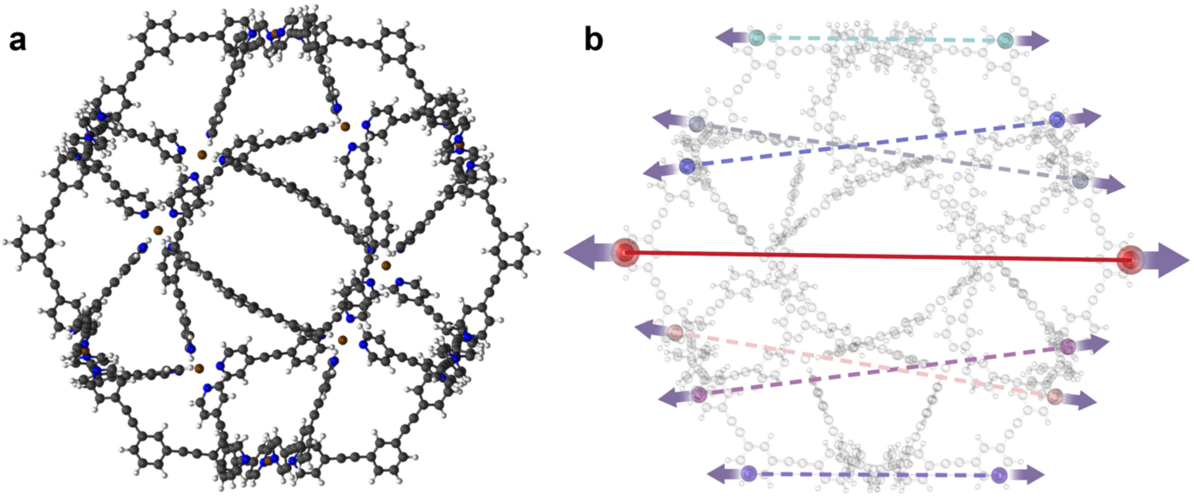

Figure S128: **a** Structure of the $Pd_{12}L_{24}$ MOS. **b** The main pulling axis is depicted as a solid red line, and the pulling pairs optimized through maximum weight matching are marked by dashed lines.

RS-MD simulations – setup

Explicit acetonitrile solvent was included in all RS-MD simulations of both structures. The cage structures were solvated using PackMol[45], with solvent placed both inside the cavity and in a microsolvation sphere around the systems. The number of solvent molecules was chosen to match the density of liquid acetonitrile (0.79 g/cm^3; ~12 molecules per nm^3) and is shown in Table S6. The inside of the cavity was defined by an inner radius r_{cav}, and the outside of the MOC was defined as a shell by two radii r_{inner} and r_{outer}, see Figures S129 and S130, respectively. The input structures generated from PackMol were allowed to thermalize for 20

ps (*i.e.*, 20,000 steps) of MD simulation time without any external force acting on the system before the ramping force was initialized.

Table S6: Radii used in the microsolvation of MOCs with PackMol, and number of of MeCN solvent molecules.

	r_{cav} (Å)	MeCN in cavity	r_{inner} (Å)	r_{outer} (Å)	MeCN around MOC	total no. of MeCN
Pd_2L_4	5	6	8.0	13.8	100	106
$Pd_{12}L_{24}$	17	230	19	25	300	530

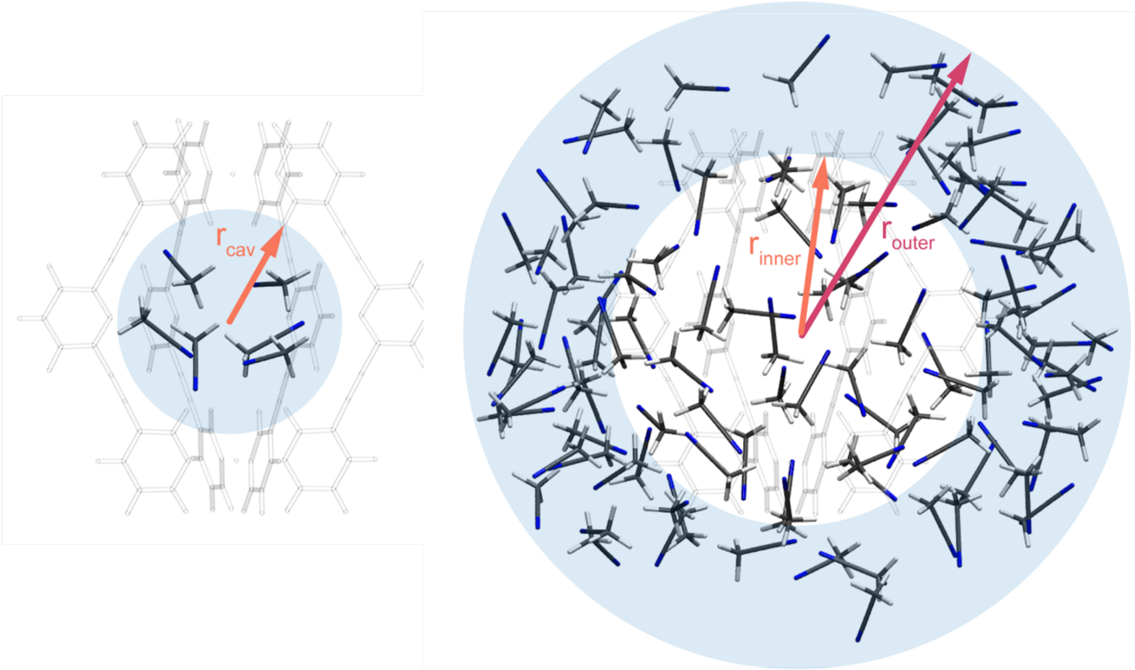

Figure S129: Placement of MeCN solvent molecules inside the cavity of Pd_2L_4 (left), and in a shell surrounding the cage structure (right).

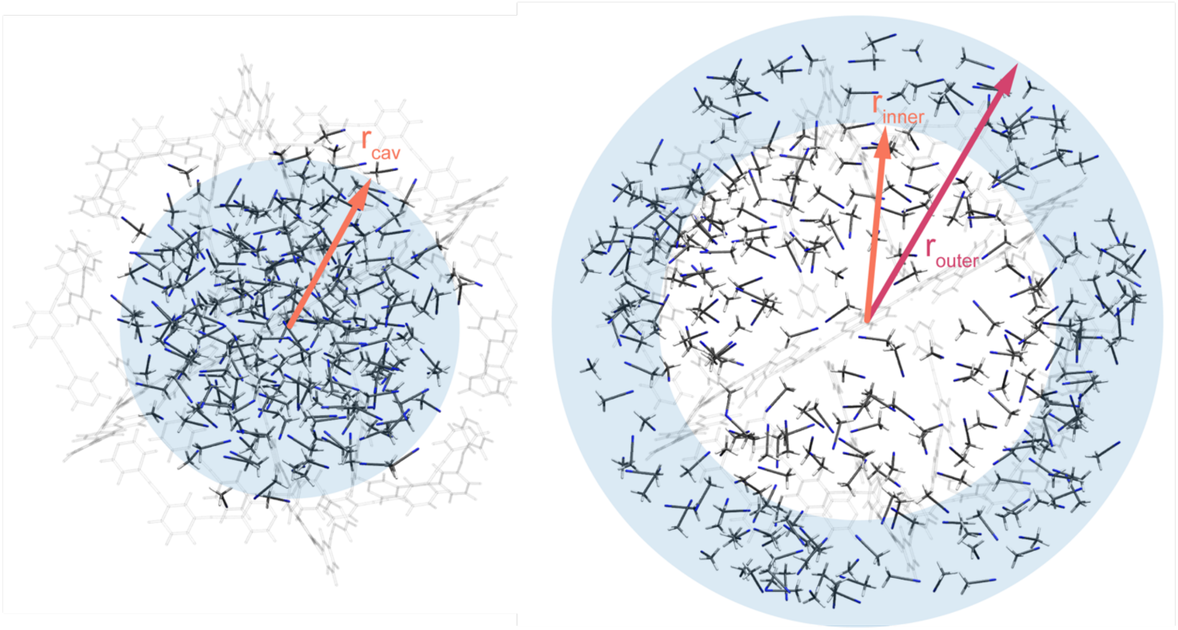

Figure S130: Placement of MeCN solvent molecules inside the cavity of $Pd_{12}L_{24}$ (left), and in a shell surrounding the cage structure (right).

RS-MD simulations with different ramping rates were performed to determine the onset force of the MOCs' mechanochemical decomposition. For the simulations of the disassembly of Pd_2L_4, the pulling geometries shown in Figure S127 were utilized, while for the examination of $Pd_{12}L_{24}$, the pulling mode illustrated in Figure S128 was used. Tables S7 and S8 contain details about the ramping rates $\dot{k}$, the maximum simulation time and the number of trajectories per utilized ramp for both cages.

Table S7: Settings of the RS-MD simulations scanning different ramping rates for Pd_2L_4. The maximum simulation time includes 20 ps of thermal equilibration before ramping of the external force was initialized.

Ramping rate $\dot{k}$ (pN/ps)	maximum simulation time (ps)	no. of trajectories
0.0	520	5
4.8	1020	10
9.6	520	10
10.2	520	10

Table S8: Settings of the RS-MD simulations investigating the multi-pulling mode of $Pd_{12}L_{24}$. The maximum simulation time includes 20 ps of thermal equilibration before ramping of the external force was initialized.

Ramping rate $\dot{k}^{\dagger}$ (pN/ps)	maximum simulation time (ps)	no. of trajectories
0.0	120	5
5.8	320	10
11.7	220	10
17.5	120	100

† applied per pulling pair

RS-MD simulations – results

Running simulations with these settings for all pulling geometries shown in Figure S103, it was possible to associate the dissociation forces F_D with the time of dissociation t_D for every RS-MD run. Note that only Pd–N bond breaks were observed and tracked by the bond distance using a criterion of 3.0 Å. Once this threshold is surpassed, a bond is considered broken. Here, only the first bond break is taken into account to determine the onset force and the value for t_D for the mechanochemical disassembly. The values of F_D and t_D are given in Tables S9-S12**.**

Table S9: Dissociation times and corresponding dissociation forces acting on the pulling atoms for **single *trans* pulling** on Pd_2L_4 for each force ramp.

Run#	$\dot{k}$ = 4.8 pN/ps		$\dot{k}$ = 9.6 pN/ps		$\dot{k}$ = 19.2 pN/ps	
	t_D§ (fs)	F_D (nN)	t_D§ (fs)	F_D (nN)	t_D§ (fs)	F_D (nN)
1	723569	3.48	355010	3.41	179593	3.45
2	726012	3.49	379055	3.65	186331	3.58
3	671999	3.23	383570	3.69	188239	3.62
4	677739	3.26	378134	3.64	200048	3.85
5	668590	3.21	318201	3.06	197038	3.79
6	651088	3.13	350444	3.37	176224	3.39
7	713938	3.43	382851	3.68	194168	3.73
8	689583	3.32	381553	3.67	192001	3.69
9	694124	3.34	345479	3.32	193834	3.73
10	697904	3.36	354557	3.41	184978	3.56
**Average**	691455	3.32	362885	3.49	189245	3.64

§ not including 20 ps of thermalization

Table S10: Dissociation times and corresponding dissociation forces acting on the pulling atoms for **single *cis* pulling** on Pd_2L_4 for each force ramp.

Run#	$\dot{k}$ = 4.8 pN/ps		$\dot{k}$ = 9.6 pN/ps		$\dot{k}$ = 19.2 pN/ps	
	t_D§ (fs)	F_D (nN)	t_D§ (fs)	F_D (nN)	t_D§ (fs)	F_D (nN)
1	564232	2.71	267679	2.57	160001	3.08
2	525439	2.53	321044	3.09	158740	3.05
3	524043	2.52	307297	2.96	152909	2.94
4	518966	2.50	315707	3.04	155135	2.98
5	576084	2.77	266600	2.56	136433	2.62
6	657133	3.16	303806	2.92	157135	3.02
7	587416	2.82	265564	2.55	154994	2.98
8	537929	2.59	299933	2.88	146166	2.81
9	581935	2.80	315740	3.04	151642	2.92
10	468269	2.25	287394	2.76	160333	3.08
**Average**	554145	2.66	295076	2.84	153349	2.95

§ not including 20 ps of thermalization

Table S11: Dissociation times and corresponding dissociation forces acting on the pulling atoms for **dual *trans* pulling** on Pd_2L_4 for each force ramp.

Run#	$\dot{k}$ = 4.8 pN/ps		$\dot{k}$ = 9.6 pN/ps		$\dot{k}$ = 19.2 pN/ps	
	t_D§ (fs)	F_D (nN)	t_D§ (fs)	F_D (nN)	t_D§ (fs)	F_D (nN)
1	510676	2.46	304274	2.93	195745	3.76
2	733988	3.53	309724	2.98	161063	3.10
3	602291	2.90	348723	3.35	165536	3.18
4	599711	2.88	319195	3.07	147407	2.84
5	645649	3.10	383119	3.68	180662	3.47
6	703113	3.38	355171	3.42	189550	3.65
7	739590	3.56	252568	2.43	138396	2.66
8	575067	2.77	321464	3.09	190260	3.66
9	585946	2.82	345757	3.33	158390	3.05
10	592231	2.85	335830	3.23	141731	2.73
**Average**	628826	3.02	327583	3.15	166874	3.21

§ not including 20 ps of thermalization

Table S12: Dissociation times and corresponding dissociation forces acting on the pulling atoms for **dual *cis* pulling** on Pd_2L_4 for each force ramp.

Run#	$\dot{k}$ = 4.8 pN/ps		$\dot{k}$ = 9.6 pN/ps		$\dot{k}$ = 19.2 pN/ps	
	t_D§ (fs)	F_D (nN)	t_D§ (fs)	F_D (nN)	t_D§ (fs)	F_D (nN)
1	365500	1.78	211868	2.04	115484	2.22
2	385055	1.85	252681	2.43	115661	2.22
3	410997	1.97	239574	2.30	120676	2.32
4	423902	2.04	255485	2.46	116161	2.23
5	411851	1.98	237365	2.28	124114	2.39
6	333243	1.60	225206	2.17	103382	1.99
7	427021	2.05	205677	1.98	106100	2.04
8	376034	1.81	187760	1.81	107944	2.08
9	399378	1.92	228496	2.20	134914	2.60
10	423664	2.04	236177	2.27	127137	2.45
**Average**	395665	1.90	228029	2.19	117157	2.25

§ not including 20 ps of thermalization

For the study of the $Pd_{12}L_{24}$ multi-pulling mode, forces were applied equally to the seven pulling pairs (see Figure S128). This multi-pulling likely provides a better model of the effect of forces induced by sonochemical cavitation events on the MOS, as it is unlikely that only a single polymer strand would be affected. The external force was applied per atom, so that the effective total force acting on the system becomes large enough to trigger reaction events. Hence, the values of F_D are difficult to interpret, as the resulting values appear rather small, but are applied to seven pulling pairs. The values of F_D and t_D are given in Table S13. Figure S131 shows the statistical distribution of the dissociation times t_D and the dissociation forces F_D of the RS-MD simulations of $Pd_{12}L_{24}$ using a force ramp of 17.5 pN · ps^{-1}.

Table S13: Dissociation times and corresponding dissociation forces acting on the pulling atoms for multiple pulling pairs for $Pd_{12}L_{24}$ for each force ramp.

Run#	$\dot{k}$ = 5.8 pN/ps		$\dot{k}$ = 11.7 pN/ps		$\dot{k}$ = 17.5 pN/ps	
	t_D§ (fs)	F_D (nN)	t_D§ (fs)	F_D (nN)	t_D§ (fs)	F_D (nN)
1	220135	1.28	96651	1.13	68396	1.20
2	191659	1.12	96932	1.13	69210	1.21
3	205282	1.20	107632	1.26	68642	1.20
4	182567	1.07	93490	1.09	72982	1.28

5	183053	1.07	106011	1.24	72837	1.28
6	209431	1.22	102448	1.20	70628	1.24
7	188086	1.10	102371	1.19	70393	1.23
8	197212	1.15	102803	1.20	64451	1.13
9	203532	1.19	98776	1.15	74239	1.30
10	192163	1.12	97700	1.14	72573	1.27
11	-	-	-	-	76498	1.34
12	-	-	-	-	62964	1.10
13	-	-	-	-	62581	1.10
14	-	-	-	-	69120	1.21
15	-	-	-	-	64148	1.12
16	-	-	-	-	68267	1.20
17	-	-	-	-	69556	1.22
18	-	-	-	-	73163	1.28
19	-	-	-	-	75369	1.32
20	-	-	-	-	60846	1.07
21	-	-	-	-	72275	1.27
22	-	-	-	-	66862	1.17
23	-	-	-	-	69488	1.22
24	-	-	-	-	68090	1.19
25	-	-	-	-	66719	1.17
26	-	-	-	-	65066	1.14
27	-	-	-	-	65708	1.15
28	-	-	-	-	65505	1.15
29	-	-	-	-	63358	1.11
30	-	-	-	-	70887	1.24
31	-	-	-	-	67688	1.19
32	-	-	-	-	69625	1.22
33	-	-	-	-	70279	1.23
34	-	-	-	-	66907	1.17
35	-	-	-	-	64230	1.12
36	-	-	-	-	63793	1.12
37	-	-	-	-	73318	1.28
38	-	-	-	-	70102	1.23

39	-	-	-	-	74307	1.30
40	-	-	-	-	64271	1.13
41	-	-	-	-	71791	1.26
42	-	-	-	-	67865	1.19
43	-	-	-	-	62240	1.09
44	-	-	-	-	75156	1.32
45	-	-	-	-	65530	1.15
46	-	-	-	-	70956	1.24
47	-	-	-	-	76864	1.35
48	-	-	-	-	63029	1.10
49	-	-	-	-	63607	1.11
50	-	-	-	-	74593	1.31
51	-	-	-	-	65666	1.15
52	-	-	-	-	68674	1.20
53	-	-	-	-	69593	1.22
54	-	-	-	-	66614	1.17
55	-	-	-	-	70638	1.24
56	-	-	-	-	72568	1.27
57	-	-	-	-	75355	1.32
58	-	-	-	-	75747	1.33
59	-	-	-	-	66192	1.16
60	-	-	-	-	64584	1.13
61	-	-	-	-	65020	1.14
62	-	-	-	-	63169	1.11
63	-	-	-	-	71872	1.26
64	-	-	-	-	73442	1.29
65	-	-	-	-	72954	1.28
66	-	-	-	-	68210	1.19
67	-	-	-	-	67994	1.19
68	-	-	-	-	68731	1.20
69	-	-	-	-	66461	1.16
70	-	-	-	-	72985	1.28
71	-	-	-	-	67161	1.18
72	-	-	-	-	75007	1.31

73	-	-	-	-	80716	1.41
74	-	-	-	-	76072	1.33
75	-	-	-	-	72491	1.27
76	-	-	-	-	68769	1.20
77	-	-	-	-	67644	1.18
78	-	-	-	-	71790	1.26
79	-	-	-	-	68206	1.19
80	-	-	-	-	73453	1.29
81	-	-	-	-	73955	1.29
82	-	-	-	-	74610	1.31
83	-	-	-	-	70038	1.23
84	-	-	-	-	61448	1.08
85	-	-	-	-	72258	1.27
86	-	-	-	-	74772	1.31
87	-	-	-	-	68510	1.20
88	-	-	-	-	73005	1.28
89	-	-	-	-	68284	1.20
90	-	-	-	-	76058	1.33
91	-	-	-	-	72832	1.28
92	-	-	-	-	72368	1.27
93	-	-	-	-	74022	1.30
94	-	-	-	-	71159	1.25
95	-	-	-	-	77829	1.36
96	-	-	-	-	70611	1.24
97	-	-	-	-	65508	1.15
98	-	-	-	-	71841	1.26
99	-	-	-	-	70618	1.24
100	-	-	-	-	73692	1.29
**Average**	197312	1.15	100481	1.17	69782	1.22

§ not including 20 ps of thermalization

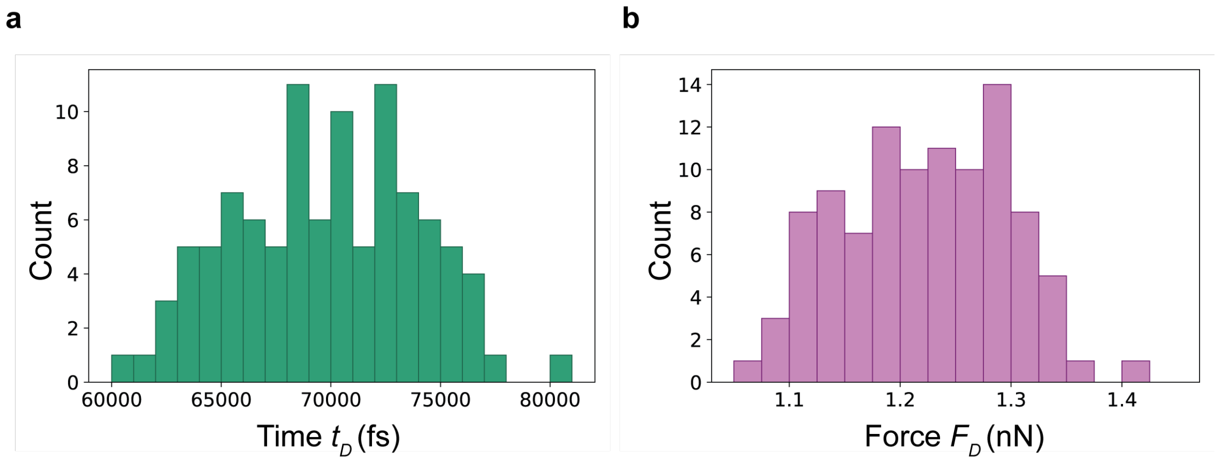

Figure S131: **a** Statistical distribution of the dissociation time t_D for the RS-MD simulations of $Pd_{12}L_{24}$. **b** statistical distribution of the dissociation force F_D for the RS-MD simulations of $Pd_{12}L_{24}$.

Kinetic analysis

In the RS-MD-approach, the external force $F(t)$ increases linearly in time with $F(t) = \dot{k} \cdot t$ (Eq. S13), where t is the time and $\dot{k}$ represents the force ramp. Assuming that the free activation energy $\Delta G^{\ddagger}$ decreases linearly with increasing force for the first bond breaking event, the force-dependent reaction rate for a trajectory can be approximated by

$$k(F) = A \cdot e^{-\frac{\Delta G^{\ddagger}(0) - F \cdot x}{RT}} = k_0 \cdot e^{\frac{F \cdot x}{RT}}, \tag{S14}$$

Where A is the pre-exponential factor, k_0 is the effective zero-force rate constant, x is the effective distance from the reactant to the transition state along the pulling coordinate, R is the universal gas constant and T is the temperature. Inserting the linear ramp (Eq. S13) gives a time-dependent rate.

$$k(t) = k_0 \cdot e^{\frac{\dot{k}x}{RT} \cdot t} = k_0 \cdot e^{bt} \tag{S15}$$

with

$$b = \frac{\dot{k}x}{RT}. \tag{S16}$$

The survival probability $S(t)$, i.e. the probability that a cage has not dissociated up to time t, can be expressed as

$$S(t) = \exp\left(-\frac{k_0}{b}\left(e^{bt} - 1\right)\right). \tag{S17}$$

The corresponding dissociation time probability density $f(t)$ is

$$f(t) = -\frac{dS}{dt} = k(t) \cdot S(t) = k_0 \cdot e^{bt} \cdot \exp\left(-\frac{k_0}{b}\left(e^{bt} - 1\right)\right) \tag{S18}$$

In our analysis, the parameters k_0 and x are fitted to the dissociation time statistics obtained from the RS-MD trajectories using this survival model. We estimate k_0 and x by the maximum likelihood method, i.e., by selecting the parameter values that best reflect the actual detected failure and survival times under the model. For each trajectory i, we record whether a bond break occurs and at what time. If a dissociation is detected, the time t_i enters the likelihood as an event; if no dissociation occurs within the simulation window, the total observation time is treated as a right-censored data point. The total (log-)likelihood is the sum over all trajectories and the parameters k_0 and x are obtained by maximizing the corresponding (censored) likelihood using the L-BFGS-B optimizer as implemented in SciPy[46].

For Pd_2L_4 with four different pulling geometries, we perform a joint "shared k_0" fit, in which a single global k_0 is fitted across all geometries, while each pulling geometry g has its own x_g. This reflects the assumption that all pulling protocols probe the same underlying bond-breaking process with the same zero-force barrier $\Delta G^{\ddagger}(0)$ and that the different pulling geometries mainly change how strongly the barrier is coupled with the external force. For the $Pd_{12}L_{24}$ MOS, only one pulling geometry is present, so a single pair of k_0 and x is fitted. Figure S132 shows the fitted survival probability of the cages in comparison to the empirical data obtained from the trajectories. Table S14 shows the fitted parameters k_0 and x for both the Pd_2L_4 and $Pd_{12}L_{24}$.

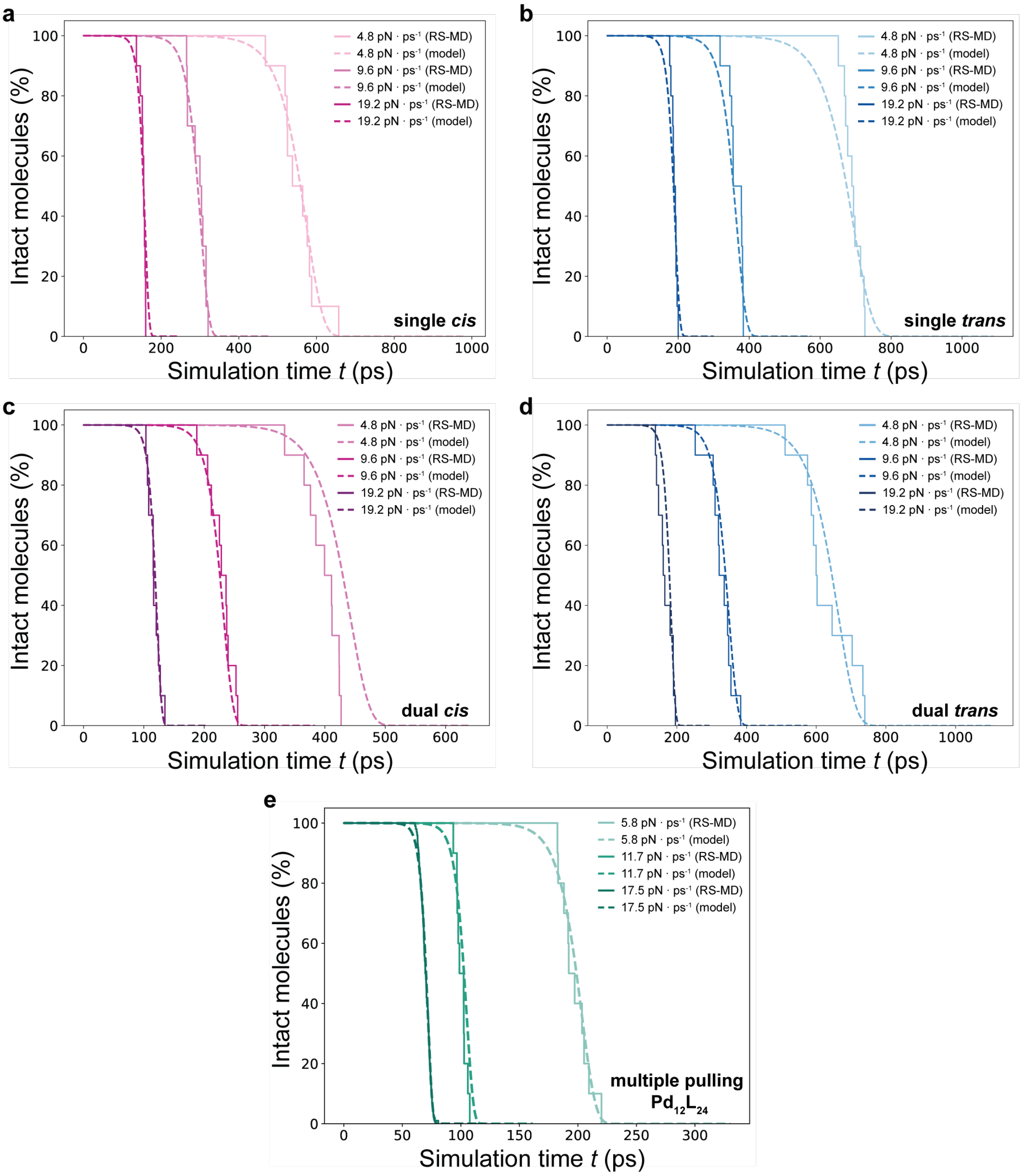

Figure S132: Survival of cage molecules under different pulling geometries and loading rates. Shown is the fraction of intact molecules as a function of simulation time obtained from RS-MD simulations and the corresponding modelled survival curves. **a** Single *cis* pulling on Pd_2L_4. **b** Single *trans* pulling on Pd_2L_4. **c** Dual *cis* pulling on Pd_2L_4. **d** Dual *trans* pulling on Pd_2L_4. **e** Multi-pulling on $Pd_{12}L_{24}$. Solid lines denote empirical simulation data, dashed lines the model prediction. Loading rates are indicated (in pN · ps^{-1}) in the legends.

Table S12: Fitted parameters k_0 and x for Pd_2L_4 and $Pd_{12}L_{24}$.

Cage	Pulling geometry	k_0 (ps^{-1})	x (Å)	$\frac{d\Delta G^{\ddagger}}{dF}$ (kcal · mol^{-1} · nN^{-1})
Pd_2L_4	single *cis*	$3.56 \cdot 10^{-8}$	0.20	-2.90
Pd_2L_4	single *trans*	$3.56 \cdot 10^{-8}$	0.16	-2.36
Pd_2L_4	dual *cis*	$3.56 \cdot 10^{-8}$	0.27	-3.85
Pd_2L_4	dual *trans*	$3.56 \cdot 10^{-8}$	0.17	-2.48
$Pd_{12}L_{24}$	multiple pulling	$3.53 \cdot 10^{-9}$	0.60	-8.59

Fragment analysis

The fragments resulting from the mechanochemical destruction of the two MOCs were analyzed by detecting Pd–N bond scission events during the RS-MD simulations. The resulting fragments were classified according to their number of Pd atoms, Ligands, Pd-N bonds, and their connectivity. Only Pd–N bond scission reactions were considered in this analysis. A Pd–N bond is considered broken once the distance between the two atoms surpasses 3 Å.

The results for the different pulling modes of Pd_2L_4 are presented in Tables S15 to S18, and those of $Pd_{12}L_{24}$ are listed in Table S19. Figure S133 shows the evolution of the occurring fragments over time for $Pd_{12}L_{24}$. Figure S134 gives closer insights on the evolution of Fragments containing 10 and 11 Pd atoms.

Table S15: List of unique fragments and number of their occurrence in the end of the RS-MD simulations for **single *cis* pulling** on Pd_2L_4 for each force ramp.

force ramp (pN/ps)	Pd_2L_3, N_{Pd-N}: 6	Pd_2L_2, N_{Pd-N}: 4	Pd_2L_2, N_{Pd-N}: 3	Ligand
4.8	3	6	1	17
9.6	6	4	0	14
19.2	0	7	3	20

Table S16: List of unique fragments and number of their occurrence in the end of the RS-MD simulations for **single *trans* pulling** on Pd_2L_4 for each force ramp.

force ramp (pN/ps)	Pd_2L_2, N_{Pd-N}: 4	Pd_2L_2, N_{Pd-N}: 3	Ligand
4.8	8	2	20
9.6	10	0	20
19.2	9	1	20

Table S17: List of unique fragments and number of their occurrence in the end of the RS-MD simulations for **dual *cis* pulling** on Pd_2L_4 for each force ramp.

**force ramp (pN/ps)**	**Pd_2L_2, $N_{Pd\text{-}N}$: 4**	**Pd_2L_1, $N_{Pd\text{-}N}$: 2**	**Pd_1L_2, $N_{Pd\text{-}N}$: 2**	**Pd_1L_1, $N_{Pd\text{-}N}$: 1**	**Ligand**
4.8	5	1	4	1	20
9.6	5	0	5	1	19
19.2	0	0	0	0	40

Table S18: List of unique fragments and number of their occurrence in the end of the RS-MD simulations for **dual *trans* pulling** on Pd_2L_4 for each force ramp.

**force ramp (pN/ps)**	**Pd_2L_2, $N_{Pd\text{-}N}$: 4**	**Pd_2L_1, $N_{Pd\text{-}N}$: 2**	**Pd_1L_2, $N_{Pd\text{-}N}$: 2**	**Pd_1L_1, $N_{Pd\text{-}N}$: 1**	**Ligand**
4.8	2	1	2	6	25
9.6	1	2	0	0	36
19.2	0	0	0	0	40

Table S19: List of unique fragments and number of their occurrence in the RS-MD simulations of the $Pd_{12}L_{24}$ MOC with a force ramping rate of 17.5 pN/ps.

**fragment type**	**count**	**fragment type**	**count**	**fragment type**	**count**
$Pd_{12}L_{24}$, $N_{Pd\text{-}N}$: 48 (MOS)	100	$Pd_{11}L_{22}$, $N_{Pd\text{-}N}$: 34	1	Pd_9L_{18}, $N_{Pd\text{-}N}$: 29	1
$Pd_{12}L_{24}$, $N_{Pd\text{-}N}$: 47	100	$Pd_{11}L_{22}$, $N_{Pd\text{-}N}$: 33	2	Pd_9L_{17}, $N_{Pd\text{-}N}$: 26	2
$Pd_{12}L_{24}$, $N_{Pd\text{-}N}$: 46	95	$Pd_{11}L_{21}$, $N_{Pd\text{-}N}$: 39	1	Pd_9L_{16}, $N_{Pd\text{-}N}$: 28	1
$Pd_{12}L_{24}$, $N_{Pd\text{-}N}$: 45	80	$Pd_{11}L_{21}$, $N_{Pd\text{-}N}$: 38	2	Pd_9L_{16}, $N_{Pd\text{-}N}$: 27	3
$Pd_{12}L_{24}$, $N_{Pd\text{-}N}$: 44	62	$Pd_{11}L_{21}$, $N_{Pd\text{-}N}$: 37	2	Pd_9L_{16}, $N_{Pd\text{-}N}$: 26	4
$Pd_{12}L_{24}$, $N_{Pd\text{-}N}$: 43	38	$Pd_{11}L_{21}$, $N_{Pd\text{-}N}$: 36	2	Pd_9L_{16}, $N_{Pd\text{-}N}$: 25	1
$Pd_{12}L_{24}$, $N_{Pd\text{-}N}$: 42	20	$Pd_{11}L_{21}$, $N_{Pd\text{-}N}$: 35	1	Pd_9L_{15}, $N_{Pd\text{-}N}$: 28	1
$Pd_{12}L_{24}$, $N_{Pd\text{-}N}$: 41	15	$Pd_{11}L_{21}$, $N_{Pd\text{-}N}$: 34	2	Pd_9L_{15}, $N_{Pd\text{-}N}$: 27	1
$Pd_{12}L_{24}$, $N_{Pd\text{-}N}$: 40	9	$Pd_{11}L_{21}$, $N_{Pd\text{-}N}$: 33	1	Pd_9L_{15}, $N_{Pd\text{-}N}$: 26	3
$Pd_{12}L_{24}$, $N_{Pd\text{-}N}$: 39	4	$Pd_{11}L_{21}$, $N_{Pd\text{-}N}$: 32	2	Pd_9L_{15}, $N_{Pd\text{-}N}$: 25	7
$Pd_{12}L_{24}$, $N_{Pd\text{-}N}$: 38	1	$Pd_{11}L_{20}$, $N_{Pd\text{-}N}$: 36	5	Pd_9L_{15}, $N_{Pd\text{-}N}$: 24	6
$Pd_{12}L_{24}$, $N_{Pd\text{-}N}$: 37	1	$Pd_{11}L_{20}$, $N_{Pd\text{-}N}$: 35	8	Pd_9L_{15}, $N_{Pd\text{-}N}$: 23	2
$Pd_{12}L_{24}$, $N_{Pd\text{-}N}$: 36	1	$Pd_{11}L_{20}$, $N_{Pd\text{-}N}$: 34	9	Pd_9L_{14}, $N_{Pd\text{-}N}$: 26	1
$Pd_{12}L_{23}$, $N_{Pd\text{-}N}$: 46	5	$Pd_{11}L_{20}$, $N_{Pd\text{-}N}$: 33	9	Pd_9L_{14}, $N_{Pd\text{-}N}$: 25	5
$Pd_{12}L_{23}$, $N_{Pd\text{-}N}$: 45	20	$Pd_{11}L_{20}$, $N_{Pd\text{-}N}$: 32	5	Pd_9L_{14}, $N_{Pd\text{-}N}$: 24	5
$Pd_{12}L_{23}$, $N_{Pd\text{-}N}$: 44	33	$Pd_{11}L_{20}$, $N_{Pd\text{-}N}$: 31	1	Pd_9L_{14}, $N_{Pd\text{-}N}$: 23	8
$Pd_{12}L_{23}$, $N_{Pd\text{-}N}$: 43	47	$Pd_{11}L_{19}$, $N_{Pd\text{-}N}$: 36	1	Pd_9L_{13}, $N_{Pd\text{-}N}$: 24	2
$Pd_{12}L_{23}$, $N_{Pd\text{-}N}$: 42	57	$Pd_{11}L_{19}$, $N_{Pd\text{-}N}$: 35	4	Pd_9L_{13}, $N_{Pd\text{-}N}$: 23	6
$Pd_{12}L_{23}$, $N_{Pd\text{-}N}$: 41	47	$Pd_{11}L_{19}$, $N_{Pd\text{-}N}$: 34	7	Pd_9L_{13}, $N_{Pd\text{-}N}$: 22	7
$Pd_{12}L_{23}$, $N_{Pd\text{-}N}$: 40	35	$Pd_{11}L_{19}$, $N_{Pd\text{-}N}$: 33	10	Pd_9L_{13}, $N_{Pd\text{-}N}$: 21	1
$Pd_{12}L_{23}$, $N_{Pd\text{-}N}$: 39	31	$Pd_{11}L_{19}$, $N_{Pd\text{-}N}$: 32	15	Pd_9L_{12}, $N_{Pd\text{-}N}$: 22	2

$Pd_{12}L_{23}$, $N_{Pd\text{-}N}$: 38	23	$Pd_{11}L_{19}$, $N_{Pd\text{-}N}$: 31	20	$Pd_{9}L_{12}$, $N_{Pd\text{-}N}$: 21	1
$Pd_{12}L_{23}$, $N_{Pd\text{-}N}$: 37	13	$Pd_{11}L_{19}$, $N_{Pd\text{-}N}$: 30	11	$Pd_{8}L_{15}$, $N_{Pd\text{-}N}$: 27	1
$Pd_{12}L_{23}$, $N_{Pd\text{-}N}$: 36	7	$Pd_{11}L_{19}$, $N_{Pd\text{-}N}$: 29	2	$Pd_{8}L_{15}$, $N_{Pd\text{-}N}$: 24	1
$Pd_{12}L_{23}$, $N_{Pd\text{-}N}$: 35	3	$Pd_{11}L_{18}$, $N_{Pd\text{-}N}$: 34	4	$Pd_{8}L_{14}$, $N_{Pd\text{-}N}$: 22	1
$Pd_{12}L_{22}$, $N_{Pd\text{-}N}$: 44	5	$Pd_{11}L_{18}$, $N_{Pd\text{-}N}$: 33	4	$Pd_{8}L_{13}$, $N_{Pd\text{-}N}$: 22	3
$Pd_{12}L_{22}$, $N_{Pd\text{-}N}$: 43	15	$Pd_{11}L_{18}$, $N_{Pd\text{-}N}$: 32	9	$Pd_{8}L_{13}$, $N_{Pd\text{-}N}$: 21	8
$Pd_{12}L_{22}$, $N_{Pd\text{-}N}$: 42	20	$Pd_{11}L_{18}$, $N_{Pd\text{-}N}$: 31	13	$Pd_{8}L_{13}$, $N_{Pd\text{-}N}$: 20	2
$Pd_{12}L_{22}$, $N_{Pd\text{-}N}$: 41	32	$Pd_{11}L_{18}$, $N_{Pd\text{-}N}$: 30	13	$Pd_{8}L_{12}$, $N_{Pd\text{-}N}$: 21	2
$Pd_{12}L_{22}$, $N_{Pd\text{-}N}$: 40	39	$Pd_{11}L_{18}$, $N_{Pd\text{-}N}$: 29	4	$Pd_{8}L_{12}$, $N_{Pd\text{-}N}$: 20	1
$Pd_{12}L_{22}$, $N_{Pd\text{-}N}$: 39	41	$Pd_{11}L_{18}$, $N_{Pd\text{-}N}$: 28	1	$Pd_{8}L_{11}$, $N_{Pd\text{-}N}$: 20	2
$Pd_{12}L_{22}$, $N_{Pd\text{-}N}$: 38	40	$Pd_{11}L_{17}$, $N_{Pd\text{-}N}$: 29	5	$Pd_{8}L_{11}$, $N_{Pd\text{-}N}$: 19	1
$Pd_{12}L_{22}$, $N_{Pd\text{-}N}$: 37	39	$Pd_{10}L_{21}$, $N_{Pd\text{-}N}$: 33	1	$Pd_{8}L_{10}$, $N_{Pd\text{-}N}$: 20	4
$Pd_{12}L_{22}$, $N_{Pd\text{-}N}$: 36	33	$Pd_{10}L_{20}$, $N_{Pd\text{-}N}$: 31	1	$Pd_{8}L_{10}$, $N_{Pd\text{-}N}$: 19	1
$Pd_{12}L_{22}$, $N_{Pd\text{-}N}$: 35	23	$Pd_{10}L_{20}$, $N_{Pd\text{-}N}$: 30	2	$Pd_{7}L_{12}$, $N_{Pd\text{-}N}$: 20	1
$Pd_{12}L_{22}$, $N_{Pd\text{-}N}$: 34	6	$Pd_{10}L_{19}$, $N_{Pd\text{-}N}$: 32	3	$Pd_{7}L_{12}$, $N_{Pd\text{-}N}$: 19	1
$Pd_{12}L_{21}$, $N_{Pd\text{-}N}$: 42	2	$Pd_{10}L_{19}$, $N_{Pd\text{-}N}$: 31	3	$Pd_{7}L_{9}$, $N_{Pd\text{-}N}$: 17	3
$Pd_{12}L_{21}$, $N_{Pd\text{-}N}$: 41	4	$Pd_{10}L_{19}$, $N_{Pd\text{-}N}$: 30	5	$Pd_{7}L_{9}$, $N_{Pd\text{-}N}$: 16	4
$Pd_{12}L_{21}$, $N_{Pd\text{-}N}$: 40	11	$Pd_{10}L_{19}$, $N_{Pd\text{-}N}$: 29	4	$Pd_{7}L_{9}$, $N_{Pd\text{-}N}$: 15	2
$Pd_{12}L_{21}$, $N_{Pd\text{-}N}$: 39	14	$Pd_{10}L_{19}$, $N_{Pd\text{-}N}$: 28	1	$Pd_{6}L_{8}$, $N_{Pd\text{-}N}$: 14	1
$Pd_{12}L_{21}$, $N_{Pd\text{-}N}$: 38	21	$Pd_{10}L_{18}$, $N_{Pd\text{-}N}$: 31	2	$Pd_{5}L_{9}$, $N_{Pd\text{-}N}$: 15	1
$Pd_{12}L_{21}$, $N_{Pd\text{-}N}$: 37	20	$Pd_{10}L_{18}$, $N_{Pd\text{-}N}$: 30	2	$Pd_{5}L_{9}$, $N_{Pd\text{-}N}$: 14	1
$Pd_{12}L_{21}$, $N_{Pd\text{-}N}$: 36	19	$Pd_{10}L_{18}$, $N_{Pd\text{-}N}$: 29	2	$Pd_{5}L_{9}$, $N_{Pd\text{-}N}$: 13	1
$Pd_{12}L_{21}$, $N_{Pd\text{-}N}$: 35	14	$Pd_{10}L_{18}$, $N_{Pd\text{-}N}$: 28	3	$Pd_{4}L_{9}$, $N_{Pd\text{-}N}$: 13	1
$Pd_{12}L_{21}$, $N_{Pd\text{-}N}$: 34	7	$Pd_{10}L_{17}$, $N_{Pd\text{-}N}$: 30	1	$Pd_{4}L_{9}$, $N_{Pd\text{-}N}$: 12	1
$Pd_{12}L_{21}$, $N_{Pd\text{-}N}$: 33	2	$Pd_{10}L_{17}$, $N_{Pd\text{-}N}$: 29	6	$Pd_{4}L_{5}$, $N_{Pd\text{-}N}$: 9	1
$Pd_{12}L_{21}$, $N_{Pd\text{-}N}$: 32	1	$Pd_{10}L_{17}$, $N_{Pd\text{-}N}$: 28	4	$Pd_{3}L_{7}$, $N_{Pd\text{-}N}$: 9	4
$Pd_{12}L_{20}$, $N_{Pd\text{-}N}$: 40	1	$Pd_{10}L_{17}$, $N_{Pd\text{-}N}$: 27	4	$Pd_{3}L_{6}$, $N_{Pd\text{-}N}$: 9	1
$Pd_{12}L_{20}$, $N_{Pd\text{-}N}$: 39	1	$Pd_{10}L_{17}$, $N_{Pd\text{-}N}$: 26	2	$Pd_{3}L_{6}$, $N_{Pd\text{-}N}$: 8	4
$Pd_{12}L_{20}$, $N_{Pd\text{-}N}$: 38	1	$Pd_{10}L_{16}$, $N_{Pd\text{-}N}$: 30	3	$Pd_{3}L_{5}$, $N_{Pd\text{-}N}$: 7	2
$Pd_{12}L_{20}$, $N_{Pd\text{-}N}$: 37	2	$Pd_{10}L_{16}$, $N_{Pd\text{-}N}$: 29	4	$Pd_{2}L_{7}$, $N_{Pd\text{-}N}$: 8	3
$Pd_{12}L_{20}$, $N_{Pd\text{-}N}$: 36	5	$Pd_{10}L_{16}$, $N_{Pd\text{-}N}$: 28	8	$Pd_{2}L_{6}$, $N_{Pd\text{-}N}$: 7	6
$Pd_{12}L_{20}$, $N_{Pd\text{-}N}$: 35	5	$Pd_{10}L_{16}$, $N_{Pd\text{-}N}$: 27	13	$Pd_{2}L_{5}$, $N_{Pd\text{-}N}$: 6	9
$Pd_{12}L_{20}$, $N_{Pd\text{-}N}$: 34	6	$Pd_{10}L_{16}$, $N_{Pd\text{-}N}$: 26	10	$Pd_{2}L_{4}$, $N_{Pd\text{-}N}$: 5	27
$Pd_{12}L_{20}$, $N_{Pd\text{-}N}$: 33	3	$Pd_{10}L_{16}$, $N_{Pd\text{-}N}$: 25	1	$Pd_{2}L_{3}$, $N_{Pd\text{-}N}$: 4	3
$Pd_{12}L_{20}$, $N_{Pd\text{-}N}$: 32	3	$Pd_{10}L_{15}$, $N_{Pd\text{-}N}$: 28	1	$Pd_{1}L_{4}$, $N_{Pd\text{-}N}$: 4	24
$Pd_{12}L_{20}$, $N_{Pd\text{-}N}$: 31	1	$Pd_{10}L_{15}$, $N_{Pd\text{-}N}$: 27	6	$Pd_{1}L_{3}$, $N_{Pd\text{-}N}$: 3	145
$Pd_{12}L_{19}$, $N_{Pd\text{-}N}$: 33	1	$Pd_{10}L_{15}$, $N_{Pd\text{-}N}$: 26	3	$Pd_{1}L_{2}$, $N_{Pd\text{-}N}$: 2	7
$Pd_{12}L_{19}$, $N_{Pd\text{-}N}$: 32	2	$Pd_{10}L_{15}$, $N_{Pd\text{-}N}$: 25	3	$Pd_{1}L_{1}$, $N_{Pd\text{-}N}$: 1	7
$Pd_{12}L_{19}$, $N_{Pd\text{-}N}$: 31	1	$Pd_{10}L_{15}$, $N_{Pd\text{-}N}$: 24	1	Ligand	237
$Pd_{12}L_{18}$, $N_{Pd\text{-}N}$: 30	1	$Pd_{10}L_{14}$, $N_{Pd\text{-}N}$: 24	1		

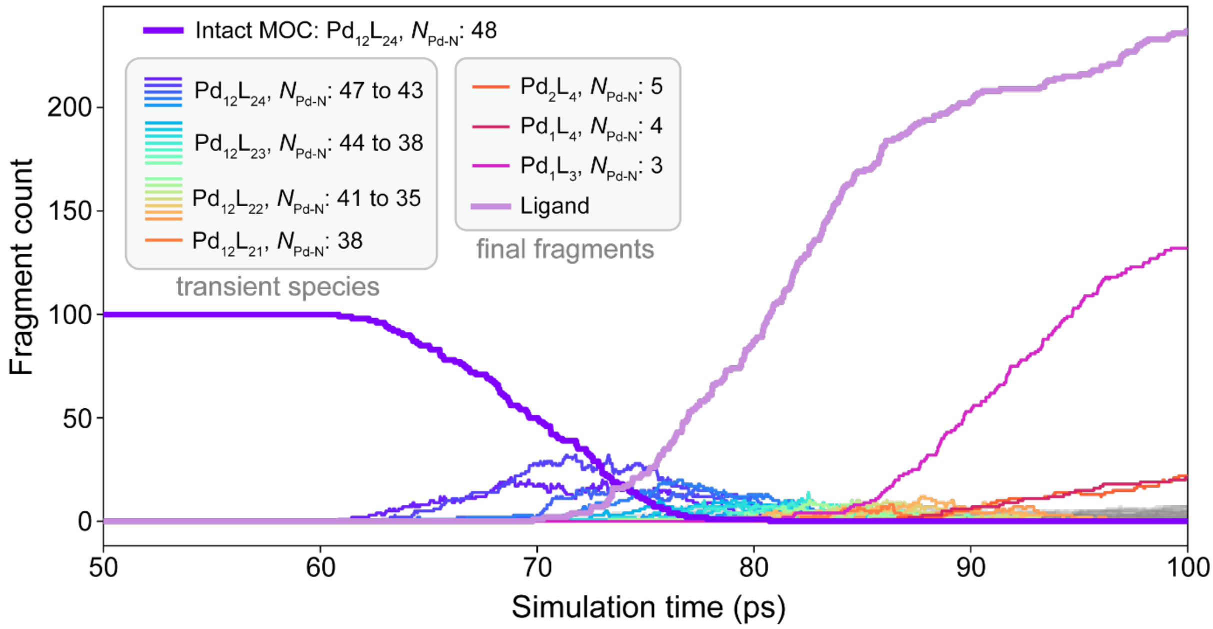

Figure S133: Fragmentation of $Pd_{12}L_{24}$ over time during RS-MD simulations using a force ramp of 17.5 pN · ps^{-1}. Fragments that occur less than twenty times in the 100 simulations are marked in grey. $N_{Pd–N}$ indicates the number of Pd–N bonds in the fragment.

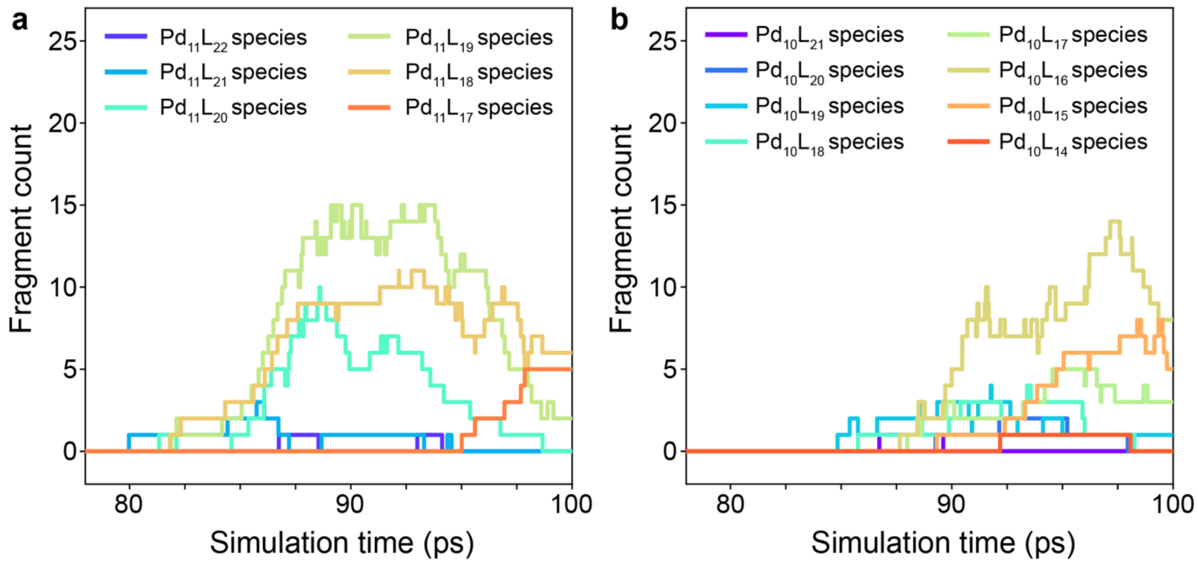

Figure S134: Fragmentation of $Pd_{12}L_{24}$ over time during RS-MD simulations using a force ramp of 17.5 pN · ps^{-1}. **a** Fragments containing 11 Pd atoms, **b** fragments containing 10 Pd atoms.

XIII. Spectra

Synthesis

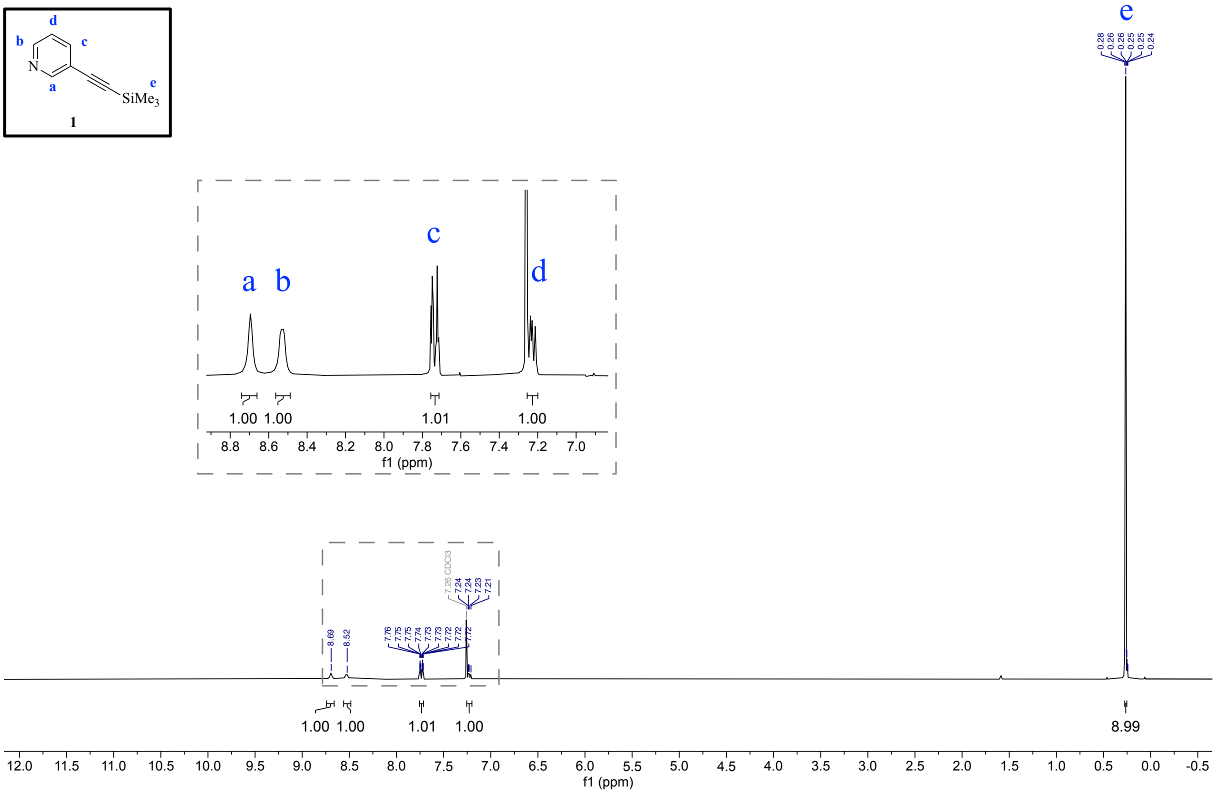

Figure S135: ^{1}H NMR spectrum (300 MHz, $CDCl_3$, 298 K) of **1**.

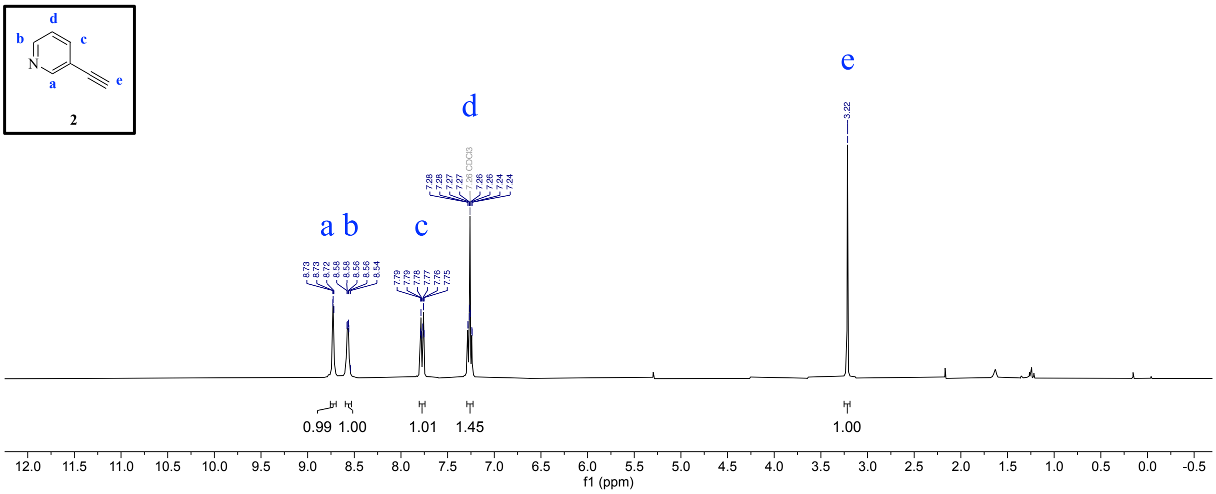

Figure S136: ^{1}H NMR spectrum (300 MHz, $CDCl_3$, 298 K) of **2**.

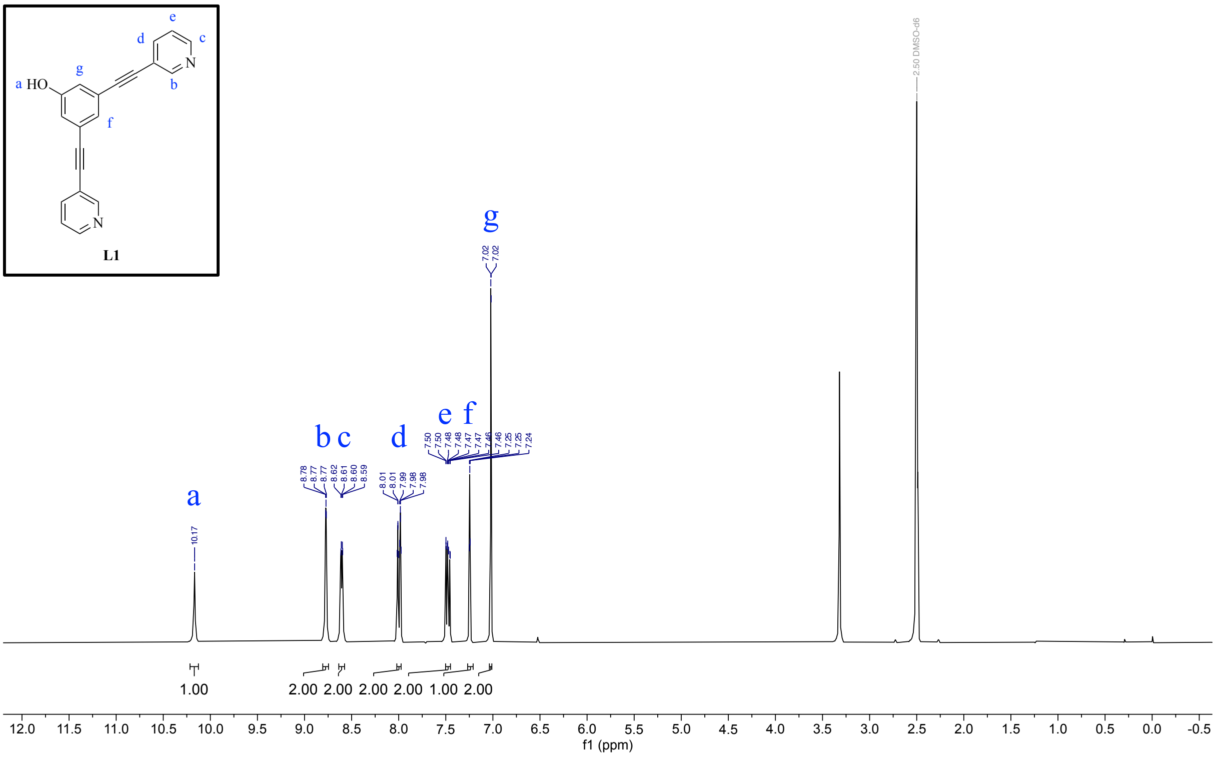

Figure S137: ^{1}H NMR spectrum (300 MHz, DMSO-d6, 298 K) of **L1**.

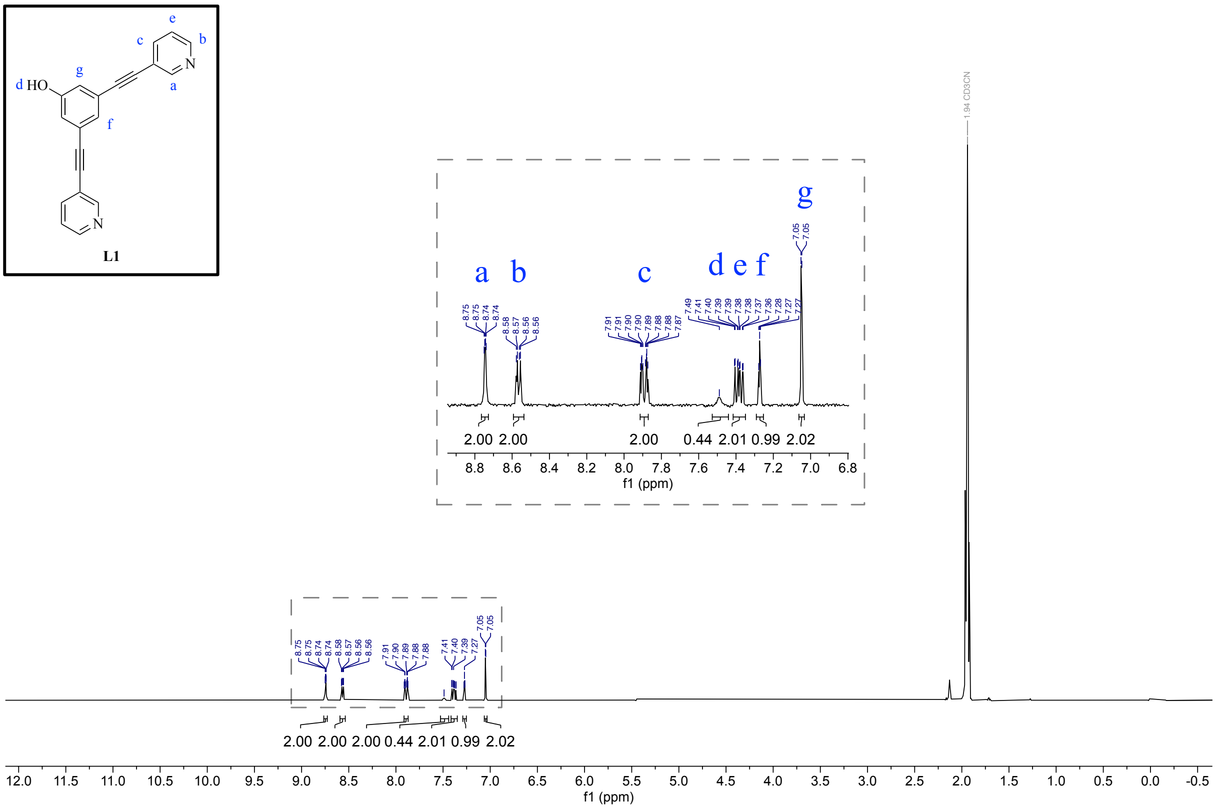

Figure S138: ^{1}H NMR spectrum (300 MHz, CD_3CN, 298 K) of **L1**.

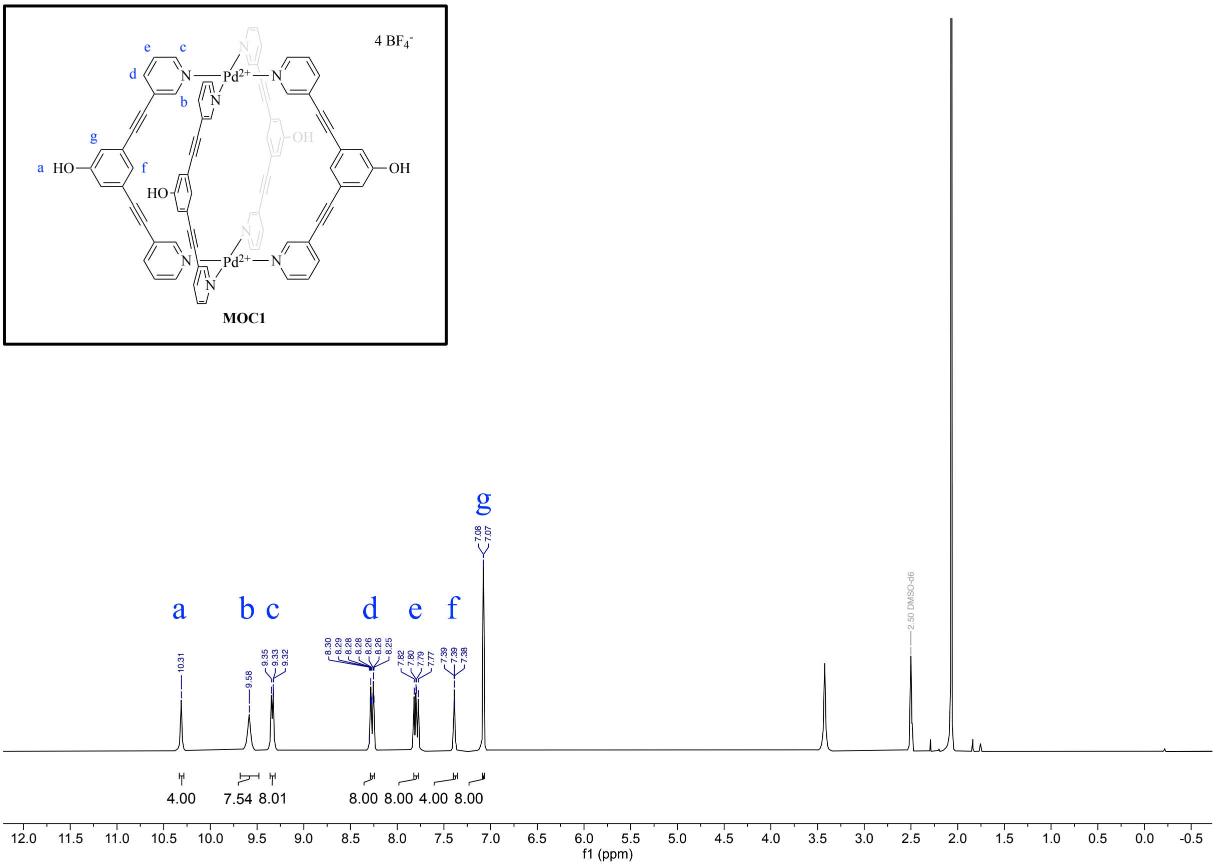

Figure S139: ^{1}H NMR spectrum (300 MHz, DMSO-d6, 298 K) of **MOC1**.

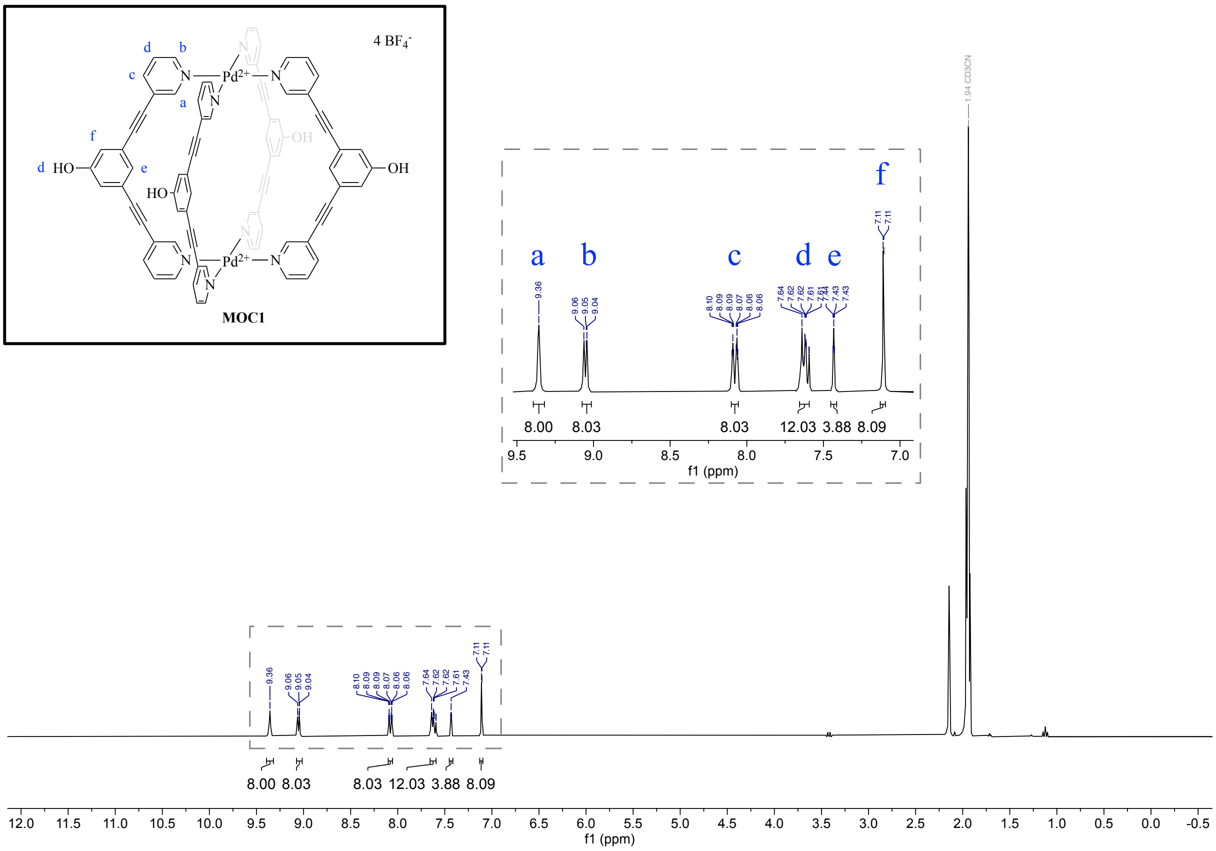

Figure S140: ^{1}H NMR spectrum (300 MHz, CD_3CN, 298 K) of **MOC1**.

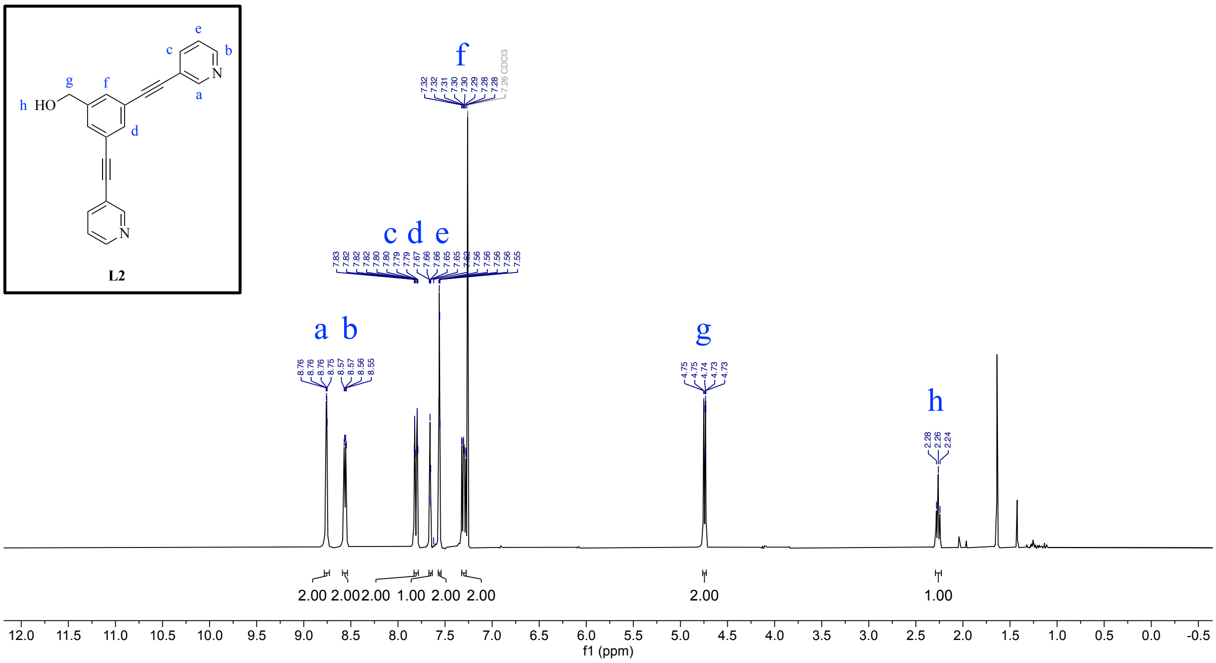

Figure S141: ^{1}H NMR spectrum (300 MHz, $CDCl_3$, 298 K) of **L2**.

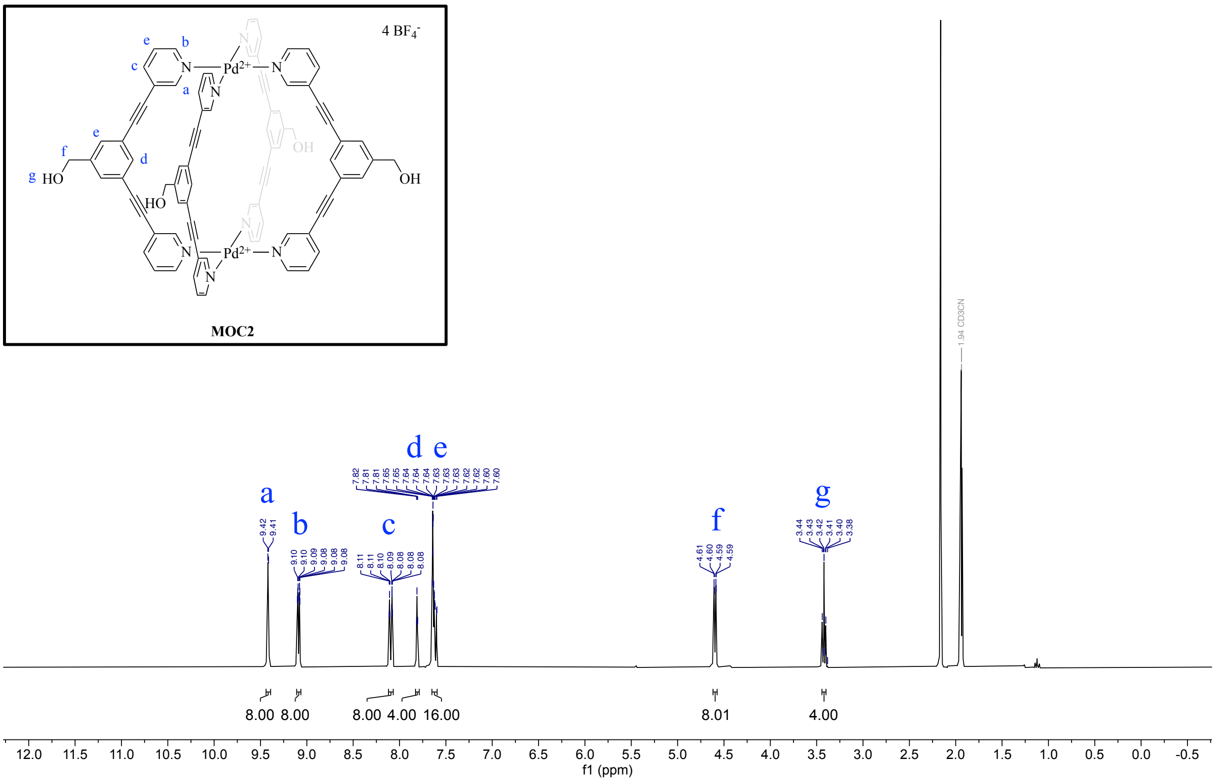

Figure S142: ^{1}H NMR spectrum (300 MHz, CD_3CN, 298 K) of **MOC2**.

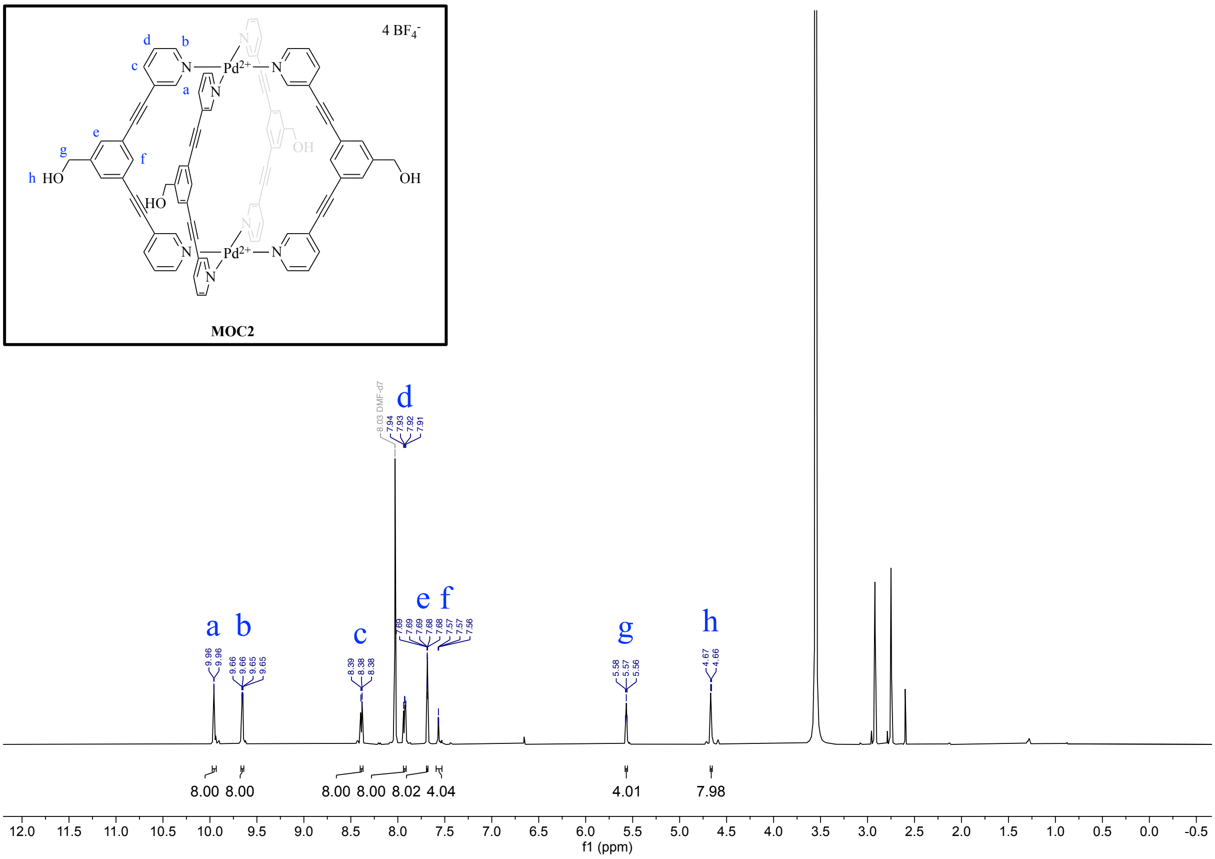

Figure S143: ^{1}H NMR spectrum (300 MHz, DMF-d7, 298 K) of **MOC2**.

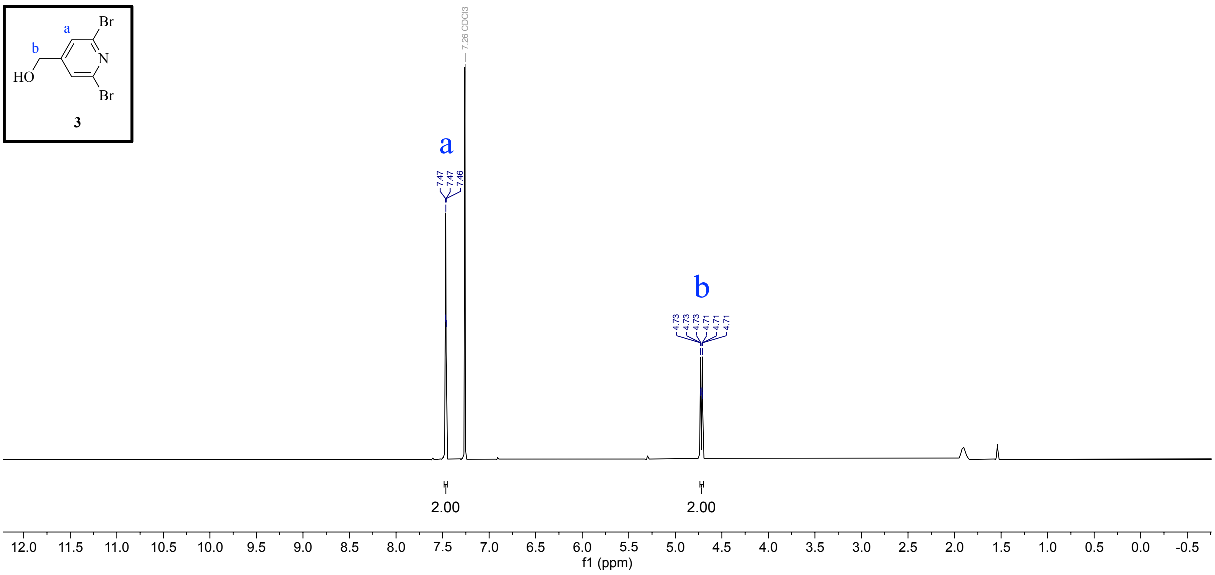

Figure S144: ^{1}H NMR spectrum (300 MHz, $CDCl_3$, 298 K) of **3**.

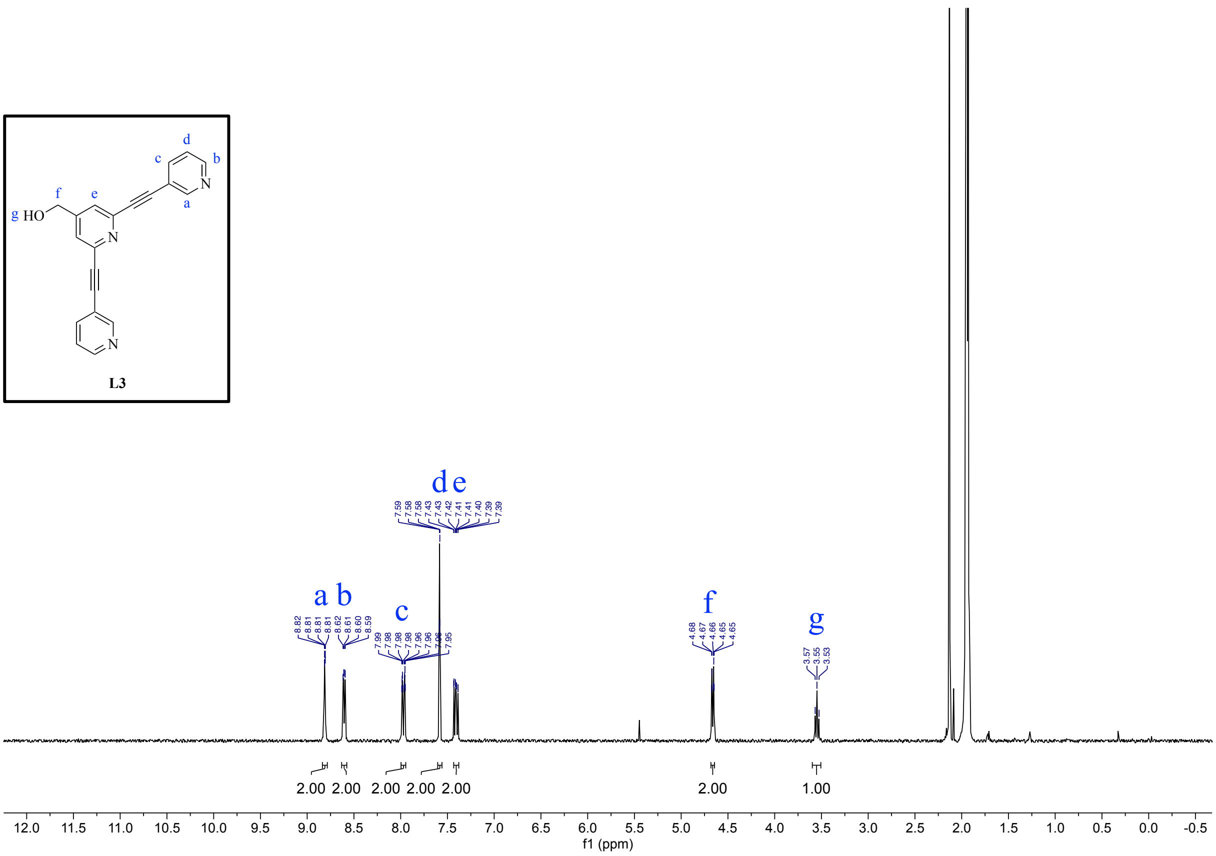

Figure S145: ^{1}H NMR spectrum (300 MHz, CD_3CN, 298 K) of **L3**.

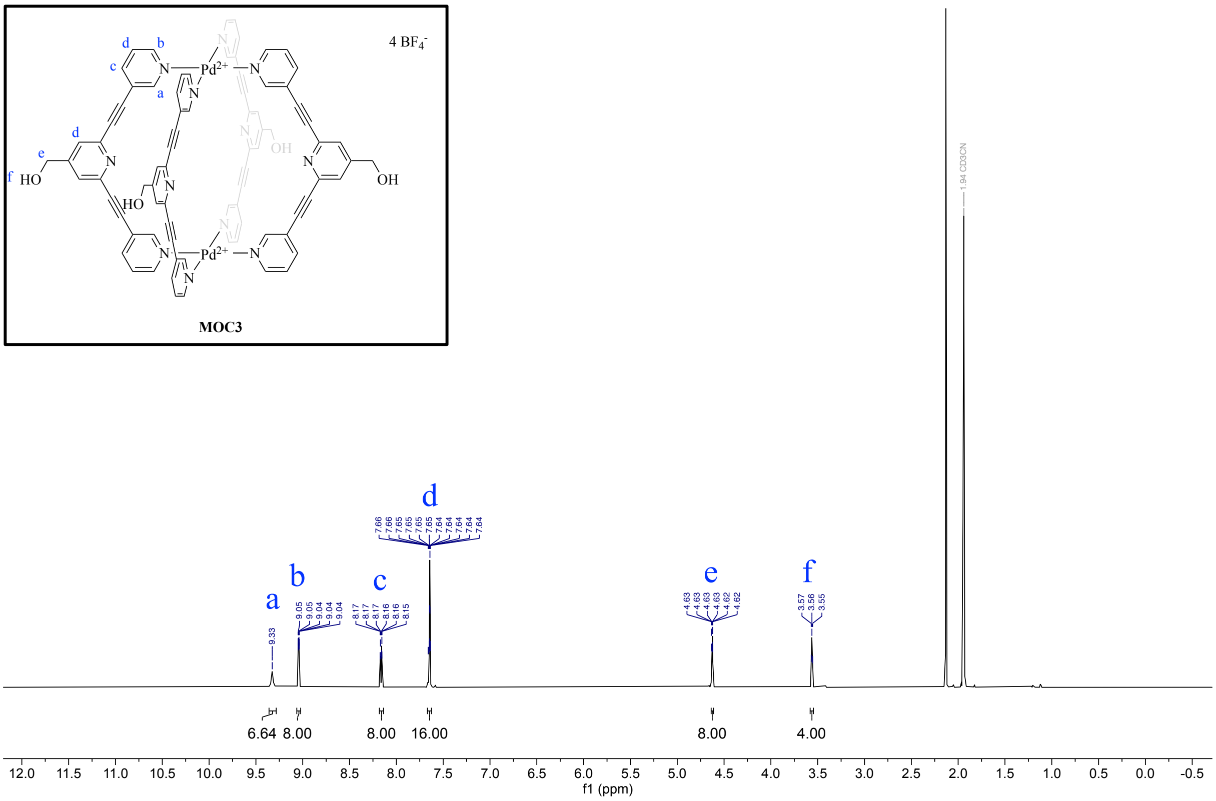

Figure S146: ^{1}H NMR spectrum (600 MHz, CD_3CN, 298 K) of **MOC3**.

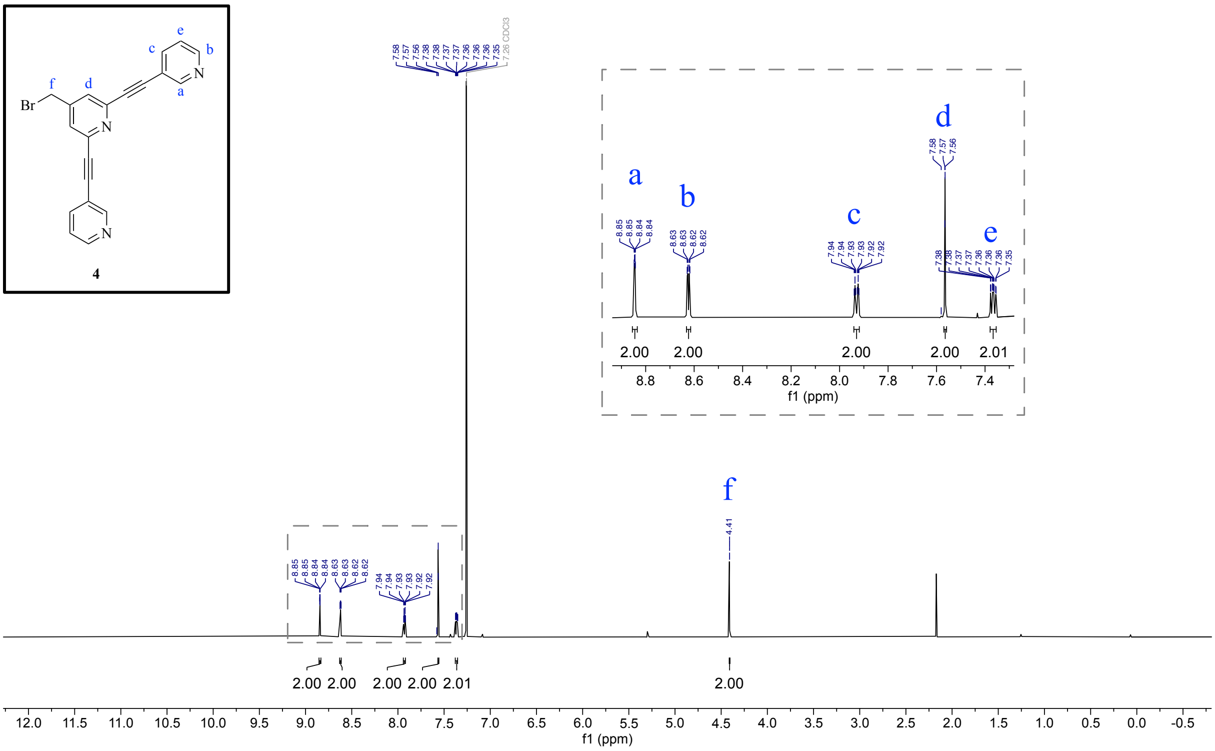

Figure S147: ^{1}H NMR spectrum (600 MHz, $CDCl_3$, 298 K) of **4**.

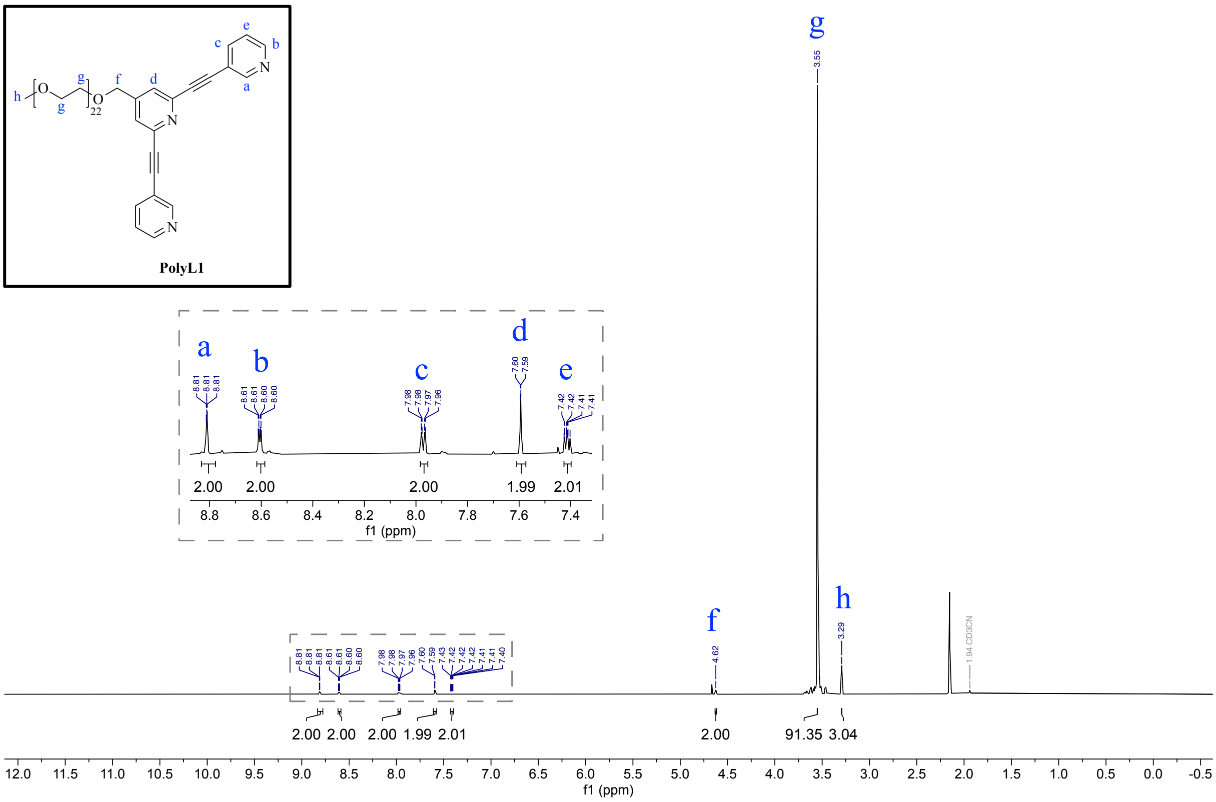

Figure S148: ^{1}H NMR spectrum (600 MHz, CD_3CN, 298 K) of **PolyL1**.

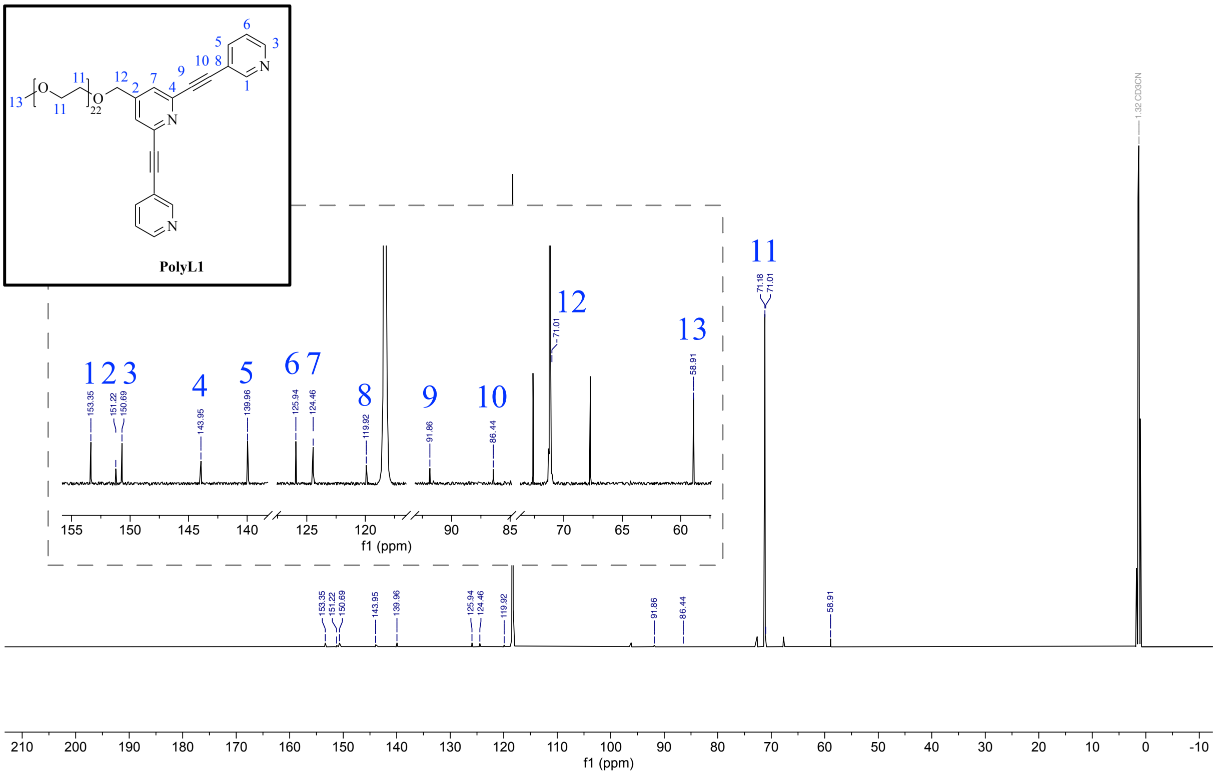

Figure S149: $^{13}C\{^{1}H\}$ NMR spectrum (151 MHz, CD_3CN, 298 K) of **PolyL1**.

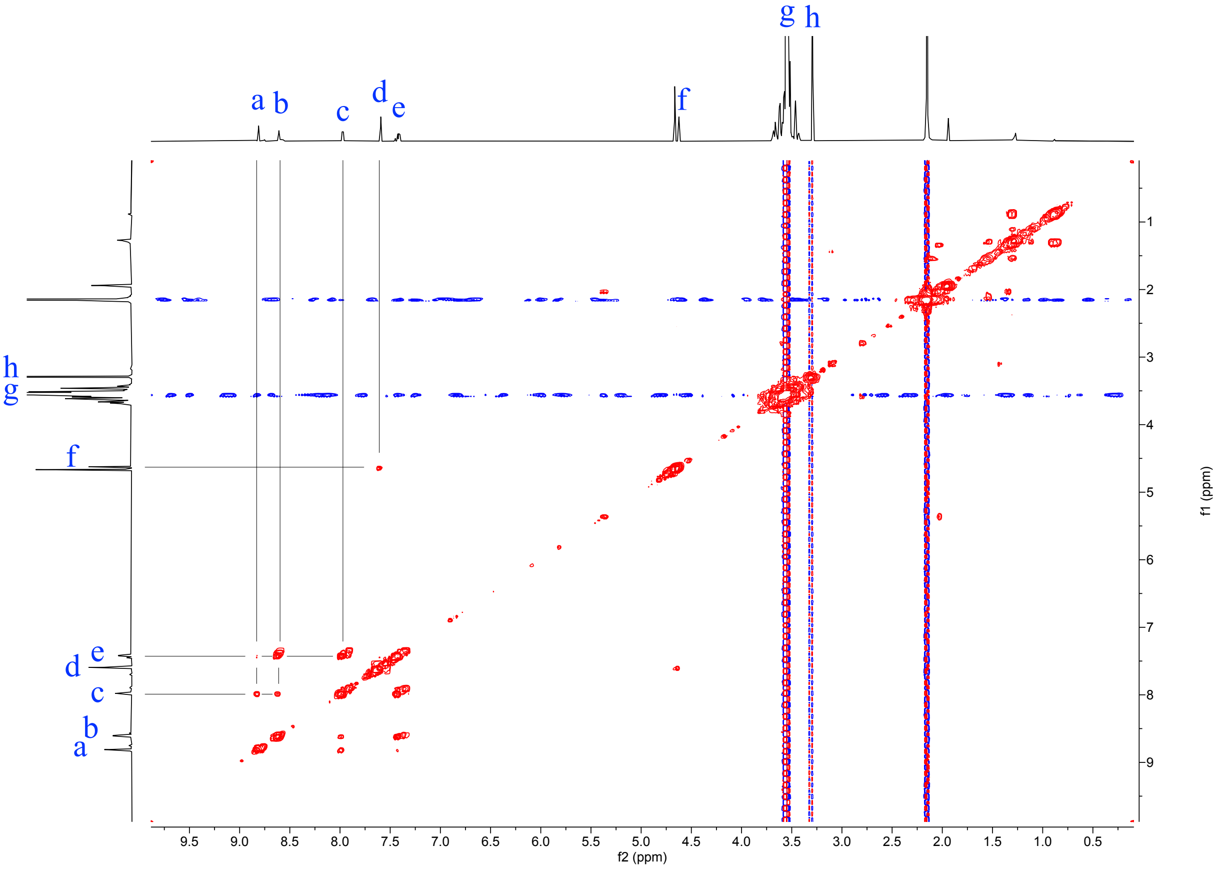

Figure S150: ^{1}H-^{1}H COSY spectrum (600 MHz, CD_3CN, 298 K) of **PolyL1**.

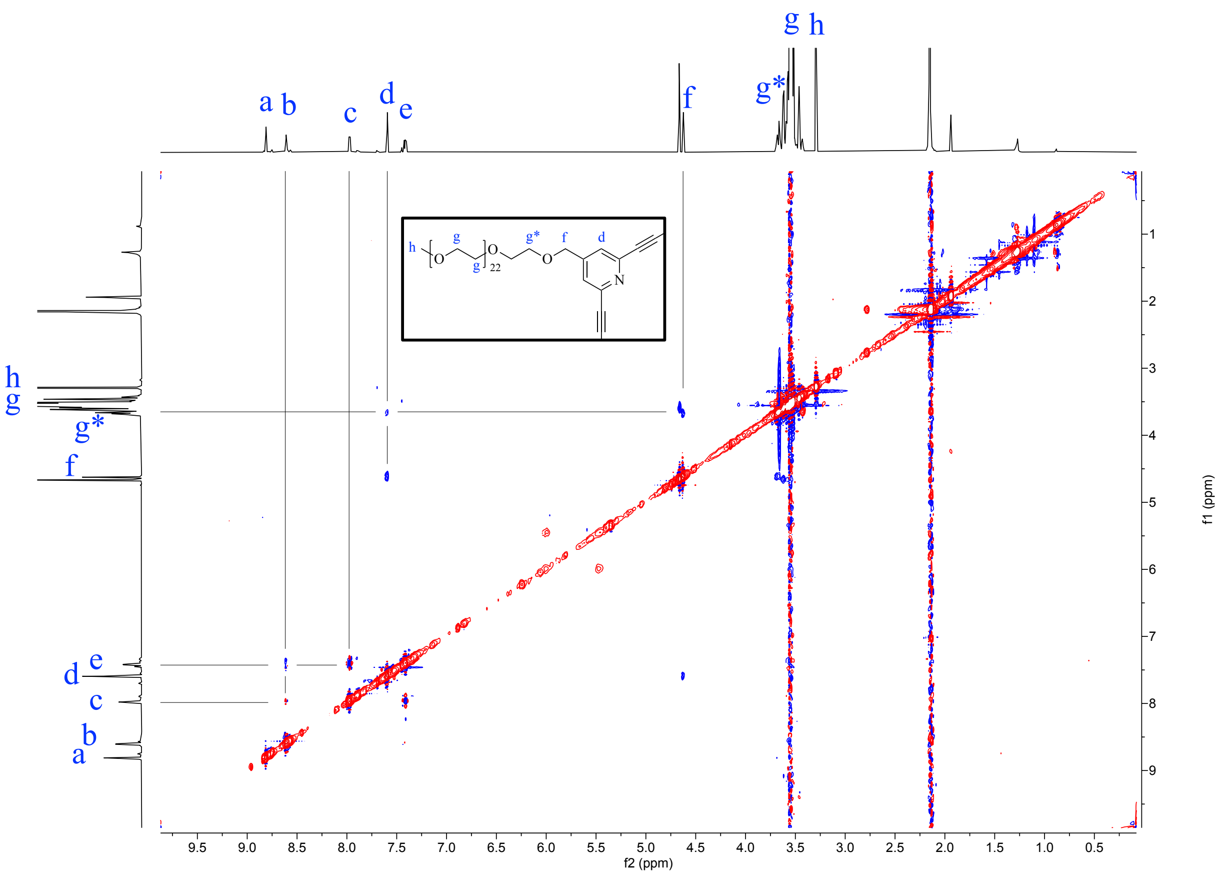

Figure S151: ^{1}H-^{1}H NOESY spectrum (600 MHz, CD_3CN, 298 K) of **PolyL1**.

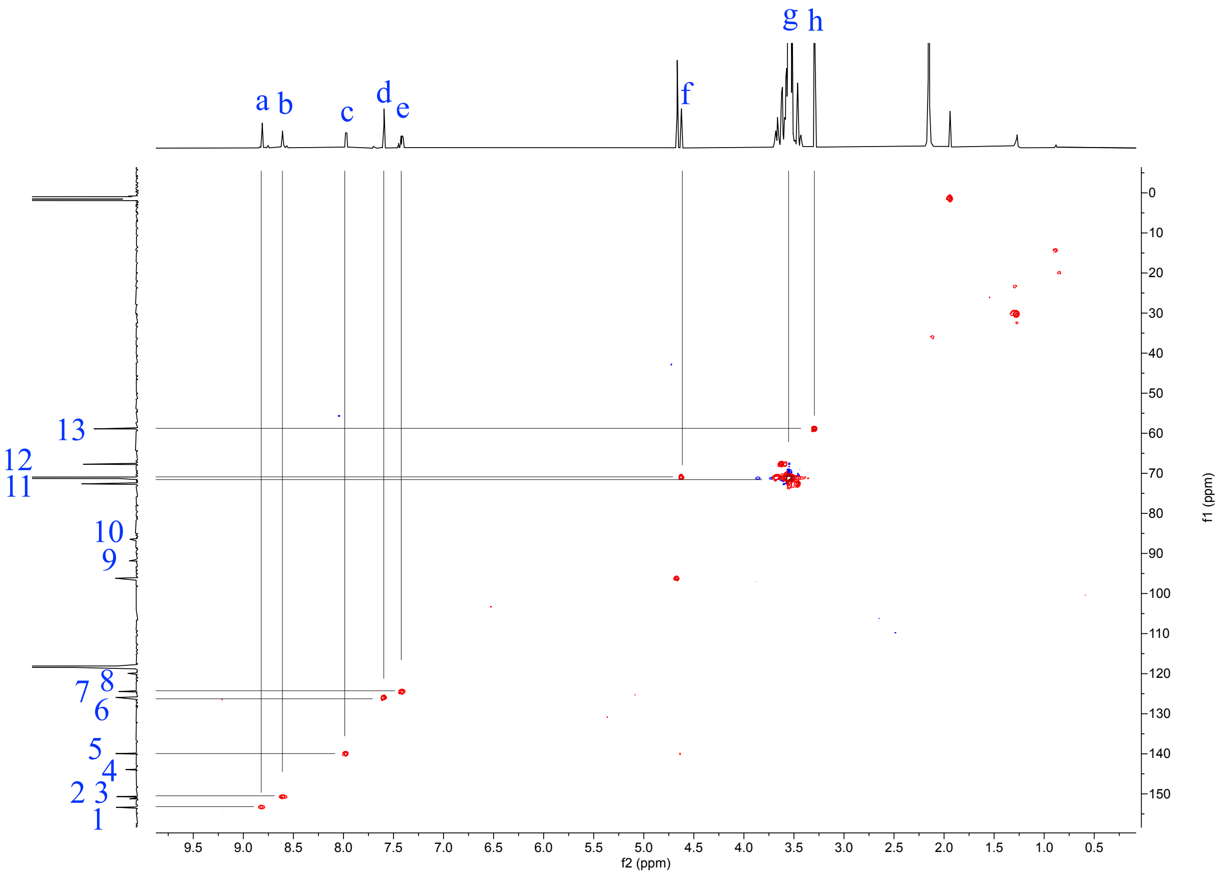

Figure S152: ^{1}H-^{13}C HSQC spectrum (600 MHz, CD_3CN, 298 K) of **PolyL1**.

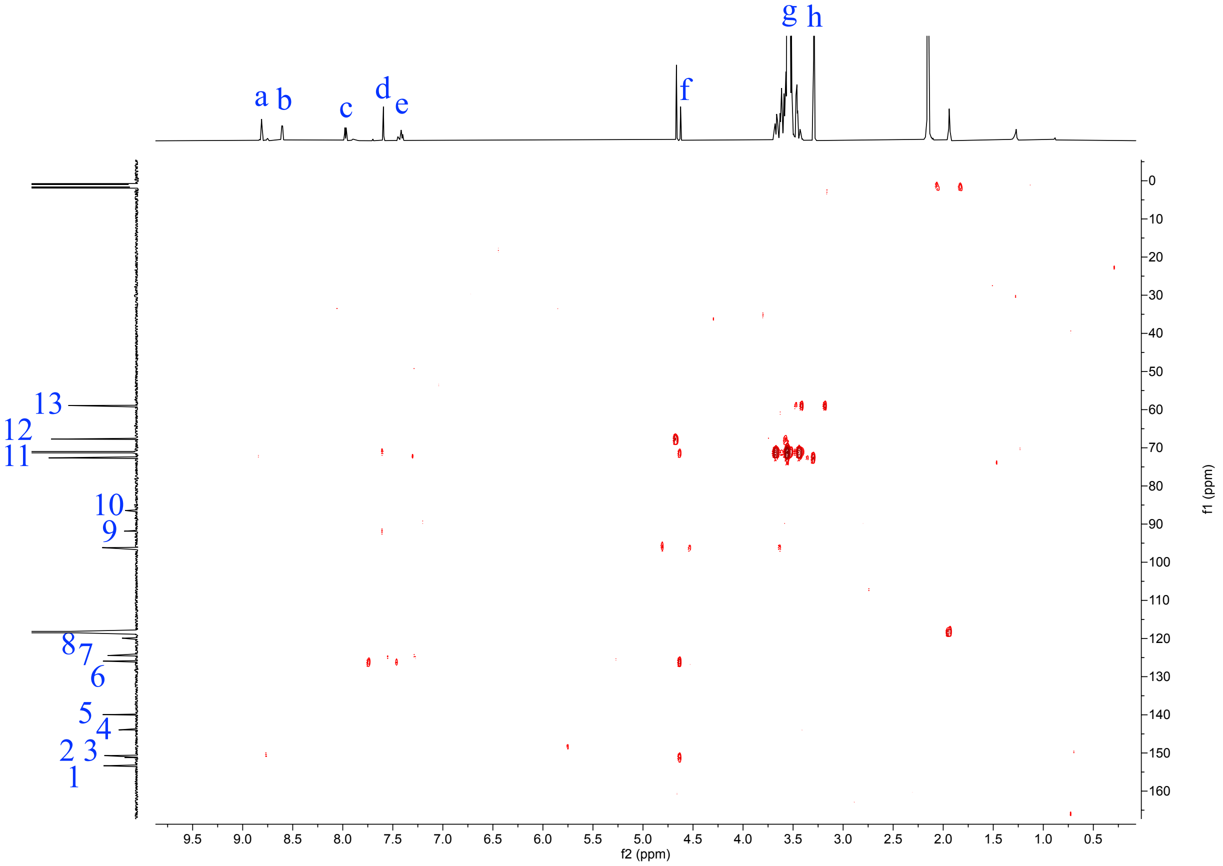

Figure S153: ^{1}H-^{13}C HMBC spectrum (600 MHz, CD_3CN, 298 K) of **PolyL1**.

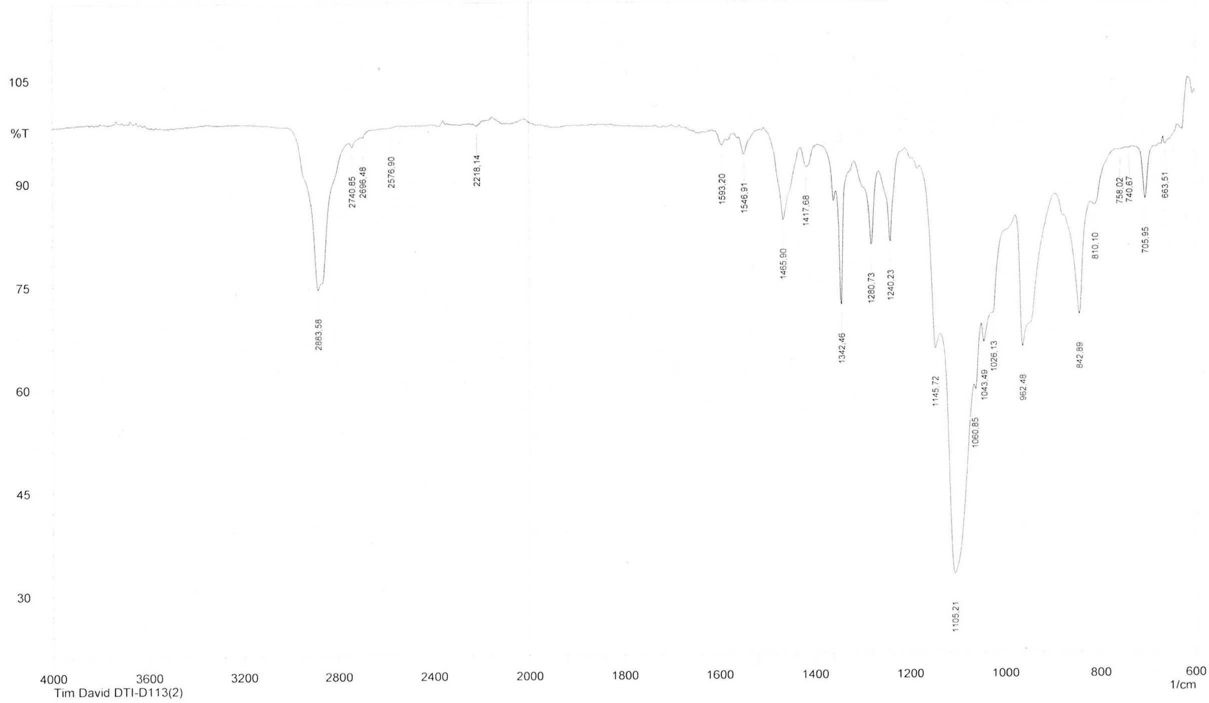

Figure S154: IR spectrum of **PolyL1**.

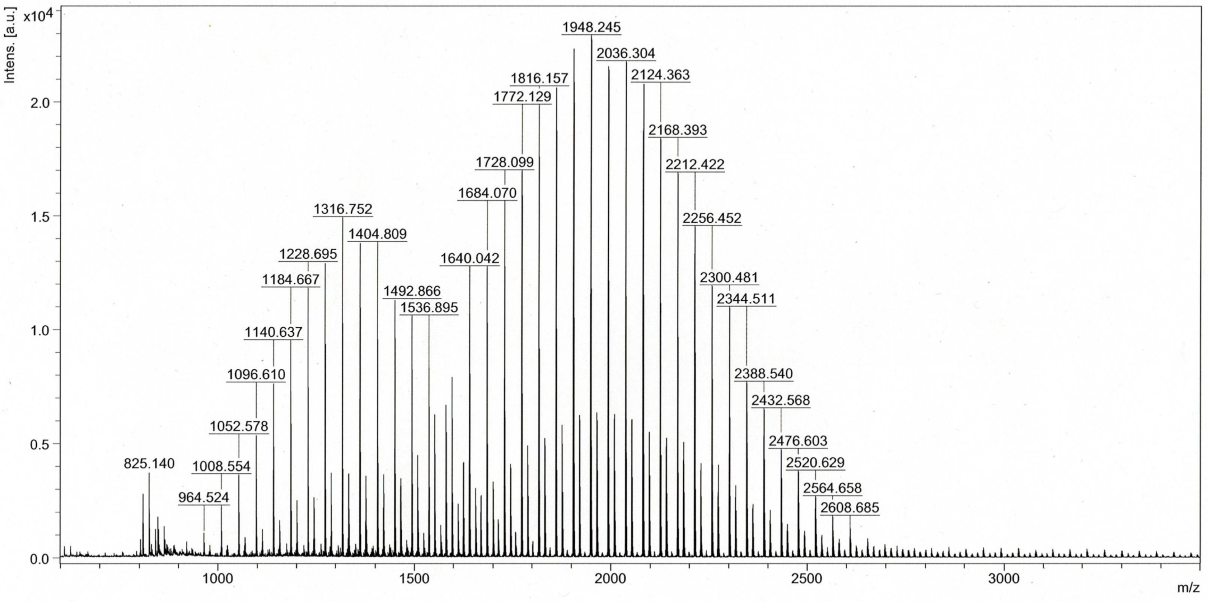

Figure S155: MALDI-MS spectrum of **PolyL1**.

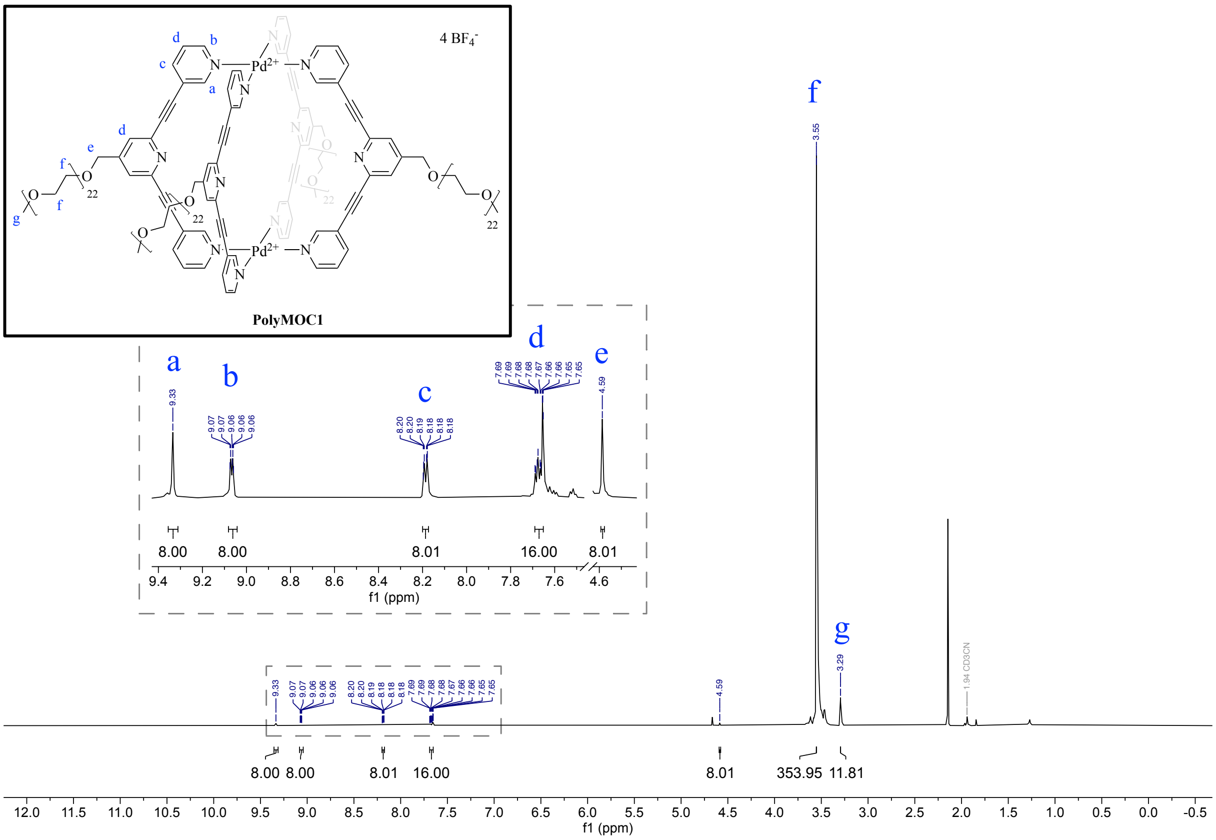

Figure S156: ^{1}H NMR spectrum (600 MHz, CD_3CN, 298 K) of **PolyMOC1**.

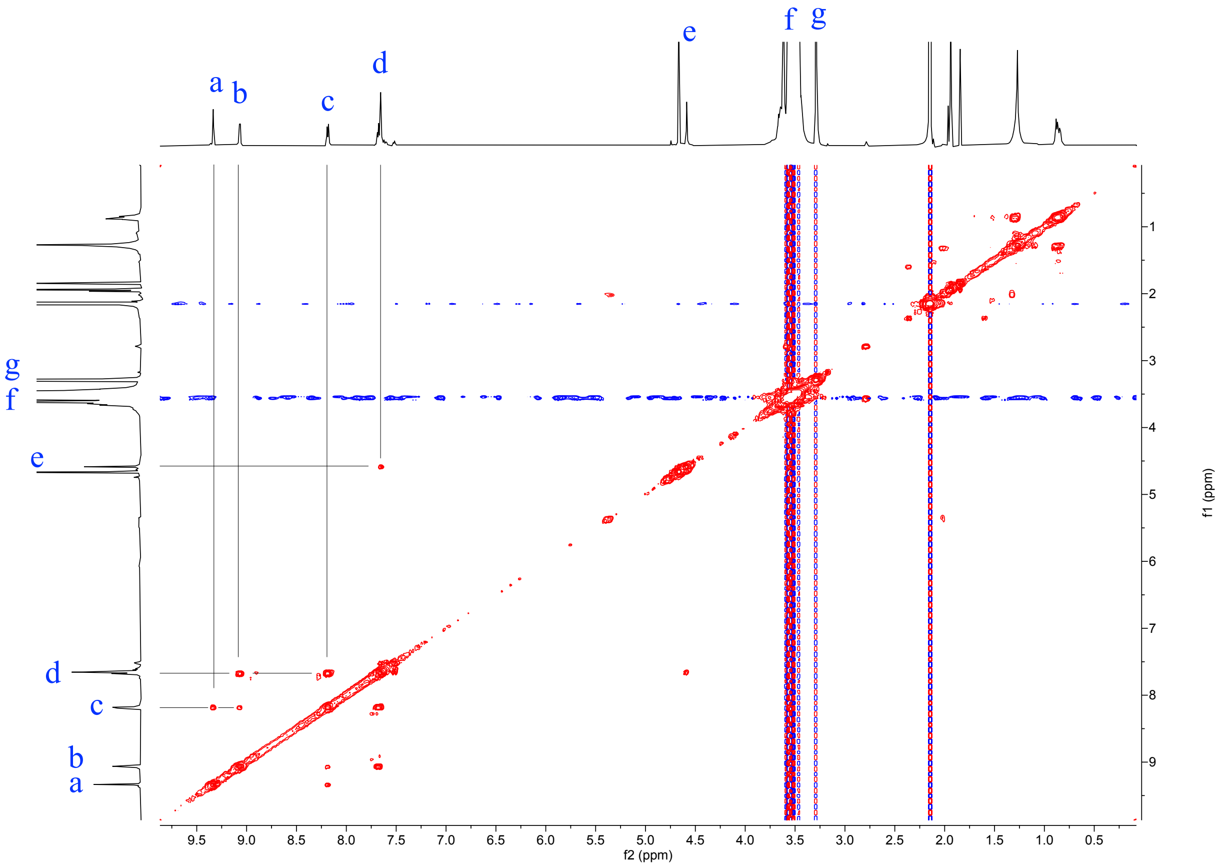

Figure S157: ^{1}H-^{1}H COSY spectrum (600 MHz, CD_3CN, 298 K) of **PolyMOC1**.

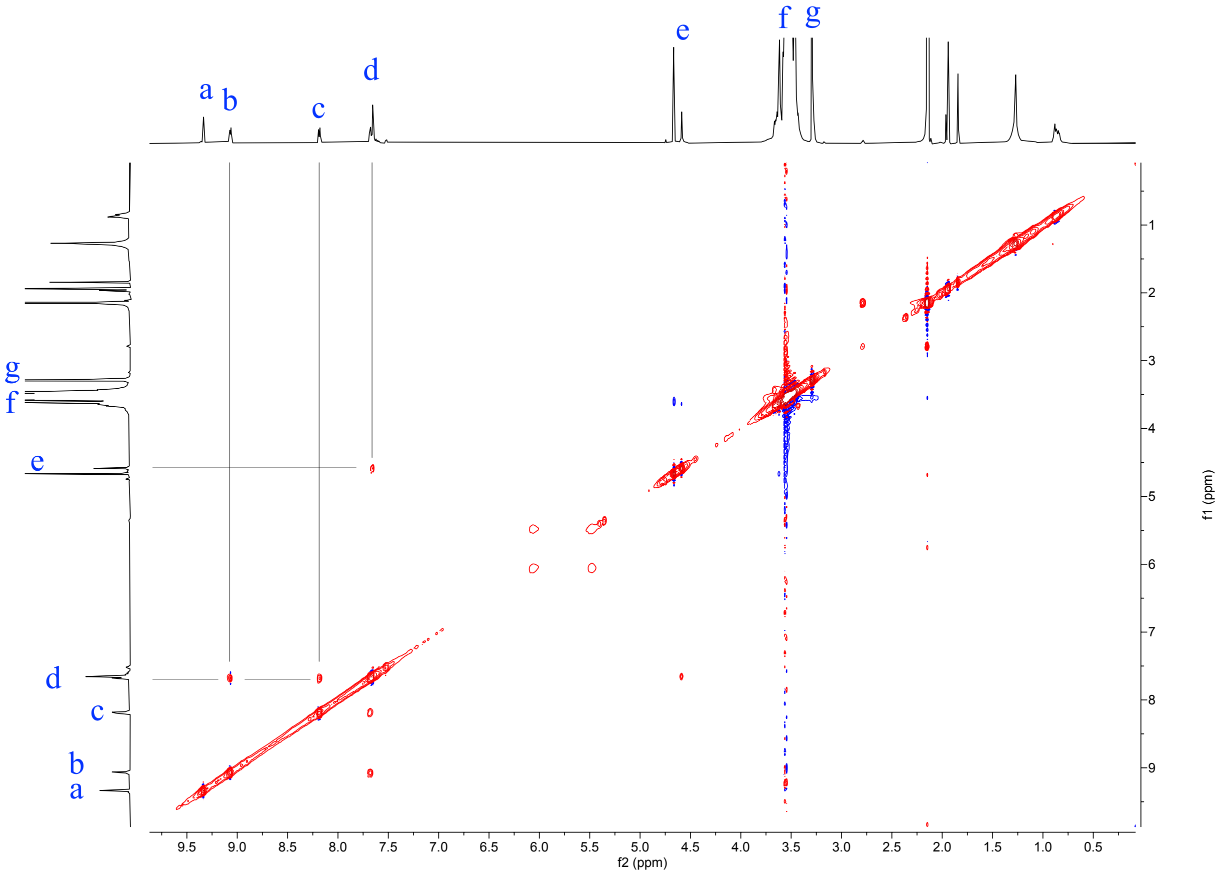

Figure S158: ^{1}H-^{1}H NOESY spectrum (600 MHz, CD_3CN, 298 K) of **PolyMOC1**.

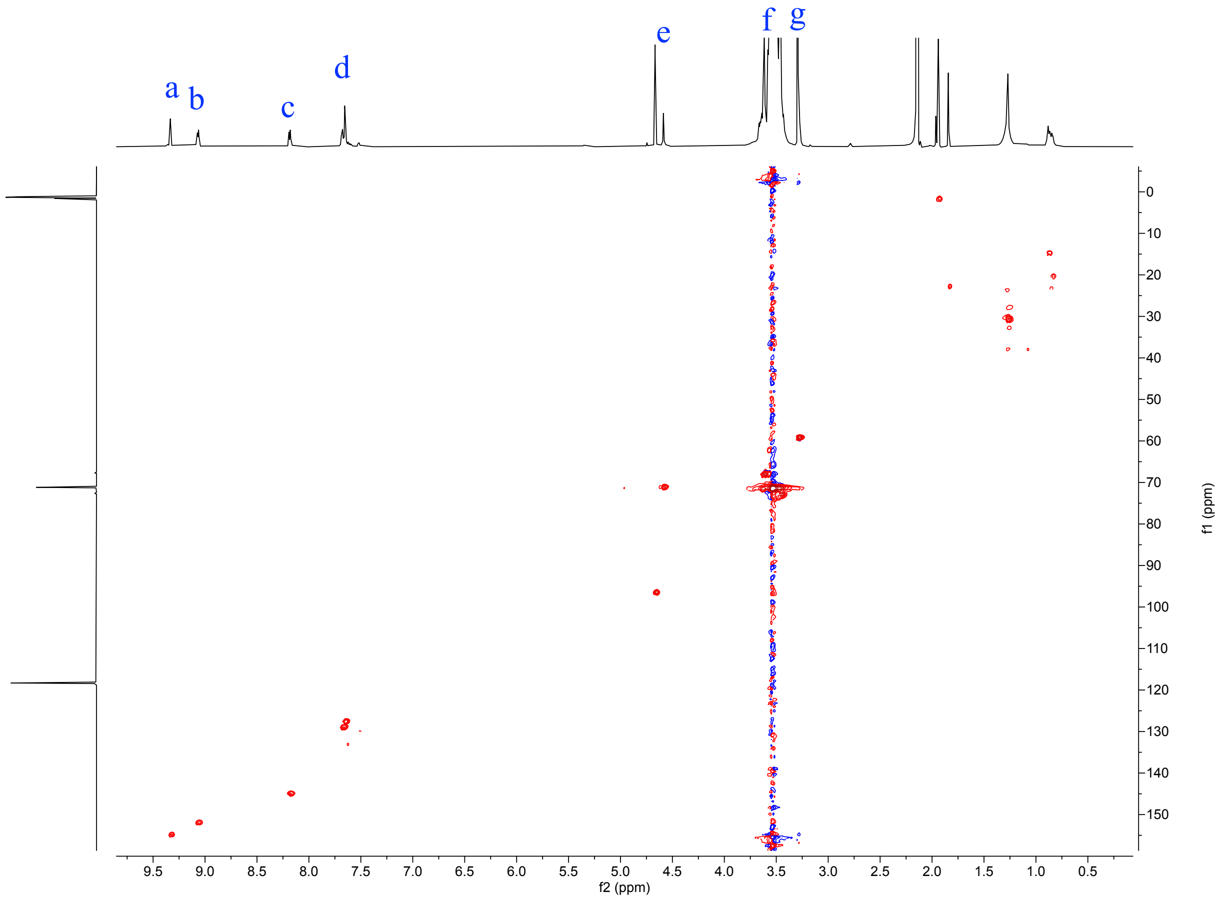

Figure S159: ^{1}H-^{13}C HSQC spectrum (600 MHz, CD_3CN, 298 K) of **PolyMOC1**.

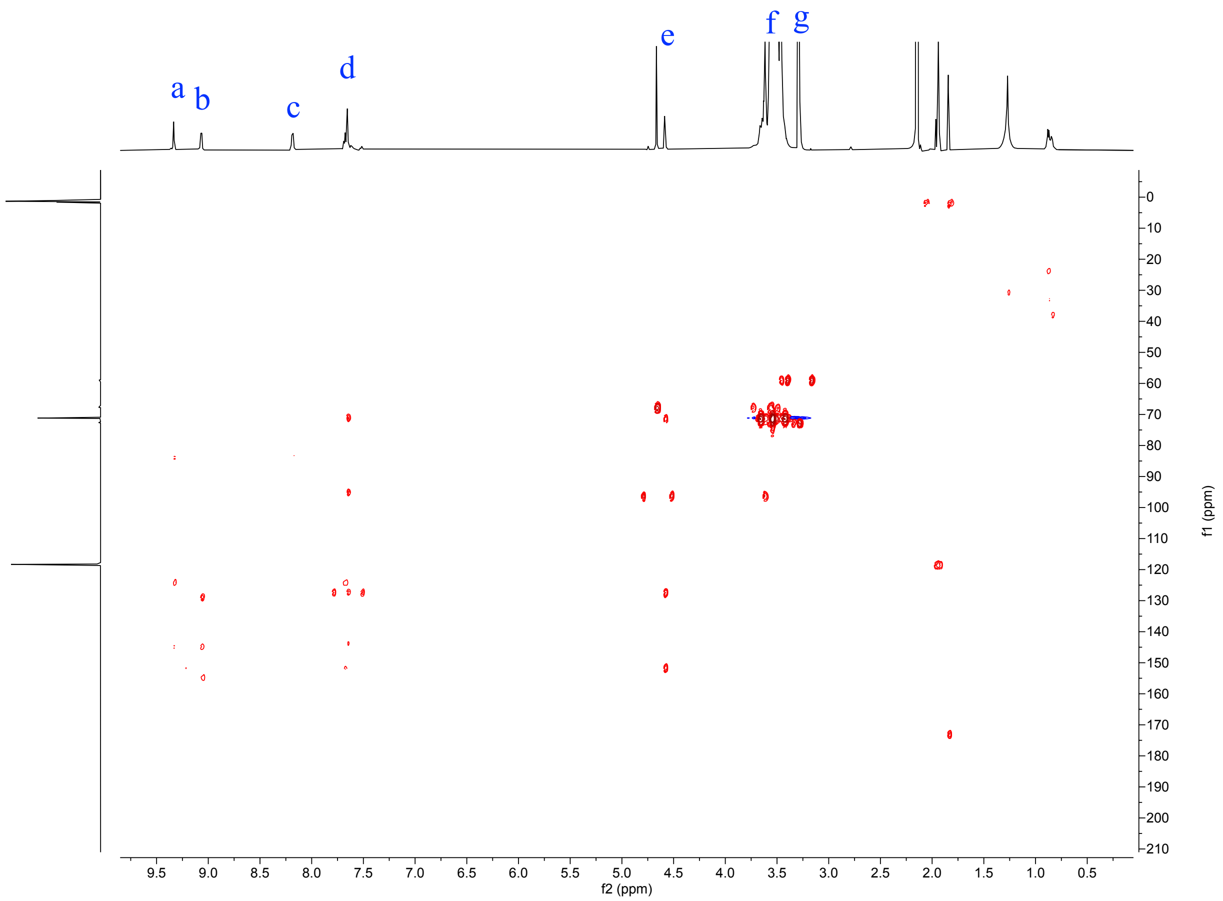

Figure S160: ^{1}H-^{13}C HMBC spectrum (600 MHz, CD_3CN, 298 K) of **PolyMOC1**.

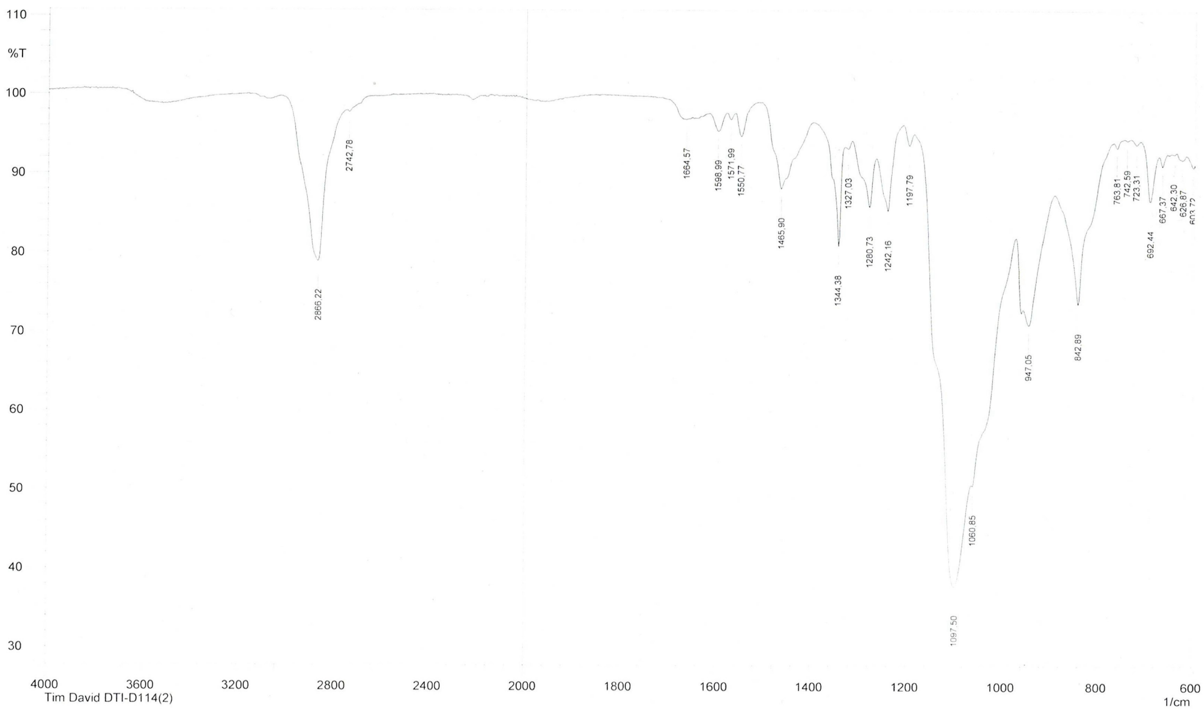

Figure S161: IR spectrum of **PolyMOC1**.

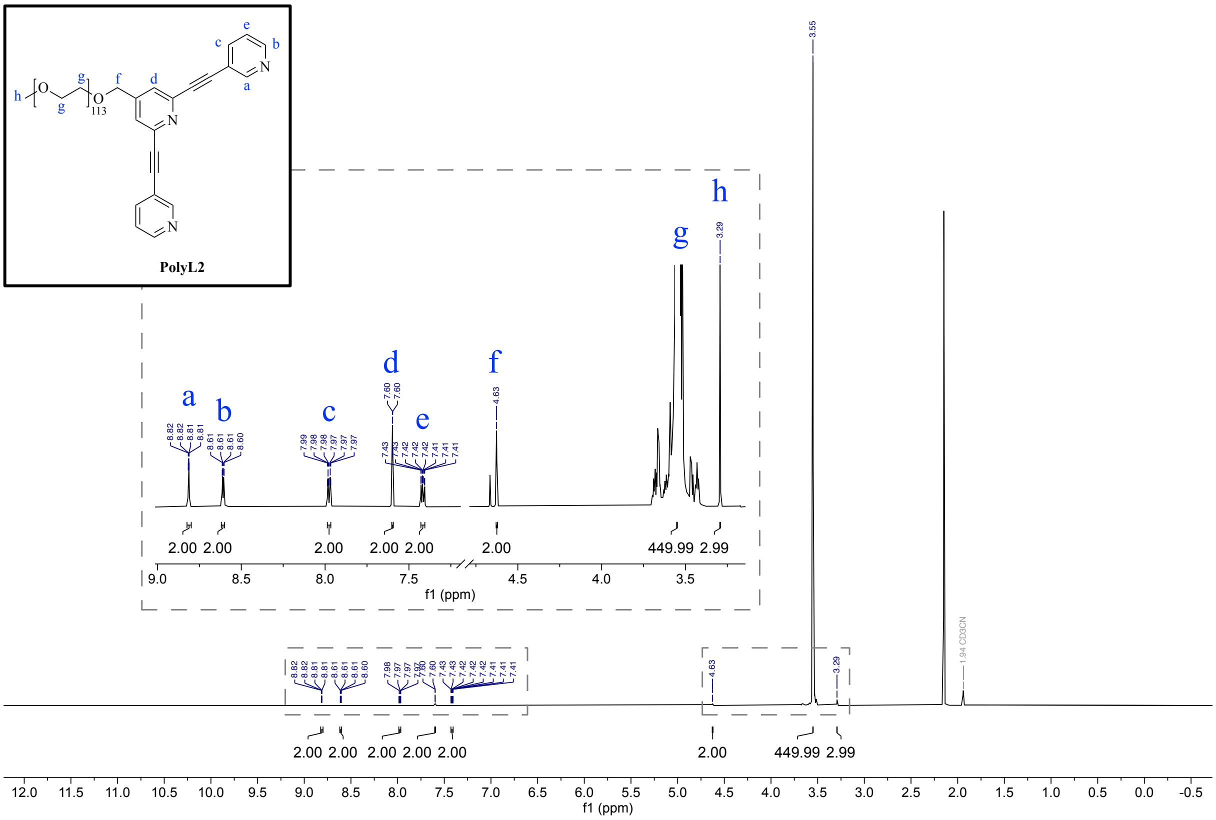

Figure S162: ^{1}H NMR spectrum (600 MHz, CD_3CN, 298 K) of **PolyL2**.

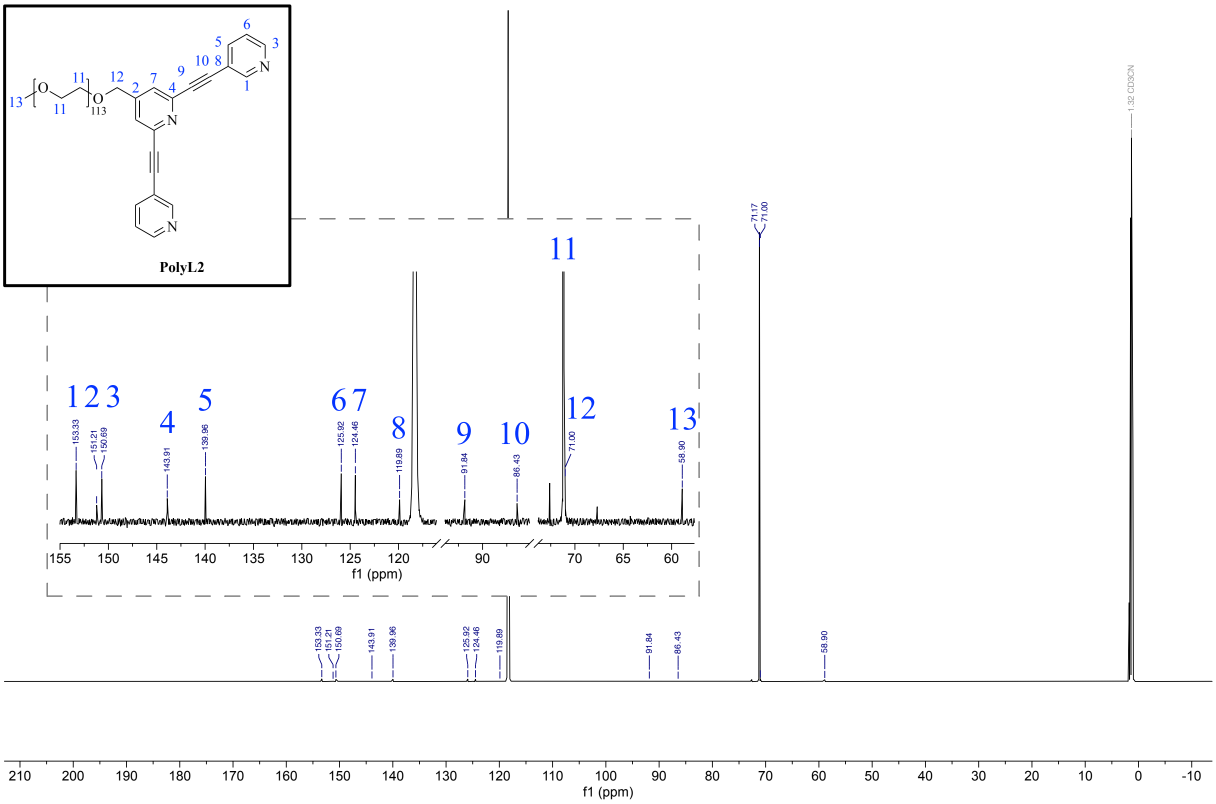

Figure S163: $^{13}C\{^{1}H\}$ NMR spectrum (151 MHz, CD_3CN, 298 K) of **PolyL2**.

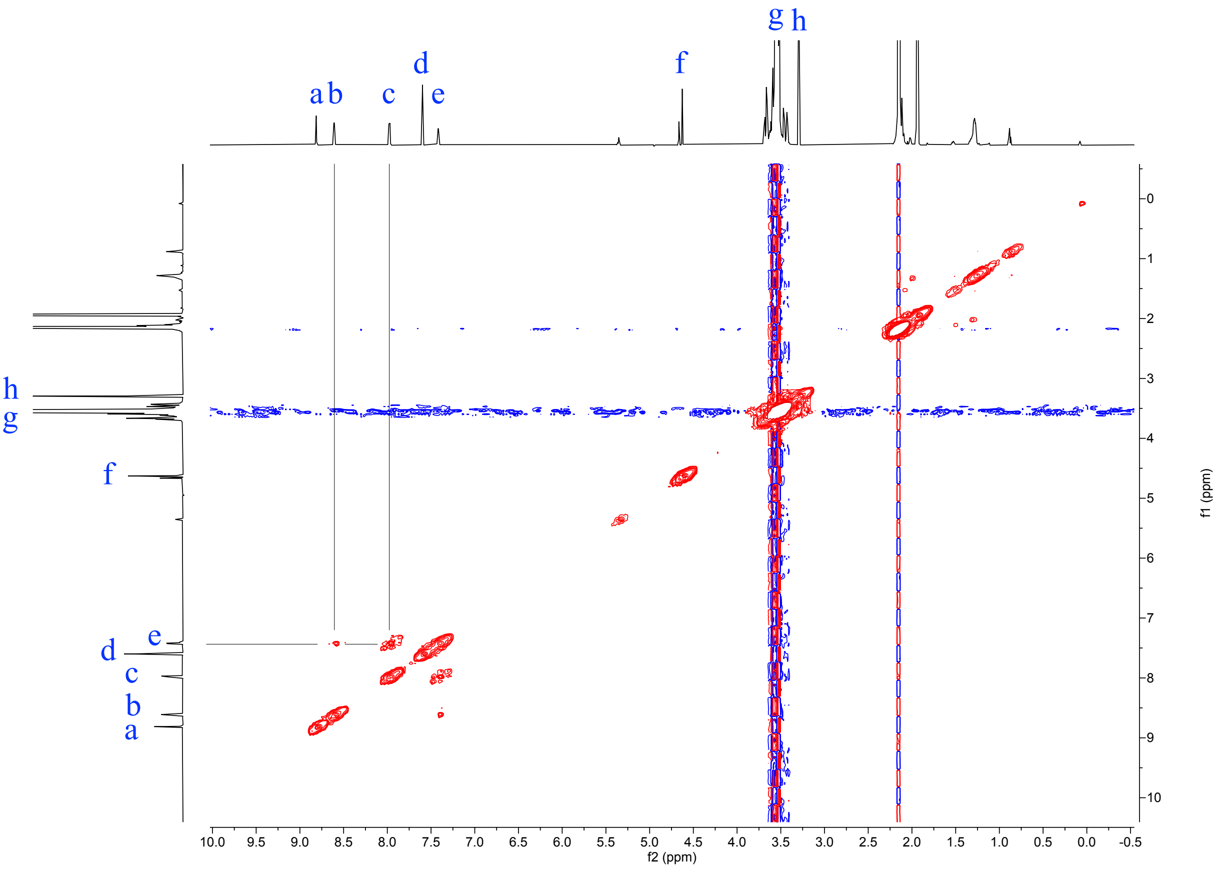

Figure S164: ^{1}H-^{1}H COSY spectrum (600 MHz, CD_3CN, 298 K) of **PolyL2**.

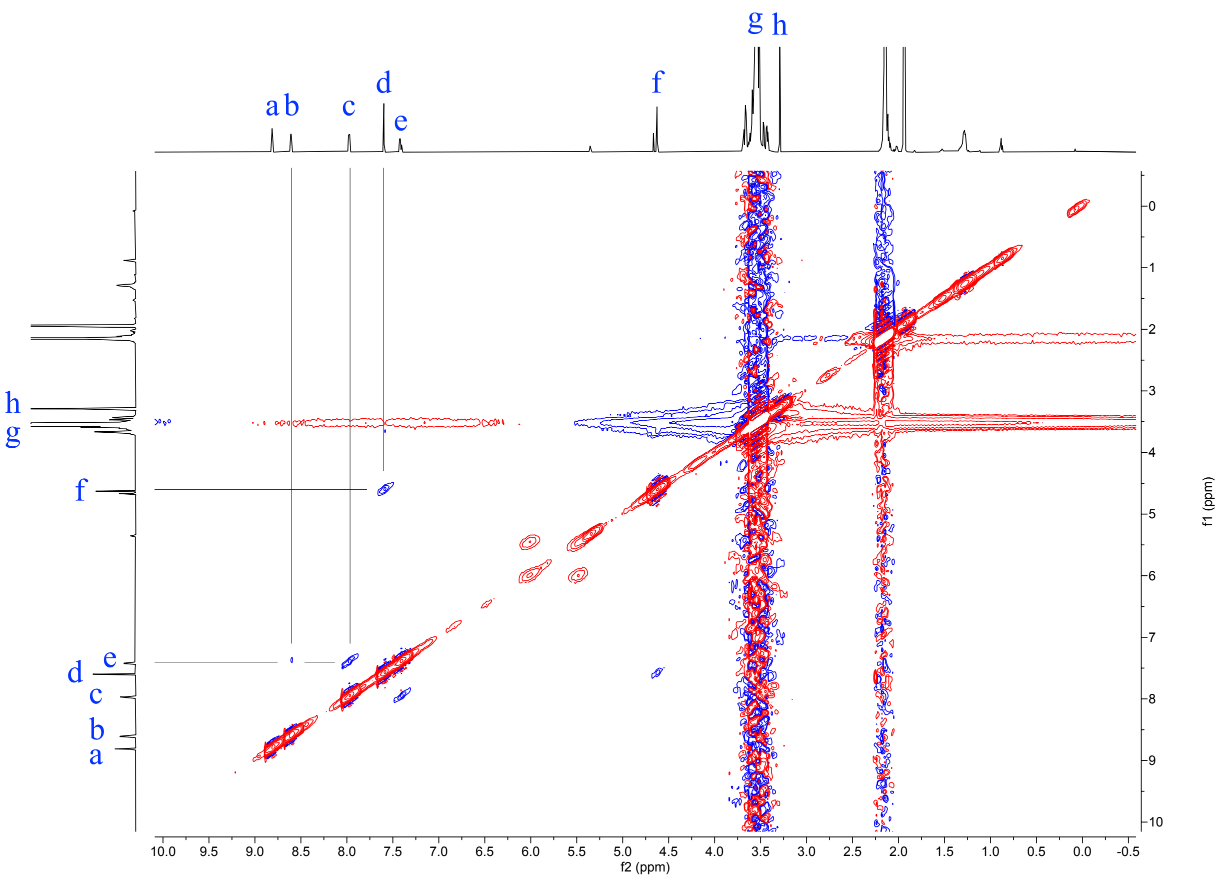

Figure S165: ^{1}H-^{1}H NOESY spectrum (600 MHz, CD_3CN, 298 K) of **PolyL2**.

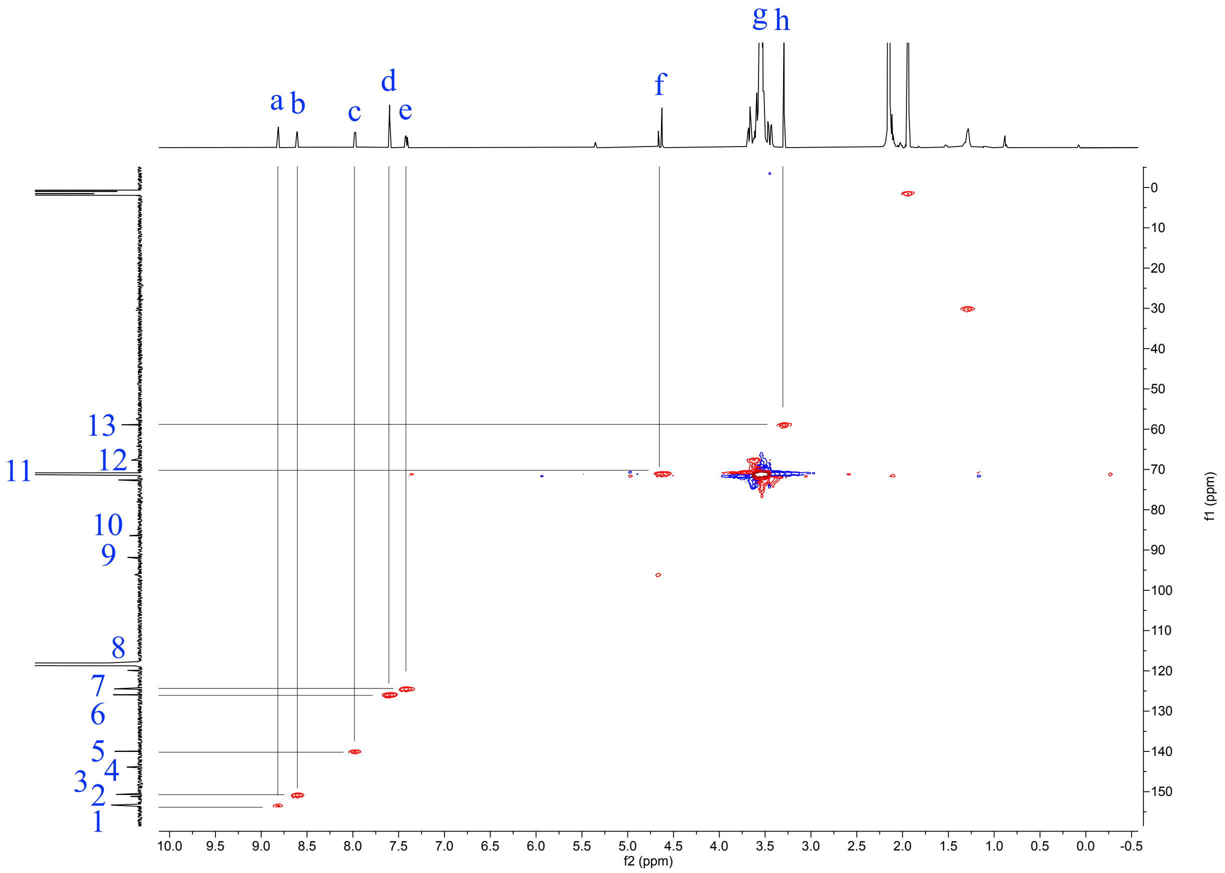

Figure S166: ^{1}H-^{13}C HSQC spectrum (600 MHz, CD_3CN, 298 K) of **PolyL2**.

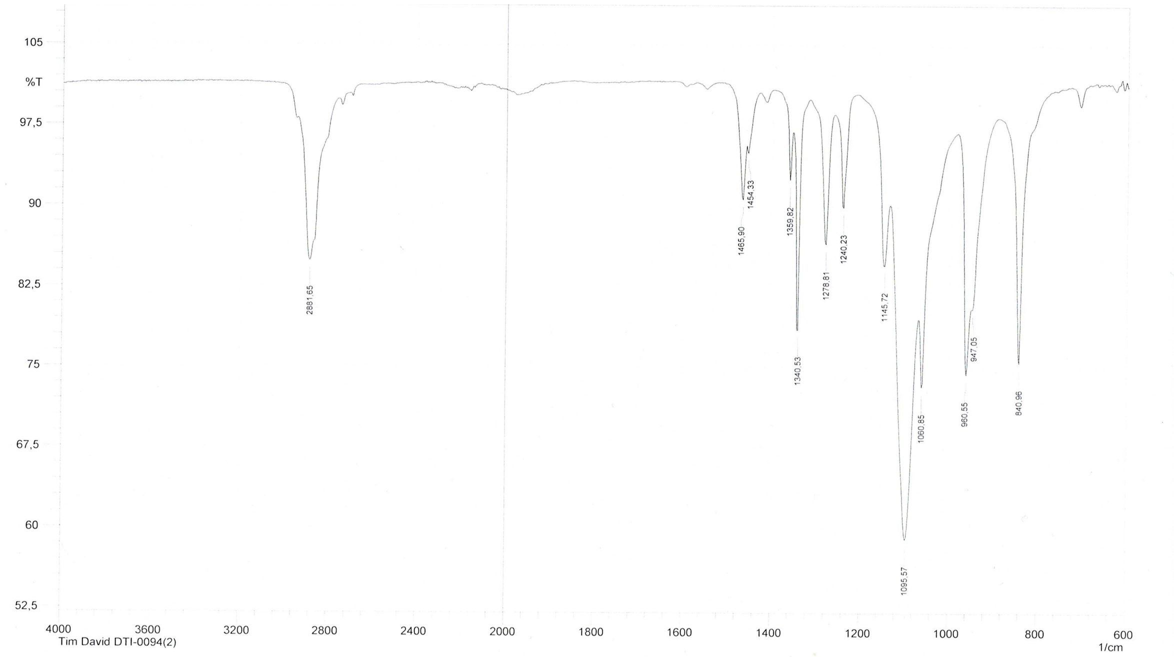

Figure S167: IR spectrum of **PolyL2**.

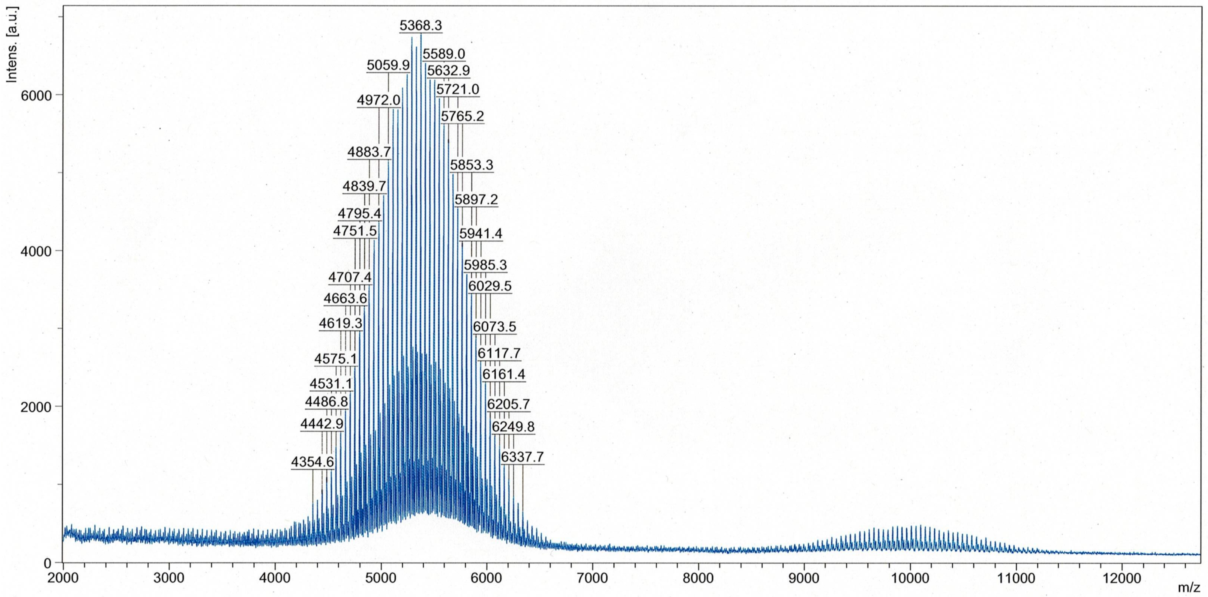

Figure S168: MALDI-MS spectrum of **PolyL2**.

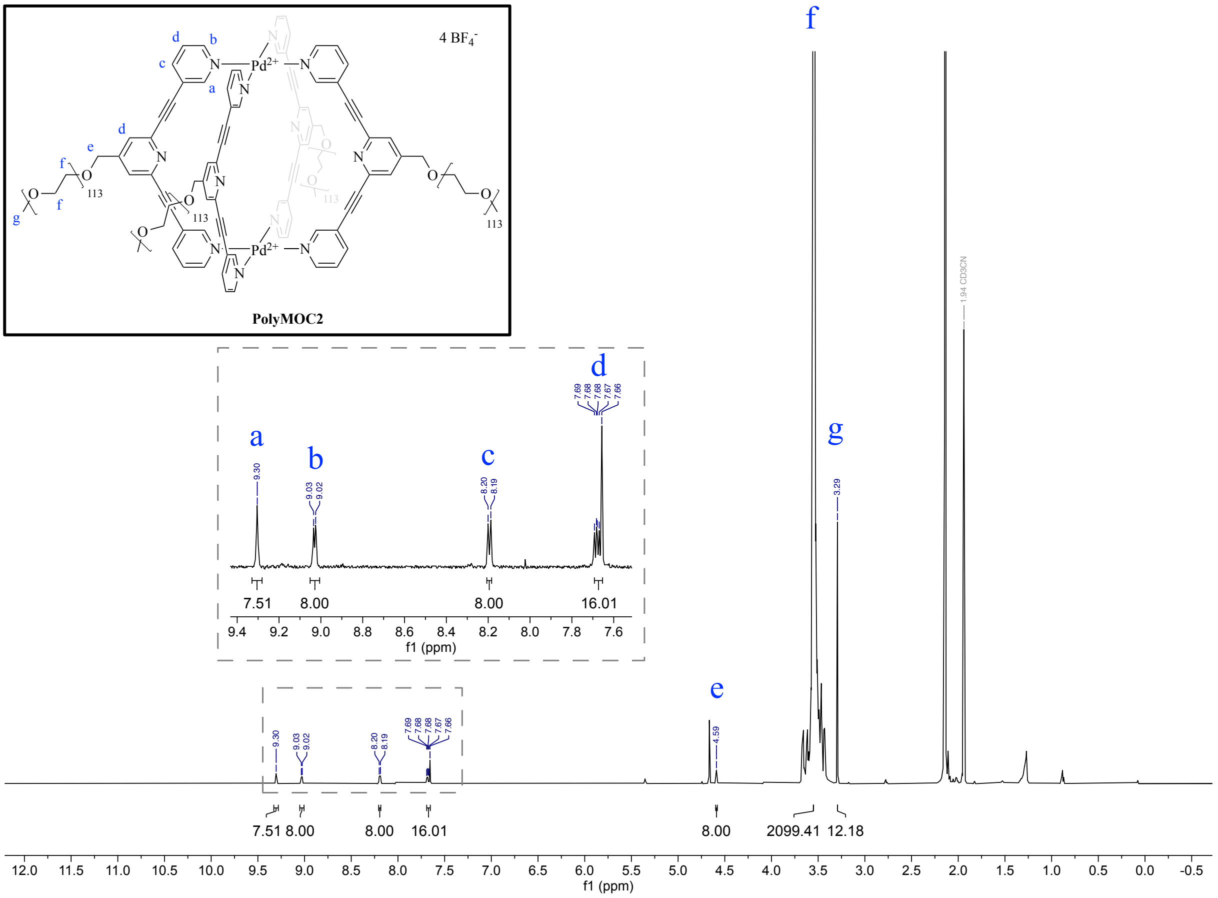

Figure S169: ^{1}H NMR spectrum (600 MHz, CD_3CN, 298 K) of **PolyMOC2**.

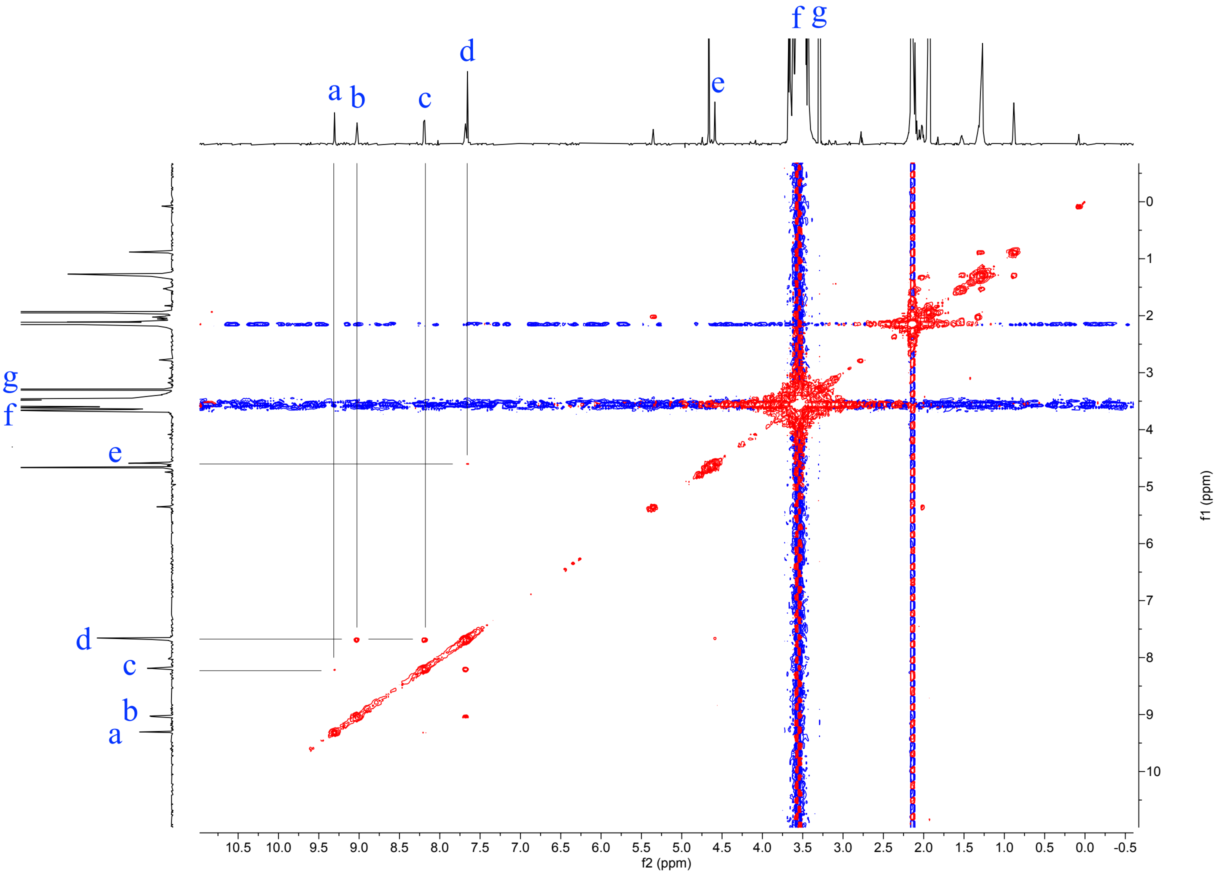

Figure S170: ^{1}H-^{1}H COSY spectrum (600 MHz, CD_3CN, 298 K) of **PolyMOC2**.

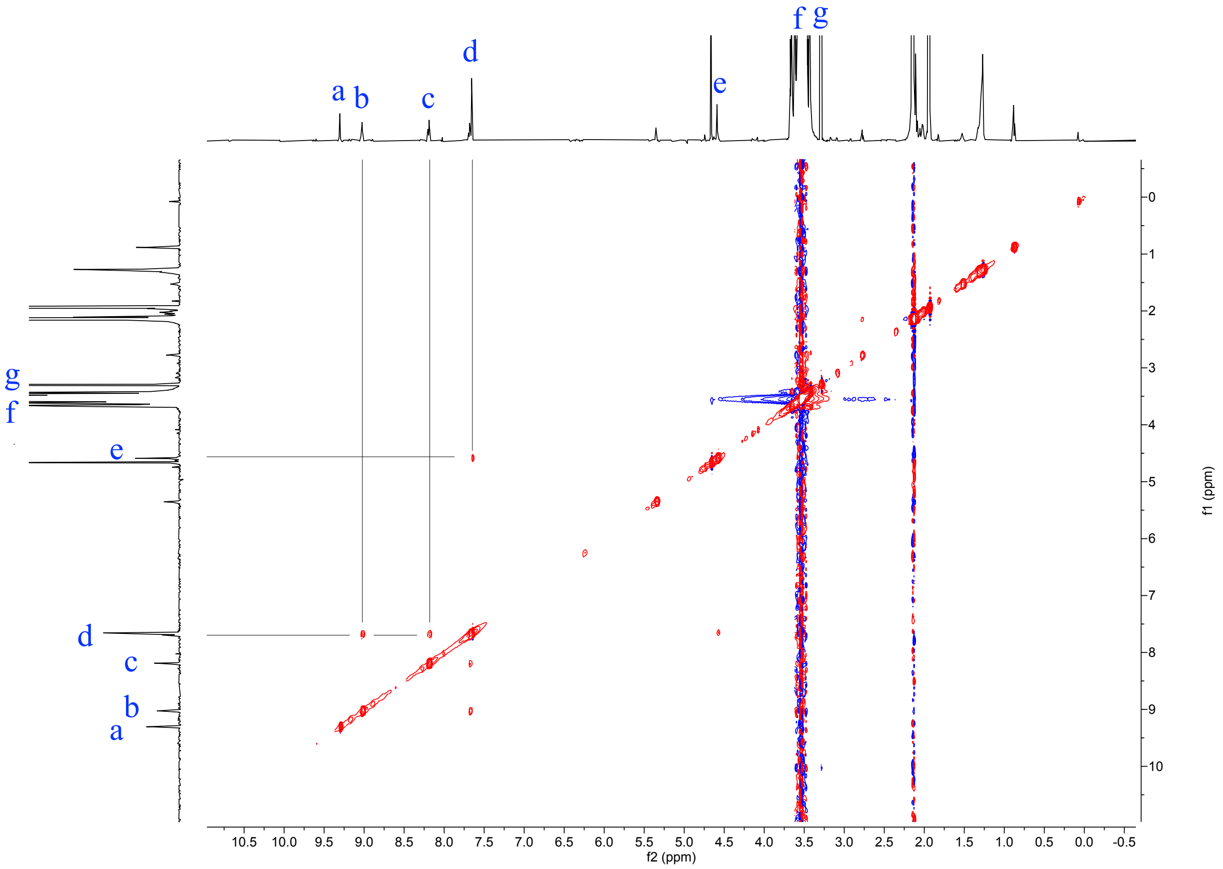

Figure S171: ^{1}H-^{1}H NOESY spectrum (600 MHz, CD_3CN, 298 K) of **PolyMOC2**.

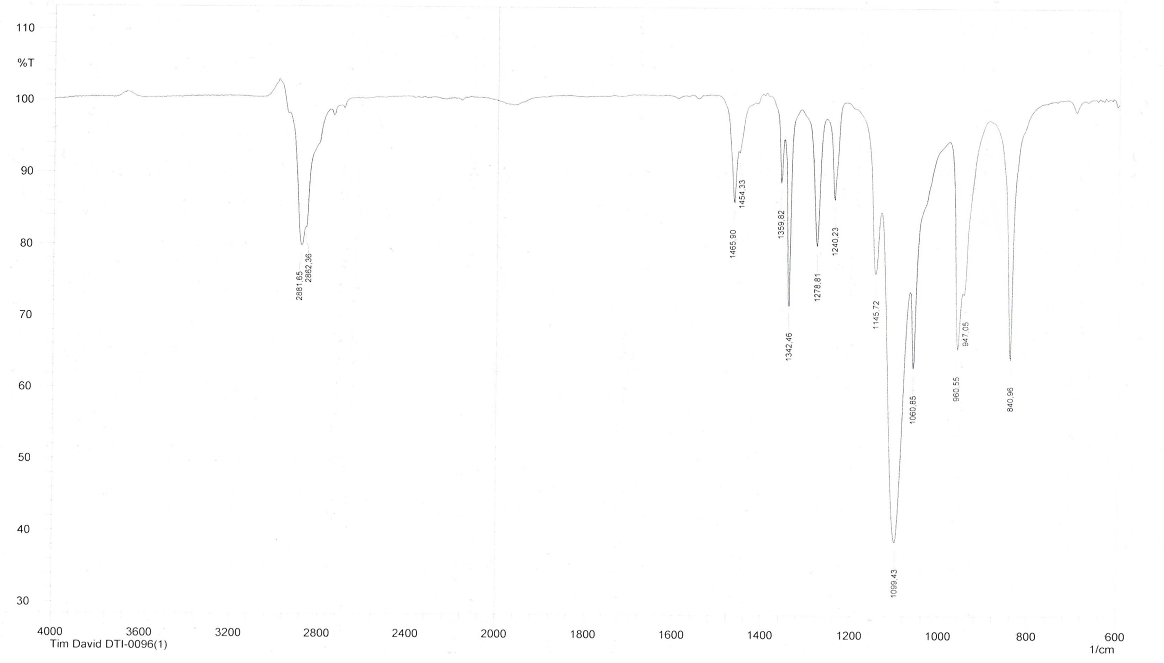

Figure S172: IR spectrum of **PolyMOC2**.

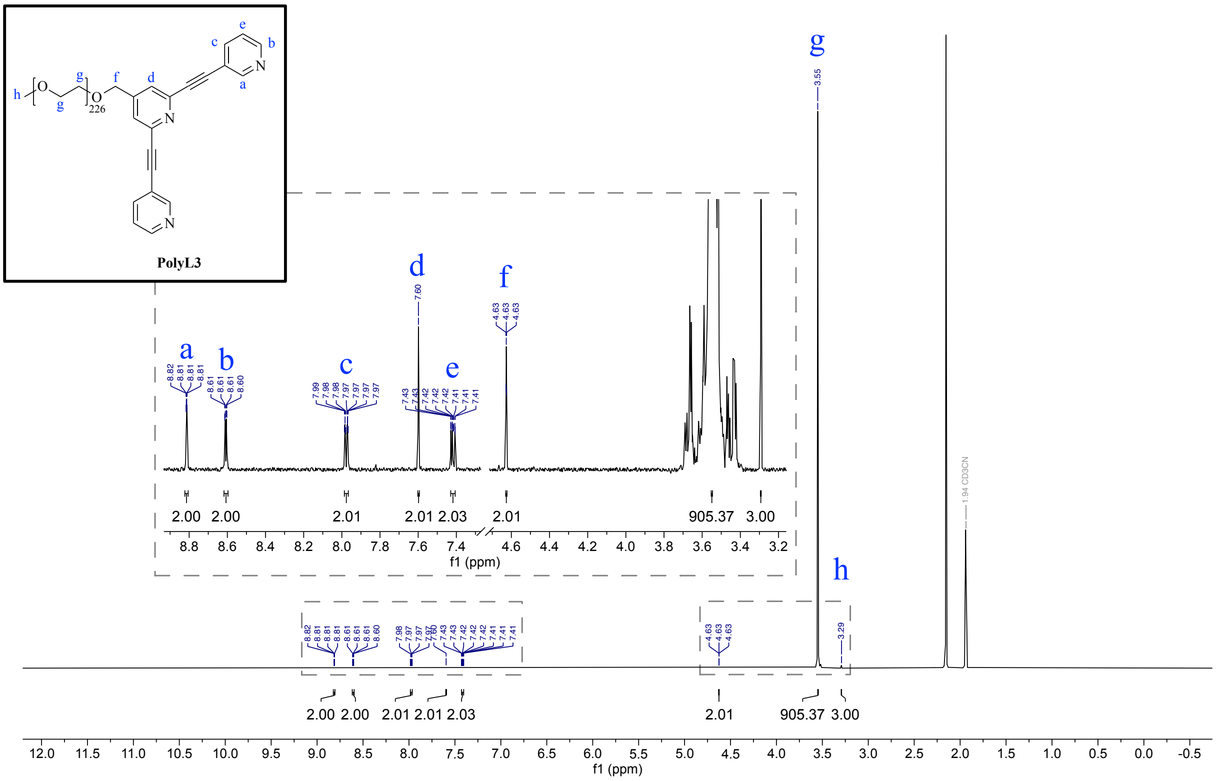

Figure S173: ^{1}H NMR spectrum (600 MHz, CD_3CN, 298 K) of **PolyL3**.

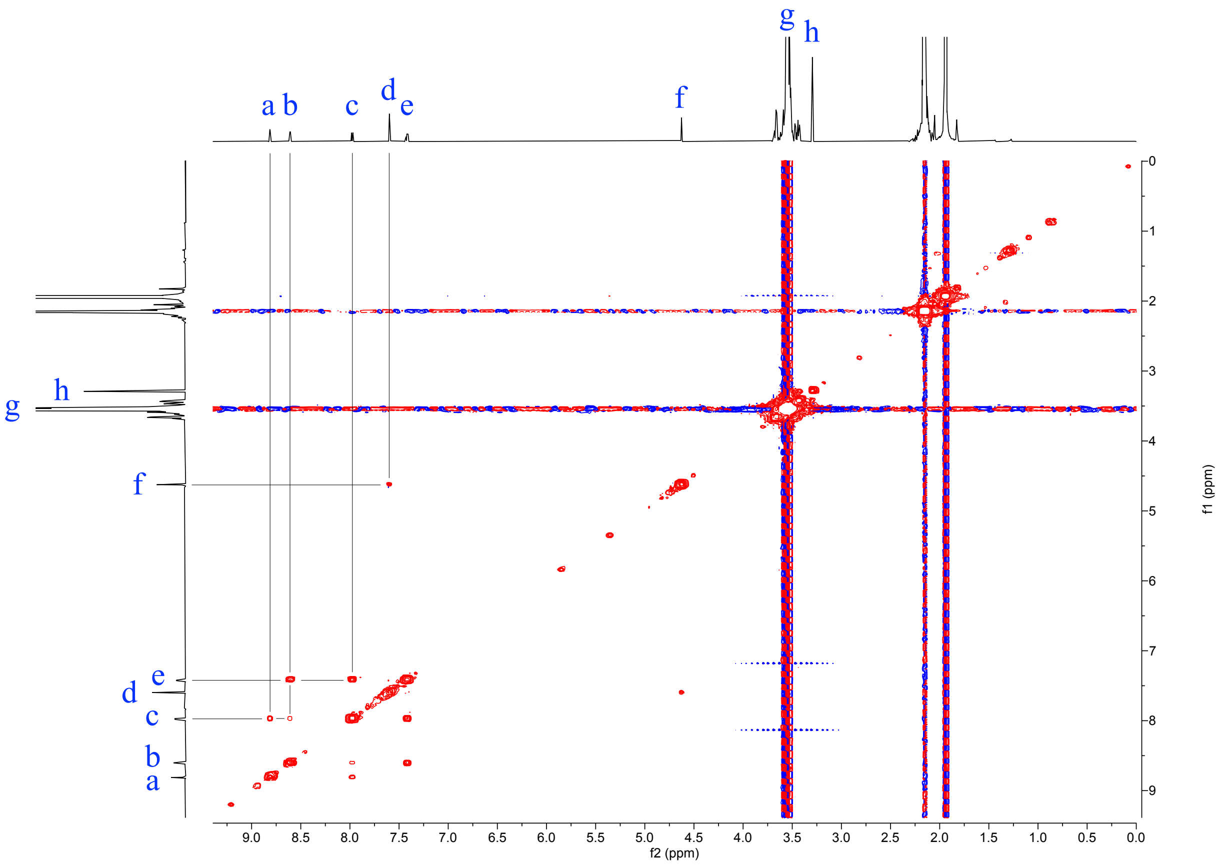

Figure S174: ^{1}H-^{1}H COSY spectrum (600 MHz, CD_3CN, 298 K) of **PolyL3**.

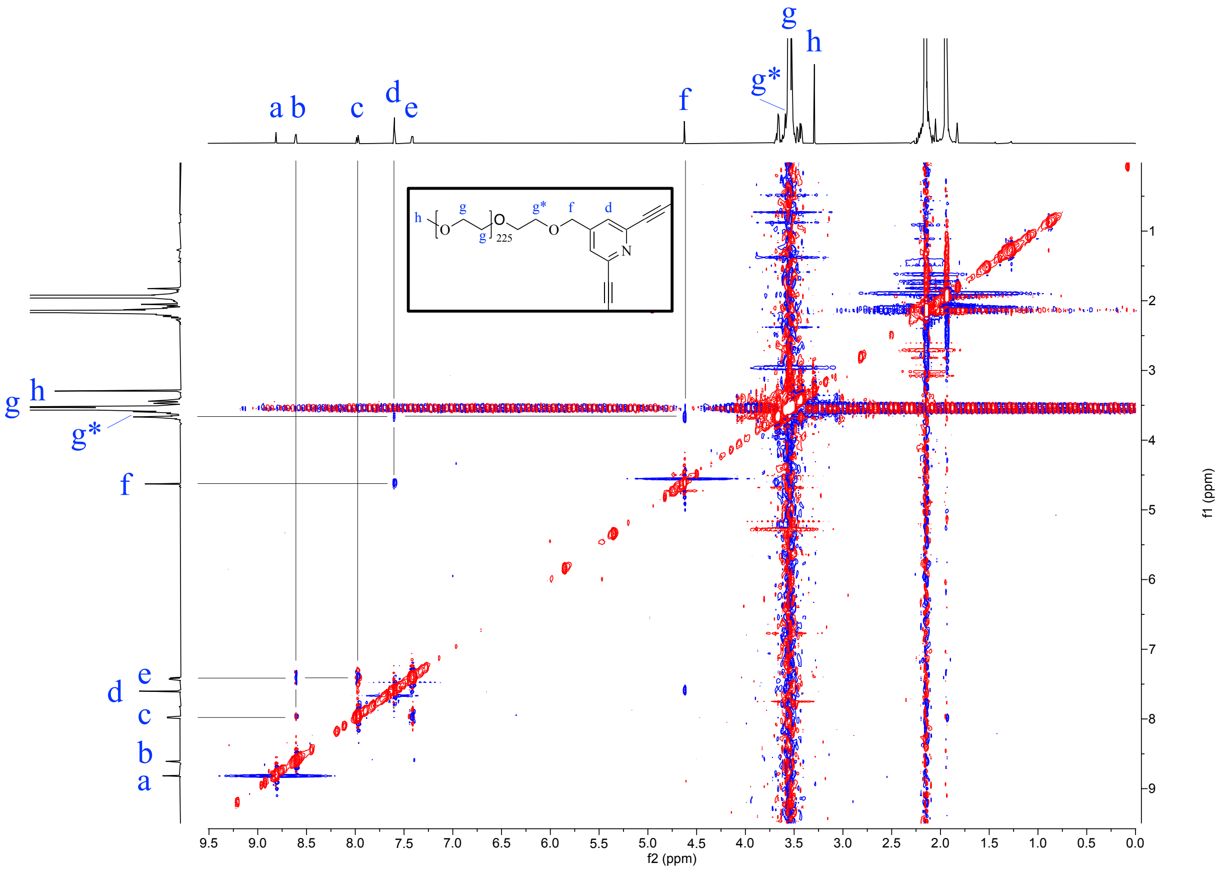

Figure S175: ^{1}H-^{1}H NOESY spectrum (600 MHz, CD_3CN, 298 K) of **PolyL3**.

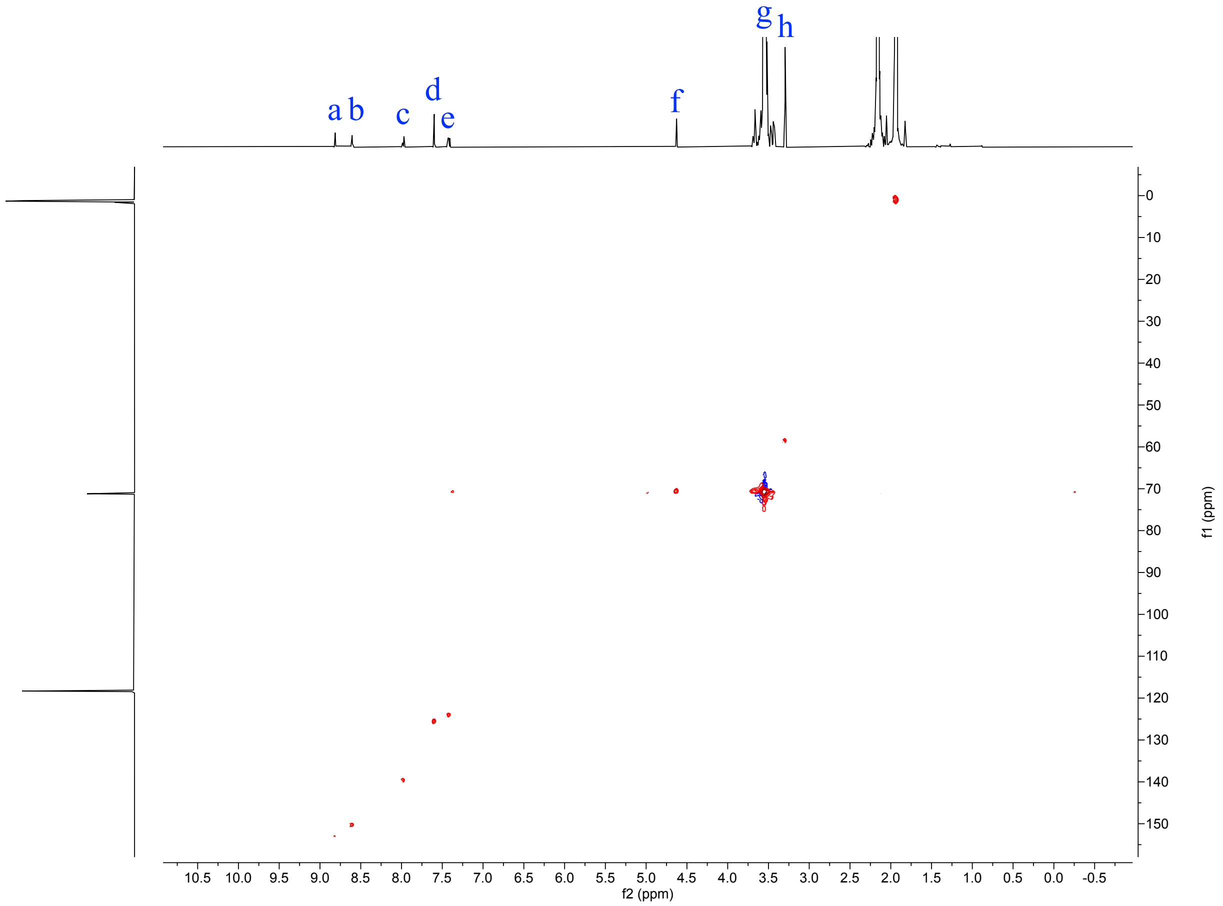

Figure S176: ^{1}H-^{13}C HSQC spectrum (600 MHz, CD_3CN, 298 K) of **PolyL3**.

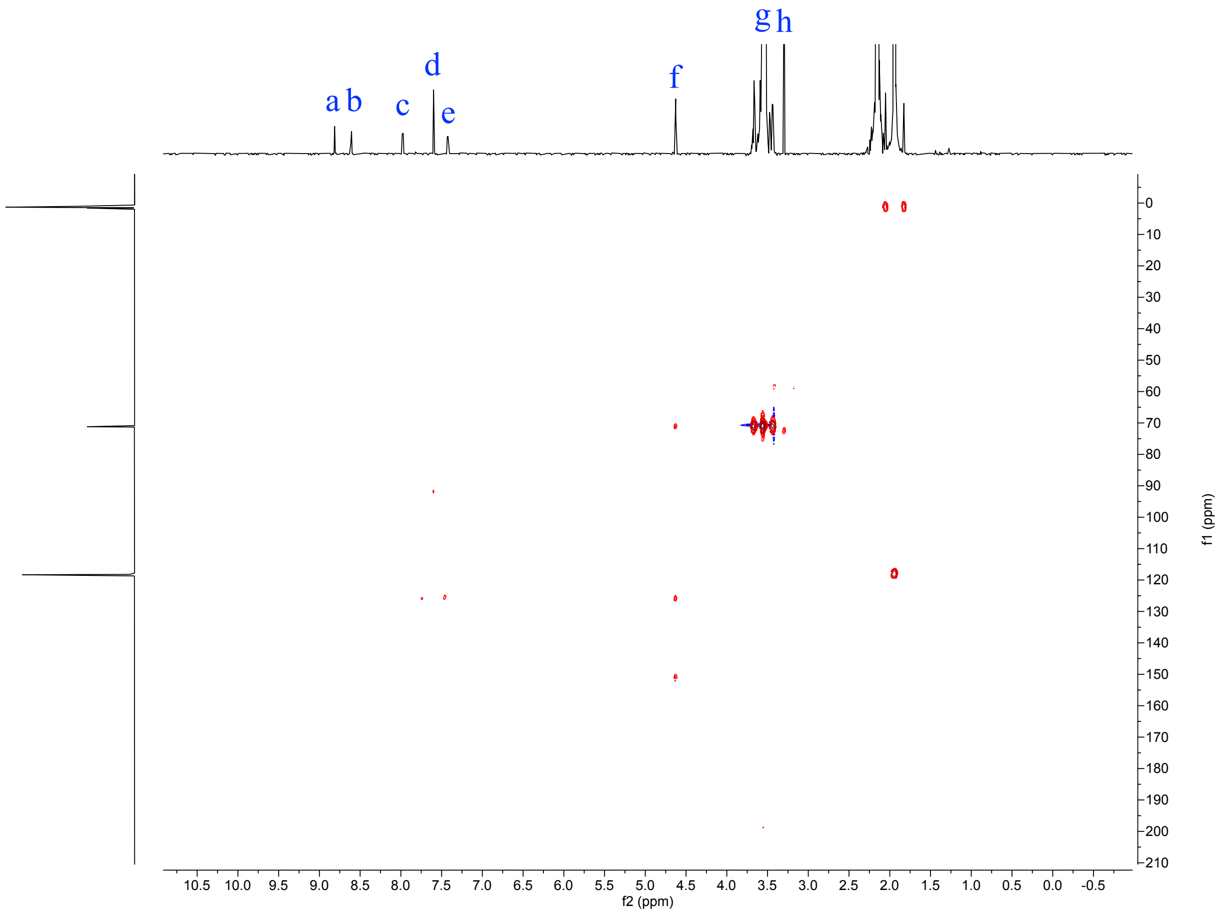

Figure S177: ^1H-^{13}C HMBC spectrum (600 MHz, CD_3CN, 298 K) of **PolyL3**.

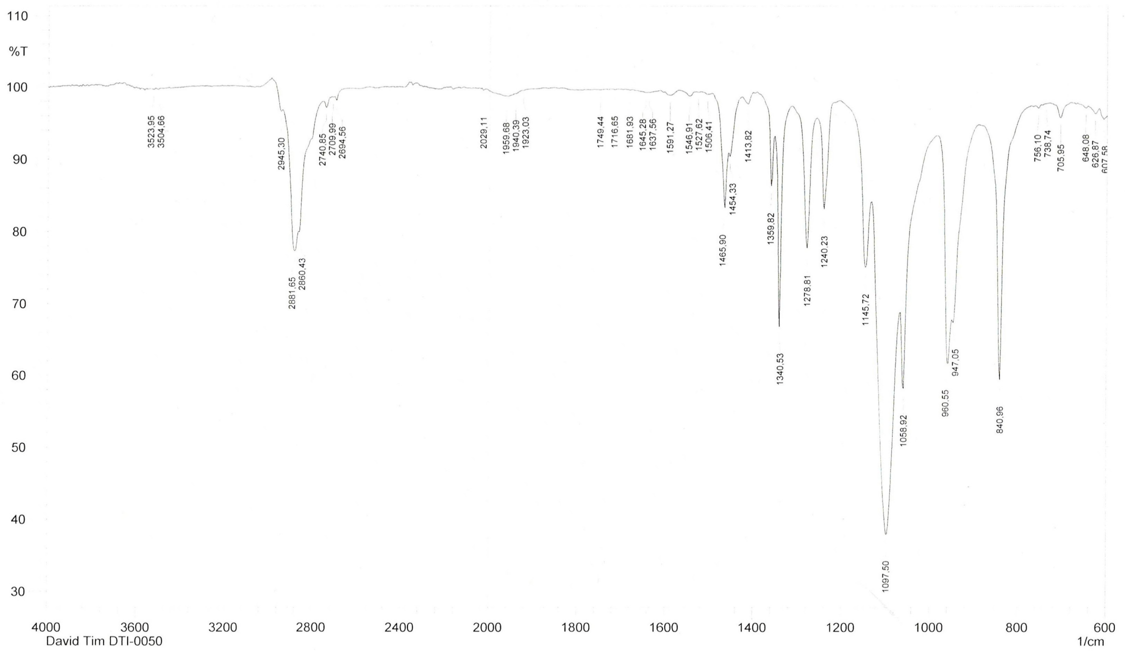

Figure S178: IR spectrum of **PolyL3**.

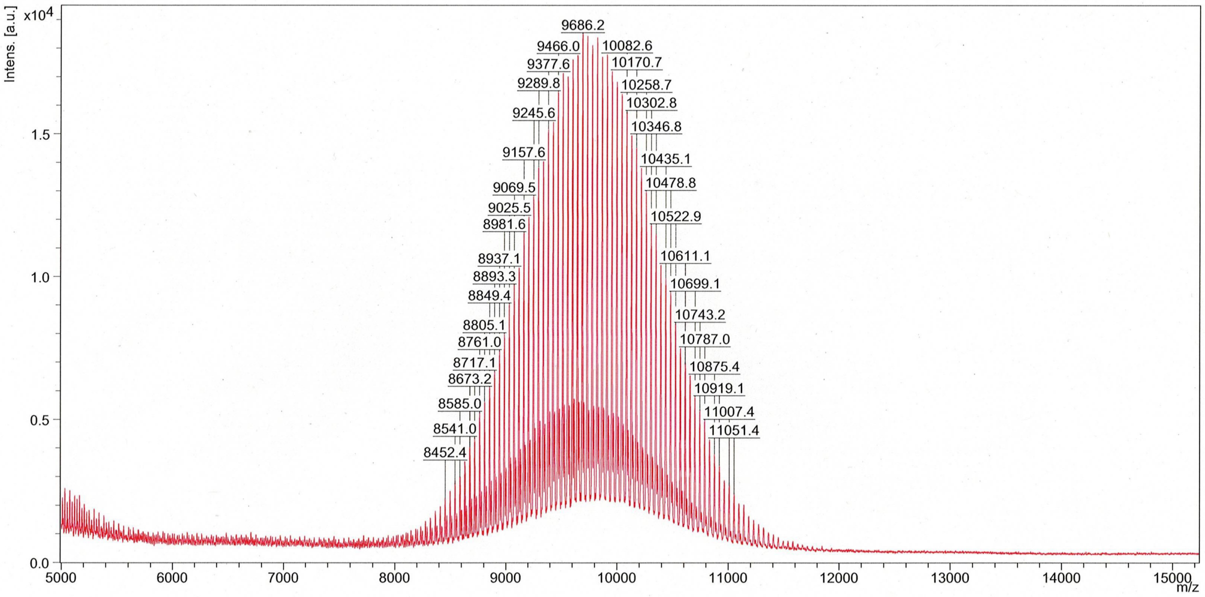

Figure S179: MALDI-MS spectrum of **PolyL3**.

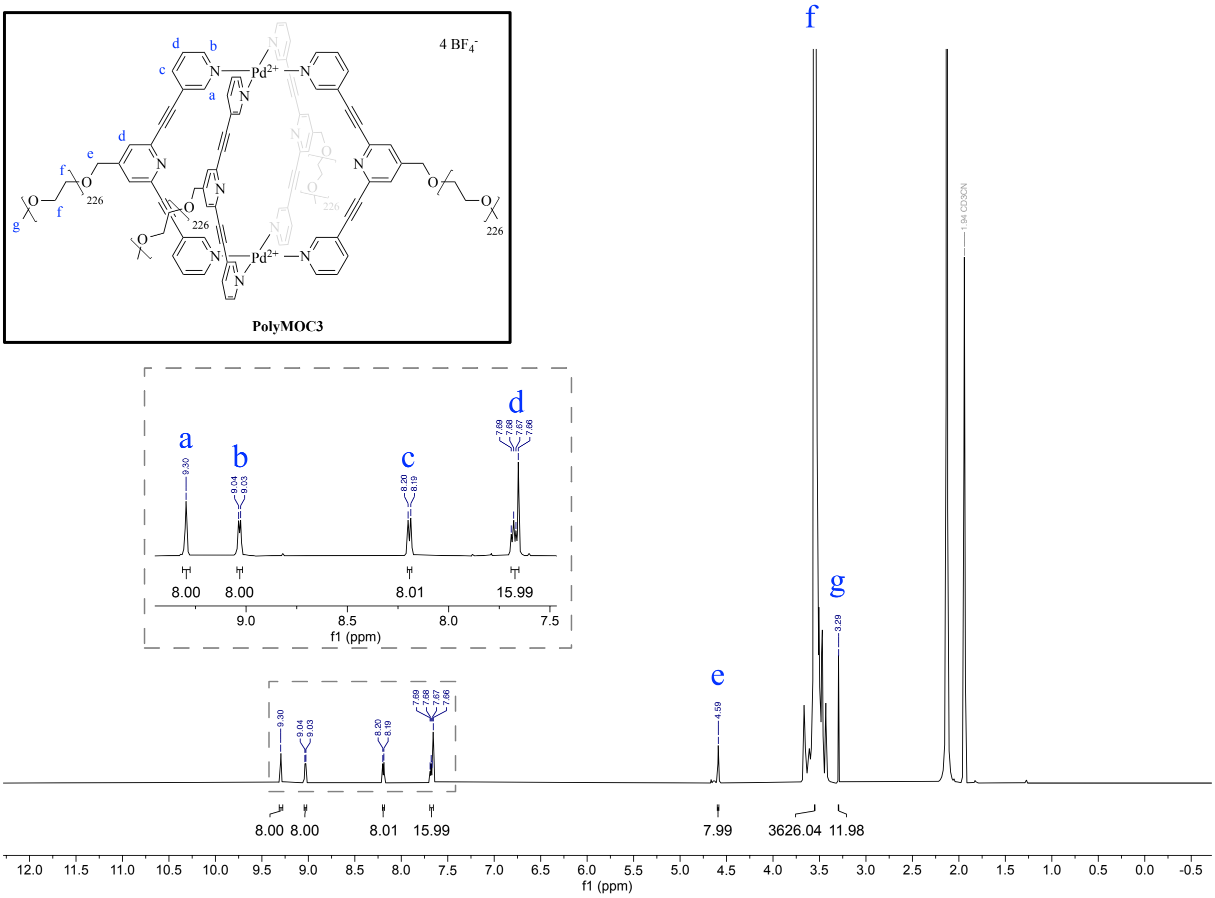

Figure S180: ^{1}H NMR spectrum (600 MHz, CD_3CN, 298 K) of **PolyMOC3**.

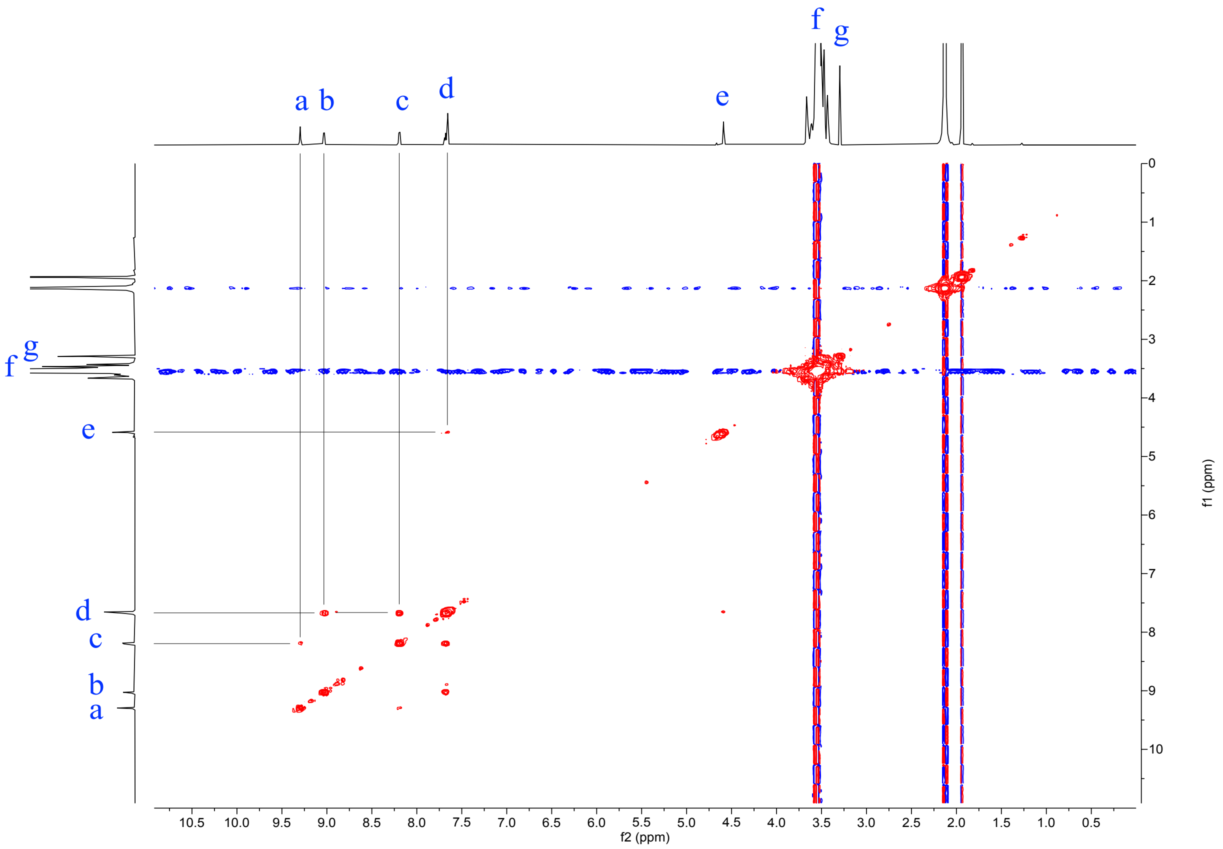

Figure S181: ^{1}H-^{1}H COSY spectrum (600 MHz, CD_3CN, 298 K) of **PolyMOC3**.

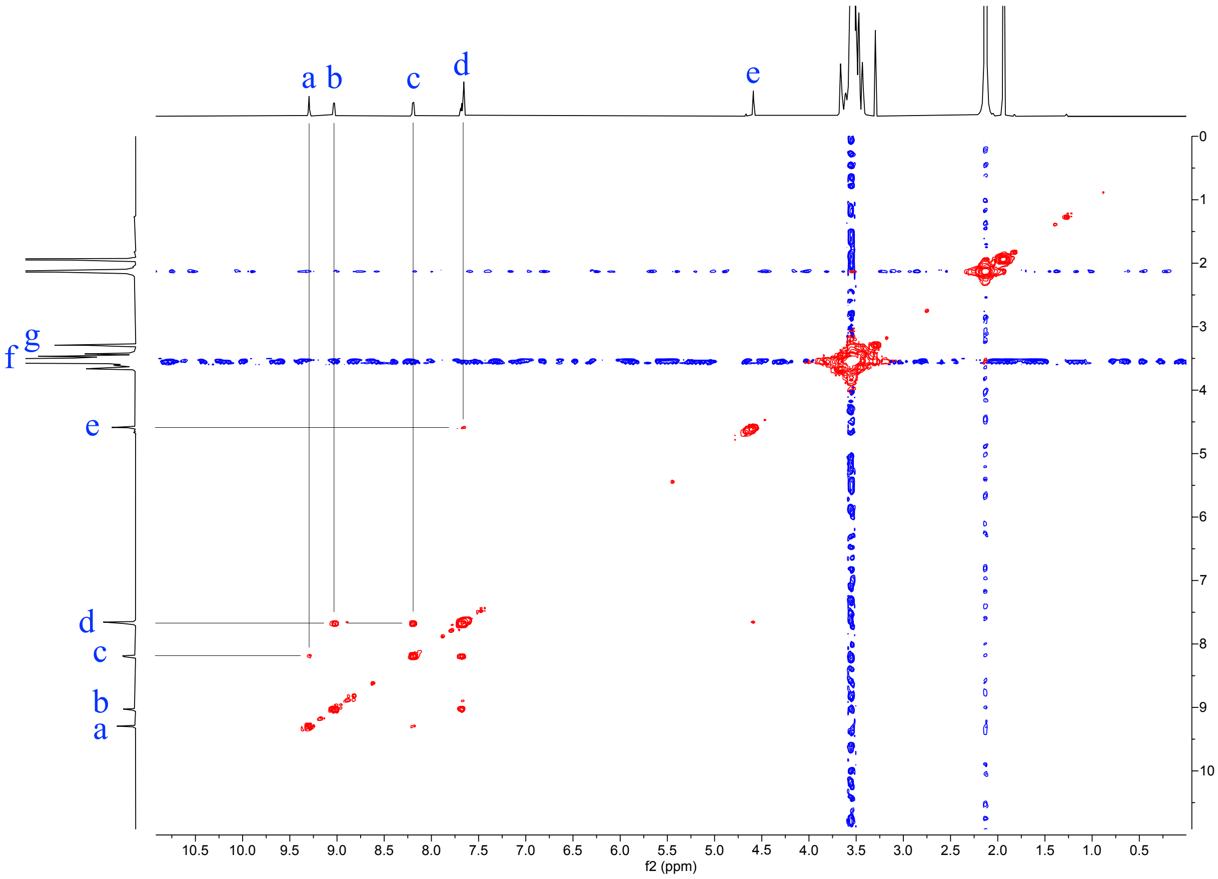

Figure S182: ^{1}H-^{1}H NOESY spectrum(600 MHz, CD_3CN, 298 K) of **PolyMOC3**.

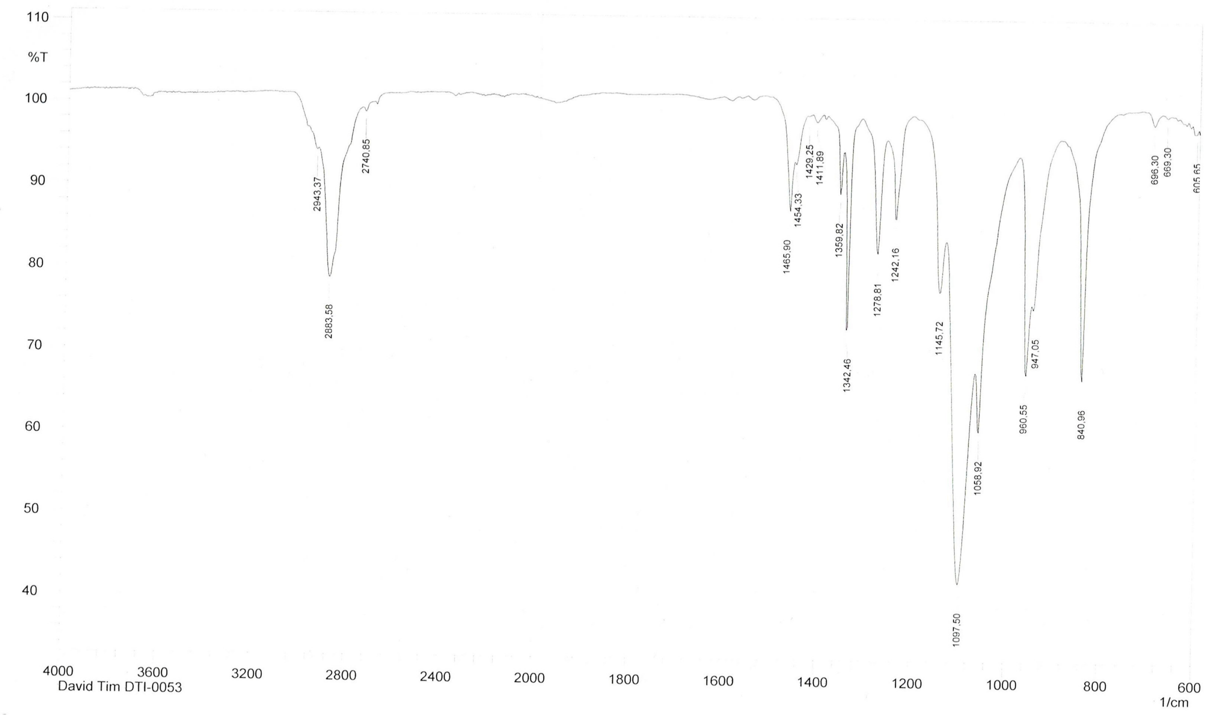

Figure S183: IR spectrum of **PolyMOC3**.

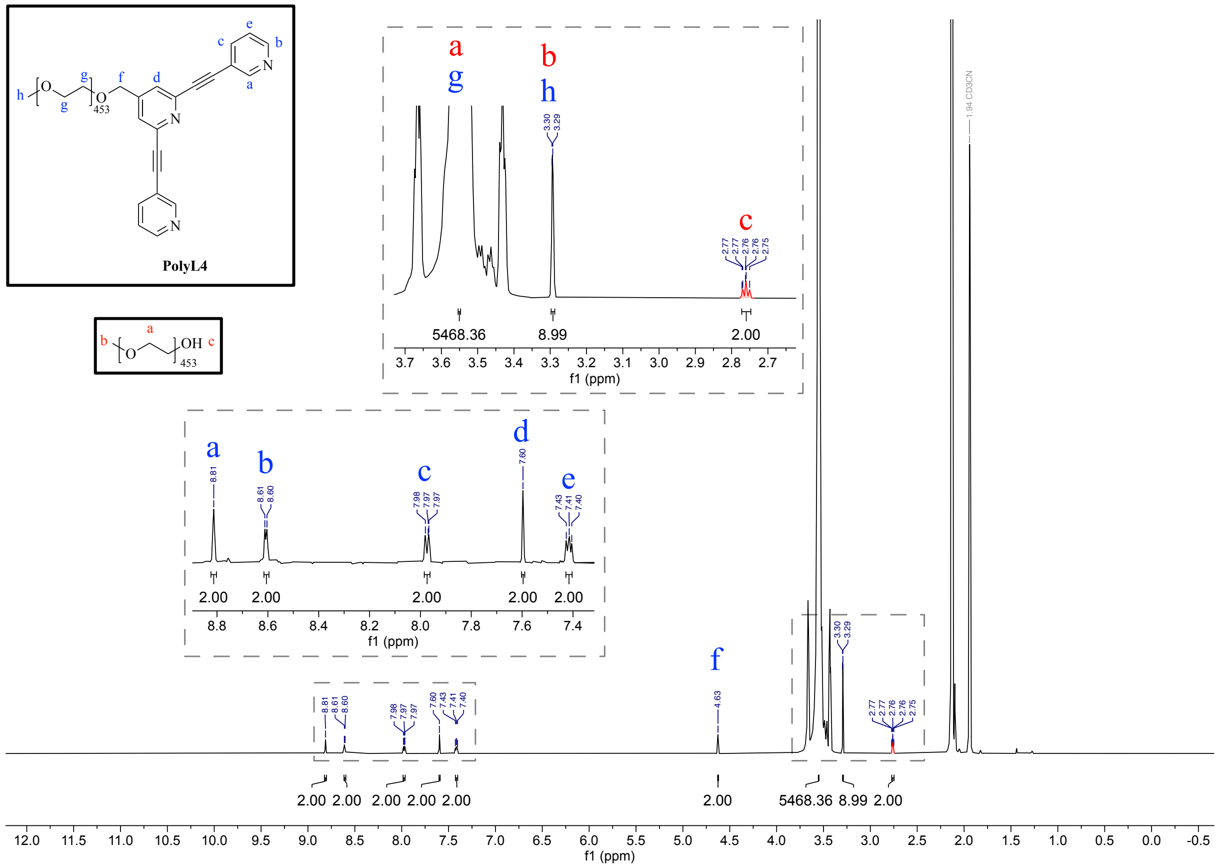

Figure S184: ^{1}H NMR spectrum (600 MHz, CD_3CN, 298 K) of **PolyL4**. Letters in red and the red-colored signal (c) correspond to the residual $mPEG_{20kDa}$.

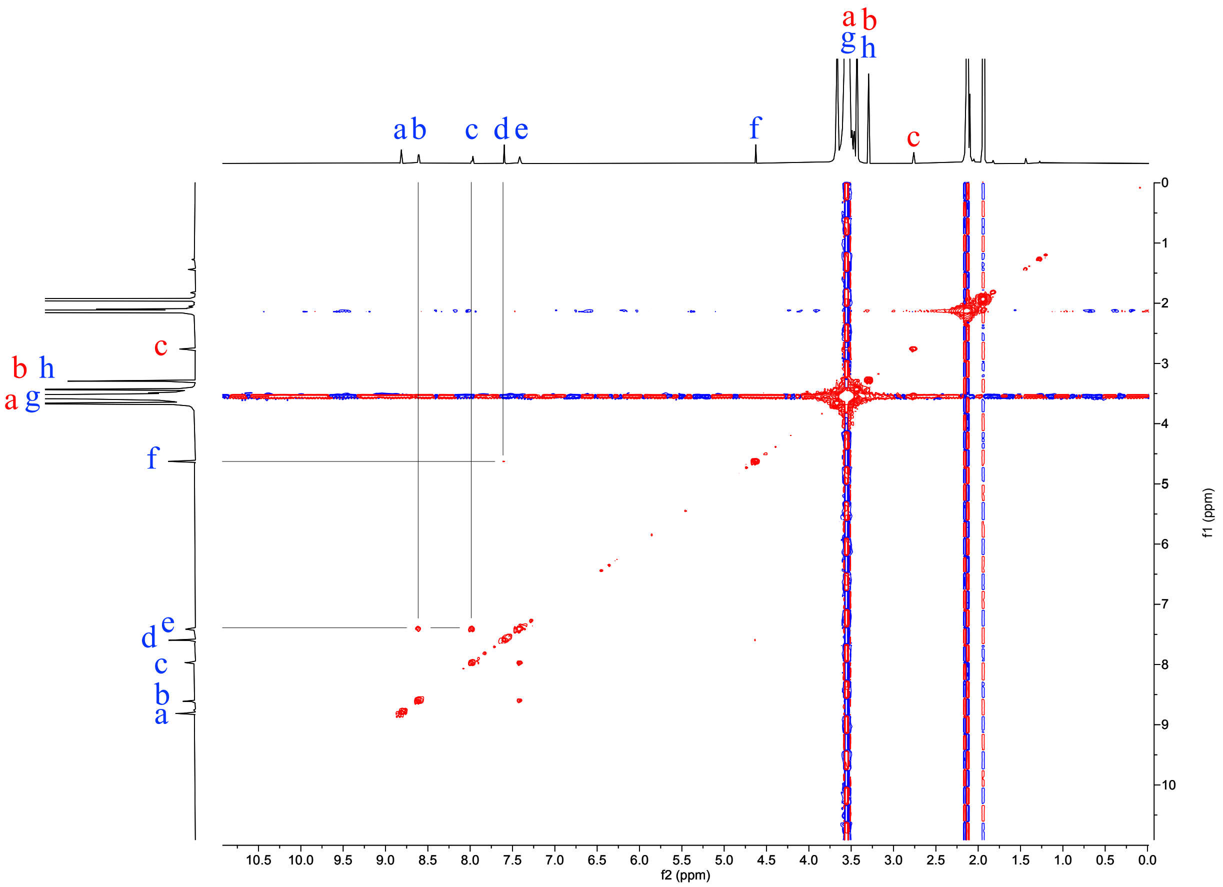

Figure S185: ^{1}H-^{1}H COSY spectrum (600 MHz, CD_3CN, 298 K) of **PolyL4**. Letters in red and the red-colored signal (c) correspond to the residual $mPEG_{20kDa}$.

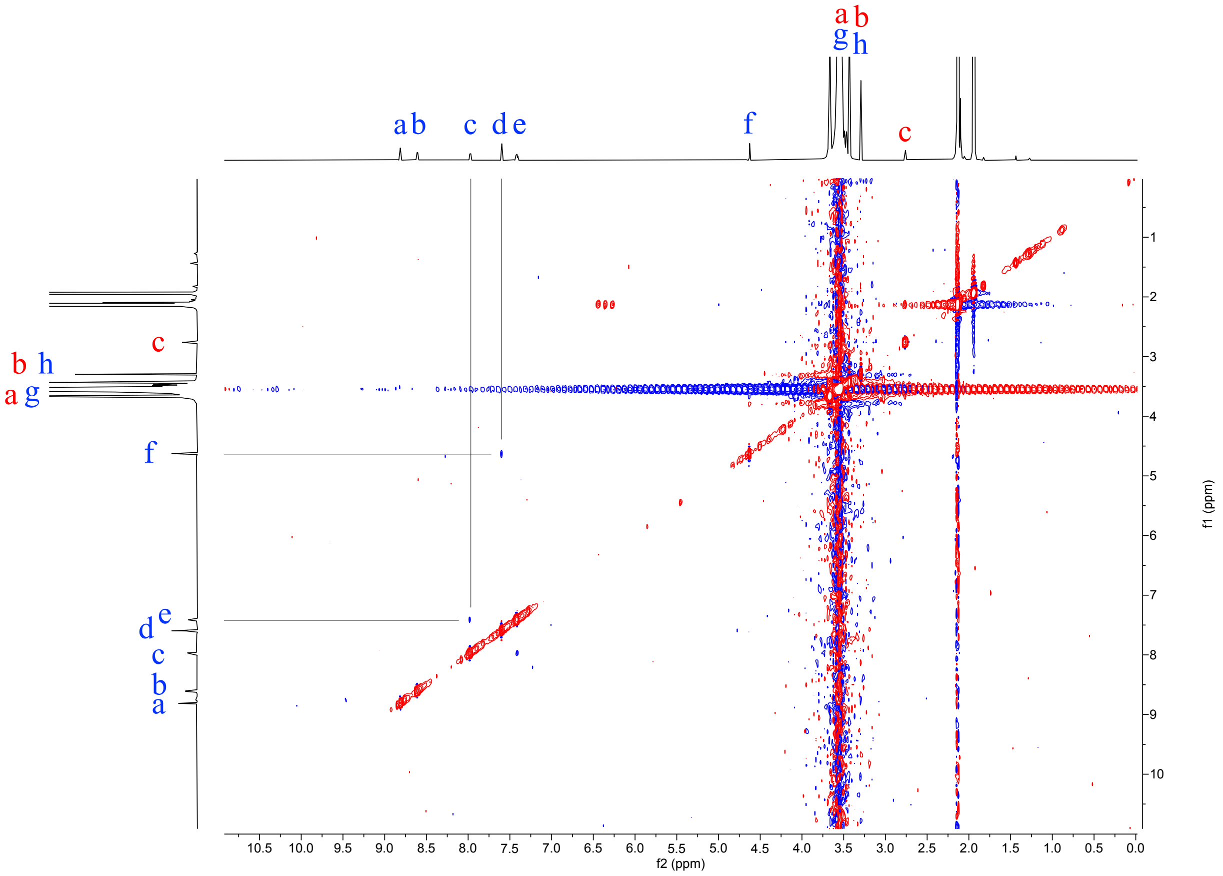

Figure S186: ^{1}H-^{1}H NOESY spectrum (600 MHz, CD_3CN, 298 K) of **PolyL4**. Letters in red and the red-colored signal (c) correspond to the residual $mPEG_{20kDa}$.

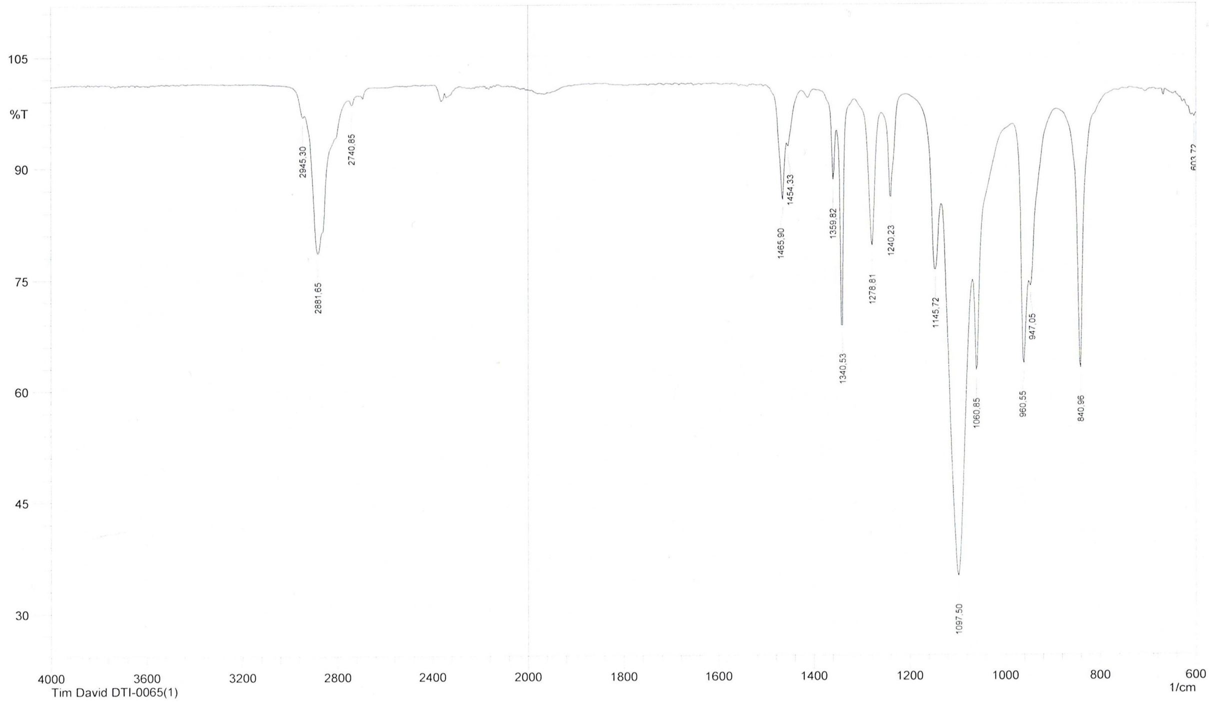

Figure S187: IR spectrum of **PolyL4**.

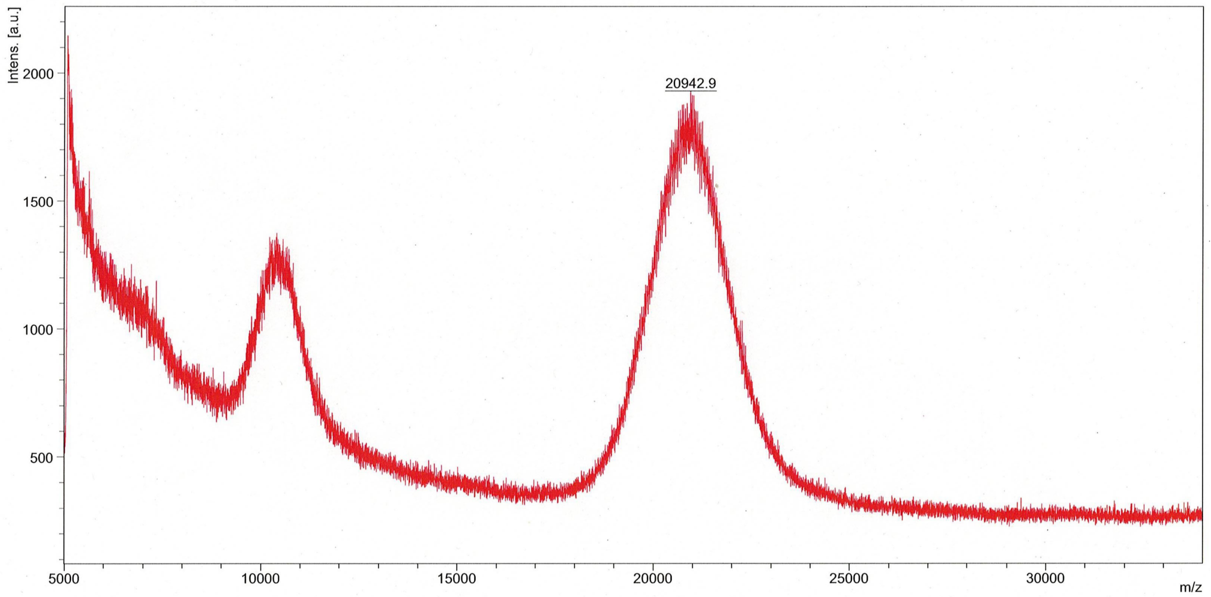

Figure S188: MALDI-MS spectrum of **PolyL4**.

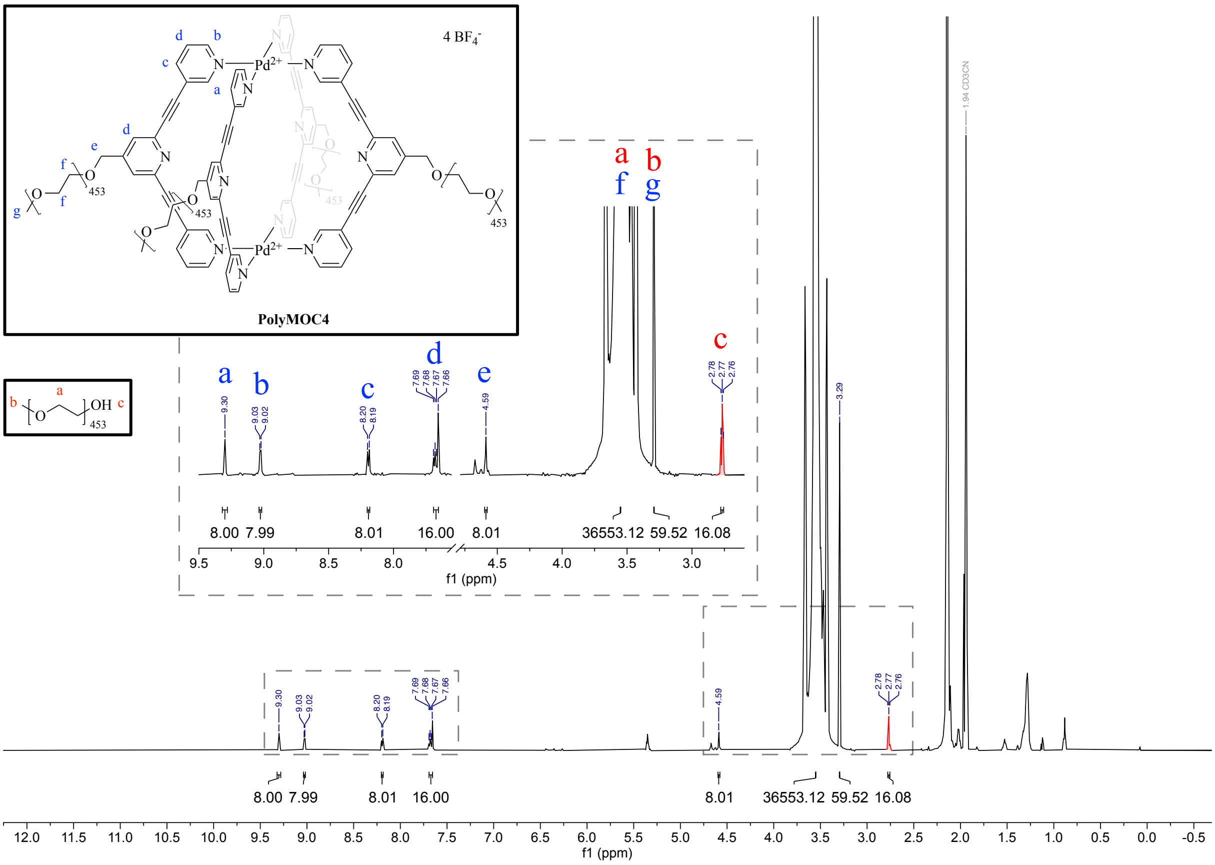

Figure S189: ^{1}H NMR spectrum (600 MHz, CD_3CN, 298 K) of **PolyMOC4**. Letters in red and the red-colored signal (c) correspond to the residual $mPEG_{20kDa}$.

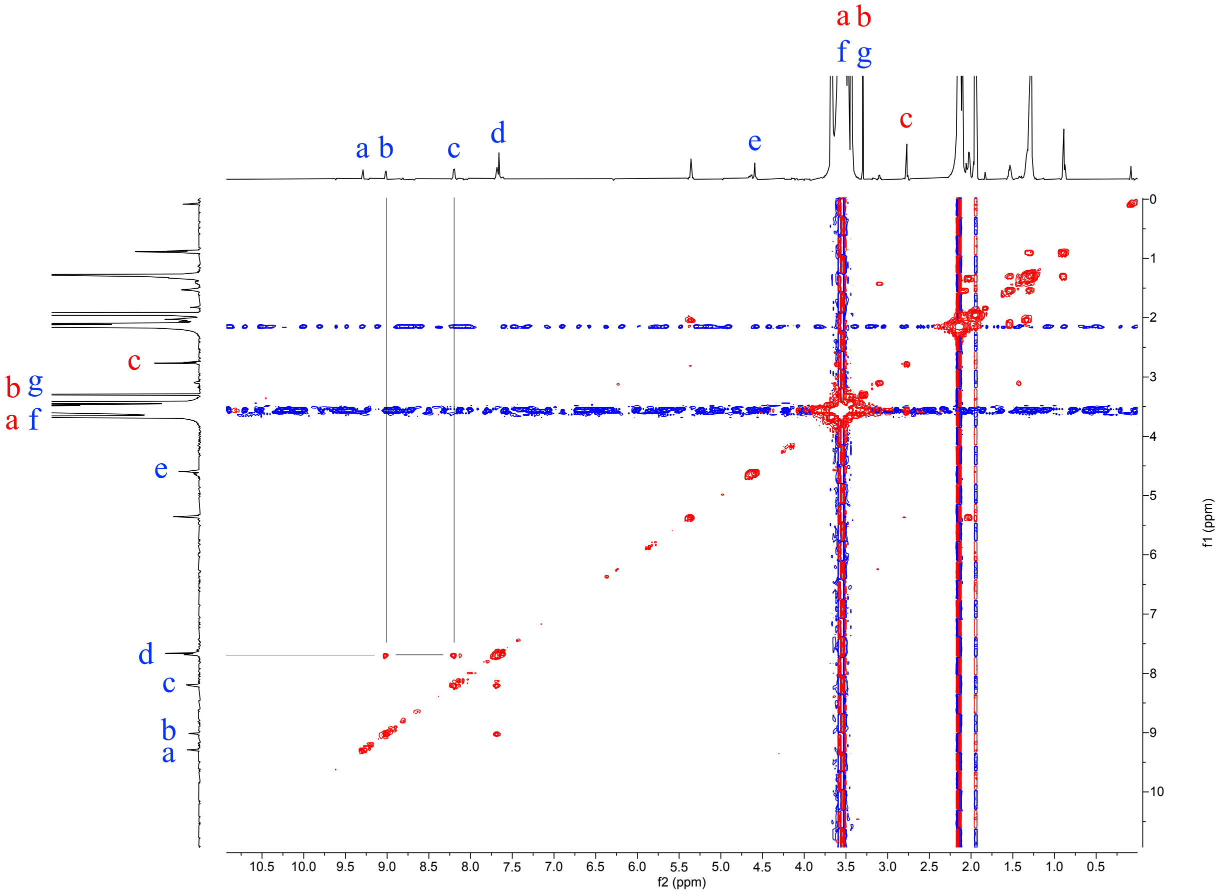

Figure S190: ^{1}H-^{1}H COSY spectrum (600 MHz, CD_3CN, 298 K) of **PolyMOC4**. Letters in red and the red-colored signal (c) correspond to the residual $mPEG_{20kDa}$.

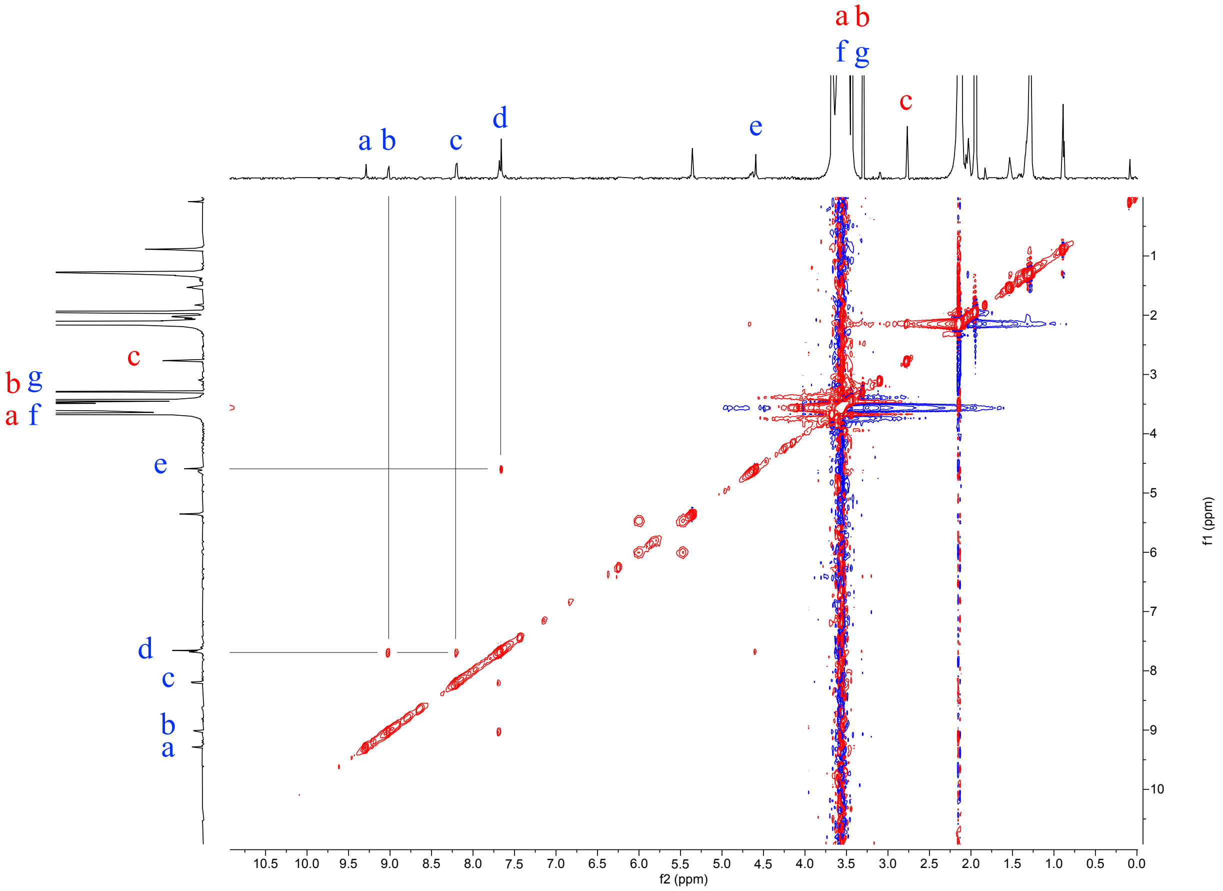

Figure S191: ^{1}H-^{1}H NOESY spectrum (600 MHz, CD_3CN, 298 K) of **PolyMOC4**. Letters in red and the red-colored signal (c) correspond to the residual $mPEG_{20kDa}$.

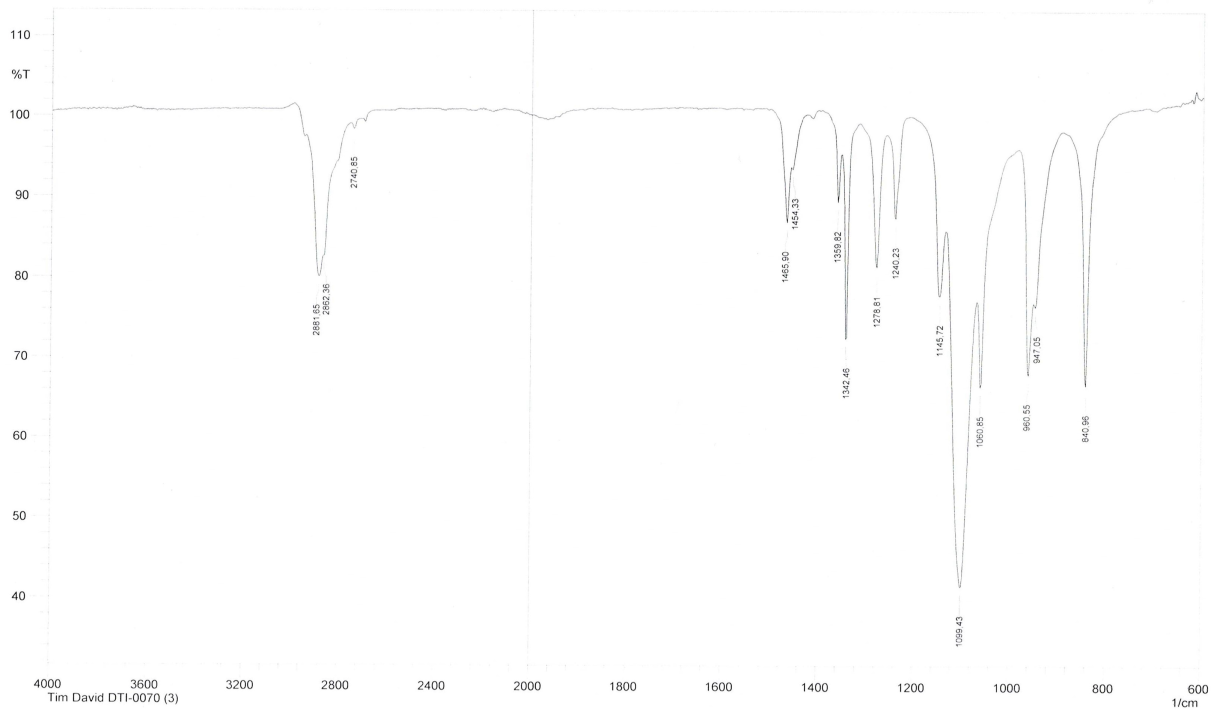

Figure S192: IR spectrum of **PolyMOC4**.

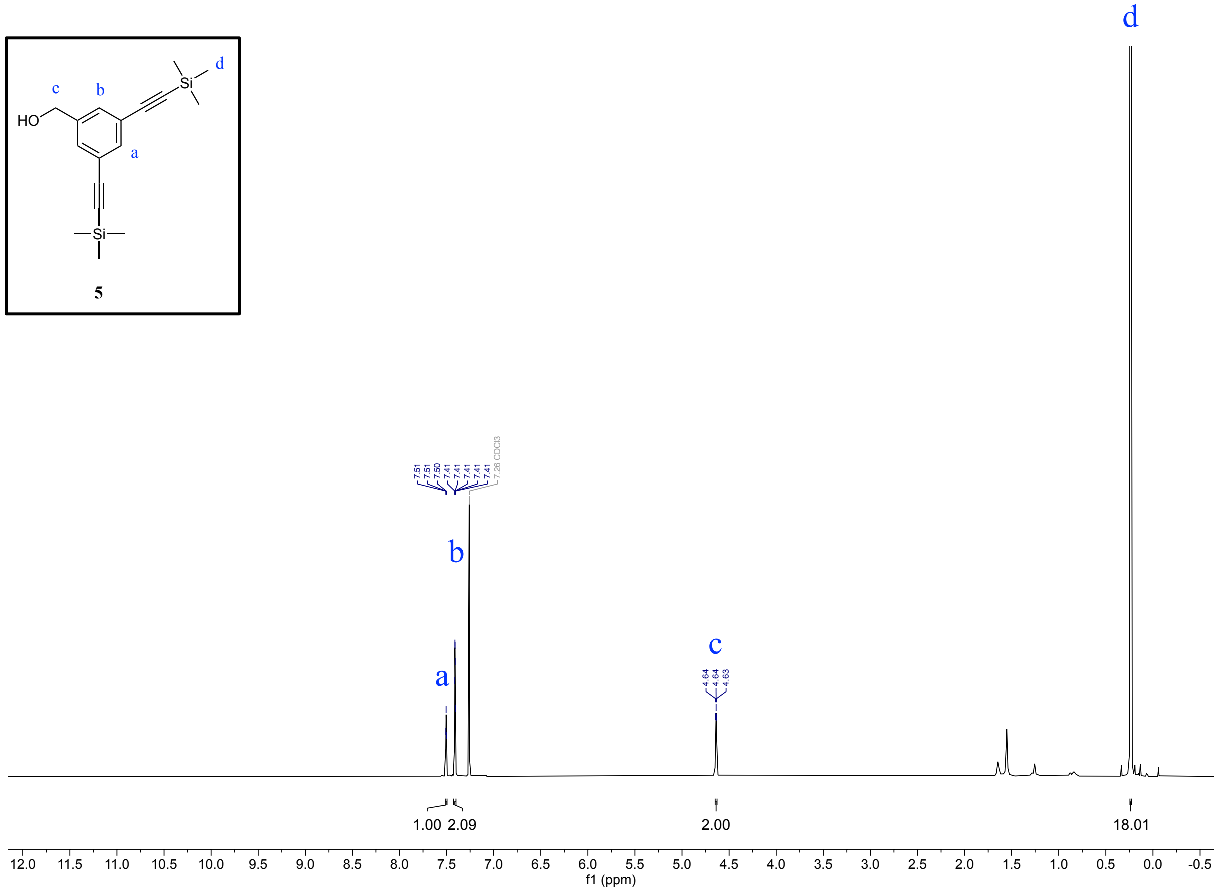

Figure S193: ^{1}H NMR spectrum (600 MHz, $CDCl_3$, 298 K) of **5**.

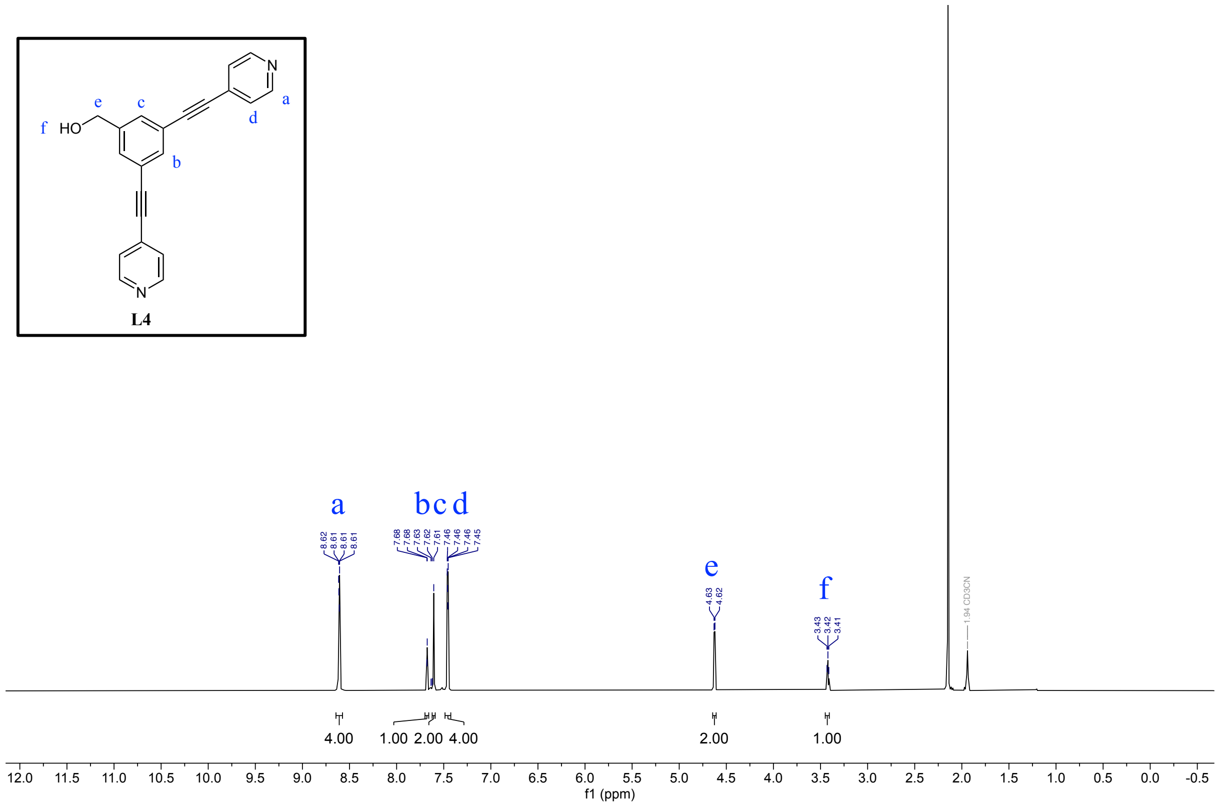

Figure S194: ^{1}H NMR spectrum (600 MHz, $CDCl_3$, 298 K) of **L4**.

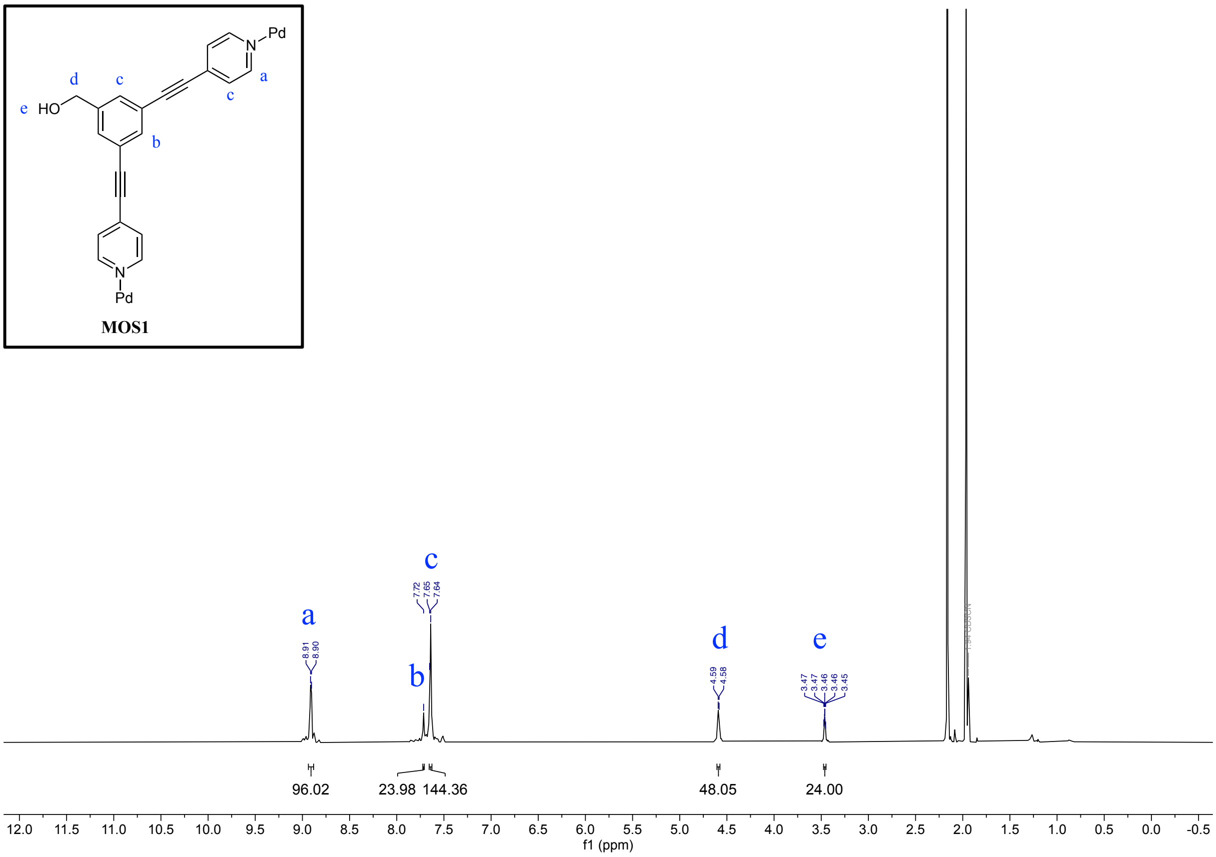

Figure S195: ^{1}H NMR spectrum (600 MHz, $CDCl_3$, 298 K) of **MOS1**.

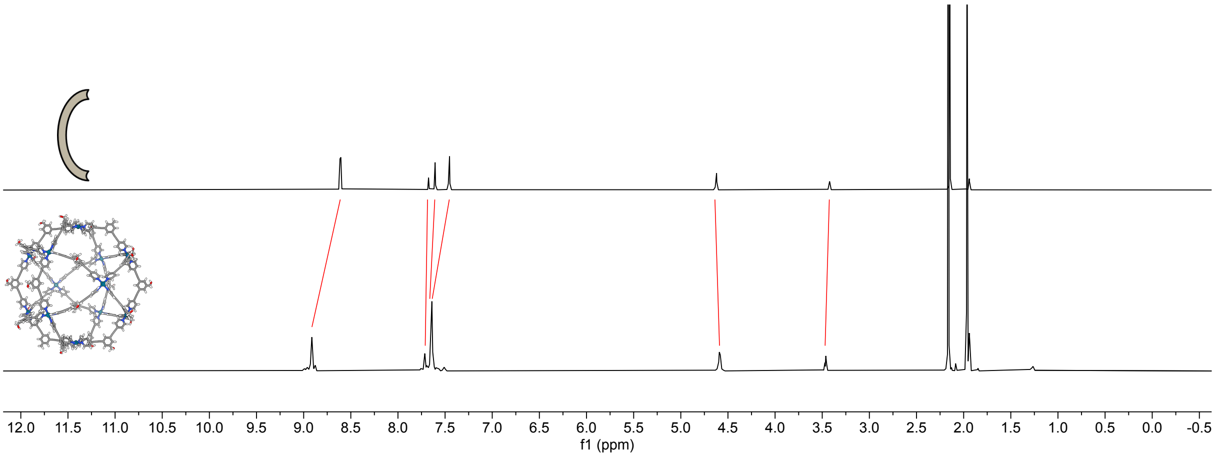

Figure S196: Stacked ^{1}H NMR spectra (600 MHz, CD_3CN, 298 K) spectra of **L4** (top) and **MOS1** (bottom).

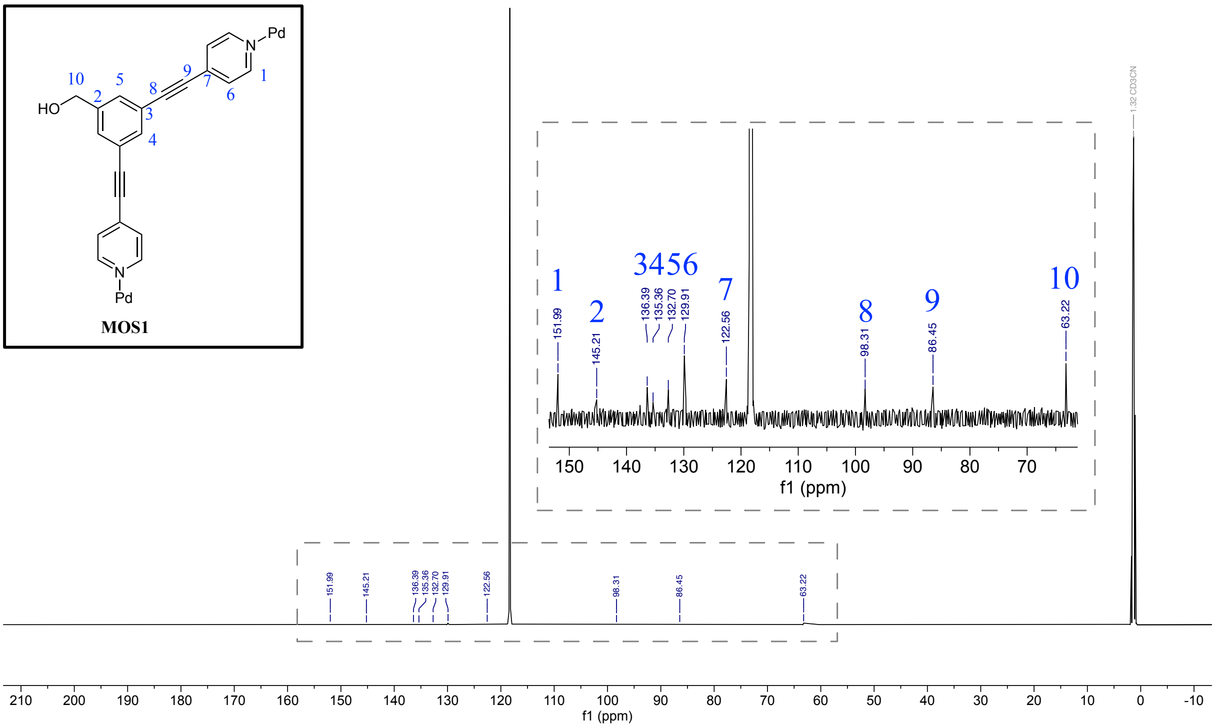

Figure S197: $^{13}C\{^{1}H\}$ NMR spectrum (151 MHz, CD_3CN, 298 K) of **MOS1**.

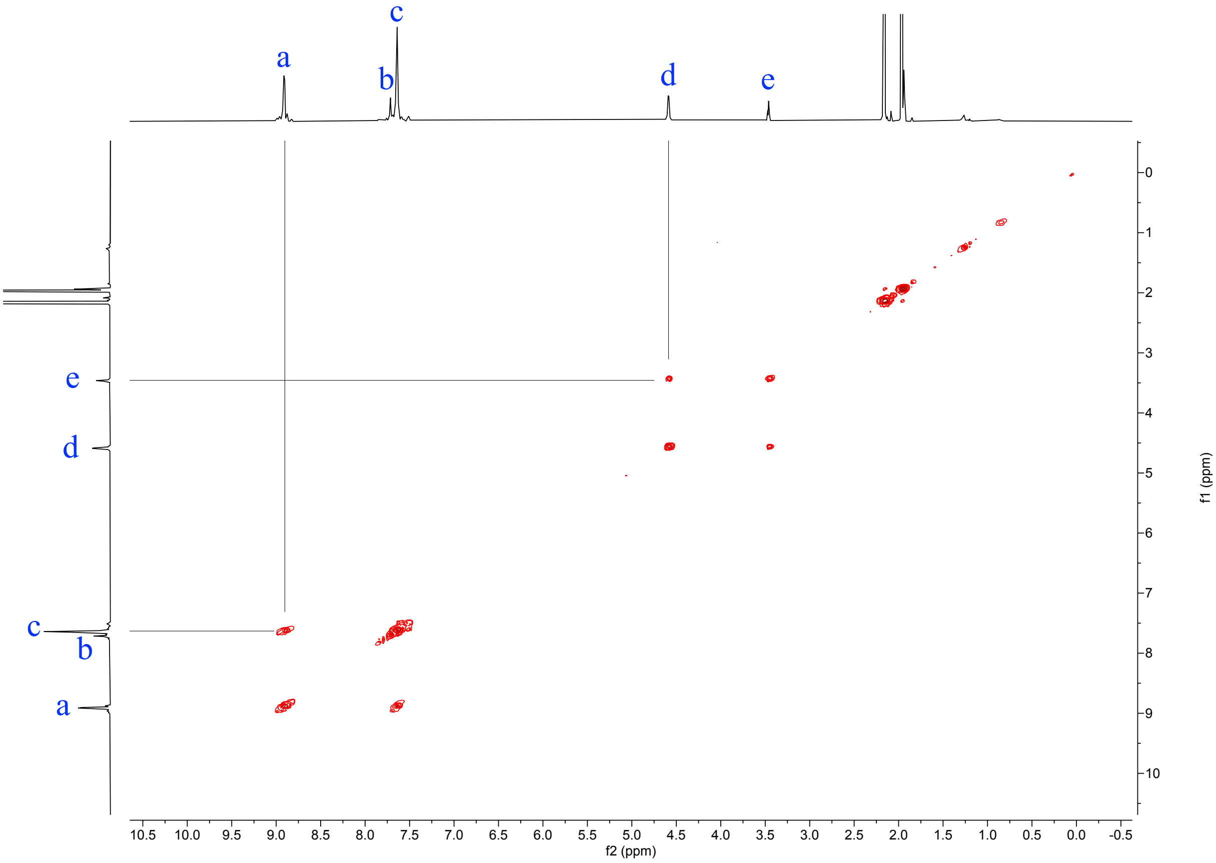

Figure S198: ^{1}H-^{1}H COSY spectrum (600 MHz, CD_3CN, 298 K) of **MOS1**.

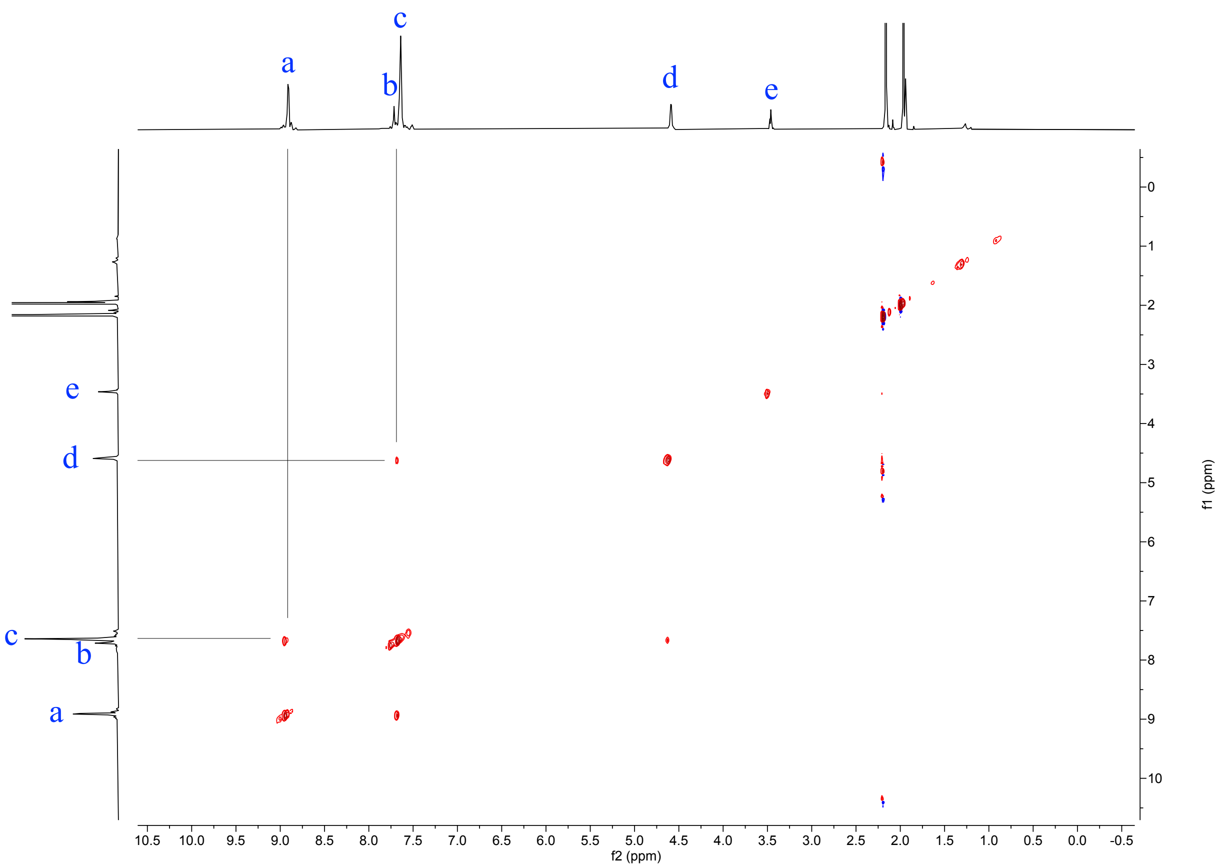

Figure S199: ^{1}H-^{1}H NOESY spectrum (600 MHz, CD_3CN, 298 K) of **MOS1**.

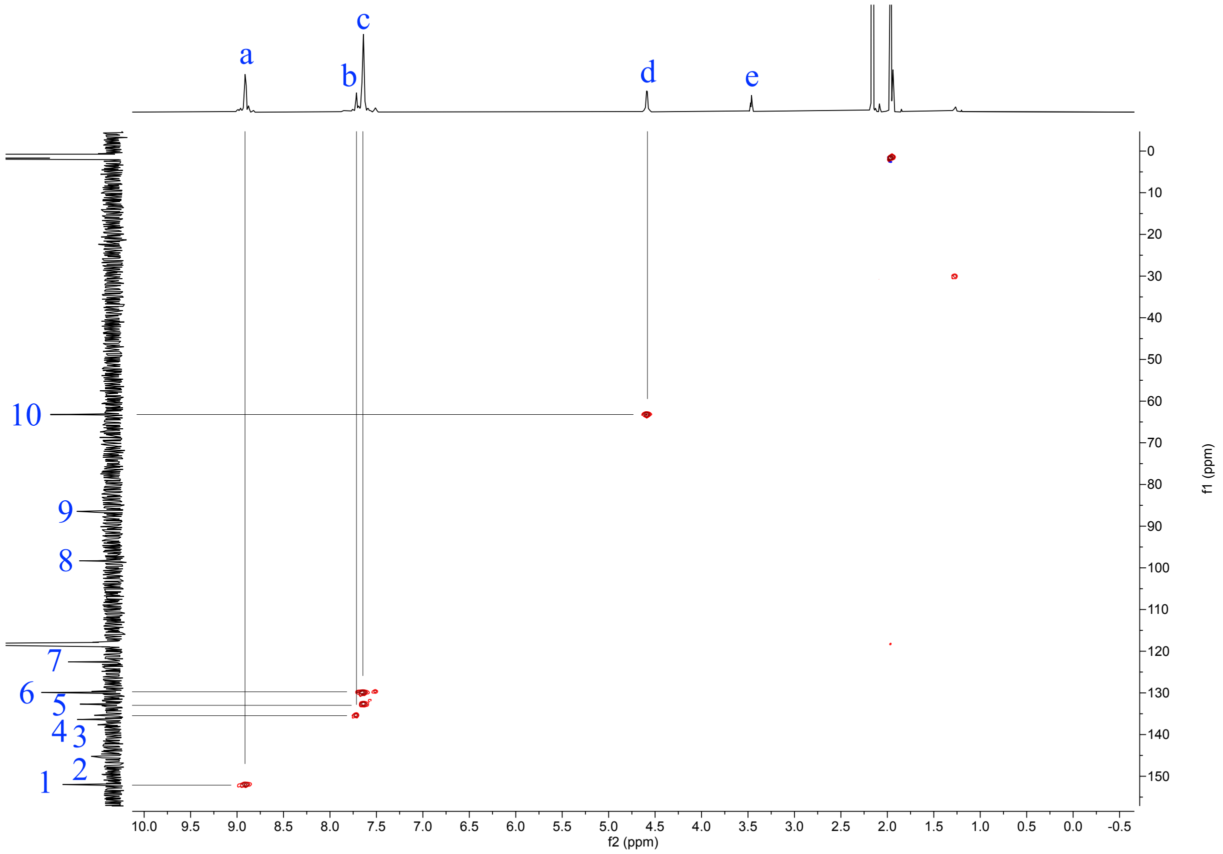

Figure S200: ^{1}H-^{13}C HSQC spectrum (600 MHz, CD_3CN, 298 K) of **MOS1**.

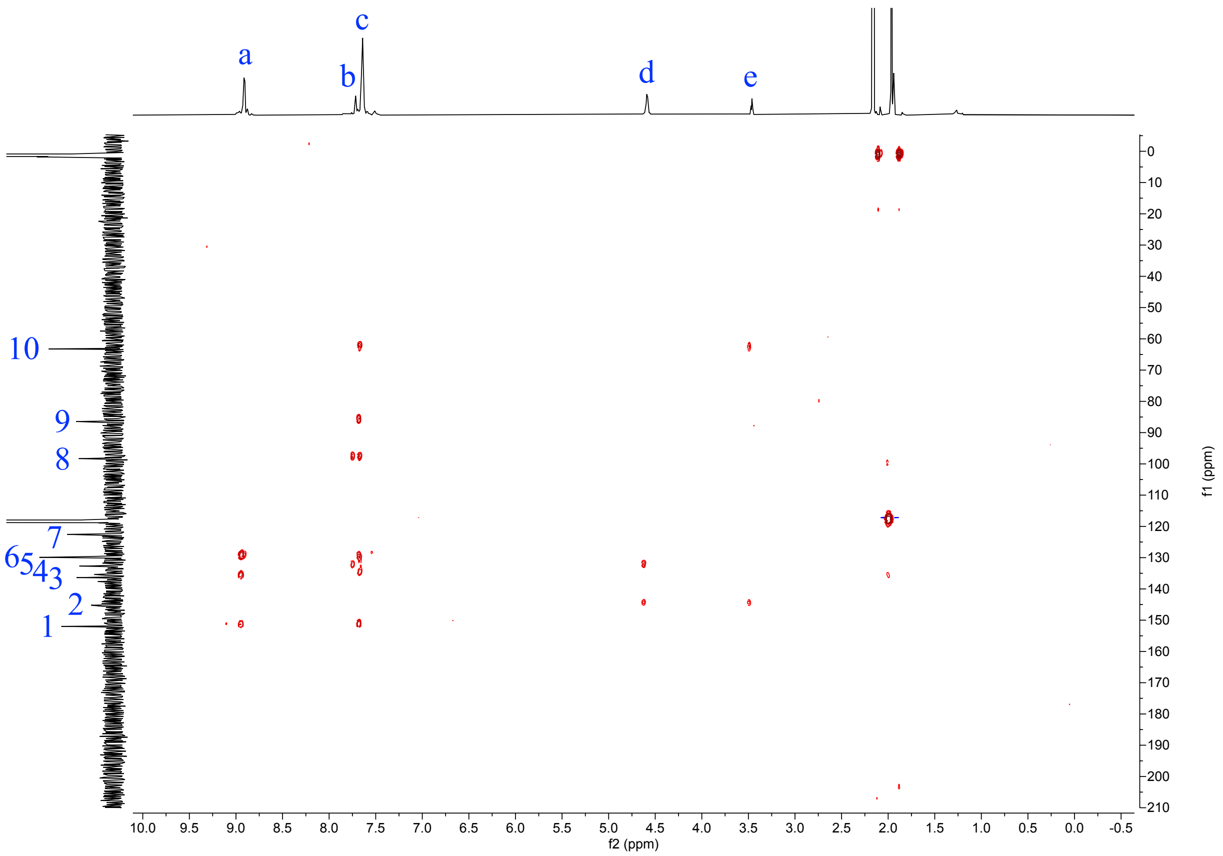

Figure S201: ^{1}H-^{13}C HMBC spectrum (600 MHz, CD_3CN, 298 K) of **MOS1**.

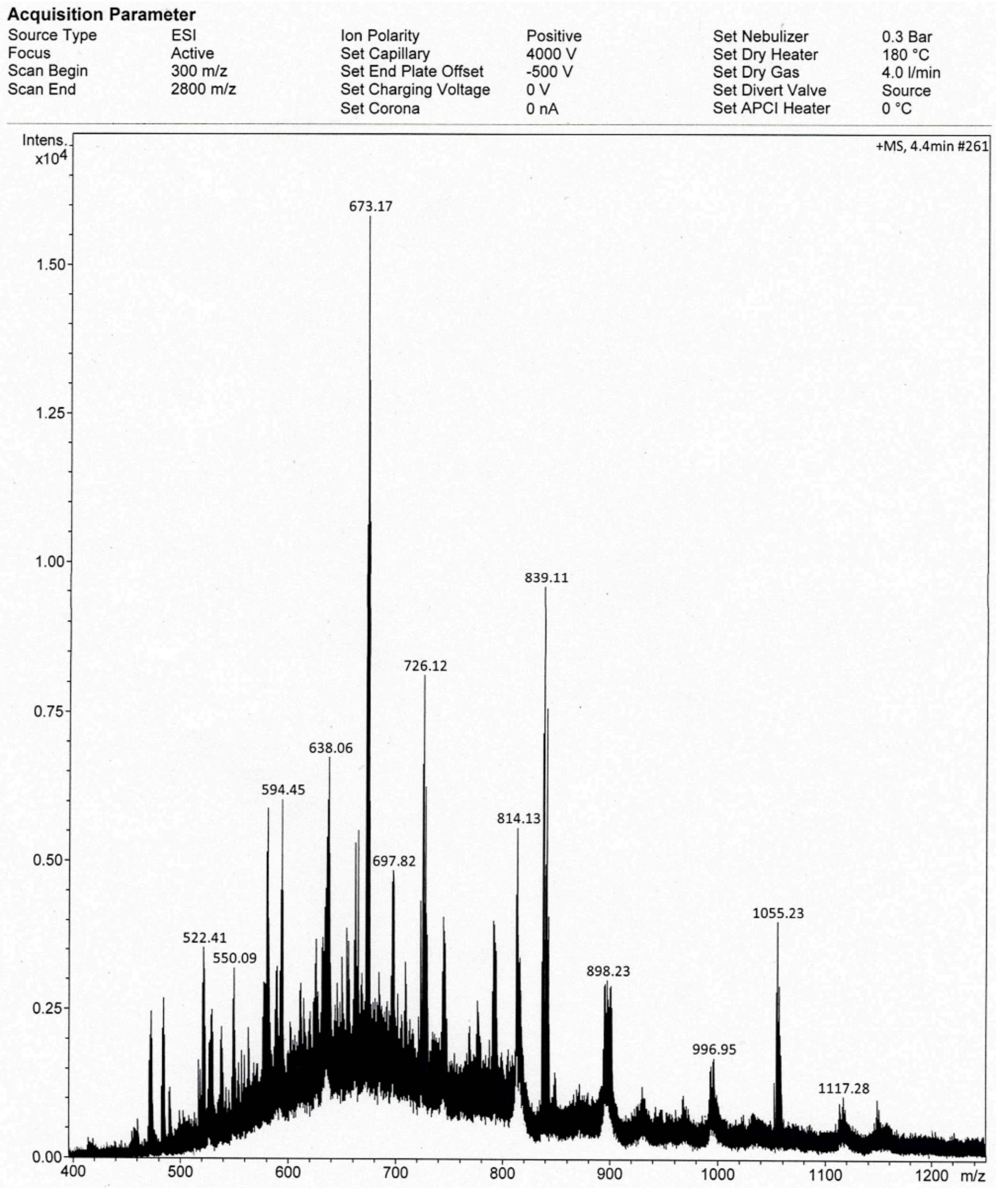

Figure S202: ESI-MS spectrum of **MOS1**.

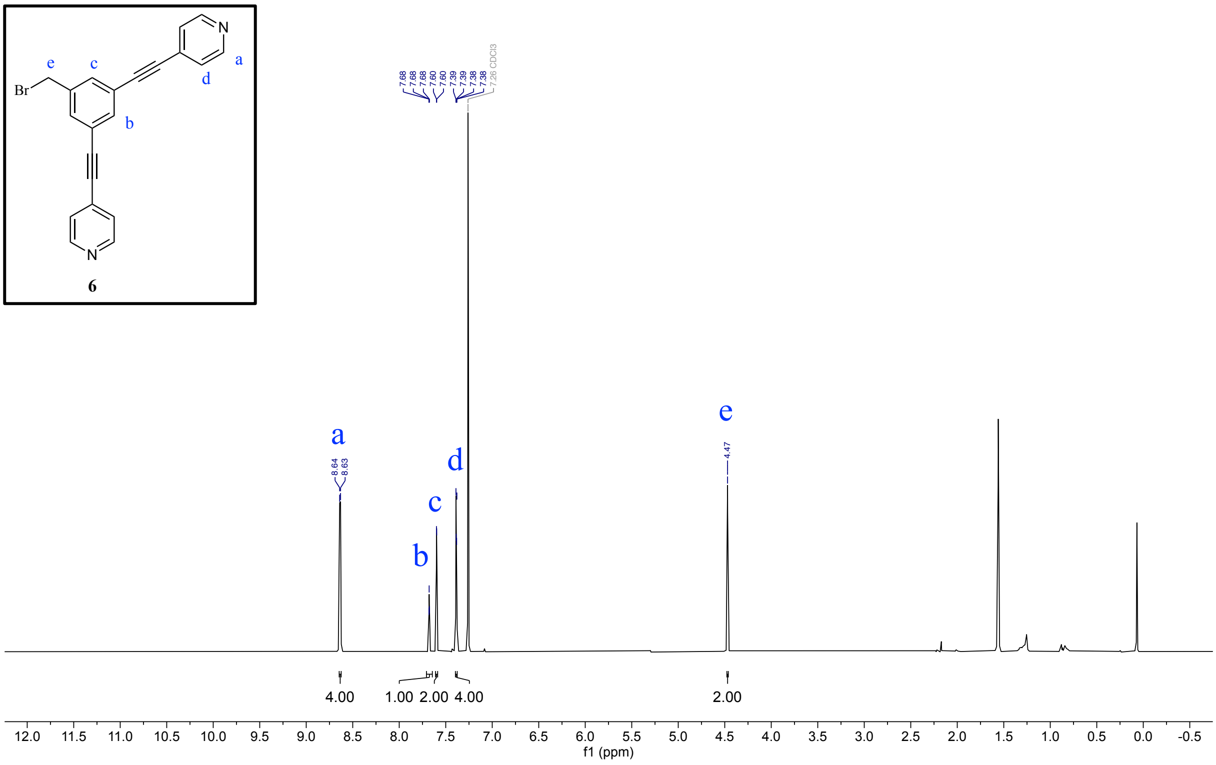

Figure S203: ^{1}H NMR spectrum (600 MHz, $CDCl_3$, 298 K) of **6**.

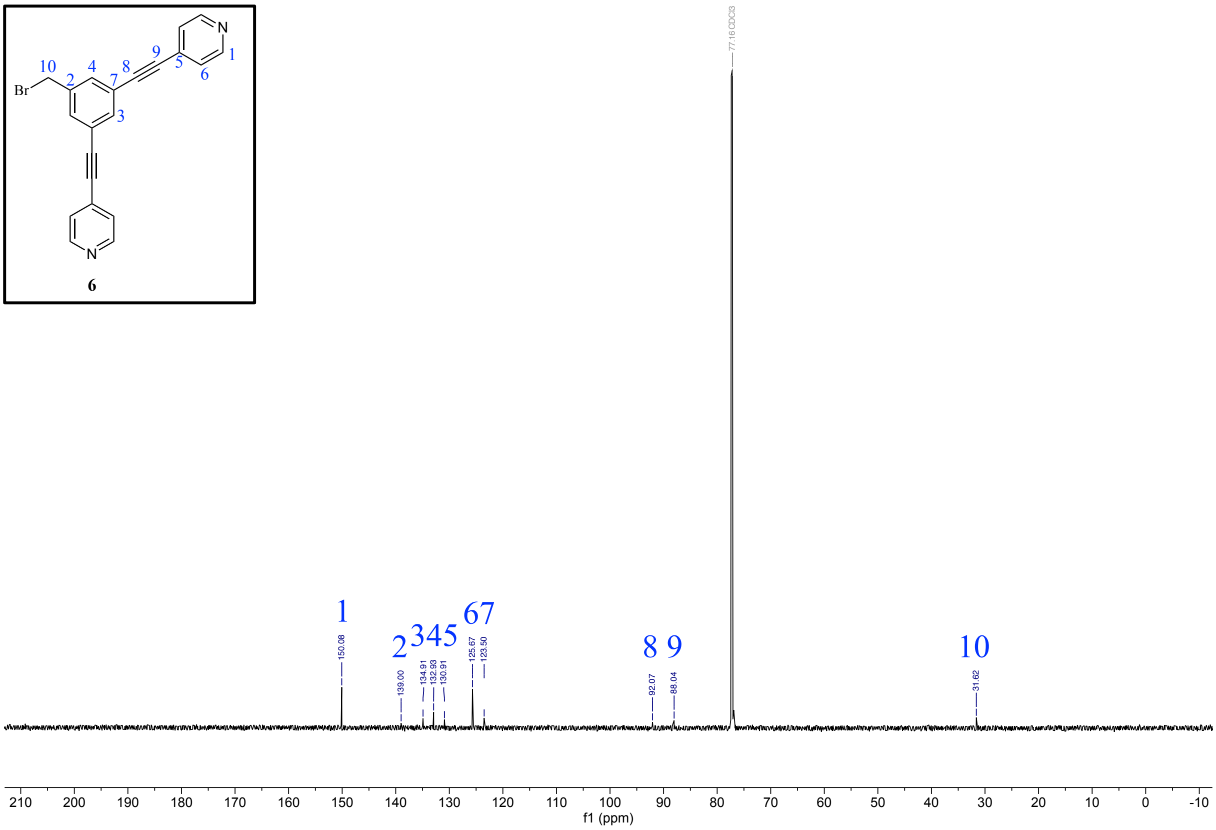

Figure S204: $^{13}C\{^{1}H\}$ NMR spectrum (151 MHz, $CDCl_3$, 298 K) of **6**.

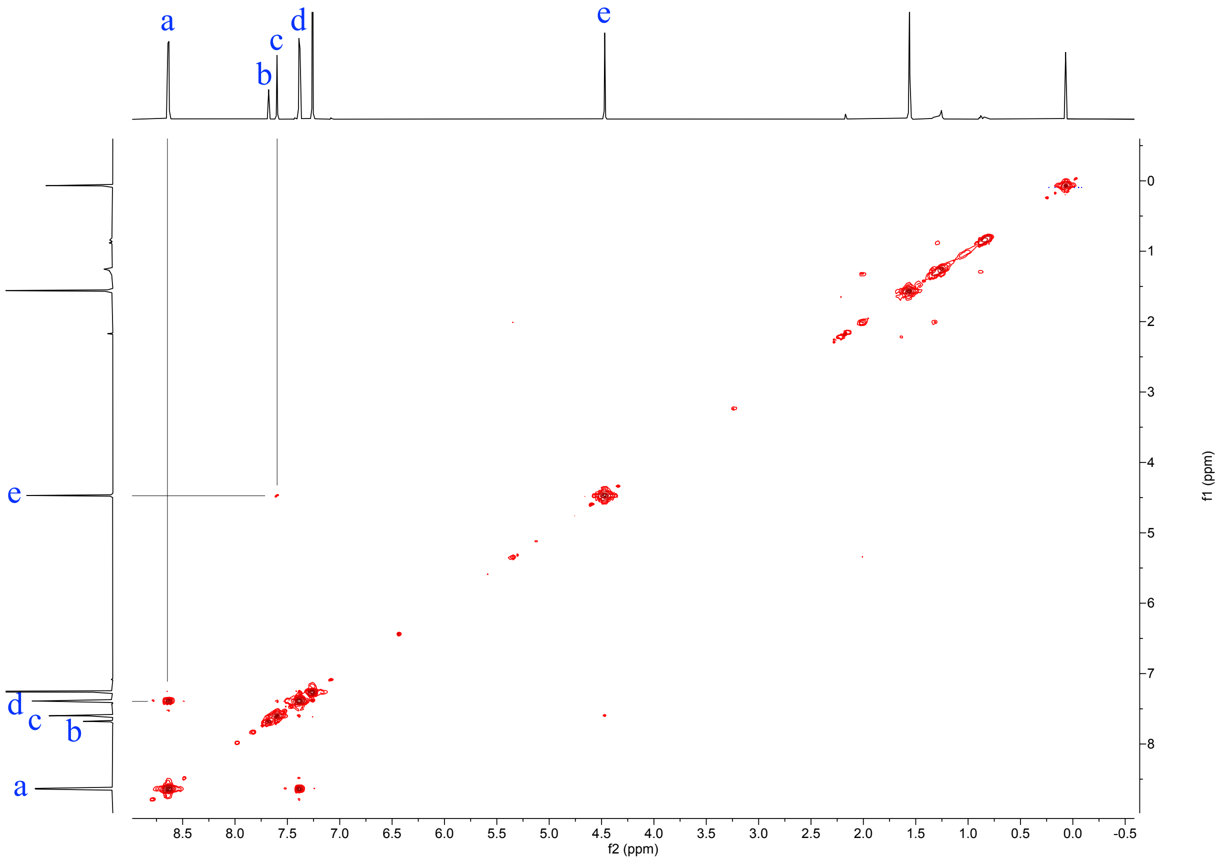

Figure S205: ^{1}H-^{1}H COSY spectrum (600 MHz, CD_3CN, 298 K) of **6**.

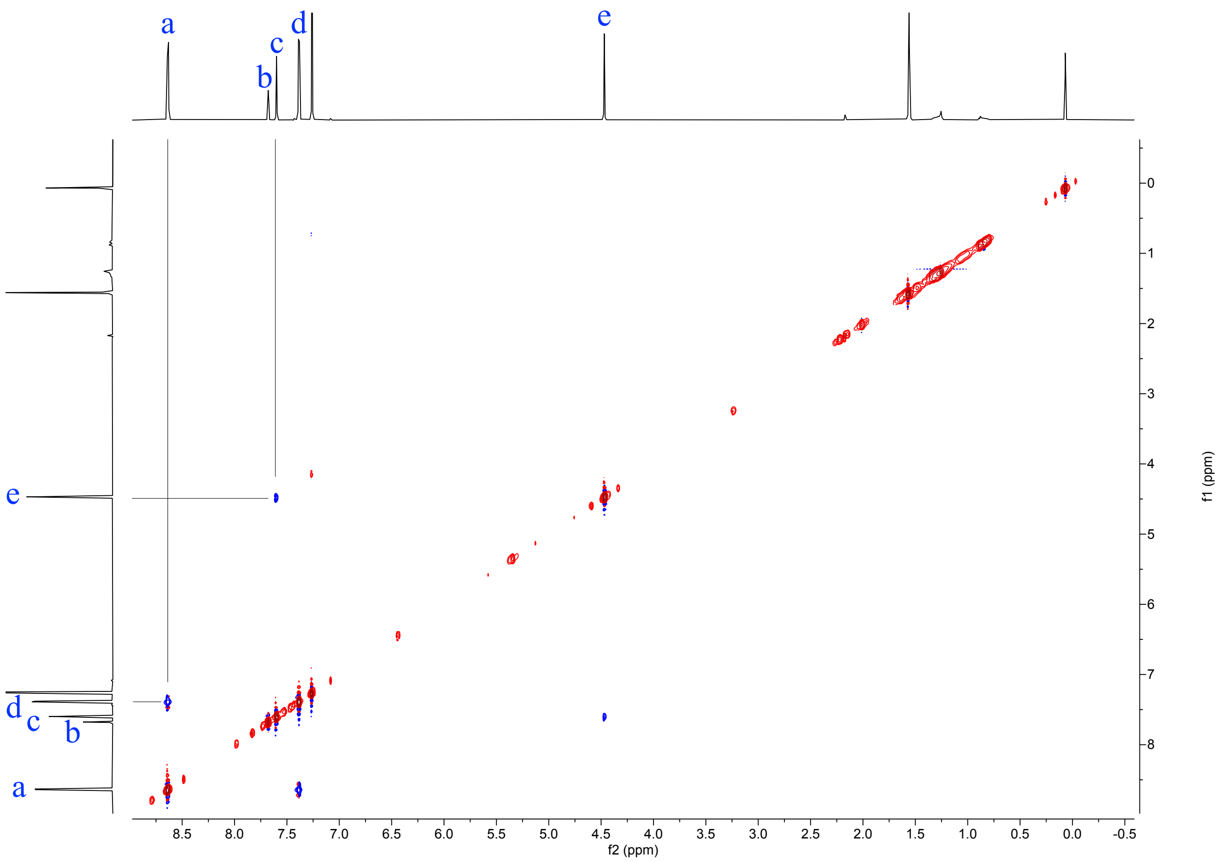

Figure S206: ^{1}H-^{1}H NOESY spectrum (600 MHz, CD_3CN, 298 K) of **6**.

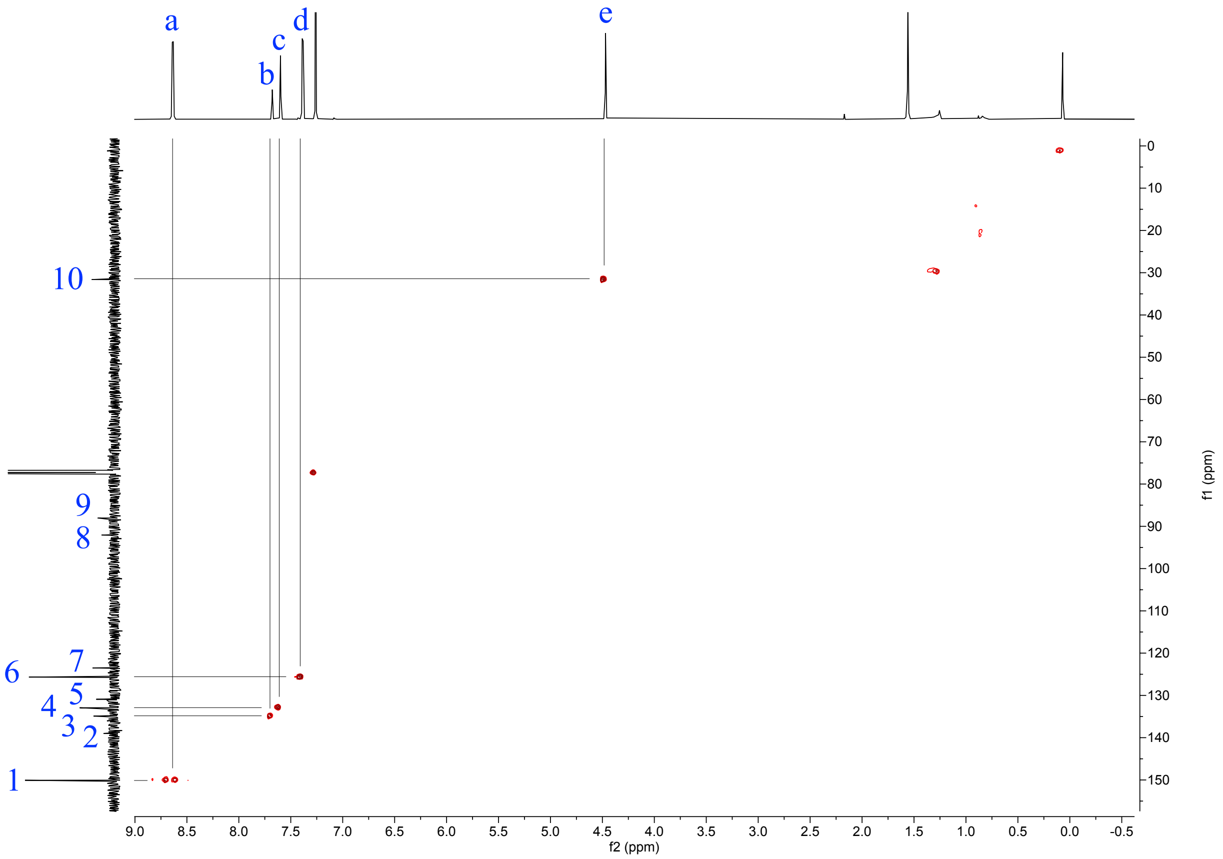

Figure S207: ^{1}H-^{13}C HSQC spectrum (600 MHz, CD_3CN, 298 K) of **6**.

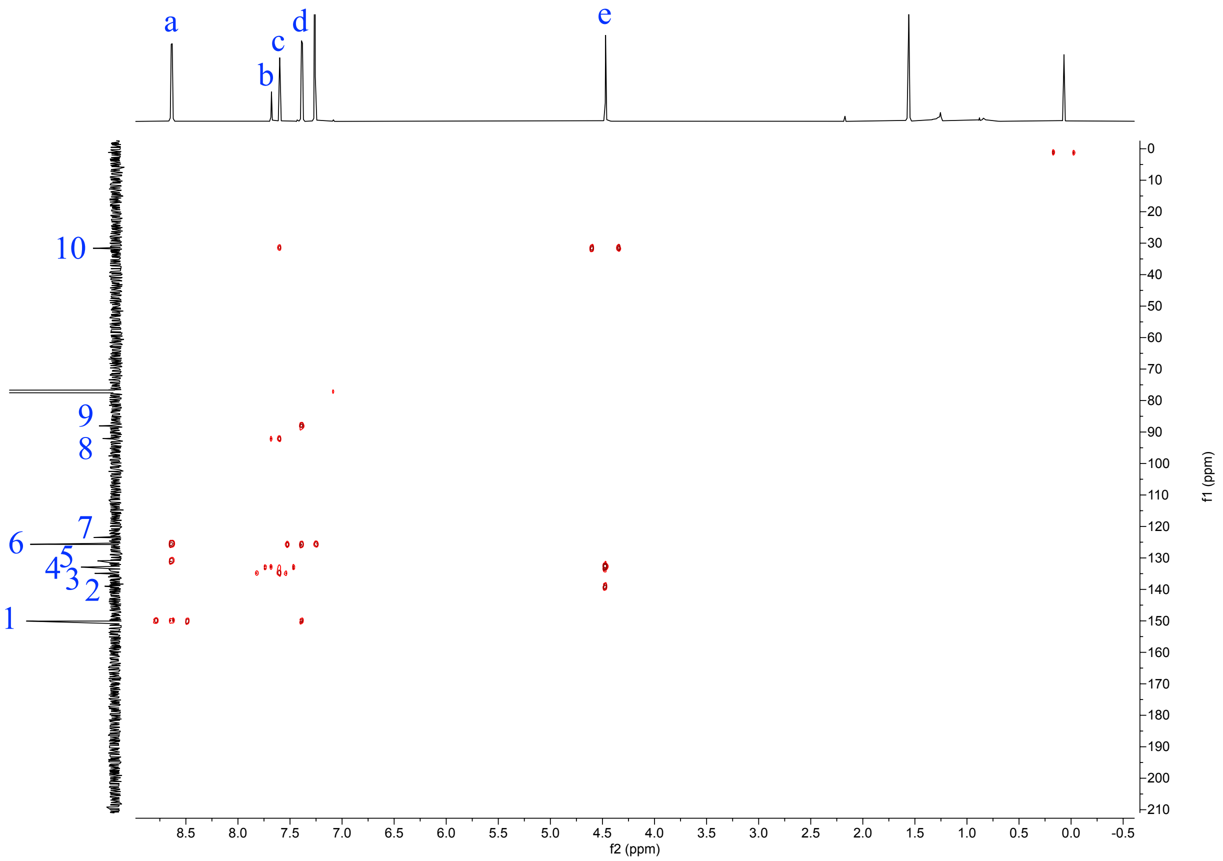

Figure S208: ^{1}H-^{13}C HMBC spectrum (600 MHz, CD_3CN, 298 K) of **6**.

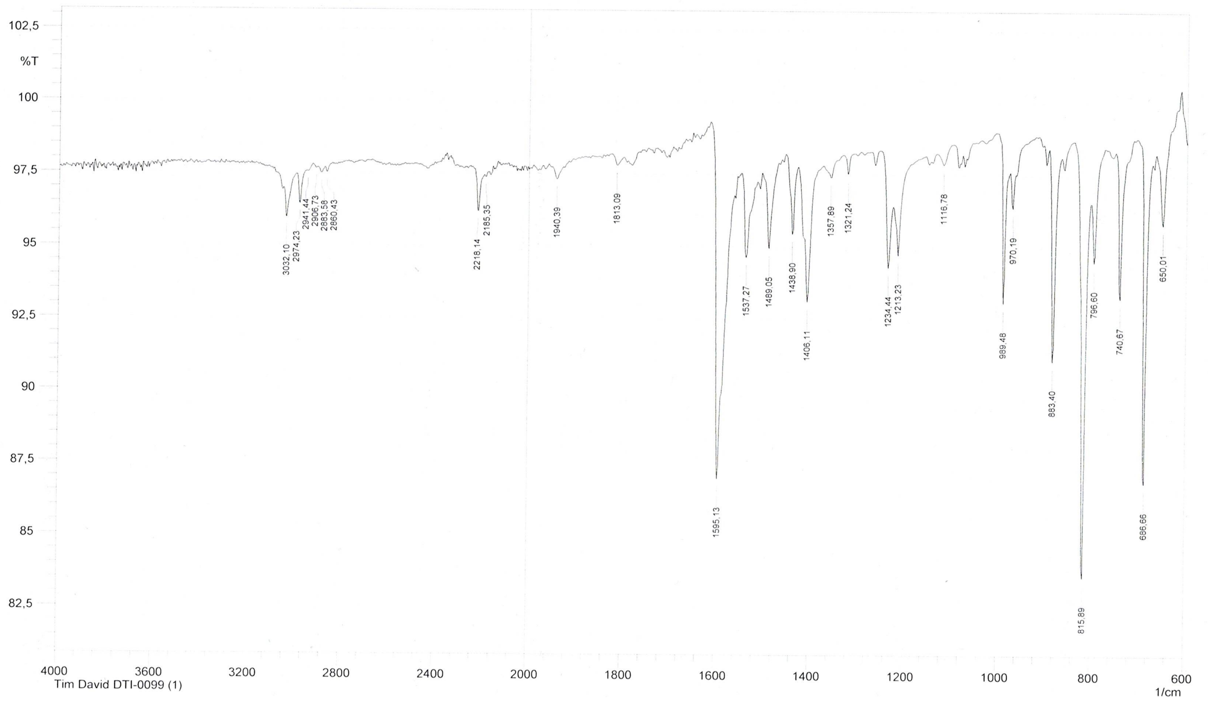

Figure S209: IR spectrum of **6**.

Acquisition Parameter

Source Type	ESI	Ion Polarity	Positive	Set Nebulizer	0.3 Bar
Focus	Not active	Set Capillary	4000 V	Set Dry Heater	180 °C
Scan Begin	50 m/z	Set End Plate Offset	-500 V	Set Dry Gas	4.0 l/min
Scan End	1500 m/z	Set Collision Cell RF	600.0 Vpp	Set Divert Valve	Source

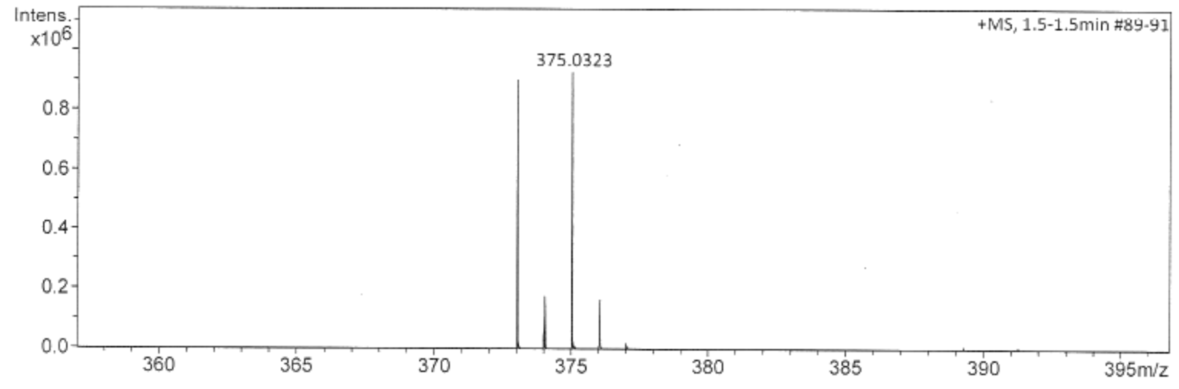

Meas. m/z	#	Ion Formula	m/z	err [ppm]	mSigma	# mSigma	Score	rdb	e⁻ Conf	N-Rule
373.0342	1	C21H14BrN2	373.0335	-1.8	33.5	1	100.00	15.5	even	ok

Figure S210: ESI (HRMS) spectrum of **6**.

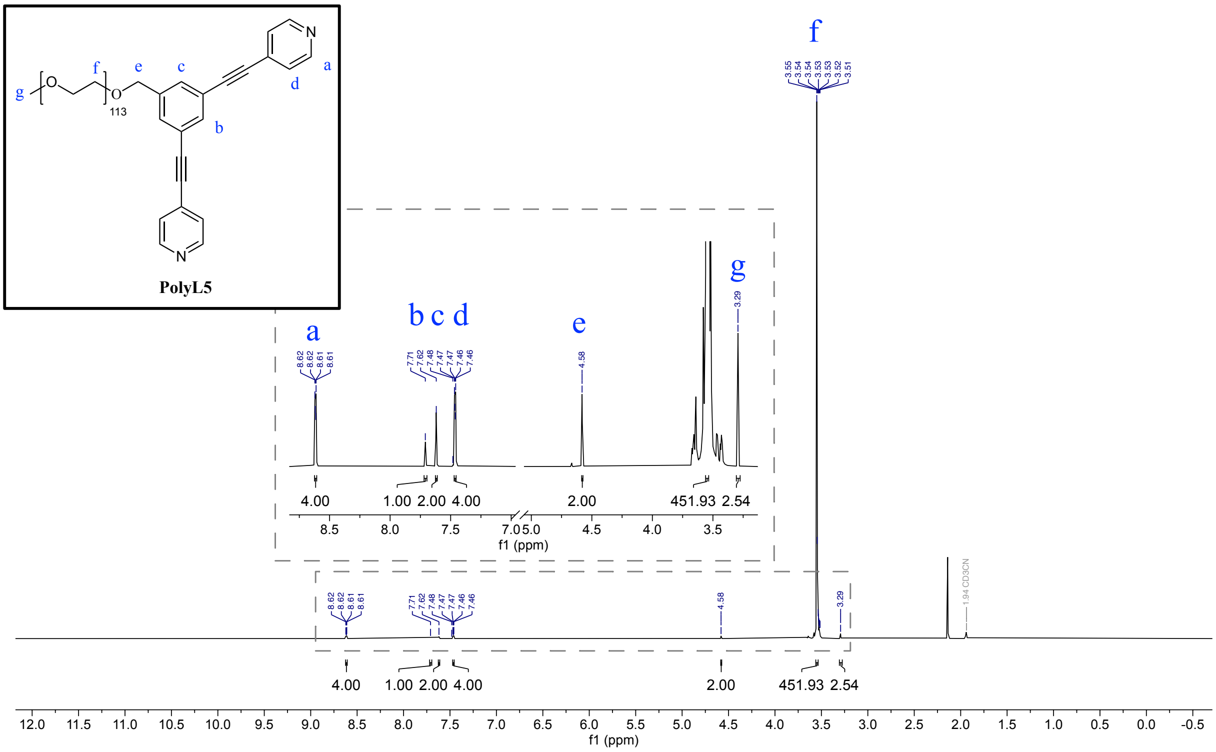

Figure S211: ^{1}H NMR spectrum (600 MHz, CD_3CN, 298 K) of **PolyL5**.

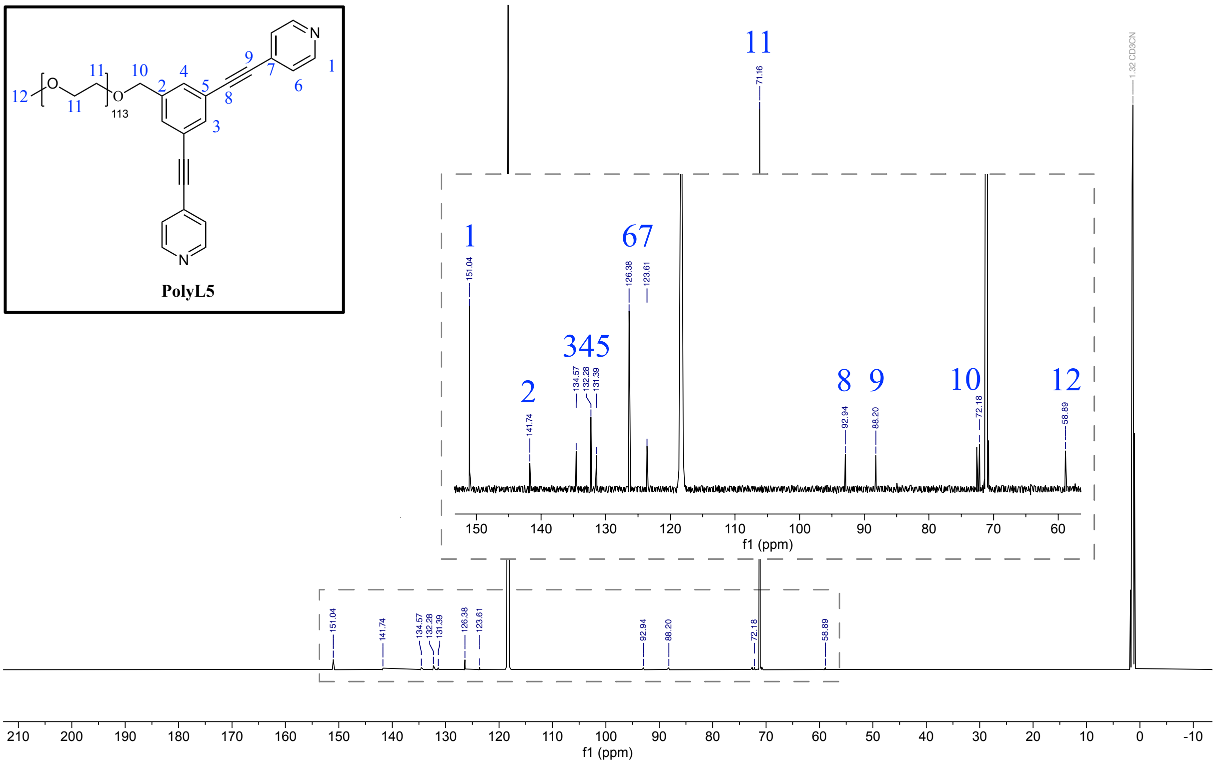

Figure S212: $^{13}C\{^{1}H\}$ NMR spectrum (151 MHz, CD_3CN, 298 K) of **PolyL5**.

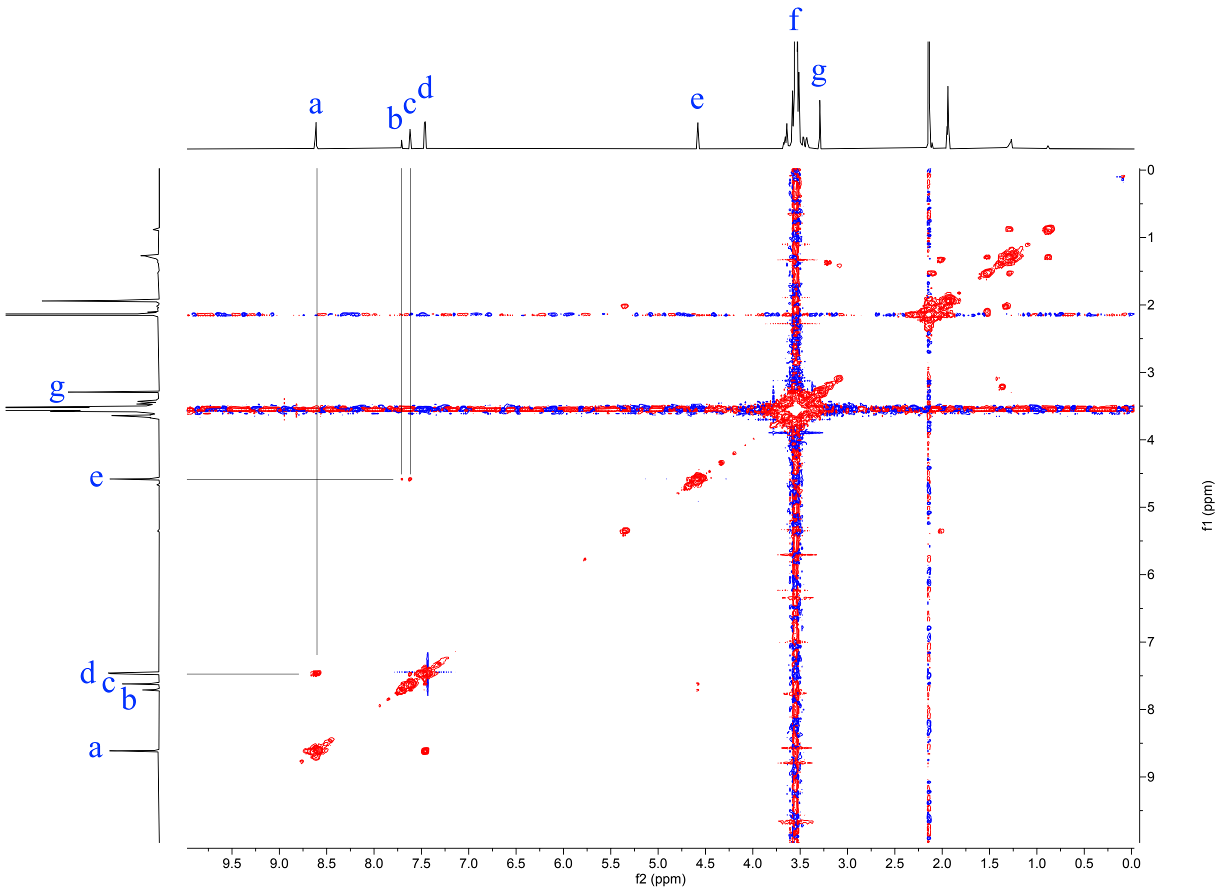

Figure S213: ^{1}H-^{1}H COSY spectrum (600 MHz, CD_3CN, 298 K) of **PolyL5**.

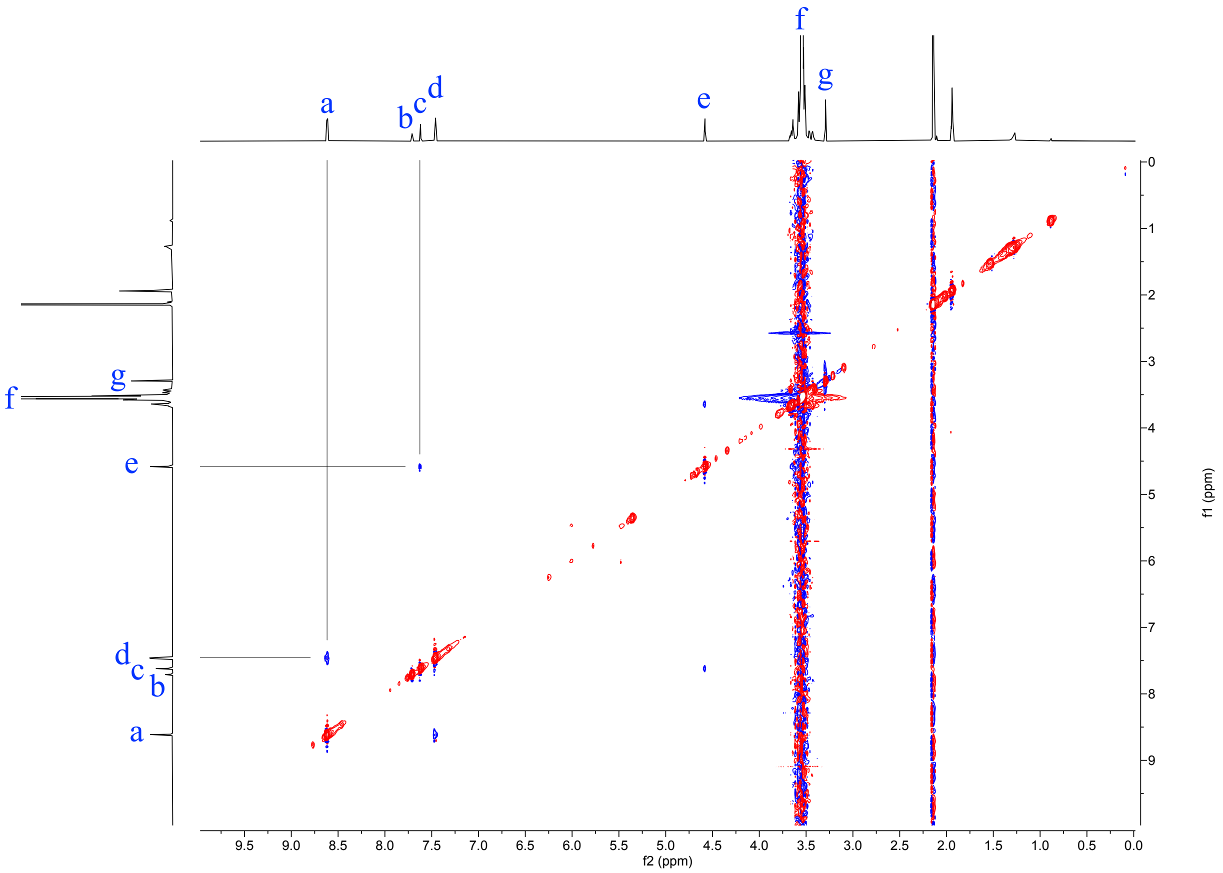

Figure S214: ^{1}H-^{1}H NOESY spectrum (600 MHz, CD_3CN, 298 K) of **PolyL5**.

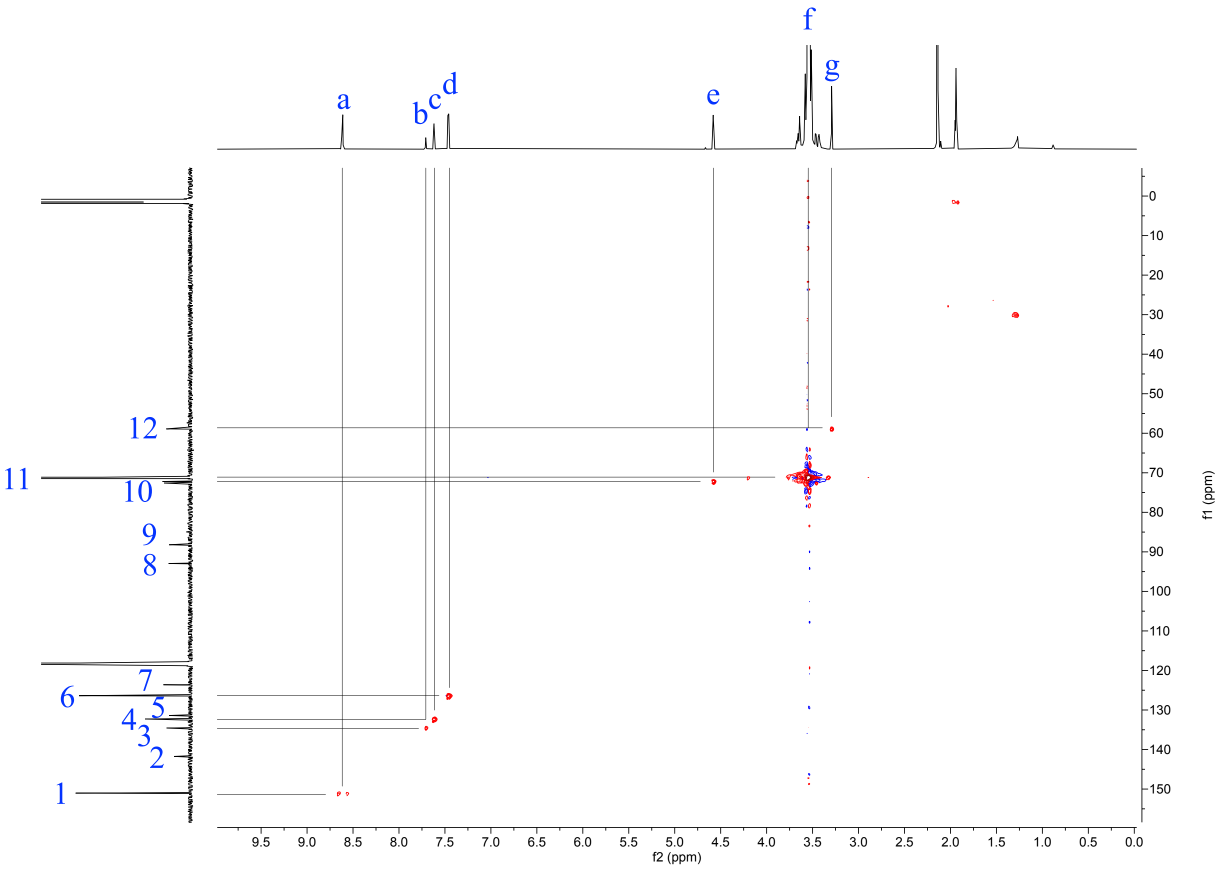

Figure S215: $^{1}H-^{13}C$ HSQC spectrum (600 MHz, CD_3CN, 298 K) of **PolyL5**.

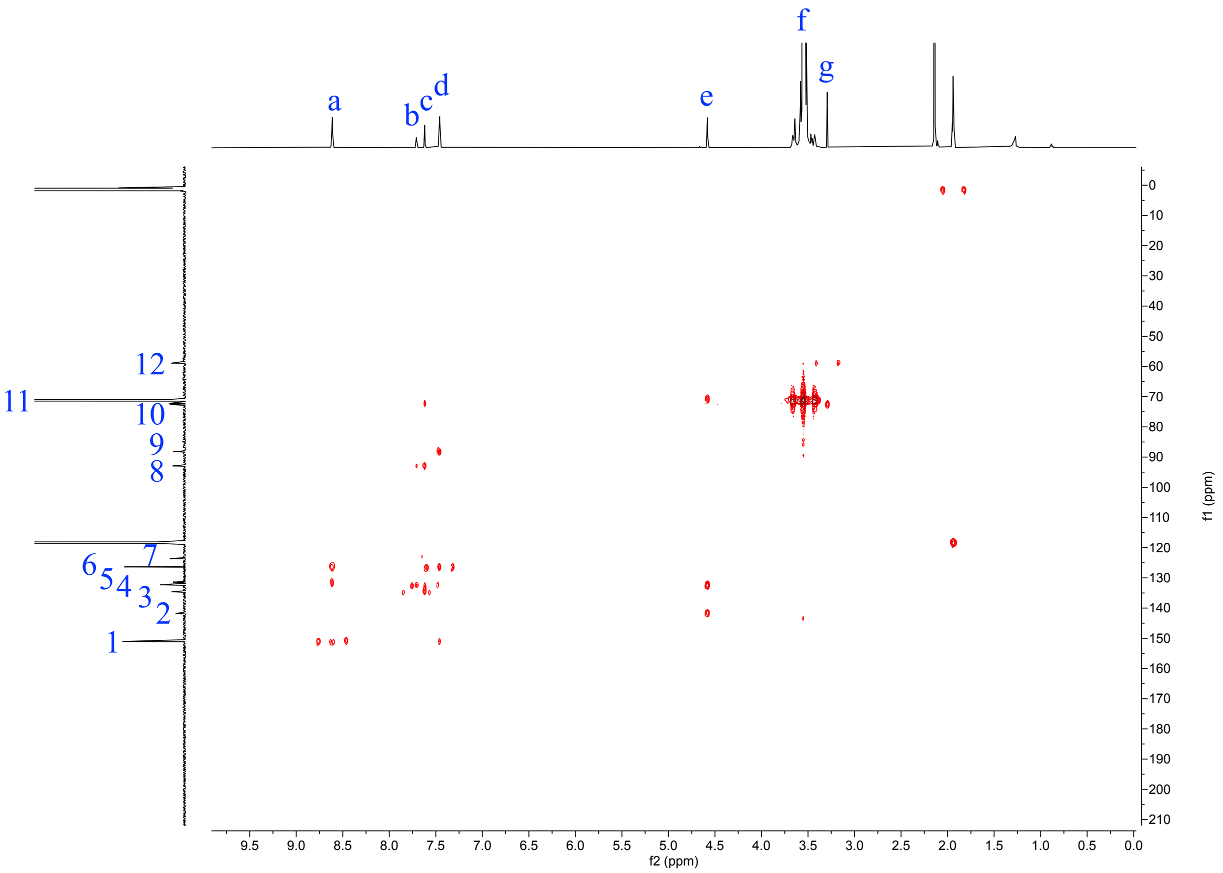

Figure S216: $^{1}H-^{13}C$ HMBC spectrum (600 MHz, CD_3CN, 298 K) of **PolyL5**.

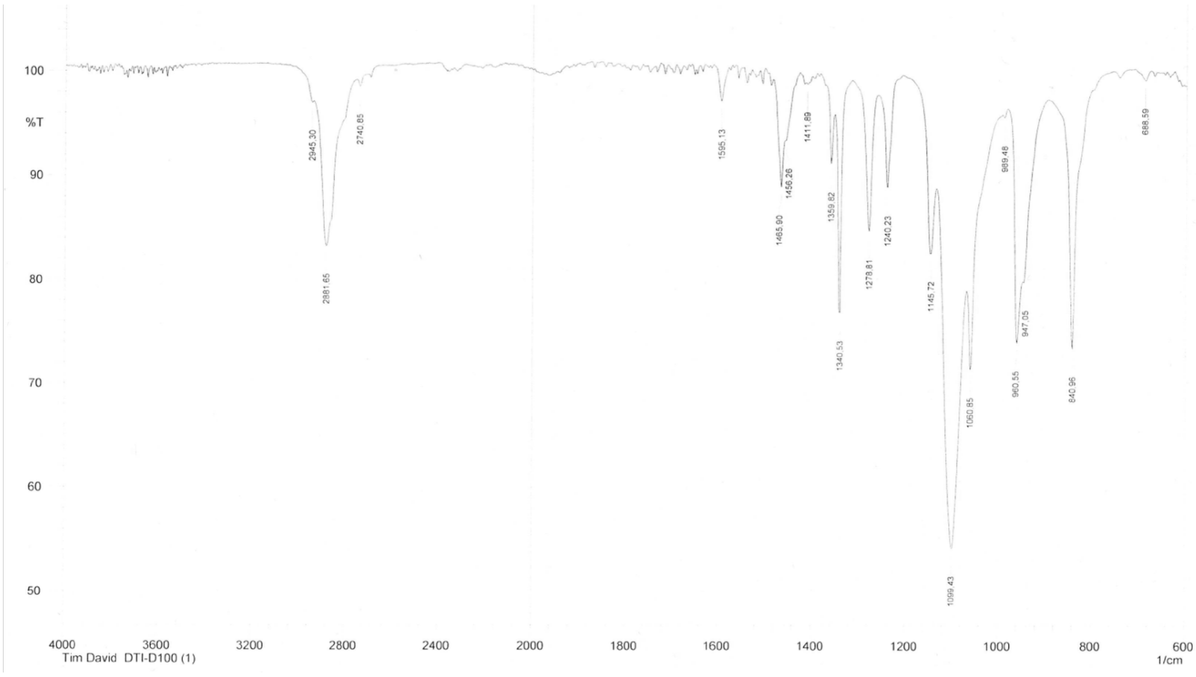

Figure S217: IR spectrum of **PolyL5**.

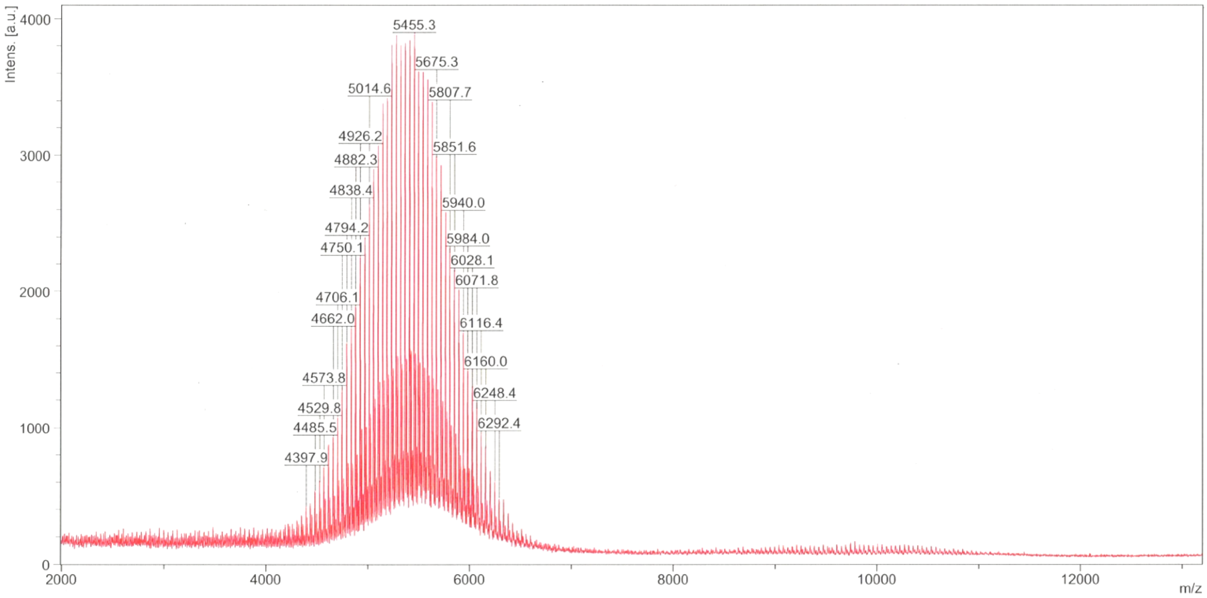

Figure S218: MALDI-MS spectrum of **PolyL5**.

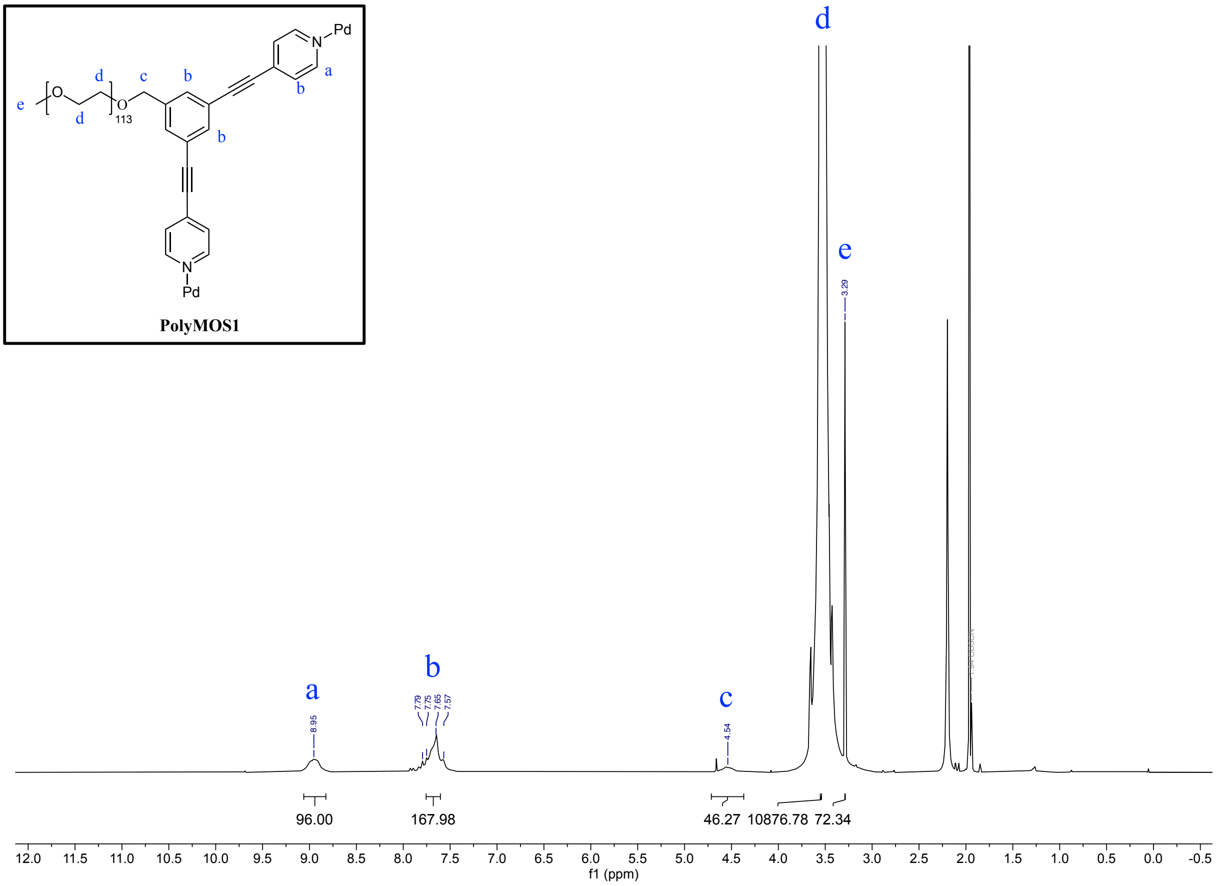

Figure S219: ^{1}H NMR spectrum (600 MHz, CD_3CN, 298 K) of **PolyMOS1**.

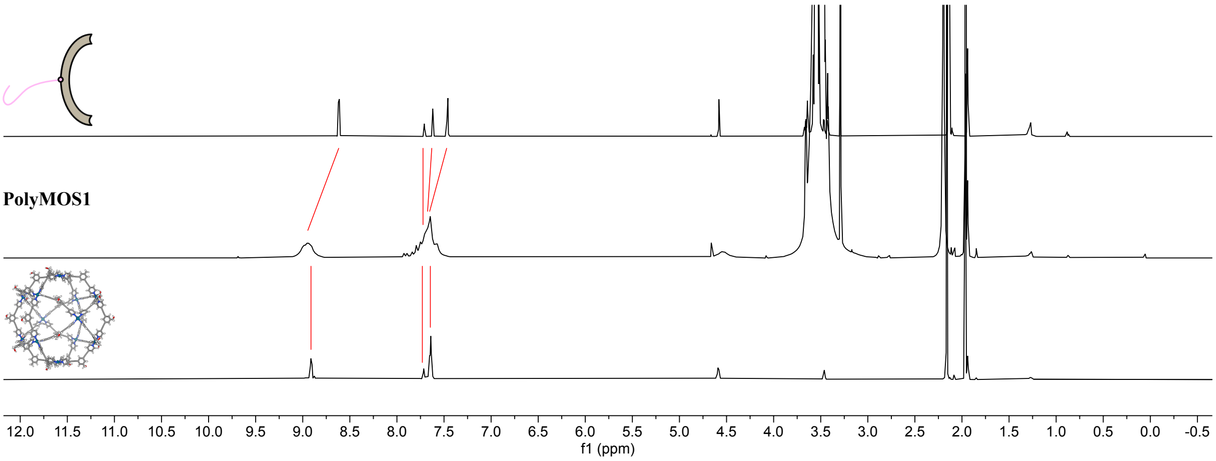

Figure S220: Stacked ^{1}H NMR spectra (600 MHz, CD_3CN, 298 K) spectra of **PolyL4** (top), **PolyMOS1** (middle), and **MOS1** (bottom).

Cisplatin encapsulations

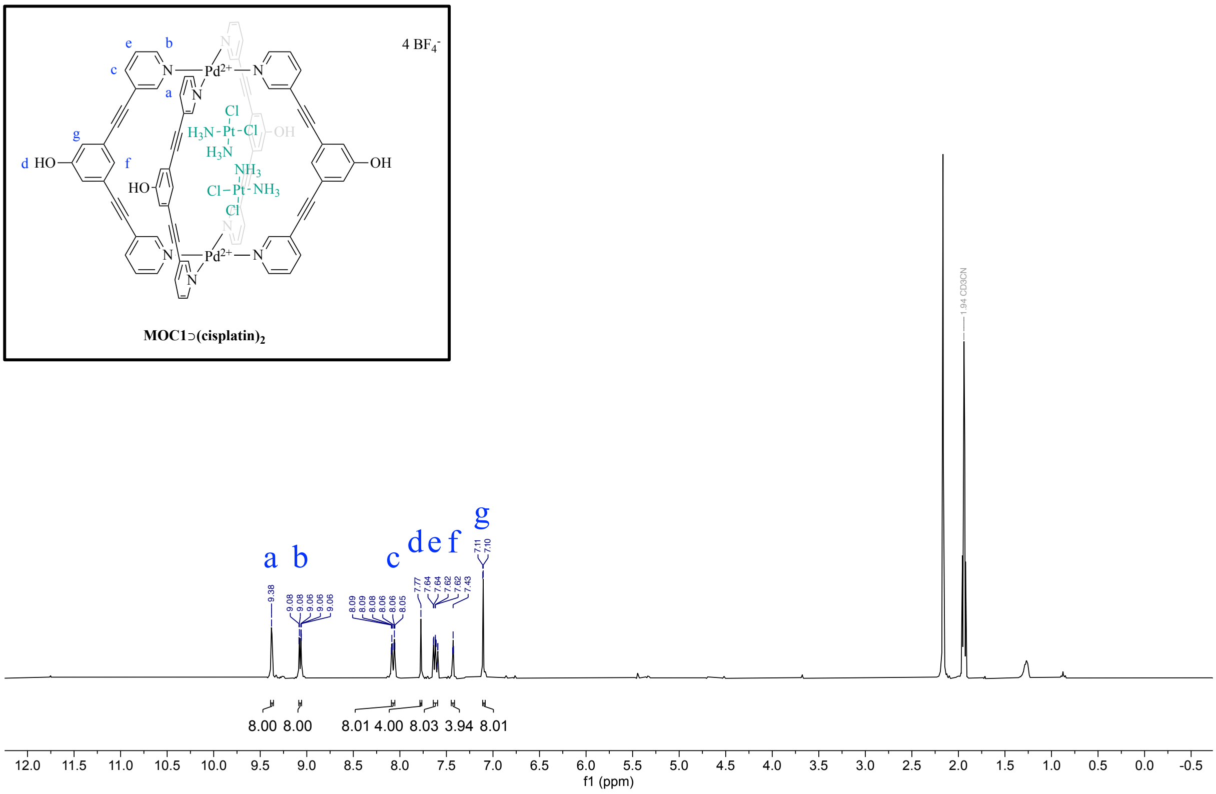

Figure S221: ^{1}H NMR spectrum (300 MHz, CD_3CN, 298 K) of **MOC1⊃(cisplatin)₂**.

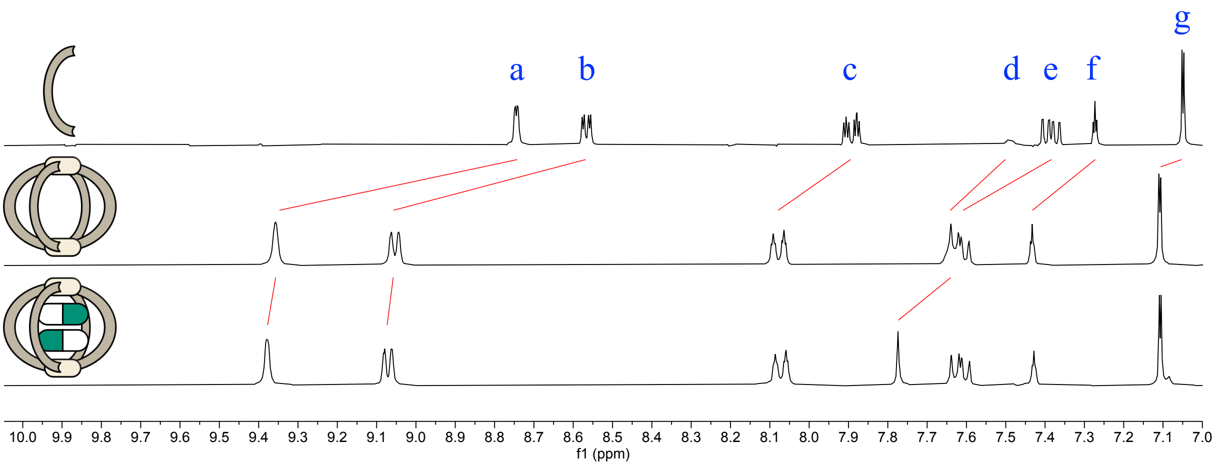

Figure S222: Stacked ^{1}H NMR spectra (300 MHz, CD_3CN, 298 K) spectra of **L1** (top), **MOC1** (middle), and **MOC1⊃(cisplatin)₂** (bottom).

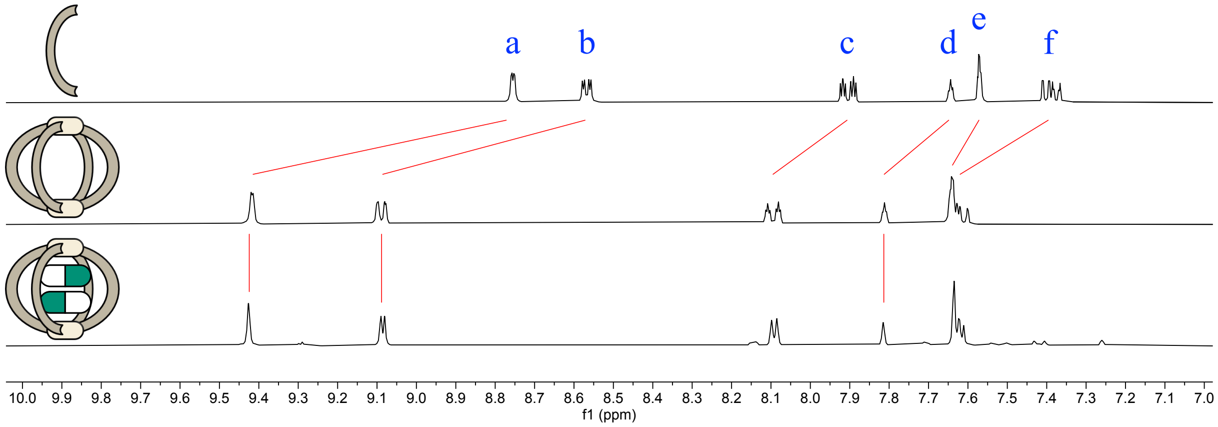

Figure S223: Stacked ^{1}H NMR spectra (600 MHz, CD_3CN, 298 K) spectra of **L2** (top), **MOC2** (middle), and **MOC2** after the addition of 5.00 equivalents of cisplatin (bottom).

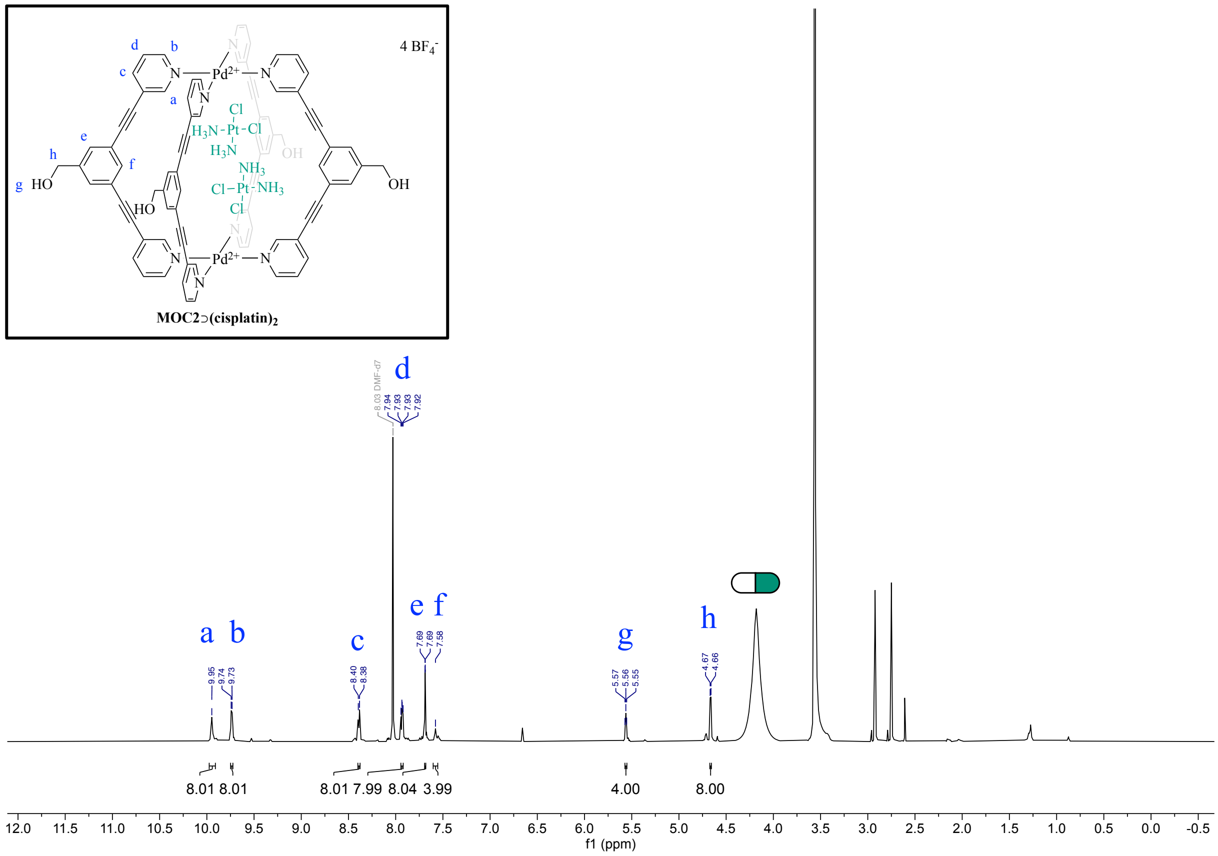

Figure S224: ^{1}H NMR spectrum (600 MHz, DMF-d7, 298 K) of **MOC2⊃(cisplatin)$_2$**.

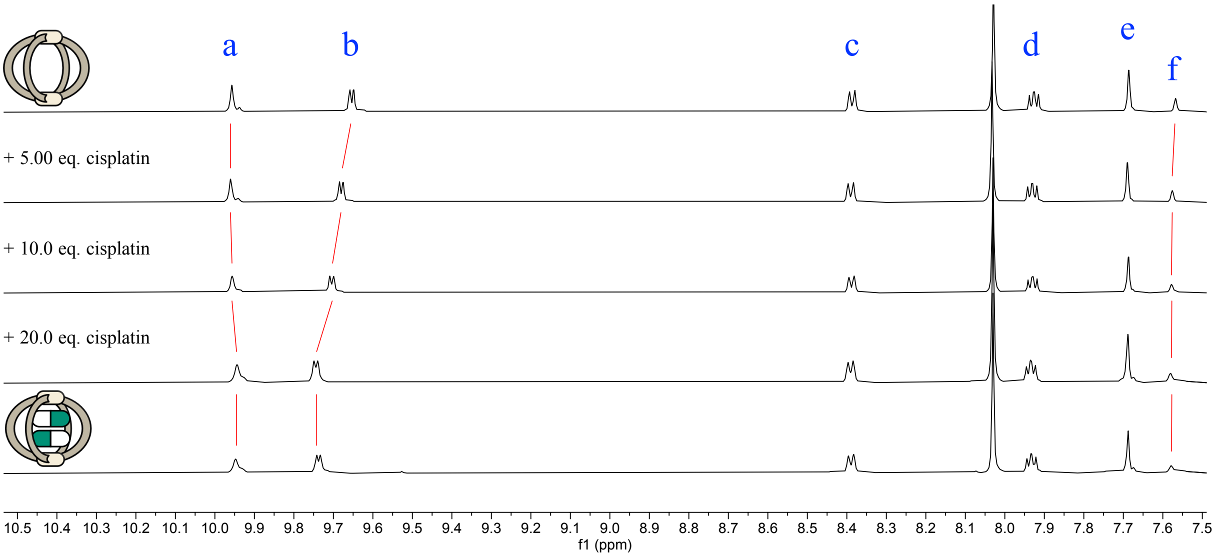

Figure S225: Stacked ^{1}H NMR spectra (600 MHz, DMF-d7, 298 K) spectra of **MOC2** (top), **MOC2** after the addition of 5.00 eq., 10.0 eq., 20.0 eq. cisplatin (middle), **MOC2⊃(cisplatin)₂** (bottom).

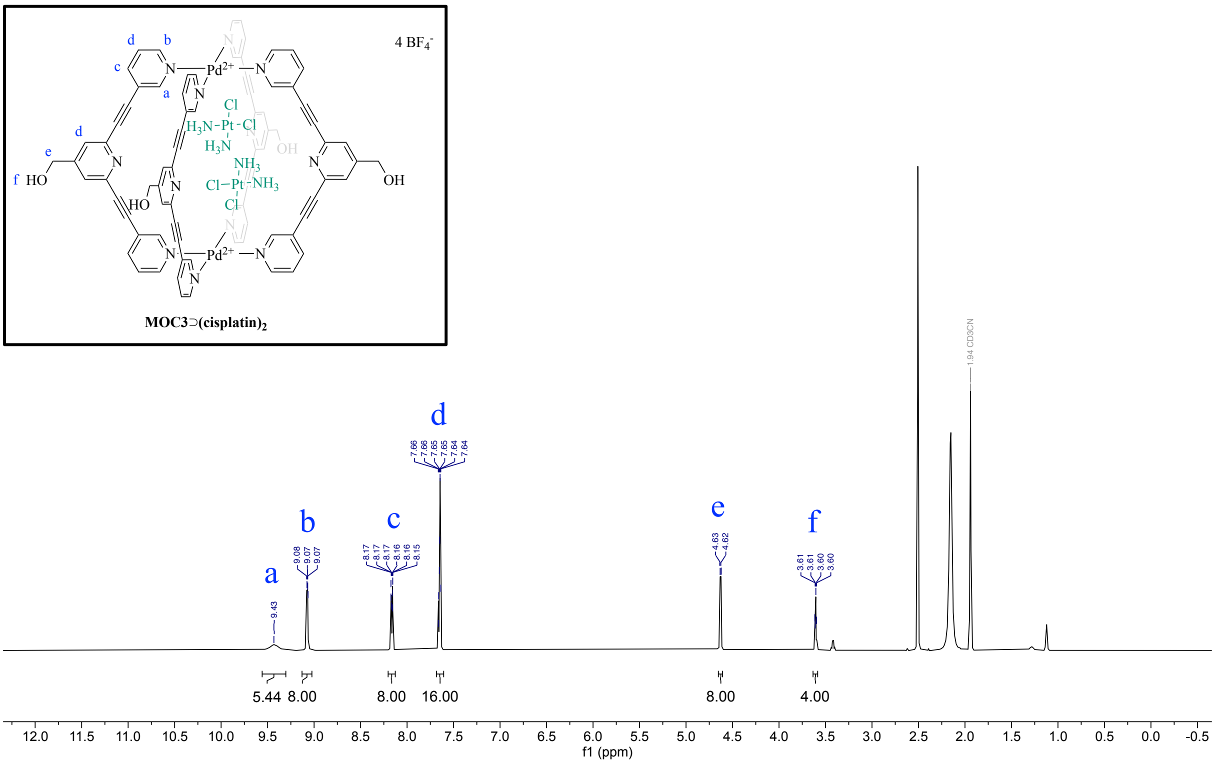

Figure S226: ^{1}H NMR spectrum (600 MHz, CD_3CN, 298 K) of **MOC3⊃(cisplatin)₂**.

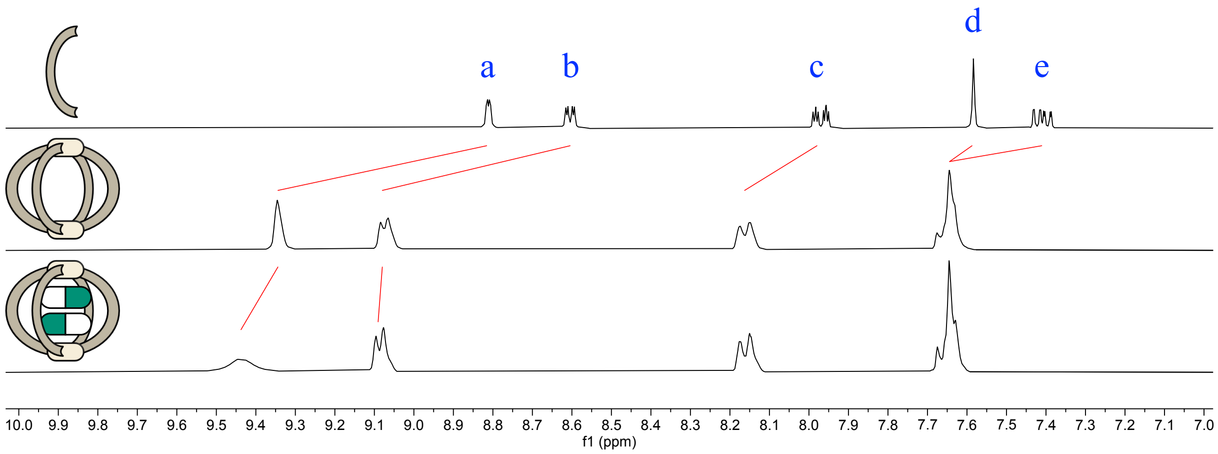

Figure S227: Stacked ^{1}H NMR spectra (600 MHz, CD_3CN, 298 K) spectra of **L3** (top), **MOC3** (middle), and **MOC3⊃(cisplatin)₂** (bottom).

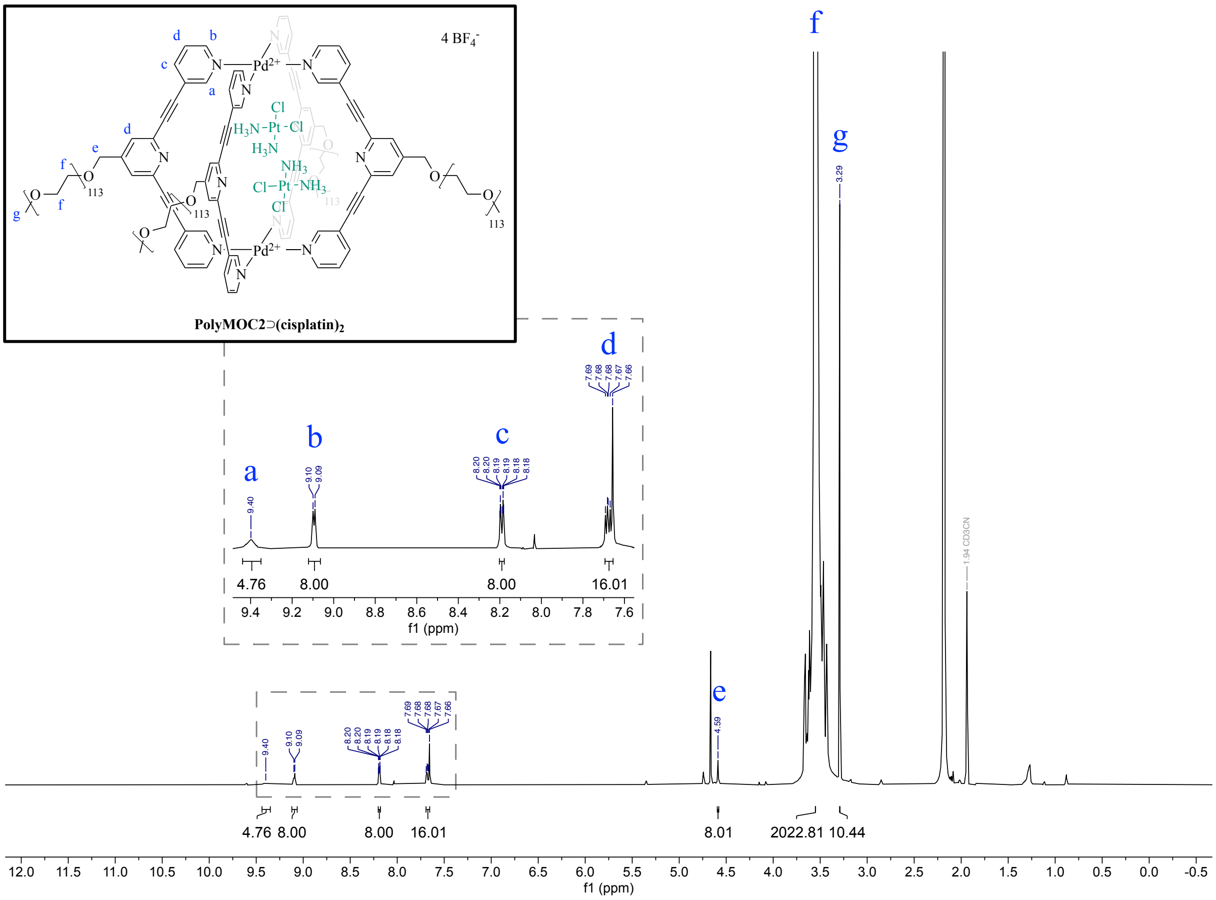

Figure S228: ^{1}H NMR spectrum (600 MHz, CD_3CN, 298 K) of **PolyMOC2⊃(cisplatin)₂**.

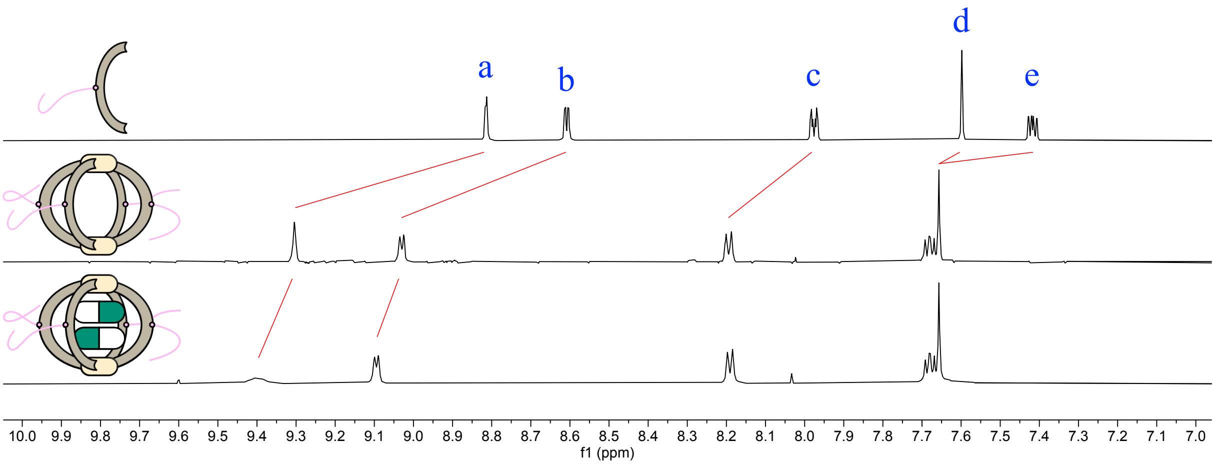

Figure S229: Stacked ^{1}H NMR spectra (600 MHz, CD_3CN, 298 K) spectra of **PolyL2** (top), **PolyMOC2** (middle), and **PolyMOC2⊃(cisplatin)₂** (bottom).

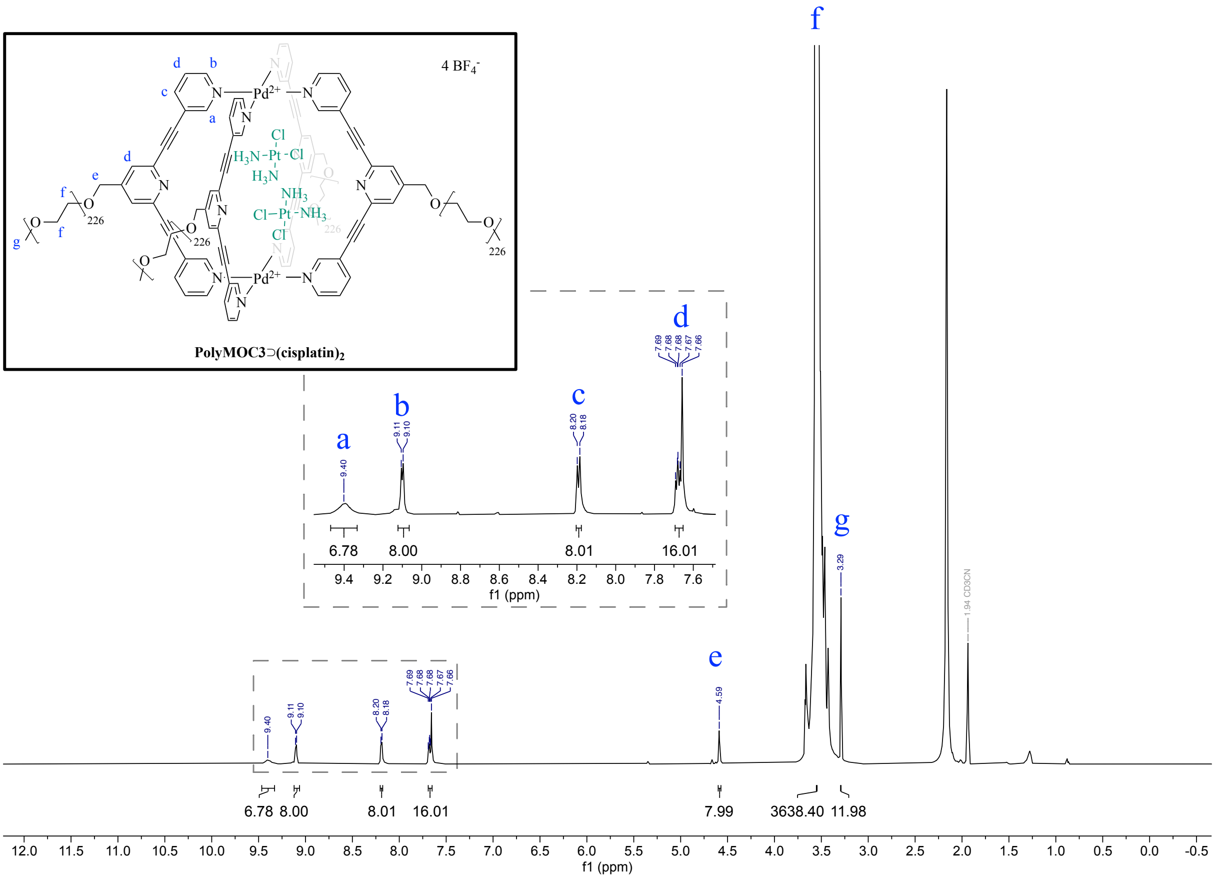

Figure S230: ^{1}H NMR spectrum (600 MHz, CD_3CN, 298 K) of **PolyMOC3⊃(cisplatin)₂**.

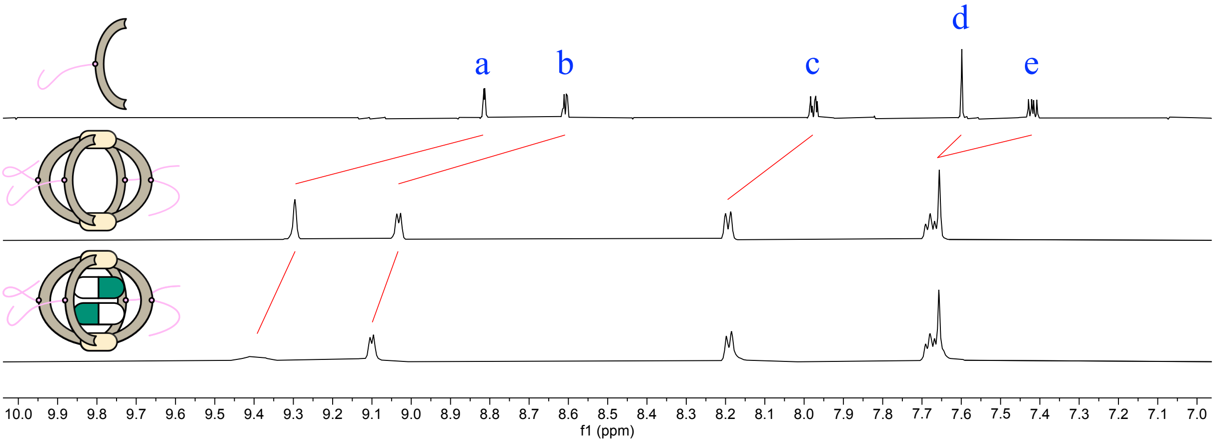

Figure S231: Stacked ^{1}H NMR spectra (600 MHz, CD_3CN, 298 K) spectra of **PolyL3** (top), **PolyMOC3** (middle), and **PolyMOC3⊃(cisplatin)₂** (bottom).

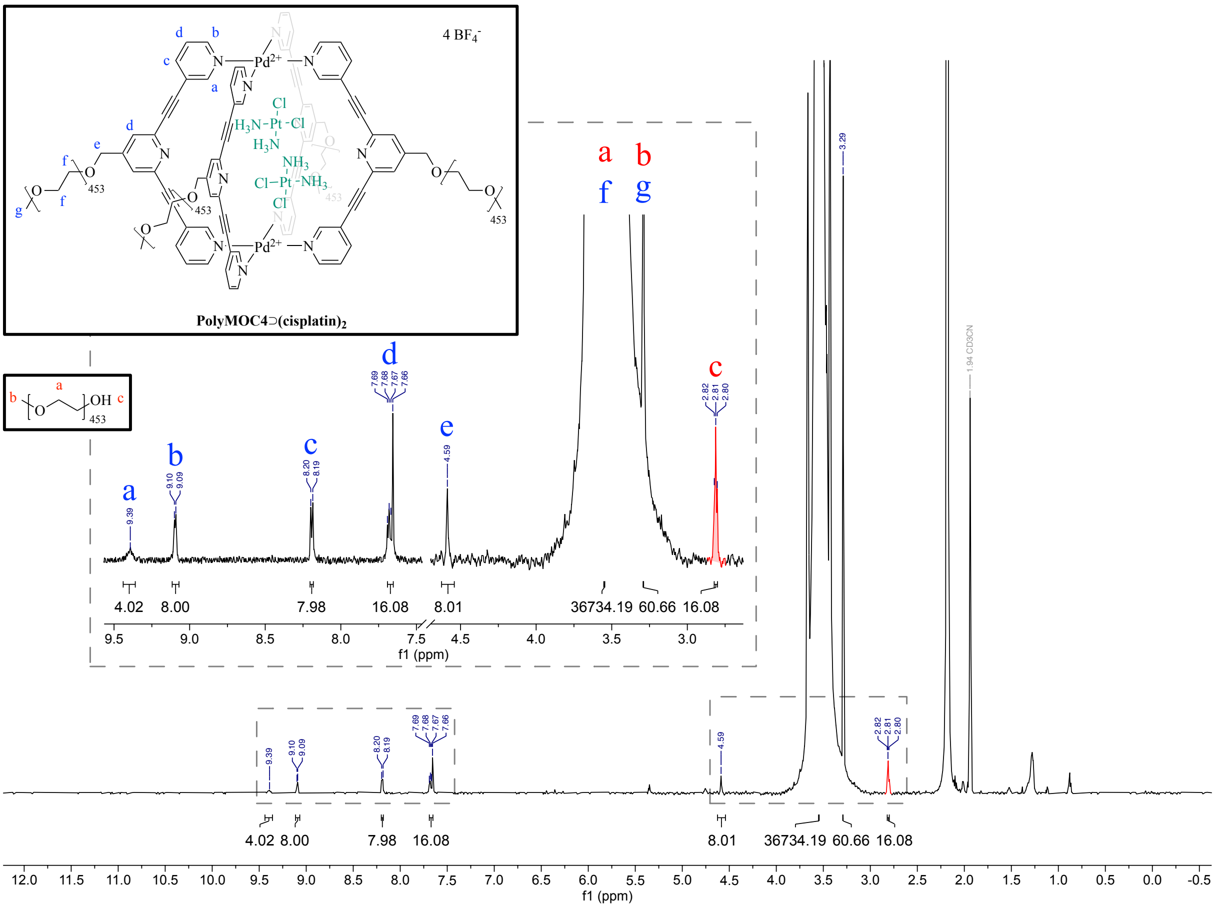

Figure S232: ^{1}H NMR spectrum (600 MHz, CD_3CN, 298 K) of **PolyMOC4⊃(cisplatin)₂**.

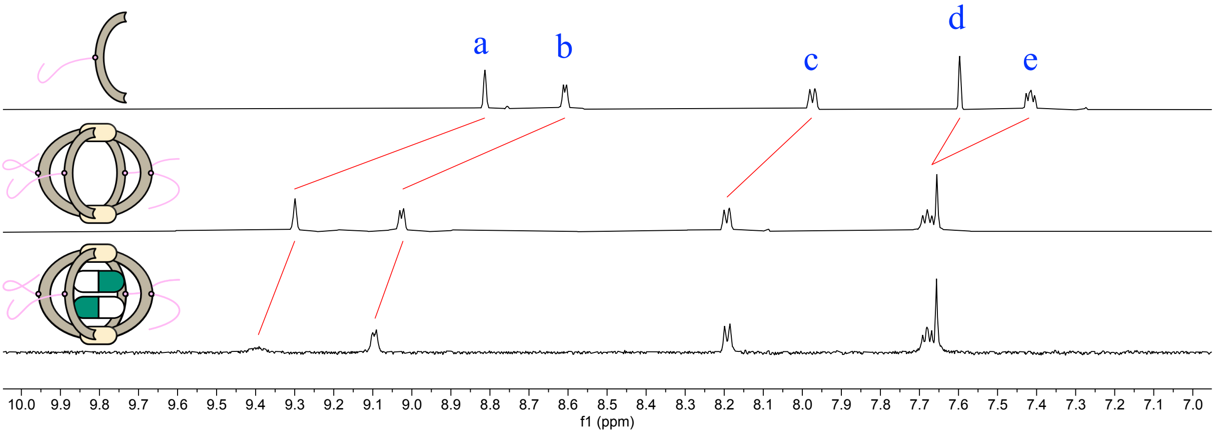

Figure S233: Stacked ^{1}H NMR spectra (600 MHz, CD_3CN, 298 K) spectra of **PolyL4** (top), **PolyMOC4** (middle), and **PolyMOC4⊃(cisplatin)$_2$** (bottom).

DOSY experiments

DOSY NMR experiments were recorded at 298 K. The hydrodynamic radii were estimated using the unmodified Stokes-Einstein equation. This equation was solved for r_H using values for η (η_{CD3CN} = 0.343 mPa s).

$$D = \frac{k_B T}{6\pi\eta r_H} \quad \text{(S19)}$$

D is the measured diffusion coefficient ($m^2\ s^{-1}$)

k_B is the Boltzmann constant ($1.3806485 \times 10^{-23}\ m^2\ kg\ s^{-2}\ K^{-1}$)

T is the temperature (K)

r_H is the hydrodynamic radius of the analyte (m)

η is the viscosity of deuterated acetonitrile at 25 °C (0.343 mPa s)

The hydrodynamic radii were estimated to be r_H = 5.09 Å for **L4** and r_H = 25.5 Å for **MOS1**.

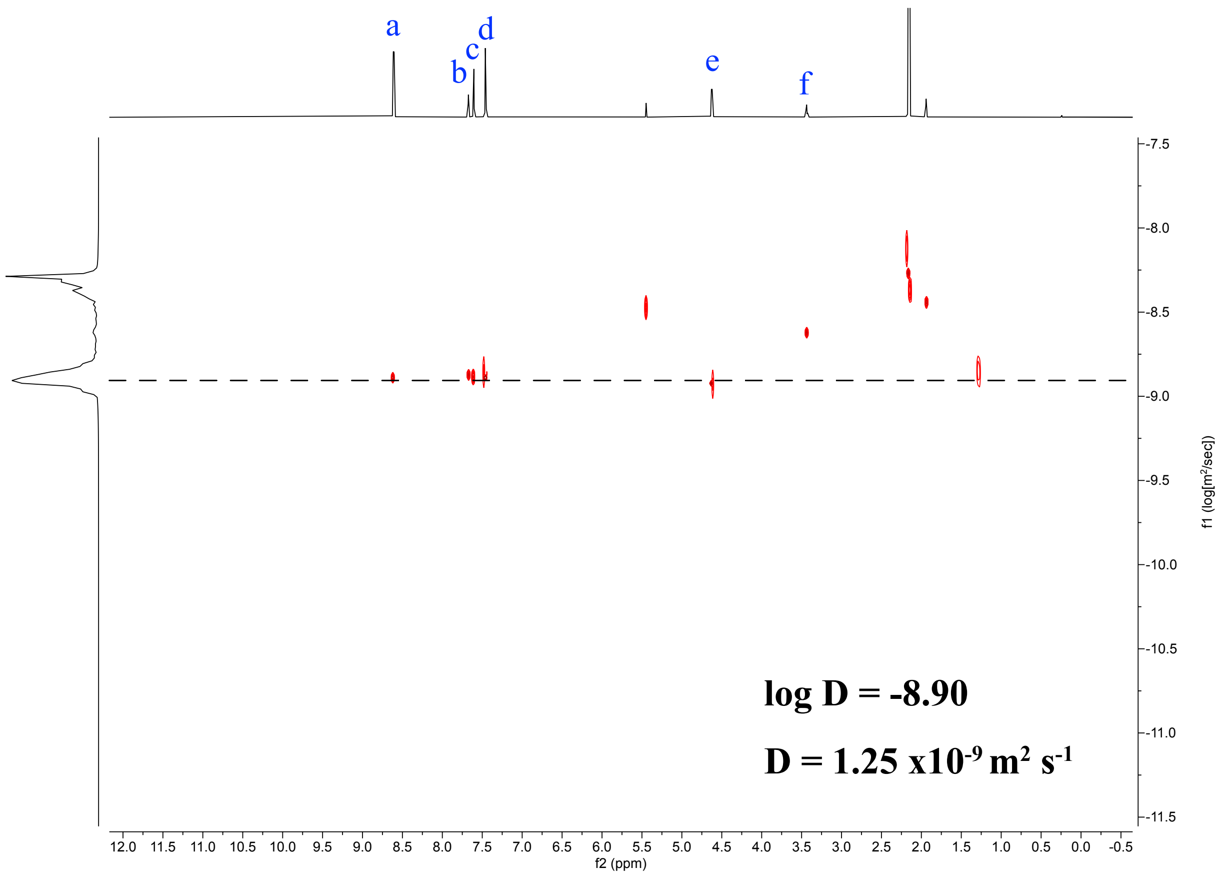

Figure S234: ^{1}H DOSY NMR (600 MHz, CD_3CN, 298 K) spectra of **L4.**

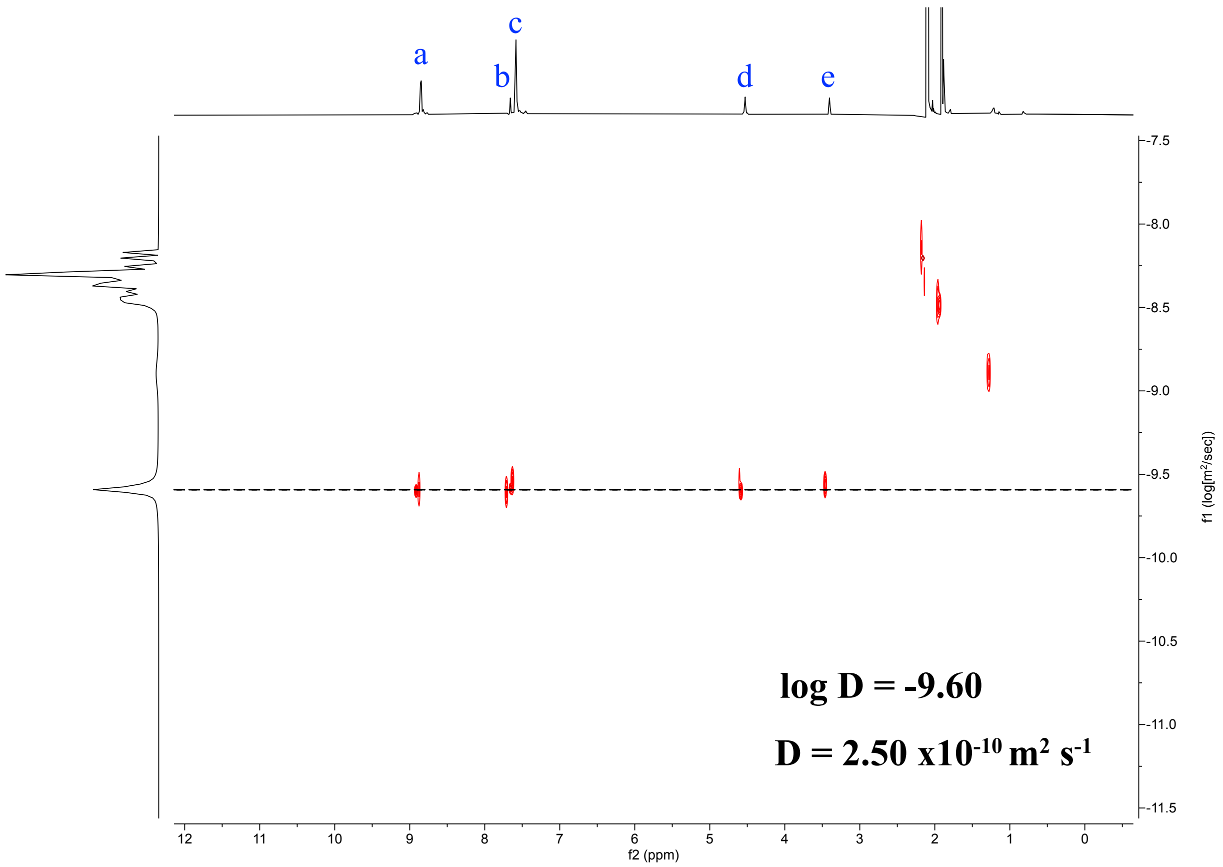

Figure S235: ^{1}H DOSY NMR (600 MHz, CD_3CN, 298 K) spectra of **MOS1.**

XIV. References

1 Kim, T. Y., Digal, L., Gardiner, M. G., Lucas, N. T. & Crowley, J. D. Octahedral [Pd6L8]12+ Metallosupramolecular Cages: Synthesis, Structures and Guest-Encapsulation Studies. *Chem. Eur. J.* **23**, 15089-15097 (2017).

2 Bartucci, M. A. & Ciszek, J. W. Substituent Parameters Impacting Isomer Composition and Optical Properties of Dihydroindolizine Molecular Switches. *J. Org. Chem.* **79**, 5586-5594 (2014).

3 Schmidt, A. *et al.* Evaluation of New Palladium Cages as Potential Delivery Systems for the Anticancer Drug Cisplatin. *Chem. Eur. J.* **22**, 2253-2256 (2016).

4 El Bakkari, M. & Vincent, J.-M. Fluorous Phase-Switching of Pyridyl-Tagged Substrates/Products. *Org. Lett.* **6**, 2765-2767 (2004).

5 Ngo, K. *et al.* Protein-Induced Change in Ligand Protonation during Trypsin and Thrombin Binding: Hint on Differences in Selectivity Determinants of Both Proteins? *J. Med. Chem.* **63**, 3274-3289 (2020).

6 Preston, D., McNeill, S. M., Lewis, J. E. M., Giles, G. I. & Crowley, J. D. Enhanced kinetic stability of [Pd2L4]4+ cages through ligand substitution. *Dalton Trans.* **45**, 8050-8060 (2016).

7 Lewis, J. E. M., John McAdam, C., Gardiner, M. G. & Crowley, J. D. A facile "click" approach to functionalised metallosupramolecular architectures. *Chem. Commun.* **49**, 3398-3400 (2013).

8 Watkins, D. L. & Fujiwara, T. Synthesis, characterization, and solvent-independent photochromism of spironaphthooxazine dimers. *J. Photochem. Photobiol. A: Chem.* **228**, 51-59 (2012).

9 Brown, C. M. *et al.* Endohedrally Functionalized Metal–Organic Cage-Cross-Linked Polymer Gels as Modular Heterogeneous Catalysts. *J. Am. Chem. Soc.* **144**, 13276-13284 (2022).

10 Lewis, J. E. M., Gavey, E. L., Cameron, S. A. & Crowley, J. D. Stimuli-responsive Pd2L4 metallosupramolecular cages: towards targeted cisplatin drug delivery. *Chem. Sci.* **3**, 778-784 (2012).

11 Nain, A. K. Densities and volumetric properties of (acetonitrile+an amide) binary mixtures at temperatures between 293.15K and 318.15K. *J. Chem. Thermodyn.* **38**, 1362-1370 (2006).

12 Bigg, P. H. Density of water in SI units over the range 0-40°C. *Br. J. Appl. Phys.* **18**, 521 (1967).

13 Niepmann, R. Thermodynamic properties of acetonitrile 1. Speeds of sound between 240 and 475 K and up to 60 MPa. *J. Chem. Thermodyn.* **16**, 779-785 (1984).

14 Benedetto, G. *et al.* Speed of Sound in Pure Water at Temperatures between 274 and 394 K and at Pressures up to 90 MPa. *Int. J. Thermophys.* **26**, 1667-1680 (2005).

15 Becke, A. D. Density-functional exchange-energy approximation with correct asymptotic behavior. *Phys. Rev. A* **38**, 3098-3100 (1988).

16 Becke, A. D. Density-functional thermochemistry. III. The role of exact exchange. *J. Chem. Phys.* **98**, 5648-5652 (1993).

17 Grimme, S., Antony, J., Ehrlich, S. & Krieg, H. A consistent and accurate ab initio parametrization of density functional dispersion correction (DFT-D) for the 94 elements H-Pu. *J. Chem. Phys.* **132** (2010).

18 Stephens, P. J., Devlin, F. J., Chabalowski, C. F. & Frisch, M. J. Ab Initio Calculation of Vibrational Absorption and Circular Dichroism Spectra Using Density Functional Force Fields. *J. Chem. Phys.* **98**, 11623-11627 (1994).

19 Vosko, S. H., Wilk, L. & Nusair, M. Accurate spin-dependent electron liquid correlation energies for local spin density calculations: a critical analysis. *Can. J. Phys.* **58**, 1200-1211 (1980).
20 Hariharan, P. C. & Pople, J. A. The influence of polarization functions on molecular orbital hydrogenation energies. *Theor. Chim. Acta* **28**, 213-222 (1973).
21 Hehre, W. J., Ditchfield, R. & Pople, J. A. Self—Consistent Molecular Orbital Methods. XII. Further Extensions of Gaussian—Type Basis Sets for Use in Molecular Orbital Studies of Organic Molecules. *J. Chem. Phys.* **56**, 2257-2261 (1972).
22 Ditchfield, R., Hehre, W. J. & Pople, J. A. Self-Consistent Molecular-Orbital Methods. IX. An Extended Gaussian-Type Basis for Molecular-Orbital Studies of Organic Molecules. *J. Chem. Phys.* **54**, 724-728 (1971).
23 Hay, P. J. & Wadt, W. R. Ab initio effective core potentials for molecular calculations. Potentials for K to Au including the outermost core orbitals. *J. Chem. Phys.* **82**, 299-310 (1985).
24 Ufimtsev, I. S. & Martinez, T. J. Quantum Chemistry on Graphical Processing Units. 3. Analytical Energy Gradients, Geometry Optimization, and First Principles Molecular Dynamics. *J. Chem. Theory Comput.* **5**, 2619-2628 (2009).
25 Ufimtsev, I. S. & Martínez, T. J. Quantum Chemistry on Graphical Processing Units. 1. Strategies for Two-Electron Integral Evaluation. *J. Chem. Theory Comput.* **4**, 222-231 (2008).
26 Seritan, S. *et al.* TeraChem: A graphical processing unit-accelerated electronic structure package for large-scale ab initio molecular dynamics. *Wiley Interdiscip. Rev. Comput. Mol. Sci.* **11**, e1494 (2021).
27 Yanai, T., Tew, D. P. & Handy, N. C. A new hybrid exchange–correlation functional using the Coulomb-attenuating method (CAM-B3LYP). *Chem. Phys. Lett.* **393**, 51-57 (2004).
28 Weigend, F. & Ahlrichs, R. Balanced basis sets of split valence, triple zeta valence and quadruple zeta valence quality for H to Rn: Design and assessment of accuracy. *Phys. Chem. Chem. Phys.* **7**, 3297-3305 (2005).
29 Andrae, D., Häußermann, U., Dolg, M., Stoll, H. & Preuß, H. Energy-adjustedab initio pseudopotentials for the second and third row transition elements. *Theor. Chim. Acta* **77**, 123-141 (1990).
30 Furche, F. *et al.* Turbomole. *Wiley Interdiscip. Rev. Comput. Mol. Sci.* **4**, 91-100 (2014).
31 Bannwarth, C., Ehlert, S. & Grimme, S. GFN2-xTB—An Accurate and Broadly Parametrized Self-Consistent Tight-Binding Quantum Chemical Method with Multipole Electrostatics and Density-Dependent Dispersion Contributions. *J. Chem. Theory Comput.* **15**, 1652-1671 (2019).
32 Unke, O. T. *et al.* SpookyNet: Learning force fields with electronic degrees of freedom and nonlocal effects. *Nat. Commun.* **12**, 7273 (2021).
33 Wood, B. M. *et al.* UMA: A Family of Universal Models for Atoms. *arXiv* (2025), https://doi.org/10.48550/arXiv.2506.23971.
34 Kästner, J. *et al.* DL-FIND: An Open-Source Geometry Optimizer for Atomistic Simulations. *J. Phys. Chem. A* **113**, 11856-11865 (2009).
35 Metz, S., Kästner, J., Sokol, A. A., Keal, T. W. & Sherwood, P. ChemShell—a modular software package for QM/MM simulations. *Wiley Interdiscip. Rev. Comput. Mol. Sci.* **4**, 101-110 (2014).
36 Loshchilov, I. & Hutter, F. Decoupled Weight Decay Regularization. *arXiv* (2019), https://doi.org/10.48550/arXiv.1711.05101.
37 Loshchilov, I. & Hutter, F. SGDR: Stochastic Gradient Descent with Warm Restarts. *arXiv* (2017), https://doi.org/10.48550/arXiv.1608.03983.

38 Jónsson, H., Mills, G. & Jacobsen, K. W. Nudged elastic band method for finding minimum energy paths of transitions. *Classical and Quantum Dynamics in Condensed Phase Simulations* 385-404 (World Scientific Publishing Co Pte Ltd, 1998).
39 Henkelman, G., Uberuaga, B. P. & Jónsson, H. A climbing image nudged elastic band method for finding saddle points and minimum energy paths. *J. Chem. Phys.* **113**, 9901-9904 (2000).
40 Ong, M. T., Leiding, J., Tao, H., Virshup, A. M. & Martínez, T. J. First Principles Dynamics and Minimum Energy Pathways for Mechanochemical Ring Opening of Cyclobutene. *J. Am. Chem. Soc.* **131**, 6377-6379 (2009).
41 Beyer, M. K. The mechanical strength of a covalent bond calculated by density functional theory. *J. Chem. Phys.* **112**, 7307-7312 (2000).
42 Ribas-Arino, J., Shiga, M. & Marx, D. Understanding Covalent Mechanochemistry. *Angew. Chem., Int. Ed.* **48**, 4190-4193 (2009).
43 Bussi, G. & Parrinello, M. Accurate sampling using Langevin dynamics. *Phys. Rev. E* **75**, 056707 (2007).
44 Hagberg, A., Swart, P. J. & Schult, D. A. Exploring Network Structure, Dynamics, and Function Using NetworkX. *osti.gov* (2007), https://www.osti.gov/biblio/960616.
45 Martínez, L., Andrade, R., Birgin, E. G. & Martínez, J. M. PACKMOL: A package for building initial configurations for molecular dynamics simulations. *J. Comput. Chem.* **30**, 2157-2164 (2009).
46 Virtanen, P. *et al.* SciPy 1.0: fundamental algorithms for scientific computing in Python. *Nat. Methods* **17**, 261-272 (2020).
