# Supplementary material for: Mechanochemical disassembly pathways of self-assembled polymer-decorated PdnL2n supramolecular architectures
Source: Nat Commun. 2026 Jun 19;17:5459. doi: 10.1038/s41467-026-74561-4 (PMC13282382; doi:10.1038/s41467-026-74561-4)
Supplement: Supplementary file 2 — Transparent Peer Review file [file 41467_2026_74561_MOESM2_ESM.pdf]

nature portfolio

Peer Review File

Mechanochemical disassembly pathways of self-assembled polymer-decorated PdnL2n supramolecular architectures

Corresponding Author: Dr Bernd Schmidt

This file contains all reviewer reports in order by version, followed by all author rebuttals in order by version.

Version 0:

Reviewer comments:

Reviewer #1

(Remarks to the Author)
This interesting manuscript reports the mechanochemical disassembly of Pd2L4 cages composed of polymer-tethered tridentate ligands, PolyL1–4 having a PEG chain of varying molecular weight (from 1 kDa to 20 kDa). In particular, PolyL4 having the largest PEG chain (20 kDa) was used to create a large metal organic cage (MOC), PolyMOS1, a PEG-decorated Pd12L24 cage. Similar MOCs have been used to create polymer gels due to their controllable functionality (f) (Nat Chem. 2016, 8, 33). In this work, the authors investigated the mechanical disassembly of PolyMOCs using sonication in solution and ball milling. A series of PolyMOC1–4 has been synthesized and fully characterized by NMR and MALDI-MS. However, the synthesis of PolyMOC4 might require further proof as the ligand PolyL4 was a mixture of the synthesized ligand and free PEG. The resulting PolyMOCs were subjected to ultrasonication in CD3CN using N2 or Ar as the saturation gas. NMR spectra of these solutions showed disassembly of the MOCs, liberating free ligands. The mode of disassembly of MOCs was estimated by computational simulation, suggesting that PEG chains could be pulled to liberate ligands to create Pd2L2 fragment, for example. These PEG-decorated MOCs could be potentially used as an encapsulant for cisplatin. Mechanical activation of this MOC could release cisplatin in a stimuli-responsive manner.
This work introduces the disassembly of MOCs using mechanical force as a stimulus through experiments and computational simulations. In addition, some of the results suggest potential applications of PEG-decorated MOCs. However, this work does not offer new and exciting discoveries or insights to the Nat Commun readership. I feel that this manuscript is better suited for more specialized journals.

Reviewer #2

(Remarks to the Author)
The manuscript presents an ambitious and multifaceted study of the mechanochemically induced disassembly of polymer-decorated PdnL2n supramolecular coordination architectures. The authors convincingly integrate advanced supramolecular and polymer chemistry with sonochemistry and state-of-the-art molecular dynamics simulations based on a fine-tuned machine-learning interatomic potential (MLIP), complemented by a functional demonstration of controlled cisplatin release. Importantly, the work goes beyond a single model system, encompassing both classical Pd2L4 cages and exceptionally large Pd12L24 nanospheres, thereby lending the study a clear sense of generality. Overall, the presented approach provides new and meaningful insights into force-induced kinetic disassembly pathways of coordination assemblies and constitutes a significant contribution to the fields of supramolecular mechanochemistry and responsive materials.
The synthetic procedures and structural characterization of the polymer-decorated ligands and cages are generally sound and well documented. The NMR, DOSY, MS and IR data are consistent with the proposed architectures, and the authors appropriately acknowledge the limitations associated with the largest polymer-decorated systems. However, for systems with incomplete conversion or mixtures with free PEG, this limitation (if any) should be more clearly stated in the main text to avoid overinterpretation of quantitative trends.
The sonochemical activation experiments are carefully designed, with systematic variation of polymer chain length, concentration, saturation gas and solvent. The experimental trends are internally consistent and in good agreement with established sonochemical principles. Nevertheless, the definition of the “activation ratio” (new species formed per cage) is not fully transparent and would benefit from a clearer, quantitative definition or reformulation to improve interpretability for the reader. Additional evidence supporting the formation of Pd2L3 as well as Pd2L2 species (e.g., ESI-MS or DOSY NMR data) would further strengthen the structural assignments. The presentation of Fig. 2b-d is somewhat non-intuitive and difficult to

follow, particularly in comparison with the discussion in the main text (lines 163 - 168). Moreover, the authors refer to a red curve in Fig. 2d, whereas only blue and green curves are visible.
The demonstration of reversible disassembly and reassembly, including the use of solid-state ball milling as an alternative mechanical activation pathway, is a strong aspect of the manuscript. This section convincingly extends the scope of the study beyond ultrasound-based activation and highlights the robustness and generality of the proposed mechanochemical concept. However, this section deserves to be highlighted more strongly in the Abstract or at least in the Conclusion paragraph.
The proof-of-concept experiments on ultrasound-triggered cisplatin release are well executed and supported by consistent NMR evidence. The influence of the saturation gas on the disassembly pathway is clearly demonstrated.
Other Issue:
1) The term PolyMOCs is commonly used in the literature to describe polymers formed by interconnected metal–organic cages. In this context, the nomenclature adopted in the present manuscript may be slightly misleading. The authors may wish to consider renaming their compounds to avoid potential confusion for readers.
2) Editorial corrections are required throughout the manuscript. These include overly long sentences in the Introduction and typographical inconsistencies: e.g., Pd2L2n (it should be rather PdnL2n). In supplementary information: Synthesis of PolyMOC3 - 0.75eq of Pd salt over the arrow, 0.78eq in the description; Synthesis of PolyMOC4 - 1.5eq of Pd salt over the arrow, 1eq in the description; similar situations with PolyMOS1, encapsulation of cis-platin to MOC2 in DMF. Formatting errors are also present (missing superscript in 1H NMR on the page 47, multiple mistakes such as "Figure S48: Stacked 1H NMR spectra (600 MHz, D2O, 298 K) of PolyMOC1-3 after six hours of sonochemical activation in CD3CN "
3) Fig 3a does not display units on the axes
4) Depiction "1H NMR" in Supplementary Information appears in inconsistent fonts, see for example. Figure S60 and S61
5) Given the large number of variables explored (including solvent, polymer chain length, concentration, and saturation gas), the results are difficult to follow in their current presentation. A comprehensive summary table compiling all key experimental conditions and corresponding outcomes is necessary to ensure clarity and allow the reader to properly assess trends and reproducibility. This should be included, at minimum, in the Supplementary Information.
Recommendation: Major revision

Reviewer #3

(Remarks to the Author)
Key results

Key findings:

* Ultrasound leads to shear-induced disassembly of supramolecular PdnL2n.

* This allows controlled release of anticancer drug.

* Molecular dynamics (MD) identify force thresholds for Pd-N bond rupture.

The study demonstrates that ultrasound can induce shear forces sufficient to trigger the disassembly of supramolecular Pd_nL_{2n} coordination cages, enabling externally controlled release of an encapsulated anticancer drug. A particularly notable aspect is the mechanistic insight provided by molecular dynamics simulations, which quantify the force thresholds required for Pd–N bond rupture.

Key findings of simulation specifically:

* Steered MD explores four different pulling modes, labeled single cis, dual cis, single trans, dual trans.

* cis pulling leads to faster activation (disassembly) than trans pulling

* Starting with Pd2L4, Pd2L2 fragment formation more likely than Pd2L3 fragment formation in dissociation

* Starting with Pd12L24, Pd2L4, Pd1L4, and Pd1L3 fragment formation most likely.

The authors use steered molecular dynamics to systematically compare four distinct pulling geometries, single cis, dual cis, single trans, and dual trans, revealing a clear mechanistic dependence of cage activation on force directionality. Notably, cis pulling modes lead to more rapid disassembly than trans configurations. Furthermore, the simulations indicate that Pd_2L_2 fragments are formed more frequently than Pd_2L_3 species during dissociation

Validity

For Pd2L4, the MD simulations are well-documented, technically solid and statistically reliable. Four different pulling modes have been simulated with ten trajectories each for three different pulling forces, with an additional five trajectories without an applied external force. This means a parametric study in a three-dimensional parametric space spanned by (pulling mode, pulling force, replica), amounting to 140 trajectories in total. The simulation time of 500 ps each is short, but apparently sufficient as shown by the dissociation events illustrated in Fig. 3c and d.

For Pd12L24, the MD simulations are not as unambiguously documented in the main body. 120 MD simulations were performed. It is, however, unclear how these 120 runs have been distributed across the three different pulling forces, pulling modes, and replicas. Simulation times are not stated. The Supporting Information clearly states the parameter matrix on p. 126, Table S6, but the total number of simulations here is in disagreement with the 120 trajectories mentioned in the main body.

The very detailed Supporting Information, however, gives a very thorough simulation protocol that allows full trust in the computational results' validity and robustness. With minor edits, main body and Supporting Information can certainly be brought to smooth agreement.
Significance

Judging the scientific significance of the findings exceeds my expertise. However, I encourage the authors to clarify the significance of their computational results within the wider scope of their experimental work. To me as a reader, the importance of embedding the well-conducted computational study within this manuscript is not obvious. In it's current form, the manuscript could function as a purely experimental study equally well even if the computational contribution was missing.

Data and methodology

ll. 501-503 (Methods: Computations)

"A Python-based software that integrates TeraChem69,70 and the FAIRChem package71 was used to perform steered MD and RS-MD, respectively."

The authors mention a Python-based software but do not provide more detail. A little more detail is "hidden" in the Supporting Information, p. 122. The mentioned software is an in-house development. I encourage the authors to publish their code on github, gitlab, or Zenodo.

ll. 503-506 (Methods: Computations)

"For the dataset creation, calculations were performed on the level of density functional theory (DFT) employing ..."

The authors do not specify what "the dataset" refers to. The subsequent mentioning of DFT-level computations and ll. 236-238 suggest it is the generation of training data for the fine-tuned MLIP. Please make this clearer.

ll 506-507 (Methods: Computations)

"The RS-MD simulations were conducted using a finetuned UMA-S model."

Do I understand correctly that this fine-tuned model results from MLIP training on the dataset described in the preceding sentence?

ll. 513-517 (Data availability)

The authors state that "the Source Data are openly available in Zenodo at https://doi.org/10.5281/zenodo.17939706". However, in the manuscript they do not state clearly whether this Source Data is raw experimental data, simulation results, or both. The dataset published on Zenodo contains a short description of the contents:

'This Zenodo record contains data associated with the paper "Mechanochemical disassembly pathways of self-assembled PdnL2n supramolecular architectures." It includes the fine-tuned Universal Model for Atoms S (UMA-S-PdN) provided as a PyTorch checkpoint (.pt), as well as training/validation/test datasets in ASE LMDB format (.aselmdb). Example trajectories for the two systems studied in this work are provided in XYZ format (.xyz) and include every 100th frame from the original trajectories. Minimal Python code is included to load the .aselmdb files.'

Thus, I understand that the published dataset contains the trained MLIP model used in this study as well as the training data. The short description of file types '.aselmdb' and '.xyz' , however, does not make it very clear how properly access and possibly reuse the provided tar archives. I strongly suggest adding a README file to the data repository that outlines a minimal access protocol for the provided archives.

Analytical approach

The Supporting Information conveys convincingly the statistical robustness of all computational results and the derived properties, such as dissociation forces, fragment frequencies, and activation energies.

Suggested improvements

ll. 14-17(Abstract)

“Whereas their thermodynamically driven assembly has been extensively studied, controlled strategies for disassembly remain scarce. Here we show that ultrasound provides a powerful stimulus to trigger and probe disassembly pathways.”

vs ll. 429-432 (Conclusion)

“In summary, we have developed a versatile synthetic strategy for polymer-decorated ligands that enables the preparation of metallosupramolecular cage mechanophores across the PdnL2n family.”

When initially skimming abstract and conclusion, the reader might feel confused about the key content of this work. The abstract makes us anticipate a thorough study on disassembly pathways, while the conclusion initially claims a synthesis study.

ll. 32-74 (Introduction)

The introduction puts the experimental part of this study into context, but not the computational part. I suggest embedding the steered MD simulations into relevant computational literature as well for context

Furthermore, neither abstract nor introduction make clear why molecular dynamics studies are necessary next to the experimental work. What additional insight do we gain from MD that experiments cannot provide?

ll. 231-234 (Computational simulations)

“Simulating the disassembly of PolyMOCs poses a serious challenge for quantum chemical methods due to the large system size, particularly when including solvent molecules explicitly. A tremendous speedup can be achieved when a machine-learnt interatomic potential (MLIP) is used.”

The authors make the jump from ab-initio methods to MLIP directly. Why are classical interatomic potentials not an option? What are the benefits of MLIP above classical force field families here?

ll. 235-238 (Computational simulations)

“FAIRChem’s pre-trained Universal Model for Atoms S (UMA-S)67, however, leads to highly asymmetric, deformed structures, which is an indicator for improper description (Supplementary Figure S97). Therefore, we fine-tuned UMA-S to describe the disassembly of the Pd2L4 PolyMOCs (Supplementary Information, Chapter XII).”

Why is the UMA-S MLIP chosen for this study? What other MLIP are available, and why are they rejected in favor of UMA-S, in particular when the “out-of-the-box” pre-trained UMA-S apparently perform poorly and requires fine-tuning? These question arose when reading the section, and I only found out that the benchmark on pp. 114-115 of the Suppl. Inf. addresses them well later. Consider explicitly stating that these important aspects are discussed there in the main body.

ll. 296-305 (Fig. 3)

Fig. 3a is superfluous and could be omitted for the sake of brevity. l. 244 eq. 1 states clearly the linearity of pulling force and no visualization if the linear time-dependency is necessary.

I recommend adding a legend to Fig. 3c. The caption clarifies the meaning of blue and purple plots, but labels or a legend embedded in the figure would make it more significant.

I did not understand that Fig. 3b shows top views and Fig. 3c shows side views of the same complex until I saw Figure S97 of the supplementary information. Maybe some coordinate axes along the structures or explanation in the caption would help here.

ll. 376-379 (Fig. 5d)

From the text body and the computational results presented in Fig. 3, it is possible to infer the meaning of the colored arrows in the first panel of Fig. 5d as an illustration of pulling directions. For clarity, a legend and an explanation in the caption should be added to make the figure self-consistent.

ll. 402-417

More computational results are “hidden” here as a little appendix to an otherwise experimental section. I almost overlooked them. Why are they not presented on the same level of prominence as the previous computational section of l. 230ff, in particular as the methodology parallels this previous section exactly? Could it be beneficial for the structure of the whole manuscript to present the Pd2L4 and Pd12L24 simulation results together? From my point of view as a reader, the latter merely adds another dimension, the actual molecular structure, to the previously explored three-dimensional parametric space of pulling mode, pulling force, and replica.

It is unclear how the mentioned 120 runs have been distributed across the three different pulling forces, pulling modes, and replicas.

Simulation times are not stated explicitly. Fig. 6 implies 100 ps.

ll. 422-423 (Fig. 6)

“Fragments that occur less than ten times in the 100 simulations are marked in grey.”

100 simulations mentioned here contradict 120 simulations mentioned on l. 402.

ll. 501-503 (Methods: Computations)

“A Python-based software that integrates TeraChem69,70 and the FAIRChem package71 was used to perform steered MD and RS-MD, respectively.”

Steered MD and RS-MD are mentioned in parallel. This looks like a tautology to me. In the context of this work, I do not understand the distinction here. Only RS-MD (ramped, steered molecular dynamics) results have been presented in the main body. These are, of course, steered.

The Supporting Information describes steered MD runs that are not ramped. They are, however, not presented in the main body.

Supporting information, Benchmarking, p. 114

“The total charge of the system is +2”

How is a system of non-neutral charge handled in your MD simulations?

Supporting information, Generation of ML training data, p. 116

“To construct the dataset for fine-tuning UMA-S, steered MD simulations for six different molecular fragments were conducted (Figure S98). The steering was necessary to increase the diversity of the dataset and to include the effects of a pulling force on molecular structures in the dataset.”

Why was it necessary to include steering for increased diversity? What’s the evidence that the quality of the training dataset would have been poor without pulling?

Supporting information, Generation of ML training data, Figure S98, p. 117

What do the numbers N in the figure caption represent?

Supporting information, Fine tuning UMA-S, p. 119

“... showing very good agreement with the DFT (B3LYP+D3/6-31G*) reference (Figure S5).”

The reference to Figure S5 seems to be wrong.Fig. S100?

Supporting information, Fine tuning UMA-S, p. 120

“Both the MLIP NEB and the DFT energies are shown in Figure SX.”

Again, likely wrong Figure reference. Fig. S101?

Supporting information, RS-MD, p. 121

“Rather than applying an external force of constant strength, we the external force applied in the simulation is gradually increased ...“

Remove “we”

Supporting information, RS-MD, p. 122

“All RS-MD simulations were performed using the UMA-S-PdN described in the previous section. The simulations were run in an NVT ensemble using the Bussi-Parrinello version of Langevin dynamics43 with a timestep of 1 fs, a thermostat temperature of 300 K, and a friction coefficient of 7 ps-1.”

These are important parameters of the simulation protocol and I recommend placing them in the main body’s “Methods:

Computations” section. Also, I recommend replacing the term “friction coefficient” with “thermostat damping parameter” or similar, since this does not refer to a true, mechanical friction coefficient.

Supporting information, RS-MD simulations – setup, Table S6, p. 126

Is the no. of 10 trajectories for ramping rate 17.5 nN/ps reported wrong? Table S11 implies 100 runs.

Clarity and context

The manuscript is written clearly and, for the computational part, I understand very well what has been done. Yet, I have difficulties understanding why it has been done. As mentioned above, I believe the manuscript would benefit profoundly if the computational results were contextualized properly within the otherwise experimental scope.

Moreover, I believe the second batch of computational results on Pd12L24 is obscured a little and would benefit from a presentation on par with the Pd12L24 results.

References

The manuscript does not attempt to embed its computational contribution into relevant literature.
Your expertise

I am trained in the disciplines of physics and engineering sciences with a heavy focus on computational methods. I specialize on computational nanotribology, with a specific methodological focus on molecular dynamics simulations of interfacial phenomena and FAIR data. Hence, I see myself in a valid position to evaluate the modeling and data-handling aspects of this manuscript. I have no chemistry background, hence commenting on the manuscript’s experimental merits would exceed my expertise.

Hence, my review specifically targets the main body’s Abstract(ll. 12-30), Introduction (ll. 32-74), Results and Discussion: Computational simulations of PolyMOC disassembly (ll. 230-305, inc. Fig. 3), an additional computational section without separate heading (ll. 402-427, inc. Fig. 6), Conclusion (ll. 429-453), Methods: Computations (ll. 501-507), and Data Availability (ll. 513-517) as well as the Supporting Information’s chapter XII: Molecular dynamics simulations and computational analysis of PdnL2n disassembly (p. 114-139).

Reviewer #4

(Remarks to the Author)
I co-reviewed this manuscript with one of the reviewers who provided the listed reports. This is part of the Nature Communications initiative to facilitate training in peer review and to provide appropriate recognition for Early Career Researchers who co-review manuscripts.

Version 1:

Reviewer comments:

Reviewer #2

(Remarks to the Author)
The authors have carefully addressed all of the points and concerns raised in my previous review. The revised manuscript has been significantly improved, and the clarifications and additional data provided satisfactorily resolve my comments. I therefore recommend acceptance of the manuscript in its present form.

Reviewer #3

(Remarks to the Author)
I thank the authors for addressing all minor issues very thoroughly and in detail. Every single issue raised by me and my colleague has been addressed.

Reviewer #4

(Remarks to the Author)
I co-reviewed this manuscript with one of the reviewers who provided the listed reports. This is part of the Nature Communications initiative to facilitate training in peer review and to provide appropriate recognition for Early Career Researchers who co-review manuscripts.

Open Access This Peer Review File is licensed under a Creative Commons Attribution 4.0 International License, which permits use, sharing, adaptation, distribution and reproduction in any medium or format, as long as you give appropriate credit to the original author(s) and the source, provide a link to the Creative Commons license, and indicate if changes were made.
In cases where reviewers are anonymous, credit should be given to 'Anonymous Referee' and the source.
The images or other third party material in this Peer Review File are included in the article's Creative Commons license, unless indicated otherwise in a credit line to the material. If material is not included in the article's Creative Commons license and your intended use is not permitted by statutory regulation or exceeds the permitted use, you will need to obtain permission directly from the copyright holder.
To view a copy of this license, visit https://creativecommons.org/licenses/by/4.0/

REVIEWER COMMENTS

Reviewer #1 (Remarks to the Author):

R1.1 This interesting manuscript reports the mechanochemical disassembly of Pd2L4 cages composed of polymer-tethered tridentate ligands, PolyL1–4 having a PEG chain of varying molecular weight (from 1 kDa to 20 kDa). In particular, PolyL4 having the largest PEG chain (20 kDa) was used to create a large metal organic cage (MOC), PolyMOS1, a PEG-decorated Pd12L24 cage. Similar MOCs have been used to create polymer gels due to their controllable functionality (f) (Nat Chem. 2016, 8, 33). In this work, the authors investigated the mechanical disassembly of PolyMOCs using sonication in solution and ball milling. A series of PolyMOC1–4 has been synthesized and fully characterized by NMR and MALDI-MS. However, the synthesis of PolyMOC4 might require further proof as the ligand PolyL4 was a mixture of the synthesized ligand and free PEG. The resulting PolyMOCs were subjected to ultrasonication in CD3CN using N2 or Ar as the saturation gas. NMR spectra of these solutions showed disassembly of the MOCs, liberating free ligands. The mode of disassembly of MOCs was estimated by computational simulation, suggesting that PEG chains could be pulled to liberate ligands to create Pd2L2 fragment, for example. These PEG-decorated MOCs could be potentially used as an encapsulant for cisplatin. Mechanical activation of this MOC could release cisplatin in a stimuli-responsive manner. This work introduces the disassembly of MOCs using mechanical force as a stimulus through experiments and computational simulations. In addition, some of the results suggest potential applications of PEG-decorated MOCs. However, this work does not offer new and exciting discoveries or insights to the Nat Commun readership. I feel that this manuscript is better suited for more specialized journals.

We sincerely thank the reviewer for the careful evaluation of our manuscript and for the thoughtful comments. We greatly appreciate the time and expertise invested in assessing our work. We would like to emphasise that the reviewer did not identify any technical errors or other scientific shortcomings in our work, which we greatly appreciate. Regarding the reviewer's concern, we respectfully offer a pointed summary showcasing our perspective.

In our view, this study introduces several conceptually new and meaningful contributions to the field:

1. Mechanochemical activation of discrete metal-organic cages in solution

While mechanochemistry has been extensively explored in polymer systems and bulk materials, controlled mechanochemical disassembly of well-defined supramolecular metal-organic cages in solution remains largely unexplored. Our work demonstrates that mechanical force can be used as a deliberate stimulus to trigger structural disassembly pathways of discrete MOCs.

2. Polymer-tethered cage architectures as mechanochemically addressable systems

By incorporating PEG chains of systematically varied molecular weight, we establish a platform for controlled force transduction into discrete supramolecular architectures and, importantly, enable a systematic experimental investigation of sonochemical parameters, including chain length dependence, concentration effects, and the role of the saturation gas. Such a comprehensive analysis has thus far remained unexplored, especially in the context of discrete metal-organic systems. Importantly, these findings provide fundamental insight into the conditions required for efficient sonochemical activation, offering principles that are broadly transferable beyond the specific systems studied here and thereby contributing significantly to the development of force-responsive supramolecular systems.

3. Mechanistic insight through combined experimental and computational analysis

The computational simulations based on a fine-tuned machine-learning interatomic potential deliver molecular-level insight into force-induced ligand dissociation pathways and provide a mechanistic framework that not only supports but also rationalises the experimentally observed disassembly, thereby bridging macroscopic mechanical input with molecular-scale structural response. The established computational simulation and analysis tools will support researchers in this field.

4. Conceptual implications for stimuli-responsive drug delivery systems

While we agree that the cisplatin encapsulation aspect is presented as a prospective application, the work establishes a general strategy for mechanically triggered cargo release from discrete supramolecular containers, a concept that broadens the scope of stimuli-responsive MOCs beyond traditional chemical, thermal, or photochemical triggers.

We respectfully align with the assessment of reviewer 2 that the study provides new and meaningful insights into the emerging area of force-responsive supramolecular architectures. In particular, the combination of polymer mechanochemistry with well-defined metal-organic cage chemistry and analysis of molecular dynamics simulations opens a new conceptual direction for stimulus-controlled disassembly processes. We therefore believe that the manuscript contributes to a growing interdisciplinary field at the interface of supramolecular chemistry, materials science, sonochemistry and mechanochemistry, and may be of interest to the broad readership of Nature Communications.

R1.2 However, the synthesis of PolyMOC4 might require further proof as the ligand PolyL4 was a mixture of the synthesized ligand and free PEG.

We thank the reviewer for raising this important point regarding the purity and structural assignment of PolyMOC4. The presence of residual, non-converted PEG that could not be separated from PolyL4 is fully and transparently disclosed in the Supporting Information, and now, see below, this is also stated clearly in the main manuscript. In Figures S160 (^{1}H NMR of PolyL4) and S165 (^{1}H NMR of PolyMOC4), the signals corresponding to free PEG are explicitly labelled for both PolyL4 and PolyMOC4. This residual PEG fraction was carefully accounted for the yield calculation.

*"Filtration, followed by washing with diethyl ether and drying in air, yields a 1:2 mixture of **PolyL4** and **mPEG$_{20kDa}$** (1.12 g, 55.2 µmol, 75 %) as a colorless powder, containing **PolyL4** (0.37 g, 18.2 µmol, 25 %)."*

*"Precipitation in cold diethyl ether and subsequent washing with cold diethyl ether before drying in air yields a 1:16 mixture of **PolyMOC4** and **mPEG$_{20kDa}$** (0.17 g, 8.12 µmol, 83 %), containing **PolyMOC4** (10.0 mg, 0.12 µmol, 15 %) as a colorless solid."*

Importantly, the ^{1}H NMR spectra of both PolyL4 and PolyMOC4 show a single, well-defined signal set attributable to one discrete species for the ligand scaffold and the corresponding assembled cage. No additional signal sets indicative of multiple cage species or structural heterogeneity are observed. The chemical shifts are fully consistent with those of the other isostructural compounds in the series. Furthermore, upon metal coordination, all signals exhibit the characteristic downfield shifts. The magnitude and pattern of these coordination-induced shifts are fully comparable to those of PolyMOC1–3, strongly supporting successful cage formation in the case of PolyMOC4. Taken together, the spectroscopic data consistently indicate the formation of a single, well-defined Pd-based assembly, and we therefore believe that the structural assignment of PolyMOC4 is well supported. Additionally, we have added a statement in the main text highlighting this limitation and clarifying how the ratios of PolyL4 and PolyMOC4 to unreacted mPEG were determined, ensuring accurate representation of the quantitative trends.

"Complete conversion of the mPEG could not be achieved due to the low reactivity, hampered diffusion, and increased viscosity of the 20 kDa mPEG in solution."

"Notably, increasing the mPEG chain length resulted in prolonged assembly times, with reactions requiring one hour for PolyMOC1-3 and up to four hours for PolyMOC4. Because the ligand precursor PolyL4 was used as a mixture containing residual unreacted mPEG, the self-assembly afforded a mixture of PolyMOC4 and excess mPEG. Given that four ligands are incorporated per cage, unreacted mPEG accumulates in the final mixture, leading to an overall PolyMOC4 to mPEG ratio of 1:16. This ratio was determined by integration of the ^{1}H NMR signals assigned to the free hydroxyl group of mPEG and the aromatic protons of the ligand or assembled cage (see Supplementary Information, Figure S160 and S165)."

Reviewer #2 (Remarks to the Author):

R2.1 The manuscript presents an ambitious and multifaceted study of the mechanochemically induced disassembly of polymer-decorated PdnL2n supramolecular coordination architectures. The authors convincingly integrate advanced supramolecular and polymer chemistry with sonochemistry and state-

of-the-art molecular dynamics simulations based on a fine-tuned machine-learning interatomic potential (MLIP), complemented by a functional demonstration of controlled cisplatin release. Importantly, the work goes beyond a single model system, encompassing both classical Pd2L4 cages and exceptionally large Pd12L24 nanospheres, thereby lending the study a clear sense of generality. Overall, the presented approach provides new and meaningful insights into force-induced kinetic disassembly pathways of coordination assemblies and constitutes a significant contribution to the fields of supramolecular mechanochemistry and responsive materials. The synthetic procedures and structural characterization of the polymer-decorated ligands and cages are generally sound and well documented. The NMR, DOSY, MS and IR data are consistent with the proposed architectures, and the authors appropriately acknowledge the limitations associated with the largest polymer-decorated systems.

We sincerely thank the reviewer for the careful evaluation of our manuscript and for the highly encouraging comment. We are particularly grateful for the recognition that our study provides new and meaningful insights into force-induced disassembly pathways of coordination assemblies and represents a significant contribution to supramolecular mechanochemistry and responsive materials, as it reflects the central conceptual aim of our work. We also thank the reviewer for the constructive feedback and comments. We have addressed all points raised and revised the manuscript accordingly to further strengthen the clarity.

R2.2 However, for systems with incomplete conversion or mixtures with free PEG, this limitation (if any) should be more clearly stated in the main text to avoid overinterpretation of quantitative trends.

We thank the reviewer for this valuable suggestion. We agree that clarity regarding incomplete conversion or the presence of free PEG is important to avoid overinterpretation. In response, we have added a statement in the main text highlighting this limitation, clarifying how the ratios of PolyL4 and PolyMOC4 to unreacted mPEG were determined, ensuring accurate representation of the quantitative trends. We further showcased the product distribution in the final mixtures in the experimental procedures.

"Complete conversion of the mPEG could not be achieved due to the low reactivity, hampered diffusion, and increased viscosity of the 20 kDa mPEG in solution."

"Notably, increasing the mPEG chain length resulted in prolonged assembly times, with reactions requiring one hour for PolyMOC1-3 and up to four hours for PolyMOC4. Because the ligand precursor PolyL4 was used as a mixture containing residual unreacted mPEG, the self-assembly afforded a mixture of PolyMOC4 and excess mPEG. Given that four ligands are incorporated per cage, unreacted mPEG accumulates in the final mixture, leading to an overall PolyMOC4 to mPEG ratio of 1:16. This ratio was determined by integration of the ^{1}H NMR signals assigned to the free hydroxyl group of mPEG and the aromatic protons of the ligand or assembled cage (see Supplementary Information, Figure S160,165)."

*"Filtration, followed by washing with diethyl ether and drying in air, yields a 1:2 mixture of **PolyL4** and **mPEG$_{20kDa}$** (1.12 g, 55.2 µmol, 75 %) as a colorless powder, containing **PolyL4** (0.37 g, 18.2 µmol, 25 %)."*

*"Precipitation in cold diethyl ether and subsequent washing with cold diethyl ether before drying in air yields a 1:16 mixture of **PolyMOC4** and **mPEG$_{20kDa}$** (0.17 g, 8.12 µmol, 83 %), containing **PolyMOC4** (10.0 mg, 0.12 µmol, 15 %) as a colorless solid."*

R2.3 The sonochemical activation experiments are carefully designed, with systematic variation of polymer chain length, concentration, saturation gas and solvent. The experimental trends are internally consistent and in good agreement with established sonochemical principles. Nevertheless, the definition of the "activation ratio" (new species formed per cage) is not fully transparent and would benefit from a clearer, quantitative definition or reformulation to improve interpretability for the reader.

We thank the reviewer for highlighting this important point. We agree that the original definition of the "activation ratio" was not clear enough. In response, we have revised this section of the manuscript and now report the activation efficiency in a more transparent and quantitative manner, which is also much easier to understand at a glance. Specifically, we provide the final product distribution in percent, detailing the fractions of intact PolyMOC, Pd_2L_3 species, and free ligand present in the mixture after activation. The calculation procedure is described in detail in the SI to ensure full transparency and

reproducibility. We are grateful for this constructive comment, which has allowed us to significantly improve the clarity.

<u>R2.4</u> Additional evidence supporting the formation of Pd2L3 as well as Pd2L2 species (e.g., ESI-MS or DOSY NMR data) would further strengthen the structural assignments.

We appreciate the Reviewer's suggestion to provide further analytical data to substantiate the assignment of Pd_2L_3 and Pd_2L_2 species. We explored multiple complementary techniques in this context. ESI- and MALDI-MS measurements were attempted, but at the concentrations required for detection, the transient species are not stable against ionisation, which is true even for most metal-organic cages and is always difficult. In addition, stronger dilution does not allow reliable qualitative analysis due to possible dilution effects. The high molecular weight range of approximately 20 to 50 kDa, together with the polydispersity due to the polymer-decorated metal-organic cages, further complicates mass spectrometric characterisation anyway, since broad peak distributions are obtained. ^{1}H DOSY NMR experiments are likewise not informative, as the spectra are completely dominated by the intense polymer signals, so that no distinct signals of transient species can be resolved meaningfully, but the bigger problem is that T2 relaxation becomes the dominant problem, as large assemblies tumble slowly, giving very short T2, pushing hardware limits. ^{1}H DOSY can only meaningfully be used for regular supramolecular assemblies (up to 5 nm maybe) but becomes inapplicable for vesicles, membranes and micelles. Despite considerable effort, these technical limitations unfortunately prevented additional direct analytical confirmation of the proposed intermediates.

<u>R2.5</u> The presentation of Fig. 2b-d is somewhat non-intuitive and difficult to follow, particularly in comparison with the discussion in the main text (lines 163 - 168). Moreover, the authors refer to a red curve in Fig. 2d, whereas only blue and green curves are visible.

We agree that the original presentation of Fig. 2b-d was not sufficiently intuitive and that the description in the main text could lead to confusion. We are also grateful for pointing out the incorrect reference to a red curve in Fig. 2d. In response, we have revised Fig. 2b-d and implemented a new representation of the data that is significantly clearer and more intuitive, see below.

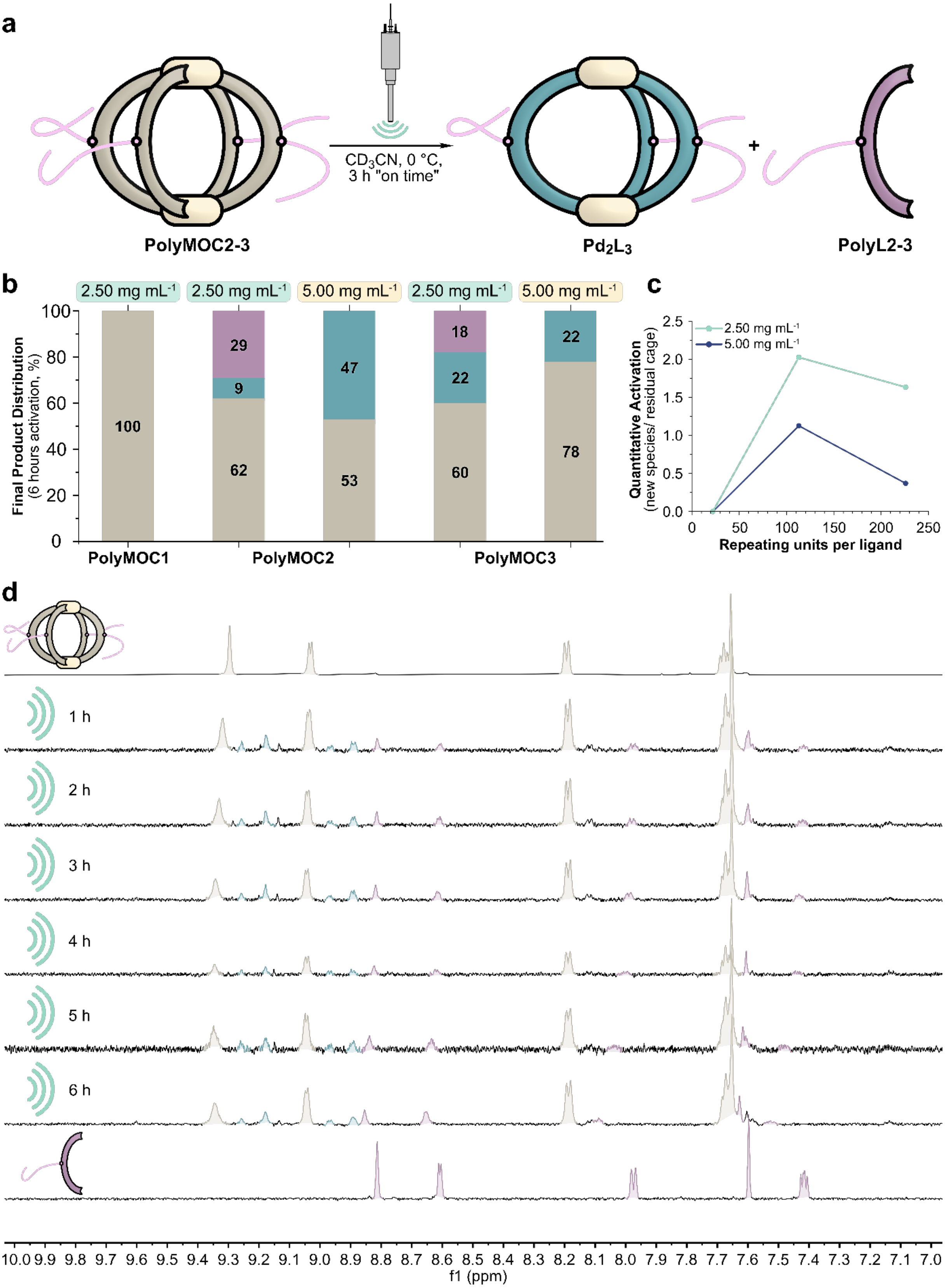

R2.6 The demonstration of reversible disassembly and reassembly, including the use of solid-state ball milling as an alternative mechanical activation pathway, is a strong aspect of the manuscript. This section convincingly extends the scope of the study beyond ultrasound-based activation and highlights the robustness and generality of the proposed mechanochemical concept. However, this section deserves to be highlighted more strongly in the Abstract or at least in the Conclusion paragraph.

We agree that the demonstration of reversible disassembly and reassembly beyond ultrasound-based activation significantly strengthens the generality of the mechanochemical concept. In response to this comment, we have revised both the abstract and the conclusion to more prominently emphasise the role of solid-state ball milling and its relevance to the overall mechanochemical framework presented in this study.

Abstract:
"Furthermore, we demonstrated that quantitative and entirely reversible activation can be further achieved through solid-state ball milling."

Conclusion:
"Additionally, we demonstrated that solid-state ball milling provides a powerful alternative to achieve quantitative disassembly in a fully reversible manner."

The proof-of-concept experiments on ultrasound-triggered cisplatin release are well executed and supported by consistent NMR evidence. The influence of the saturation gas on the disassembly pathway is clearly demonstrated.

Other Issue:

R2.7 The term PolyMOCs is commonly used in the literature to describe polymers formed by interconnected metal-organic cages. In this context, the nomenclature adopted in the present manuscript may be slightly misleading. The authors may wish to consider renaming their compounds to avoid potential confusion for readers.

We thank the reviewer for this valuable comment regarding the nomenclature. We are aware that the term "PolyMOCs" is frequently used to describe polymeric materials formed through interconnected metal-organic cages. However, we believe that the term remains appropriate in the present context. The systems described in our manuscript consist of metal-organic cages that are covalently integrated into polymer chains or bear polymer chains as structural components. In this sense, they represent star-shaped polymeric materials whose macromolecular architecture is defined by the incorporation of MOC motifs. While our systems differ from previously reported PolyMOC hydrogels in that the cages are not interconnected into extended networks, they are nevertheless polymers whose structure and properties arise directly from MOC formation. We therefore consider the designation "PolyMOC" to be conceptually consistent and chemically descriptive, while clearly distinct from networked PolyMOC materials reported in the literature.

R2.8 Editorial corrections are required throughout the manuscript. These include overly long sentences in the Introduction and typographical inconsistencies: e.g., Pd2L2n (it should be rather PdnL2n). In supplementary information: Synthesis of PolyMOC3 - 0.75eq of Pd salt over the arrow, 0.78eq in the description; Synthesis of PolyMOC4 - 1.5eq of Pd salt over the arrow, 1eq in the description; similar situations with PolyMOS1, encapsulation of cis-platin to MOC2 in DMF. Formatting errors are also present (missing superscript in 1H NMR on the page 47, multiple mistakes such as "Figure S48: Stacked 1H NMR spectra (600 MHz, D2O, 298 K) of PolyMOC1-3 after six hours of sonochemical activation in CD3CN "

We sincerely thank the reviewer for the careful and thorough evaluation of the manuscript and Supporting Information. All indicated issues, including compound notation corrections (e.g., Pd_nL_{2n}), inconsistencies in reagent equivalents in the synthetic schemes and descriptions, and formatting errors such as missing superscripts in ^{1}H NMR annotations, have been carefully revised. The manuscript and the Supporting Information have been thoroughly proofread again and corrected to ensure consistency throughout.

R2.9 Fig 3a does not display units on the axes

We thank the reviewer for pointing this out. In the revised manuscript, Figure 3a has been removed during revision because the panel was considered redundant.

R2.10 Depiction "1H NMR" in Supplementary Information appears in inconsistent fonts, see for example. Figure S60 and S61

The font style of "^{1}H NMR" in the Supplementary Information, including Figures S60 and S61, has been corrected to ensure consistent formatting throughout the document.

R2.11 Given the large number of variables explored (including solvent, polymer chain length, concentration, and saturation gas), the results are difficult to follow in their current presentation. A comprehensive summary table compiling all key experimental conditions and corresponding outcomes is necessary to ensure clarity and allow the reader to properly assess trends and reproducibility. This should be included, at minimum, in the Supplementary Information.

We fully agree that, given the number of variables investigated, a consolidated overview greatly improves clarity and facilitates the evaluation of trends. We have included two comprehensive summary tables in the revised Supporting Information: one compiling all performed disassembly experiments and their corresponding experimental conditions and outcomes, and a second summarising all cisplatin release experiments. These tables are provided in the respective sections of the Supporting Information.

"**Table S1**: Summary of disassembly experiments and corresponding conditions."

PolyMOC	Solvent	Polymer Chain Length (RU)	Concentration (mg mL^{-1})	Concentration (µM)	Saturation Gas	Reversibility	Result/Discription
1	CD_3CN	22	2.50	412	Nitrogen	no	no activation 100% inatct PolyMOC1
1	CD_3CN	22	0.77	125	Nitrogen	no	no activation 100% inatct PolyMOC1
2	CD_3CN	113	2.50	125	Nitrogen	no	62% PolyMOC2, 9% Pd_2L_3, 29% PolyL2
2	CD_3CN	113	5.00	250	Nitrogen	no	53% PolyMOC2, 47% Pd_2L_3
2	CD_3CN	113	2.50	125	Argon	no	0% PolyMOC2, 100% Pd_2L_2
2	CD_3CN	113	5.00	250	Argon	no	PolyMOC2 traces, mostly Pd_2L_2
3	CD_3CN	226	2.50	62.5	Nitrogen	no	60% PolyMOC2, 22% Pd_2L_3, 18% PolyL2
3	CD_3CN	226	5.00	125	Nitrogen	**yes**	78% PolyMOC2, 22% Pd_2L_3
3	CD_3CN	226	2.50	62.5	Argon	no	0% PolyMOC3, 100% Pd_2L_2
3	CD_3CN	226	5.00	125	Argon	no	PolyMOC3 traces, mostly Pd_2L_2
3	D_2O	226	2.50	62.5	Nitrogen	no	Oxidation, no corresponding signals after 4 hours
3	D_2O	226	2.50	62.5	Argon	no	Oxidation, no corresponding signals after 3 hours

"**Table S3**: Summary of cisplatin release experiments and corresponding conditions."

PolyMOC	Solvent	Polymer Chain Length (RU)	Concentration (mg mL^{-1})	Saturation Gas	Result/Discription
2	CD_3CN	113	2.50	Nitrogen	Cisplatin release 0.5h, complete PolyMOC dissociation 5h
2	CD_3CN	113	2.50	Argon	Cisplatin release and complete PolyMOC dissociation 0.5h
3	CD_3CN	226	2.50	Nitrogen	Cisplatin release 0.5h, complete PolyMOC dissociation 4h
3	CD_3CN	226	2.50	Argon	Cisplatin release and complete PolyMOC dissociation 0.5h
4	CD_3CN	453	5.00	Nitrogen	Cisplatin released
4	CD_3CN	453	5.00	Argon	Cisplatin released

Reviewer #3 (Remarks to the Author):

R3.1 Key results Key findings:

* Ultrasound leads to shear-induced disassembly of supramolecular PdnL2n.
* This allows controlled release of anticancer drug.
* Molecular dynamics (MD) identify force thresholds for Pd-N bond rupture.

The study demonstrates that ultrasound can induce shear forces sufficient to trigger the disassembly of supramolecular Pd_nL_{2n} coordination cages, enabling externally controlled release of an encapsulated anticancer drug. A particularly notable aspect is the mechanistic insight provided by molecular dynamics simulations, which quantify the force thresholds required for Pd–N bond rupture.

R3.2 Key findings of simulation specifically:

* Steered MD explores four different pulling modes, labeled single cis, dual cis, single trans, dual trans.
* cis pulling leads to faster activation (disassembly) than trans pulling
* Starting with Pd2L4, Pd2L2 fragment formation more likely than Pd2L3 fragment formation in dissociation
* Starting with Pd12L24, Pd2L4, Pd1L4, and Pd1L3 fragment formation most likely.

The authors use steered molecular dynamics to systematically compare four distinct pulling geometries, single cis, dual cis, single trans, and dual trans, revealing a clear mechanistic dependence of cage activation on force directionality. Notably, cis pulling modes lead to more rapid disassembly than trans configurations. Furthermore, the simulations indicate that Pd_2L_2 fragments are formed more frequently than Pd_2L_3 species during dissociation.

R3.3 Validity
For Pd2L4, the MD simulations are well-documented, technically solid and statistically reliable. Four different pulling modes have been simulated with ten trajectories each for three different pulling forces, with an additional five trajectories without an applied external force. This means a parametric study in a three-dimensional parametric space spanned by (pulling mode, pulling force, replica), amounting to 140 trajectories in total. The simulation time of 500 ps each is short, but apparently sufficient as shown by the dissociation events illustrated in Fig. 3c and d.

For Pd12L24, the MD simulations are not as unambiguously documented in the main body. 120 MD simulations were performed. It is, however, unclear how these 120 runs have been distributed across the three different pulling forces, pulling modes, and replicas. Simulation times are not stated. The Supporting Information clearly states the parameter matrix on p. 126, Table S6, but the total number of simulations here is in disagreement with the 120 trajectories mentioned in the main body.

The very detailed Supporting Information, however, gives a very thorough simulation protocol that allows full trust in the computational results' validity and robustness. With minor edits, main body and Supporting Information can certainly be brought to smooth agreement. Significance. Judging the scientific significance of the findings exceeds my expertise. However, I encourage the authors to clarify the significance of their computational results within the wider scope of their experimental work. To me as a reader, the importance of embedding the well-conducted computational study within this manuscript is not obvious. In it's current form, the manuscript could function as a purely experimental study equally well even if the computational contribution was missing.

We thank the reviewer for this careful assessment of the validity and the presentation of the MD simulations. We are pleased that the reviewer considers the simulations for Pd_2L_4 to be well documented and statistically reliable and that the Supporting Information is viewed as sufficiently detailed to support the robustness of the computational results.
We agree that the description of the $Pd_{12}L_{24}$ simulations in the main text was not sufficiently clear. In particular, we acknowledge that the presentation could give rise to confusion regarding the total number and distribution of simulations. This has been clarified in the revised manuscript. For the $Pd_{12}L_{24}$ system, only one pulling mode is employed with one polyMOS being pulled in each simulation. The 120 simulations in total are distributed across the three force ramps, namely $\dot{k}$ = 5.8 pN · ps^{-1} (10 replicas), 11.7 pN · ps^{-1} (10 replicas) and 17.5 pN · ps^{-1} (100 replicas).
The simulation times depend on the applied force ramp and are therefore not identical for all trajectories. These details are provided in the Supporting Information in Table S8.
We also thank the reviewer for the suggestion to better highlight the significance of the computational work within the overall manuscript. In response, we rearranged the manuscript and revised the discussion of the results to more clearly emphasise the insights provided by the simulations and how these results support the interpretation of the experimentally observed ultrasound-triggered cage disassembly.

R3.4 Data and methodology
II. 501-503 (Methods: Computations)

"A Python-based software that integrates TeraChem69,70 and the FAIRChem package71 was used to perform steered MD and RS-MD, respectively." The authors mention a Python-based software but do not provide more detail. A little more detail is "hidden" in the Supporting Information, p. 122. The mentioned software is an in-house development. encourage the authors to publish their code on github, gitlab, or Zenodo.

We thank the reviewer for this suggestion. We agree that the original description of the in-house Python-based software was too limited. To allow reproducibility, we have now made the code available through Zenodo. The corresponding reference and access information can be found in the revised manuscript in the section "Code availability".

*“**Code availability***

The software Minirauche used for the simulations in this study is publicly available on Zenodo at https://doi.org/10.5281/zenodo.19498996.”

ll. 503-506 (Methods: Computations)

“For the dataset creation, calculations were performed on the level of density functional theory (DFT) employing ...”

The authors do not specify what “the dataset” refers to. The subsequent mentioning of DFT-level computations and ll. 236-238 suggest it is the generation of training data for the fine-tuned MLIP. Please make this clearer.

We thank the reviewer for pointing this out. We agree that the term “the dataset” was too vague in the original version. We have therefore revised the text to clarify that this refers to the dataset generated for fine-tuning the UMA-S MLIP. The revised text now reads:

“For fine-tuning the UMA-S MLIP a dataset of 1,600,332 structures of six different molecular fragments was created. To construct this dataset, calculations were performed on the level of density functional theory (DFT) employing the B3LYP functional[72] using D3-dispersion correction[73] and the 6-31G basis set[74-76] with LanL2DZ effective core potential[77] for Pd^{2+}.”*

ll. 506-507 (Methods: Computations)

“The RS-MD simulations were conducted using a finetuned UMA-S model.”

Do I understand correctly that this fine-tuned model results from MLIP training on the dataset described in the preceding sentence?

We thank the review for this question. The reviewer’s understanding is correct. The fine-tuned UMA-S model used for the RS-MD simulations was obtained from training on the dataset described in the preceding sentence. We have revised to manuscript to make this connection more explicit.

“The RS-MD simulations were conducted using the finetuned UMA-S-PdN model described above”

ll. 513-517 (Data availability)

The authors state that “the Source Data are openly available in Zenodo at https://doi.org/10.5281/zenodo.17939706”. However, in the manuscript they do not state clearly whether this Source Data is raw experimental data, simulation results, or both. The dataset published on Zenodo contains a short description of the contents:

‘This Zenodo record contains data associated with the paper “Mechanochemical disassembly pathways of self-assembled PdnL2n supramolecular architectures.” It includes the fine-tuned Universal Model for Atoms S (UMA-S-PdN) provided as a PyTorch checkpoint (.pt), as well as training/validation/test datasets in ASE LMDB format (.aselmdb). Example trajectories for the two systems studied in this work are provided in XYZ format (.xyz) and include every 100th frame from the original trajectories. Minimal Python code is included to load the .aselmdb files.’

Thus, I understand that the published dataset contains the trained MLIP model used in this study as well as the training data. The short description of file types ‘.aselmdb’ and ‘.xyz’ , however, does not make it very clear how properly access and possibly reuse the provided tar archives. I strongly suggest adding a README file to the data repository that outlines a minimal access protocol for the provided archives.

We thank the reviewer for this helpful suggestion. We agree that the original description of the Zenodo record did not make sufficiently clear which types of data are included and how the archived files can be accessed and reused. In response, we have revised the Zenodo record accordingly and added a README file that provides a concise description of the repository contents as well as minimal instructions for accessing and using the provided .aselmdb and .xyz data.

Analytical approach

The Supporting Information conveys convincingly the statistical robustness of all computational results and the derived properties, such as dissociation forces, fragment frequencies, and activation energies.

We thank the reviewer for this positive assessment of the Supporting Information and for acknowledging the statistical robustness of the computational results.

Suggested improvements

R3.4 ll. 14-17(Abstract)

“Whereas their thermodynamically driven assembly has been extensively studied, controlled strategies for disassembly remain scarce. Here we show that ultrasound provides a powerful stimulus to trigger and probe disassembly pathways.”

vs ll. 429-432 (Conclusion)

“In summary, we have developed a versatile synthetic strategy for polymer-decorated ligands that enables the preparation of metallosupramolecular cage mechanophores across the PdnL2n family.”

When initially skimming abstract and conclusion, the reader might feel confused about the key content of this work. The abstract makes us anticipate a thorough study on disassembly pathways, while the conclusion initially claims a synthesis study.

We appreciate this comment, as clarity of the central message is essential.

“In summary, we have developed a versatile synthetic strategy for polymer-decorated ligands that enables the preparation of metallosupramolecular cage mechanophores across the Pd_nL_{2n} family as well as their mechanochemical disassembly.”

R3.5 ll. 32-74 (Introduction)

The introduction puts the experimental part of this study into context, but not the computational part. I suggest embedding the steered MD simulations into relevant computational literature as well for context

Furthermore, neither abstract nor introduction make clear why molecular dynamics studies are necessary next to the experimental work. What additional insight do we gain from MD that experiments cannot provide?

We agree with the assessment that the computational aspects were underrepresented in the introduction, and have amended this by adding the following section:

“Parallel to experimental advances in mechanochemistry, computational tools have been developed to enable ab initio investigations of force-induced reactions. This is achieved by incorporating an external potential term modelling the mechanical force in addition to the established Born-Oppenheimer potential energy surface, resulting in a force-modified potential energy surface (FM-PES)[62,63]. On this FM-PES, reaction path optimisations and ab initio steered molecular dynamics (AISMD) simulations can provide insight into mechanochemical reactivity, revealing especially the importance of dynamic effects[64-67]. The most significant drawback of AISMD, however, is the comparatively long computational time, which limits the simulation timescale and system size. This has restricted the investigation of force-modified reaction dynamics to mechanophores based on small molecular motifs. While classical force field potentials enable the simulation of very large systems, they do not allow the breakage or formation of bonds, making them unsuited for the study of chemical reactions. Reactive force fields can circumvent this restriction, but their parametrisation is complex and requires considerable expertise[68]. In recent years, machine learning interatomic potentials (MLIPs) have emerged as a powerful alternative for reactive simulations[69,70]. Universal MLIPs such as MACE[71] and FairChem’s Universal Models for Atoms (UMA)[72], trained on large-scale ab initio datasets, can reach accuracy comparable to the quantum chemical training data within their training domain but orders of magnitude faster. This is rapidly expanding the scope of systems that can be studied, enabling large-scale reactive dynamics over extended time scales.”

To better highlight the role of the MD studies in the presented work, the following introductory statement was added to the beginning of the revised computational results section:

"To further elucidate the observed experimental behaviour and access mechanistic details at the molecular level, computational studies were carried out, providing insights into both the qualitative disassembly mechanism and the magnitude of force needed to trigger this process."

R3.6 ll. 231-234 (Computational simulations)

"Simulating the disassembly of PolyMOCs poses a serious challenge for quantum chemical methods due to the large system size, particularly when including solvent molecules explicitly. A tremendous speedup can be achieved when a machine-learnt interatomic potential (MLIP) is used."

The authors make the jump from ab-initio methods to MLIP directly. Why are classical interatomic potentials not an option? What are the benefits of MLIP above classical force field families here?

We thank the reviewer for their question. Since classical interatomic potentials are not suitable to describe bond-breaking reactions in molecular dynamics simulations, it was not an option to employ one, as we study reactivity under the impact of an external force. We added a statement in the introduction, accordingly.

R3.7 ll. 235-238 (Computational simulations)

"FAIRChem's pre-trained Universal Model for Atoms S (UMA-S)67, however, leads to highly asymmetric, deformed structures, which is an indicator for improper description (Supplementary Figure S97).

Therefore, we fine-tuned UMA-S to describe the disassembly of the Pd2L4 PolyMOCs (Supplementary Information, Chapter XII)."

Why is the UMA-S MLIP chosen for this study? What other MLIP are available, and why are they rejected in favor of UMA-S, in particular when the "out-of-the-box" pre-trained UMA-S apparently perform poorly and requires fine-tuning? These question arose when reading the section, and I only found out that the benchmark on pp. 114-115 of the Suppl. Inf. addresses them well later. Consider explicitly stating that these important aspects are discussed there in the main body.

Several computational methods were systematically benchmarked for Pd–N dissociation on a Pd(II)Py_4 model compound (Supplementary Information, Chapter XII, Table S4–S5). Among the tested methods, GFN2-xTB substantially underestimates the dissociation energy (12.6 kcal/mol) and SpookyNet fails to reproduce the dissociation barrier entirely. Pre-trained UMA-S yields dissociation energies (25.0 kcal/mol) and rupture forces (2.41 nN) in good agreement with DFT references (24.8–29.2 kcal/mol depending on functional and basis set). UMA-S was therefore selected as the most promising foundation model, as it is trained on the OMol25 dataset which includes transition metal complexes with variable charge and spin at a high level of theory (ωB97M-V/def2-TZVPD). However, unconstrained optimization of the full Pd2L4 cage produces deformed structures (Supplementary Figure S97), indicating that the pre-trained model does not fully generalize to the global geometry of the assembly. Fine-tuning on DFT reference data for the target system resolved this (Supplementary Information, Chapter XII). The following sentence was added to the main text to direct the reader to this benchmark:

"Benchmarking different potentials using a model Pd^{2+}-complex showed that the energies obtained for restraint optimisations using FAIRChem's pretrained Universal Model for Atoms S (UMA-S) are in good agreement (Supplementary Figure S96)."

R3.8 ll. 296-305 (Fig. 3)

Fig. 3a is superfluous and could be omitted for the sake of brevity. l. 244 eq. 1 states clearly the linearity of pulling force and no visualization if the linear time-dependency is necessary.

I recommend adding a legend to Fig. 3c. The caption clarifies the meaning of blue and purple plots, but labels or a legend embedded in the figure would make it more significant.

I did not understand that Fig. 3b shows top views and Fig. 3c shows side views of the same complex until I saw Figure S97 of the supplementary information. Maybe some coordinate axes along the structures or explanation in the caption would help here.

We thank the review for these helpful suggestions. Figure 3a has been removed from the revised manuscript, as we agree that this panel was not essential and could be omitted for the sake of brevity. We also followed the reviewer's suggestion to improve the readability of Figure 3c (now Figure 5b) and added a legend directly to the figure. In addition, we revised Figure 3b (now Figure 5a) to make the viewing direction clearer by adding a small coordinate system indicating the top-view perspective. The corresponding figure captions were revised accordingly to make the distinction between top view and side view.

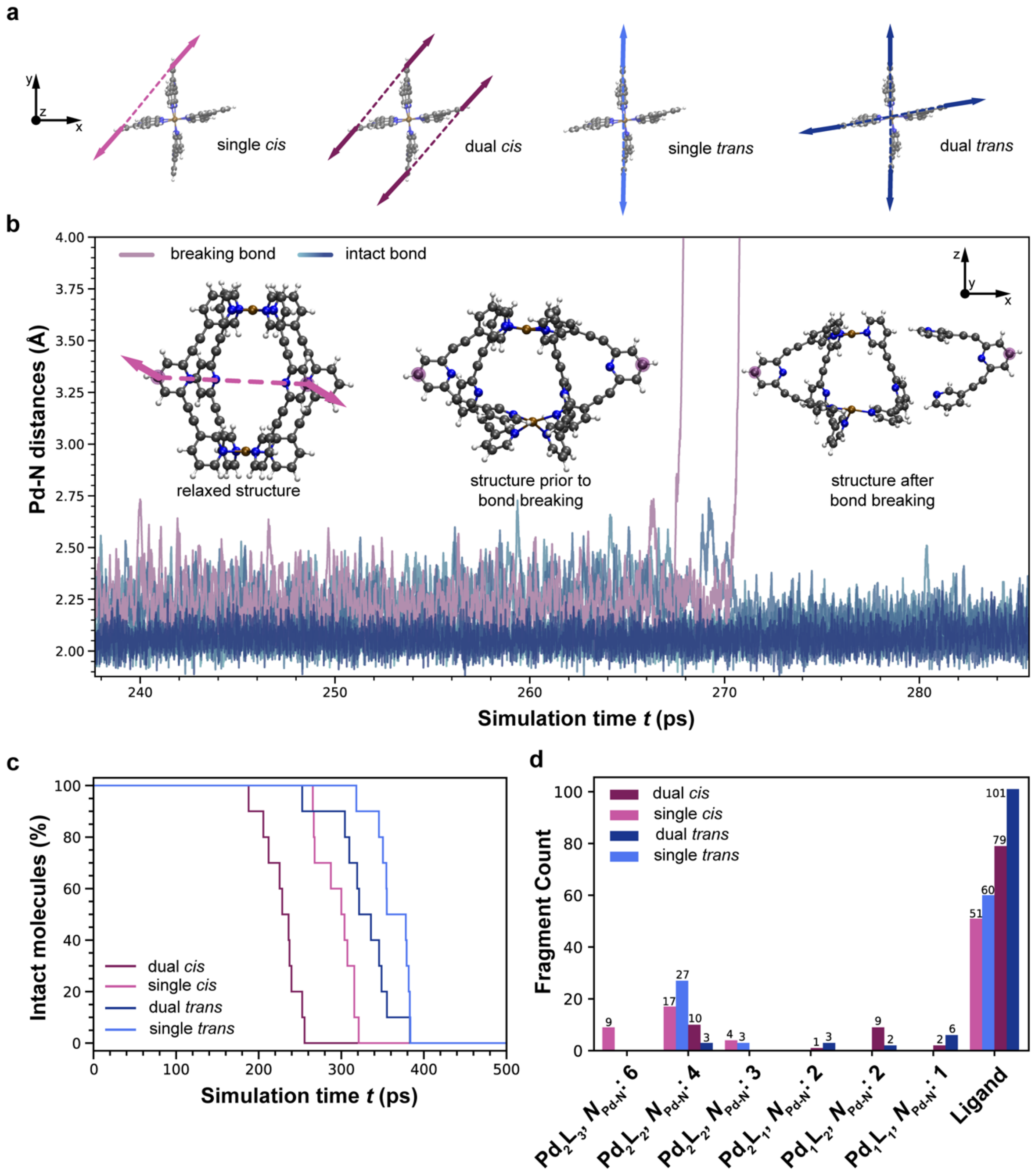

"**Fig. 5 a** *Illustration of the different pulling geometries utilised for RS-MD simulations. The Pd_2L_4-MOC is shown in top view.* ***b*** *Distance-time profiles of all eight Pd–N coordination bonds (single pair cis-pulling, $\dot{k}$ = 9.6 pN · ps^{-1}) of one exemplary RS-MD trajectory. Blue curves: Pd–N bonds that remain intact. Purple curves: Pd–N bonds that undergo scission (distance > 3.0 Å). Included are representative snapshots showing the relaxed cage, the stretched configuration prior to bond breaking and the system after bond scission. Solvent is omitted in the visualisation for clarity. The Pd_2L_4-MOC is presented in*

side view. ***c*** *Survival fraction of intact cages (%,) versus time for the different pulling geometries ($\dot{k}$= 9.6 pN · ps^{-1}).* ***d*** *Fragments observed at the end of the Pd_2L_4 RS-MD simulations. Shown are the results of all trajectories and force ramps, i.e., 30 trajectories each for single trans, single cis, dual cis and dual trans pulling. $N_{Pd\text{-}N}$ indicates the number of Pd–N bonds in the fragment."*

R3.9 **ll. 376-379 (Fig. 5d)**

From the text body and the computational results presented in Fig. 3, it is possible to infer the meaning of the colored arrows in the first panel of Fig. 5d as an illustration of pulling directions. For clarity, a legend and an explanation in the caption should be added to make the figure self-consistent.

We thank the reviewer for this helpful suggestion. In the revised manuscript, Figure 5d is now included in Figure 6 following the reorganization of the manuscript. We revised the corresponding caption to explicitly explain the meaning of the coloured arrows and their relation to the pulling directions.

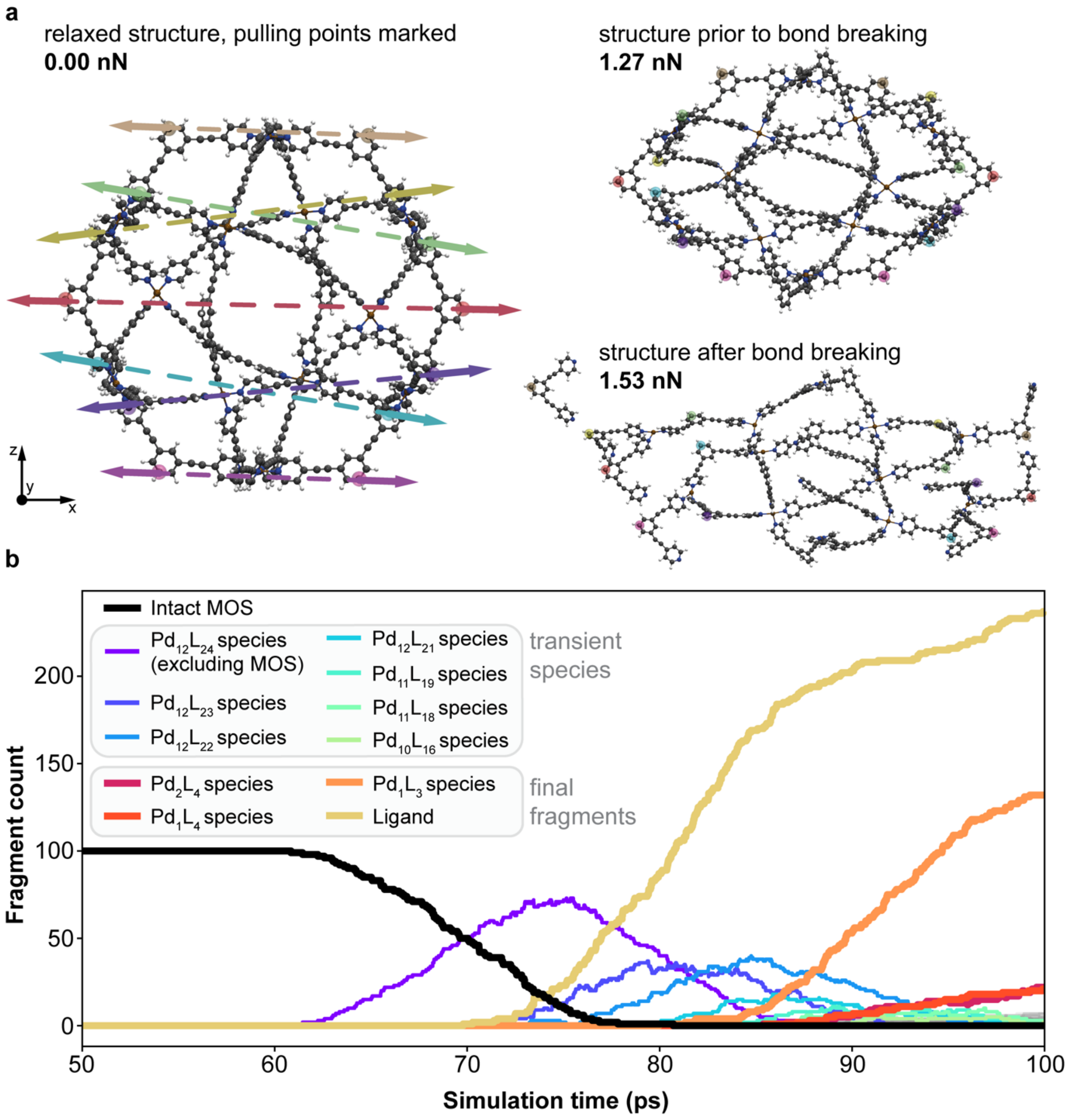

*"**Fig. 6 a** Snapshots from RS-MD simulations of $Pd_{12}L_{24}$ in acetonitrile with multiple pulling pairs ($\dot{k}$ = 17.5 pN · ps^{-1}; solvent is omitted for clarity). Three representative structures picked from one trajectory: a relaxed structure (left), a structure prior to bond breaking (top right) and a structure after bond scission (bottom right). The coloured arrows indicate the multiple pulling pairs on which force is applied.*

***b** Fragmentation of $Pd_{12}L_{24}$ MOS over time during RS-MD simulations using a force ramp of 17.5 pN · ps^{-1}. Species of varying connectivity are grouped by Pd_XL_Y compositions (Supplementary Figure S109-110, Supplementary Table S17). Fragments that occur less than ten times in the 100 simulations are marked in grey."*

<u>R3.10</u> ll. 402-417

More computational results are "hidden" here as a little appendix to an otherwise experimental section. I almost overlooked them. Why are they not presented on the same level of prominence as the previous computational section of l. 230ff, in particular as the methodology parallels this previous section exactly? Could it be beneficial for the structure of the whole manuscript to present the Pd2L4 and Pd12L24 simulation results together? From my point of view as a reader, the latter merely adds another dimension, the actual molecular structure, to the previously explored three-dimensional parametric space of pulling mode, pulling force, and replica.

We thank the reviewer for this helpful and constructive comment. We agree that the presentation of the $Pd_{12}L_{24}$ computational results in the original manuscript was not sufficiently prominent. In the revised manuscript we therefore reorganized the computational section and now present the Pd_2L_4 and $Pd_{12}L_{24}$ results together.

It is unclear how the mentioned 120 runs have been distributed across the three different pulling forces, pulling modes, and replicas.

Simulation times are not stated explicitly. Fig. 6 implies 100 ps.

We thank the reviewer for pointing out that the description is unclear. We clarified the distribution of the 120 $Pd_{12}L_{24}$ simulations in the main text. Since only one pulling setup was used for this system, the simulations are distributed across the three force ramps as follows: $\dot{k}$ = 5.8 pN · ps^{-1}: 10 simulations, 11.7 pN · ps^{-1}: 10 simulations and 17.5 pN · ps^{-1}: 100 simulations. In addition, we now explicitly state that simulation times depend on the applied force ramp and are listed in Tables S7 and S8. We further clarified that the 100 ps shown in Figure 6 specifically refer to the trajectories for the 100 simulations at $\dot{k}$ = 17.5 pN · ps^{-1}. Not that the simulation times listed in the Supporting Information include the additional 20 ps of thermalization period.

"Force was applied to seven pulling pairs in total (Figure 6a, Supplementary Figure S104b). Simulations were performed using $\dot{k}$ = 5.8 pN · ps^{-1} (10 simulations), 11.7 pN · ps^{-1} (10 simulations) and 17.5 pN · ps^{-1} (100 simulations). The simulation times depend on the applied force ramp and are listed in Table S8 of the Supporting Information."

"Analysing the fragmentation for the 100 simulations employing a force ramp of $\dot{k}$ = 17.5 pN · ps^{-1} reveals that although a large diversity of fragments is formed during the simulations, in particular Pd_{11} and Pd_{10} species, the most frequently found fragments at the end of the simulations are the free ligand, Pd_2L_4, Pd_1L_4, and Pd_1L_3 (Figure 6)."

<u>R3.11</u> ll. 422-423 (Fig. 6)

"Fragments that occur less than ten times in the 100 simulations are marked in grey."

100 simulations mentioned here contradict 120 simulations mentioned on l. 402.

We thank the reviewer for raising this point. We want to point out that Figure 6 only shows the fragmentation of the 100 simulations conducted with a force ramp of 17.5 pN ps^{-1}. The additional 20 simulations mentioned on l. 402 were run with the other two force ramps. We now explicitly stated in the main text that 100 simulations were conducted using a force ramp of 17.5 pN · $ps^{-1.}$

"Analysing the fragmentation for the 100 simulations employing a force ramp of $\dot{k}$ = 17.5 pN · ps^{-1} reveals that although a large diversity of fragments is formed during the simulations, in particular Pd_{11} and Pd_{10} species, the most frequently found fragments at the end of the simulations are the free ligand, Pd_2L_4, Pd_1L_4, and Pd_1L_3 (Figure 6)."

<u>R3.12</u> ll. 501-503 (Methods: Computations)

"A Python-based software that integrates TeraChem69,70 and the FAIRChem package71 was used to perform steered MD and RS-MD, respectively."

Steered MD and RS-MD are mentioned in parallel. This looks like a tautology to me. In the context of this work, I do not understand the distinction here. Only RS-MD (ramped, steered molecular dynamics) results have been presented in the main body. These are, of course, steered.

The Supporting Information describes steered MD runs that are not ramped. They are, however, not presented in the main body.

We thank the reviewer for pointing out that the distinction between steered MD and ramped steered MD is unclear. First of all, the steered MD runs that are described in the Supporting Information but not in the main body were performed to create the dataset for fine-tuning the UMA-S MLIP. Since we do not rigorously describe the process of data set generation in the main body, we do not mention these steered MD simulations there. However, in our manuscript we distinct between steered MD and ramped steered MD to point out that we simulated a time-dependent force in our simulations and not a constant one as you would in classical steered MD.

Supporting information, Benchmarking, p. 114

"The total charge of the system is +2"

How is a system of non-neutral charge handled in your MD simulations?

The UMA-S model in OMOL mode accepts the total system charge and spin multiplicity as explicit input parameters. These are embedded as global features that condition the predicted potential energy surface via the Mixture of Linear Experts architecture (Wood et al., arXiv:2506.23971).

Supporting information, Generation of ML training data, p. 116

"To construct the dataset for fine-tuning UMA-S, steered MD simulations for six different molecular fragments were conducted (Figure S98). The steering was necessary to increase the diversity of the dataset and to include the effects of a pulling force on molecular structures in the dataset."

Why was it necessary to include steering for increased diversity? What's the evidence that the quality of the training dataset would have been poor without pulling?

We thank the reviewer for this important comment. Our original statement was intended to convey that the steered trajectories increased the diversity of the fine-tuning dataset relative to the 0 nN simulations. However, we agree that this wording was unclear and did not sufficiently explain why this additional diversity is important in the present context. In this work, the increased diversity does not simply refer to a broader set of arbitrary conformations, but specifically to the inclusion of force-loaded, highly strained, and reaction-relevant structures generated under steered molecular dynamics. These configurations are sampled much less frequently in unbiased 0 nN simulations. Since our goal was to fine-tune UMA-S particularly for mechanochemically relevant processes, it was essential that the training set also covers these mechanically perturbed regions of configuration space. We have therefore revised the Supporting Information to clarify that the role of steering was not merely to increase diversity in a general sense, but specifically to extend the sampling into the force-bearing and reactive configurations relevant to mechanochemical transformations.

"Steering was necessary to enrich the dataset with force-loaded molecular configurations and the associated structural distortions that arise under pulling conditions."

Supporting information, Generation of ML training data, Figure S98, p.117

What do the numbers N in the figure caption represent?

Supporting information, Fine tuning UMA-S, p. 119

"... showing very good agreement with the DFT (B3LYP+D3/6-31G*) reference (Figure S5)."

The reference to Figure S5 seems to be wrong.Fig. S100?

We thank the reviewer for pointing this out. We checked the reference and corrected the Figure number.

"…showing very good agreement with the DFT (B3LYP+D3/6-31G) reference (Figure S100)."*

Supporting information, Fine tuning UMA-S, p. 120

"Both the MLIP NEB and the DFT energies are shown in Figure SX."

Again, likely wrong Figure reference. Fig. S101?

We thank the reviewer again for pointing towards the wrong Figure reference. Again, we corrected the reference.

"Both the MLIP NEB and the DFT energies are shown in Figure S101."

Supporting information, RS-MD, p. 121

"Rather than applying an external force of constant strength, we the external force applied in the simulation is gradually increased ..."

Remove "we"

We thank the reviewer for pointing this out. In the revised Supporting Information, we removed the "we".

"Rather than applying an external force of constant strength, the external force applied in the simulation is gradually increased"

Supporting information, RS-MD, p. 122

"All RS-MD simulations were performed using the UMA-S-PdN described in the previous section. The simulations were run in an NVT ensemble using the Bussi-Parrinello version of Langevin dynamics43 with a timestep of 1 fs, a thermostat temperature of 300 K, and a friction coefficient of 7 ps-1."

These are important parameters of the simulation protocol and I recommend placing them in the main body's "Methods: Computations" section. Also, I recommend replacing the term "friction coefficient" with "thermostat damping parameter" or similar, since this does not refer to a true, mechanical friction coefficient.

We thank the reviewer for this helpful suggestion. We agree that these are important simulation parameters and should be stated in the main "Methods: Computations" section. Accordingly, we have included them in the revised manuscript, while retaining them in the Supporting Information for completeness. We have also replaced the term "friction coefficient" with "thermostat damping parameter".

"The simulations were run in an NVT ensemble using the Bussi-Parrinello version of Langevin dynamics[88] with a timestep of 1 fs, a thermostat temperature of 300 K, and a thermostat damping parameter of 7 ps^{-1}."

Supporting information, RS-MD simulations – setup, Table S6, p. 126

Is the no. of 10 trajectories for ramping rate 17.5 nN/ps reported wrong? Table S11 implies 100 runs.

We thank the reviewer for catching this inconsistency. The reported number of trajectories for the force ramp of 17.5 pN · ps^{-1} in Table S8 was indeed incorrect. This has now been corrected in the revised Supporting Information. We performed 100 simulations for 17.5 pN · ps^{-1}.

Table S8: Settings of the RS-MD simulations investigating the multi-pulling mode of $Pd_{12}L_{24}$. The maximum simulation time includes 20 ps of thermal equilibration before ramping of the external force was initialized.

Ramping rate $\dot{k}^{\dagger}$ (pN/ps)	maximum simulation time (ps)	no. of trajectories
0.0	120	5
5.8	320	10
11.7	220	10
17.5	120	100

† applied per pulling pair

R3.13 Clarity and context

The manuscript is written clearly and, for the computational part, I understand very well what has been done. Yet, I have difficulties understanding why it has been done. As mentioned above, I believe the manuscript would benefit profoundly if the computational results were contextualized properly within the otherwise experimental scope.

We thank the reviewer for this important comment. We agree that the motivation and role of the computational results within the overall manuscript should be stated more clearly. The purpose of the simulations is to provide mechanistic insight that is difficult to obtain experimentally, in particular with respect to the effect and magnitude of ultrasound-induced pulling forces at the molecular level. We therefore performed computational investigations under external pulling force in order to probe qualitative disassembly mechanism and to estimate the force range required to trigger this process. We have revised the manuscript accordingly to make this motivation and the connection to the experiment more explicit.

"As the effect and magnitude of ultrasound-induced pulling forces at the molecular level is difficult to assess experimentally, we conducted a computational investigation of the synthesised systems under external pulling force. This provides insights into both the qualitative disassembly mechanism and the magnitude of force needed to trigger this process."

Moreover, I believe the second batch of computational results on Pd12L24 is obscured a little and would benefit from a presentation on par with the Pd12L24 results.

We also sincerely thank the reviewer for the suggestion to rearrange the manuscript and combine the presentation of the results on Pd_2L_4 and $Pd_{12}L_{24}$. We agree that the original manuscript did not present the results on $Pd_{12}L_{24}$ with sufficient prominence. In response, we reorganized the manuscript and now present the Pd_2L_4 and $Pd_{12}L_{24}$ computational results together.

R3.14 References

The manuscript does not attempt to embed its computational contribution into relevant literature.

We thank the reviewer for pointing out that the manuscript lacks a proper embedding in the relevant computational literature. We therefore revised the manuscript and included a section to do so.

R3.15 Your expertise

I am trained in the disciplines of physics and engineering sciences with a heavy focus on computational methods. I specialize on computational nanotribology, with a specific methodological focus on molecular dynamics simulations of interfacial phenomena and FAIR data. Hence, I see myself in a valid position

to evaluate the modeling and data-handling aspects of this manuscript. I have no chemistry background, hence commenting on the manuscript's experimental merits would exceed my expertise.

Hence, my review specifically targets the main body's Abstract(ll. 12-30), Introduction (ll. 32-74), Results and Discussion: Computational simulations of PolyMOC disassembly (ll. 230-305, inc. Fig. 3), an additional computational section without separate heading (ll. 402-427, inc. Fig. 6), Conclusion (ll. 429-453), Methods: Computations (ll. 501-507), and Data Availability (ll. 513-517) as well as the Supporting Information's chapter XII: Molecular dynamics simulations and computational analysis of PdnL2n disassembly (p. 114-139).

Reviewer #4 (Remarks to the Author):

R4.1 I co-reviewed this manuscript with one of the reviewers who provided the listed reports. This is part of the Nature Communications initiative to facilitate training in peer review and to provide appropriate recognition for Early Career Researchers who co-review manuscripts.

We sincerely thank Reviewer 4 for their contribution to the evaluation of our manuscript and for their engagement in this review process. We highly appreciate the *Nature Communications* initiative to facilitate training in peer review and to provide appropriate recognition for early career researchers who co-review manuscripts.
